# Azulenesulfonium Salts: Accessible, Stable, and Versatile Reagents for Cross‐Coupling

**DOI:** 10.1002/anie.201510666

**Published:** 2016-01-14

**Authors:** Paul Cowper, Yu Jin, Michael D. Turton, Gabriele Kociok‐Köhn, Simon E. Lewis

**Affiliations:** ^1^ Department of ChemistryUniversity of BathBathBA2 7AYUK; ^2^ Chemical Characterisation and Analysis FacilityUniversity of BathBathBA2 7AYUK

**Keywords:** azulenes, sulfonium salts, sulfoxides, Suzuki–Miyaura cross-coupling, synthetic methods

## Abstract

Azulenesulfonium salts may be readily prepared from the corresponding azulenes by an S_E_Ar reaction. These azulene sulfonium salts are bench‐stable species that may be employed as pseudohalides for cross‐coupling. Specifically, their application in Suzuki–Miyaura reactions has been demonstrated with a diverse selection of coupling partners. These azulenesulfonium salts possess significant advantages in comparison with the corresponding azulenyl halides, which are known to be unstable and difficult to prepare in pure form.

Azulene (**1**) is a non‐alternant aromatic hydrocarbon which has fascinated chemists for many years owing to its blue color and high dipole moment.^1^ Substituted azulenes have been employed in diverse contexts, including medicinal chemistry (as antiulcer,^2^ antidiabetic,^3^ anticancer,^4^ antiarrhythmic,^5^ and anti‐erectile‐dysfunction^6^ agents, and as TXA_2_ τ receptor antagonists^7^), solar cells,^8^ metal–organic frameworks for hydrogen storage,^9^ and organic electronics,^10^ among others. Uses of azulenes in stimuli‐responsive systems have also been disclosed, most commonly in halochromic materials,^11^ but also in probes for soft metal cations,^12^ fluoride,^13^ other anions,^14^ and biomolecule analytes.^15^ Furthermore, the ability to tune the absorption and emission maxima of azulenes by attaching conjugated substituents^16^ has led to applications in bioimaging and fluorescence.^17^

In all of the above instances, the ability to introduce substituents onto the azulene skeleton in a controlled manner is crucial. Substitution at the azulene 1‐ and 3‐positions has been most extensively explored, since these positions are the most reactive in S_E_Ar reactions. In certain specific cases the desired substituent may be installed directly in one step by such an S_E_Ar process.^18^ Alternatively, cross‐coupling methodologies should allow access to a much wider range of substituted azulenes. The reactivity described above suggests that the treatment of azulenes with an electrophilic halogen source should readily furnish 1‐haloazulenes for use in such cross‐coupling reactions. However, in reality this approach suffers from serious drawbacks. Thus, the treatment of azulene with one equivalent of *N*‐halosuccinimide gives the desired 1‐haloazulene **2** always as a mixture with the corresponding 1,3‐dihaloazulene **3**, as a consequence of the enhanced reactivity of the initial product **2**. Furthermore, such (di)haloazulenes are unstable to varying degrees. Ordinarily, the mixture of products of chlorination/bromination may be isolated, but decomposes if separation is attempted by chromatography on silica; products of iodination typically decompose upon removal of the solvent, but can sometimes be used as solutions. The inability to access electrophilic coupling partners has severely hampered the development of azulene cross‐coupling. Indeed, these problems of separation and stability have been explicitly commented on previously on numerous occasions.^19^

Various attempts to circumvent the problems detailed above have been described, but all have restrictions of their own. For example, mixtures of 1‐halo and 1,3‐dihaloazulenes have been taken forward crude into coupling reactions, but with the consequence that doubly coupled products or higher oligomers also form; these by‐products can be difficult to remove (Scheme 1 a).19b,19d, 20 There are limited examples of the use of other azulene derivatives (i.e. pseudohalides) as electrophilic cross‐coupling partners. The preparation and coupling of 1‐trifloxyazulenes has been reported,^21^ but these coupling partners were themselves unstable and required specific reaction conditions (Scheme 1 b).

**Scheme 1:**
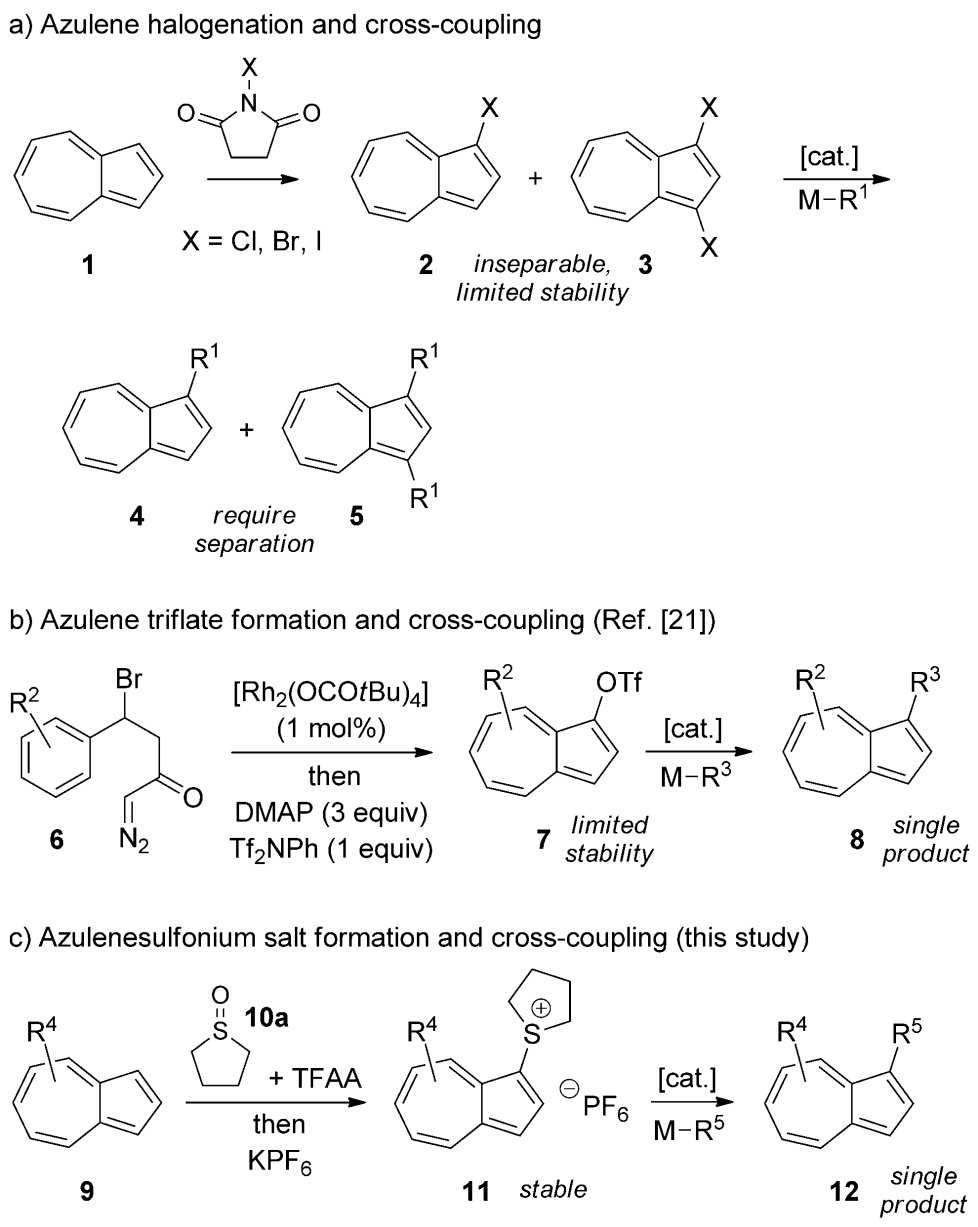
Strategies for azulene cross‐coupling. DMAP=4‐dimethylaminopyridine, Tf=trifluoromethanesulfonyl.

In an attempt to improve on the state of the art in azulene cross‐coupling, we have investigated the applicability of azulenesulfonium salts as novel pseudohalide electrophilic coupling partners (Scheme 1 c). Liebeskind and co‐workers have introduced sulfonium salts as powerful electrophilic reagents for cross‐coupling,^22^ but they have not previously been applied in the context of azulene chemistry.^23^ The results of our studies are described herein.

Of various possible routes to azulenesulfonium salts, we discounted the approach of double alkylation of the thiol, since 1‐azulenethiol is itself unstable and hard to access.^24^ Instead, we adapted a procedure reported by Shoji et al.23a and used a sulfoxide and an activating agent. Thus, the treatment of azulene (**1**) with inexpensive tetramethylene sulfoxide (**10 a**) and trifluoroacetic anhydride (TFAA), followed by anion exchange and recrystallization, gave the novel azulenesulfonium salt **11 a** (Scheme 2).

**Scheme 2:**
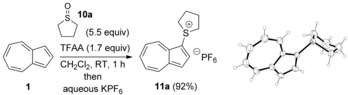
Synthesis of the parent sulfonium salt **11 a** and its X‐ray crystal structure.

Salt **11 a** is a purple crystalline solid with good stability: We have stored it for months at ambient temperature, with no attempt to exclude air, moisture, or light, without observing degradation. No evidence of 1,3‐disubstitution was observed, in keeping with our expectation, given that **11 a** is much less electron rich than **1**. With the prospective coupling partner **11 a** in hand, we sought to determine its reactivity in a representative Suzuki–Miyaura coupling reaction, with the XPhos ligand developed by Buchwald and co‐workers^25^ and 4,4,5,5‐tetramethyl‐2‐(*p*‐tolyl)‐1,3,2‐dioxaborolane (**12 a**) as the nucleophilic partner (Scheme 3). We first explored the choice of solvent, by using solvents in which **11 a** showed a degree of solubility. Reaction conversion was determined by the integration of ^1^H NMR spectra recorded in the presence of 1,4‐dimethoxybenzene as an internal standard (Table 1).

**Scheme 3:**
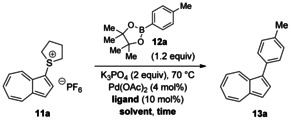
Optimization of cross‐coupling parameters.

**Table 1:** Effect of the solvent on the Suzuki–Miyaura coupling.^[a]^

Entry	Solvent	Solubility of **11 a**	Conversion [%]
1	2‐propanol	sparingly soluble	25
2	THF	sparingly soluble	40
3	2‐MeTHF	sparingly soluble	0
4	1,4‐dioxane	sparingly soluble	0
5	DMF	fully soluble	86
6	MeCN	fully soluble	53
7	acetone	fully soluble	52

[a] Reactions were carried out with the ligand 2‐dicyclohexylphosphino‐2′,4′,6′‐triisopropylbiphenyl (XPhos); the reaction time was 4 h. DMF=*N*,*N*‐dimethylformamide, 2‐MeTHF=2‐methyltetrahydrofuran.

The solubility of **11 a** was a key determinant of reaction progression; the three reactions for which the highest conversion was observed were carried out in solvents in which **11 a** was wholly soluble at the reaction concentration of 0.14 m (Table 1, entries 5–7). DMF afforded the highest conversion after 4 h (Table 1, entry 5). In the case of 2‐propanol (Table 1, entry 1), the low conversion was accompanied by the formation of traces of azulene (**1**). We next sought to evaluate the extent to which the ligand could influence the reaction progression (see Table S1 in the Supporting Information). A shorter reaction time was used for the ligand screen, and bulky monodentate biaryl phosphines led to a greater reaction rate and hence greater conversion after 2 h (albeit with the exception of *t*Bu‐BrettPhos). Chelating phosphines afforded inferior conversion, as did triaryl and trialkyl phosphines. A subsequent screen of bases did not identify a base which reliably afforded greater conversion than potassium phosphate.

Having evaluated the effects of the various reaction parameters, we next sought to apply the methodology with a variety of organoboron cross‐coupling partners, and to isolate the azulenes produced. Although various bulky biaryl phosphine ligands had effected faster conversion than XPhos, at this point we returned to the use of XPhos (in conjunction with a longer reaction time) for reasons of economy (Scheme 4; Table 2). The azulene Suzuki–Miyaura products **13 a**–**n** were isolated in moderate to good yield. In some cases it was found that the use of a boronic acid reagent led to the formation of a quantity of the corresponding boroxine cyclotrimer, which could coelute with the desired product, so pinacolboranes were sometimes employed in preference. A wide variety of functionality was tolerated, including free alcohol and aldehyde groups, as well as electron‐poor and electron‐rich heterocycles.

**Scheme 4:**
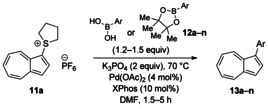
Variation of the organoboron coupling partner.

**Table 2:** Synthesis of azulene derivatives from **11 a** and different organoboron coupling partners.

Organoboron reagent	Azulene product	Yield [%]^[a]^
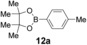	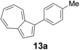	60^[b]^
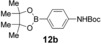	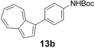	63^[b]^
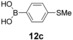	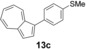	53^[b]^
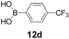	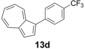	47^[c]^
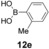	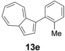	38^[b]^
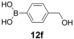	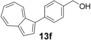	47^[c]^
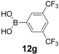	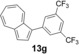	63^[b]^
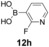	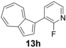	57^[b]^
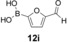	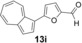	63^[b]^
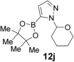	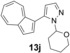	58^[b]^
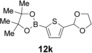	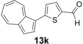	60^[b,d,e]^
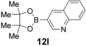	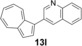	55^[b]^
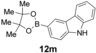	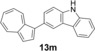	56^[b]^
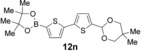	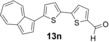	48^[b,e]^

[a] Yield of the isolated product after chromatography. [b] The product was purified on silica. [c] The product was purified on neutral alumina. [d] The reaction was carried out in *i*PrOH. [e] The yield is for two steps: cross‐coupling and acetal deprotection. Boc=*tert*‐butoxycarbonyl.

We next assessed the effects of substitution on the azulene coupling partner. Accordingly, analogues of **11 a** were prepared from substituted azulenes (Table 3) and then cross‐coupled with organoboron reagents (Table 4). In the case of guaiazulene (**9 d**), the corresponding sulfonium salt formed with **10 a** did not crystallize as readily as the others. Thus, **9 d** was treated with dimethyl sulfoxide (**10 b**) instead to give the alternative sulfonium salt **11 d**;^26^ this compound was also competent in cross‐coupling. Yields of the cross‐coupling reactions again ranged from moderate to good. Some aldehyde‐containing products (compounds **13 k**,**n**,**o**,**q**,**r**,**s**) were produced in a two‐step process involving the coupling of a pinacolborane reagent in which the aldehyde was protected as an acetal, followed by hydrolytic deprotection. However, other such products (compounds **13 i**,**p**,**t**,**u**,**v**) were prepared by direct cross‐coupling of a pinacolborane comprising a free aldehyde group. In particular, the formation of **13 v** is notable, since it is the product of two successive sulfonium‐formation/cross‐coupling cycles, and was accessed from azulene in four steps; this example highlights the applicability of this methodology to the preparation of multiply‐substituted azulenes that would be difficult to access by other methods.

**Table 3:** Preparation of sulfonium salts from substituted azulenes **9**. 
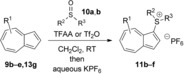

Substituted azulene	Product sulfonium salt and X‐ray structure		Yield [%]
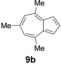	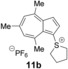	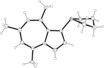	96^[a]^
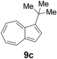	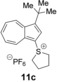	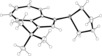	95^[a]^
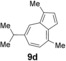	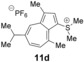	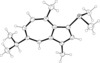	68^[a]^
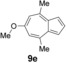	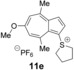	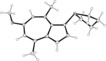	91^[a]^
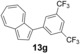	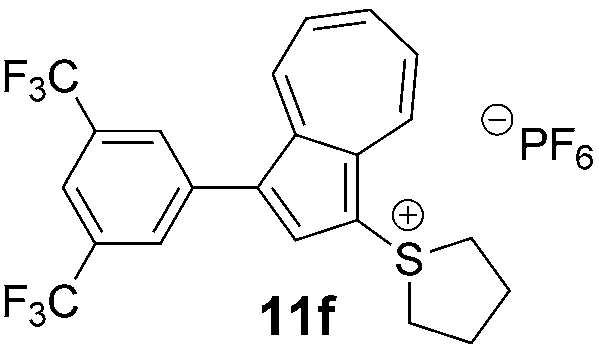	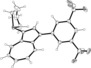	73^[b]^

[a] TFAA was used as an activating agent. [b] Tf_2_O was used as an activating agent.

**Table 4:** Scope of the reaction with respect to the azulenesulfonium coupling partner: Preparation of novel azulenes from **11 b**–**f**. 
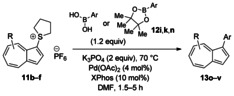

Sulfonium salt	Organoboron reagent	Product	Yield [%]^[a]^
**11 b**	**12 k**	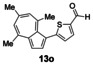	68^[b,c]^
**11 b**	**12 i**	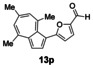	50
**11 b**	**12 n**	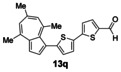	59^[b,c,d]^
**11 c**	**12 k**	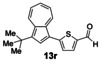	73^[b,c]^
**11 c**	**12 n**	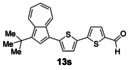	82^[b,c,d]^
**11 d**	**12 i**	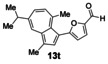	37
**11 e**	**12 i**	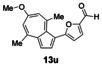	81
**11 f**	**12 i**	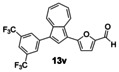	23

[a] Yield of the isolated product after chromatography. Products **13 o**–**v** were purified on silica. [b] The reaction was carried out in *i*PrOH. [c] The yield is for two steps: cross‐coupling and acetal deprotection. [d] 2‐Dicyclohexylphosphino‐2′,6′‐dimethoxybiphenyl (SPhos) was used instead of XPhos.

In summary, we have introduced azulenesulfonium salts as electrophilic reagents for cross‐coupling. These reagents have several distinct advantages over the corresponding halides, namely, more straightforward preparation and purification, as well as greatly enhanced stability.

## Supporting information

As a service to our authors and readers, this journal provides supporting information supplied by the authors. Such materials are peer reviewed and may be re‐organized for online delivery, but are not copy‐edited or typeset. Technical support issues arising from supporting information (other than missing files) should be addressed to the authors.

Supplementary
