# Supplementary material for: Azulenesulfonium Salts: Accessible, Stable, and Versatile Reagents for Cross‐Coupling
Source: Angew Chem Int Ed Engl. 2016 Jan 14;55(7):2564–8. doi: 10.1002/anie.201510666 (PMC4755203; doi:10.1002/anie.201510666)
Supplement: Supplementary file 1 — Supplementary [file ANIE-55-2564-s001.pdf]

Angewandte Chemie
Eine Zeitschrift der Gesellschaft Deutscher Chemiker

Supporting Information

Azulenesulfonium Salts: Accessible, Stable, and Versatile Reagents for Cross-Coupling

Paul Cowper, Yu Jin, Michael D. Turton, Gabriele Kociok-Köhn, and Simon E. Lewis**

anie_201510666_sm_miscellaneous_information.pdf

General Synthetic Procedures . . . . . . Page S2
Ligand Screen . . . . Page S3
Individual procedures and spectroscopic data . . . Page S4
NMR Spectra . . . . Page S19
X-Ray Crystallography – overview . . . . . Page S178
X-Ray Crystallographic data for **11a** . . . . . Page S179
X-Ray Crystallographic data for **11b** . . . . . Page S185
X-Ray Crystallographic data for **11c** . . . . . Page S194
X-Ray Crystallographic data for **11d** . . . . . Page S201
X-Ray Crystallographic data for **11e** . . . . . Page S208
X-Ray Crystallographic data for **11f** . . . . . Page S215

General Synthetic Procedures and Instrumentation. Reactions were carried out under an atmosphere of argon. Dichloromethane, hexane and diethyl ether were dried and degassed by passing through anhydrous alumina columns using an Innovative Technology Inc. PS-400-7 solvent purification system[1]. All solvents used in Suzuki–Miyaura couplings were degassed through a series of freeze-thaw cycles. Petrol refers to petroleum ether, bp 40-60 °C. TLCs were performed using aluminum-backed plates precoated with Alugram®SIL G/UV or aluminum backed plates precoated with Alugram®ALOX N/UV 254nm and visualized by UV light (254 nm) and/or $KMnO_4$ followed by gentle warming. Flash column chromatography was carried out using Alumina (activated neutral, Brockmann I) purchased from Sigma-Aldrich or Davisil LC 60Å silica gel (35-70 micron) purchased from Fisher Scientific. All reagents were purchased from the Sigma-Aldrich Chemical Co., Alfa Aesar, Tokyo Chemical Industries or Fisher Scientific Ltd. and were used without further purification. X-Phos refers to 2-dicyclohexylphosphino-2',4',6'-tri*iso*propylbiphenyl. S-Phos refers to 2-dicyclohexylphosphino-2',6'-dimethoxybiphenyl. *p*TSA refers to *para*-toluenesulfonic acid monohydrate. IR spectra were recorded on Perkin-Elmer Spectrum 1600 FT IR spectrometer with universal ATR sampling accessory, with absorbances quoted as ν in cm^{-1}. NMR spectra were run on an Agilent ProPulse 500 MHz instrument or on Bruker Avance 300, 400 or 500 MHz instruments at 298 K, unless otherwise specified. Mass spectra were recorded with a micrOTOF electrospray time-of-flight (ESI-TOF) mass spectrometer (Bruker Daltonik).

Ligand Screen for coupling of 11a and 12a

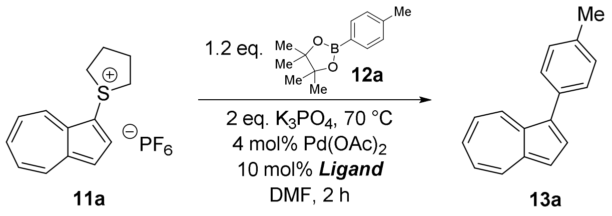

Table S1. Effect of ligand on Suzuki–Miyaura coupling.[a]

Entry	Ligand	Conversion
1	X-Phos	38%
2	S-Phos	51%
3	(Cy)-JohnPhos	61%
4	(Cy)-BrettPhos	59%
5	(*t*Bu)-BrettPhos	13%
6	(*t*Bu)-JohnPhos	54%
7	DavePhos	54%
8	TrixiePhos	73%
9	dppf[b]	12%
10	dppe	13%
11	dppp	13%
12	BINAP	20%
13	PPh_3	12%
14	PCy_3	33%

[a] 1,4-Dimethoxybenzene was used as an internal standard, with conversions being determined by ^{1}H-NMR integration. [b] 4 mol% $Pd(dppf)(Cl)_2$ used instead of $Pd(OAc)_2$ and a ligand.

*Note that entry 1 in **Table S2** is the same ligand as was used in entry 5, **Table 1**, but the reaction time is shorter.*

1-(Azulen-1-yl) tetrahydrothiophenium hexafluorophosphate (11a)

Azulene (1.28 g, 10.0 mmol, 1.0 equiv) was added into a 500 mL round bottom flask containing a magnetic stirrer bar. The flask was evacuated and filled with N_2 gas. CH_2Cl_2 (50 mL), and tetrahydrothiophene-1-oxide (5.00 mL, 55.6 mmol, 5.5 equiv) were added to the flask and the solution was stirred for 10 min. Trifluoroacetic anhydride (TFAA) (2.08 mL, 15 mmol, 1.5 equiv.) was dissolved in CH_2Cl_2 (50mL) and the solution was transferred to the reaction mixture, which was stirred for 1.5 h at room temperature. The reaction mixture was washed with water (3 × 200 mL). To the aqueous layer was added potassium hexafluorophosphate (7.20 g, 40 mmol, 4 equiv). The aqueous layer was washed with CH_2Cl_2 (3 × 150 mL). The organic layer was dried over $MgSO_4$ and filtered, then the filtrate was concentrated *in vacuo*. The crude product was purified by recrystallization in DCM/petroleum ether to afford **11a** as a purple crystalline solid (3.11 g, 9.16 mmol, 92%); m.p. 188 °C; ^{1}H NMR (500 MHz, acetone-d_6) δ (ppm) 9.01 (d, J = 9.8 Hz, 1H) 8.87 (d, J = 9.8 Hz, 1H) 8.45 (d, J = 4.5 Hz, 1H) 8.21 (t, J = 10.0 Hz, 1H) 7.89 (t, J = 9.8 Hz, 1H) 7.85 (t, J = 9.8 Hz, 1H) 7.76 (d, J = 4.5 Hz, 1H) 4.31-4.26 (m, 2H) 3.90-3.82 (m, 2H) 2.89-2.84 (m, 2H) 2.62-2.55 (m, 2H); ^{13}C NMR (126 MHz, acetone-d_6) δ (ppm) 145.1, 142.9, 142.6, 141.8, 136.9, 136.5, 129.8, 129.5. 121.8, 105.7, 51.3, 29.9; IR (neat) 2955, 1587, 1406, 819 cm$^{-1;}$ HRMS (ESI+) *m/z* calcd. for ($C_{14}H_{15}S$) $[M]^+$ 215.0889; found 215.0886.

1-(4,6,8-Trimethylazulen-1-yl) tetrahydrothiophenium hexafluorophosphate (11b)

To 4,6,8-trimethylazulene (1.36 g, 8.00 mmol, 1.0 equiv) in CH_2Cl_2 (80 mL) at room temperature was added tetrahydrothiophene-1-oxide (5.3 mL, 60 mmol, 7.5 equiv). The reaction mixture was stirred for 10 min, then a 0.5 mol dm^{-3} solution of TFAA in CH_2Cl_2 was added dropwise, until TLC indicated consumption of starting material (approx. 12 mmol, 1.5 equiv. required). The reaction mixture was diluted with water in a separating funnel and hexane was added to the organic phase to induce the product to move into the aqueous phase. The phases were separated, and KPF_6 (5.89 g, 32.0 mmol, 4.0 equiv) was added to the aqueous phase. The product was then back-extracted into CH_2Cl_2. This organic layer was then dried over $MgSO_4$ and filtered. The filtrate was concentrated *in vacuo* and the resultant solid was triturated with several portions of Et_2O to give **11b** as a bright red crystalline solid (3.09 g, 96%); m.p. 186 °C; ^{1}H NMR (500 MHz, acetone-d_6) δ (ppm) 8.16 (1H, d, J = 4.9 Hz), 7.69 (1H, br s), 7.67 (1H, br s), 7.66 (1H, d, J = 4.9 Hz), 4.33-4.28 (2H, m), 3.85-3.80 (2H, m), 3.35 (3H, s), 3.00 (3H, s), 2.88-2.80 (2H, m),

2.76 (3H, s), 2.56-2.48 (2H, m); ^{13}C NMR (126 MHz, acetone-d_6) δ (ppm) 152.5, 151.5, 149.1, 141.9, 138.2, 135.7, 134.8, 133.6, 119.8, 105.3, 52.7, 30.3, 29.8; IR (neat) 2987, 1586, 1416, 1325, 1257, 1220, 1173, 1084, 822, 776, 713 cm^{-1}; HRMS (ESI+) *m/z* calcd. for ($C_{17}H_{21}S$) $[M]^+$ 257.1358, found 257.1378.

1-(3-*tert*-Butylazulen-1-yl) tetrahydrothiophenium hexafluorophosphate (11c)

To 1-*tert*-butylazulene (437 mg, 2.38 mmol, 1.0 equiv), in CH_2Cl_2 (16 mL) was added tetrahydrothiophene-1-oxide (1.65 mL, 17.8 mmol, 5.0 equiv). A solution of TFAA (1.05 g, 0.70 mL, 5.0 mmol) in CH_2Cl_2 (9.3 mL) was prepared (i.e. a 0.5 M solution), and added dropwise to the reaction mixture at rt until TLC indicated consumption of starting material (≈ 8.0 mL of TFAA solution required). The reaction mixture was diluted with Et_2O and washed with water (×3). To the aqueous layer was added potassium hexafluorophosphate (excess). The aqueous layer was washed with CH_2Cl_2 (×3). The organic layer was dried over $MgSO_4$ and filtered, then the filtrate was concentrated *in vacuo*. Recrystallisation from DCM/petroleum ether gave **11c** as a purple crystalline solid (939 mg, 2.26 mmol, 95%); m.pt. 239-240 °C (dec); ^{1}H NMR (300 MHz, acetone-d_6) δ (ppm) 9.22 (1H, d, J = 10.0 Hz), 8.93 (1H, d, J = 9.8 Hz), 8.32 (1H, s), 8.16 (1H, t, J = 9.8 Hz), 7.81 (1H, t, J = 9.8 Hz), 7.79 (1H, t, J = 9.8 Hz), 4.29-4.20 (2H, m), 3.91-3.83 (2H, m), 2.94-2.81 (2H, m), 2.61-2.50 (2H, m), 1.62 (9H, s); ^{13}C NMR (75 MHz, acetone-d_6) δ (ppm) 144.6, 143.3, 142.3, 140.6, 140.5, 136.0, 134.4, 129.0, 128.2, 103.4, 51.0, 34.4, 32.0, 30.0; IR (neat) 2956, 2873, 1578, 1381, 1218, 1205, 1133, 879, 821, 752 cm^{-1}; HRMS (ESI+) *m/z* calcd. for ($C_{18}H_{23}S$) $[M]^+$ 271.1515, found 271.1519.

1-(3,8-Dimethyl-5-*iso*-propylazulen-1-yl) tetrahydrothiophenium hexafluorophosphate (11d)

To guaiazulene (1.025 g, 5.18 mmol, 1.0 equiv), in CH_2Cl_2 (50 mL) was added dimethylsulfoxide (0.74 mL, 10.4 mmol, 2.0 equiv). A solution of TFAA (1.43 mL, 10.4 mmol, 2.0 equiv) in CH_2Cl_2 (25 mL) was prepared and added dropwise to the reaction mixture at rt, then stirred for 1 h, until TLC indicated consumption of starting material (≈ 8.0 mL of TFAA solution required). The reaction mixture was diluted with pentane and washed with water (×3). To the aqueous layer was added potassium hexafluorophosphate (excess). The aqueous layer was washed with CH_2Cl_2 (×3). The organic layer was dried over $MgSO_4$ and filtered, then the filtrate was concentrated *in*

vacuo. Trituration with Et_2O gave **11d** as a purple crystalline solid (1.06 g, 68%); m.pt. 158 °C; ^{1}H NMR (500 MHz, acetone-d_6) δ (ppm) 8.64 (1H, d, *J* = 2.2 Hz), 8.43 (1H, s), 7.98 (1H, dd, *J* = 10.8, 2.2 Hz), 7.68 (1H, d, *J* = 10.8 Hz), 3.53 (6H, s), 3.33 (1H, septet, *J* = 6.9 Hz), 3.28 (3H, s), 2.71 (3H, s), 1.42 (6H, d, *J* = 6.9 Hz); ^{13}C NMR (126 MHz, acetone-d_6) δ (ppm) 148.7, 147.8, 142.8, 139.7, 139.4, 138.4, 135.7, 134.7, 129.8, 100.8, 38.6, 33.3, 28.9, 24.6, 13.1; IR (neat) 2966, 1698, 1525, 1429, 1392, 1375, 1143, 833 cm^{-1}; HRMS (ESI+) *m/z* calcd. for ($C_{17}H_{23}S$) $[M]^+$ 259.1515, found 259.1556.

1-(4,8-Dimethyl-6-methoxyazulen-1-yl) tetrahydrothiophenium hexafluorophosphate (11e)

To 4,8-dimethy-6-methoxyazulene (40 mg, 0.215 mmol, 1.0 equiv) in CH_2Cl_2 (5 mL) at room temperature was added tetrahydrothiophene-1-oxide (44 mg, 0.430 mmol, 2 equiv). The reaction mixture was stirred for 10 min, then a solution of TFAA (90 mg, 0.430 mmol, 2 equiv) in CH_2Cl_2 (5 mL) was added dropwise, until TLC indicated consumption of starting material (approx. 4 mL of this solution was needed). The reaction mixture was diluted with water in a separating funnel. The phases were separated, and KPF_6 (158 mg, 0.866 mmol, 4.0 equiv) was added to the aqueous phase. The product was then back-extracted into CH_2Cl_2. This organic layer was then dried over $MgSO_4$ and filtered. The filtrate was concentrated *in vacuo* and the resultant solid was triturated with several portions of Et_2O to give **11e** as a bright red/orange crystalline solid (82 mg, 91%); m.p. 197 °C; ^{1}H NMR (500 MHz, acetone-d_6) δ (ppm) 7.94 (1H, d, *J* = 4.9 Hz), 7.58 (1H, d, *J* = 4.9 Hz), 7.38 (1H, d, *J* = 2.8 Hz), 7.33 (1H, d, *J* = 2.8 Hz), 4.31-4.26 (2H, m), 4.12 (3H, s), 3.82-3.77 (2H, m), 3.32 (3H, s), 2.99 (3H, s), 2.86-2.78 (2H, m), 2.55-2.47 (2H, m); ^{13}C NMR (126 MHz, acetone-d_6) δ (ppm) 167.3, 151.5, 148.6, 137.8, 134.5, 130.5, 121.0, 119.2, 117.3, 104.7, 56.3, 51.7, 29.6, 28.9, 25.5; IR (neat) 2980, 1592, 1555, 1332, 1227, 1106, 1064, 824, 776, 715, 621 cm^{-1}; HRMS (ESI+) *m/z* calcd. for ($C_{17}H_{21}OS$) $[M]^+$ 273.1308, found 273.1378.

1-(3-(3,5-bis(Trifluoromethyl)phenyl)azulen-1-yl) tetrahydrothiophenium hexafluorophosphate (11f)

This was synthesized analogously with **11a**, using 3-(3,5-bis(trifluoromethyl)phenyl)azulene **13g** (84.5 mg, 0.248 mmol, 1.0 equiv), tetrahydrothiophene-1-oxide (26 mg, 0.248 mmol, 1.0 equiv), trifluoromethanesulfonic

anhydride (140 mg, 0.496 mmol, 2.0 equiv.) and KPF_6 (183 mg, 0.992 mmol, 4 equiv). Purification as per 1**1a** gave the desired **11f** as a purple crystalline solid (104 mg, 73%); m.p. 167 °C; ^{1}H NMR (500 MHz, acetone-d_6) δ (ppm) 9.13 (1H, d, J = 9.9 Hz), 8.99 (1H, d, J = 9.9 Hz), 8.86 (1H, s), 8.33 (2H, s), 8.31 (1H, t, J = 9.9 Hz), 8.13 (1H, s), 7.99 (1H, t, J = 9.9 Hz), 7.95 (1H, t, J = 9.9 Hz), 4.37-4.31 (2H, m), 4.02-3.97 (2H, m), 2.94-2.88 (2H, m), 2.63-2.56 (2H, m); ^{13}C NMR (75 MHz, acetone-d_6) δ (ppm) 143.9, 143.8, 140.6, 140.1, 138.9, 137.7, 137.4, 132.7 (q, $^2J_{CF}$ = 33.4 Hz), 131.1, 131.0 (q, $^3J_{CF}$ = 3.1 Hz), 130.7, 130.5, 124.5 (q, $^1J_{CF}$ = 272.3 Hz), 121.8 (septet, $^3J_{CF}$ = 3.7 Hz), 106.5, 51.4, 30.0; ^{19}F NMR (470.5 MHz, acetone-d_6) δ (ppm) –63.2 (s), –72.7 (d, $^1J_{PF}$ = 707.0 Hz); IR (neat) 3020, 2964, 1584, 1428, 1276, 1161, 1130, 818, 754, 710, 701, 682 cm$^{-1;}$ HRMS (ESI+) *m/z* calcd. for ($C_{22}H_{17}F_6S$) $[M]^+$ 427.0950, found 427.1074.

*Representative procedure for formation of Suzuki–Miyaura azulene products **13**.*

1-(*para*-Tolyl)azulene (13a)

Azulenesulfonium salt **11a** (100 mg, 0.278 mmol, 1.0 equiv.), 4,4,5,5-tetramethyl-2-(*p*-tolyl)-1,3,2-dioxaborolane (72 mg, 0.330 mmol, 1.2 equiv.), X-Phos (13 mg, 0.027 mmol, 10 mol%), palladium acetate (2.6 mg, 0.012 mmol, 4 mol%) and potassium phosphate (118 mg, 0.556 mmol, 2.0 equiv.) were added to a 5 mL vial. The vial was sealed, evacuated, filled with an atmosphere of argon and degassed DMF (2.00 mL) was added. The reaction was heated to 70 °C for 4 hours. The reaction mixture was cooled to room temperature, diluted with diethyl ether and washed with water (×3). The organic layer was further washed with 5% $LiCl_{(aq)}$ (×1) and saturated aqueous brine solution (×1). The organic layer was dried over $MgSO_4$, then filtered. The filtrate was concentrated *in vacuo*. The crude product was purified by flash column chromatography (silica gel, 1% EtOAc/petrol) to afford to the title compound **13a** as a dark blue gum (36.5 mg, 60%). R_f 0.42 (9:1 petrol:Et_2O) ^{1}H NMR (500 MHz, $CDCl_3$) δ (ppm) 8.54 (1H, d, *J* = 9.8 Hz) 8.34 (1H, d, *J* = 9.8 Hz) 8.01 (1H, d, *J* = 3.9 Hz) 7.58 (1H, t, *J* = 9.8 Hz) 7.52 (2H, d, *J* = 7.8 Hz), 7.44 (1H, d, *J* = 3.9 Hz) 7.31 (2H, d, *J* = 7.8 Hz), 7.14 (1H, t, *J* = 9.8 Hz), 7.13 (1H, t, *J* = 9.8 Hz), 2.44 (3H, s); ^{13}C NMR (126 MHz, $CDCl_3$) δ (ppm) 141.6, 138.1, 137.1, 137.0, 135.9, 135.6, 135.1, 134.5, 131.3, 129.6, 129.3, 123.1, 122.8, 117.3, 21.2; IR (neat) 3020, 2917, 2858, 1568, 1523, 1492, 1454, 1420, 1390, 782 cm^{-1}; HRMS (ESI+) *m/z* calcd. for $C_{17}H_{14}$ $[M+H]^+$ 219.1168, found 219.1097. Data agree with previous reports.[2]

***tert*-Butyl (4-(azulen-1-yl)phenyl)carbamate (13b)**

The title compound was synthesized from *tert*-butyl(4-(4,4,5,5-tetramethyl-1,3,2-dioxaborolan-2-yl)phenyl)carbamate **12b** (128 mg, 0.417 mmol, 1.5 equiv.), by analogy with **13a** (reaction time 4 h). Flash column chromatography (silica gel, 6% EtOAc/petrol) gave desired product **13b** as a dark blue gum (56 mg, 63%). R_f 0.51 (9:1 petrol:Et_2O) ^{1}H NMR (500 MHz, $CDCl_3$) δ (ppm) 8.52 (1H, d, *J* = 9.8 Hz), 8.33 (1H, d, *J* = 9.8 Hz), 7.99 (1H, d, *J* = 3.9 Hz), 7.60-7.55 (3H, m), 7.50 (2H, d, *J* = 8.2 Hz), 7.13 (1H, t, *J* = 9.8 Hz), 7.12 (1H, 7, *J* = 9.8 Hz), 6.60 (1H, br s), 1.57 (9H, s); ^{13}C NMR (126 MHz, $CDCl_3$) δ (ppm) 152.9, 141.6, 138.1, 137.2, 136.9, 136.7, 135.6, 135.1, 132.4, 130.9, 130.2, 123.1, 122.9, 119.0, 117.4, 80.6, 28.4; IR (neat) 3326, 2977, 2928, 1698, 1588, 1528, 1507, 1394, 1367, 1314, 1231, 1159, 1052, 840, 775, 742 cm^{-1}. HRMS (ESI+) *m/z* calcd. for ($C_{21}H_{21}NO_2$) $[M+H]^+$ 320.1645, found 320.1636; HRMS (ESI+) *m/z* calcd. for ($C_{21}H_{21}NO_2$) $[M+Na]^+$ 342.1459, found 342.1481.

(4-(Azulen-1-yl)phenyl)(methyl)sulfane (13c)

The title compound was synthesized from 4-(methylthio)phenylboronic acid **12c** (76 mg, 0.417 mmol, 1.5 equiv.) by analogy with **13a** (reaction time 4 h). Flash column chromatography (silica gel, 1% EtOAc/petrol) gave desired product **13c** as a dark blue gum (37 mg, 53%). R_f 0.51 (9:1 petrol:Et_2O); ^1H NMR (500 MHz, $CDCl_3$) δ (ppm) 8.54 (1H, d, J = 9.8 Hz), 8.35 (1H, d, J = 9.8 Hz), 8.01 (1H, d, J = 3.8 Hz), 7.60 (1H, t, J = 9.8 Hz), 7.56 (2H, d, J = 8.2 Hz), 7.45 (1H, d, J = 3.8 Hz), 7.41 (2H, d, J = 8.2 Hz), 7.16 (1H, t, J = 9.8 Hz), 7.15 (1H, 7, J = 9.8 Hz), 2.57 (3H, s); ^{13}C NMR (126 MHz, $CDCl_3$) δ (ppm) 141.8, 138.3, 137.3, 137.0, 136.2, 135.5, 135.2, 134.5, 130.7, 130.1, 127.2, 123.3, 123.1, 117.5, 117.5, 16.2; IR (neat) 3024, 2983, 2918, 1590, 1571, 1511, 1483, 1395, 823, 777, 741 cm^{-1}; HRMS (ESI+) *m/z* calcd. for ($C_{17}H_{14}S$) $[M+H]^+$ 251.0889, found 251.0878.

1-(4-(Trifluoromethyl)phenyl)azulene (13d)

The title compound was synthesized from 4-(trifluoromethyl)phenylboronic acid **13d** (63 mg, 0.33 mmol, 1.2 equiv) by analogy with **13a** (reaction time 1.5 h). Flash column chromatography on alumina (2.5% EtOAc/petrol) gave desired product **13d** as a blue oil (36 mg, 47%). R_f 0.48 (1:9 EtOAc:petrol, silica plate); ^1H NMR (500 MHz, $CDCl_3$) δ (ppm) 8.56 (1H, d, J = 9.8 Hz) 8.41 (1H, d, J = 9.8 Hz) 8.05 (1H, d, J = 3.9 Hz) 7.77 (2H, d, J = 8.5 Hz) 7.74 (2H, d, J = 8.5 Hz) 7.66 (1H, t, J = 9.8 Hz) 7.48 (1H, d, J = 3.9 Hz) 7.24 (1H, t, J = 9.8 Hz) 7.23 (1H t, J = 9.8 Hz); ^{13}C NMR (126 MHz, $CDCl_3$) δ (ppm) 142.1, 141.1, 138.5, 137.6, 137.1, 135.5, 135.3, 129.7, 129.5, 128.0 (q, $^2J_{CF}$ = 32.5 Hz), 125.5, (q, $^3J_{CF}$ = 3.8 Hz), 124.5 (q, $^1J_{CF}$ = 271.7 Hz), 124.0, 123.4, 117.8; ^{19}F NMR (470.5 MHz, $CDCl_3$) δ (ppm) –62.2; IR (neat) 3029, 1613, 1572, 1530, 1496, 1454, 1399, 1320, 1161, 1096, 841, 779 cm^{-1}; HRMS (APCI+) *m/z* calcd. for ($C_{17}H_{11}F_3$) $[M]^+$ 272.0807, found 272.0810. This compound has been reported previously, but complete spectroscopic data were not given.[3]

1-(*ortho*-Tolyl)azulene 13e

The title compound was synthesized from *o*-tolylboronic acid **12e** (45 mg, 0.33 mmol, 1.2 equiv.) by analogy with **13a** (reaction time 5 h). Flash column chromatography (silica gel, 1% EtOAc/petrol) gave desired product **13e** as a dark blue gum (23 mg, 38%). R_f 0.62 (9:1 petrol:Et_2O); ^1H NMR (500 MHz, $CDCl_3$) δ (ppm) 8.37 (1H, d, J = 9.3 Hz) 8.07 (1H, d, J = 9.3 Hz) 7.91 (1H, d, J = 3.9 Hz) 7.59 (1H, t, J = 9.8 Hz) 7.46 (1H, d, J = 3.9

Hz) 7.38-7.29 (4H, m) 7.17 (1H, t, J = 9.5 Hz) 7.09 (1H, t, J = 9.8 Hz) 2.19 (3H, s); ^{13}C NMR (126 MHz, $CDCl_3$) δ (ppm) 140.7, 138.0, 137.7, 137.4, 137.0, 136.9, 136.3, 135.7, 131.5, 131.0, 130.1, 127.0, 125.4, 122.8, 122.7, 116.8, 20.6; IR (neat) 3022, 2922, 1572, 1480, 1456, 1393, 765, 749 cm^{-1}; HRMS (ESI+) *m/z* calcd. for ($C_{17}H_{14}$) $[M+H]^+$ 219.1168, found 219.1193.

1-(4-(Hydroxymethyl)phenyl)azulene 13f: The title compound was synthesized from 4-(hydroxymethyl)phenylboronic acid **12f** (50 mg, 0.33 mmol, 1.2 equiv.) by analogy with **13a** (reaction time 4 h). Flash column chromatography on alumina (8% ^{i}PrOH/petrol) gave desired product **13f** as a blue oil (32 mg, 47%). R_f 0.44 (1:1 petrol:EtOAc, silica plate); ^{1}H NMR (500 MHz, $CDCl_3$) δ (ppm) 8.56 (1H, d, J = 9.8 Hz), 8.37 (1H, d, J = 9.3 Hz), 8.03 (1H, d, J = 3.9 Hz), 7.64 (2H, d, J = 8.3 Hz), 7.60 (1H, t, J = 9.7 Hz), 7.51 (2H, d, J = 8.3 Hz), 7.45 (1H, d, J = 3.9 Hz) 7.17 (1H, t, J = 9.8 Hz), 7.16 (1H, t, J = 9.8 Hz) 4.79 (s, 2 H) 1.78 (1H, br s); ^{13}C NMR (126 MHz, $CDCl_3$) δ (ppm) 141.7, 138.8, 138.2, 137.3, 137.1, 137.0, 135.5, 135.2, 130.8, 129.9, 127.4, 123.3, 123.1, 117.5, 65.3; IR (neat) 3033, 1613, 1568, 1523, 1496, 1454, 1396, 1323, 1161, 1099 cm^{-1}; HRMS (ESI+) *m/z* calcd. for ($C_{17}H_{14}O$) $[M+H]^+$ 235.1117, found 235.1113.

1-(3,5-bis(Trifluoromethyl)phenyl)azulene 13g

The title compound was synthesized from (3,5-bis(trifluoromethyl)phenyl)boronic acid **12g** (108 mg, 0.417 mmol, 1.5 equiv.) by analogy with **13a** (reaction time 4 h). Flash column chromatography (silica gel, 1% EtOAc/petrol) gave desired product **13g** as a dark blue gum (60 mg, 63%). R_f 0.60 (9:1 petrol:Et_2O); ^{1}H NMR (500 MHz, $CDCl_3$) δ (ppm) 8.47 (1H, d, J = 9.8 Hz) 8.43 (1H, d, J = 9.3 Hz), 8.05 (2H, s), 8.04 (1H, d, J = 3.9 Hz), 7.85 (1H, s) 7.70 (1H, t, J = 9.8 Hz), 7.48 (1H, d, J = 3.9 Hz), 7.29 (1H, t, J = 9.8 Hz), 7.28 (1H, t, J = 9.8 Hz); ^{13}C NMR (126 MHz, $CDCl_3$) δ (ppm) 142.3, 139.6, 138.8, 138.0, 137.1, 135.7, 134.8, 131.9 (q, $^2J_{CF}$ = 33.1 Hz), 129.3 (q, $^3J_{CF}$ = 2.5 Hz), 127.6, 124.6, 124.3, 123.6 (q, $^1J_{CF}$ = 272.6 Hz), 119.6 (septet, $^3J_{CF}$ = 3.8 Hz), 118.0; IR (neat) 3031, 1617, 1575, 1508, 1470, 1411, 1397, 1362, 1313, 1273, 1168, 1116, 1105, 989, 958, 891, 845, 777, 740, 682 cm^{-1}; HRMS (ESI+) *m/z* calcd. for ($C_{18}H_{10}F_6$) $[M+H]^+$ 341.0759; found 341.0753. Data partially agree with those previously reported.[4]

3-(Azulen-1-yl)-2-fluoropyridine 13h

The title compound was synthesized from 2-fluoropyridine-3-boronic acid **12h** (59 mg, 0.417 mmol, 1.5 equiv.) by analogy with **13a** (reaction time 4 h). Flash column

chromatography (silica gel, 1.5% EtOAc/petrol) gave desired product **13h** as a dark blue gum (36 mg, 57%). R_f 0.58 (9:1 petrol:Et_2O); ^1H NMR (500 MHz, $CDCl_3$) δ (ppm) 8.42 (1H, d, J = 9.5 Hz, H^E) 8.33 (1H, d, J = 9.8 Hz, H^I), 8.23 (1H, dt, J = 4.9, 1.5 Hz, H^M) 8.06 (1H, dd, J = 3.9, 2.0 Hz, H^B) 7.96 (1H, ddd, J = 9.6, 7.4, 2.0 Hz, H^L) 7.68 (1H, t, J = 9.9 Hz, H^G), 7.49 (1H, d, J = 3.9 Hz, H^C) 7.33 (1H, ddd, J = 7.3, 4.9, 1.9 Hz, H^N) 7.27 (1H, t, J = 9.9 Hz, H^F), 7.25 (1H, t, J = 9.9 Hz, H^H); ^{13}C NMR (126 MHz, $CDCl_3$) δ (ppm) 160.9 (d, $^1J_{CF}$ = 239.6 Hz, C^O), 145.3 (d, $^4J_{CF}$ = 14.4 Hz, C^M), 142.1 (d, $^3J_{CF}$ = 4.8 Hz, C^L), 142.0 (C^D or C^J – prob. J), 138.4 (C^G), 138.1 (d, $^4J_{CF}$ = 2.4 Hz, C^B), 137.5 (C^E), 136.2 (C^D or C^J), 135.1 ($^5J_{CF}$ = 2.3 Hz, C^I), 124.1 (C^F), 124.0 (C^H), 121.9 (d, $^3J_{CF}$ = 4.2 Hz, C^A), 121.4 (d, $^3J_{CF}$ = 4.3 Hz, C^N), 120.1 (d, $^2J_{CF}$ = 30.3 Hz, C^K), 117.8 (C^C); IR (neat) 3054, 3025, 2926, 2854, 1600, 1508, 1464, 1567, 1439, 1395, 1295, 1243, 1203, 1081, 875, 825, 777, 740 cm^{-1}; ^{19}F NMR (470.5 MHz, $CDCl_3$) δ (ppm) –71.6; HRMS (ESI+) *m/z* calcd. for ($C_{15}H_{10}FN$) $[M+H]^+$ 224.0870, found 224.0876; calcd. for ($C_{15}H_{10}FN$) $[M+Na]^+$ 246.0689, found 246.0683.

5-(Azulen-1-yl)furan-2-carbaldehyde 13i

The title compound was synthesized from 2-formylfuran-5-boronic acid **12i** (59 mg, 0.417 mmol, 1.5 equiv.) by analogy with **13a** (reaction time 4 h). Flash column chromatography (silica gel, 10% EtOAc/petrol) gave desired product **13h** as a dark green gum (39 mg, 63%). R_f 0.31 (7:3 petrol:Et_2O); ^1H NMR (500 MHz, $CDCl_3$) δ (ppm) 9.62 (1H, s), 9.16 (1H, d, J = 9.9 Hz), 8.34 (1H, d, J = 9.4 Hz), 8.20 (1H, d, J = 4.2 Hz), 7.69 (1H, t, J = 9.9 Hz), 7.41-7.37 (3H, m), 7.28 (1H, t, J = 9.6 Hz), 6.84 (1H, d, J = 3.8 Hz); ^{13}C NMR (126 MHz, $CDCl_3$) δ (ppm) 176.1, 159.1, 151.3, 143.9, 139.3, 138.0, 137.1, 136.2, 135.5, 125.9, 125.5, 124.8 (br), 119.1, 117.3, 108.0; IR (neat) 3116, 2806, 1654, 1593, 1566, 1513, 1393, 1354, 1328, 1282, 1027, 961, 849, 768, 741 cm^{-1}; HRMS (ESI+) *m/z* calcd. for ($C_{15}H_{10}O_2$) $[M+H]^+$ 223.0754, found 223.0757; calcd. for ($C_{15}H_{10}O_2$) $[M+Na]^+$ 245.0573, found 245.0575.

5-(Azulen-1-yl)-1-(tetrahydro-2*H*-pyran-2-yl)-1*H*-pyrazole 13j

The title compound was synthesized from 1-(tetrahydro-2H-pyran-2-yl)-5-(4,4,5,5-tetramethyl-1,3,2-dioxaborolan-2-yl)-1H-pyrazole **12j** (117 mg, 0.417 mmol, 1.5 equiv.) by analogy with **13a** (reaction time 5 h). Flash column chromatography (silica gel, 10% EtOAc/petrol) gave desired product **13j** as dark blue gum (45 mg, 58%). R_f 0.30 (7:3

petrol:Et_2O) ^1H NMR (500 MHz, $CDCl_3$) δ (ppm) 8.49 (1H d, *J* = 9.8 Hz) 8.42 (1H d, *J* = 9.3 Hz), 8.05 (1H, d, *J* = 3.9 Hz), 7.73 (1H, d, *J* = 1.5 Hz), 7.68 (1H, t, *J* = 9.9 Hz), 7.47 (1H, d, *J* = 3.9 Hz), 7.27 (1H, t, *J* = 9.9 Hz), 7.24 (1H, t, *J* = 9.9 Hz), 6.44 (1H d, *J* =1.5 Hz), 5.18 (1H, dd, *J* = 10.3, 2.4 Hz), 4.15-4.12 (1H, m), 3.51 (1H, td, *J* = 11.7, 2.4 Hz), 2.59 (1H, dtd, *J* = 13.0, 10.6, 4.5 Hz), 2.06-1.99 (1H, m), 1.86-1.81 (1H, m), 1.78-1.71 (1H, m), 1.54-1.44 (2H, m); ^{13}C NMR (126 MHz, $CDCl_3$) δ (ppm) 141.6, 139.7, 139.3, 138.6, 137.9, 137.7, 137.5, 135.9, 124.0, 124.0, 118.1, 117.5, 107.6, 84.2, 67.9, 29.9, 24.9, 23.0; IR (neat) 2930, 2852, 1563, 1463, 1340, 1315, 1247, 1205, 1082, 1042, 1004, 916, 859, 779, 744 cm^{-1}; HRMS (ESI+) *m/z* calcd. for ($C_{18}H_{18}N_2O$) $[M+H]^+$ 279.1492, found 279.1488; ($C_{18}H_{18}N_2O$) $[M+Na]^+$ 301.1311, found 301.1304.

5-(azulen-1-yl)thiophene-2-carbaldehyde 13k

Azulenesulfonium salt **11a** (720 mg, 2.00 mmol, 1.0 equiv.), 2-(5-(1,3-dioxolan-2-yl)thiophen-2-yl)-4,4,5,5-tetramethyl-1,3,2-dioxaborolane **12k** (846 mg, 3.00 mmol, 1.5 equiv.), X-Phos (95 mg, 0.200 mmol, 10 mol%), palladium acetate (18 mg, 0.080 mmol, 4 mol%) and potassium phosphate (848 mg, 4.00 mmol, 2.0 equiv.) were added to a 50 mL round bottom flask. The flask was sealed, evacuated, filled with an atmosphere of argon and degassed ^{i}PrOH (12.0 mL) was added. The reaction was heated to 70 °C for 2.5 hours, after which time TLC indicated completion. The reaction mixture was concentrated *in vacuo*, then the residue was taken up in Et_2O and washed with water (×3). The organic layer was further washed with saturated aqueous brine solution (×1), then dried over $MgSO_4$, then filtered. The filtrate was concentrated *in vacuo*.

The crude blue coupling product was dissolved in acetone:water 9:1 (20 mL), to which a crystal of *p*TSA was added. This was stirred at rt for 20 min, after which time TLC indicated complete removal of the acetal protecting group. The reaction mixture was extracted with Et_2O, then the organic layer was washed with saturated aqueous brine solution (×1), dried over $MgSO_4$, and then filtered. The filtrate was concentrated *in vacuo*, then the crude product was purified by flash column chromatography (silica gel, 10% EtOAc/petrol) to afford to the desired product **13k** as a green gum (286 mg, 60%). R_f 0.38 (3:7 EtOAc:petrol); ^1H NMR (300 MHz, $CDCl_3$) δ (ppm) 9.91 (1H, s), 8.86 (1H, d, *J* = 9.6 Hz), 8.37 (1H, d, *J* = 9.6 Hz), 8.13 (1H, d, *J* = 4.2 Hz), 7.81 (1H, d, *J* = 3.9 Hz), 7.70 (1H, t, *J* = 9.8 Hz), 7.42 (2H, app d, *J* = 4.2 Hz), 7.33 (1H, t, *J* = 9.8 Hz), 7.28 (1H, t, *J* = 9.8 Hz); ^{13}C NMR (75 MHz, $CDCl_3$) δ (ppm) 182.5, 150.3, 143.6, 141.2, 139.2, 138.0, 137.6, 137.3, 135.6, 135.5, 125.2, 125.1, 125.0, 122.1, 118.7; IR (neat) 1643, 1460, 1391, 1228 cm^{-1}; HRMS (ESI+) *m/z* calcd. for ($C_{15}H_{10}OS$) $[M+Na]^+$

261.0345, found 261.0359; *m/z* calcd. for ($C_{15}H_{10}OS$) $[2M+Na]^+$ 499.0797, found 499.0835.

3-(Azulen-1-yl)quinoline 13l

The title compound was synthesized from 3-(4,4,5,5-tetramethyl-1,3,2-dioxaborolan-2-yl)quinoline **12l** (107 mg, 0.417 mmol, 1.5 equiv.) by analogy with **13a** (reaction time 4 h). Flash column chromatography (silica gel, 10% EtOAc/petrol) gave desired product **13l** as a dark blue gum (39 mg, 55%). R_f 0.60 (7:3 petrol:Et_2O); ^{1}H NMR (500 MHz, $CDCl_3$) δ (ppm) 9.23 (1H d, *J* = 2.1 Hz), 8.59 (1H, d, *J* = 9.8 Hz), 8.43 (1H, d, *J* = 9.8 Hz), 8.32 (1H, d, *J* = 2.1 Hz), 8.17 (1H, d, *J* = 8.6 Hz), 8.14 (1H, d, *J* = 3.9 Hz), 7.90 (1H, d, *J* = 8.1 Hz), 7.73 (1H, ddd, *J* = 8.5, 6.9, 1.4 Hz), 7.67 (1H, t, *J* = 9.8 Hz), 7.60 (1H, ddd, *J* = 8.2, 6.9, 1.0 Hz), 7.52 (1H, d, *J* = 3.9 Hz), 7.25 (1H, t, *J* = 9.8 Hz), 7.24 (1H, t, *J* = 9.8 Hz); ^{13}C NMR (126 MHz, $CDCl_3$) δ (ppm) 152.3, 146.7, 142.1, 138.7, 137.7, 137.3, 136.1, 135.3, 134.9, 130.6, 129.3, 129.0, 128.3, 127.8, 127.2, 126.9, 124.1, 123.8, 118.0 ; IR (neat) 2927, 1572, 1395, 784, 740 cm^{-1}; HRMS (ESI+) *m/z* calcd. for ($C_{19}H_{13}N$) $[M+H]^+$ 256.1126, found 256.1131; *m/z* calcd. for ($C_{19}H_{13}N$) $[M+Na]^+$ 278.0946, found 278.0933.

3-(Azulen-1-yl)-9*H*-carbazole 13m

The title compound was synthesized from 3-(4,4,5,5-tetramethyl-1,3,2-dioxaborolan-2-yl)-9H-carbazole **12m** (123 mg, 0.417 mmol, 1.5 equiv.) by analogy with **13a** (reaction time 4 h). Flash column chromatography (silica gel, 7% EtOAc/petrol) gave desired product **13m** as a dark blue gum (46 mg, 56%). R_f 0.54 (7:3 petrol:Et_2O); ^{1}H NMR (500 MHz, $CDCl_3$) δ (ppm) 8.63 (1H, d, *J* = 9.8 Hz), 8.37 (1H, d, *J* = 9.5 Hz), 8.30 (br s), 8.14-8.09 (3H, m), 7.69 (1H, dd, *J* = 8.3, 1.6 Hz), 7.59 (1H, t, *J* = 9.8 Hz), 7.54 (1H, d, 8.3 Hz), 7.50 (1H, d, *J* = 3.8 Hz), 7.47-7.43 (2H, m), 7.27 (1H, ddd, *J* = 7.8, 6.0, 2.2 Hz), 7.15 (1H, t, *J* = 9.7 Hz), 7.14 (1H, t, *J* = 9.8 Hz); ^{13}C NMR (126 MHz, $CDCl_3$) δ (ppm) 141.4, 139.9, 138.4, 138.1, 137.4, 137.1, 135.8, 135.3, 132.3, 129.0, 128.1, 126.0, 123.8, 123.5, 123.0, 122.7, 121.3, 120.4, 119.5, 117.3, 110.7, 110.7; IR (neat) 3411, 3056, 1606, 1574, 1476, 1393, 1383, 1239, 1134, 907, 815, 772, 740 cm^{-1}; HRMS (ESI+) *m/z* calcd. for ($C_{22}H_{15}N$) $[M+Na]^+$ 316.1097, found 316.1094.

5'-(azulen-1-yl)-[2,2'-bithiophene]-5-carbaldehyde 13n

The title compound was synthesized from azulenesulfonium salt **11a** (540 mg, 1.50 mmol, 1.0 equiv.), 2-(5'-(5,5-dimethyl-1,3-dioxan-2-yl)-[2,2'-bithiophen]-5-yl)-4,4,5,5-

tetramethyl-1,3,2-dioxaborolane **12n** (756 mg, 2.00 mmol, 1.33 equiv.), X-Phos (71 mg, 0.150 mmol, 10 mol%), palladium acetate (14 mg, 0.060 mmol, 4 mol%) and potassium phosphate (636 mg, 3.00 mmol, 2.0 equiv.) by analogy with **13k**, except that the cross-coupling solvent was degassed DMF (9.0 mL). Flash column chromatography (silica gel, 10% to 20% EtOAc/petrol) gave desired product **13n** as a dark green gum (230 mg, 48%). R_f 0.33 (7:3 petrol:Et_2O); ^1H NMR (500 MHz, $CDCl_3$) δ (ppm) 9.87 (1H, s), 8.77 (1H, d, J = 9.8 Hz), 8.33 (1H, d, J = 9.2 Hz), 8.07 (1H, d, J = 4.1 Hz), 7.69 (1H, d, J = 4.0 Hz), 7.65 (1H, t, J = 9.9 Hz), 7.44 (1H, d, J = 3.8 Hz), 7.41 (1H, d, J = 4.1 Hz), 7.29 (1H, d, J = 4.0 Hz), 7.27 (1H, d, J = 3.9 Hz), 7.26 (1H, t, J = 9.9 Hz), 7.21 (1H, t, J = 9.7 Hz); ^{13}C NMR (126 MHz, $CDCl_3$) δ (ppm) 182.3, 147.7, 142.9, 142.0, 138.9, 137.7, 137.5, 137.0, 135.7, 135.1, 134.3, 127.0, 125.4, 124.3, 124.3, 123.6, 122.5, 118.3, 105.3; IR (neat) 2785, 1647, 1570, 1472, 1393, 1223, 1045, 794, 785, 735 cm^{-1}; HRMS (ESI+) *m/z* calcd. for ($C_{19}H_{12}OS_2$) $[M+H]^+$ 321.0402, found 321.0387.

5-(4,6,8-Trimethylazulen-1-yl)thiophene-2-carbaldehyde 13o

The title compound was synthesized from azulenesulfonium salt **11b** (1.209 g, 3.00 mmol, 1.0 equiv.), 2-(5-(1,3-dioxolan-2-yl)thiophen-2-yl)-4,4,5,5-tetramethyl-1,3,2-dioxaborolane **12k** (1.269 g, 4.50 mmol, 1.50 equiv.), X-Phos (143 mg, 0.300 mmol, 10 mol%), palladium acetate (27 mg, 0.120 mmol, 4 mol%) and potassium phosphate (1.272 g, 6.00 mmol, 2.0 equiv.), by analogy with **13k**, using degassed ^{i}PrOH (18 mL). Flash column chromatography (silica gel, 10% EtOAc/petrol) gave desired product **13o** as dark maroon gum (569 mg, 68%). R_f 0.53 (7:3 petrol:EtOAc); ^1H NMR (500 MHz, $CDCl_3$) δ (ppm) 9.90 (1H, s), 7.72 (1H, d, J = 3.8 Hz), 7.65 (1H, d, J = 4.2 Hz), 7.36 (1H, d, J = 4.2 Hz), 7.16 (1H, br s), 7.10 (1H, br s), 7.02 (1H, d, J = 3.8 Hz), ^{13}C NMR (126 MHz, $CDCl_3$) δ (ppm) 182.8, 154.7, 147.6, 147.2, 146.6, 142.6, 138.5, 137.2, 136.4, 132.6, 130.3, 129.2, 128.5, 121.7, 115.3, 28.4, 28.1, 25.6; IR (neat) 3068, 2922, 2803, 1655, 1577, 1462, 1412, 1227, 1042, 954, 812, 782, 724, 667 cm^{-1}; HRMS (ESI+) *m/z* calcd. for ($C_{18}H_{16}OS$) $[M+H]^+$ 281.0995, found 281.0949.

5-(4,6,8-Trimethylazulen-1-yl)furan-2-carbaldehyde 13p

The title compound was synthesized from 1-(4,6,8-trimethylazulen-1-yl)tetrahydro-1*H*-thiophen-1-ium hexafluorophosphate **11b** (113 mg, 0.28 mmol) and 2-formylfuran-5-boronic acid **12i** (59 mg, 0.417 mmol, 1.5 equiv.) by analogy with **13a** (reaction time 5 h). Flash column chromatography (silica gel, 10% EtOAc/petrol) gave desired product **13p** as a purple solid (39 mg, 50%). m.pt. 119 °C; ^1H NMR (500 MHz, $CDCl_3$) δ (ppm)

9.63 (1H, s), 7.72 (1H, d, J = 4.2 Hz), 7.37 (1H, d, J = 3.6 Hz), 7.32 (1H, d, J = 4.2 Hz), 7.17 (1H, br s), 7.15 (1H, br s), 6.61 (1H, d, J = 3.6 Hz), 2.90 (3H, s), 2.64 (6H, s); ^{13}C NMR (126 MHz, $CDCl_3$) δ (ppm) 177.1, 160.5, 151.9, 147.9, 147.5, 146.8, 139.0, 136.4, 133.5, 130.7, 129.1, 123.5 (br), 117.6, 115.4, 111.2, 28.5, 27.3, 25.7; IR (neat) 2926, 1669, 1579, 1516 cm^{-1}; HRMS (ESI+) *m/z* calcd. for ($C_{18}H_{16}O_2$) $[M+H]^+$ 265.1223, found 265.1227; calcd. for ($C_{18}H_{16}O_2$) $[M+Na]^+$ 287.1043, found 287.1052.

5'-(4,6,8-Trimethylazulen-1-yl)-[2,2'-bithiophene]-5-carbaldehyde 13q

The title compound was synthesized from azulenesulfonium salt **11b** (402 g, 1.00 mmol, 1.0 equiv.), 2-(5'-(5,5-dimethyl-1,3-dioxan-2-yl)-[2,2'-bithiophen]-5-yl)-4,4,5,5-tetramethyl-1,3,2-dioxaborolane **12n** (508 mg, 1.25 mmol, 1.25 equiv.), S-Phos (41 mg, 0.100 mmol, 10 mol%), palladium acetate (9 mg, 0.040 mmol, 4 mol%) and potassium phosphate (424 mg, 2.00 mmol, 2.0 equiv.), by analogy with **13k**, using degassed ^{i}PrOH (9 mL). The acetal removal step in acetone:water 9:1 required 2 h at 35 °C. Flash column chromatography (silica gel, 5% to 15% EtOAc/petrol) gave desired product **13q** as grey/black solid, but dark rust color in solution (215 mg, 59%). m.pt. 147-150 °C; R_f 0.44 (7:3 petrol:EtOAc); ^{1}H NMR (500 MHz, $CDCl_3$) δ (ppm) 9.86 (1H, s), 7.67 (1H, d, J = 4.1 Hz), 7.66 (1H, d, J = 4.3 Hz), 7.36 (1H, d, J = 4.1 Hz), 7.34 (1H, d, J = 3.7 Hz), 7.25 (1H, d, J = 4.0 Hz), 7.12 (1H, br s), 7.07 (1H, br s), 6.88 (1H, d, J = 3.7 Hz), 2.91 (3H, s), 2.71 (3H, s), 2.63 (3H, s); ^{13}C NMR (126 MHz, $CDCl_3$) δ (ppm) 182.4, 147.9, 147.8, 147.0, 146.4, 146.0, 141.1, 138.2, 137.5, 137.4, 135.2, 132.7, 130.0, 129.2, 128.1, 125.9, 123.6, 121.9, 115.2, 28.5, 27.9, 25.6; IR (neat) 3095, 2806, 1649, 1577, 1442, 1413, 1375, 1331, 1224, 1103, 1049, 842, 797, 662 cm^{-1}; HRMS (ESI+) *m/z* calcd. for ($C_{22}H_{18}OS_2$) $[M+H]^+$ 363.0872, found 363.0864.

5-(3-(tert-butyl)azulen-1-yl)thiophene-2-carbaldehyde 13r

The title compound was synthesized from azulenesulfonium salt **11c** (939 mg, 2.26 mmol, 1.0 equiv.), 2-(5-(1,3-dioxolan-2-yl)thiophen-2-yl)-4,4,5,5-tetramethyl-1,3,2-dioxaborolane **12k** (956 mg, 3.39 mmol, 1.50 equiv.), X-Phos (108 mg, 0.226 mmol, 10 mol%), palladium acetate (20 mg, 0.090 mmol, 4 mol%) and potassium phosphate (958 mg, 4.52 mmol, 2.0 equiv.), by analogy with **13k**, using degassed ^{i}PrOH (13.6 mL). The acetal removal step in acetone:water 9:1 required 30 min at rt. Flash column chromatography (silica gel, 10% EtOAc/petrol) gave desired product **13r** as green/grey solid (485 mg, 73%). m.pt. 150-151 °C; R_f 0.41 (7:3 petrol:EtOAc); ^{1}H NMR (500 MHz, $CDCl_3$) δ (ppm) 9.90 (1H, s), 8.77 (1H, d, J = 9.5 Hz), 8.69 (1H, d, J = 9.7 Hz), 8.02 (1H,

s), 7.80 (1H, d, J = 4.0 Hz), 7.63 (1H, t, J = 9.8 Hz), 7.39 (1H, d, J = 4.0 Hz), 7.21 (1H, t, J = 9.4 Hz), 7.2 (1H, t, J = 9.9 Hz), 1.62 (9H, s); ^{13}C NMR (126 MHz, $CDCl_3$) δ (ppm) 182.4, 150.4, 141.1, 139.9, 139.0, 138.7, 137.5, 136.8, 136.8, 136.0, 135.3, 125.0, 124.6, 123.3, 120.0, 33.2, 31.9; IR (neat) 2947, 2786, 2726, 1639, 1456, 1440, 1358, 1231, 1084, 873, 789, 746 cm^{-1}; HRMS (ESI+) *m/z* calcd. for ($C_{19}H_{18}OS$) [M+H]$^+$ 295.1151, found 295.1085; calcd. for ($C_{19}H_{18}OS$) [M+Na]$^+$ 317.0971, found 317.0907.

5'-(3-(*tert*-Butyl)azulen-1-yl)-[2,2'-bithiophene]-5-carbaldehyde 13s

The title compound was synthesized from azulenesulfonium salt **11c** (998 mg, 2.40 mmol, 1.0 equiv.), 2-(5'-(5,5-dimethyl-1,3-dioxan-2-yl)-[2,2'-bithiophen]-5-yl)-4,4,5,5-tetramethyl-1,3,2-dioxaborolane **12n** (1.218 g, 3.00 mmol, 1.25 equiv.), S-Phos (99 mg, 0.240 mmol, 10 mol%), palladium acetate (22 mg, 0.096 mmol, 4 mol%) and potassium phosphate (1.018 g, 4.80 mmol, 2.0 equiv.), by analogy with **13k**, using degassed ^{i}PrOH (22 mL). The cross-coupling required only 1 h; the acetal removal step in acetone:water 9:1 required 2.5 h at 35 °C. Flash column chromatography (silica gel, 5% to 15% EtOAc/petrol) gave desired product **13s** as red solid, but green in solution (736 mg, 82%). m.pt. 95-97 °C; R_f 0.41 (7:3 petrol:EtOAc); ^{1}H NMR (500 MHz, $CDCl_3$) δ (ppm) 9.86 (1H, s), 8.69 (1H, d, J = 9.9 Hz), 8.65 (1H, d, J = 9.9 Hz), 7.97 (1H, s), 7.69 (1H, d, J = 4.0 Hz), 7.57 (1H, t, J = 9.8 Hz), 7.43 (1H, d, J = 3.9 Hz), 7.27 (1H, d, J = 3.9 Hz), 7.23 (1H, d, J = 3.8 Hz), 7.14 (1H, t, J = 9.6 Hz), 7.12 (1H, t, J = 9.9 Hz), 1.62 (9H, s); ^{13}C NMR (126 MHz, $CDCl_3$) δ (ppm) 182.4, 147.7, 142.2, 141.0, 139.5, 138.7, 137.8, 137.5, 136.5, 136.2, 135.8, 135.3, 134.2, 127.0, 125.4, 123.6, 123.5, 122.5, 120.3, 33.3, 32.0; IR (neat) 2949, 1675, 1666, 1569, 1459, 14414, 1363, 1225, 1050, 869, 801, 789, 668 cm^{-1}; HRMS (ESI+) *m/z* calcd. for ($C_{23}H_{20}OS_2$) [M+H]$^+$ 363.1034, found 377.1015.

5-(5-*iso*-Propyl-3,8-dimethylazulen-1-yl)furan-2-carbaldehyde 13t

The title compound was synthesized from guaiazulenesulfonium salt **11d** (100 mg, 0.248 mmol, 1.0 equiv.), 2-formylfuran-5-boronic acid **12i** (52 mg, 0.371 mmol, 1.50 equiv.), X-Phos (12 mg, 0.0248 mmol, 10 mol%), palladium acetate (2 mg, 0.0099 mmol, 4 mol%) and potassium phosphate (105 mg, 0.495 mmol, 2.0 equiv.), by analogy with **13a**, using DMF (2 mL) for 3 h. Flash column chromatography (silica gel, 10% EtOAc/petrol) gave desired product **13t** as a dark green oil (27 mg, 37%). R_f 0.53 (3:1 petrol:EtOAc); ^{1}H NMR (400 MHz, $CDCl_3$) δ (ppm) 9.62 (1H, s), 8.22 (1H, d, J = 2.0 Hz), 7.72 (1H, s), 7.48 (1H, dd, J = 10.7, 1.9 Hz), 7.36 (1H, d, J = 3.6 Hz), 7.13 (1H, d, J = 10.7 Hz), 6.58 (1H, d, J = 3.6 Hz), 3.11 (1H, septet, J = 7.0 Hz), 2.66 (3H, s), 2.64 (3H, s), 1.38 (6H, d, J

= 7.0 Hz); ^{13}C NMR (126 MHz, $CDCl_3$) δ (ppm) 176.9, 159.9, 151.9, 146.6, 142.5, 139.6, 139.5, 135.8, 134.4, 134.4, 129.2, 124.8, 123.6 (br), 114.5, 111.3, 37.9, 26.6, 24.6, 12.8; IR (neat) 2960, 1668, 1525, 1506, 1460, 1354, 1285, 1098, 1023, 967, 801, 767 cm^{-1}; HRMS (ESI+) *m/z* calcd. for ($C_{20}H_{20}O_2$) $[M+H]^+$ 293.1536, found 293.1578; calcd. for ($C_{20}H_{20}O_2$) $[M+Na]^+$ 315.1356, found 315.1340.

5-(4,8-Dimethyl-6-methoxyazulen-1-yl)furan-2-carbaldehyde 13u

The title compound was synthesized from 1-(4,8-dimethyl-6-methoxyazulen-1-yl)tetrahydro-1*H*-thiophen-1-ium hexafluorophosphate **11e** (20 mg, 0.048 mmol), 2-formylfuran-5-boronic acid **12i** (10.1 mg, 0.072 mmol, 1.5 equiv.), X-Phos (2.3 mg, 0.0048 mmol, 10 mol%), palladium acetate (0.4 mg, 0.0019 mmol, 4 mol%) and potassium phosphate (20 mg, 0.096 mmol, 2.0 equiv.), by analogy with **13a** (reaction time 3 h). Flash column chromatography (silica gel, 17% EtOAc/petrol) gave desired product **13u** as a red/orange gum (10.8 mg, 81%). R_f 0.33 (7:3 petrol:EtOAc); ^{1}H NMR (500 MHz, $CDCl_3$) δ (ppm) 9.63 (1H, s), 7.56 (1H, d, *J* = 4.2 Hz), 7.36 (1H, d, *J* = 3.6 Hz), 7.29 (1H, d, *J* = 4.2 Hz), 6.89 (1H, d, *J* = 2.6 Hz), 6.84 (1H, d, *J* = 2.6 Hz), 6.61 (1H, d, *J* = 3.6 Hz), 3.96 (3H, s), 2.90 (3H, s), 2.64 (3H, s); ^{13}C NMR (126 MHz, $CDCl_3$) δ (ppm) 177.0 (br), 165.0, 160.6, 152.0, 148.4, 147.3, 136.3, 133.9, 131.0, 123.4 (br), 118.4, 116.0, 115.9, 114.9, 111.1, 55.9, 27.6, 26.1; IR (neat) 2930, 2841, 1666, 1579, 1495, 1372, 1338, 1310, 1214, 1071, 1024, 966, 865, 767 cm^{-1}; HRMS (ESI+) *m/z* calcd. for ($C_{18}H_{16}O_3$) $[M+H]^+$ 281.1178, found 281.1151; calcd. for ($C_{18}H_{16}O_3$) $[M+Na]^+$ 303.0997, found 303.0970.

5-(3-(3,5-bis(Trifluoromethyl)phenyl)azulen-1-yl)furan-2-carbaldehyde 13v

The title compound was synthesized from 1-(3-(3,5-bis(trifluoromethyl)phenyl)azulen-1-yl)tetrahydro-1*H*-thiophen-1-ium hexafluorophosphate **11f** (100 mg, 0.175 mmol, 1 equiv.), 2-formylfuran-5-boronic acid **12i** (36.7 mg, 0.262 mmol, 1.5 equiv.), X-Phos (8.3 mg, 0.017 mmol, 10 mol%), palladium acetate (1.6 mg, 0.007 mmol, 4 mol%) and potassium phosphate (74 mg, 0.349 mmol, 2.0 equiv.) by analogy with **13a** (reaction time 4 h). Flash column chromatography (silica gel, 10% EtOAc/petrol) gave desired product **13v** as a dark green solid (18 mg, 23%). m.pt. 186 °C; R_f 0.38 (7:3 petrol:Et_2O); ^{1}H NMR (500 MHz, $CDCl_3$) δ (ppm) 9.66 (1H, s), 9.21 (1H, d, *J* = 9.9 Hz), 8.41 (1H, d, *J* = 9.5 Hz), 8.33 (1H, s) 8.03 (2H, s) 7.89 (1H, s) 7.79 (1H, t, *J* = 9.8 Hz), 7.47 (1H, t, *J* = 9.9 Hz), 7.42 (1H, d, *J* = 3.8 Hz), 7.38 (1H, t, *J* = 9.7 Hz), 6.92 (1H, d, *J* = 3.8 Hz); ^{13}C NMR (126 MHz, $CDCl_3$) δ (ppm) 176.3, 157.9, 151.6, 140.7, 139.2, 138.5, 138.2, 136.9, 136.2,

136.1, 132.1 (q, $^{2}J_{CF}$ = 33.2 Hz), 129.5 (q, $^{3}J_{CF}$ = 3.3 Hz), 128.4, 127.0, 126.9, 124.6 (br??), 123.4 (q, $^{1}J_{CF}$ = 272.8 Hz), 120.4 (septet, $^{3}J_{CF}$ = 3.8 Hz), 117.0, 108.6; ^{19}F NMR (470.5 MHz, $CDCl_3$) δ (ppm) –62.8 (s); IR (neat) 2925, 2829, 1656, 1568, 1529, 1510, 1365, 1277, 1171, 1124, 1094, 1031, 994, 967, 883, 845, 800, 770, 744, 709, 683 cm^{-1}; HRMS (ESI+) *m/z* calcd. for ($C_{23}H_{12}F_6O_2$) $[M+H]^+$ 435.0820, found 435.0855; calcd. for ($C_{23}H_{12}F_6O_2$) $[M+Na]^+$ 457.0639, found 457.0655.

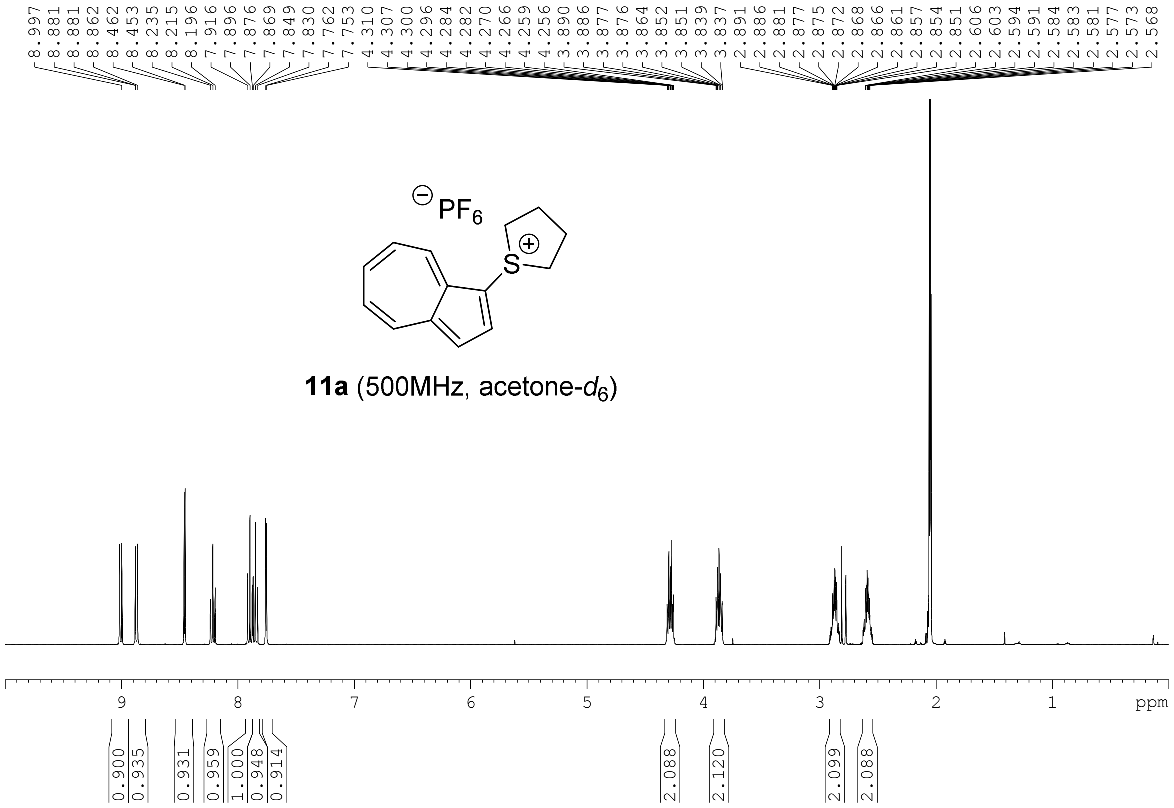
PF6
S
11a (500MHz, acetone-d6)
ppm

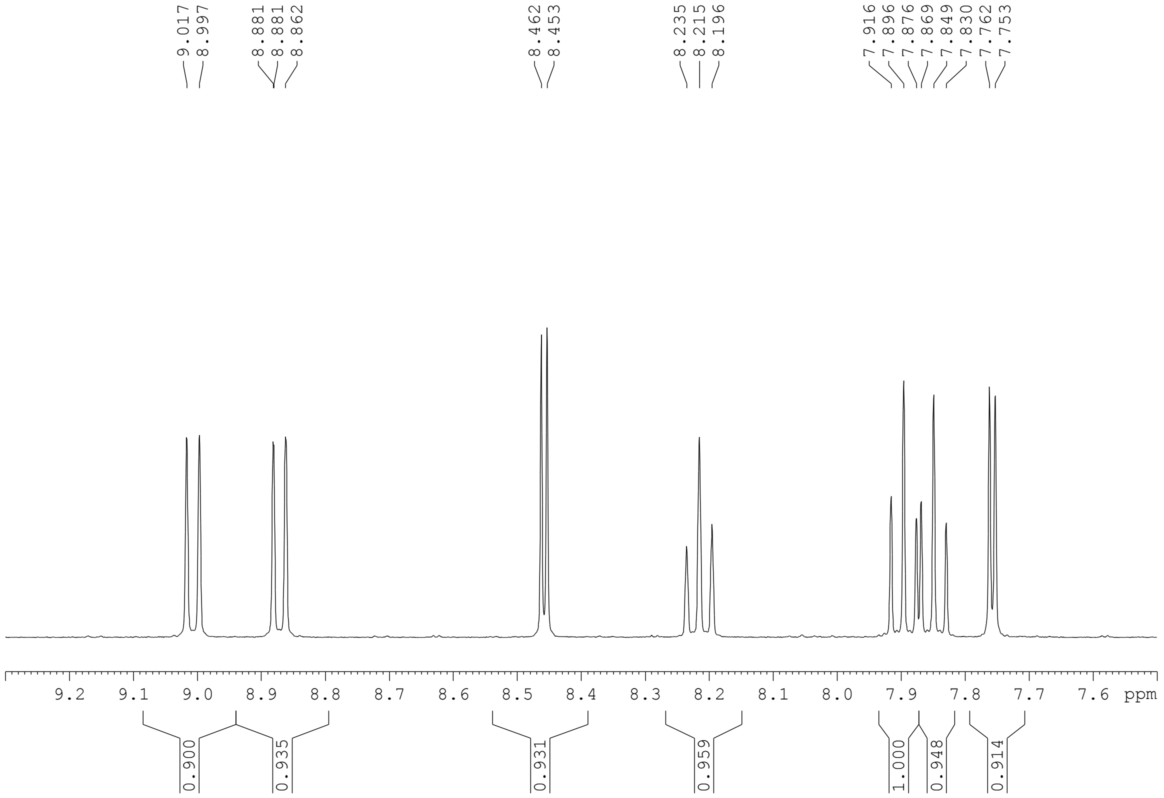
ppm

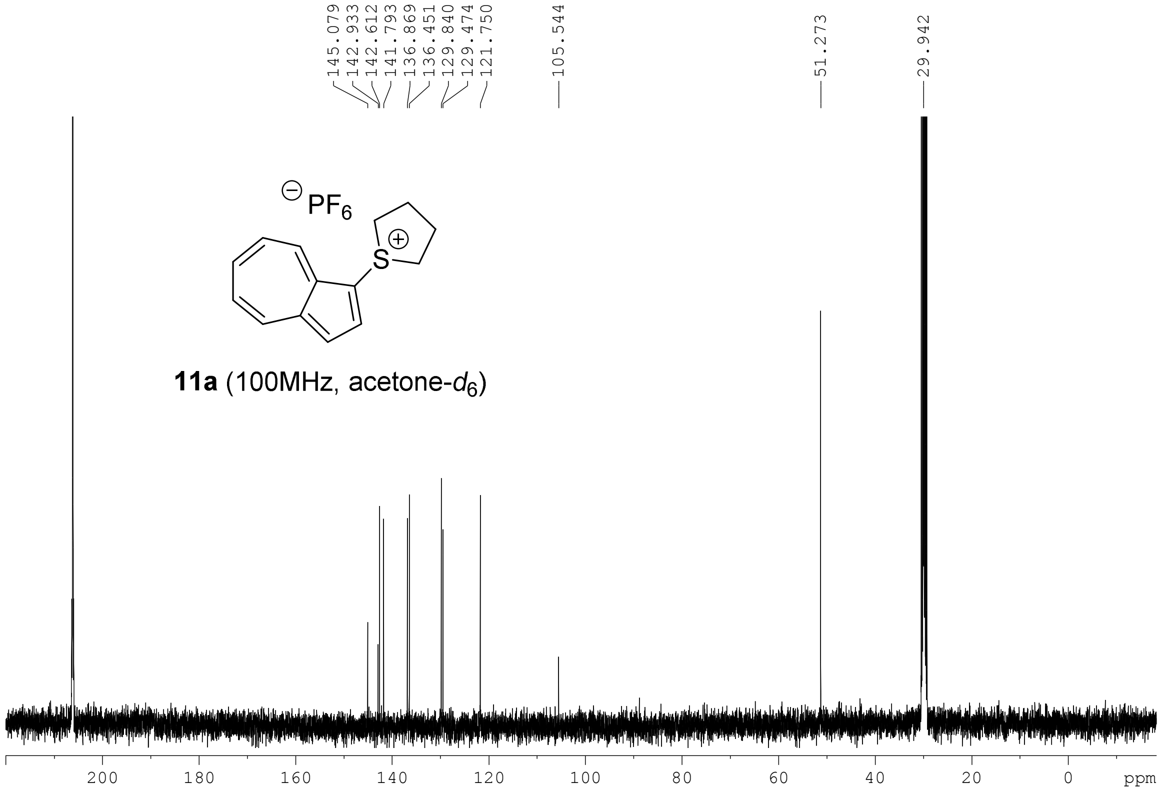
145.079
142.933
142.612
141.793
136.869
136.451
129.840
129.474
121.750
105.544
51.273
29.942
PF6
S
11a (100MHz, acetone-d6)
200
180
160
140
120
100
80
60
40
20
0
ppm

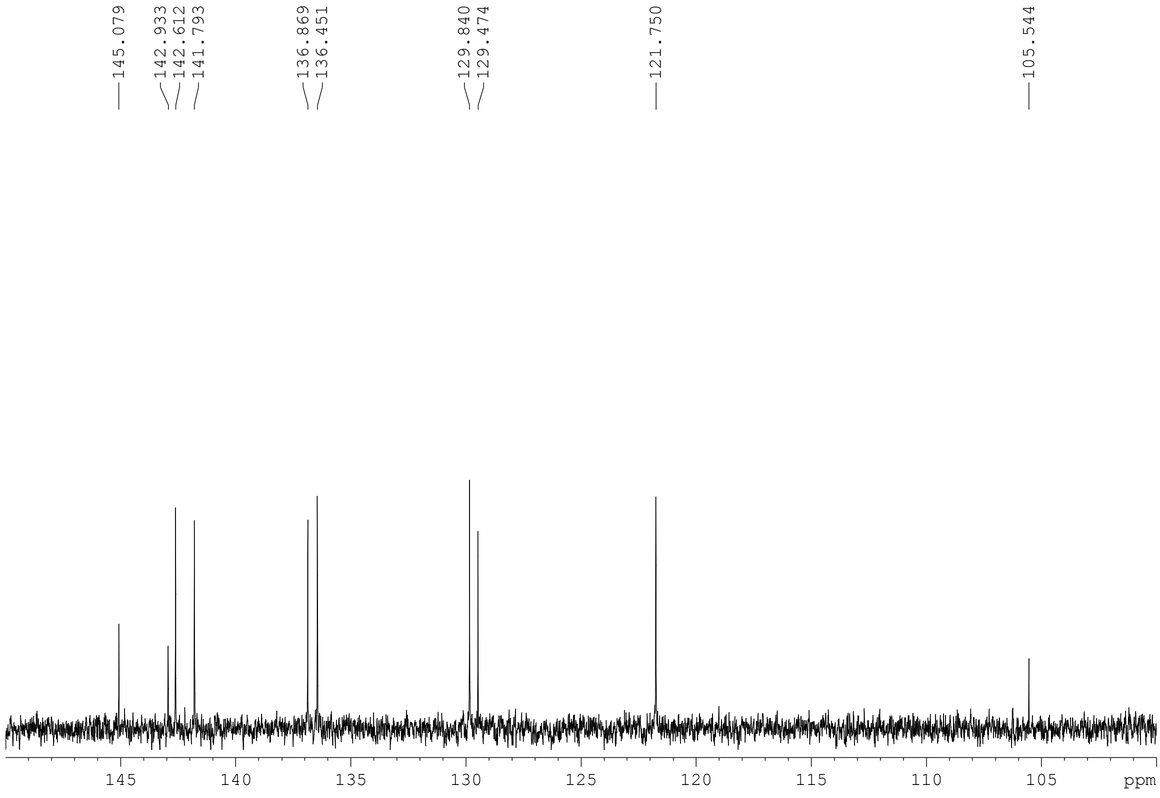
145.079
142.933
142.612
141.793
136.869
136.451
129.840
129.474
121.750
105.544
145
140
135
130
125
120
115
110
105
ppm

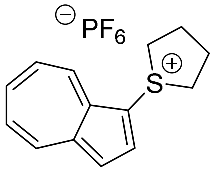

11a (100MHz, acetone-d_6)

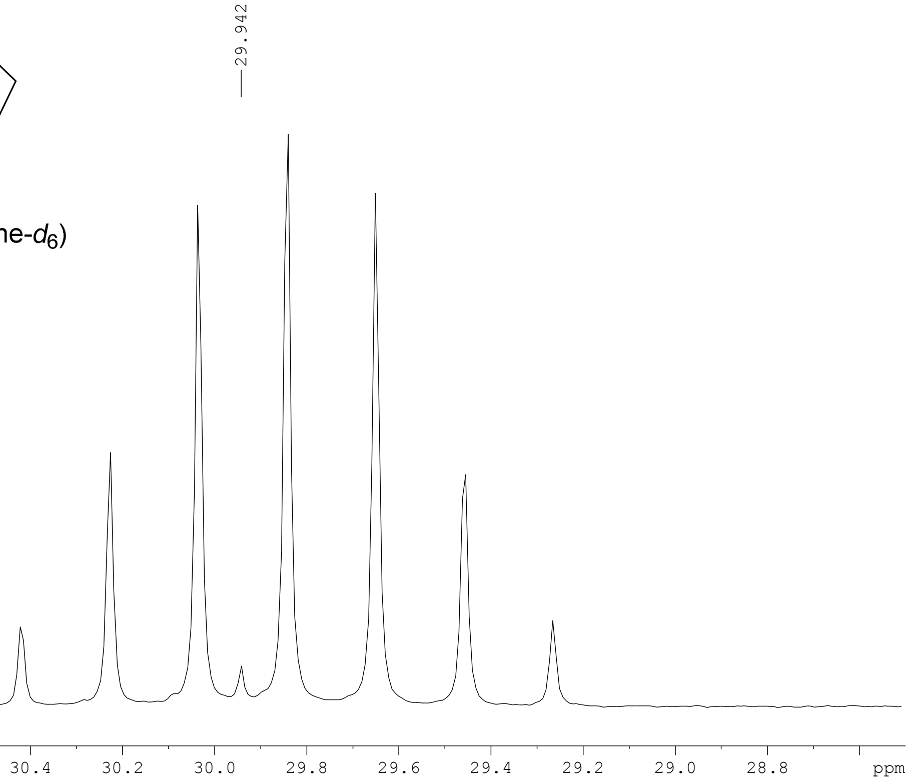

COSY

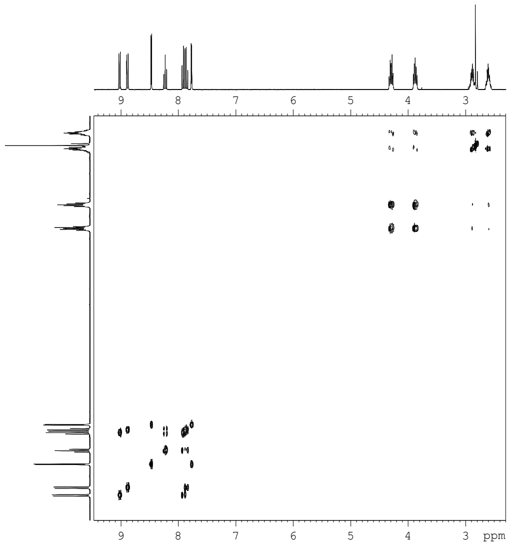

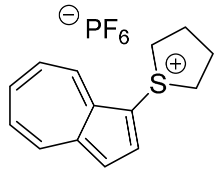

11a (500MHz, acetone-d_6)

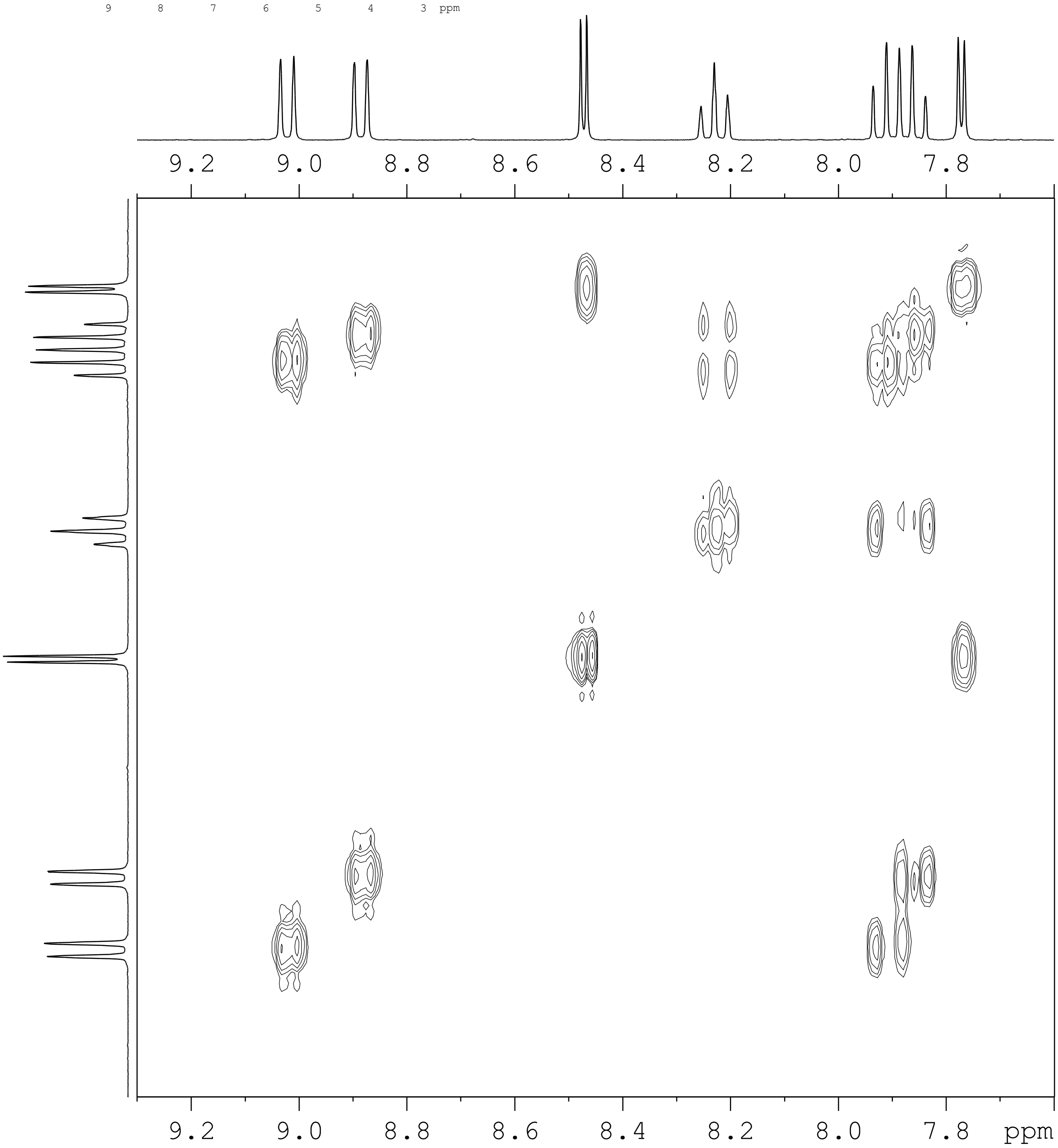

NOESY

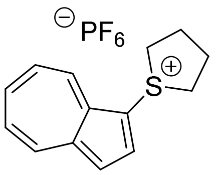

11a (500MHz, acetone-d_6)

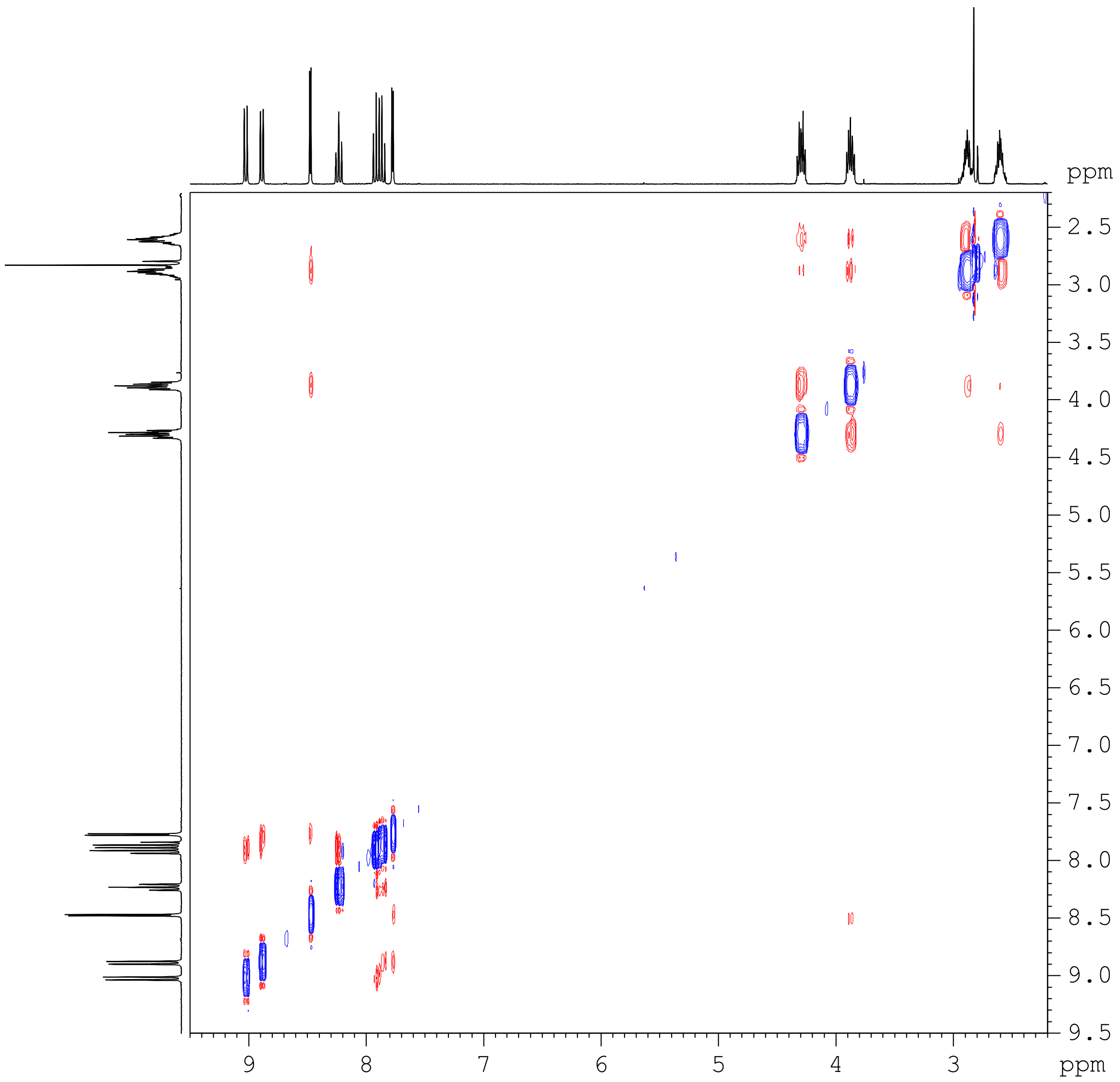

NOESY

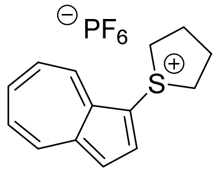

11a (500MHz, acetone-d_6)

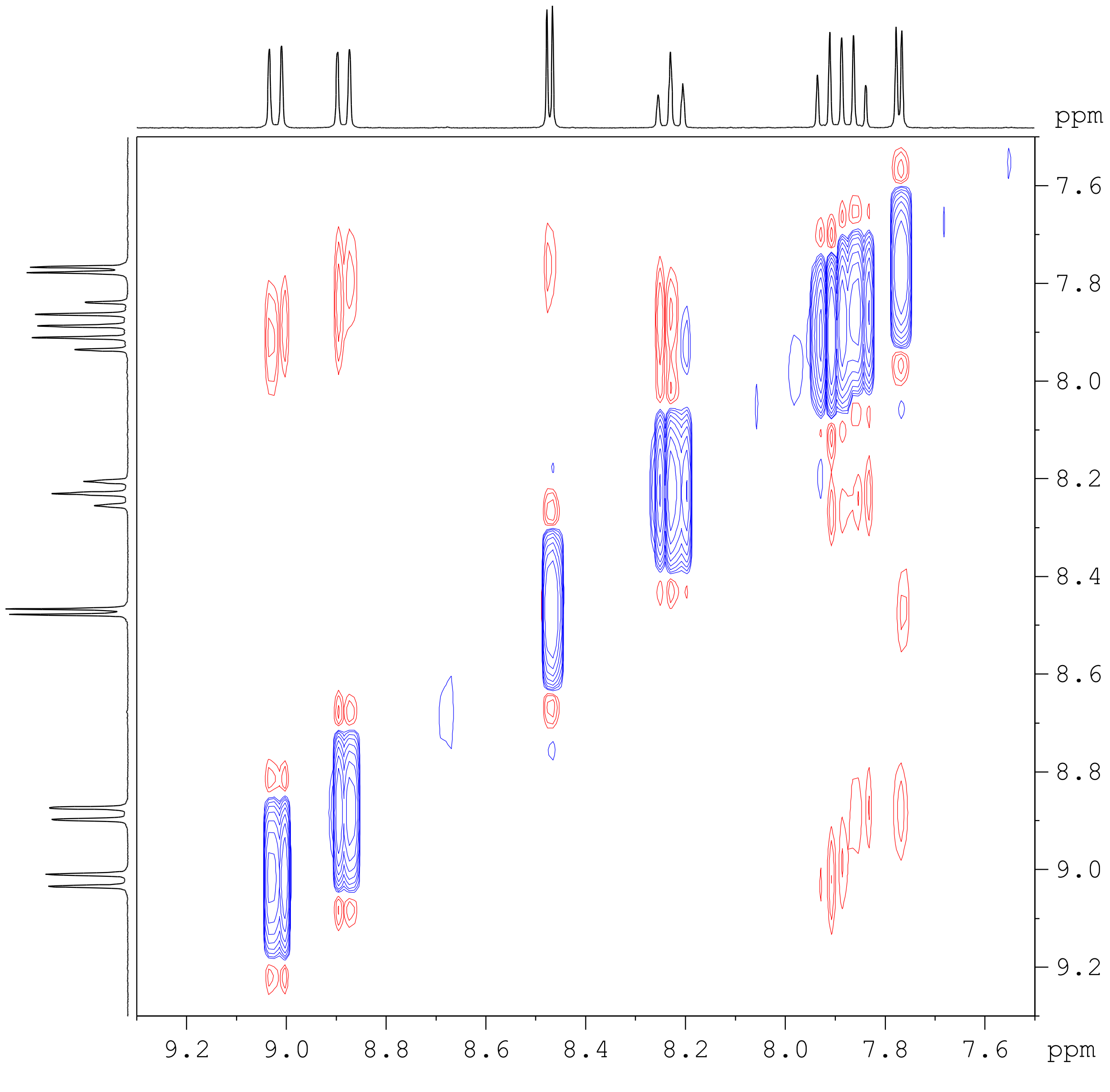

HSQC

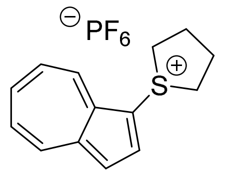

11a (500MHz, acetone-d_6)

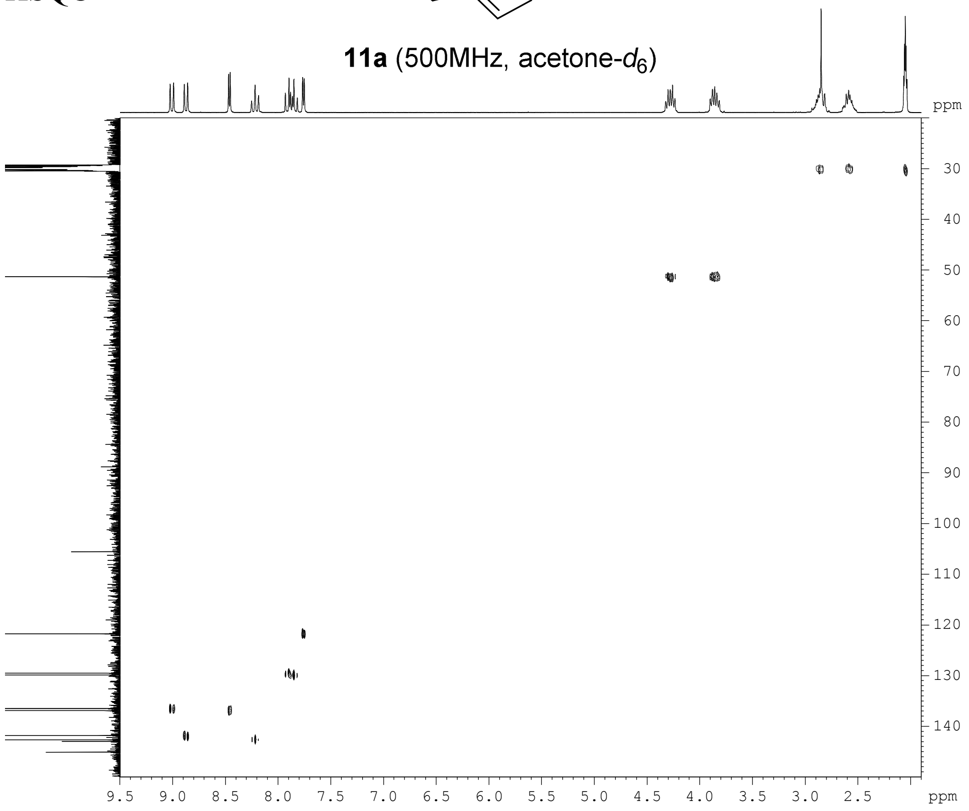

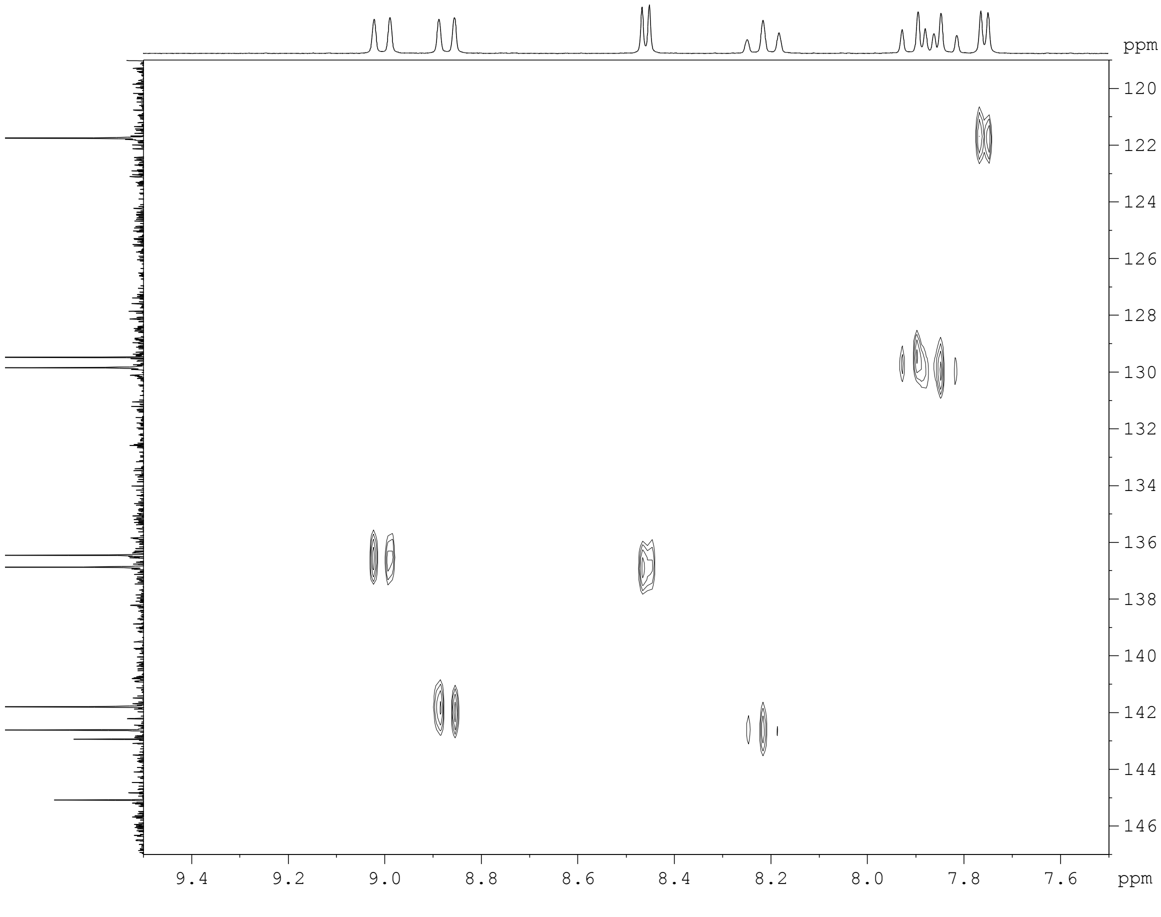

HMBC

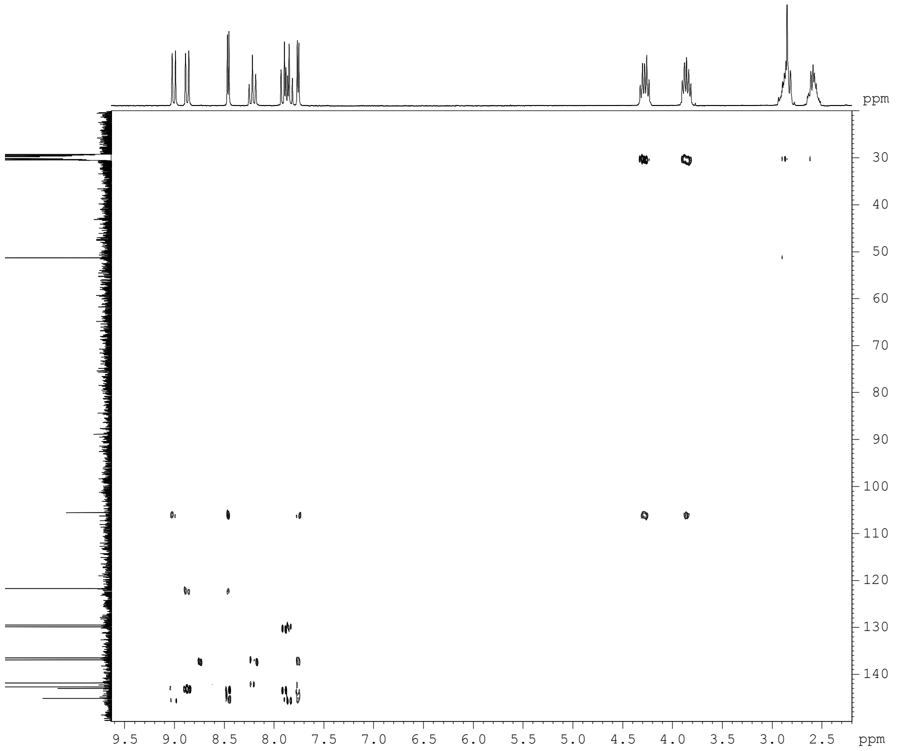

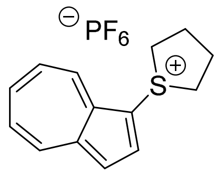

11a (500MHz, acetone-d_6)

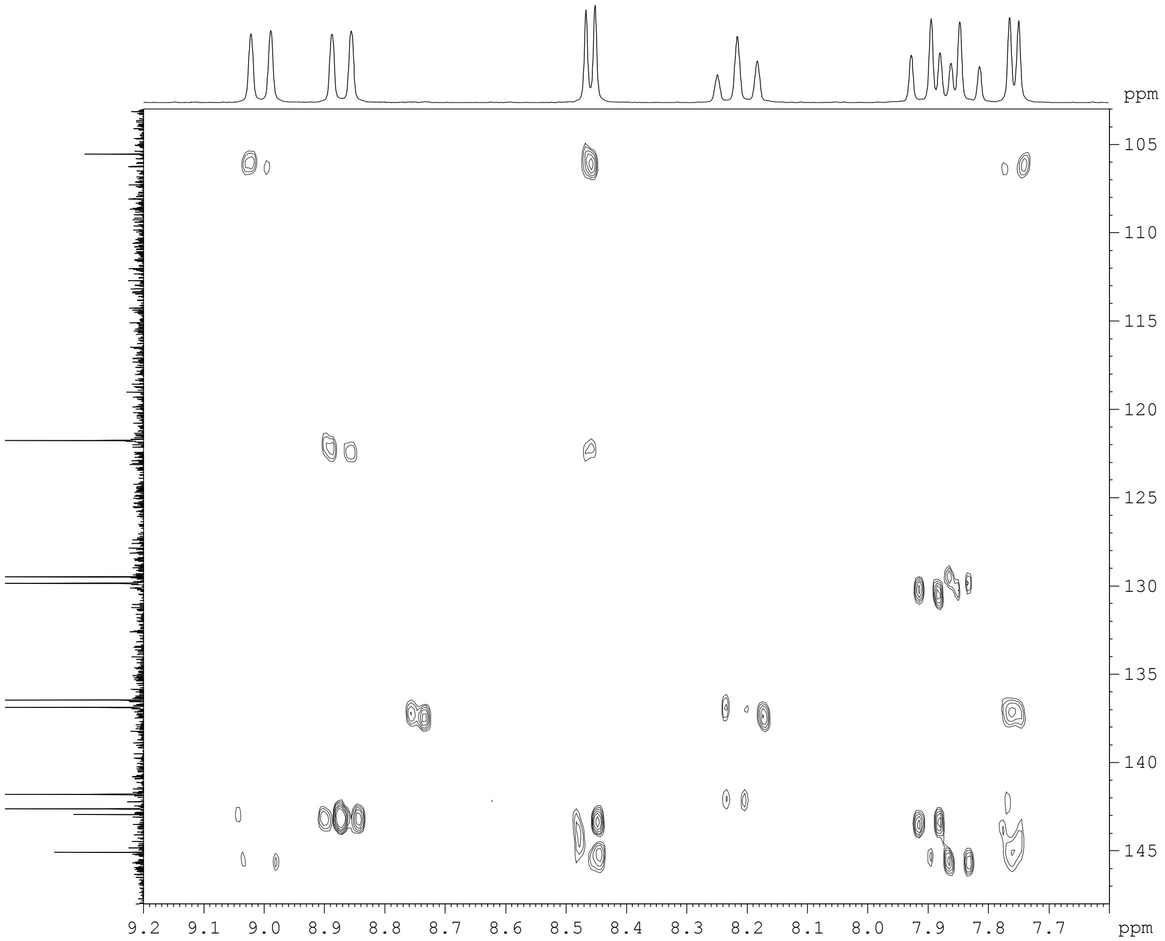

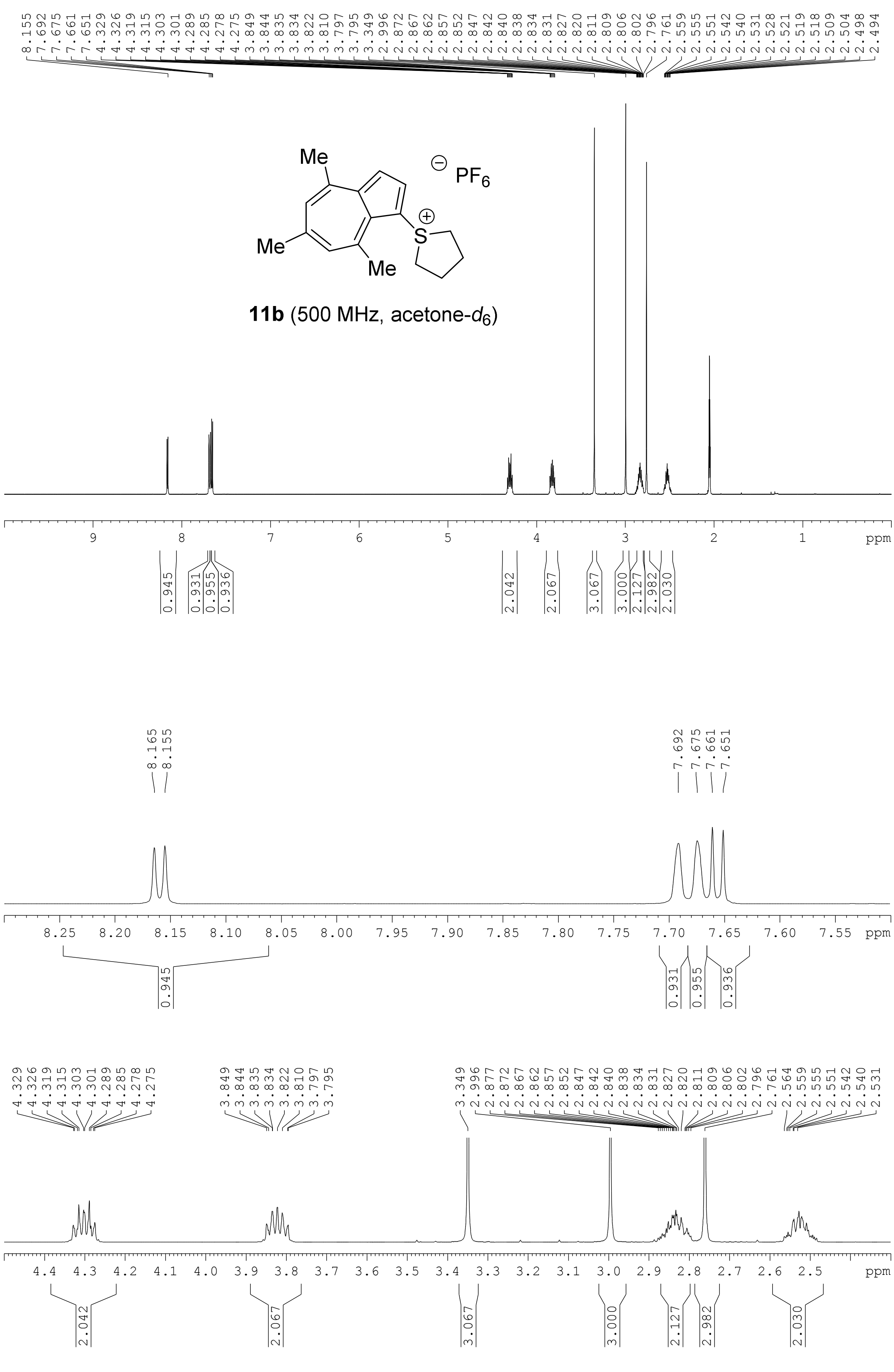

11b (500 MHz, acetone-d_6)

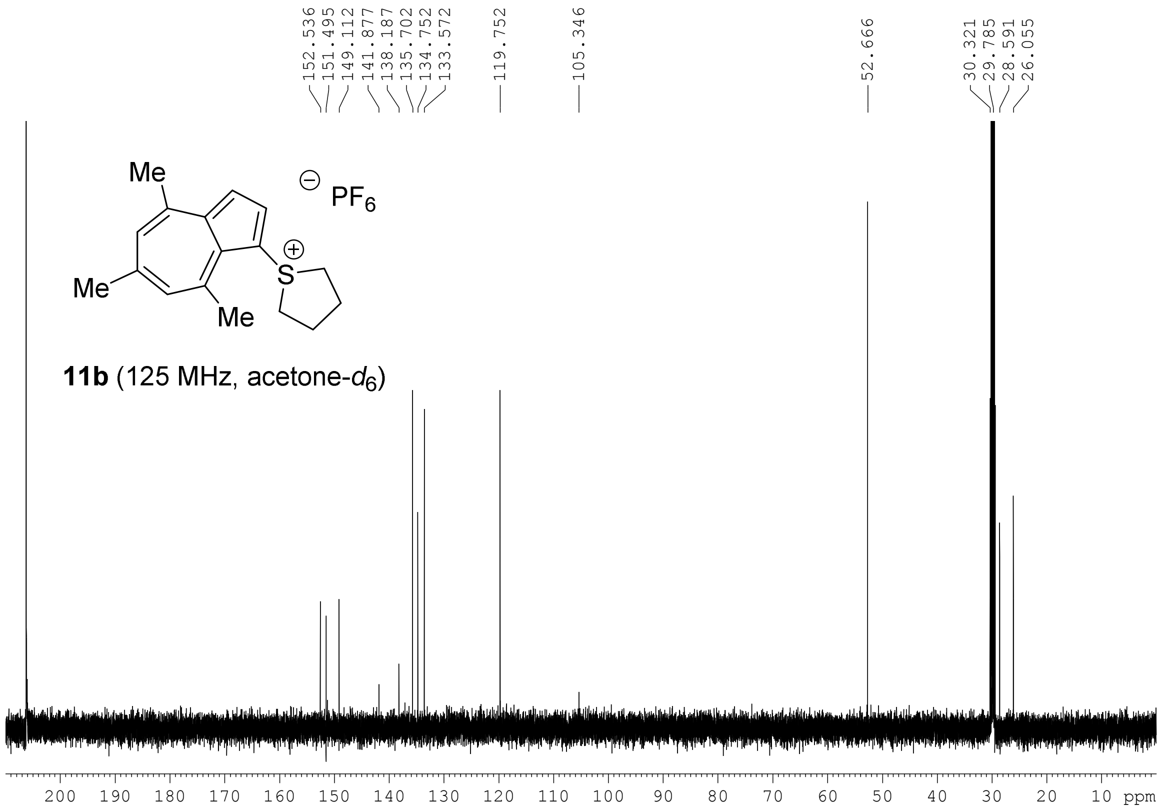

11b (125 MHz, acetone-d_6)

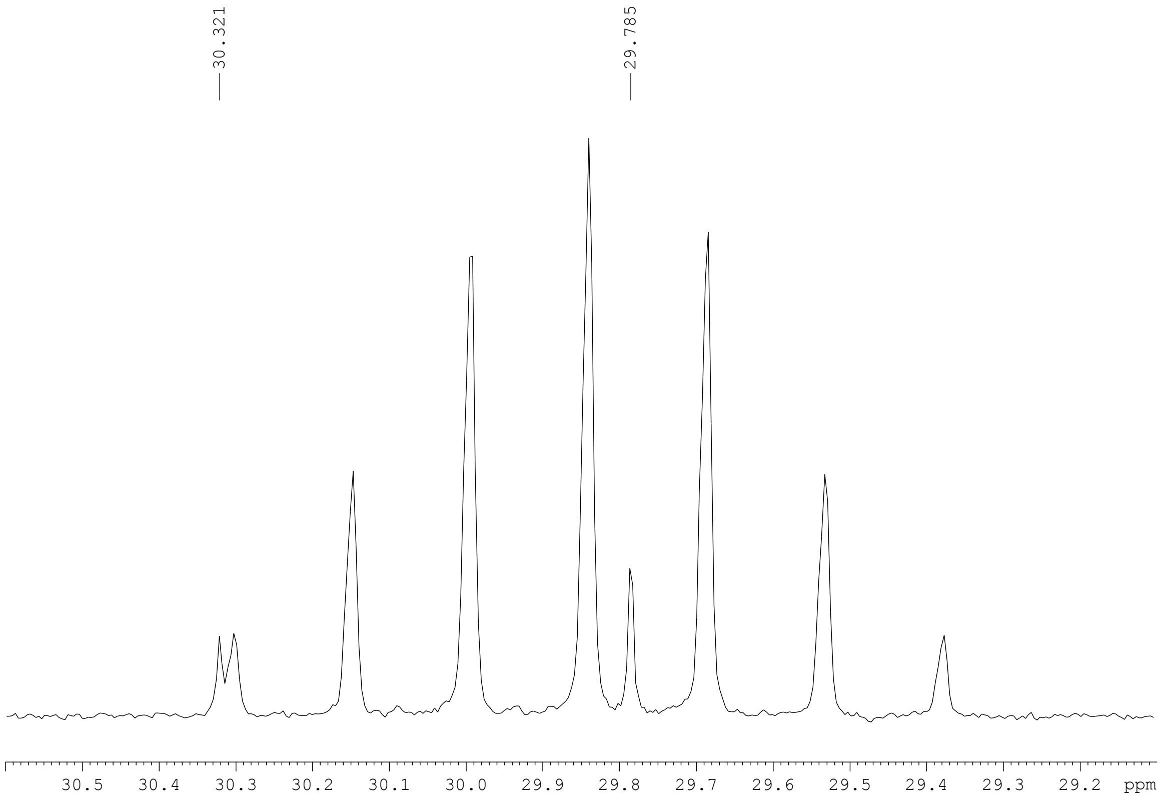

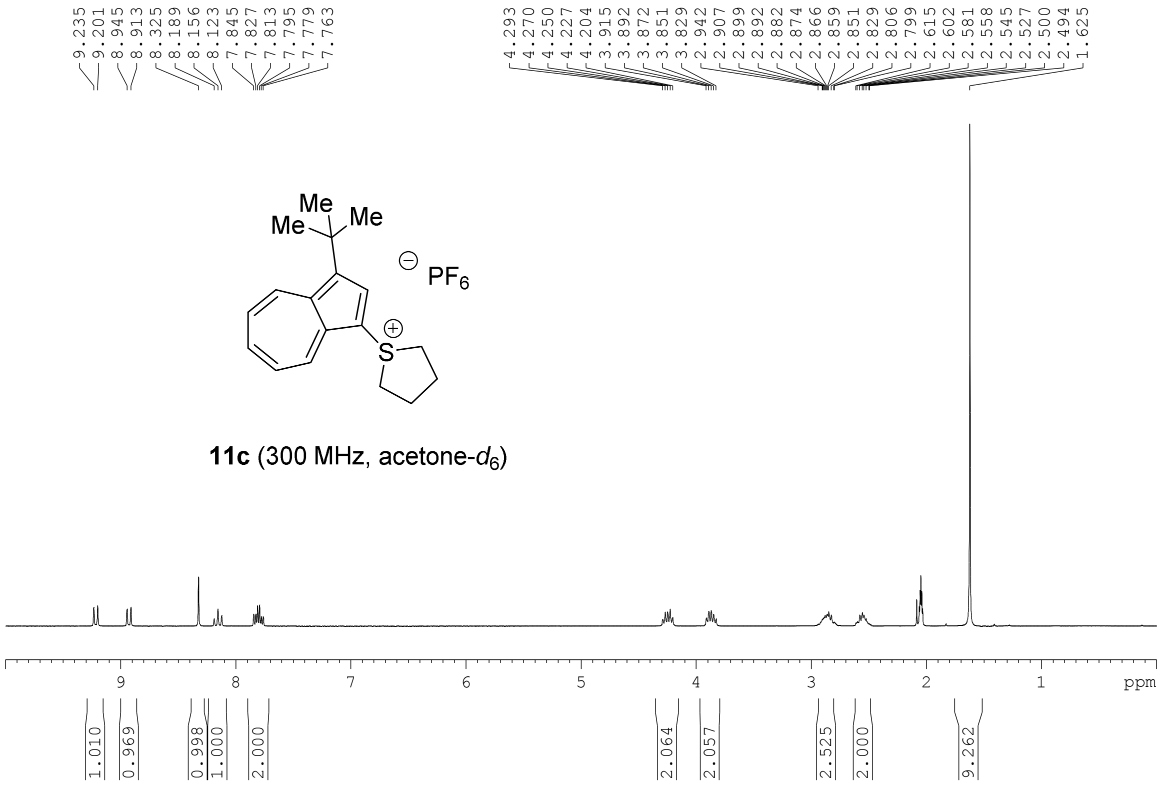

11c (300 MHz, acetone-d_6)

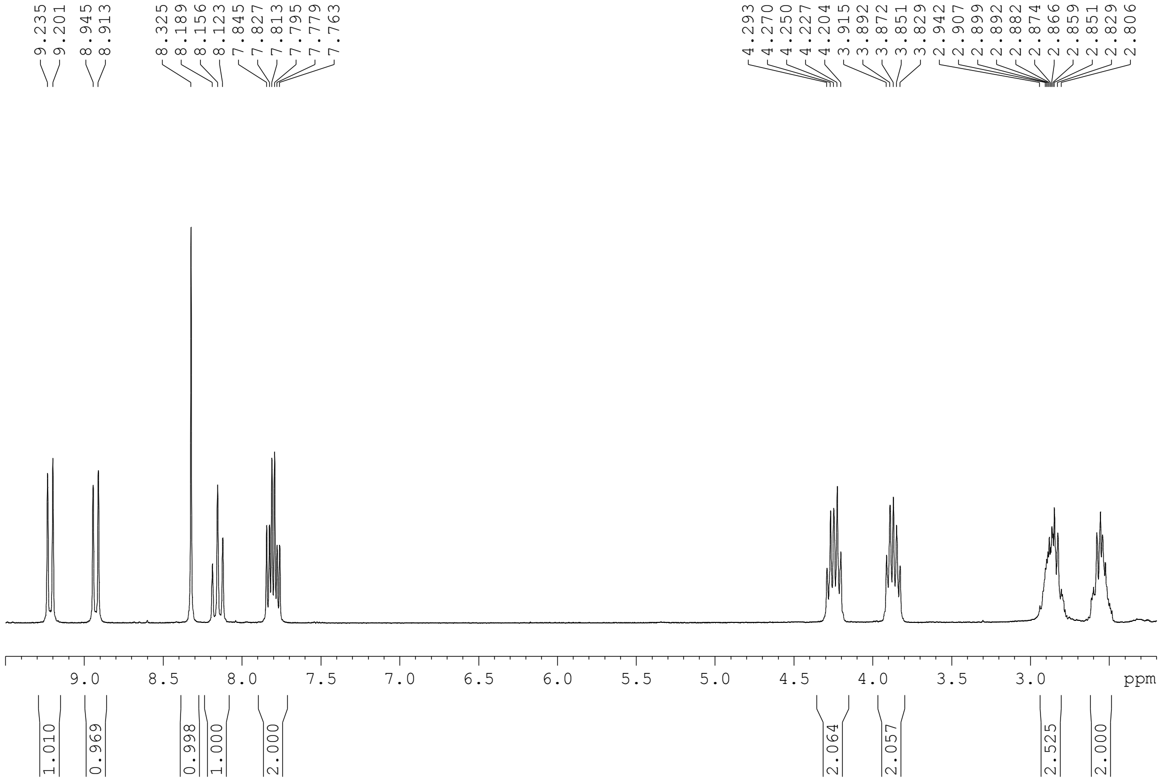

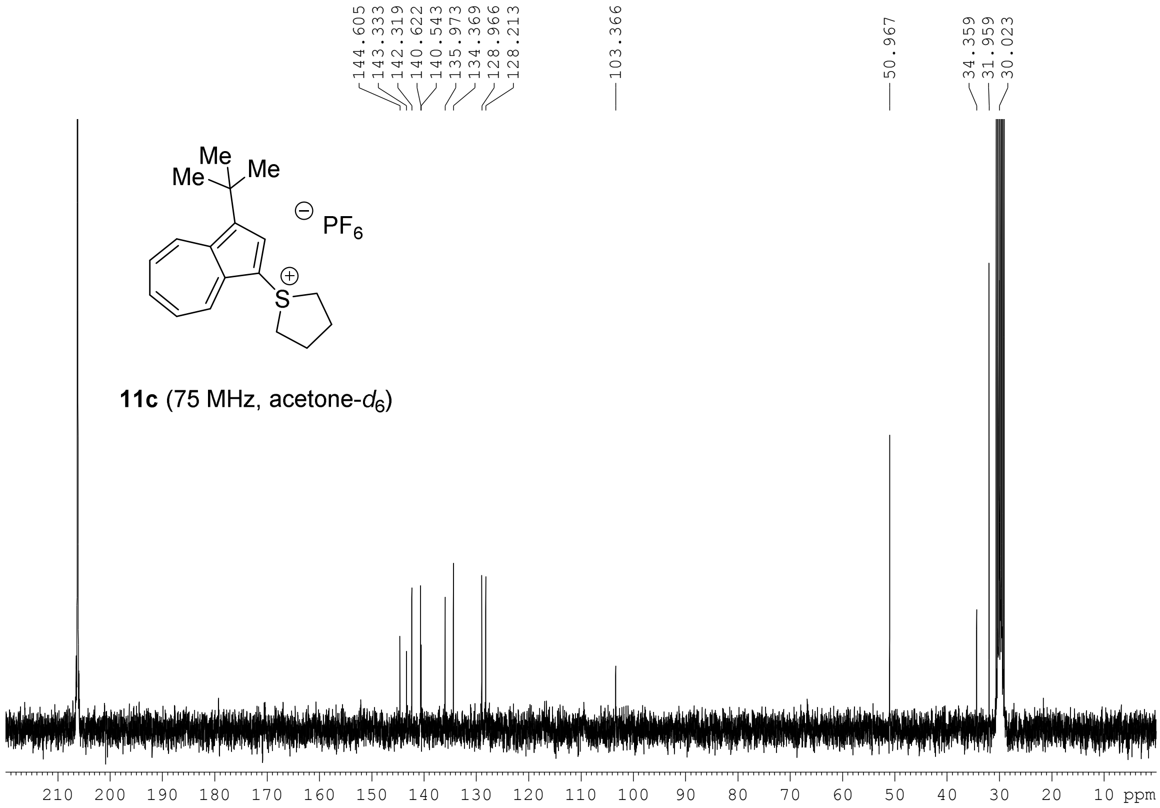
144.605
143.333
142.319
140.622
140.543
135.973
134.369
128.966
128.213
103.366
50.967
34.359
31.959
30.023
Me
Me
Me
PF6
S
11c (75 MHz, acetone-d6)
210 200 190 180 170 160 150 140 130 120 110 100 90 80 70 60 50 40 30 20 10 ppm

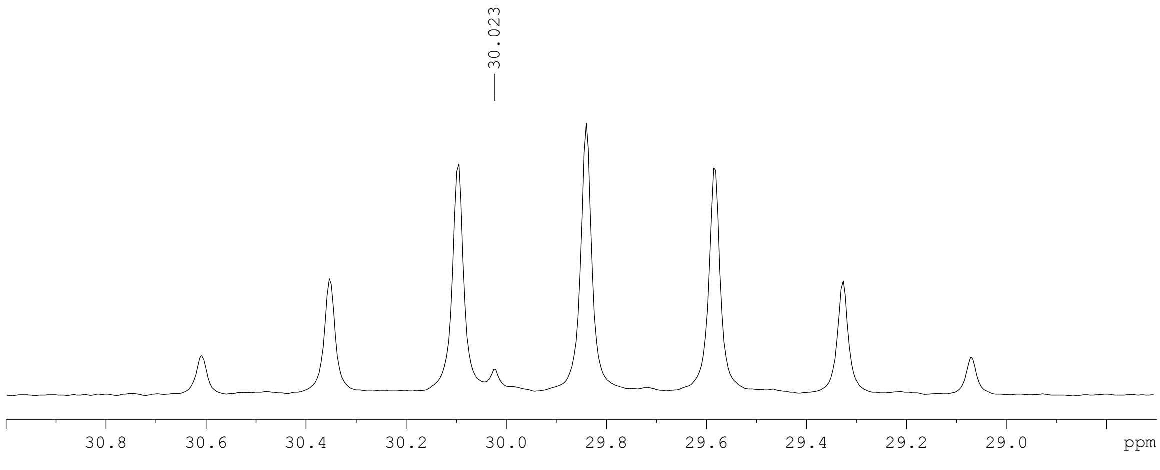
30.023
30.8 30.6 30.4 30.2 30.0 29.8 29.6 29.4 29.2 29.0 ppm

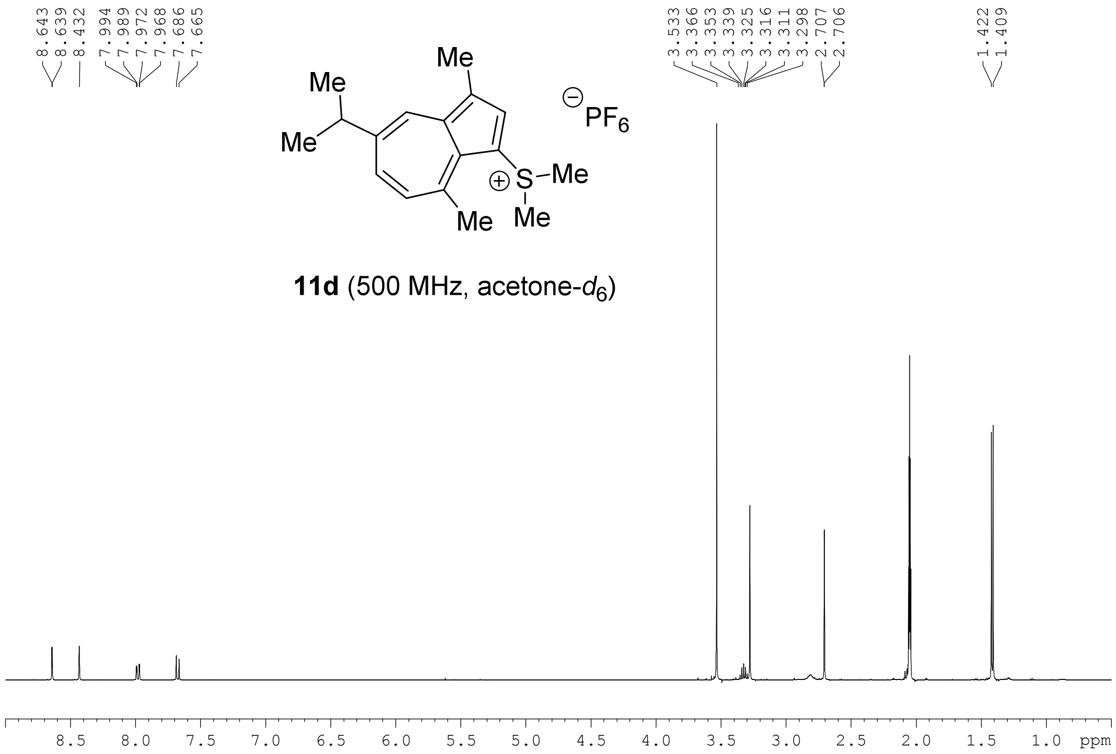

11d (500 MHz, acetone-d_6)

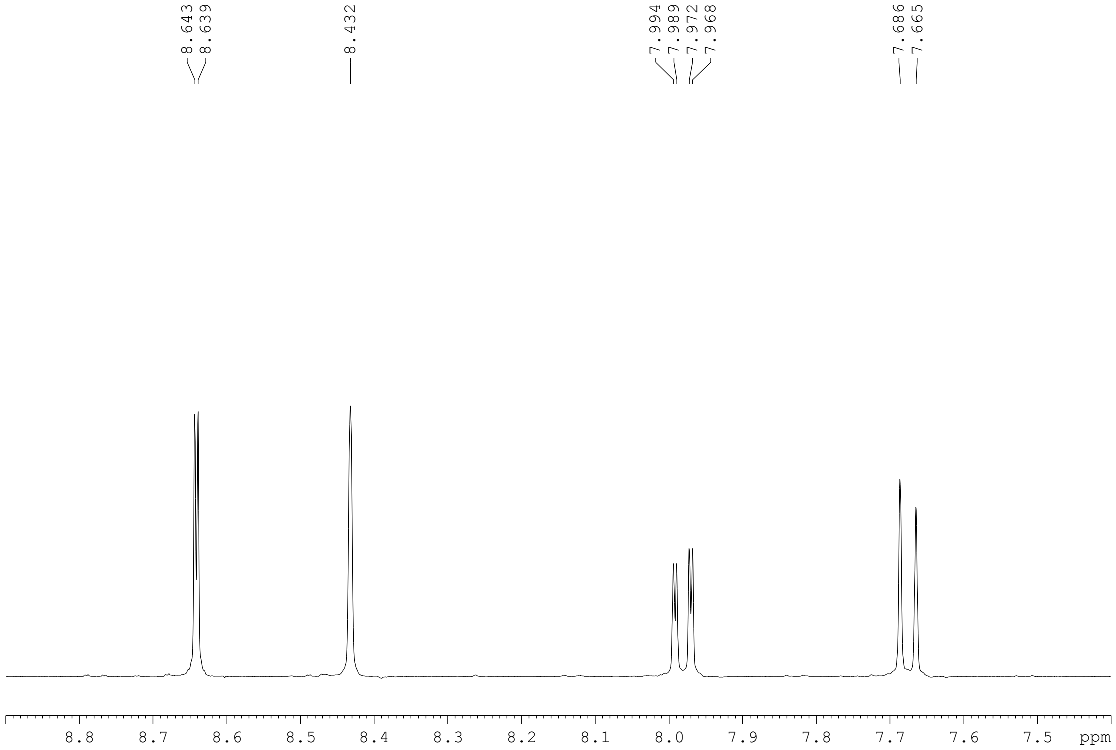

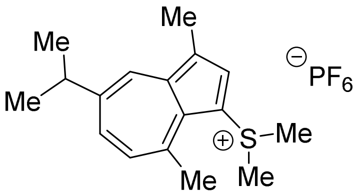

11d (126 MHz, acetone-d_6)

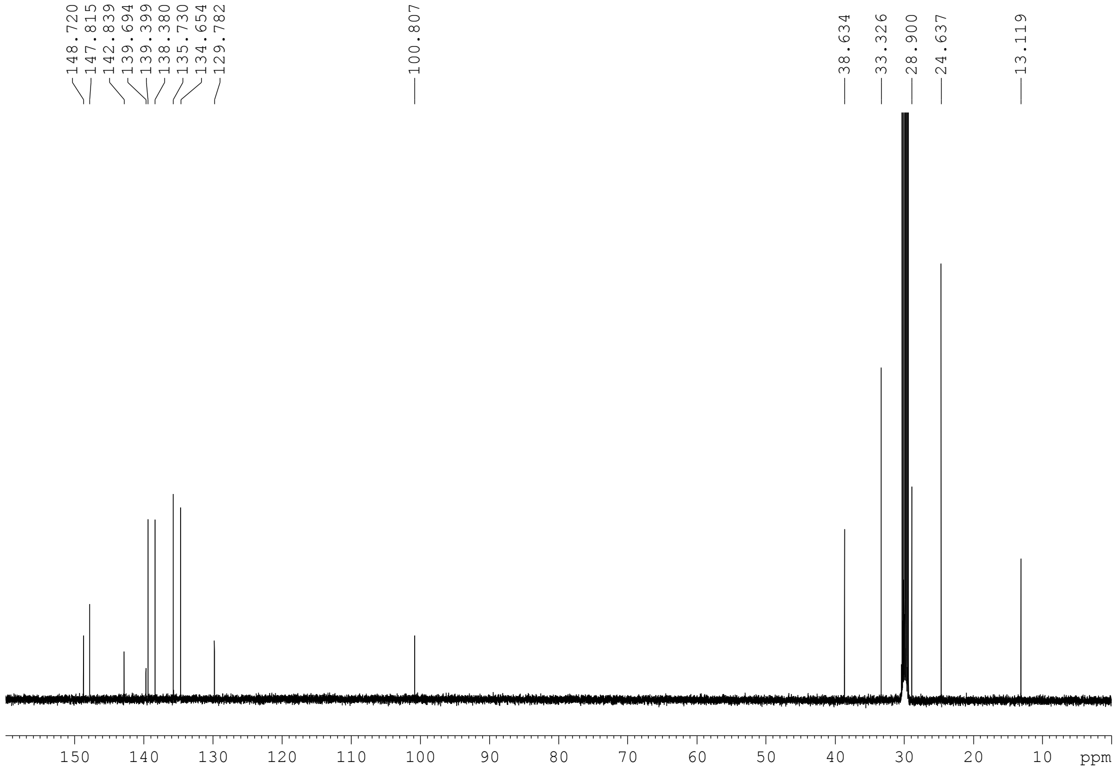

NOESY

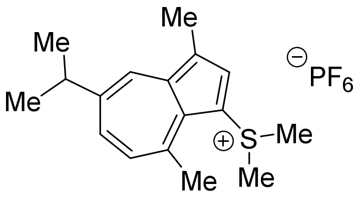

11d (500 MHz, acetone-d_6)

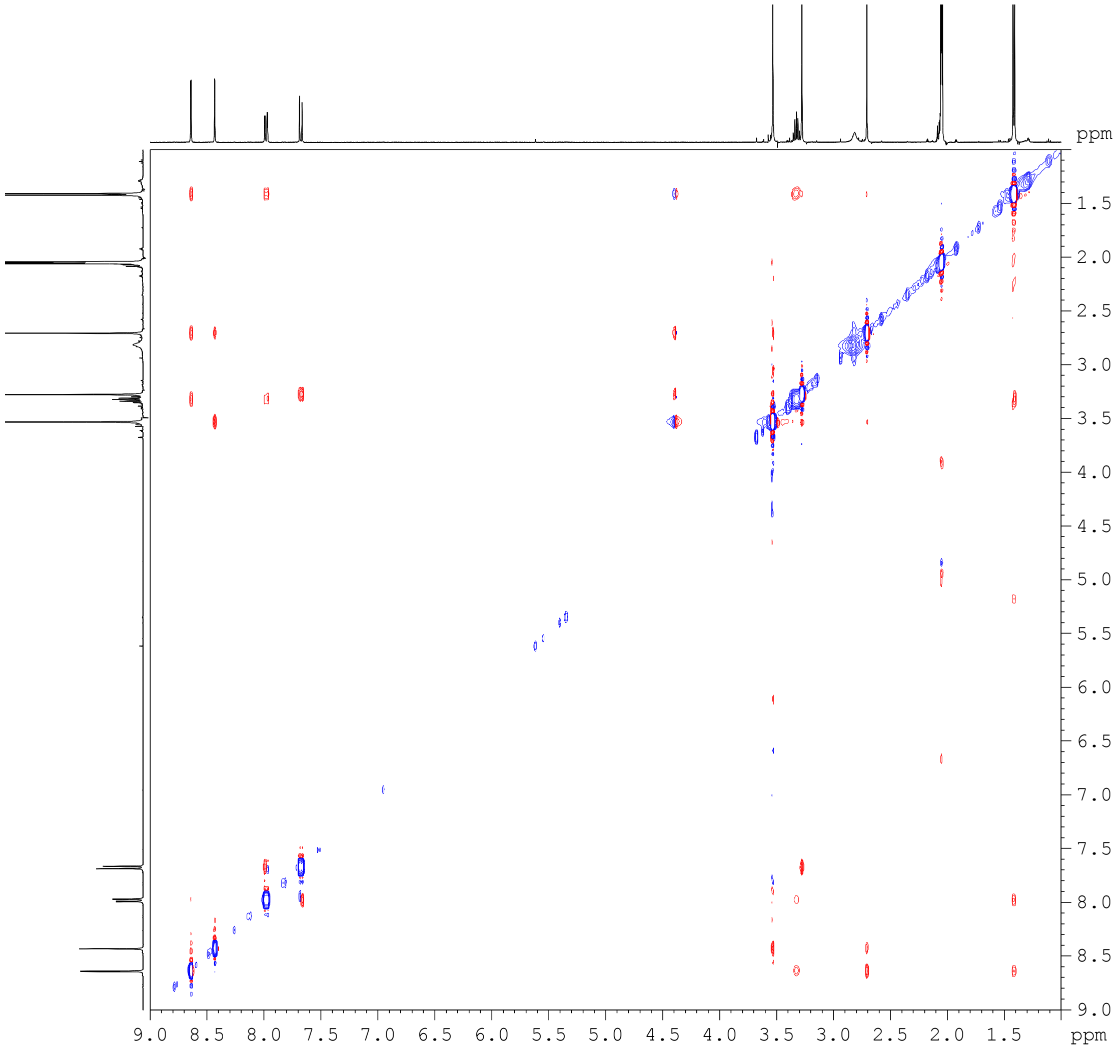

HSQC

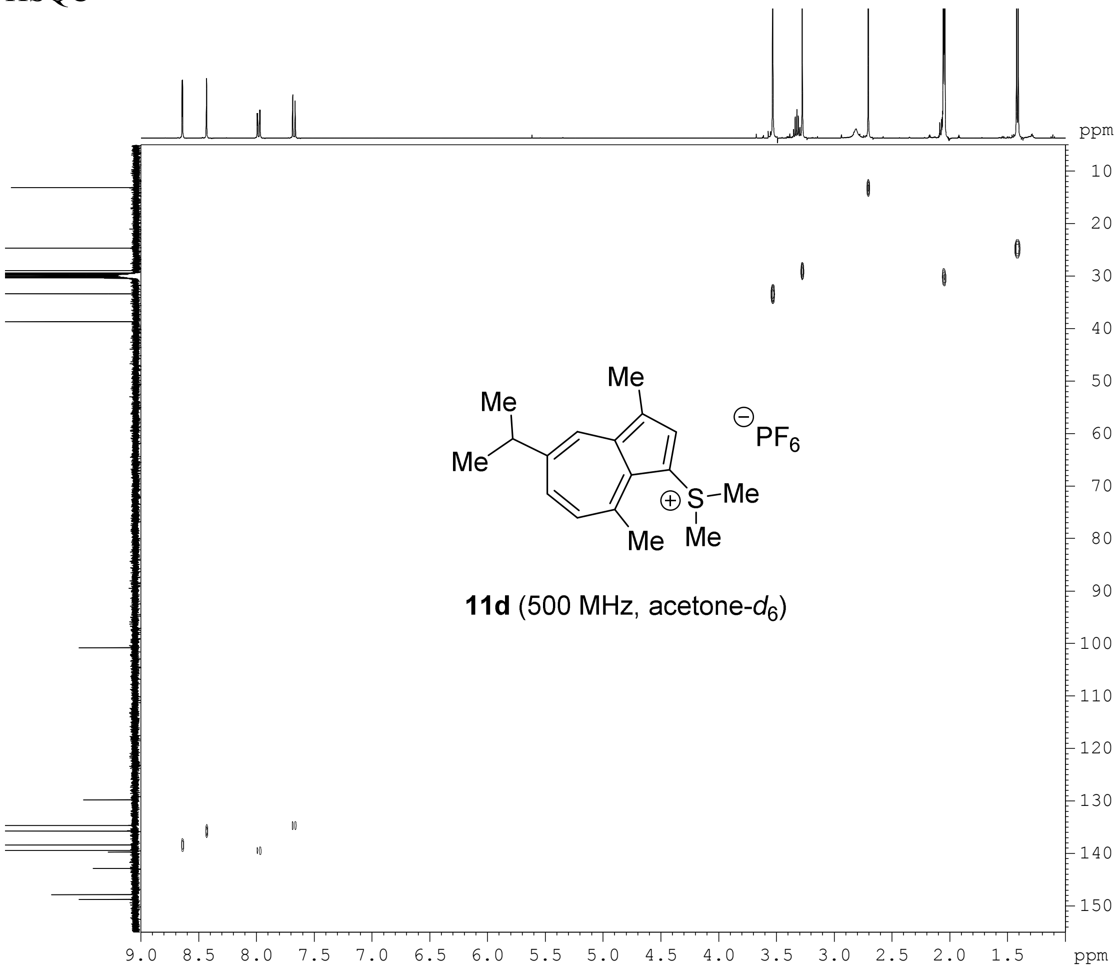
Me
Me
Me
PF6
S–Me
Me
Me
11d (500 MHz, acetone-d6)
ppm
9.0 8.5 8.0 7.5 7.0 6.5 6.0 5.5 5.0 4.5 4.0 3.5 3.0 2.5 2.0 1.5
10 20 30 40 50 60 70 80 90 100 110 120 130 140 150

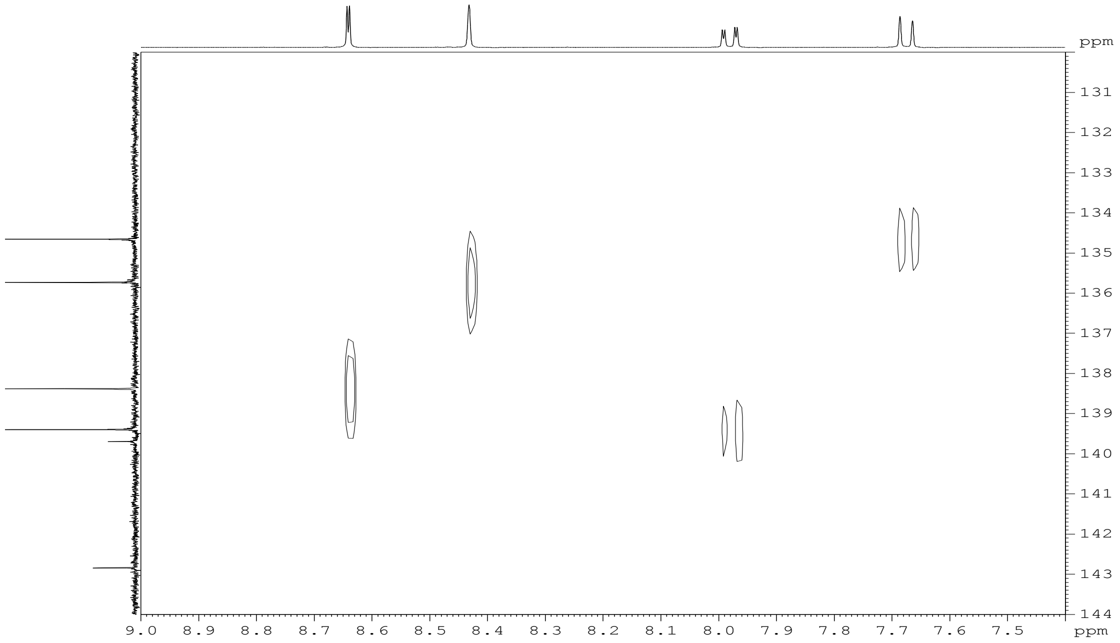
ppm
9.0 8.9 8.8 8.7 8.6 8.5 8.4 8.3 8.2 8.1 8.0 7.9 7.8 7.7 7.6 7.5
131 132 133 134 135 136 137 138 139 140 141 142 143 144

HMBC

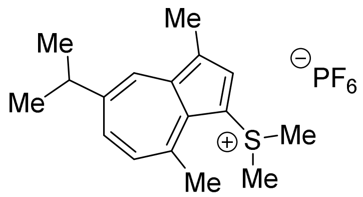

11d (500 MHz, acetone-d_6)

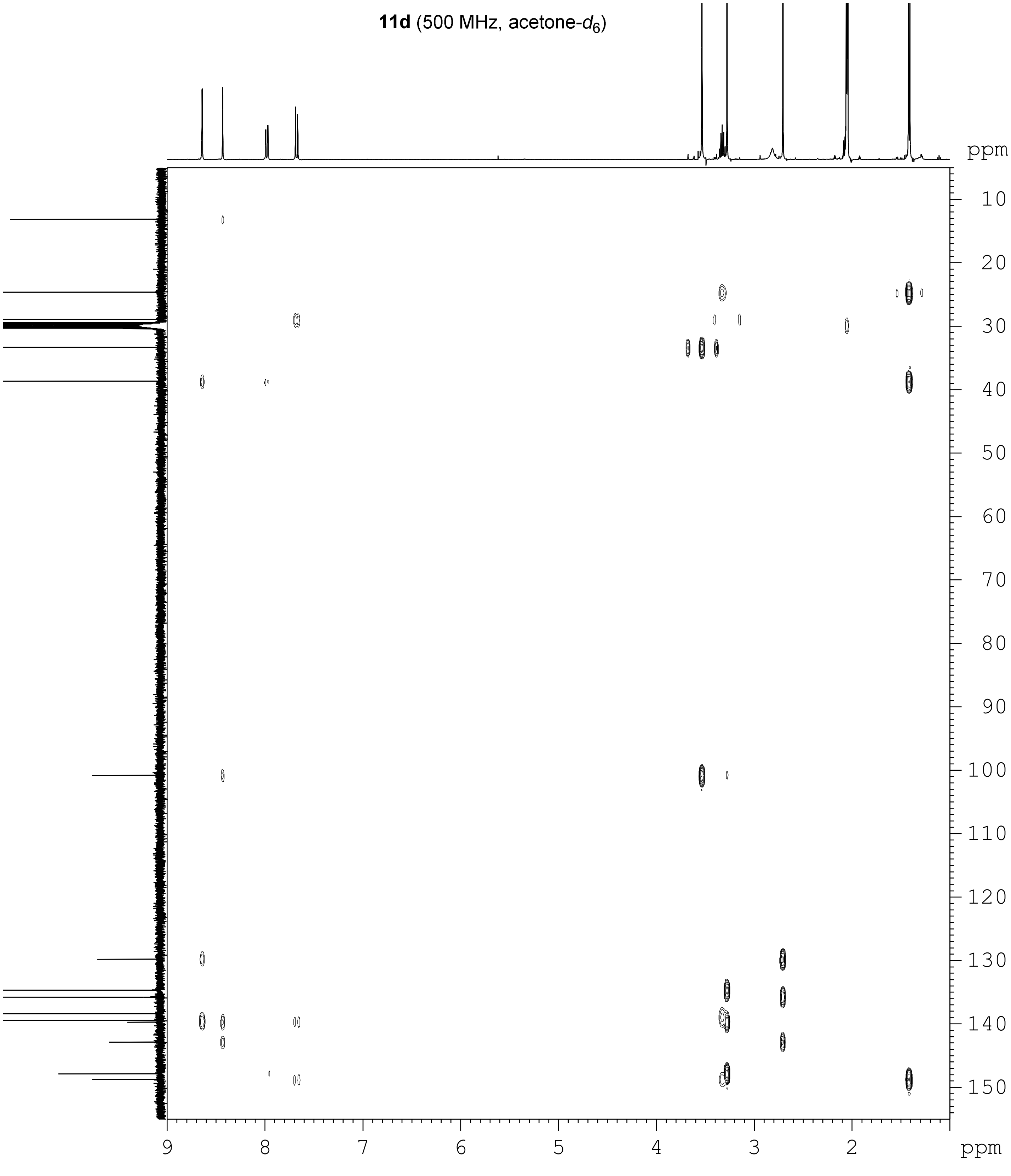

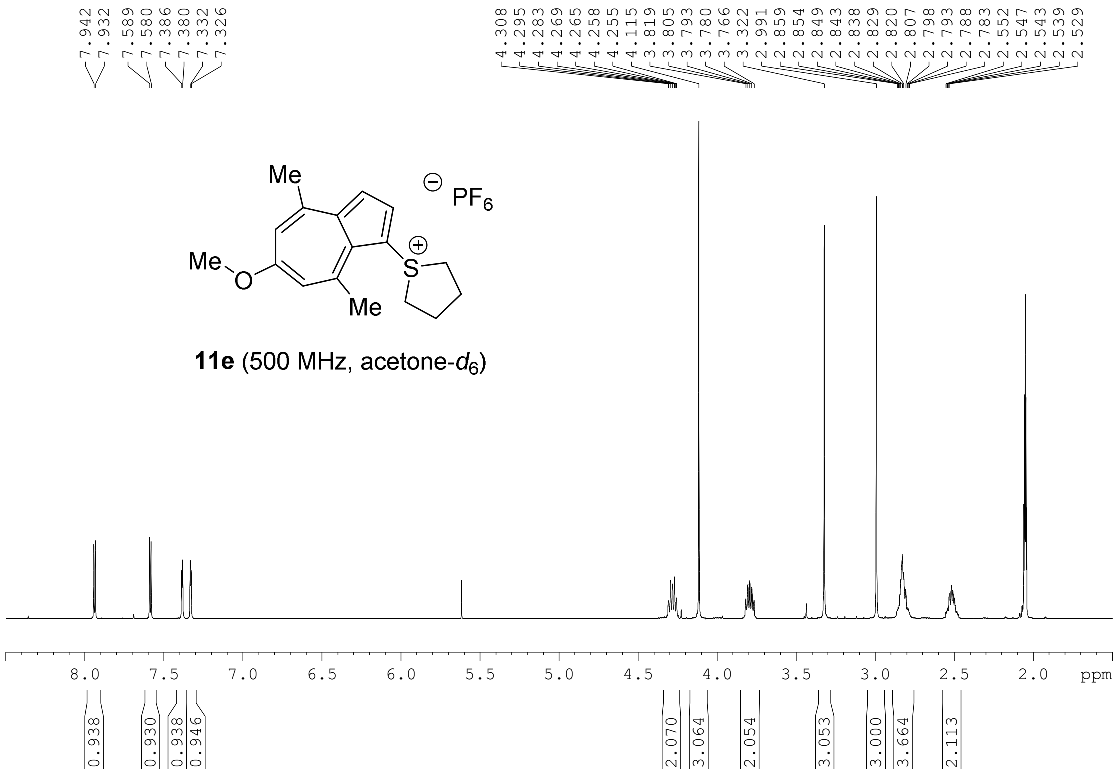

11e (500 MHz, acetone-d_6)

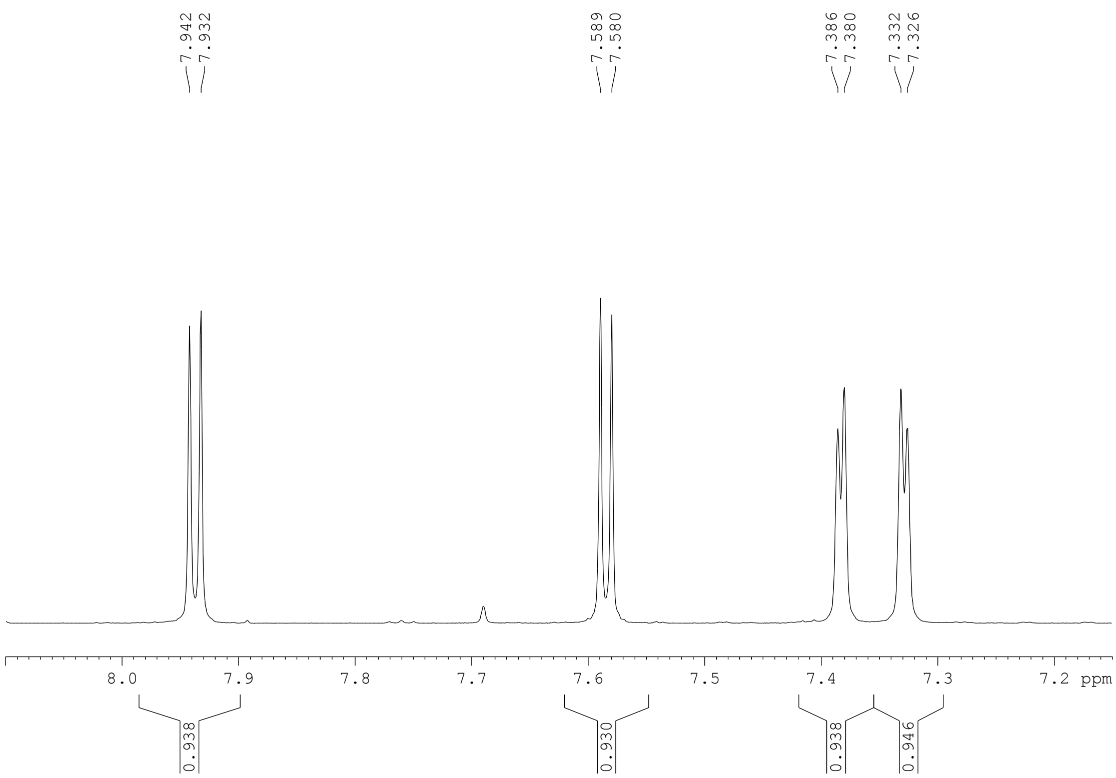

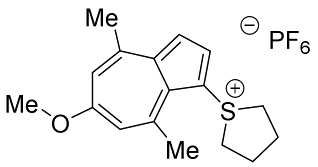

11e (500 MHz, acetone-d_6)

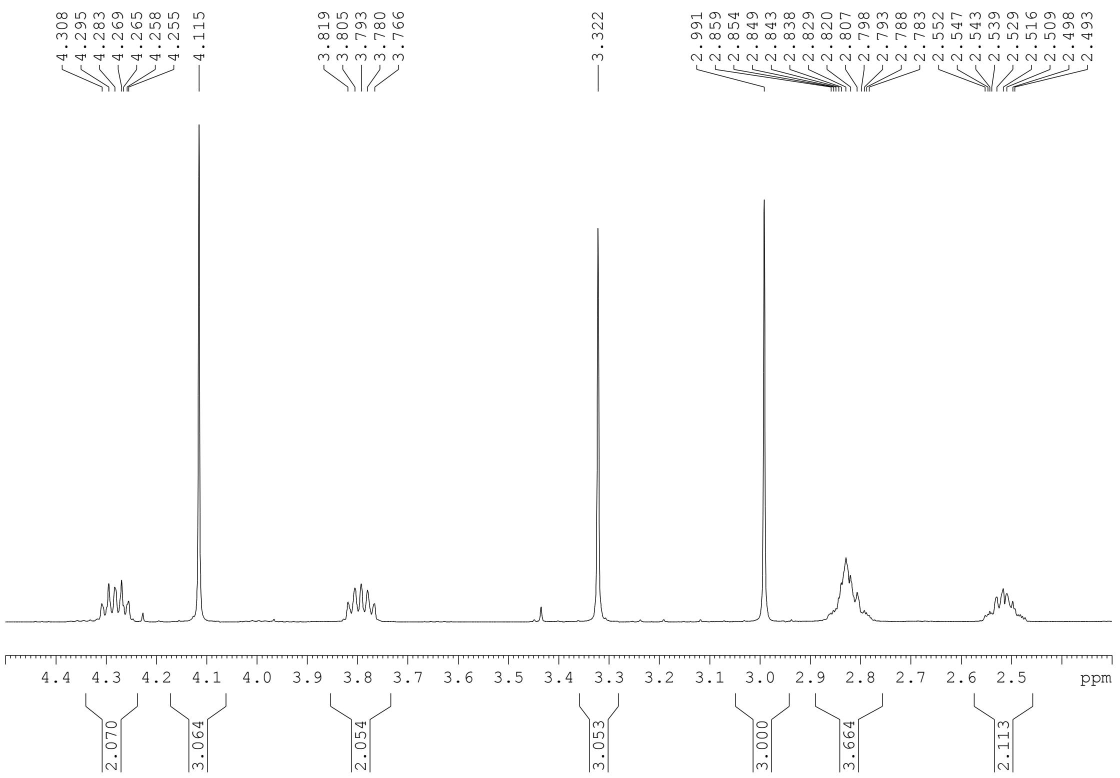

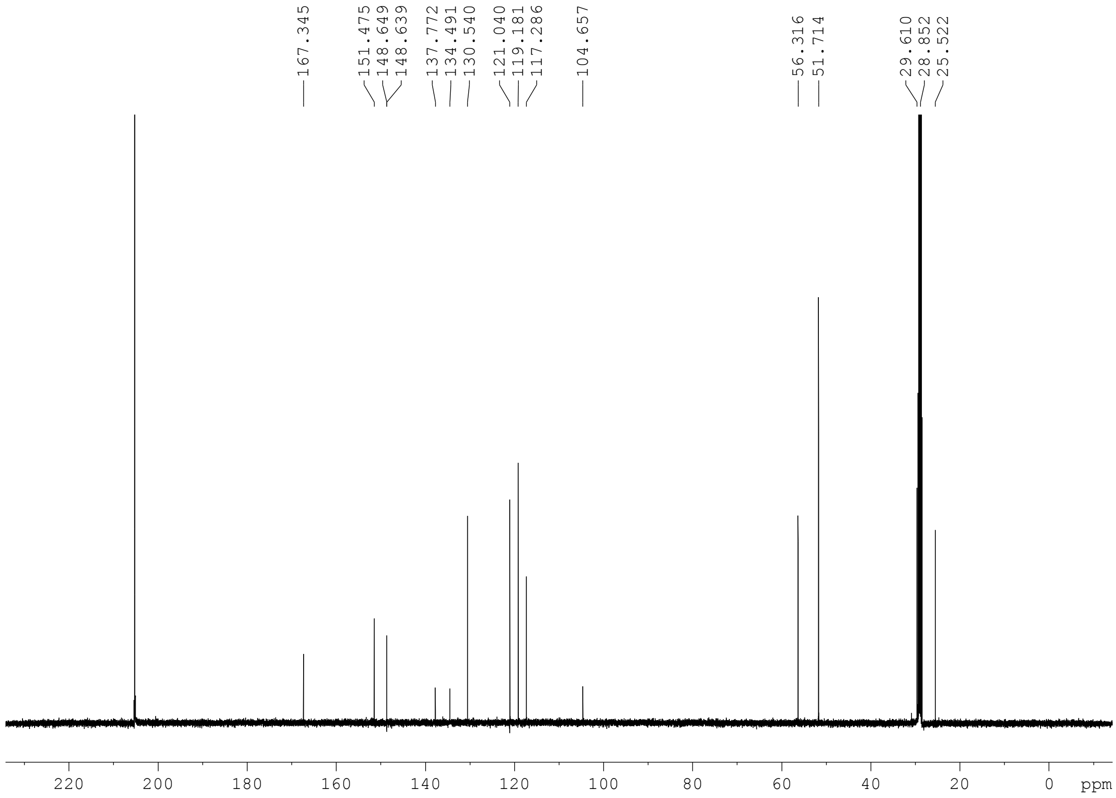

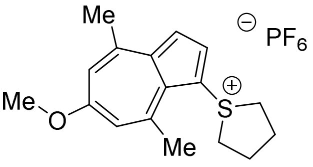

11e (126 MHz, acetone-d_6)

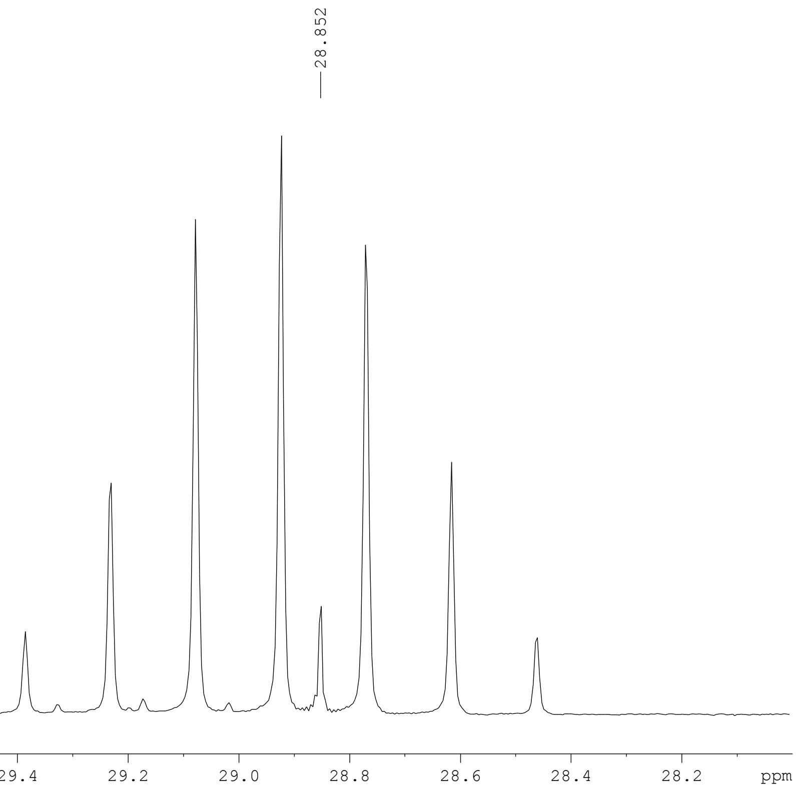

COSY

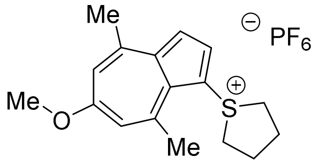

11e (500 MHz, acetone-d_6)

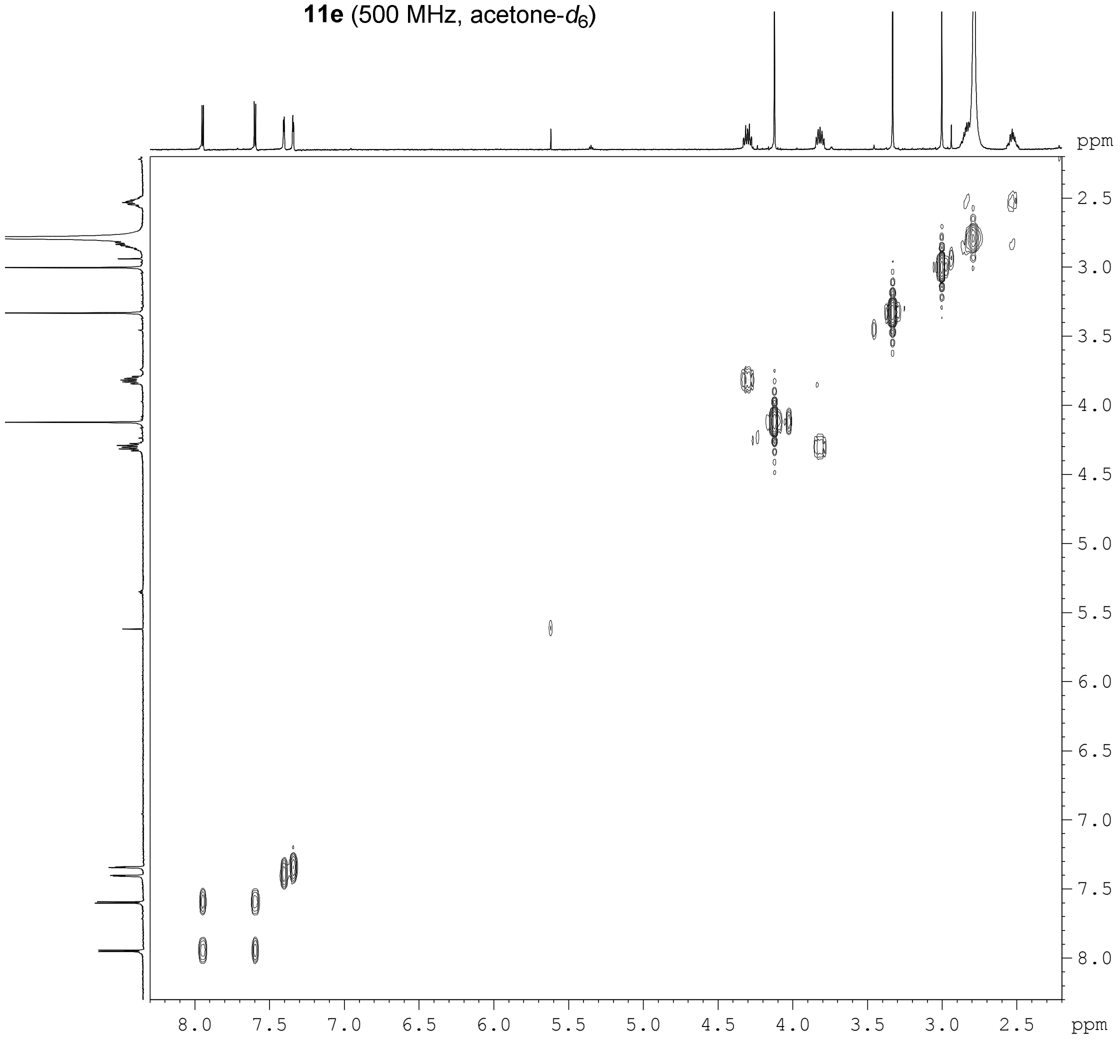

NOESY

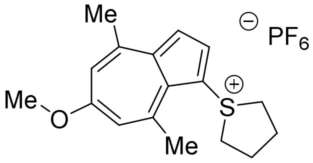

11e (500 MHz, acetone-d_6)

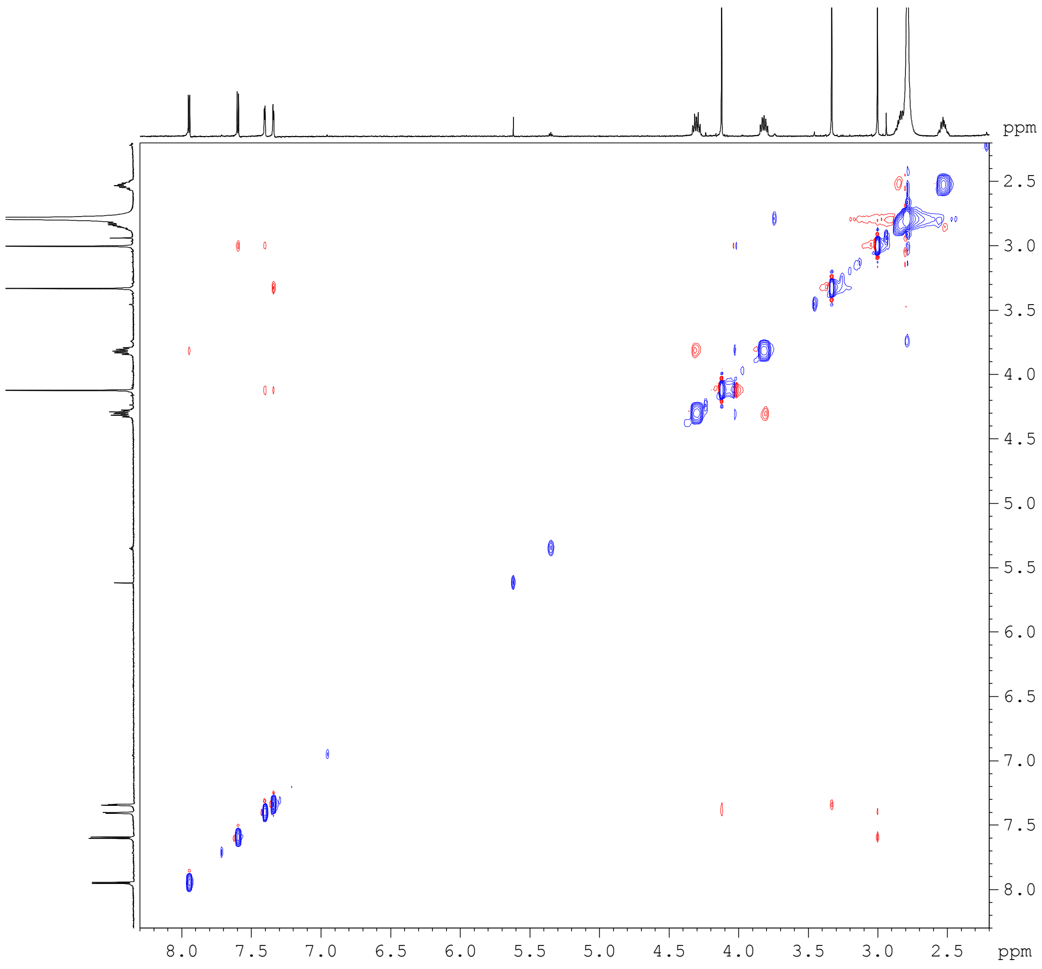

NOESY

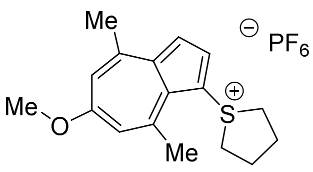

11e (500 MHz, acetone-d_6)

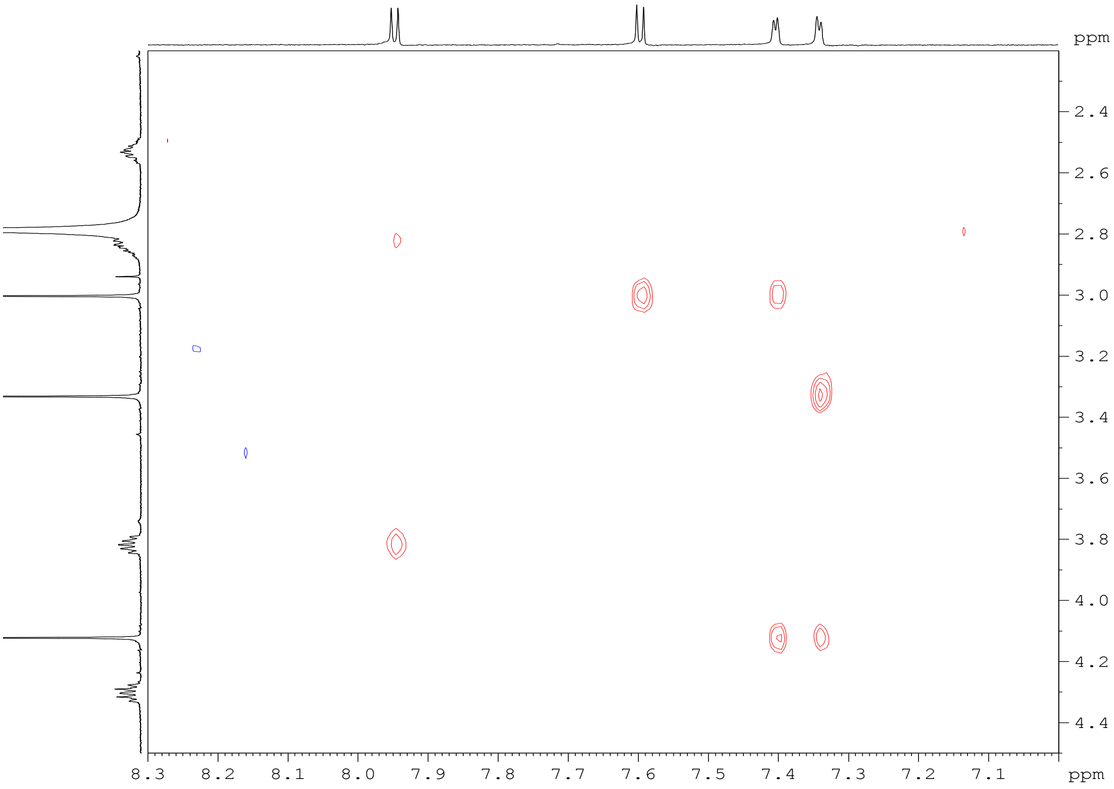

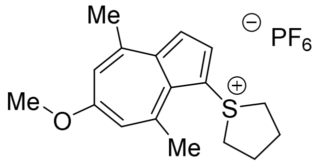

11e (^{19}F, 470.5 MHz, acetone-d_6)

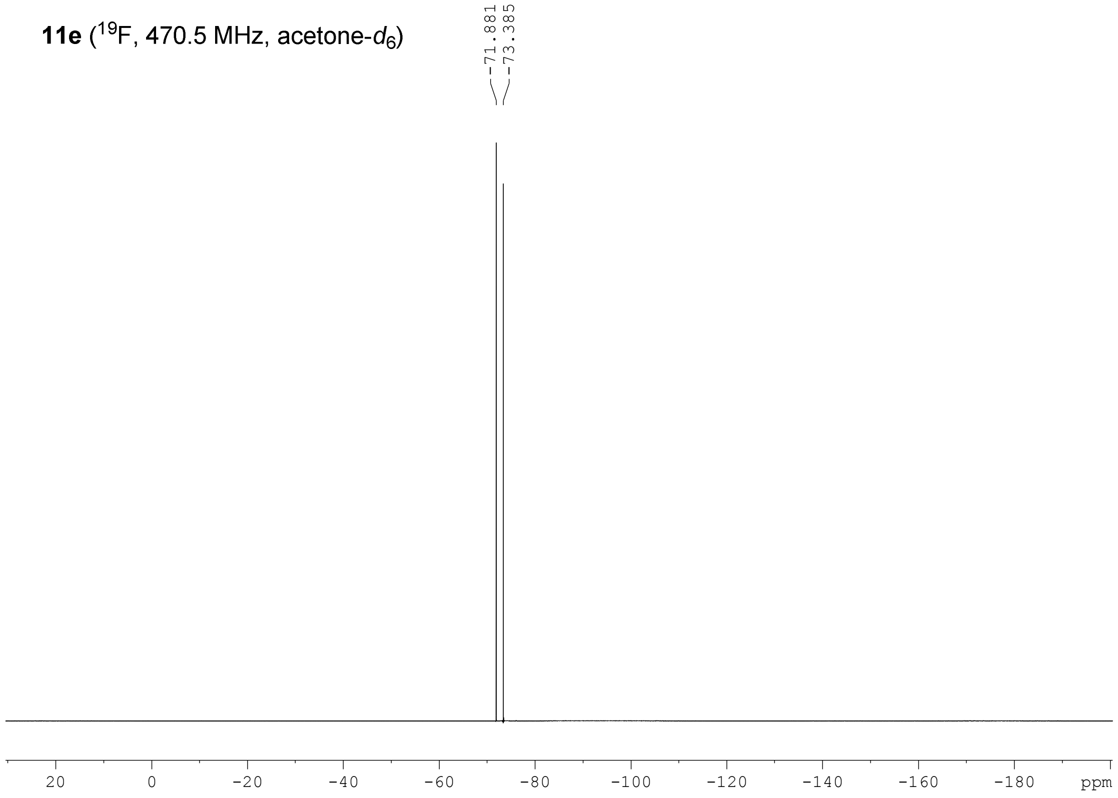

HSQC

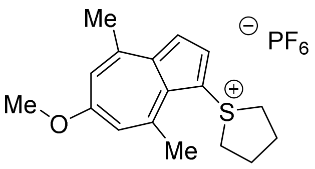

11e (500 MHz, acetone-d_6)

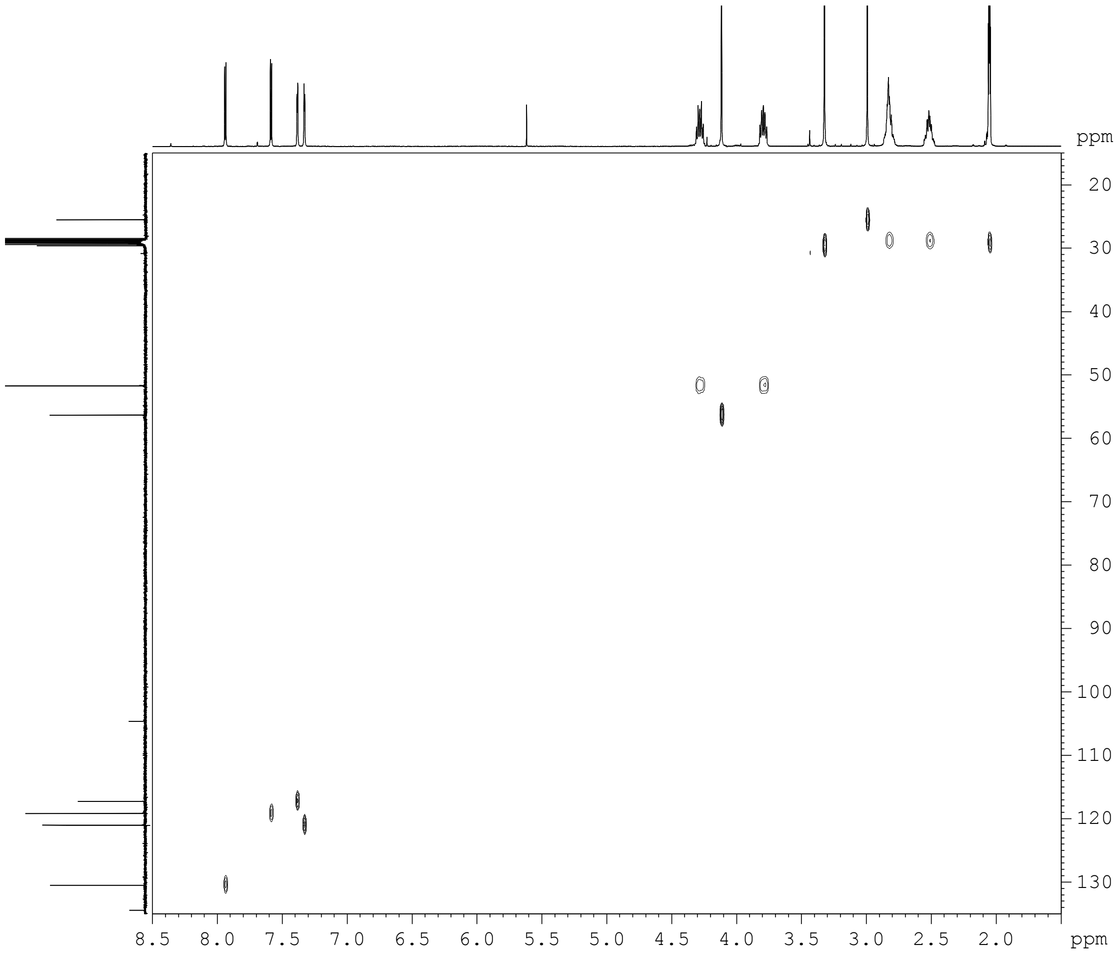

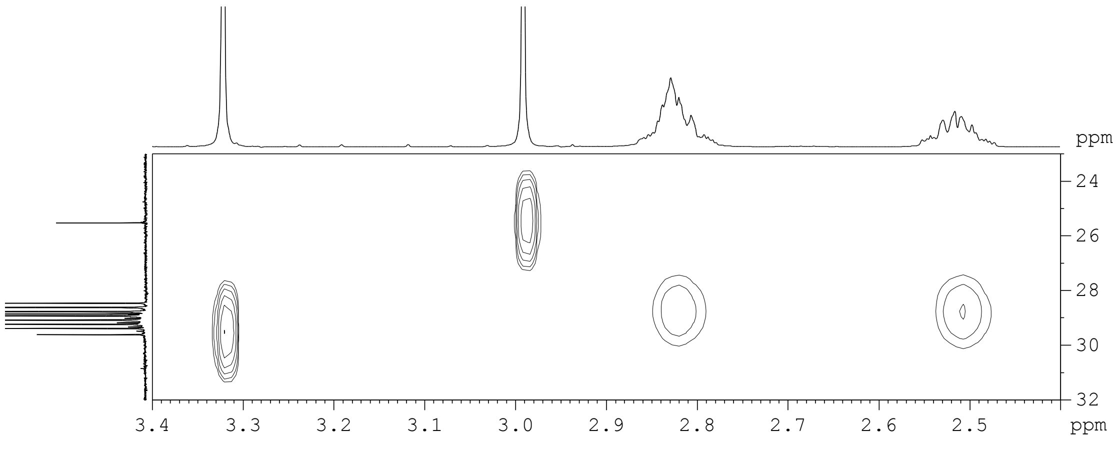

HMBC

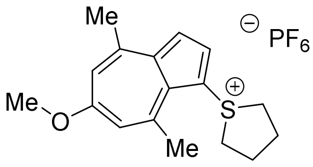

11e (500 MHz, acetone-d_6)

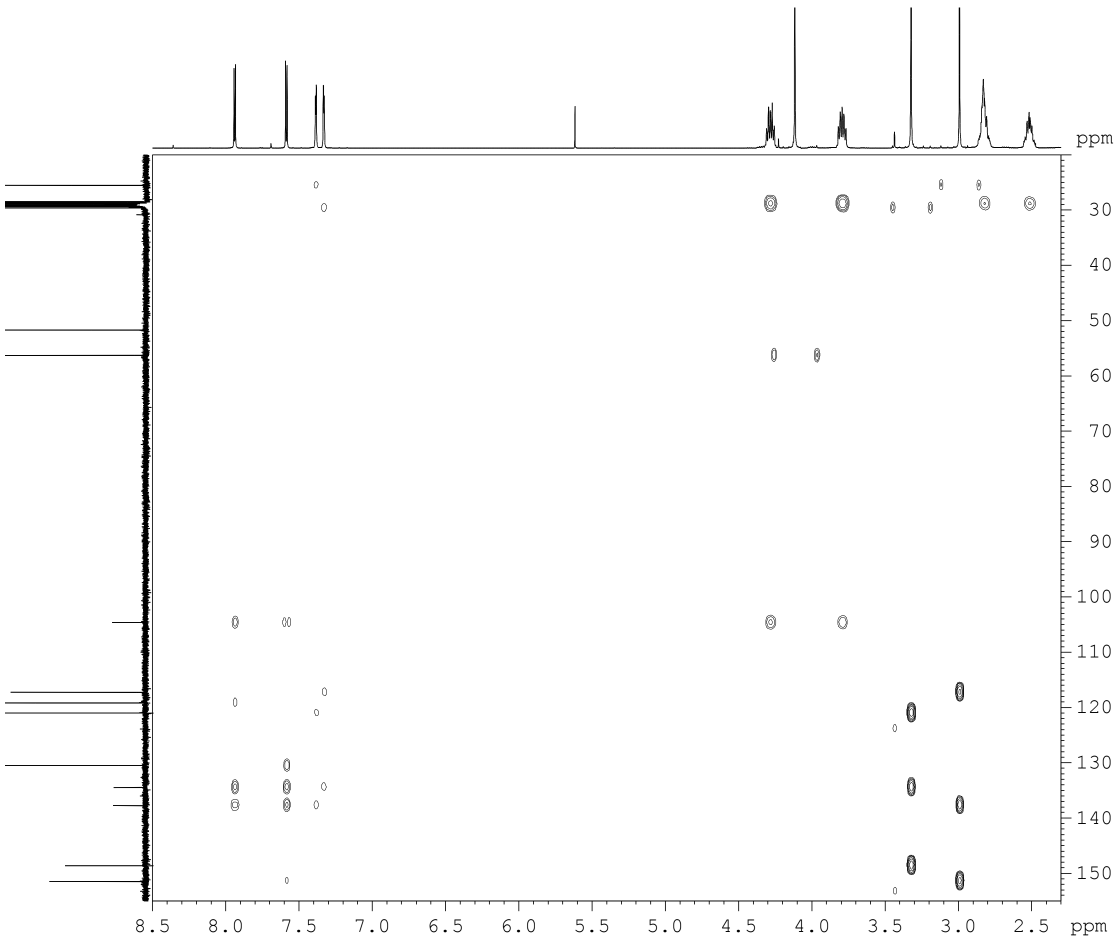

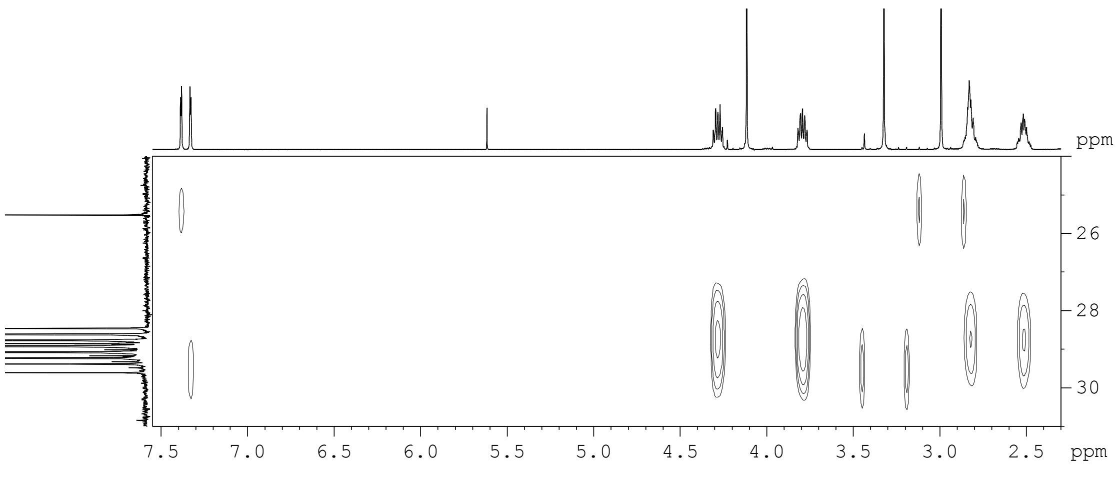

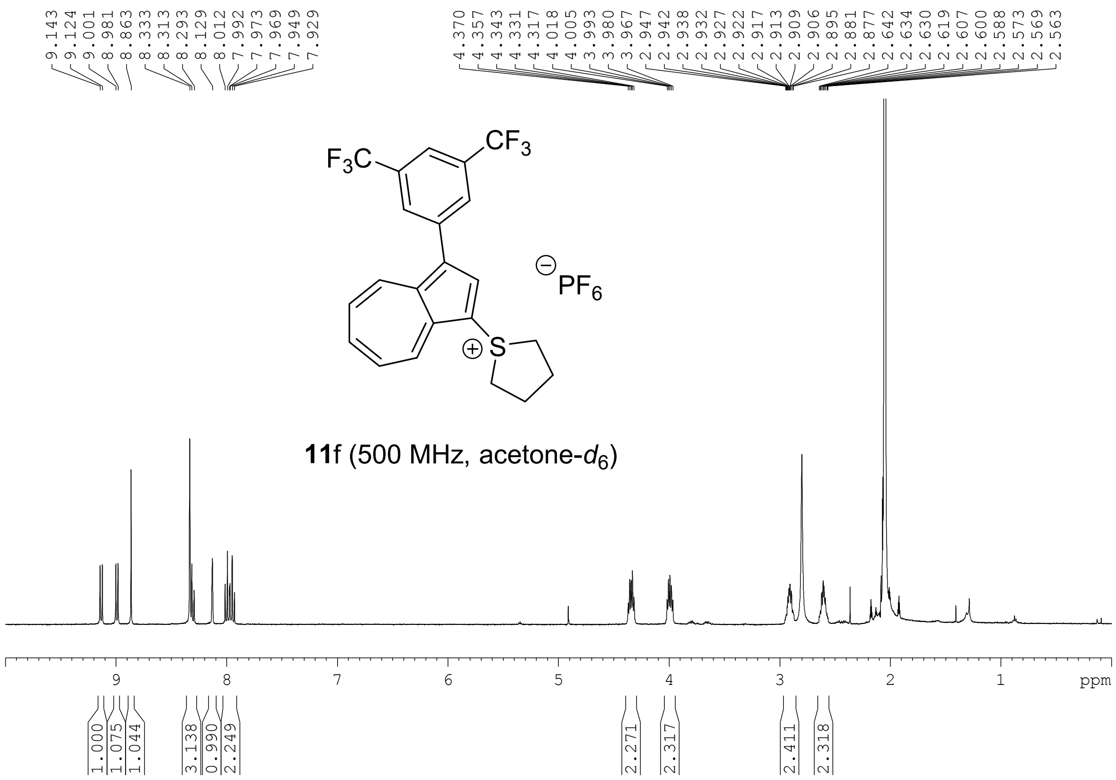
9.143 9.124 9.001 8.981 8.863 8.333 8.313 8.293 8.129 8.012 7.992 7.973 7.969 7.949 7.929
4.370 4.357 4.343 4.331 4.317 4.018 4.005 3.993 3.980 3.967 2.947 2.942 2.938 2.932 2.927 2.922 2.917 2.913 2.909 2.906 2.895 2.881 2.877 2.642 2.634 2.630 2.619 2.607 2.600 2.588 2.573 2.569 2.563
F3C
CF3
PF6
S
11f (500 MHz, acetone-d6)
9 8 7 6 5 4 3 2 1 ppm
1.000 1.075 1.044 3.138 0.990 2.249 2.271 2.317 2.411 2.318

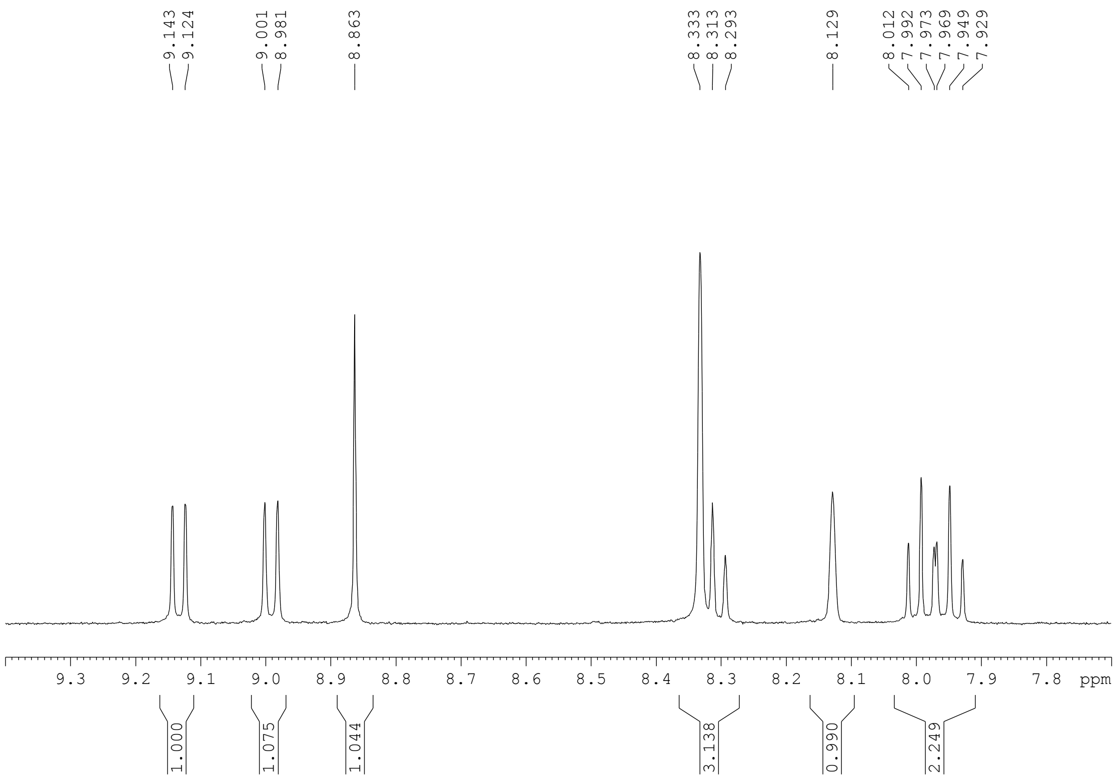
9.143 9.124 9.001 8.981 8.863 8.333 8.313 8.293 8.129 8.012 7.992 7.973 7.969 7.949 7.929
9.3 9.2 9.1 9.0 8.9 8.8 8.7 8.6 8.5 8.4 8.3 8.2 8.1 8.0 7.9 7.8 ppm
1.000 1.075 1.044 3.138 0.990 2.249

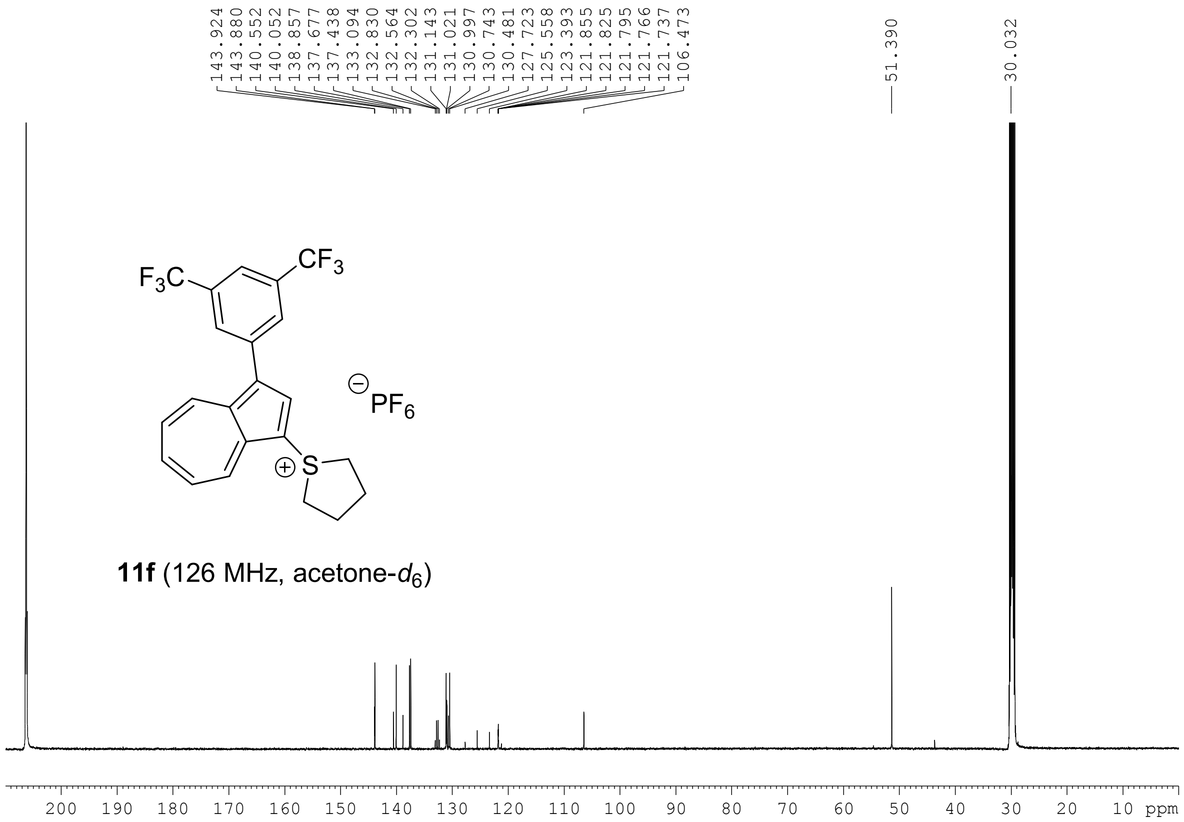
143.924
143.880
140.552
140.052
138.857
137.677
137.438
133.094
132.830
132.564
132.302
131.143
131.021
130.997
130.743
130.481
127.723
125.558
123.393
121.855
121.825
121.795
121.766
121.737
106.473
51.390
30.032
F_3C
CF_3
PF_6
S
11f (126 MHz, acetone-d_6)
200 190 180 170 160 150 140 130 120 110 100 90 80 70 60 50 40 30 20 10 ppm

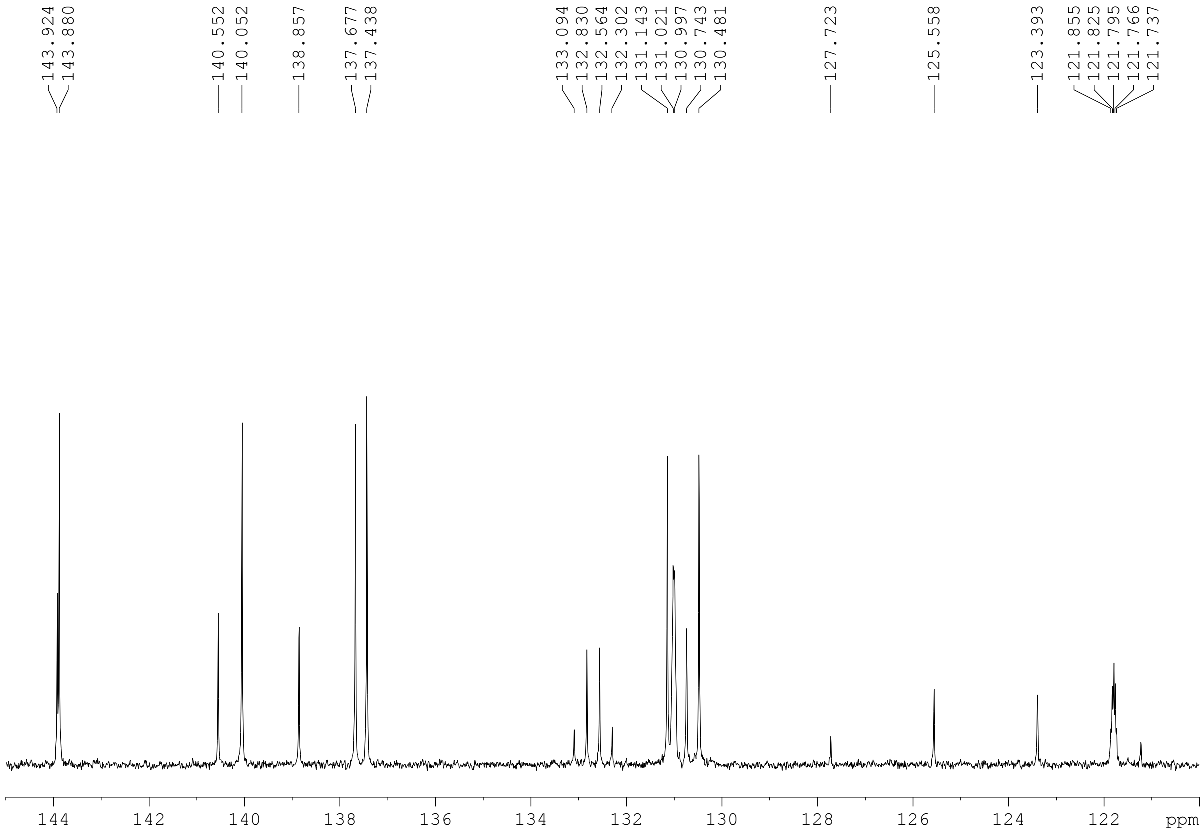
143.924
143.880
140.552
140.052
138.857
137.677
137.438
133.094
132.830
132.564
132.302
131.143
131.021
130.997
130.743
130.481
127.723
125.558
123.393
121.855
121.825
121.795
121.766
121.737
144 142 140 138 136 134 132 130 128 126 124 122 ppm

COSY

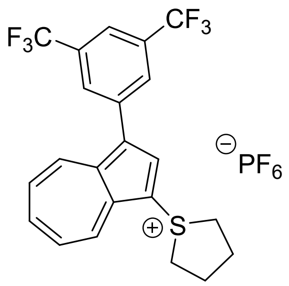

11f (500 MHz, acetone-d_6)

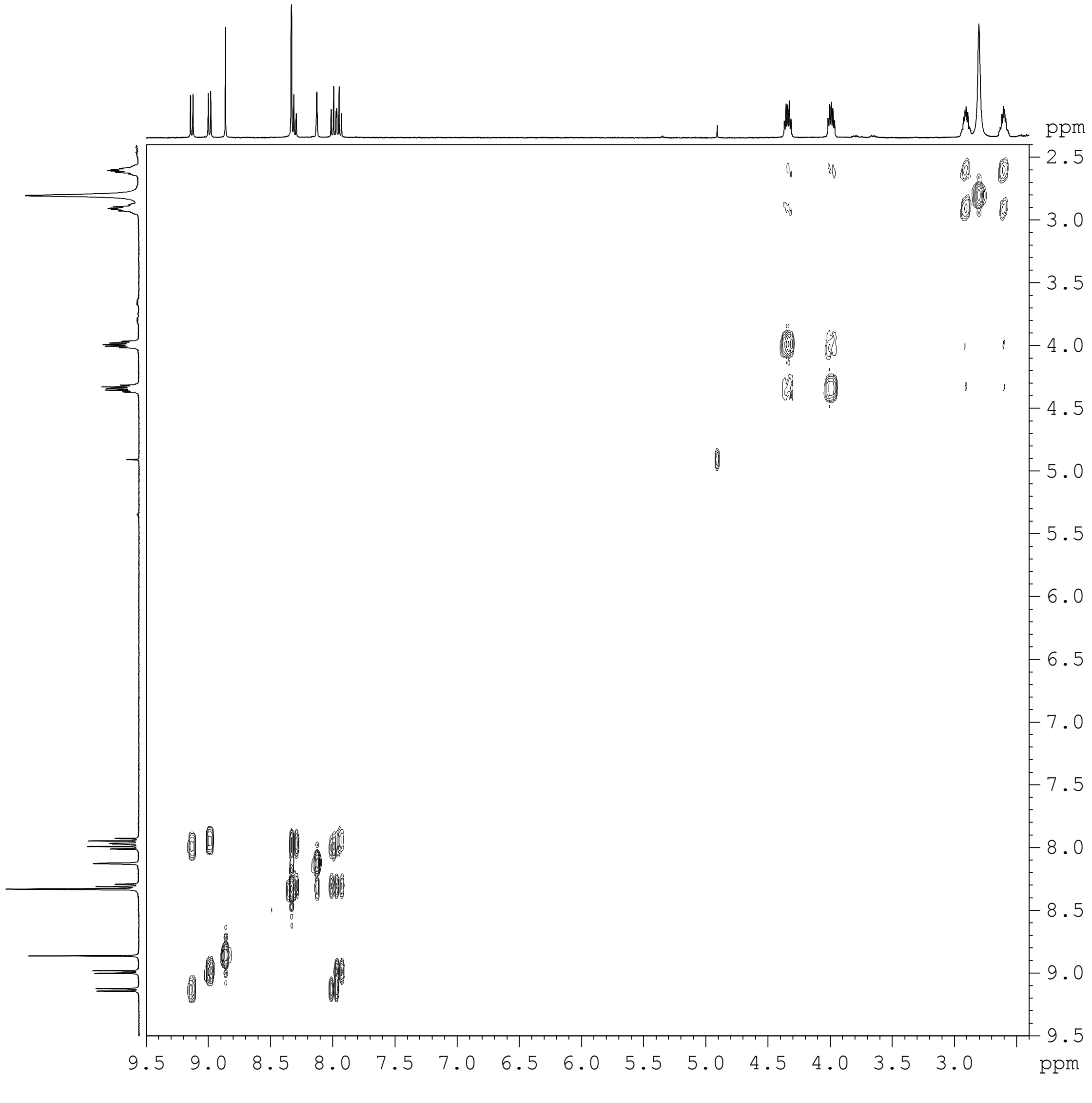

NOESY

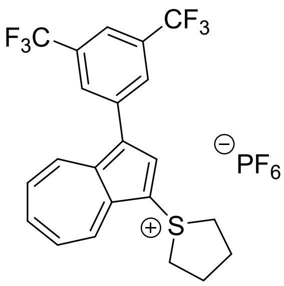

11f (500 MHz, acetone-d_6)

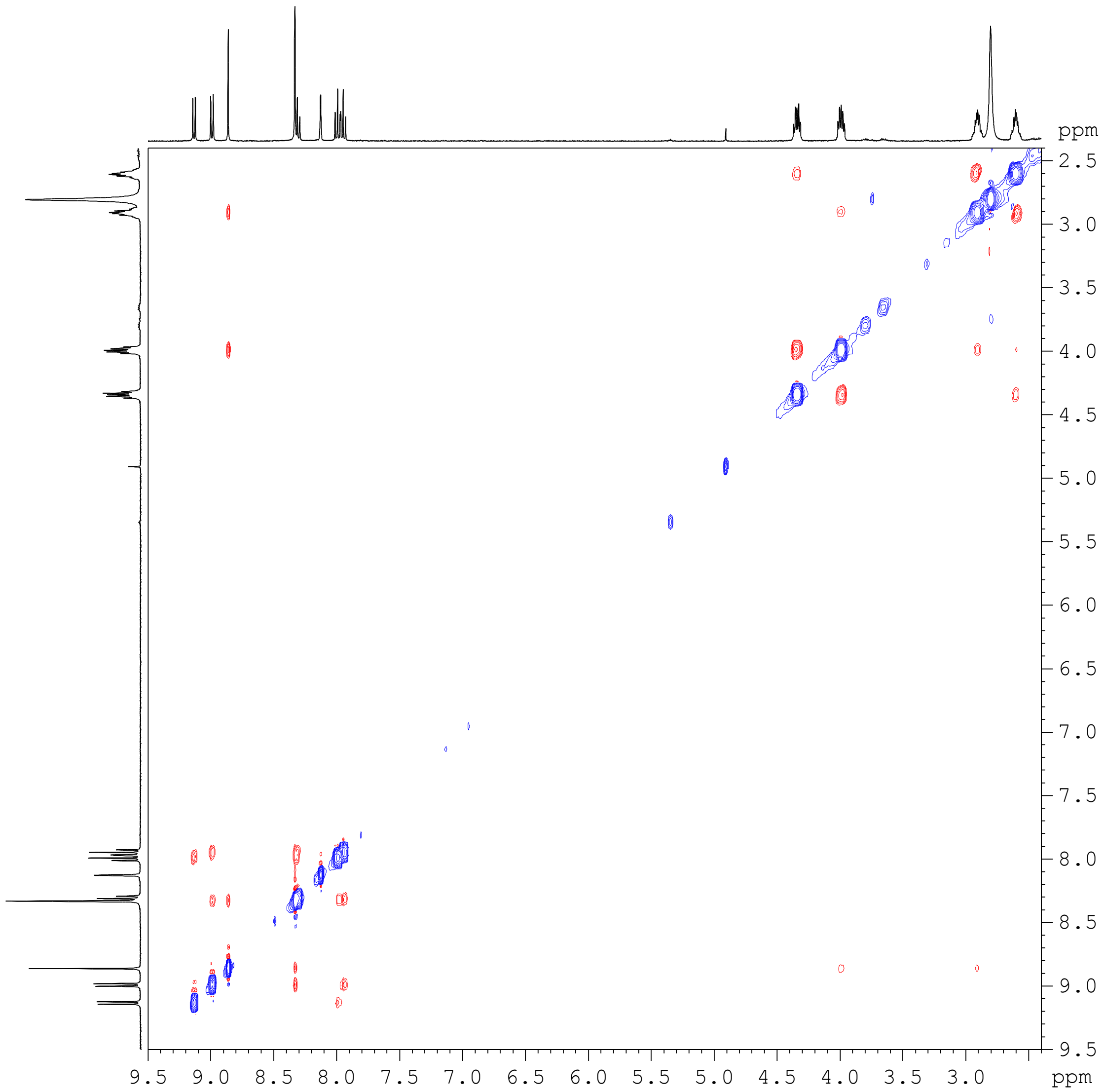

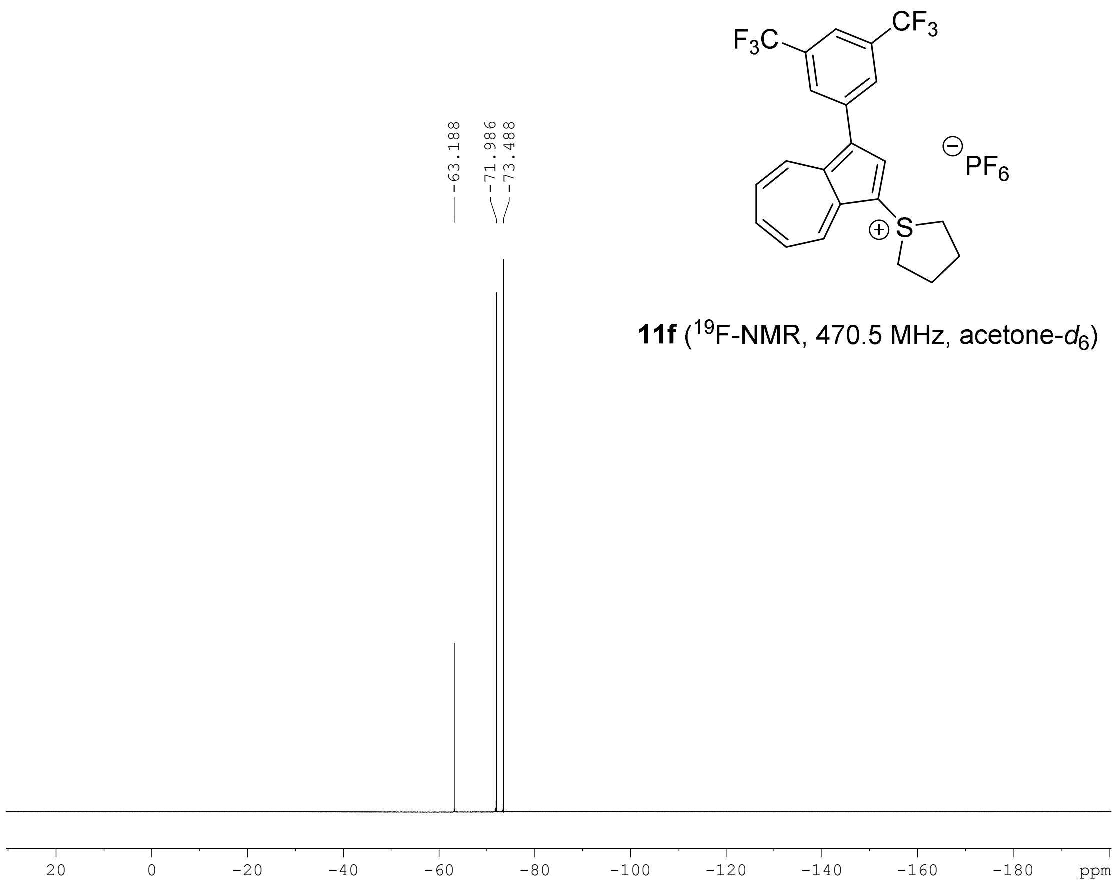

11f (^{19}F-NMR, 470.5 MHz, acetone-d_6)

HSQC

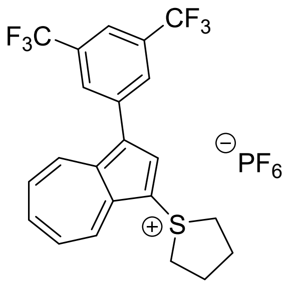

11f (500 MHz, acetone-d_6)

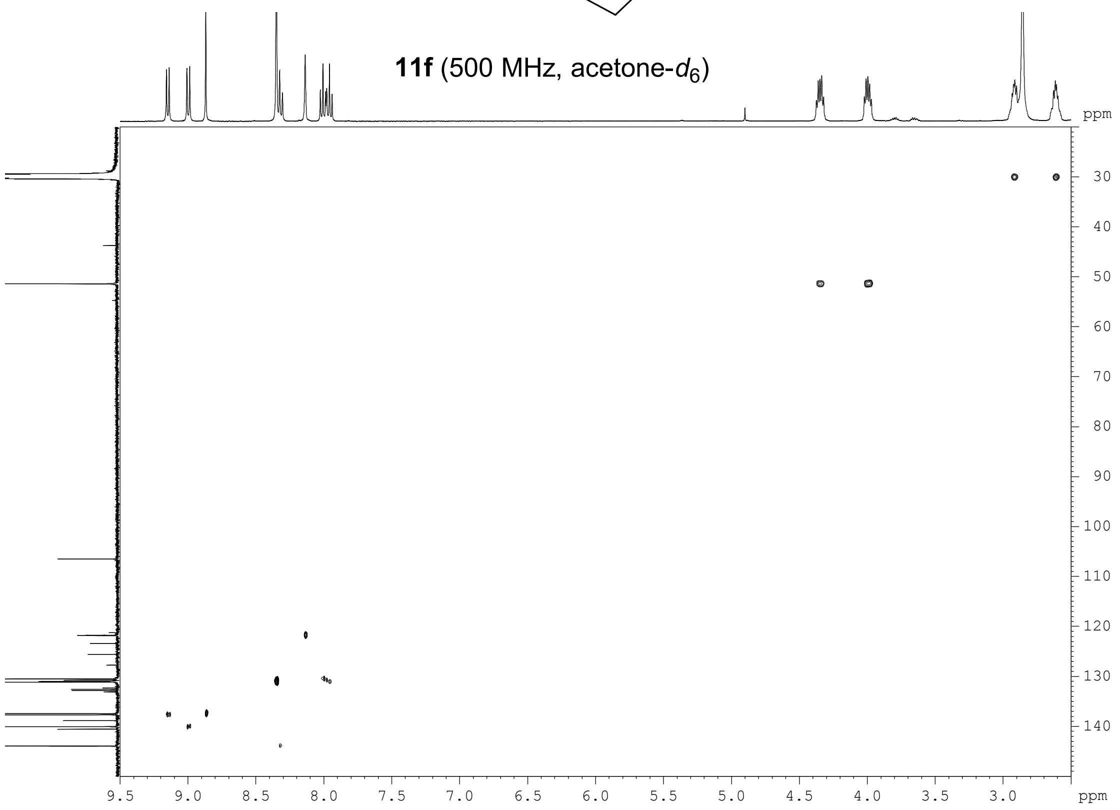

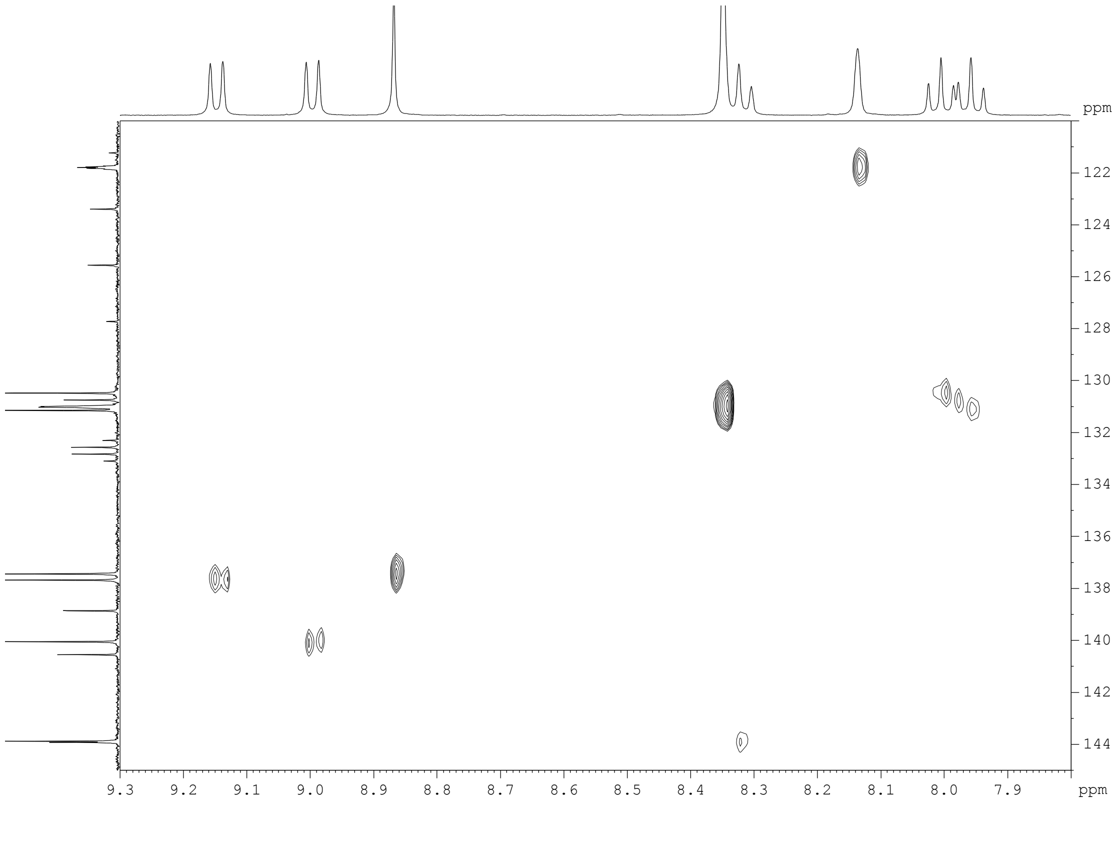

HMBC

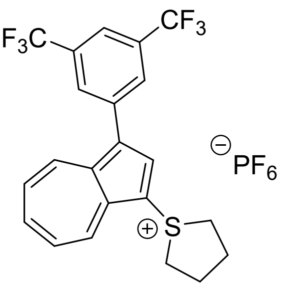

11f (500 MHz, acetone-d_6)

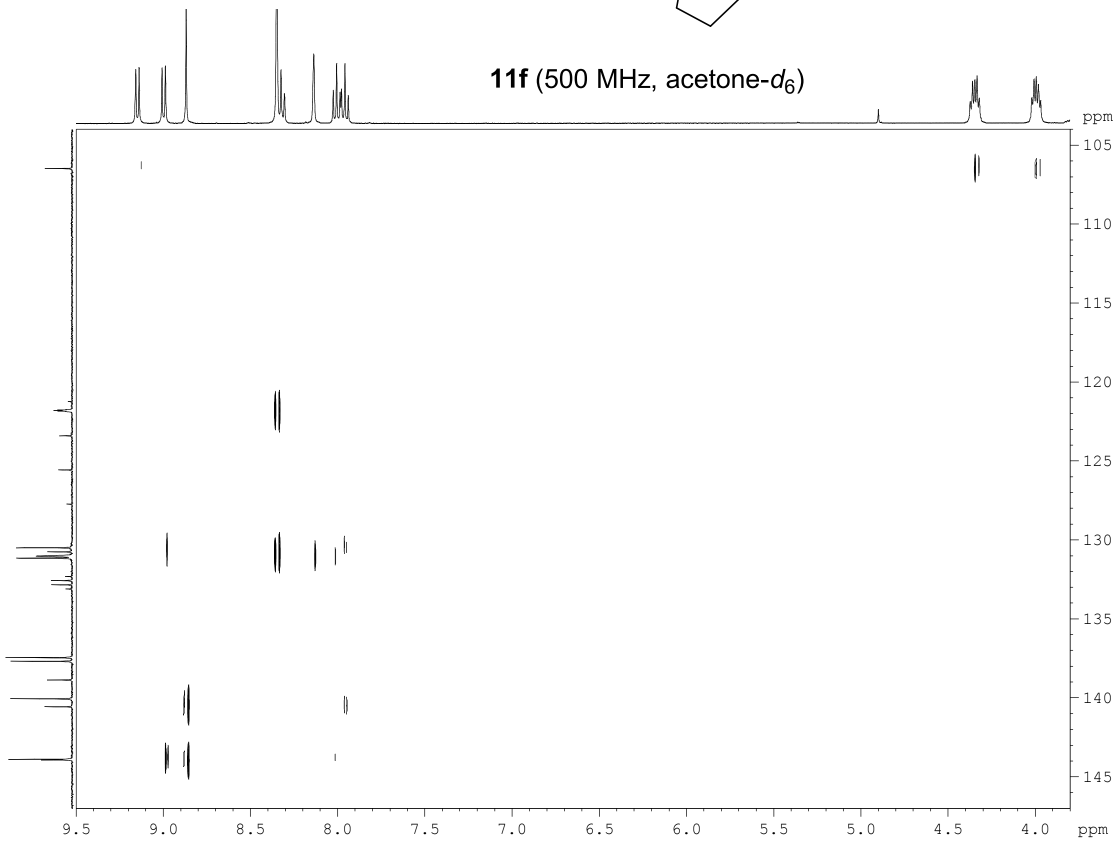

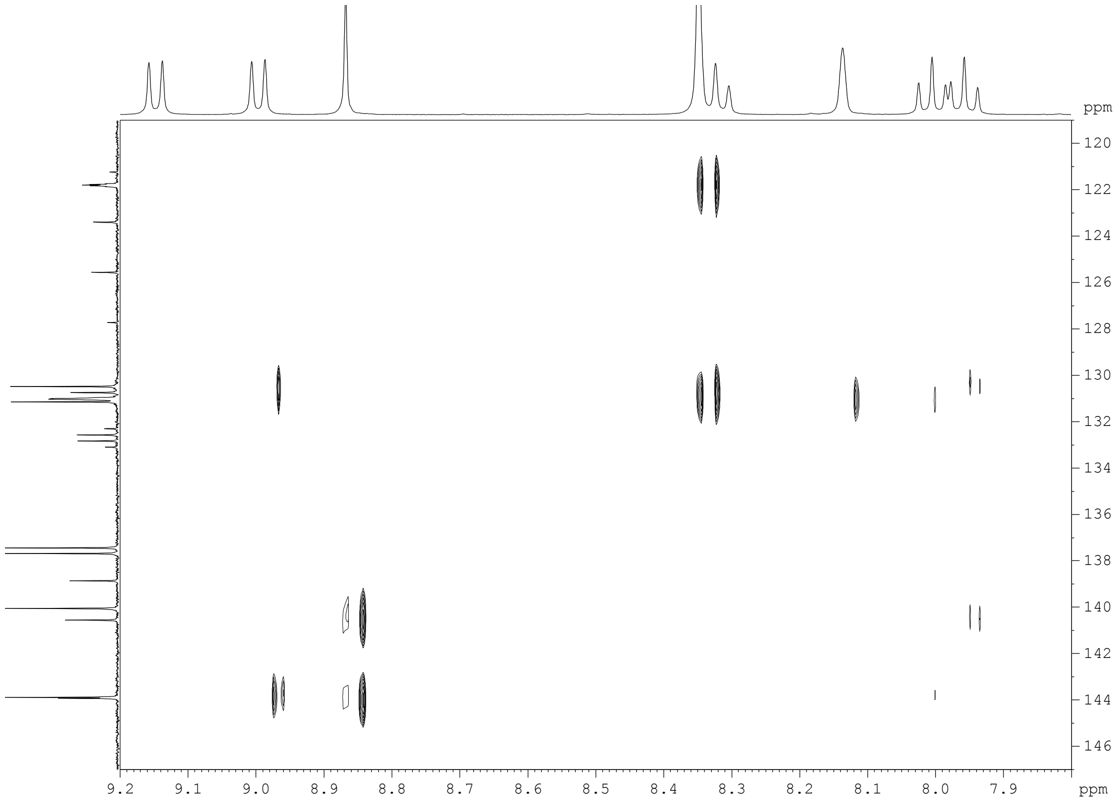

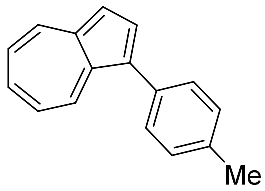

13a (500 MHz, $CDCl_3$)

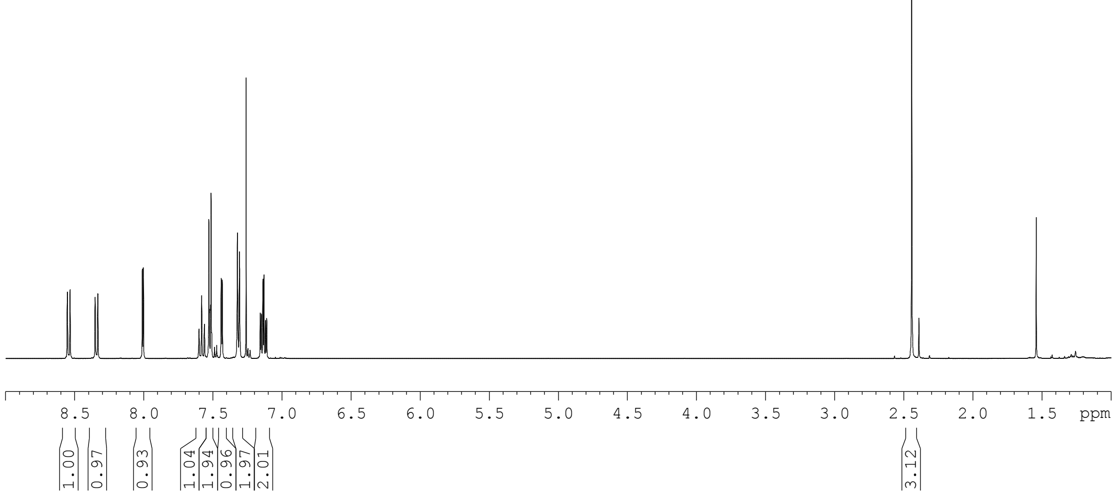

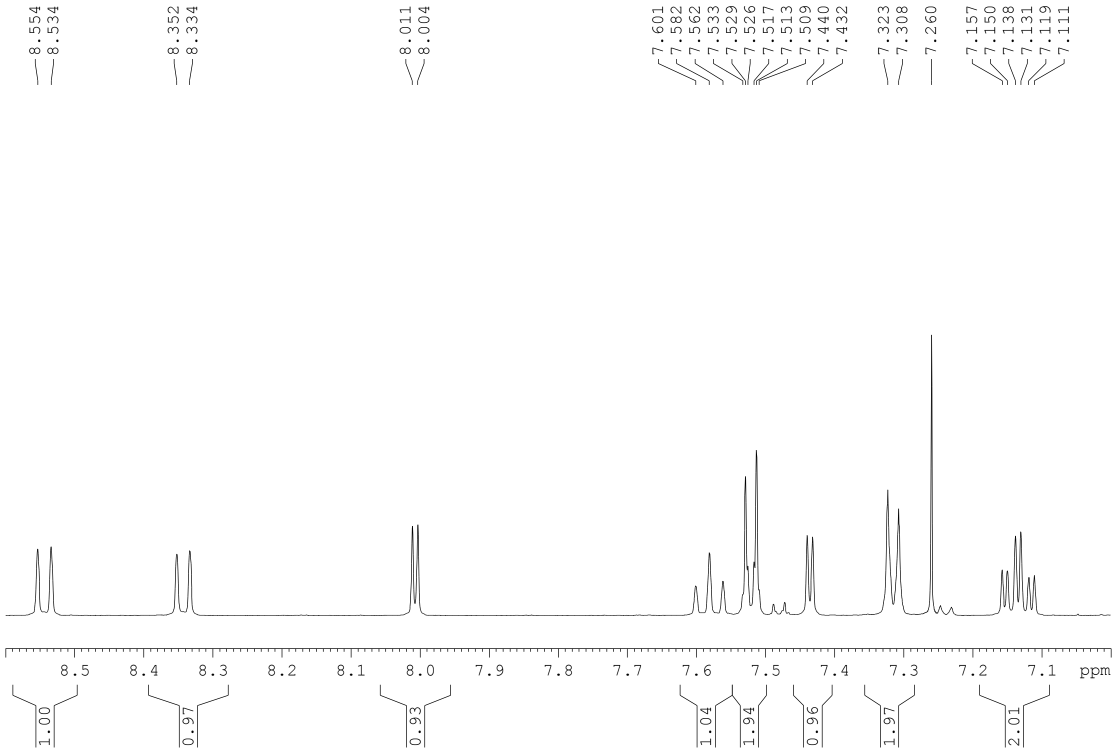

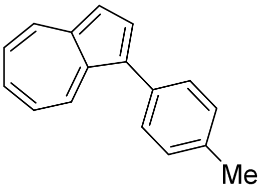

13a (126 MHz, $CDCl_3$)

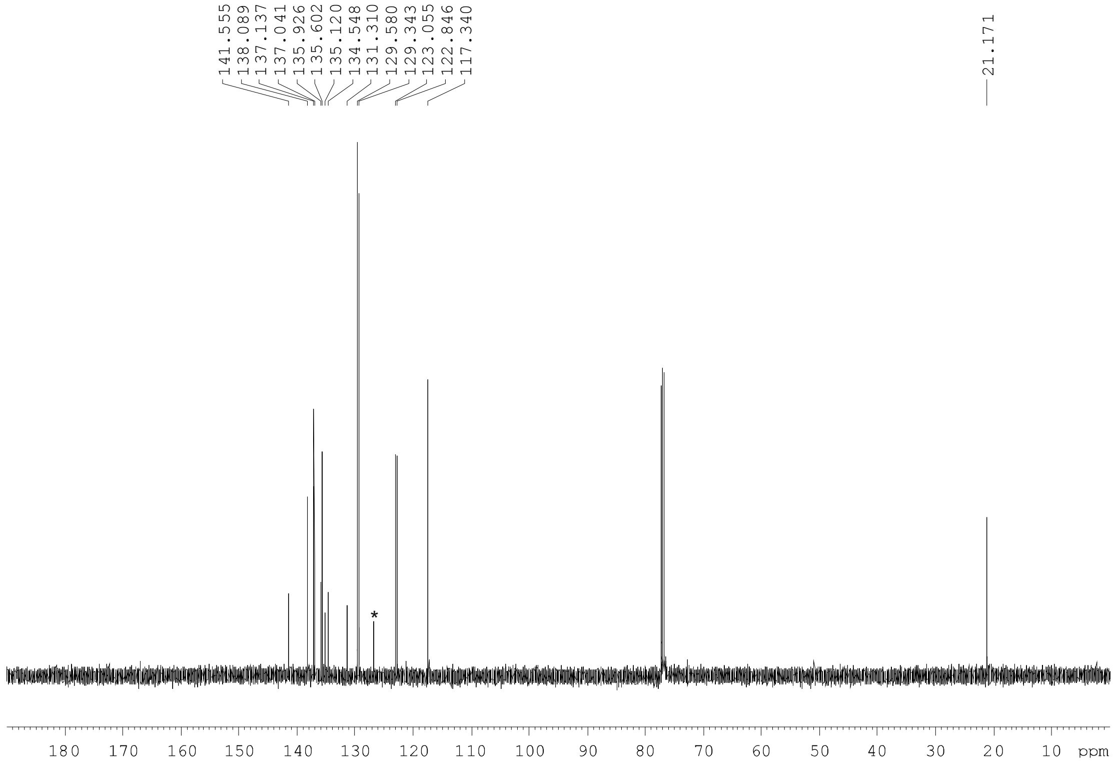

COSY

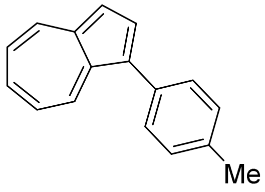

13a (500 MHz, $CDCl_3$)

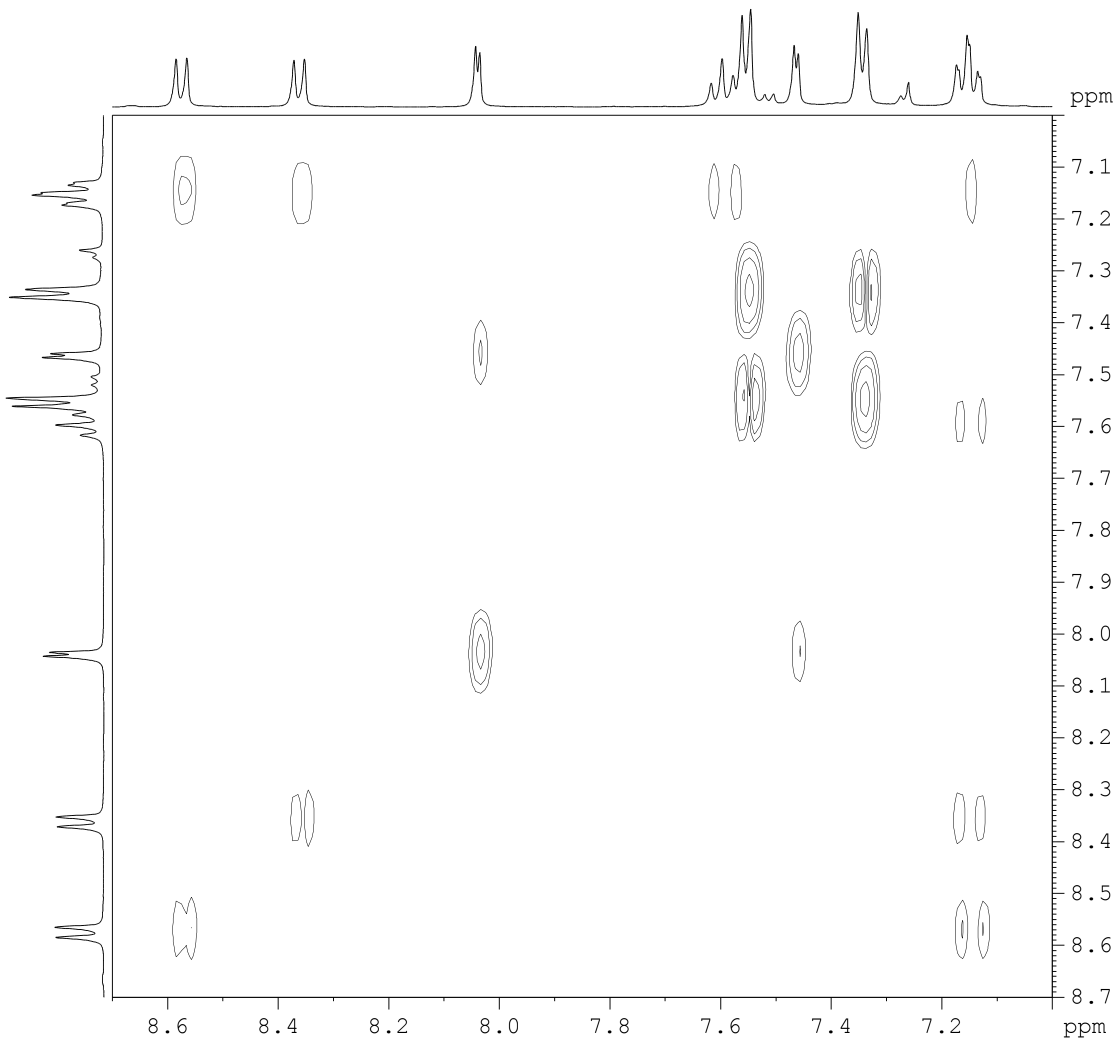

HSQC

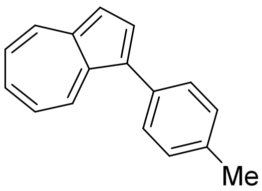

13a (500 MHz, $CDCl_3$)

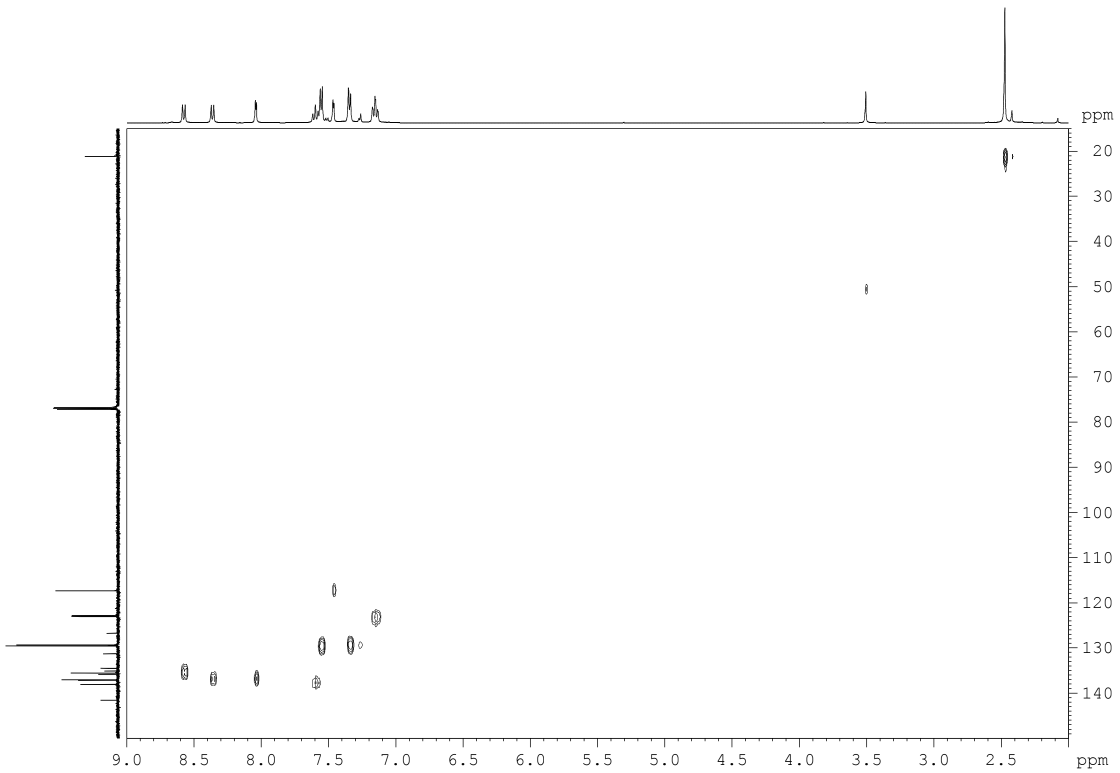

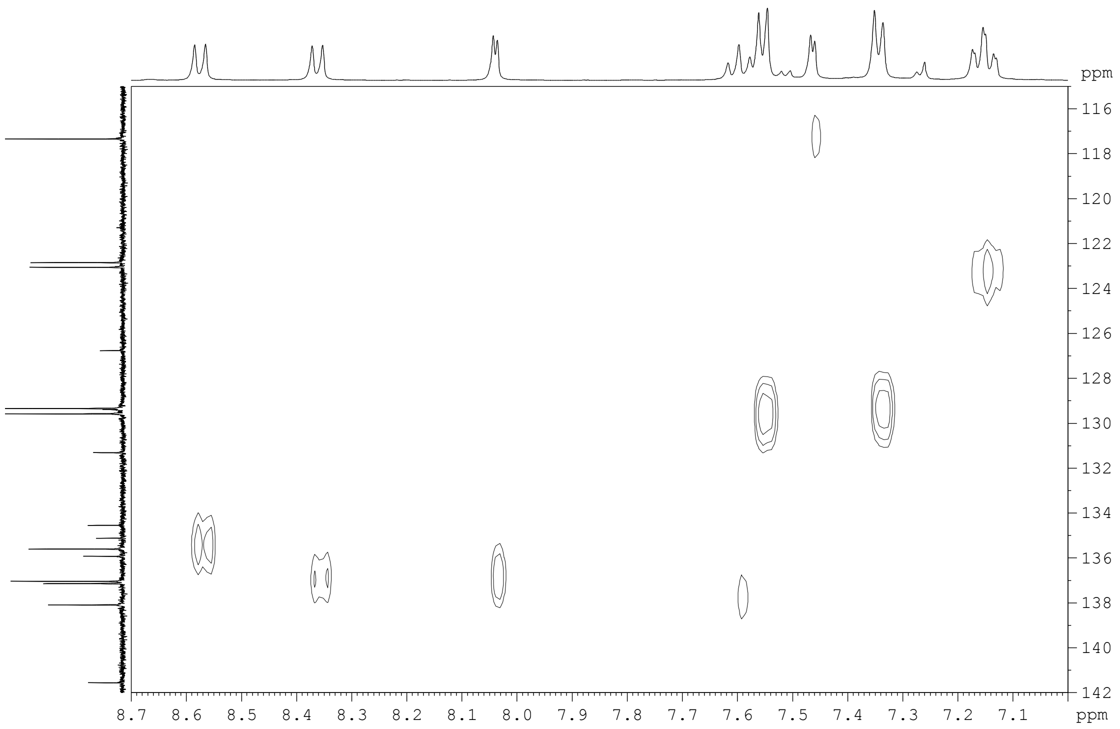

HMBC

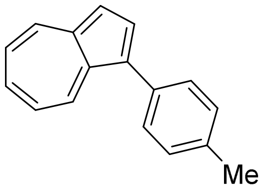

13a (500 MHz, $CDCl_3$)

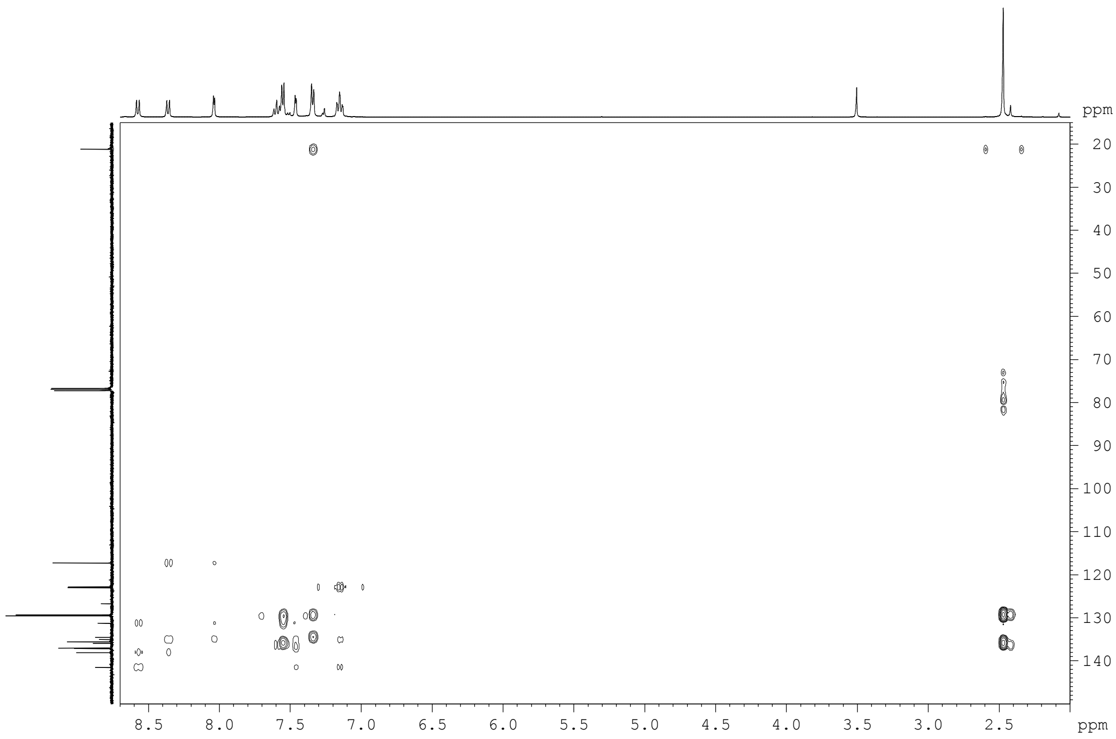

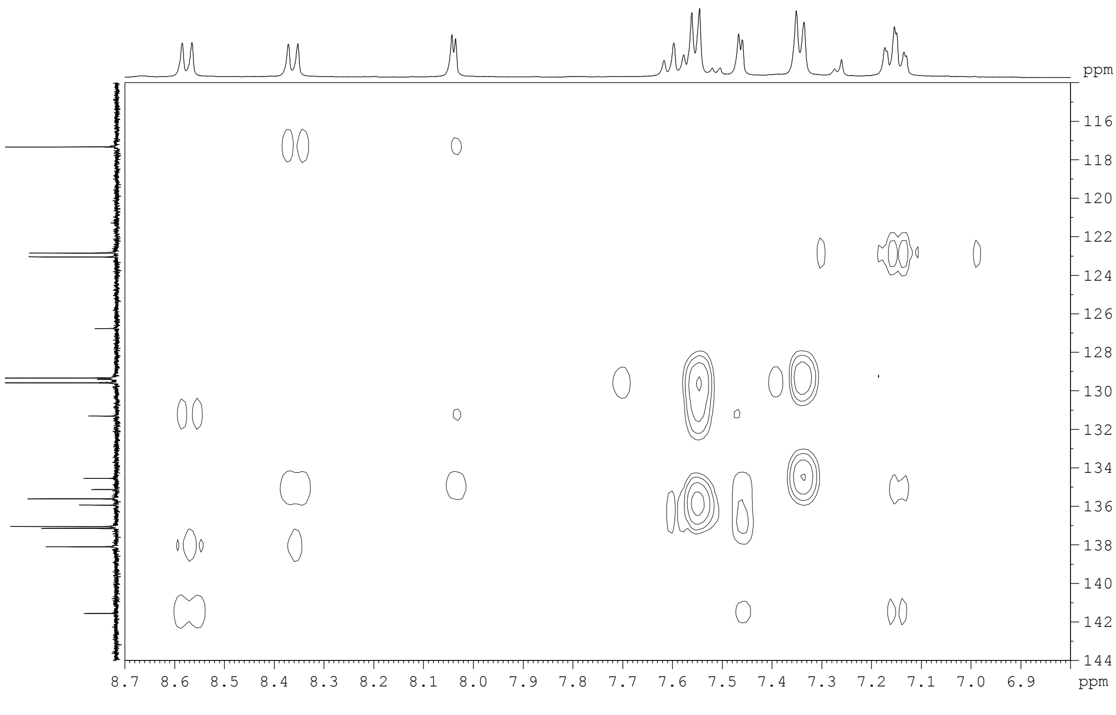

HMBC

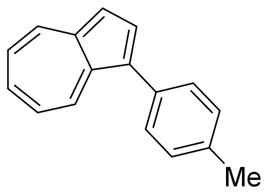

13a (500 MHz, $CDCl_3$)

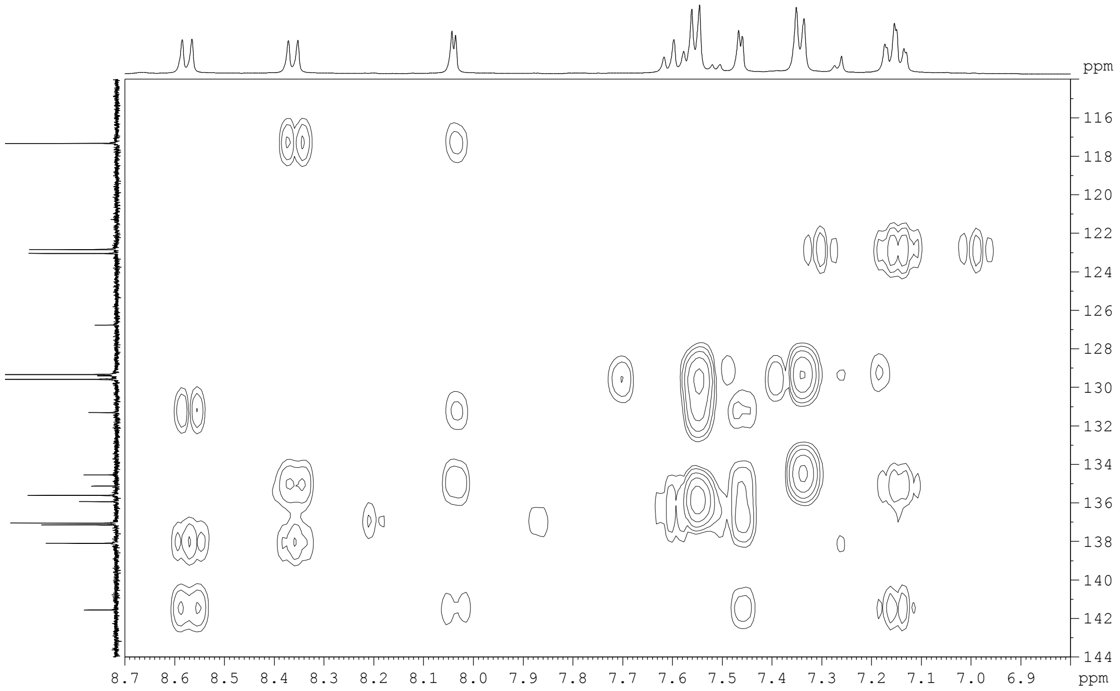

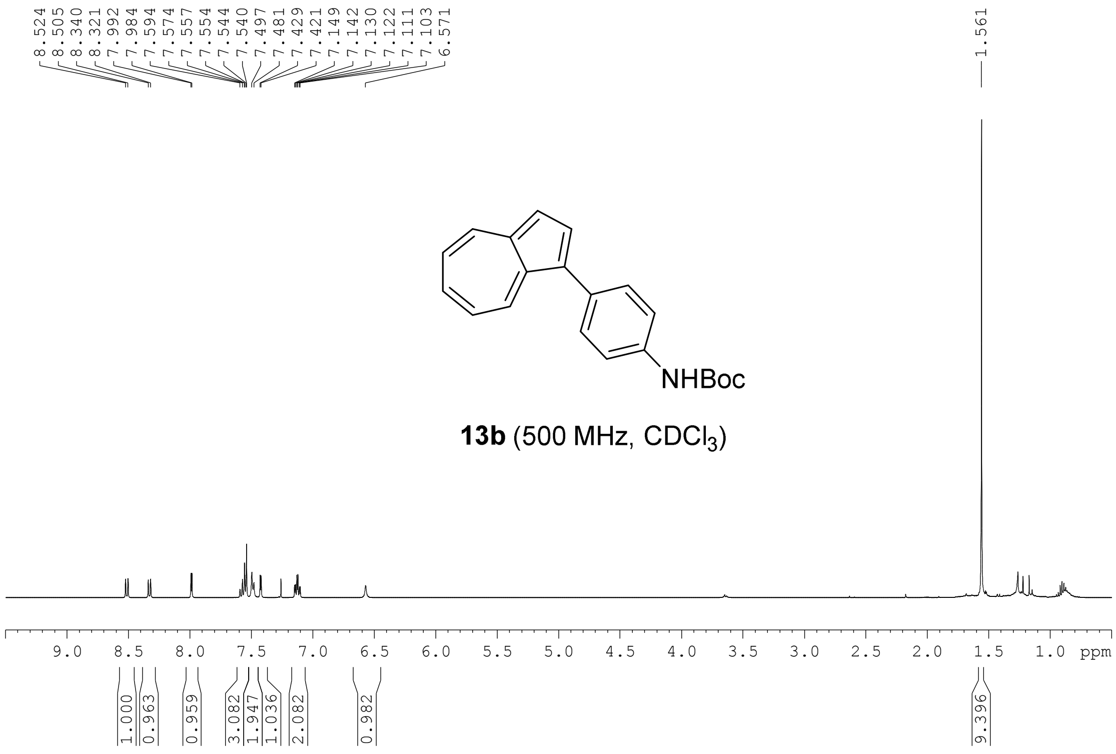

13b (500 MHz, $CDCl_3$)

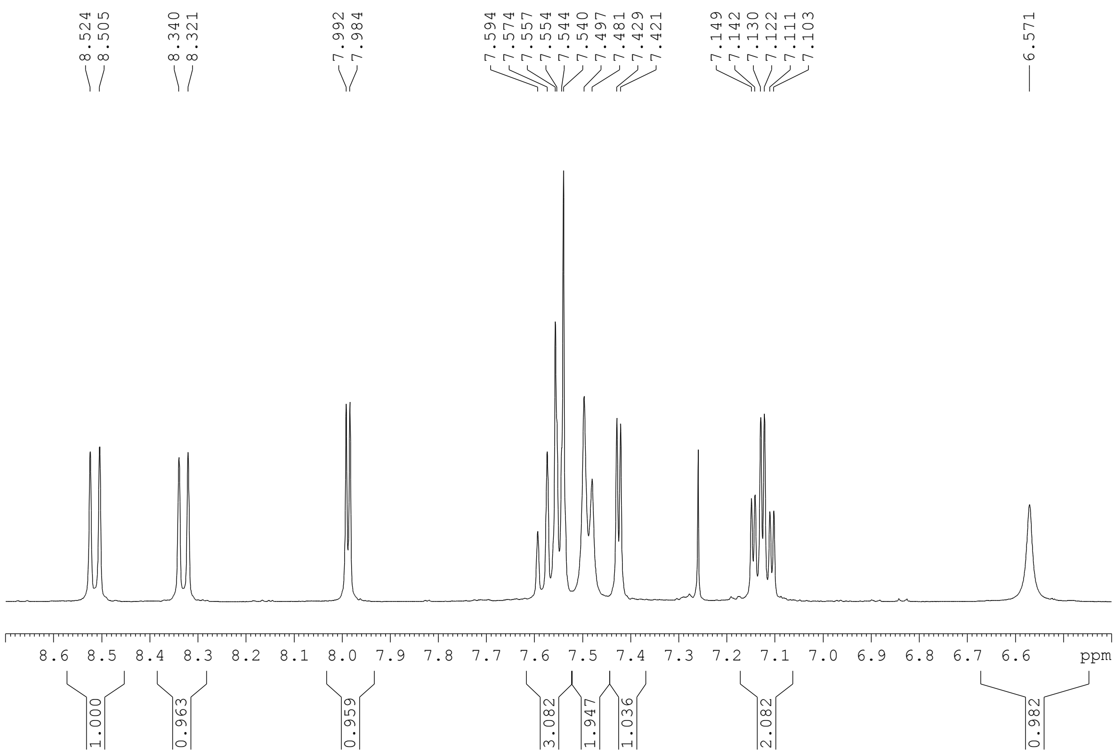

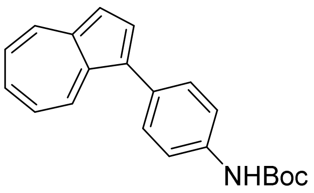

13b (126 MHz, $CDCl_3$)

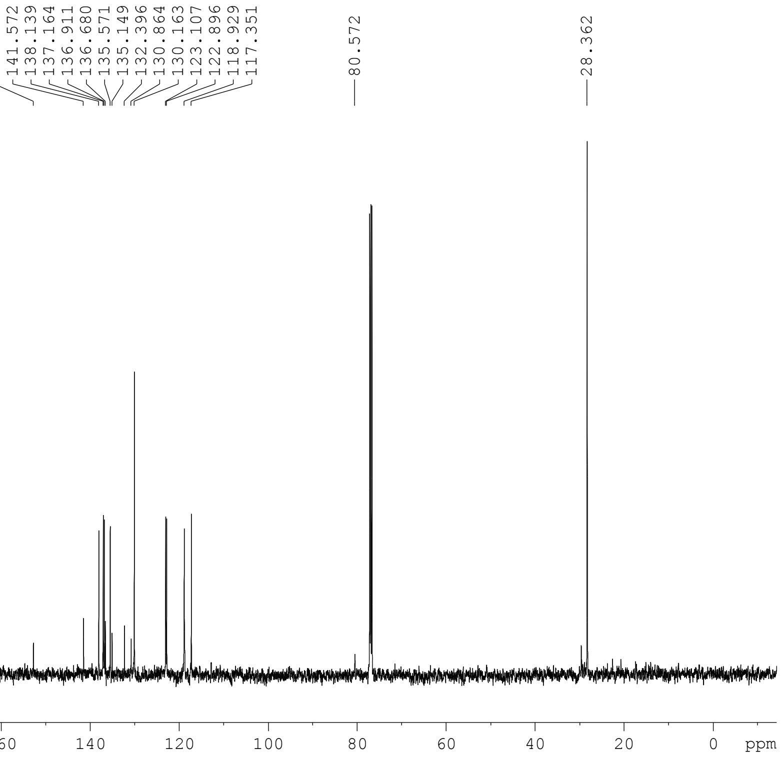

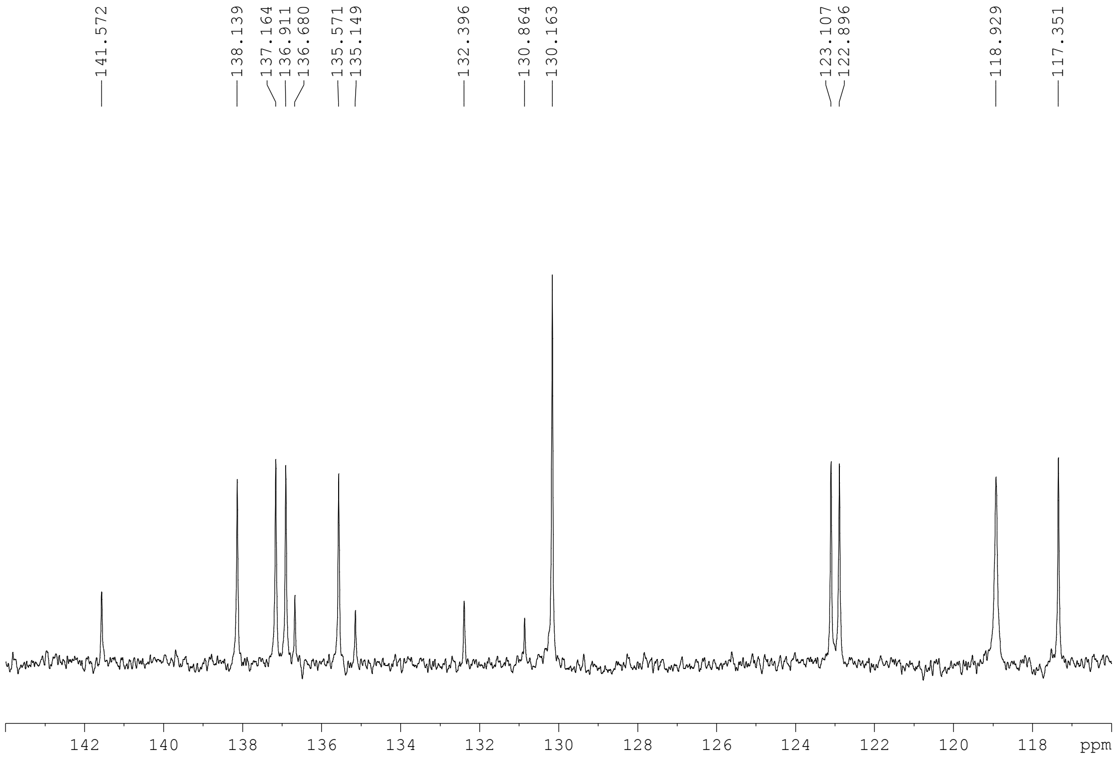

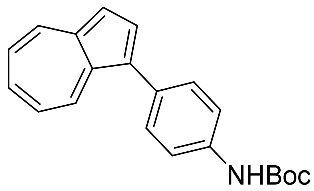

13b (500 MHz, $CDCl_3$)

COSY

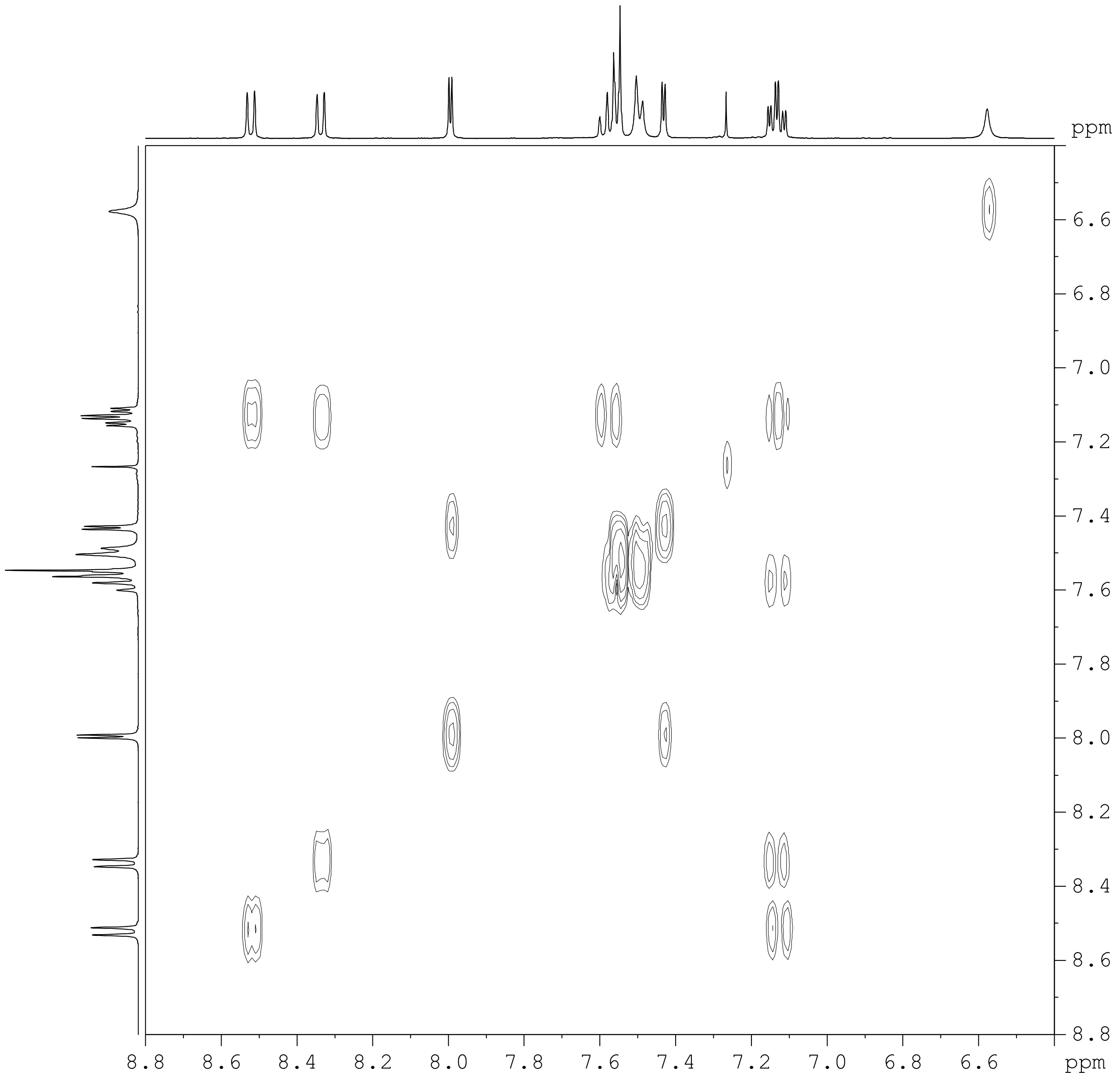

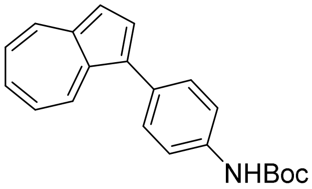

13b (500 MHz, $CDCl_3$)

NOESY

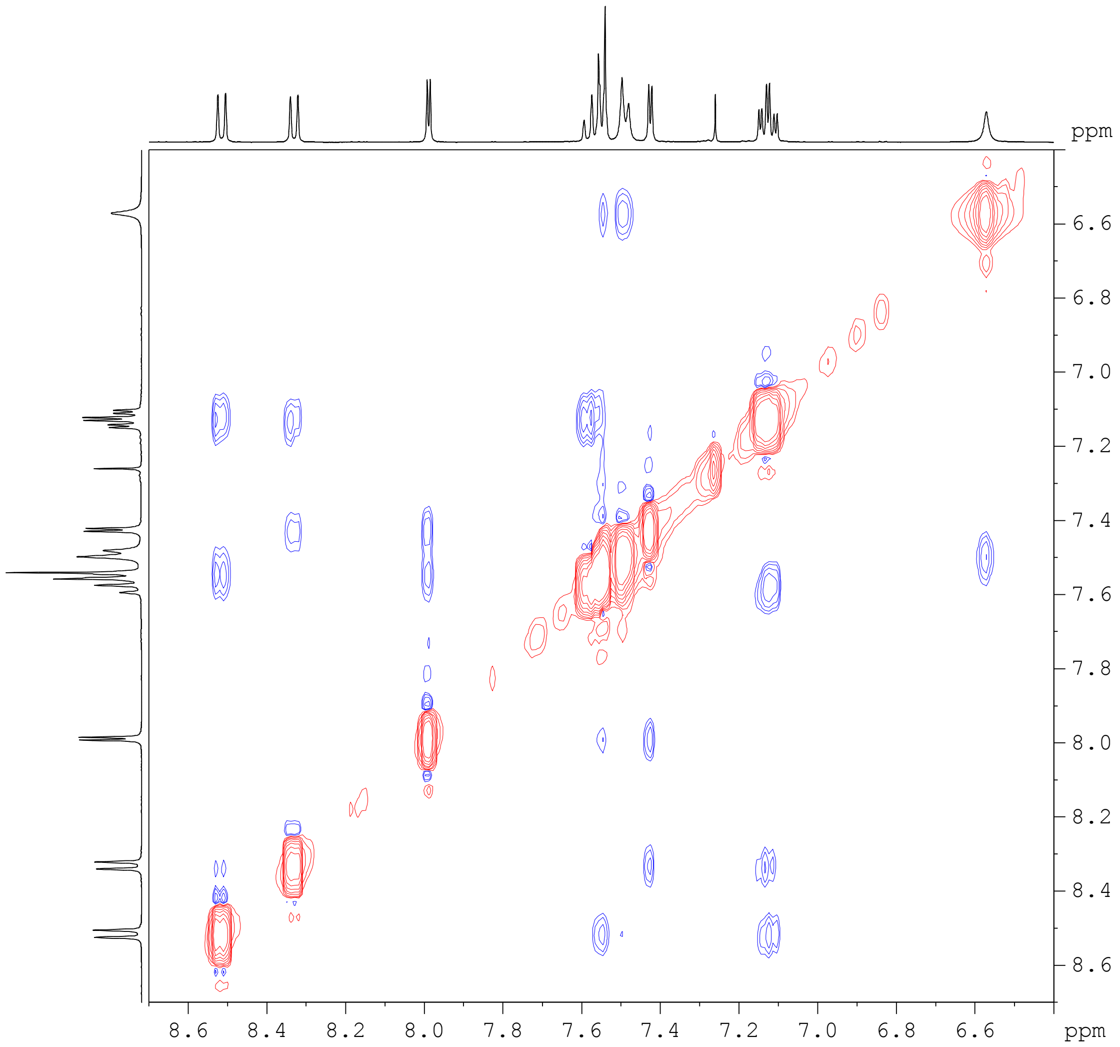

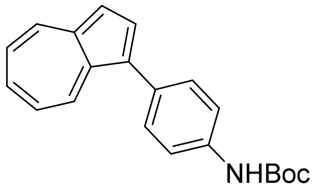

13b (500 MHz, $CDCl_3$)

HSQC

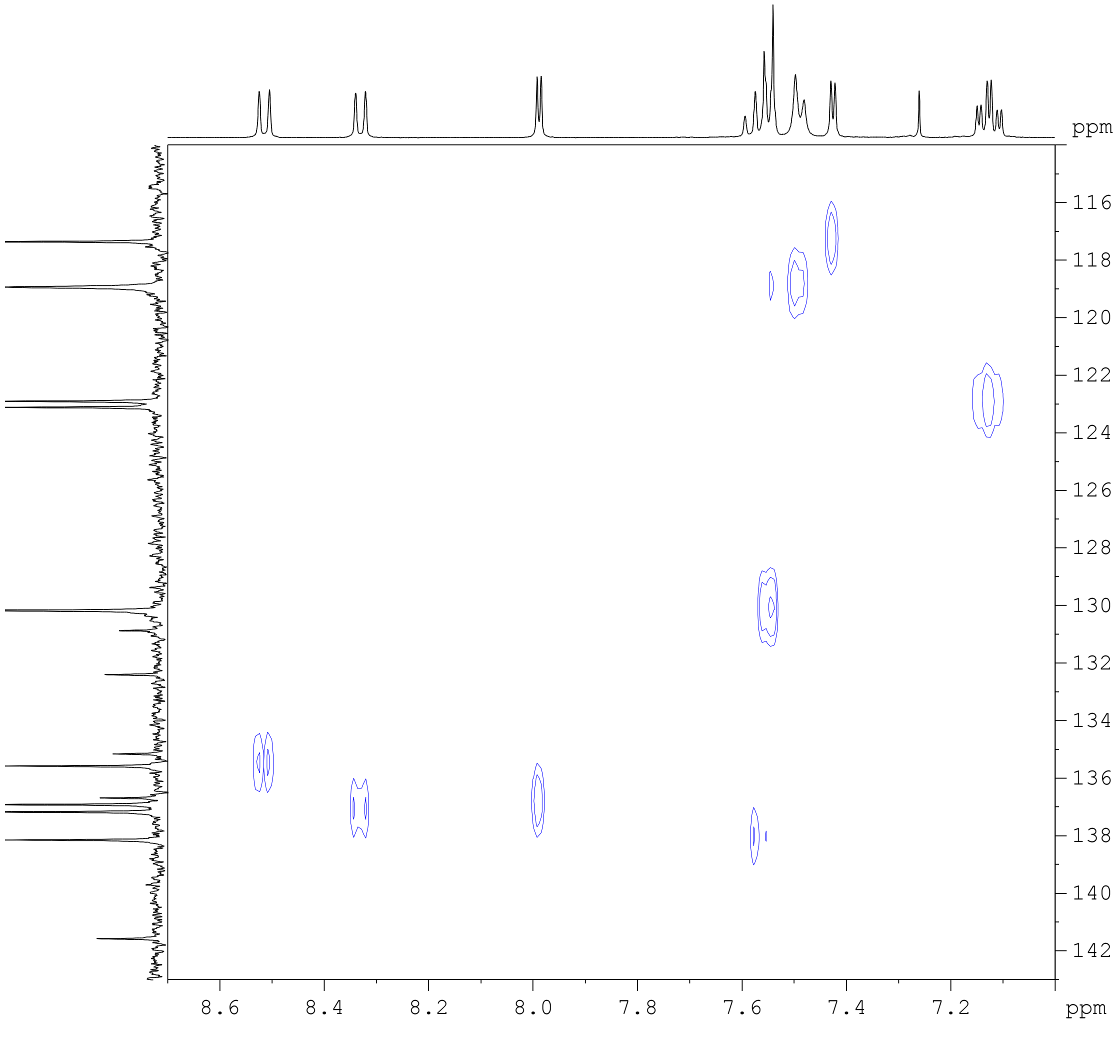

HMBC

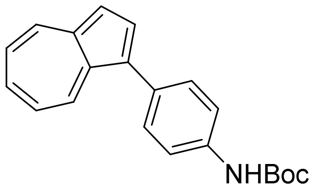

13b (500 MHz, $CDCl_3$)

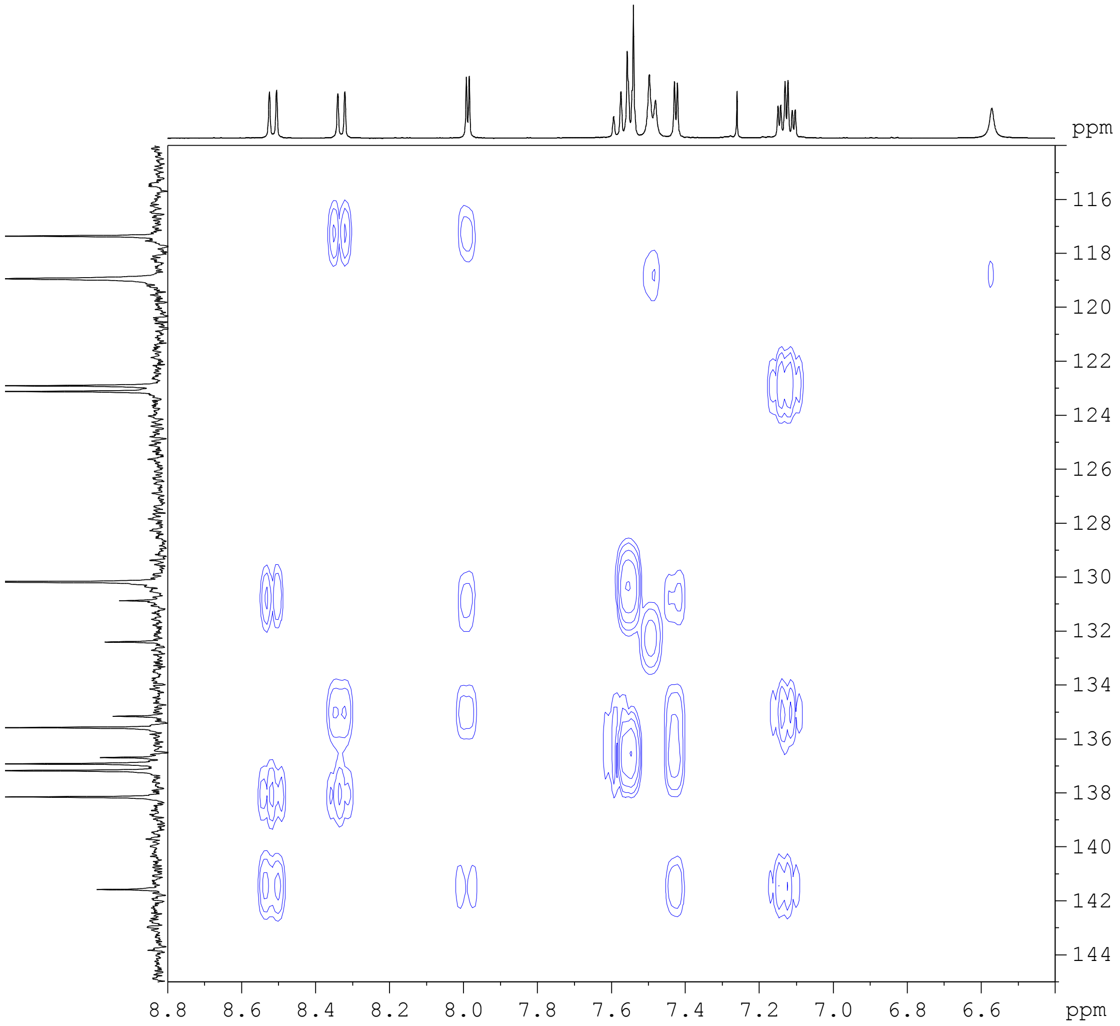

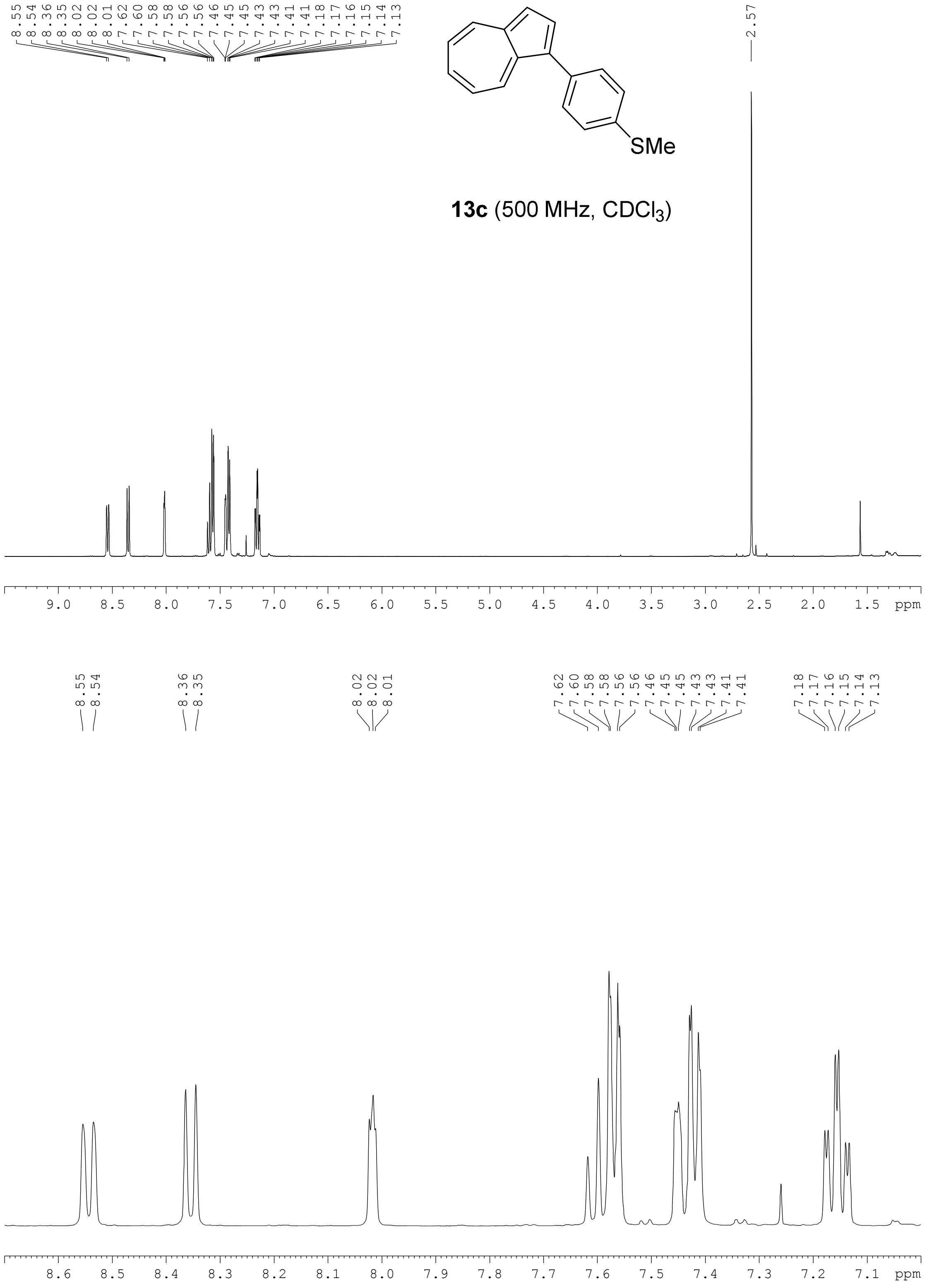

13c (500 MHz, $CDCl_3$)

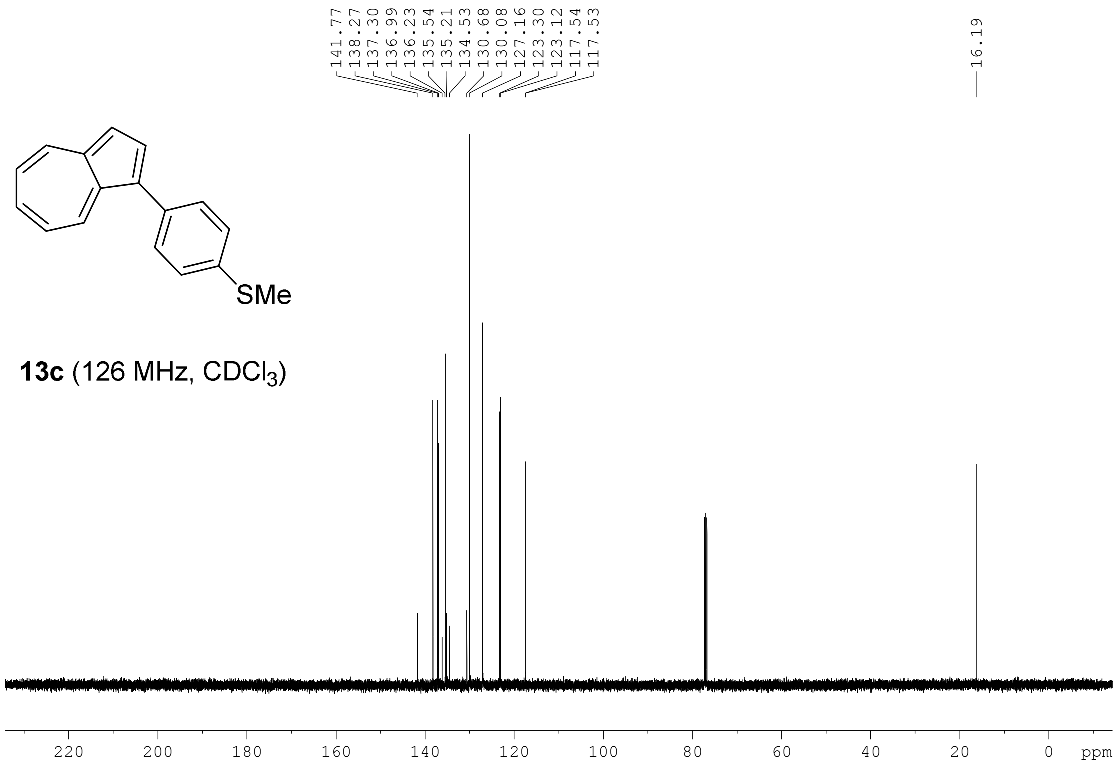

13c (126 MHz, $CDCl_3$)

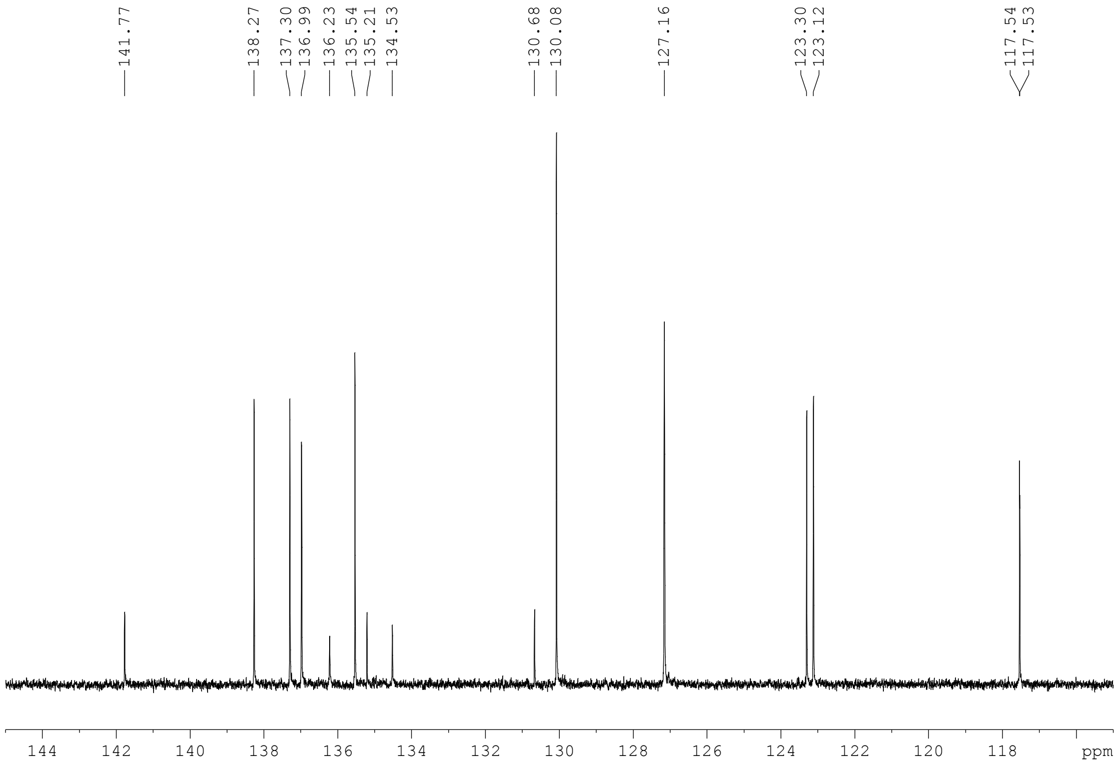

COSY

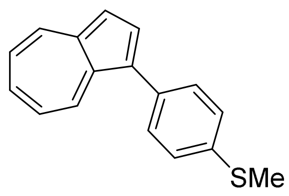

13c (500 MHz, $CDCl_3$)

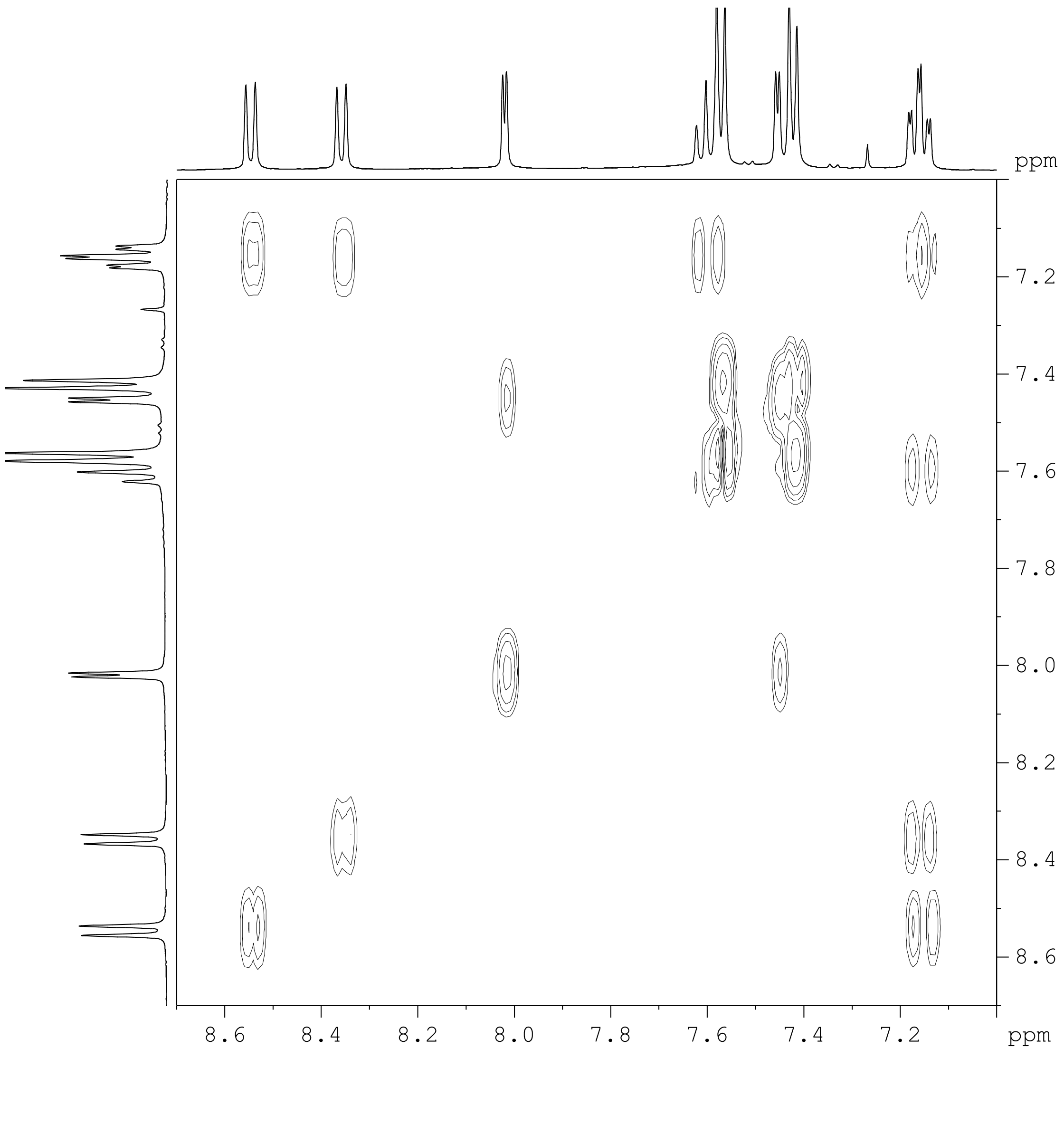

NOESY

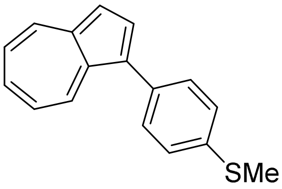

13c (500 MHz, $CDCl_3$)

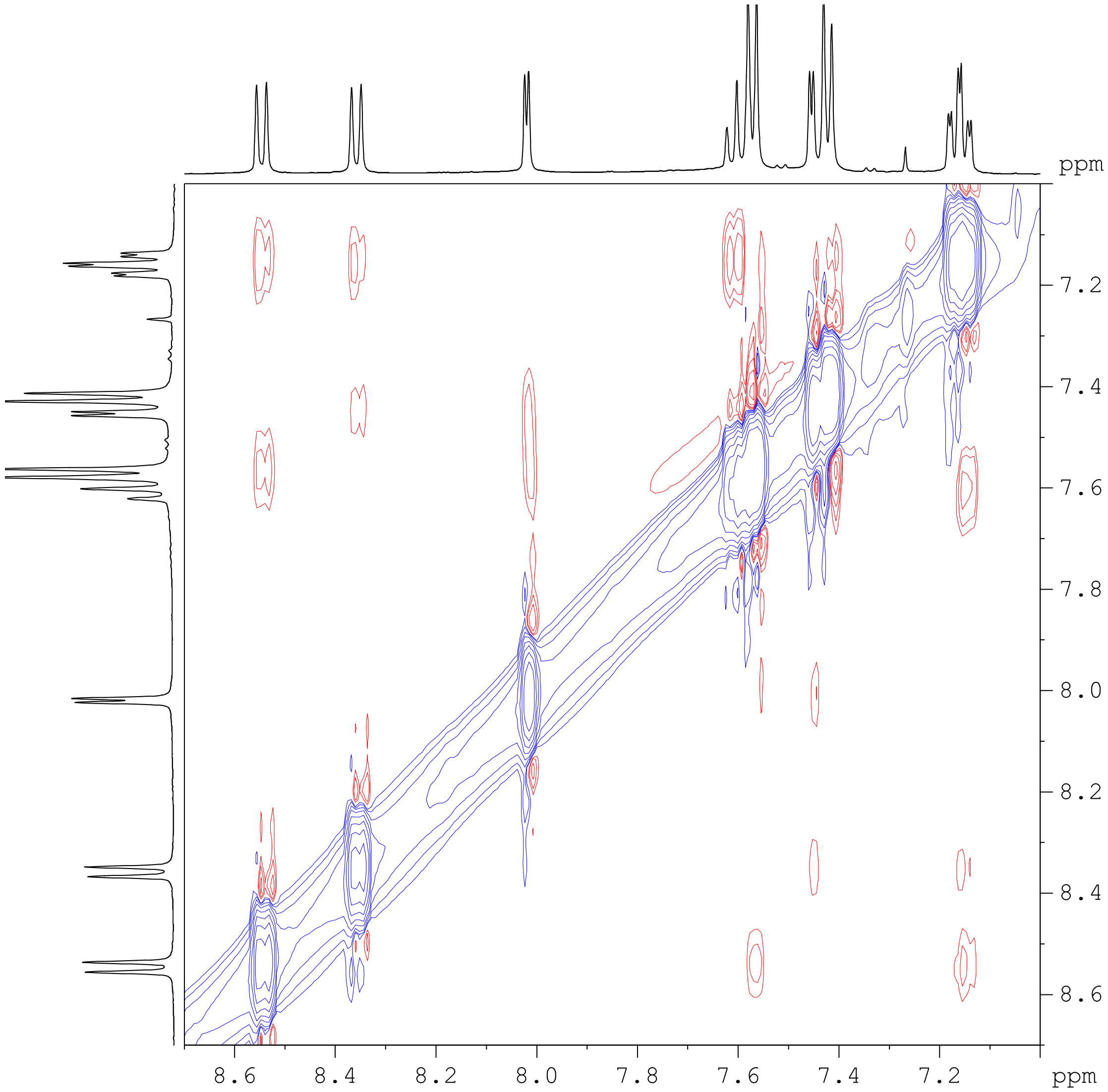

NOESY

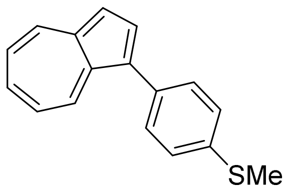

13c (500 MHz, $CDCl_3$)

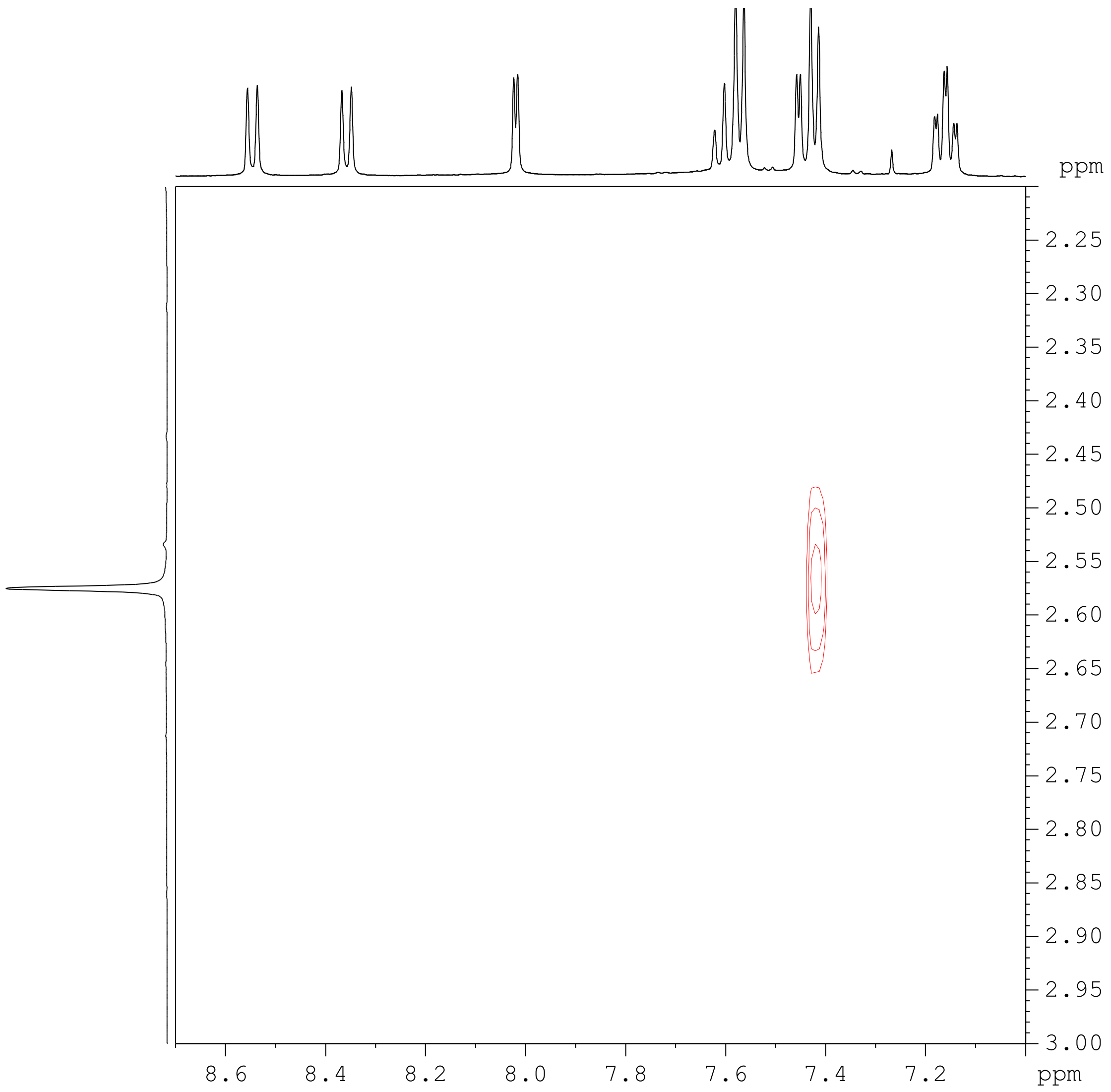

HSQC

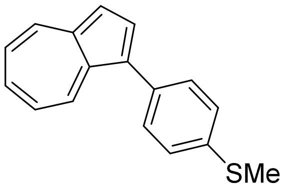

13c (500 MHz, $CDCl_3$)

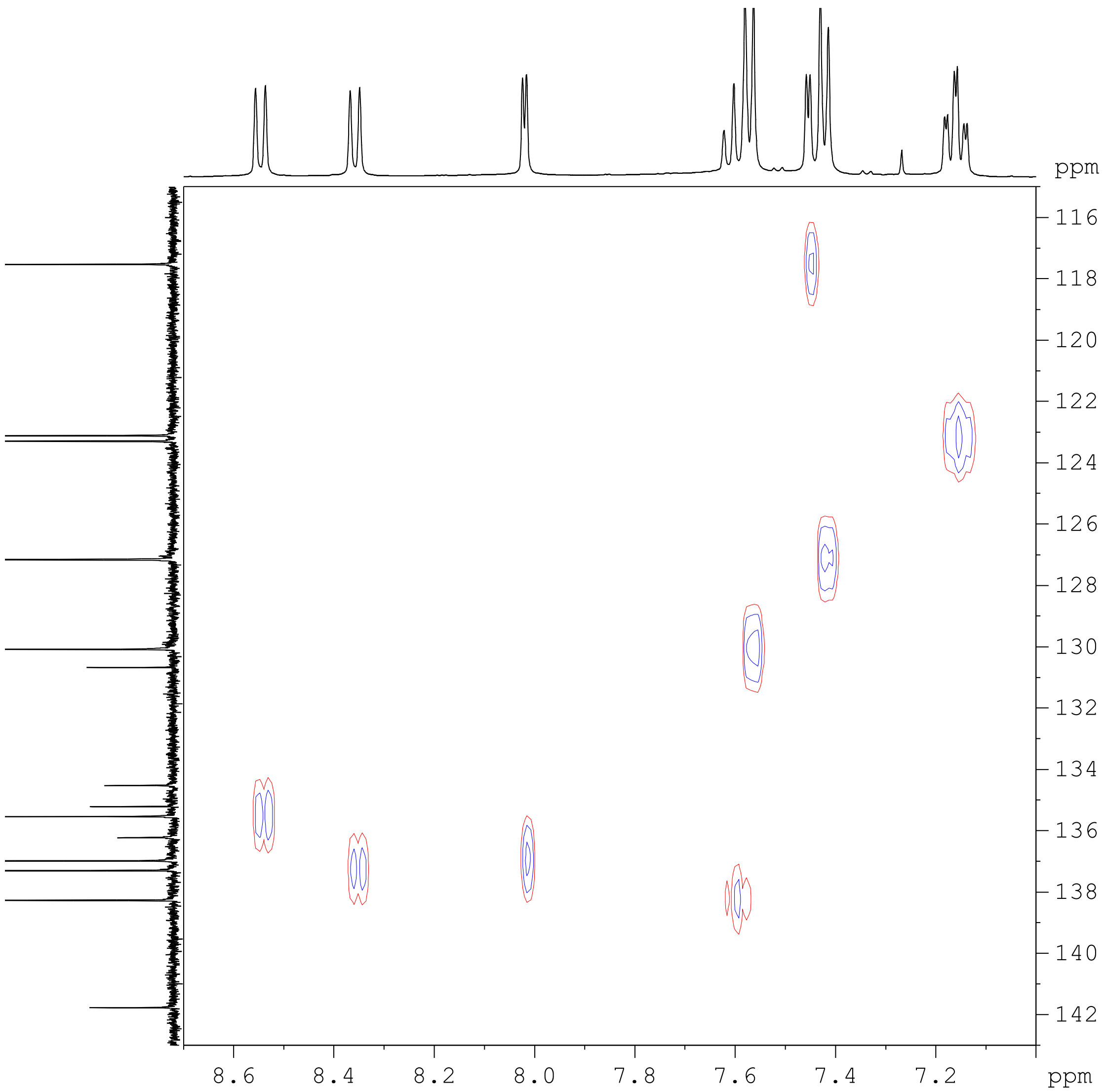

HMBC

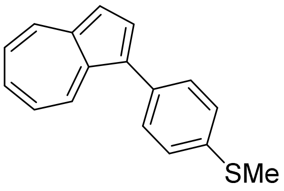

13c (500 MHz, $CDCl_3$)

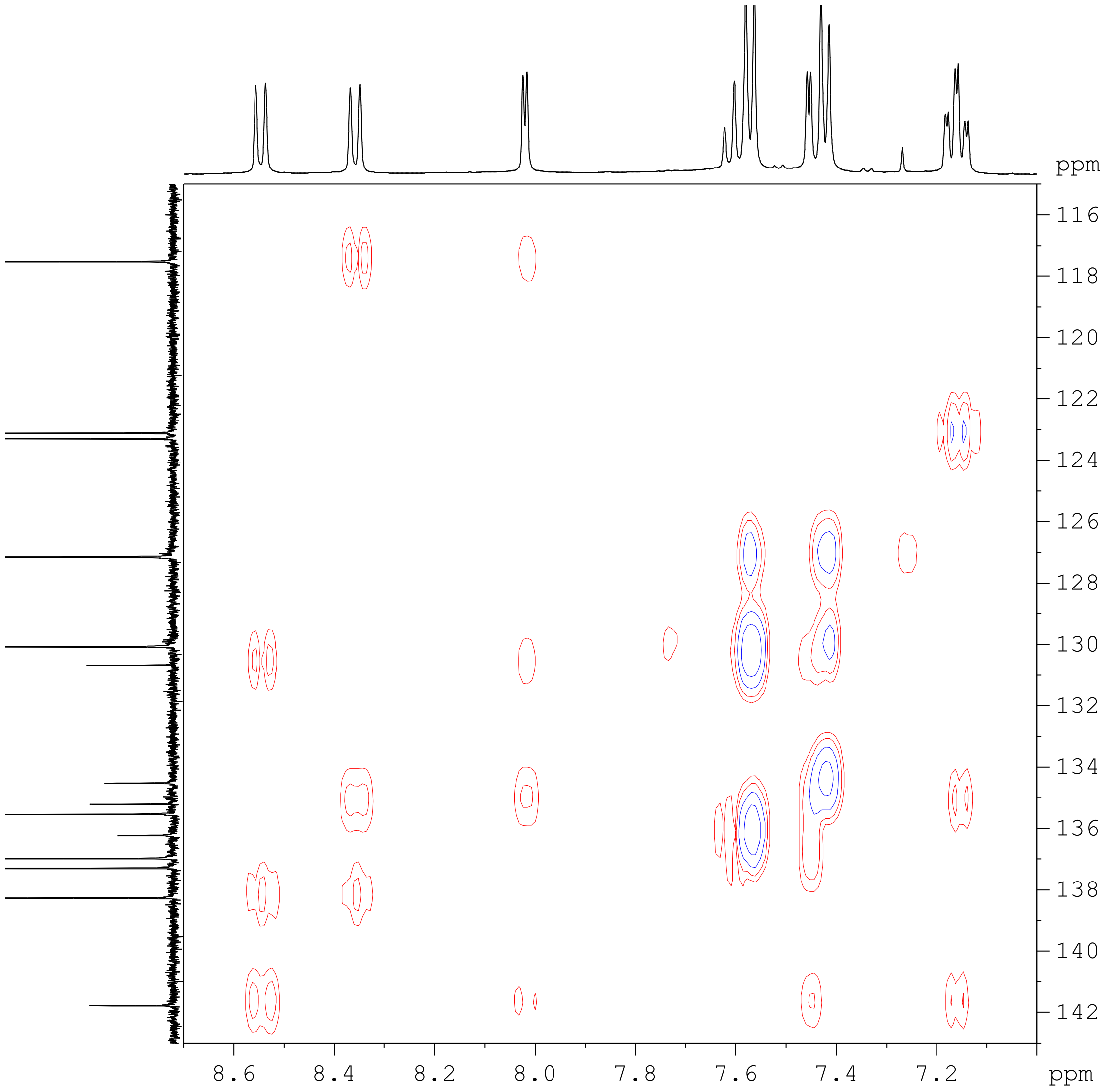

HMBC

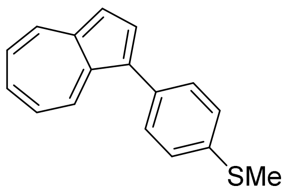

13c (500 MHz, $CDCl_3$)

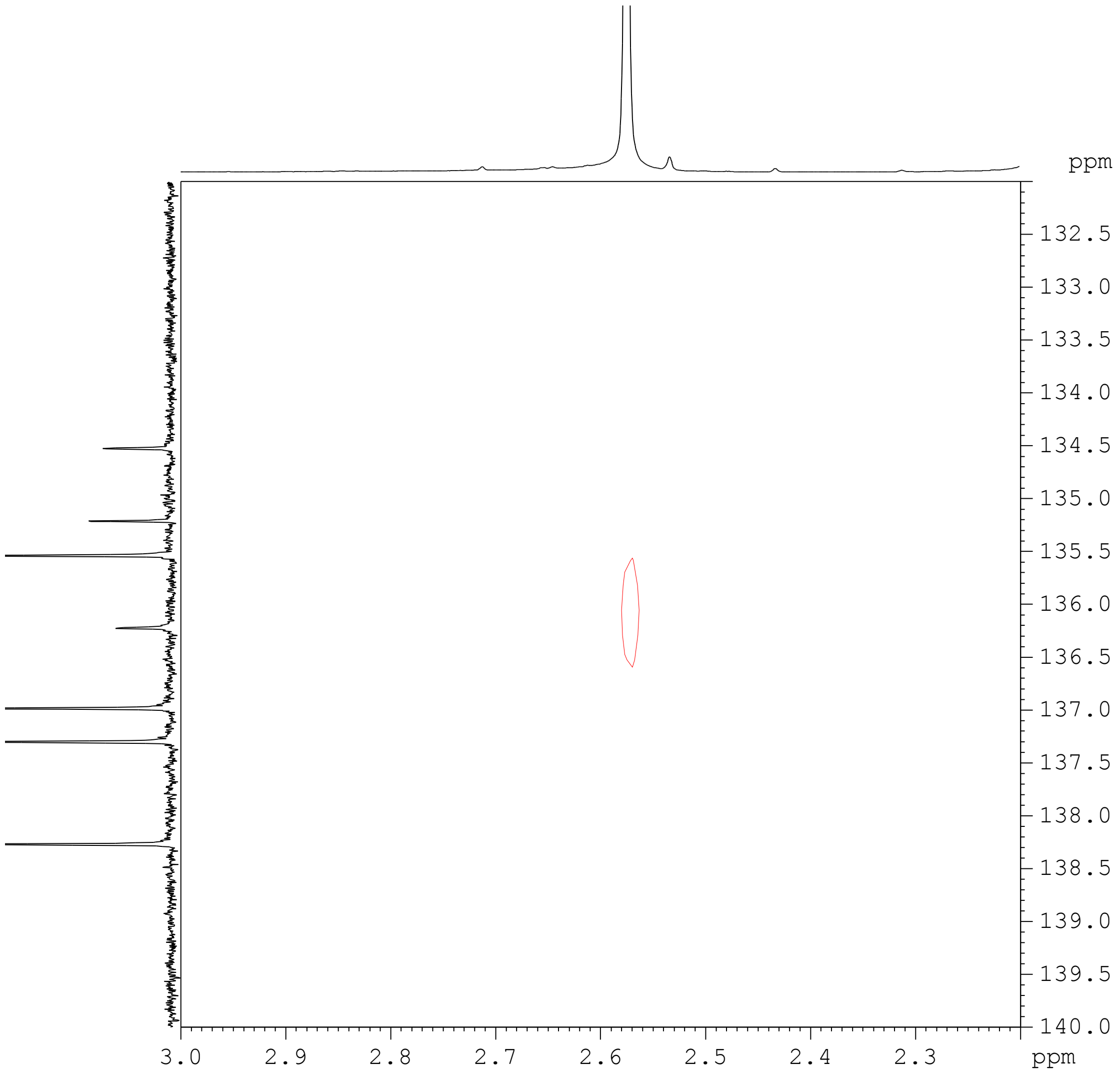

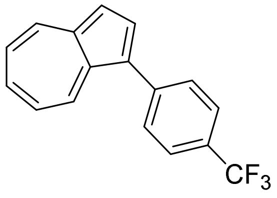

13d (500 MHz, $CDCl_3$)

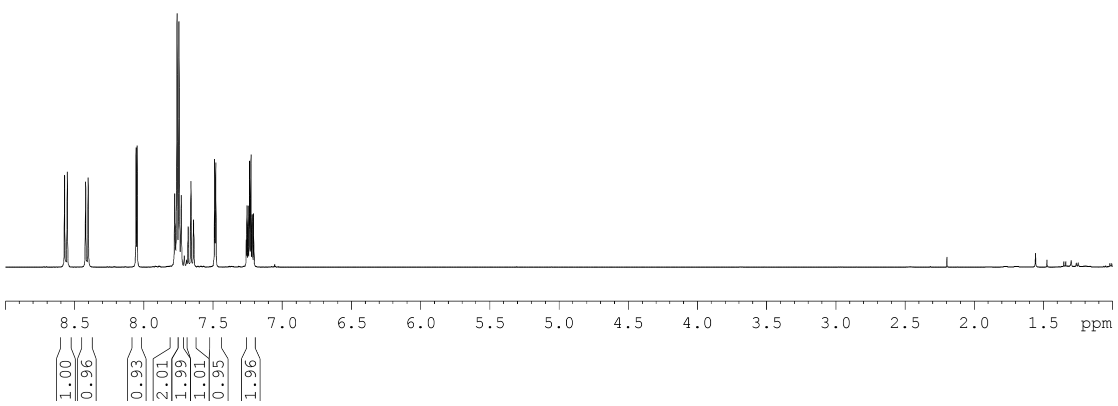

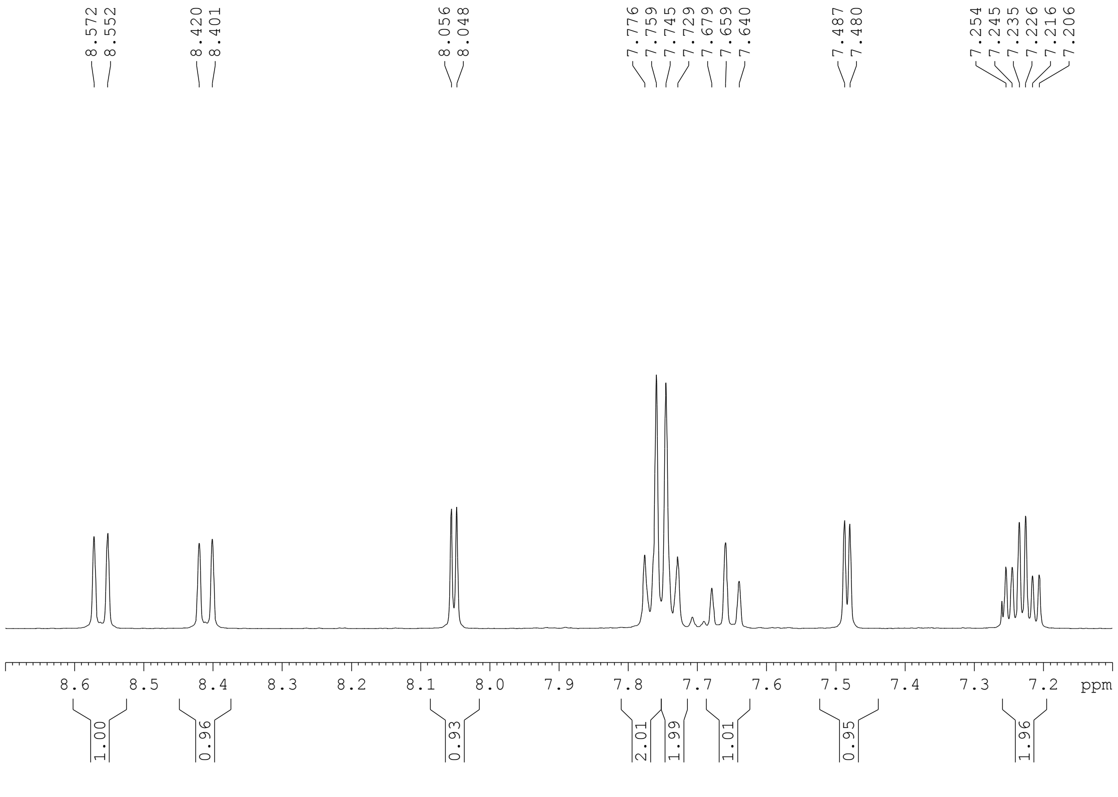

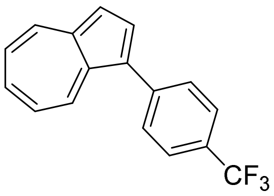

13d (126 MHz, $CDCl_3$)

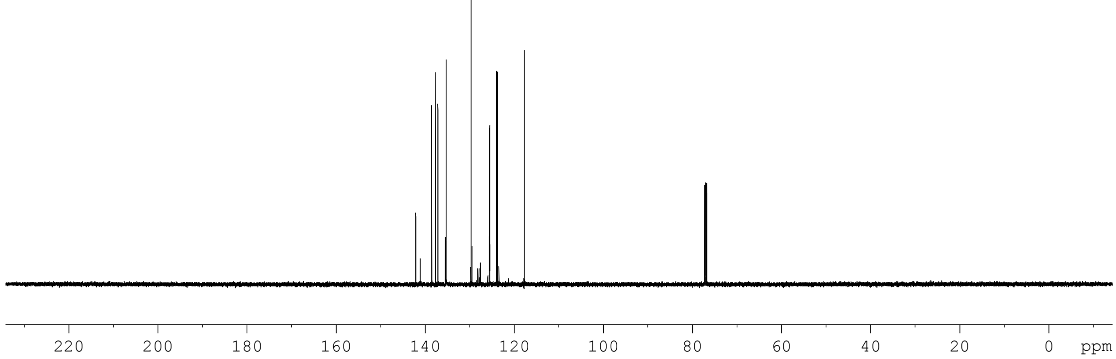

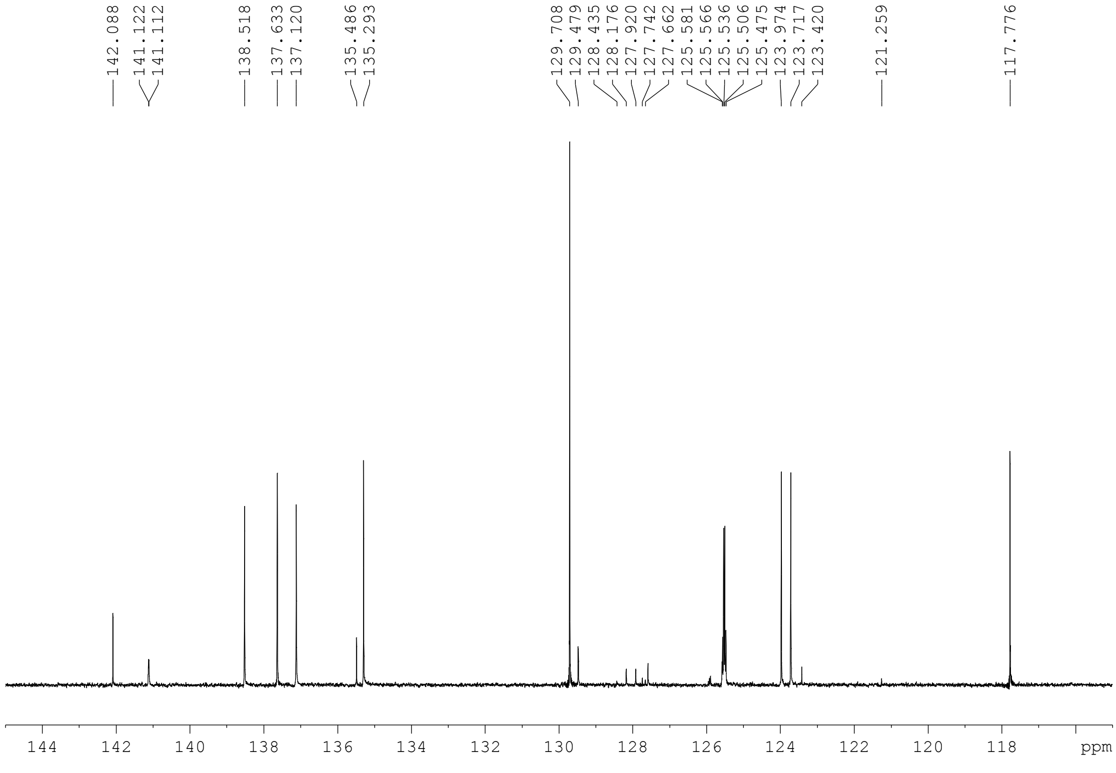

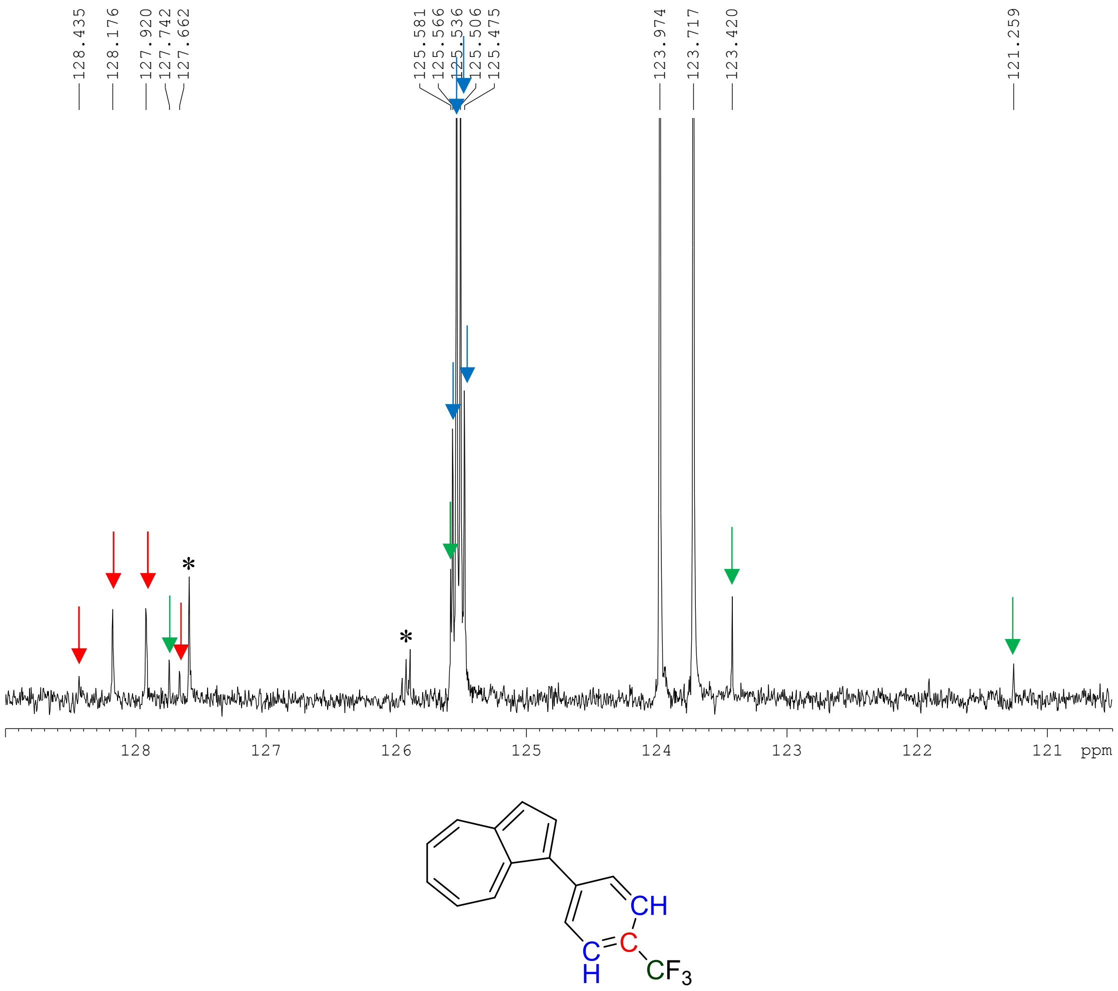
128.435
128.176
127.920
127.742
127.662
125.581
125.566
125.536
125.506
125.475
123.974
123.717
123.420
121.259
*
*
128
127
126
125
124
123
122
121
ppm
CH
C
C
H
CF3

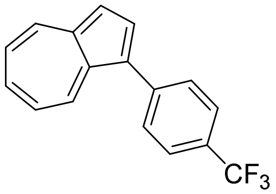

13d (470.5 MHz, $CDCl_3$)

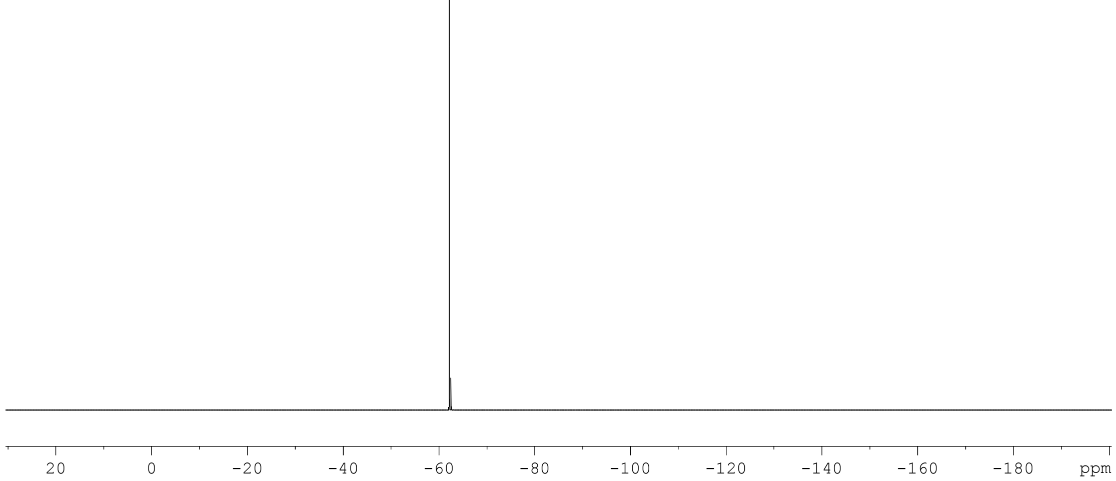

COSY

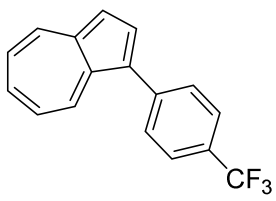

13d (500 MHz, $CDCl_3$)

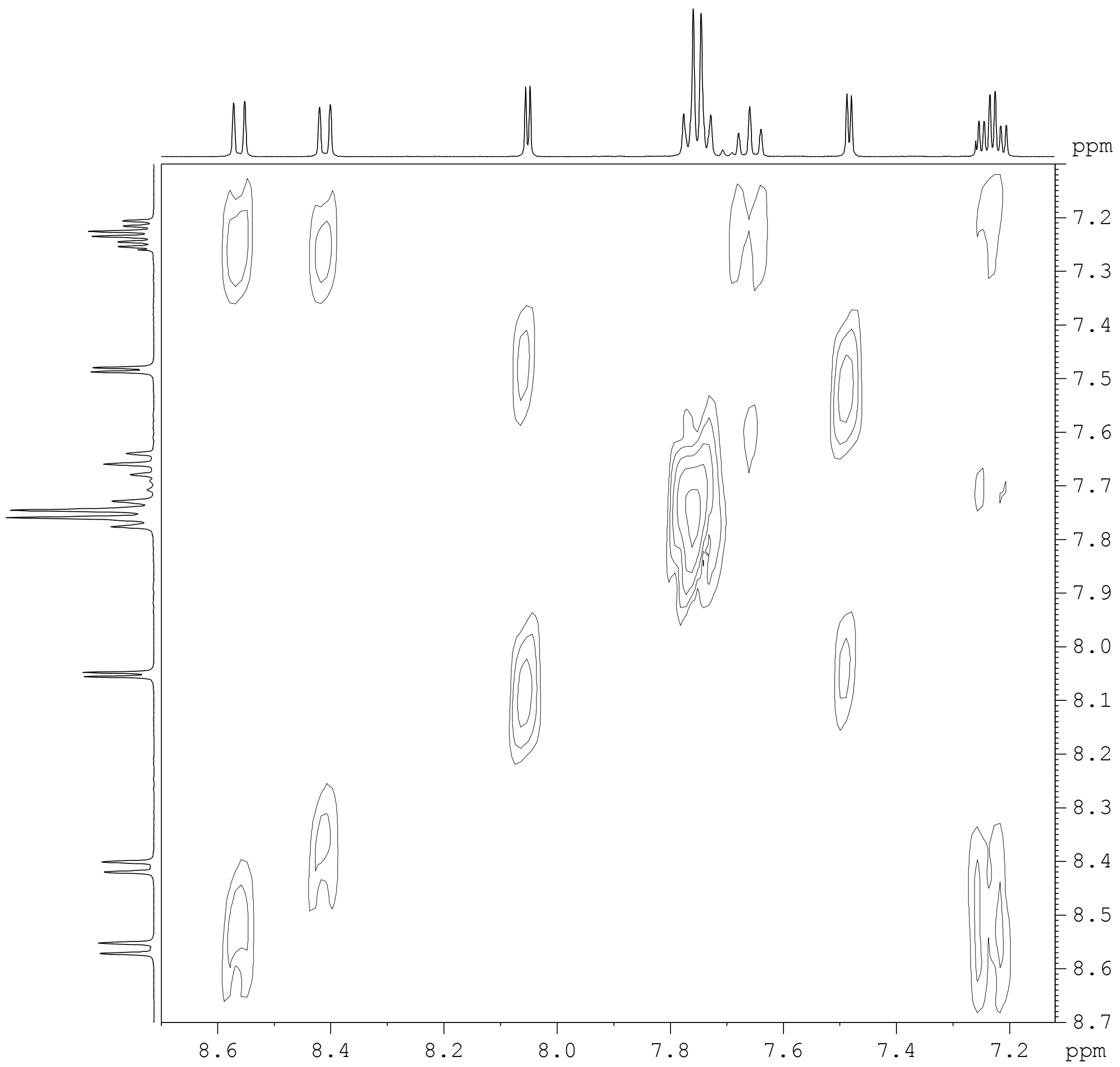

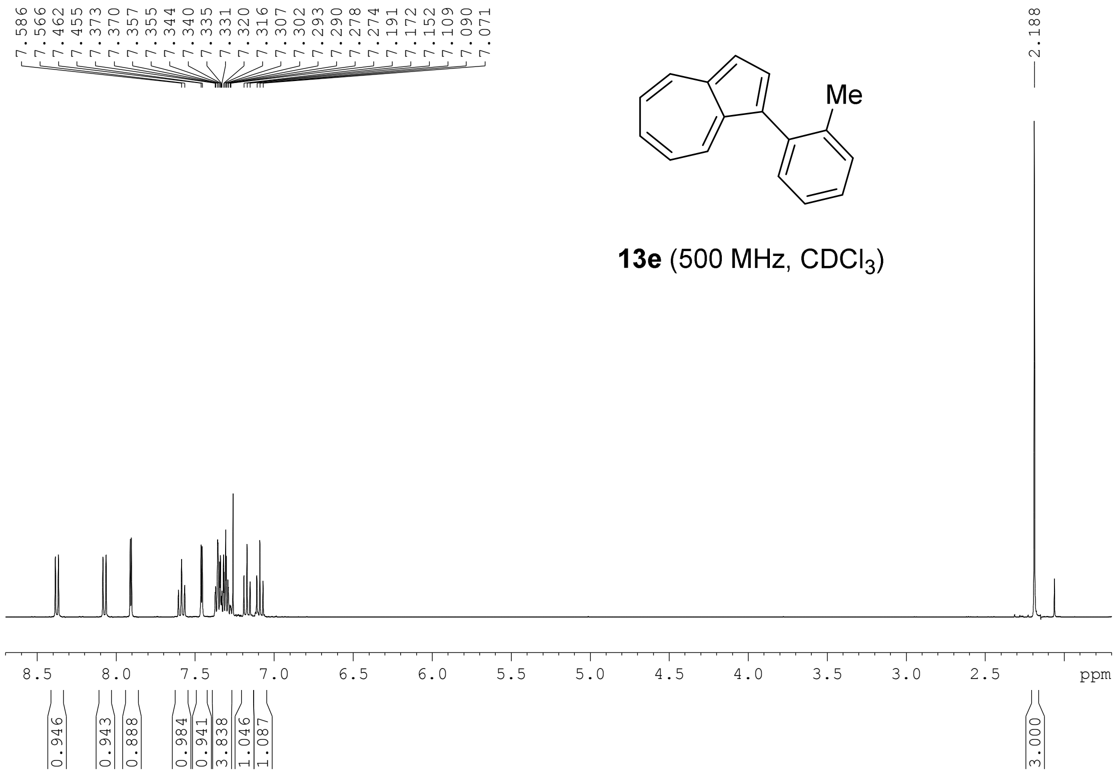
Me
13e (500 MHz, CDCl3)
ppm

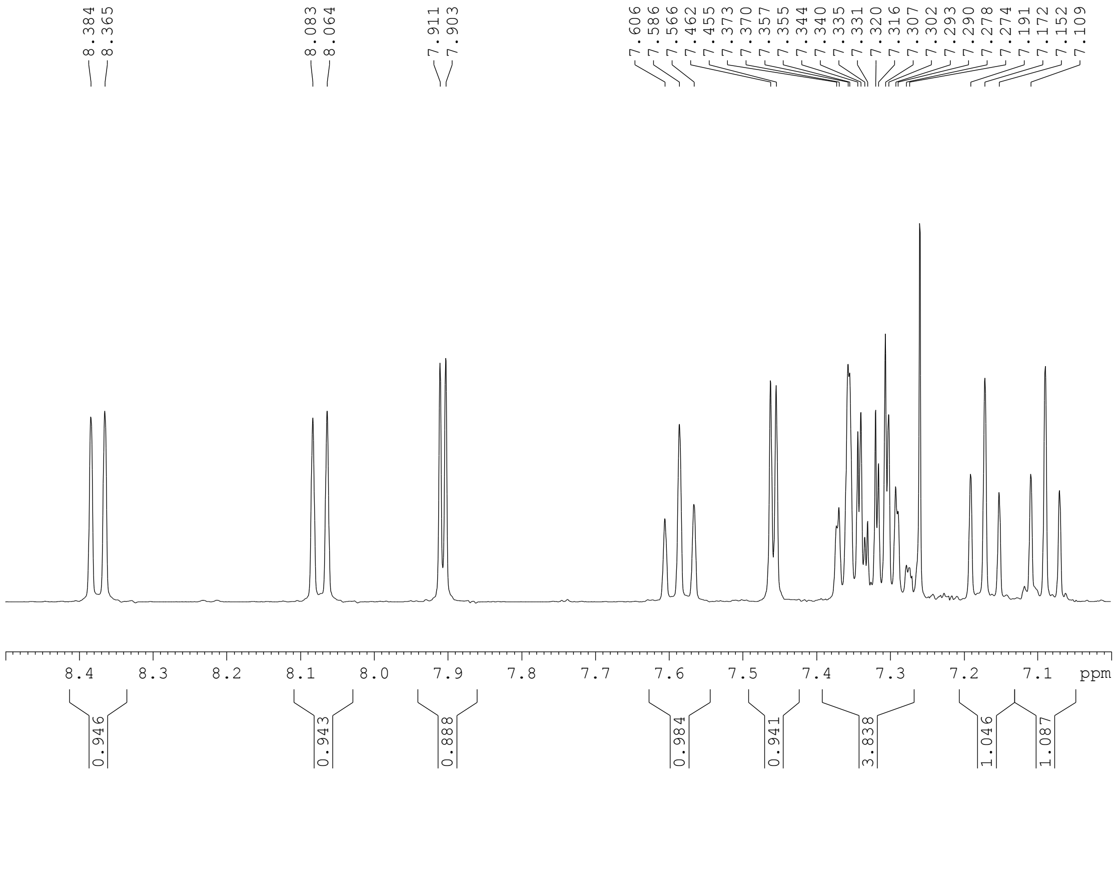
ppm

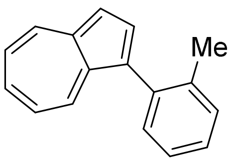

13e (126 MHz, $CDCl_3$)

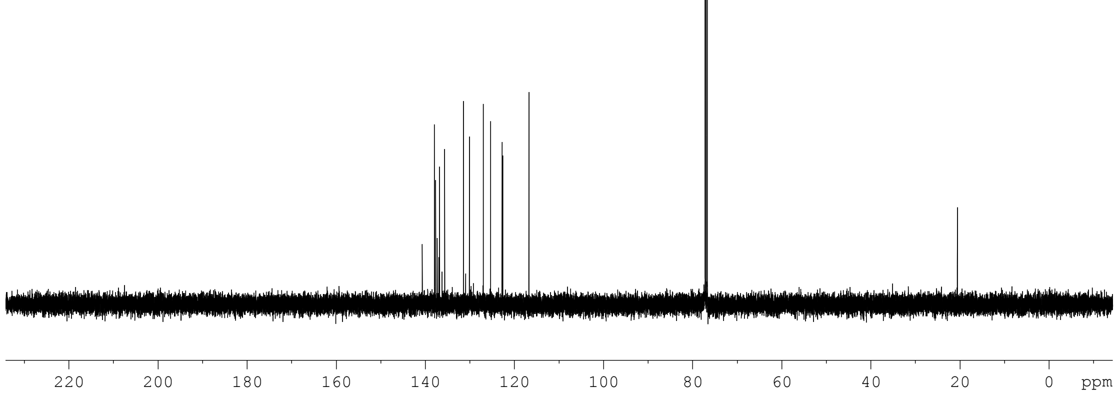

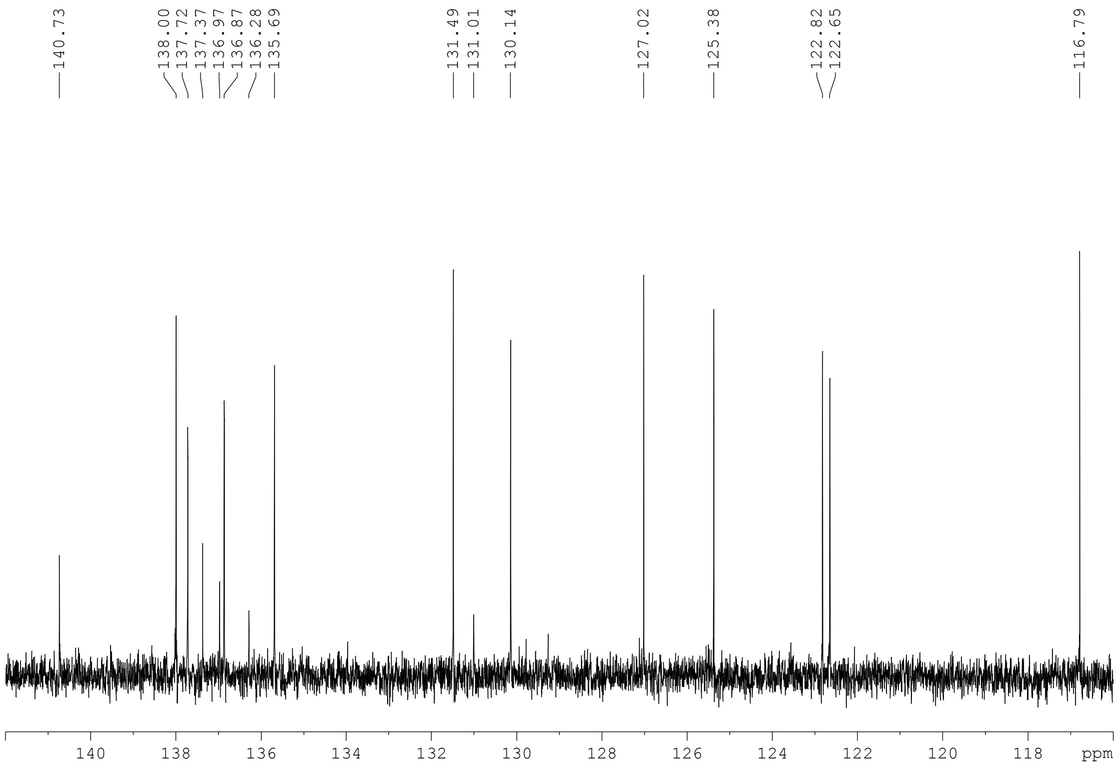

COSY

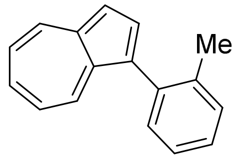

13e (500 MHz, $CDCl_3$)

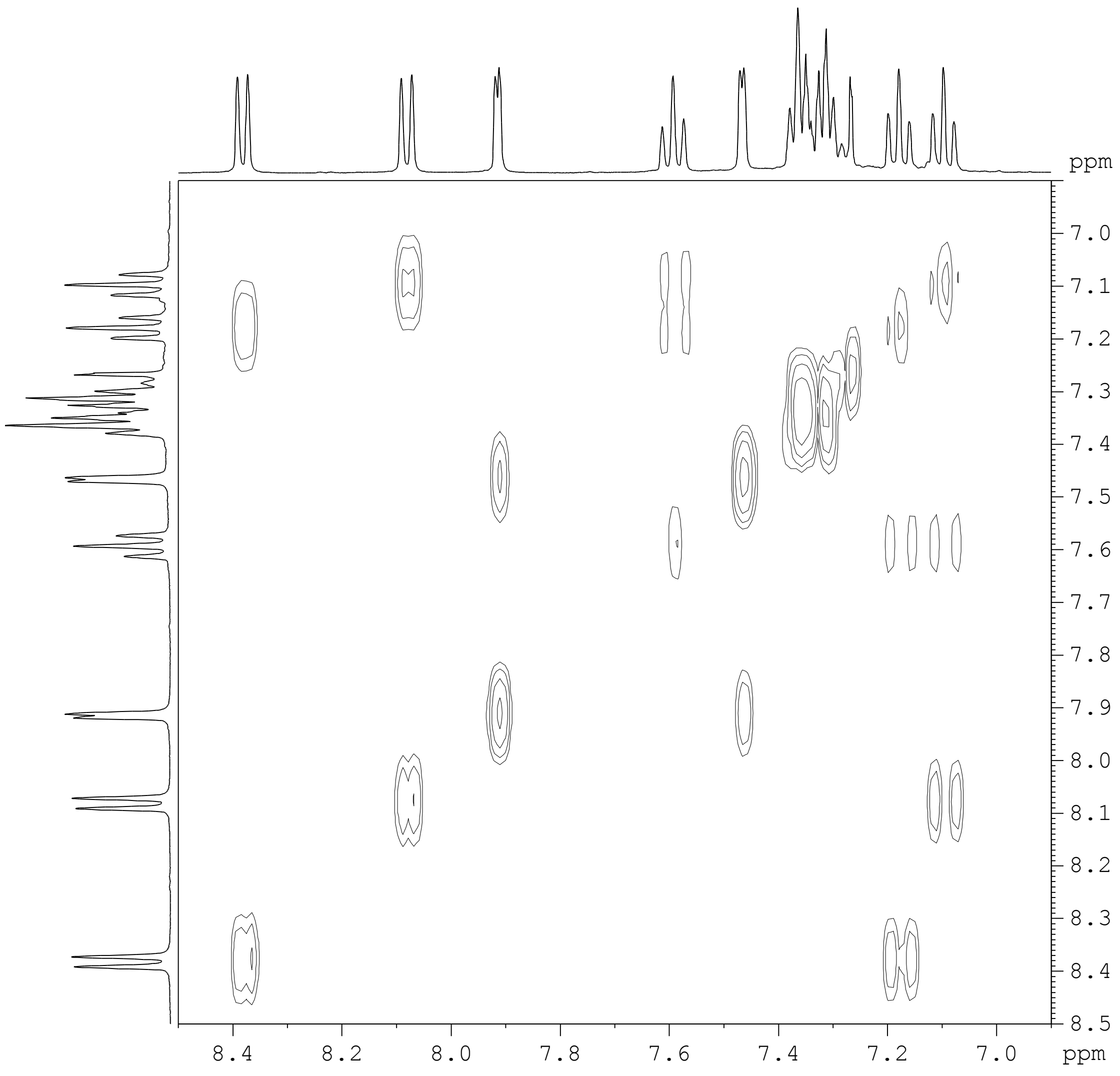

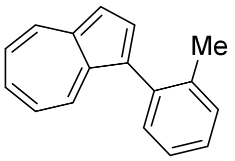

13e (500 MHz, $CDCl_3$)

NOESY

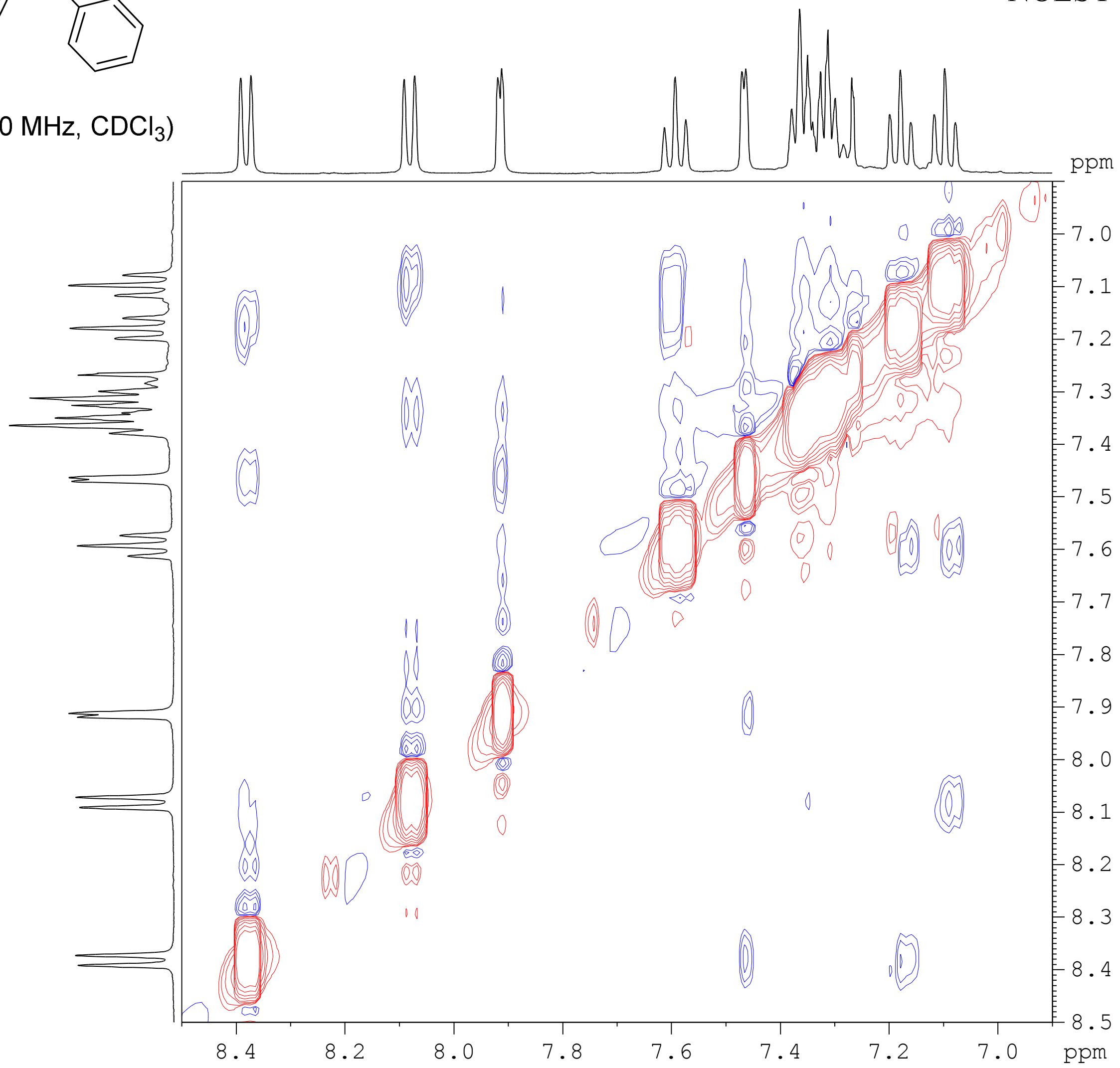

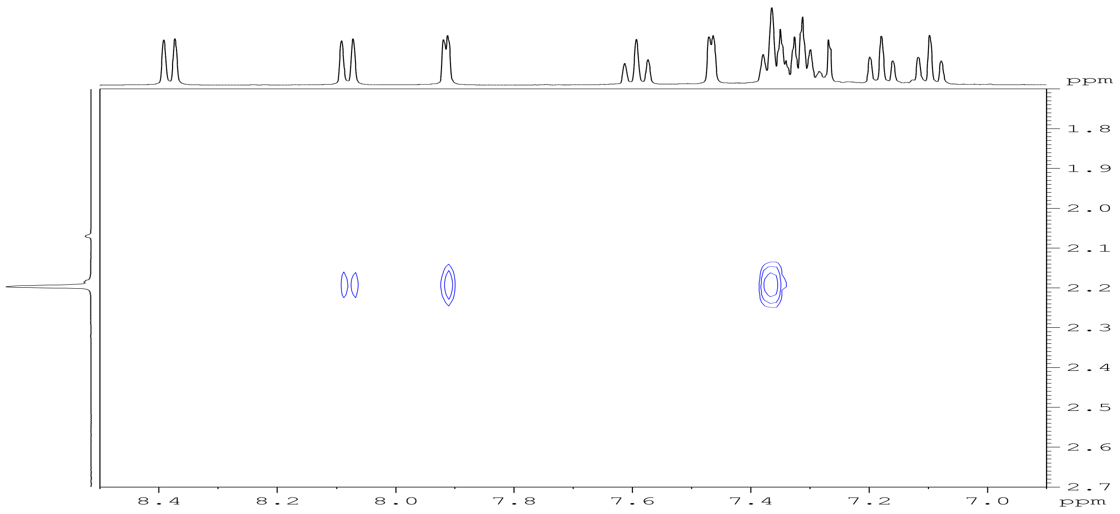

HSQC

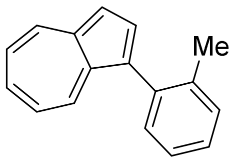

13e (500 MHz, $CDCl_3$)

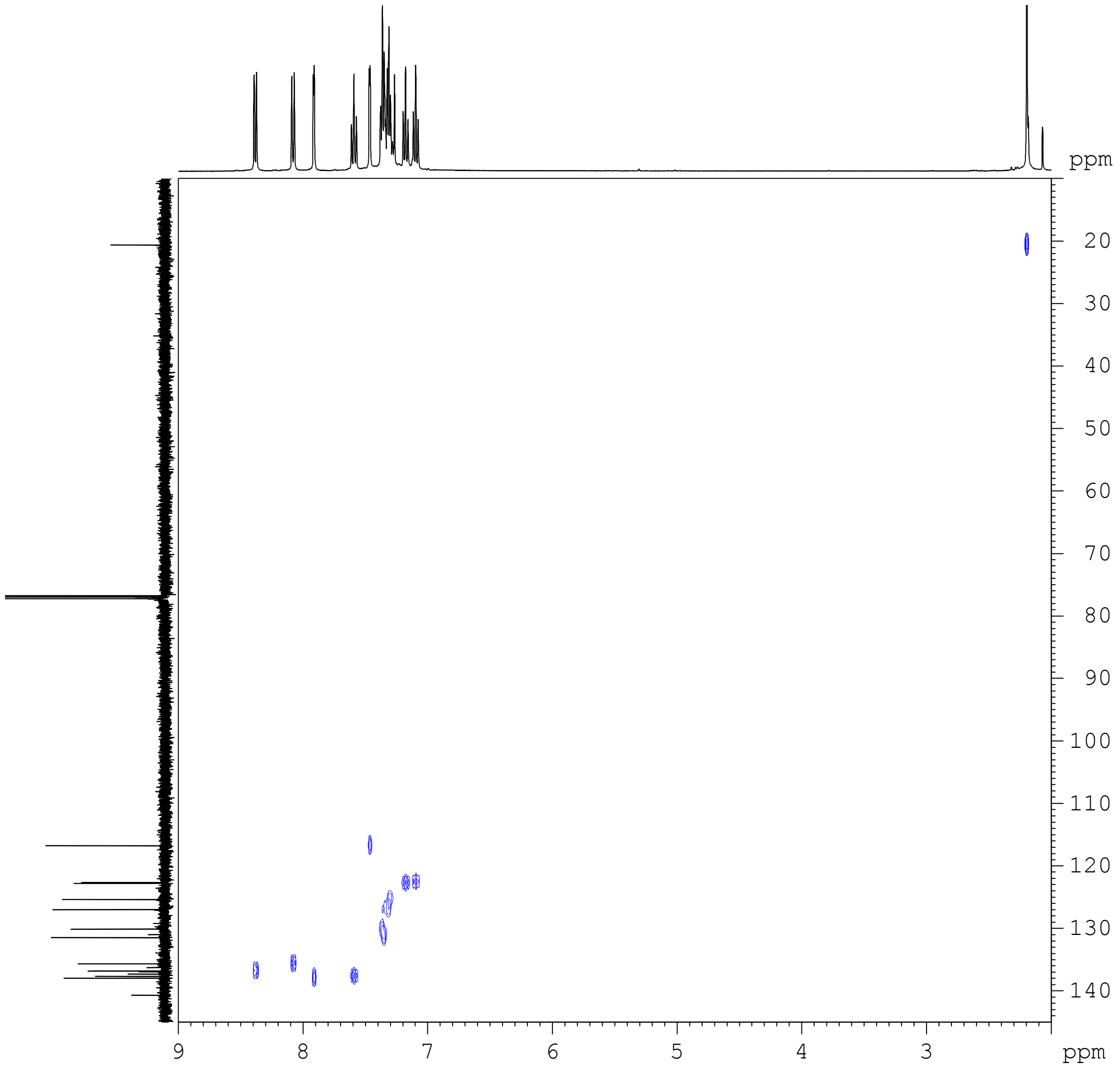

HSQC

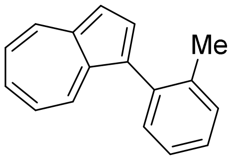

13e (500 MHz, $CDCl_3$)

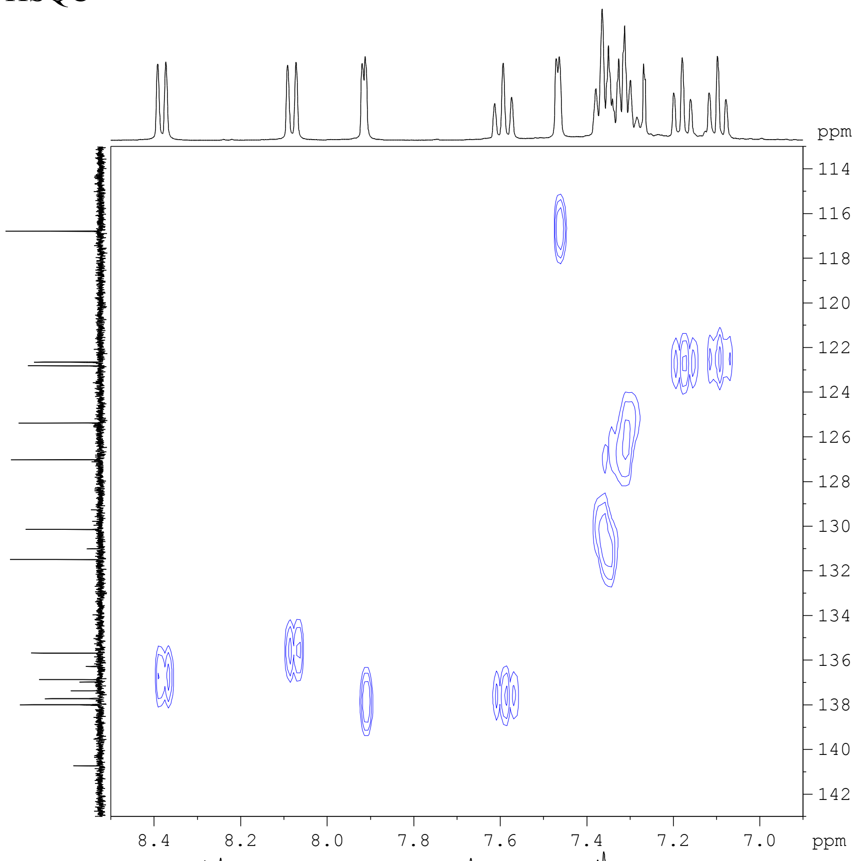

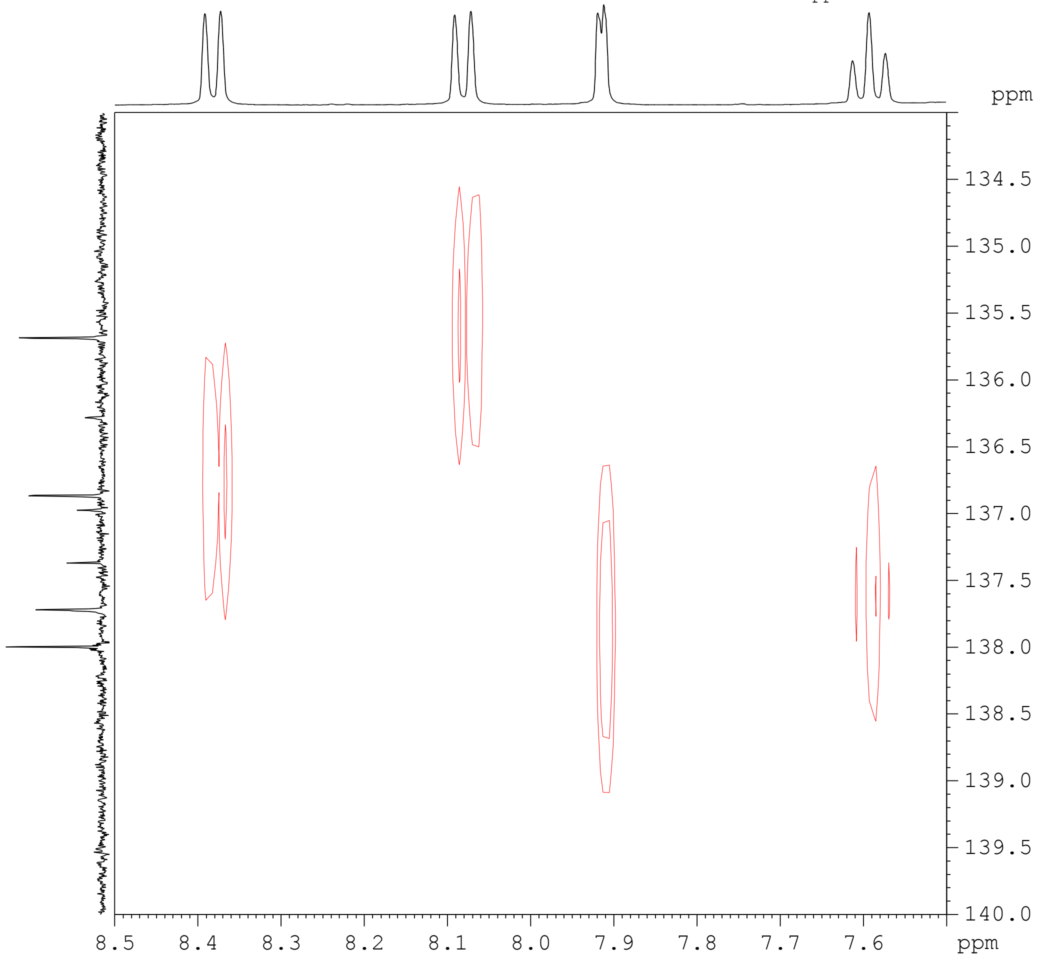

HMBC

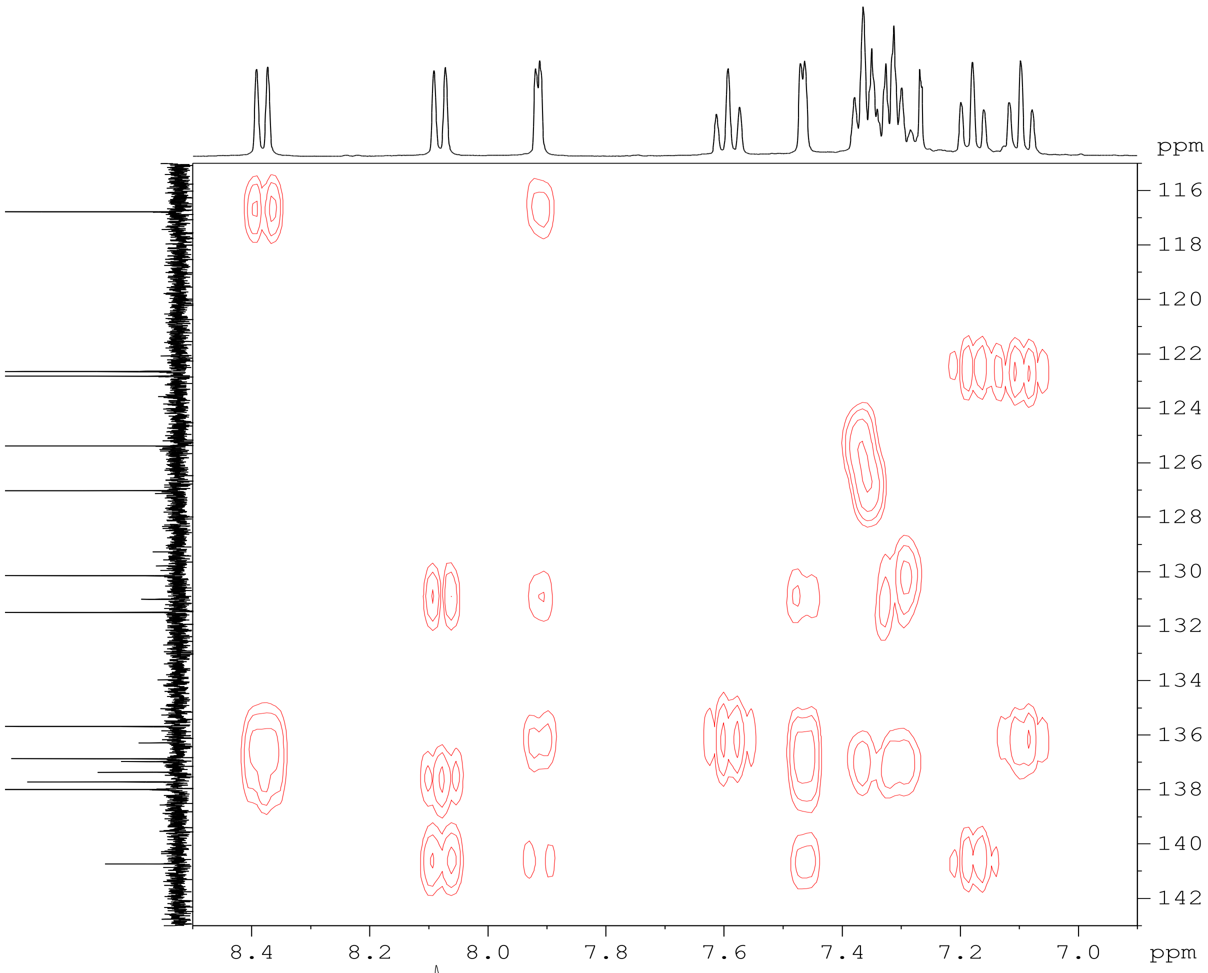

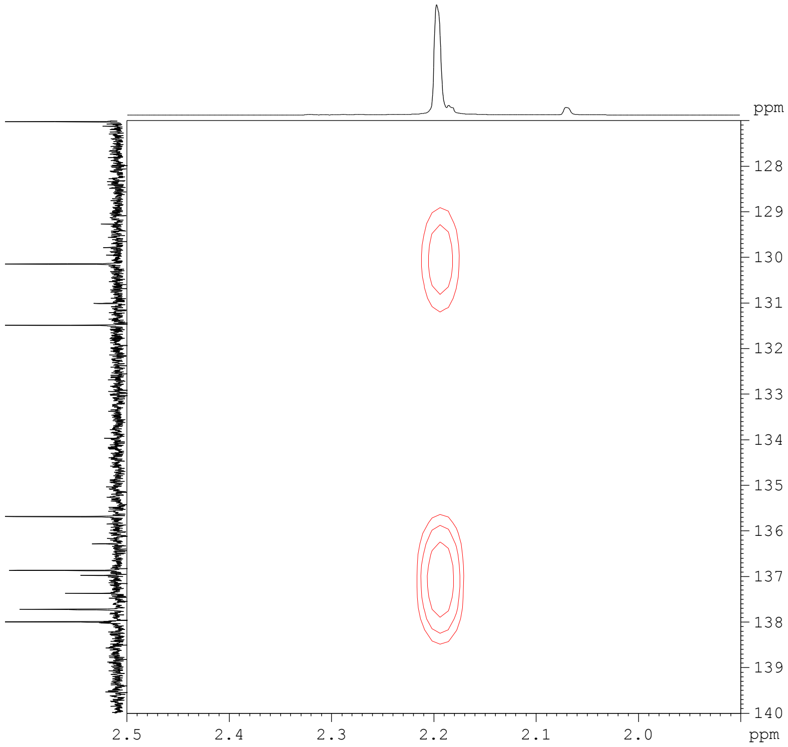

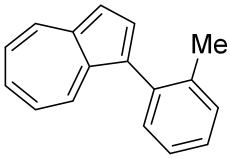

13e (500 MHz, $CDCl_3$)

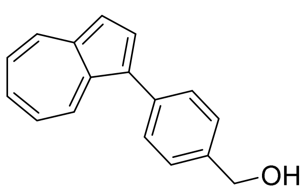

13f (500 MHz, $CDCl_3$)

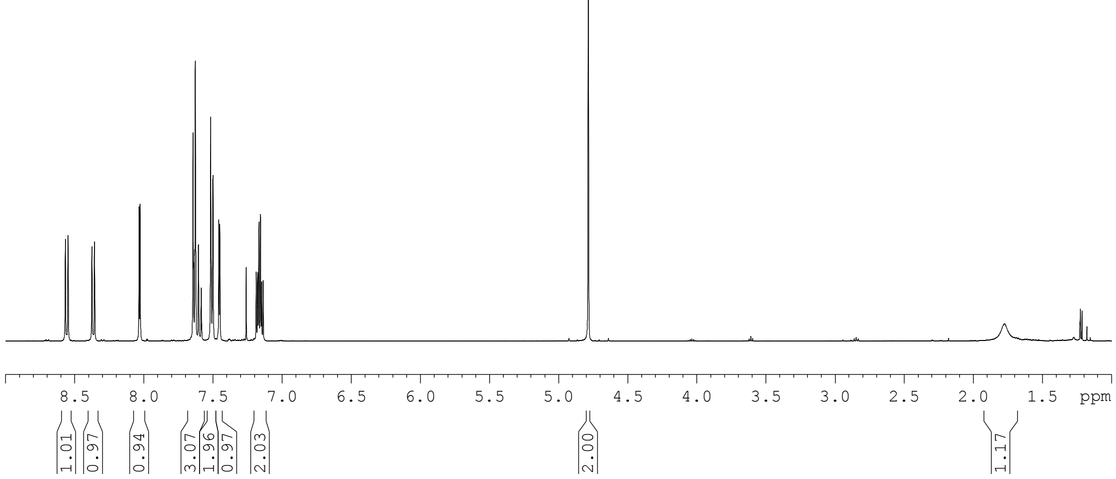

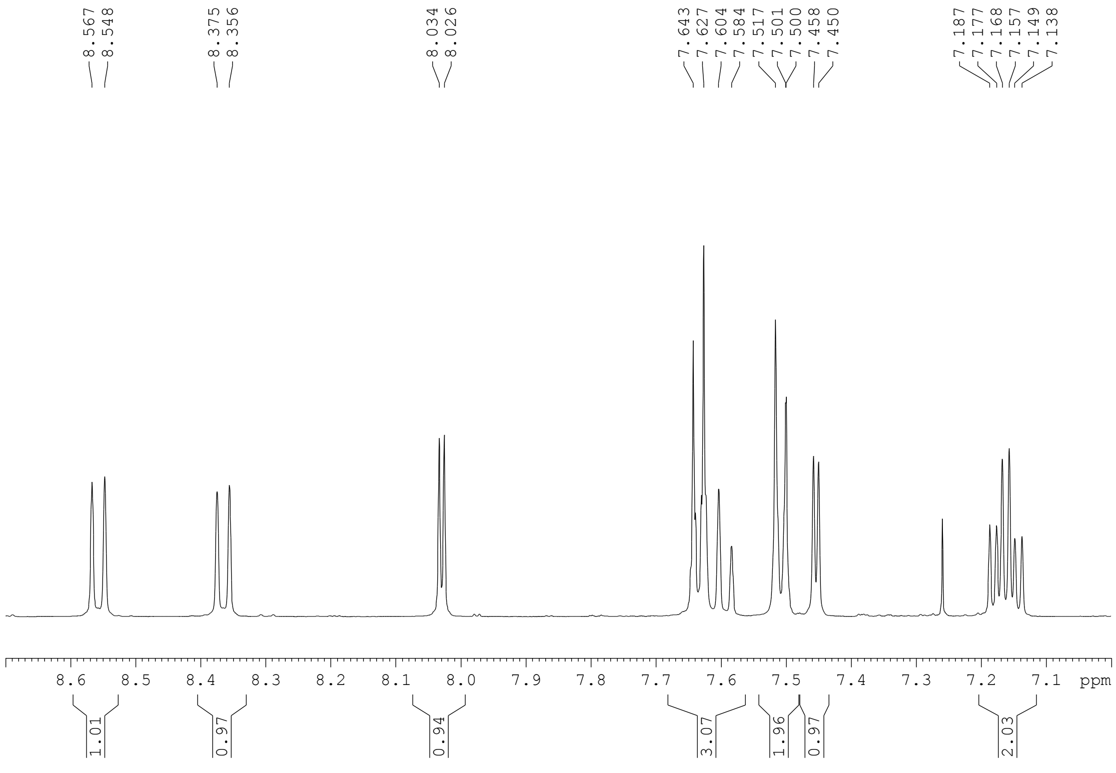

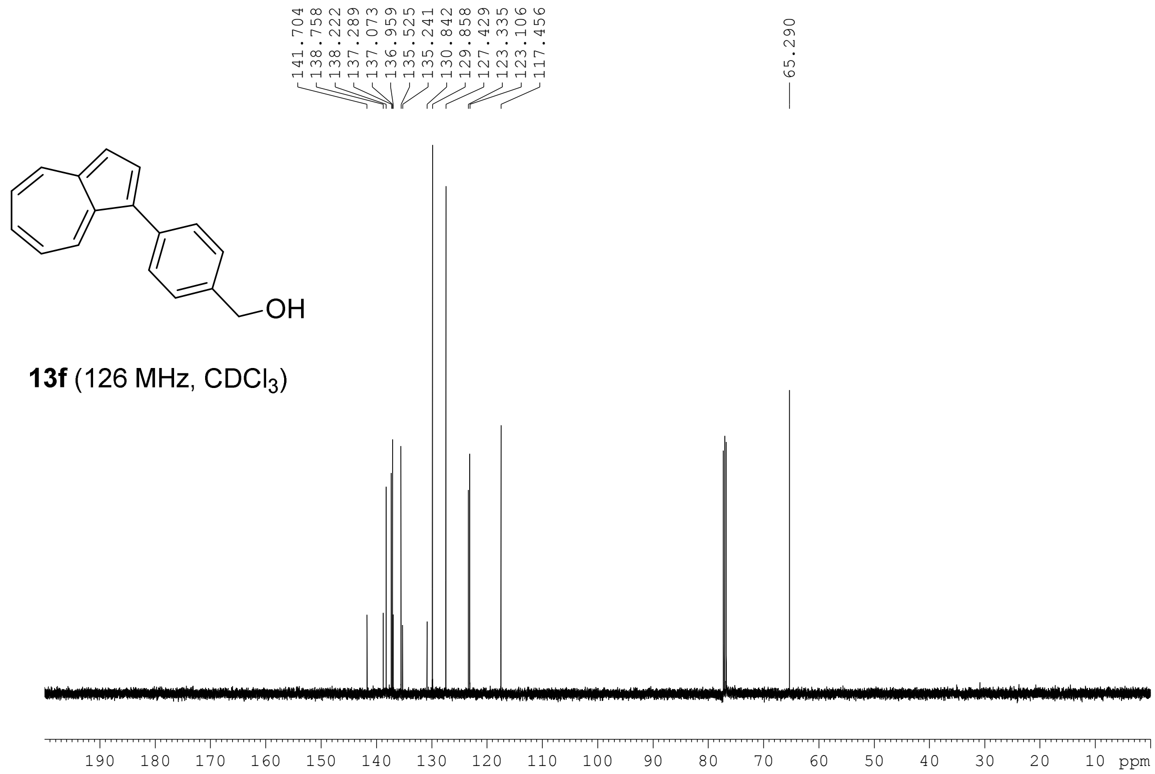

13f (126 MHz, $CDCl_3$)

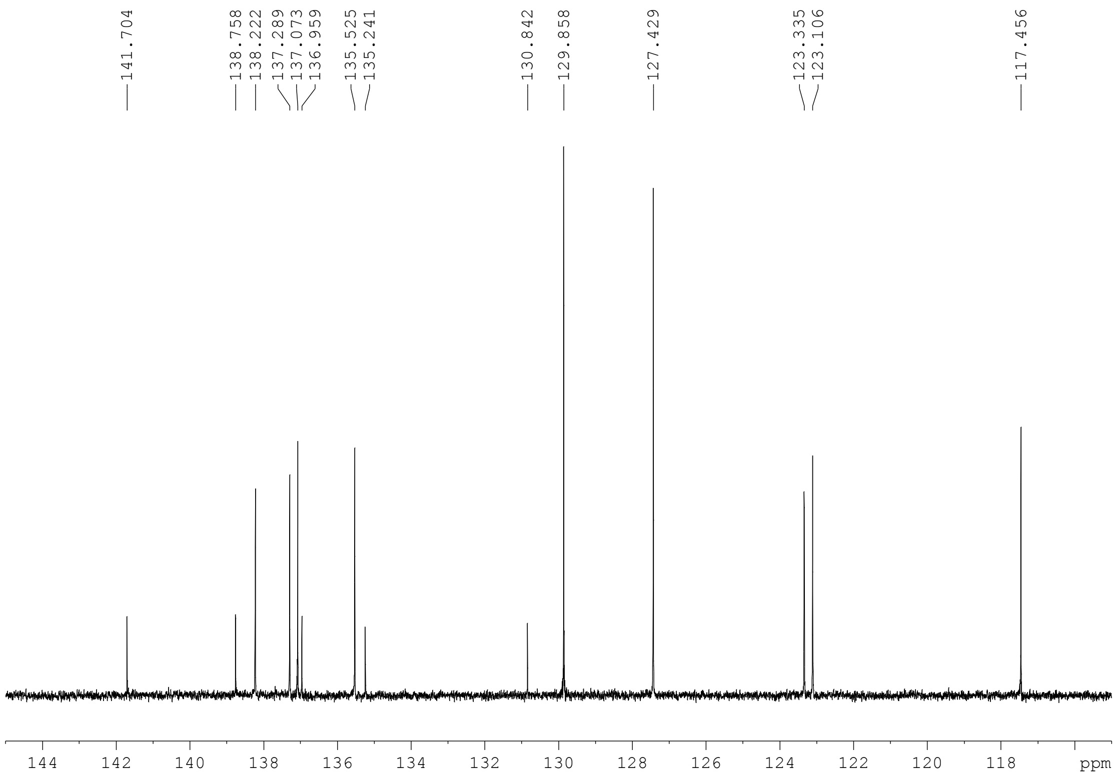

COSY

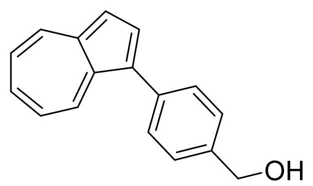

13f (500 MHz, $CDCl_3$)

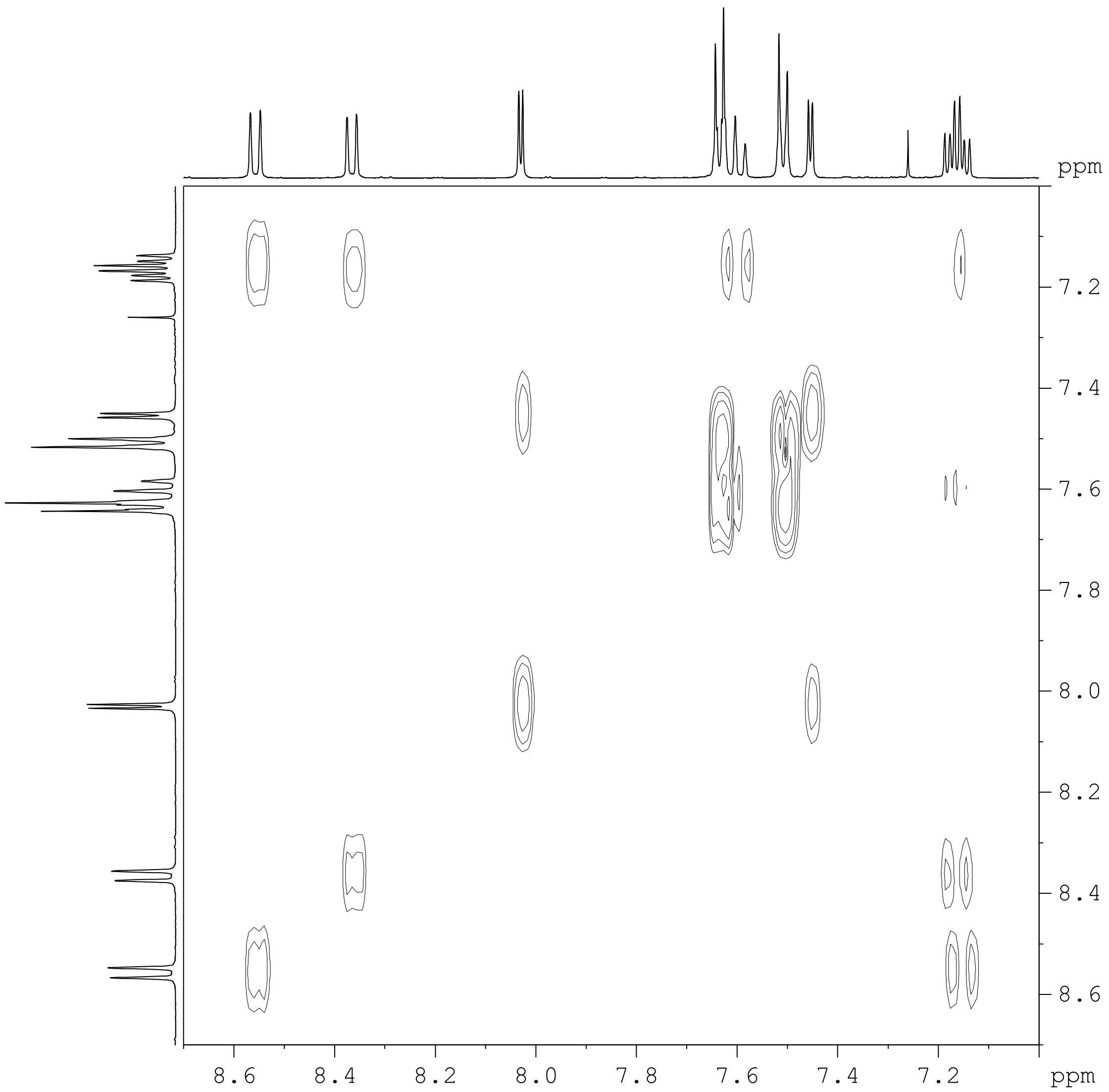

HSQC

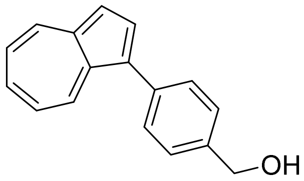

13f (500 MHz, $CDCl_3$)

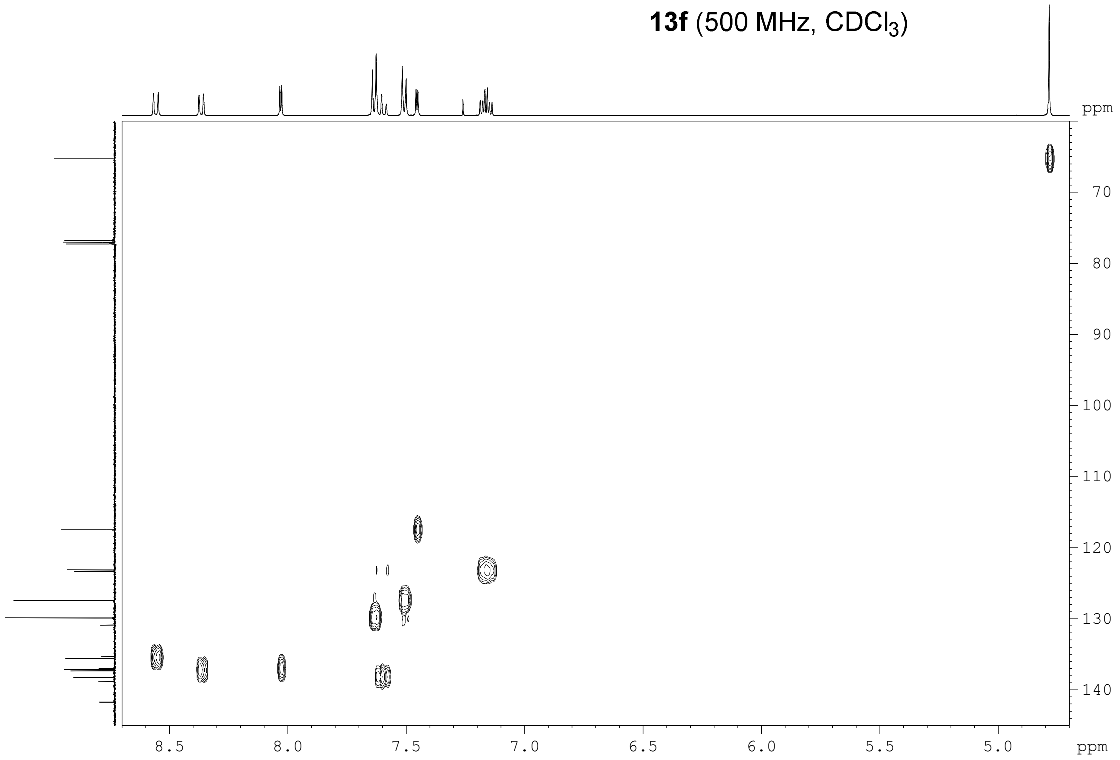

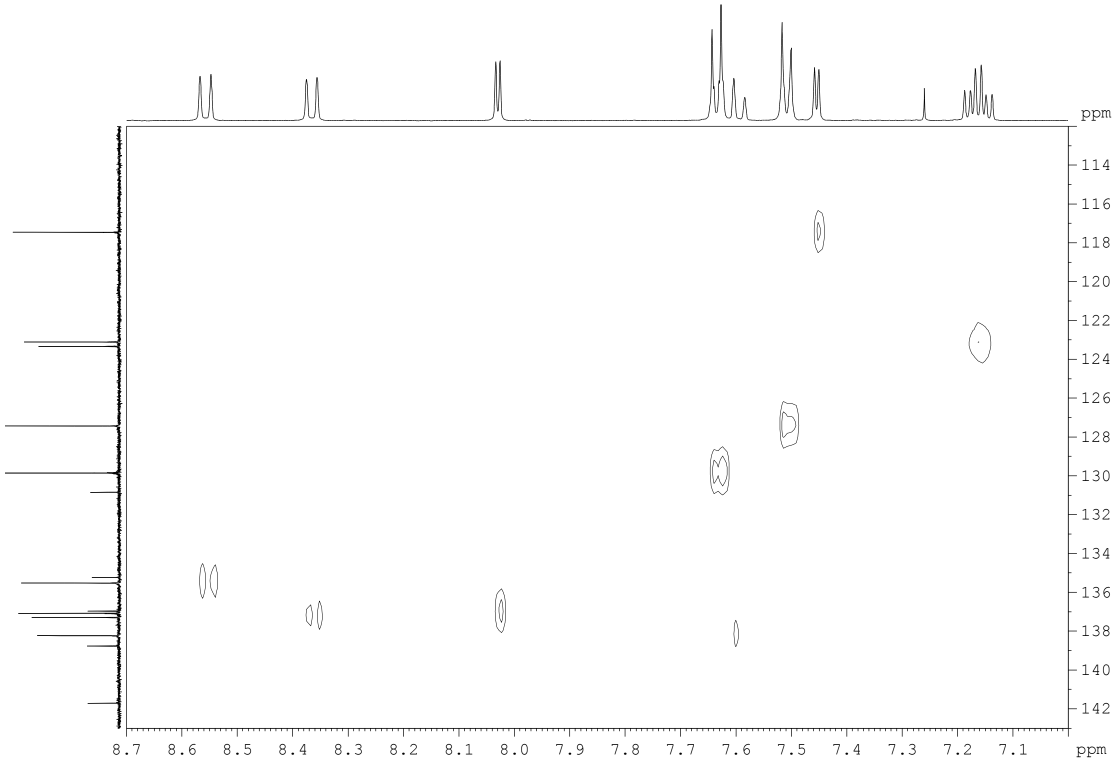

HMBC

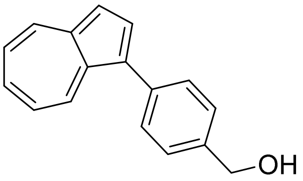

13f (500 MHz, $CDCl_3$)

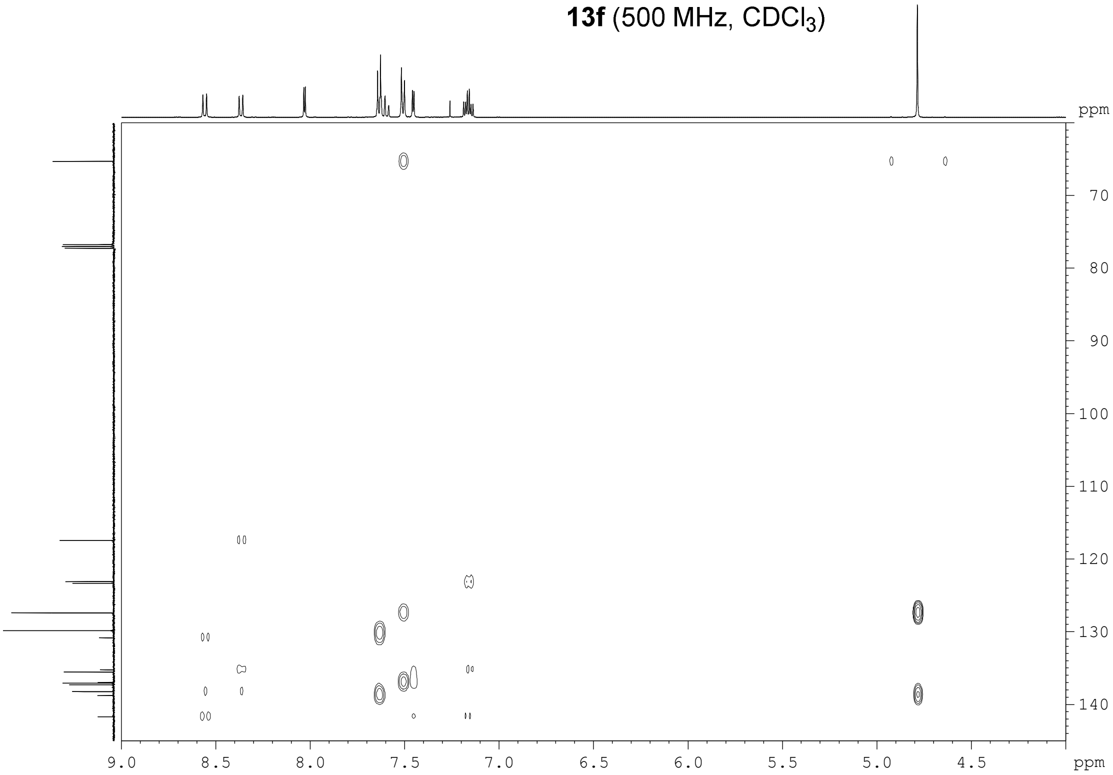

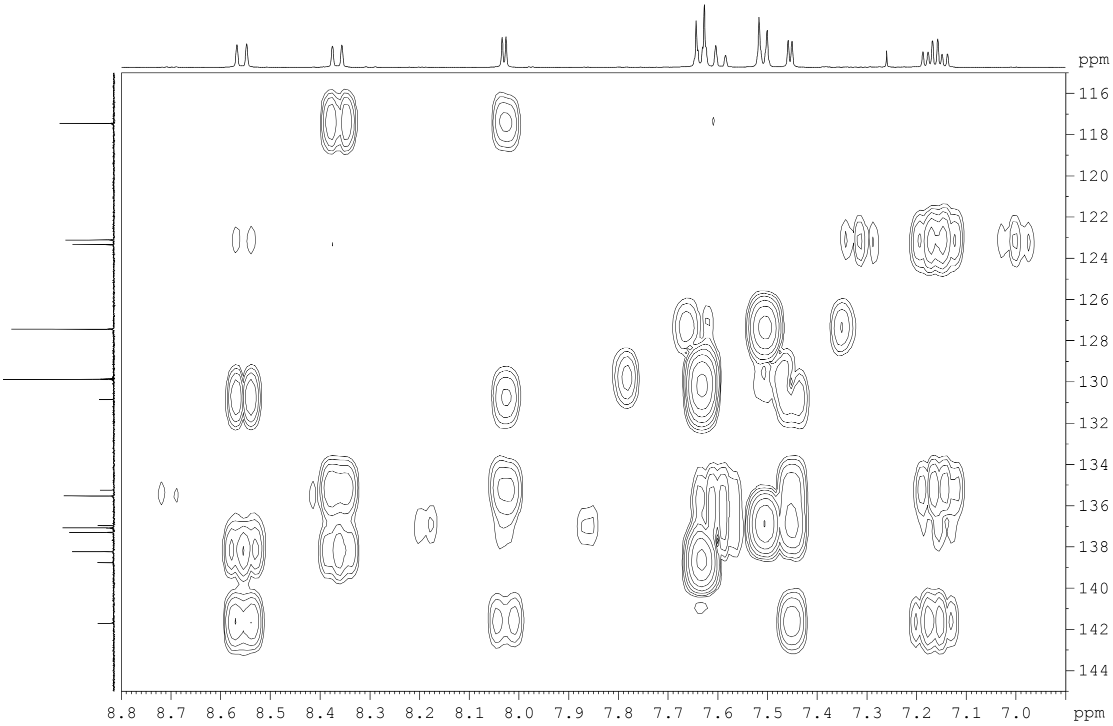

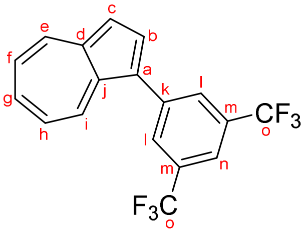

13g (500 MHz, $CDCl_3$)

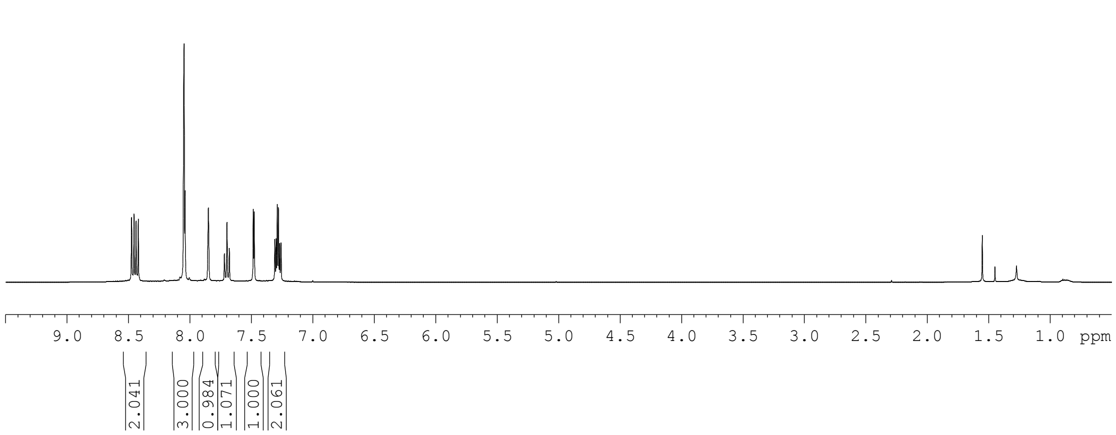

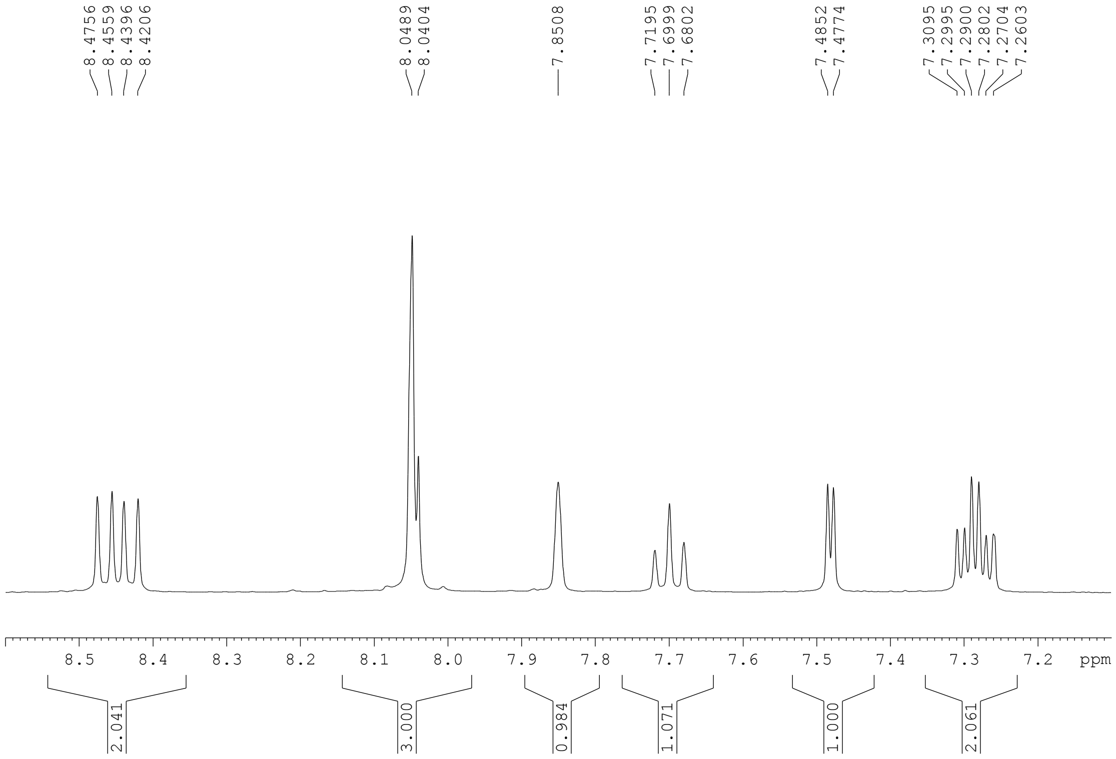

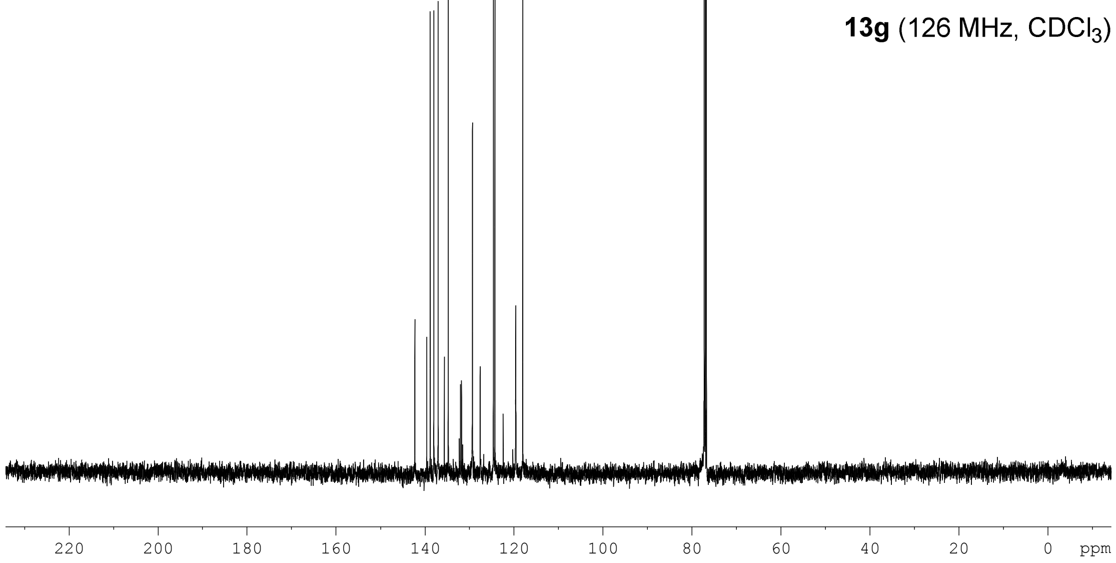

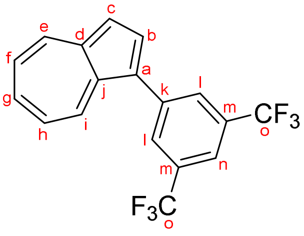

13g (126 MHz, $CDCl_3$)

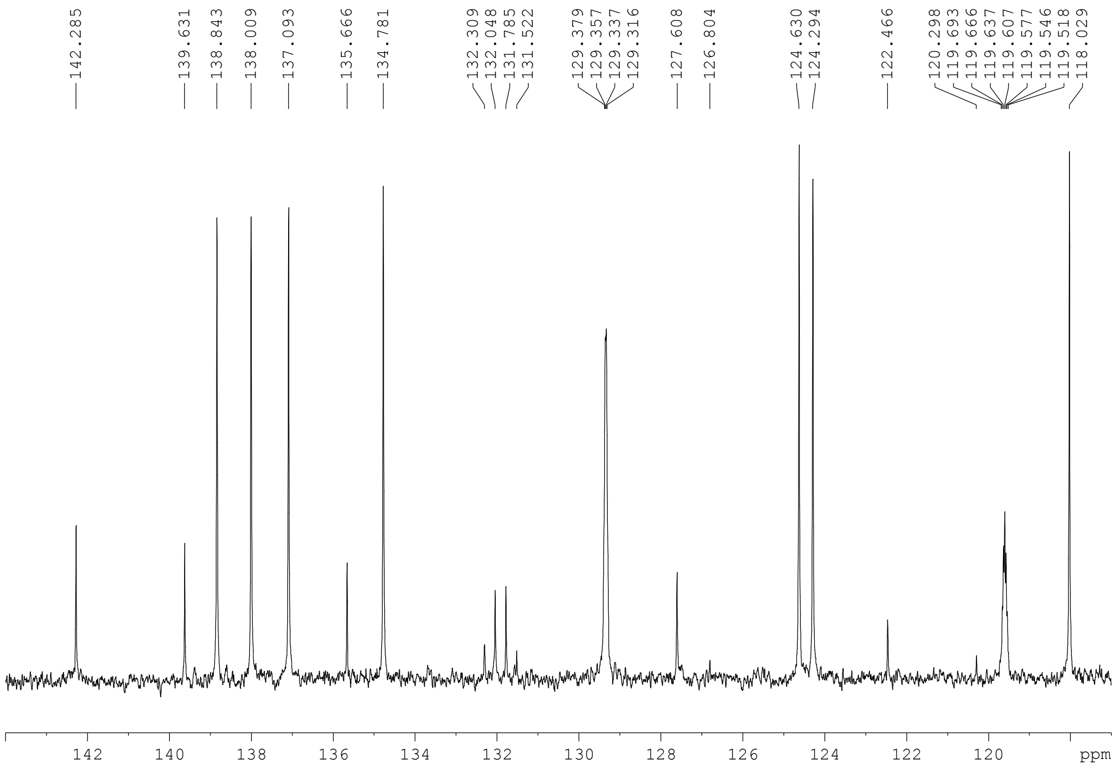

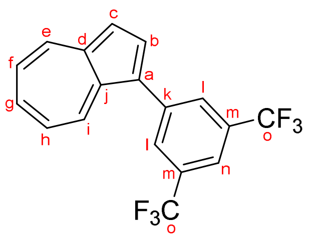

13g (126 MHz, $CDCl_3$)

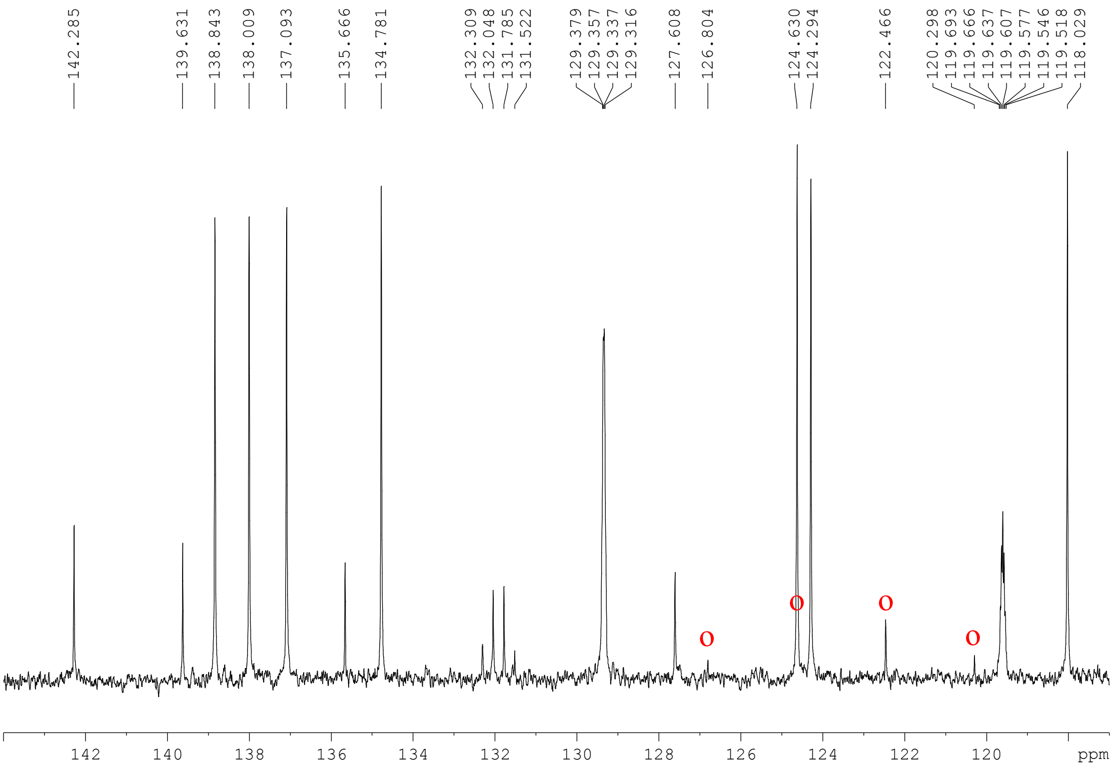

(n) 119.6, septet, $^{3}J_{CF}$ = 3.8 Hz

(o) 123.6, q, $^{1}J_{CF}$ = 272.6 Hz

(l) 129.3, q, $^{3}J_{CF}$ = 2.5 Hz

(m) 131.9, q, $^{2}J_{CF}$ = 33.1 Hz

COSY

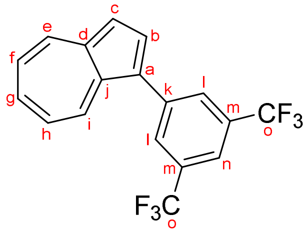

13g (500 MHz, $CDCl_3$)

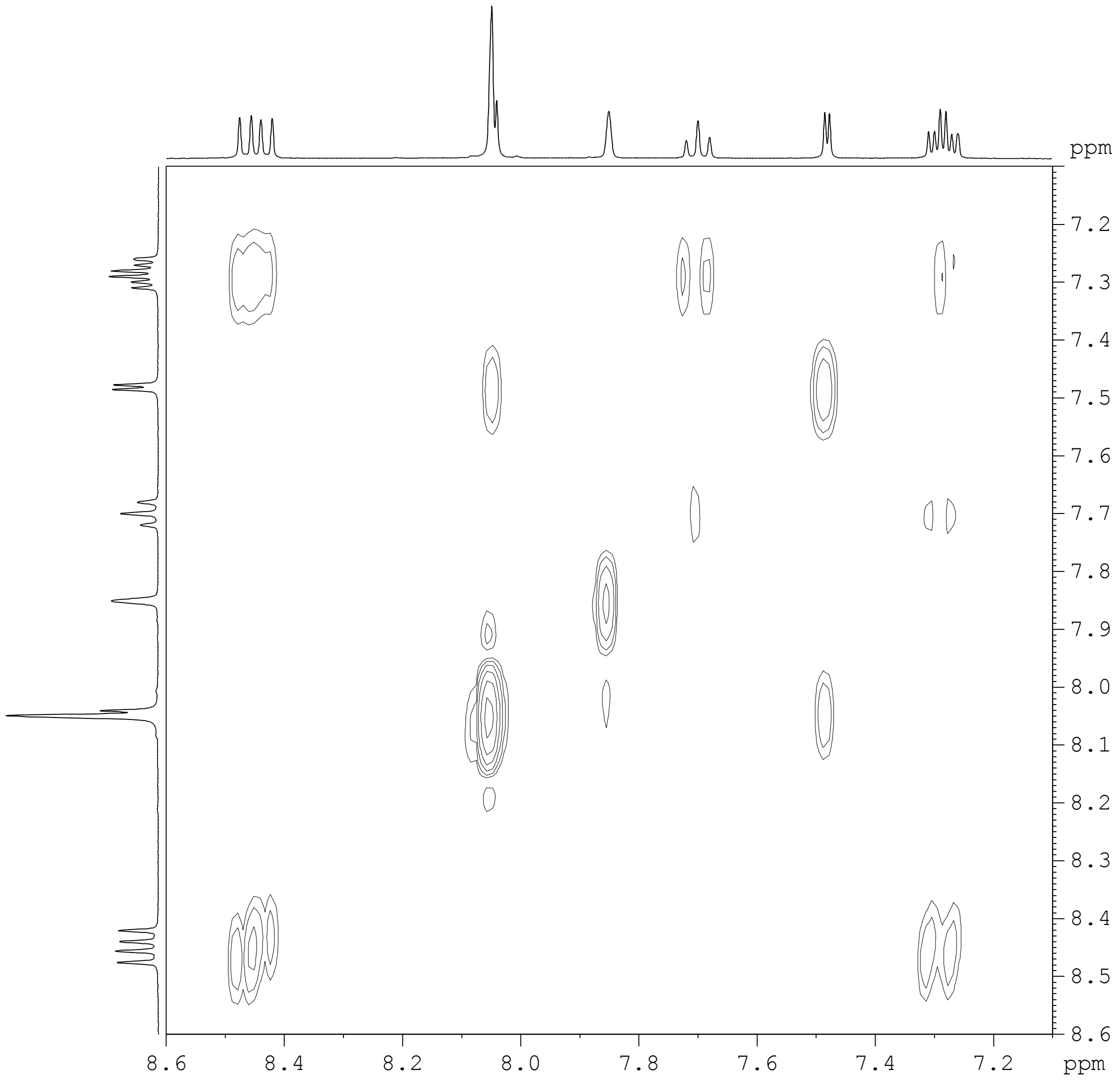

NOESY

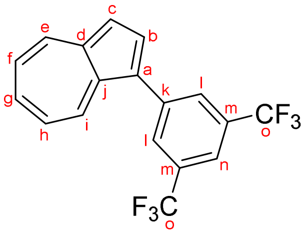

13g (500 MHz, $CDCl_3$)

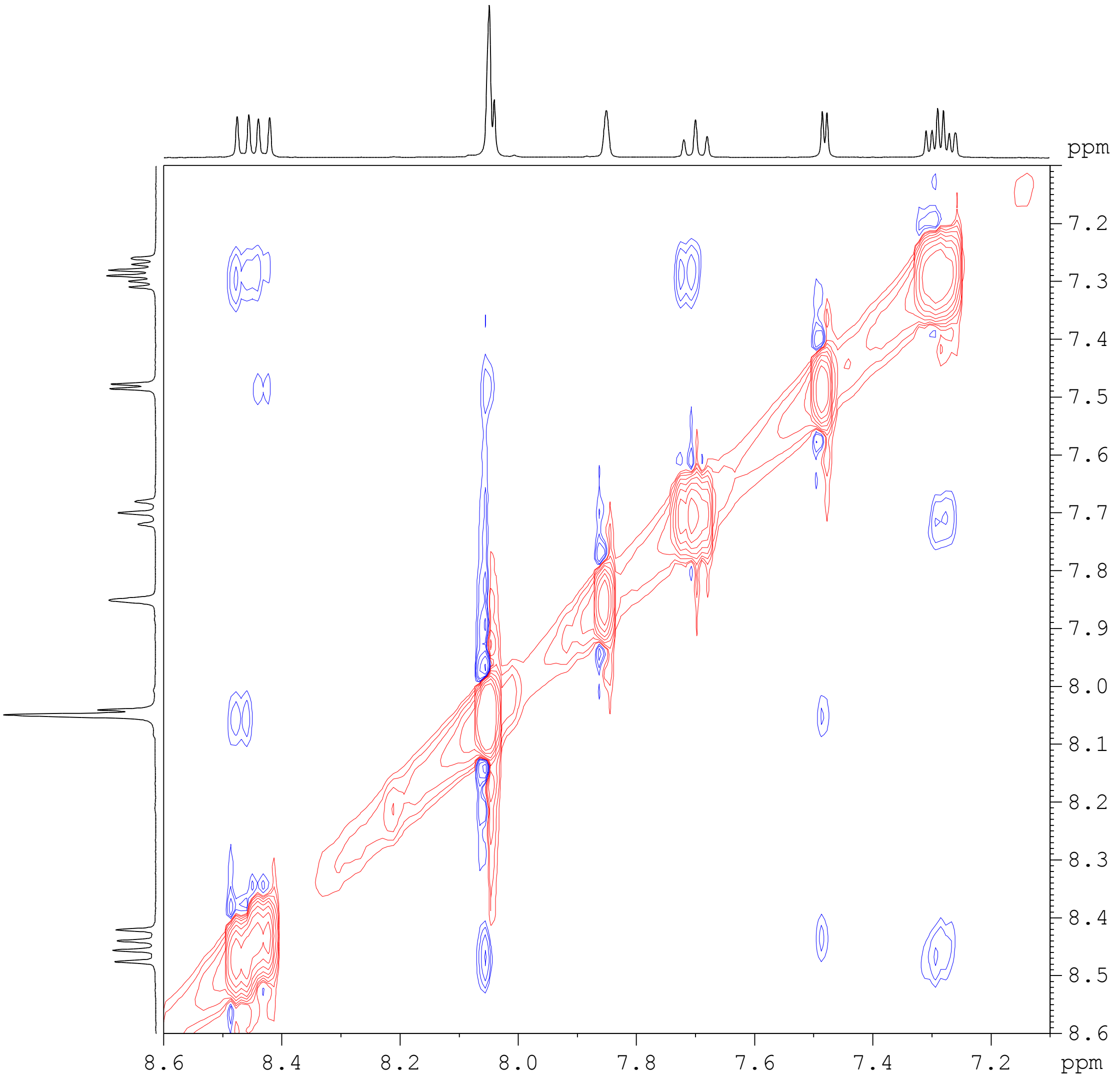

HSQC

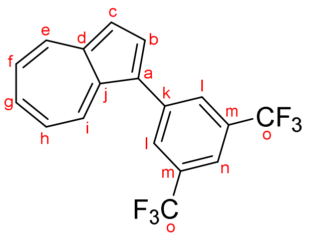

13g (500 MHz, $CDCl_3$)

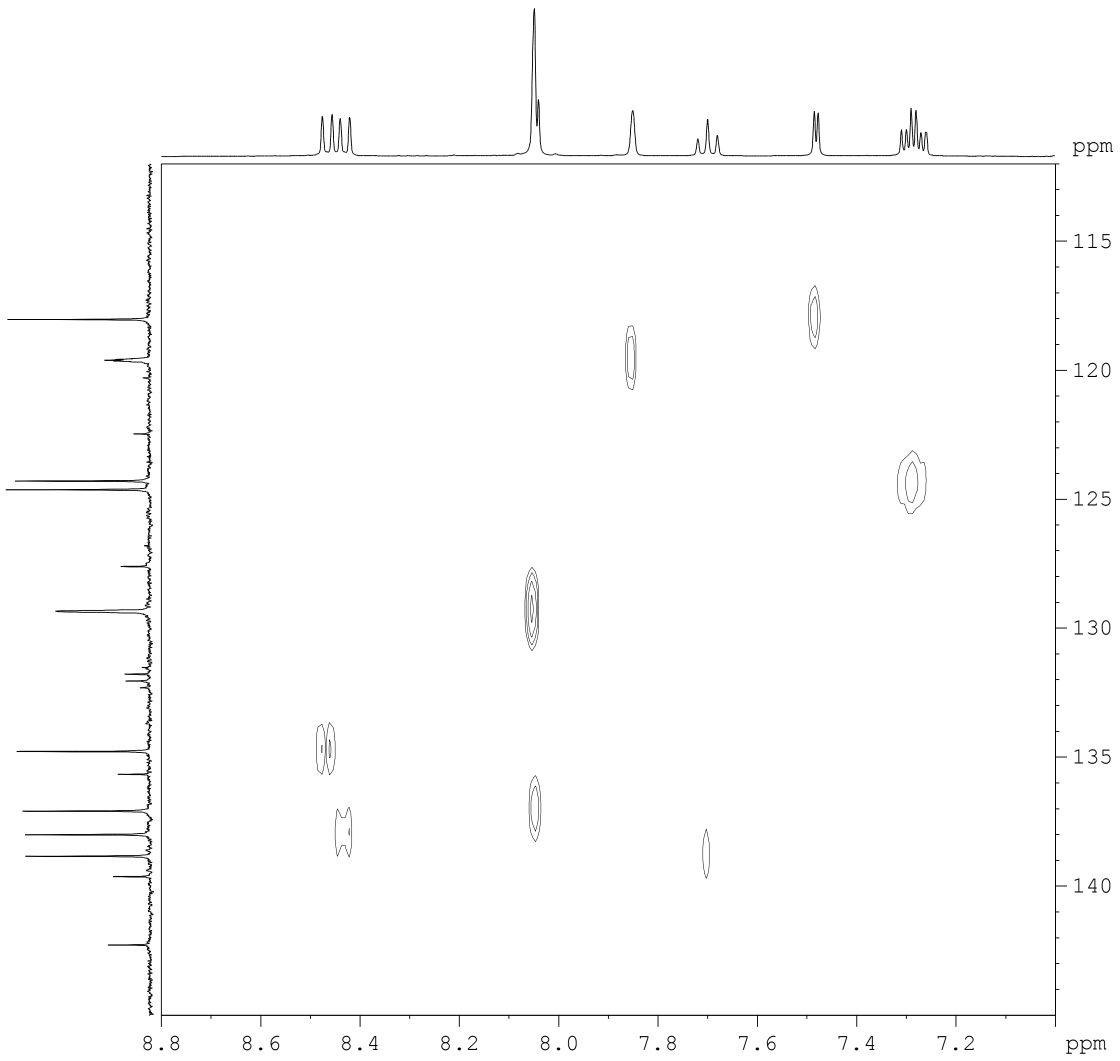

HMBC

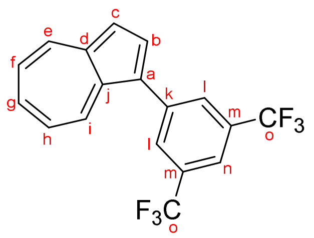

13g (500 MHz, $CDCl_3$)

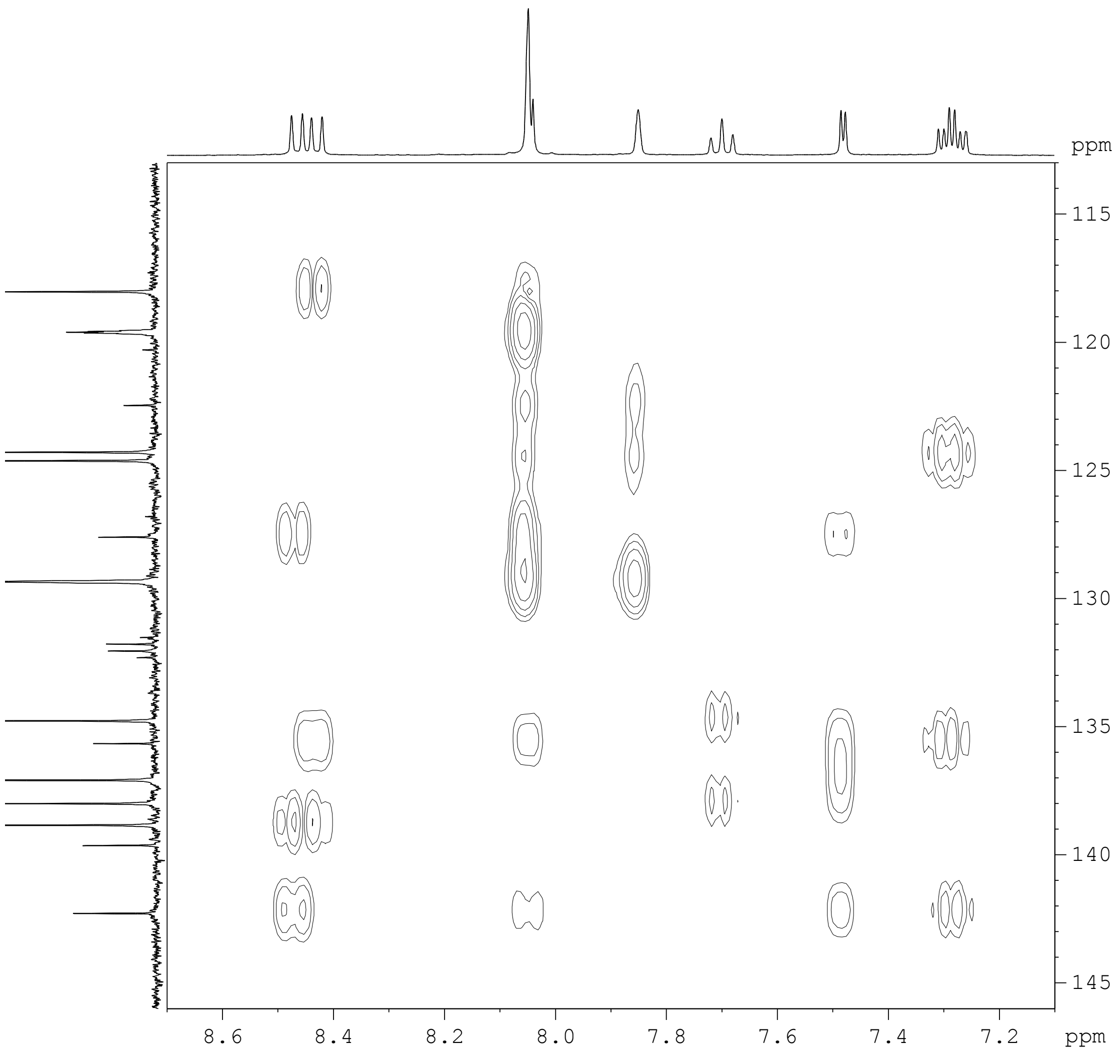

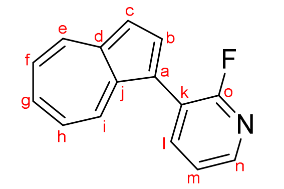

13h (500 MHz, $CDCl_3$)

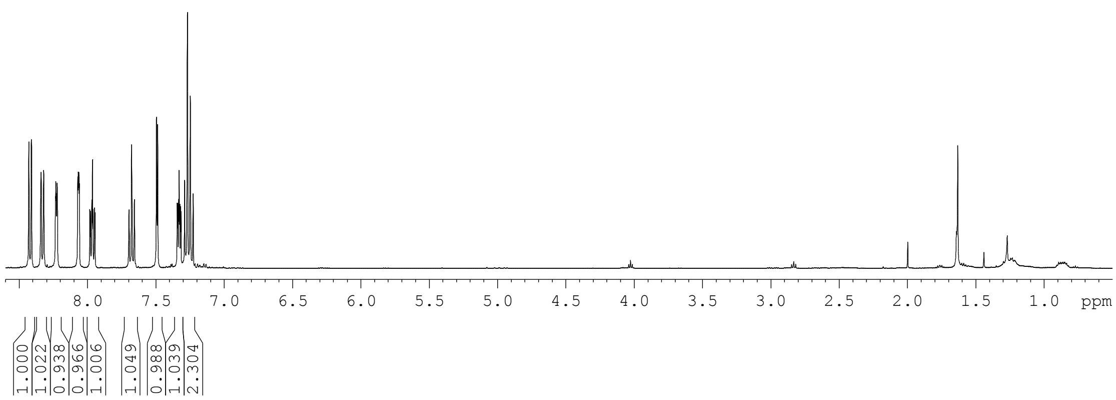

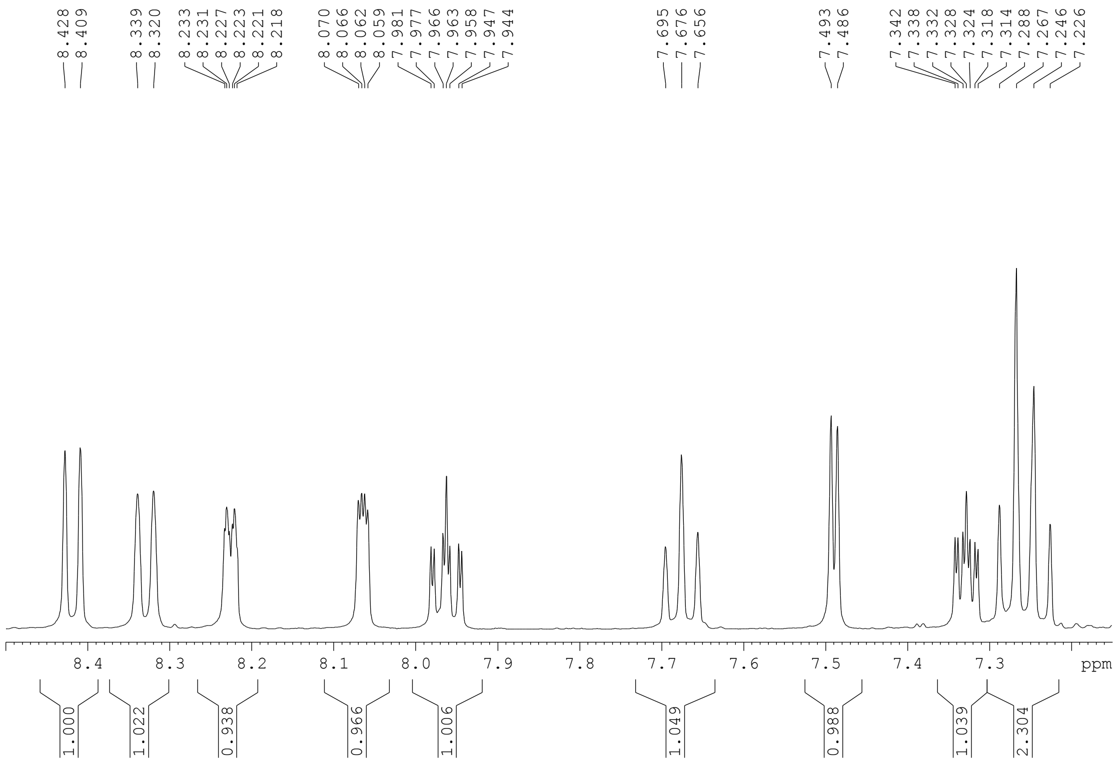

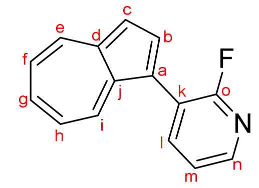

13h (126 MHz, $CDCl_3$)

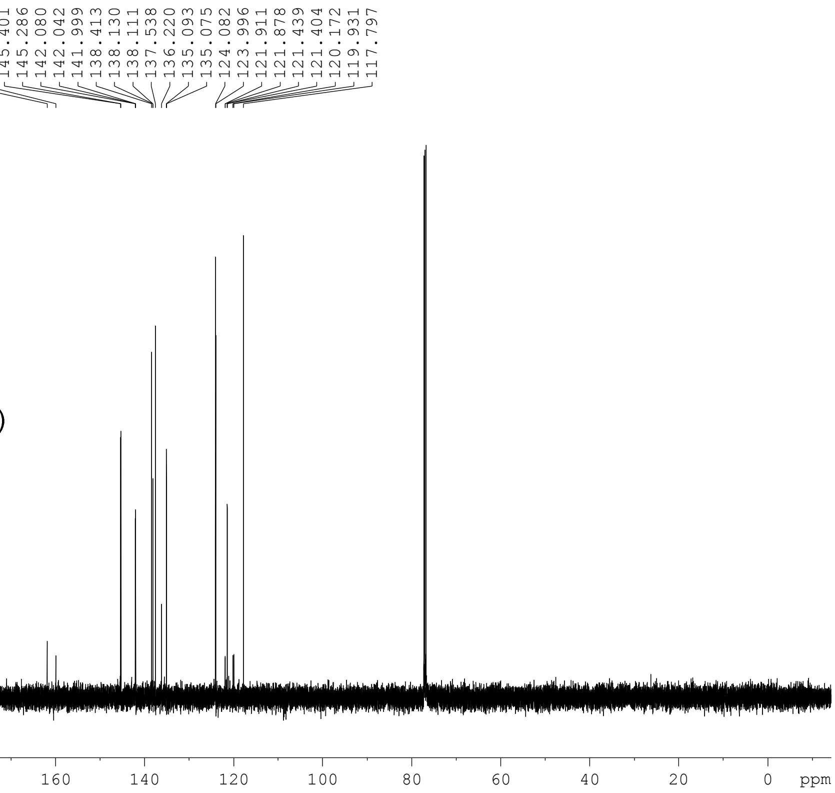

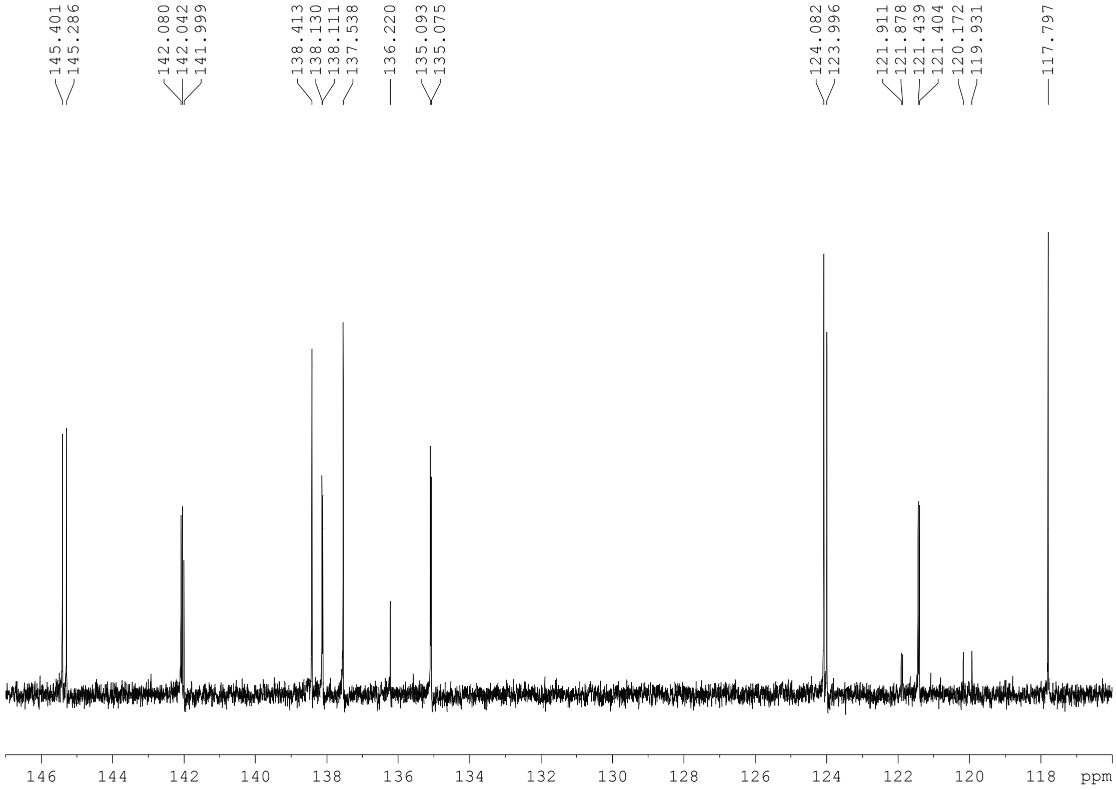

COSY

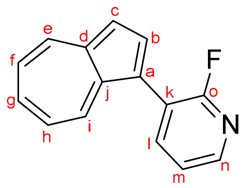

13h (500 MHz, $CDCl_3$)

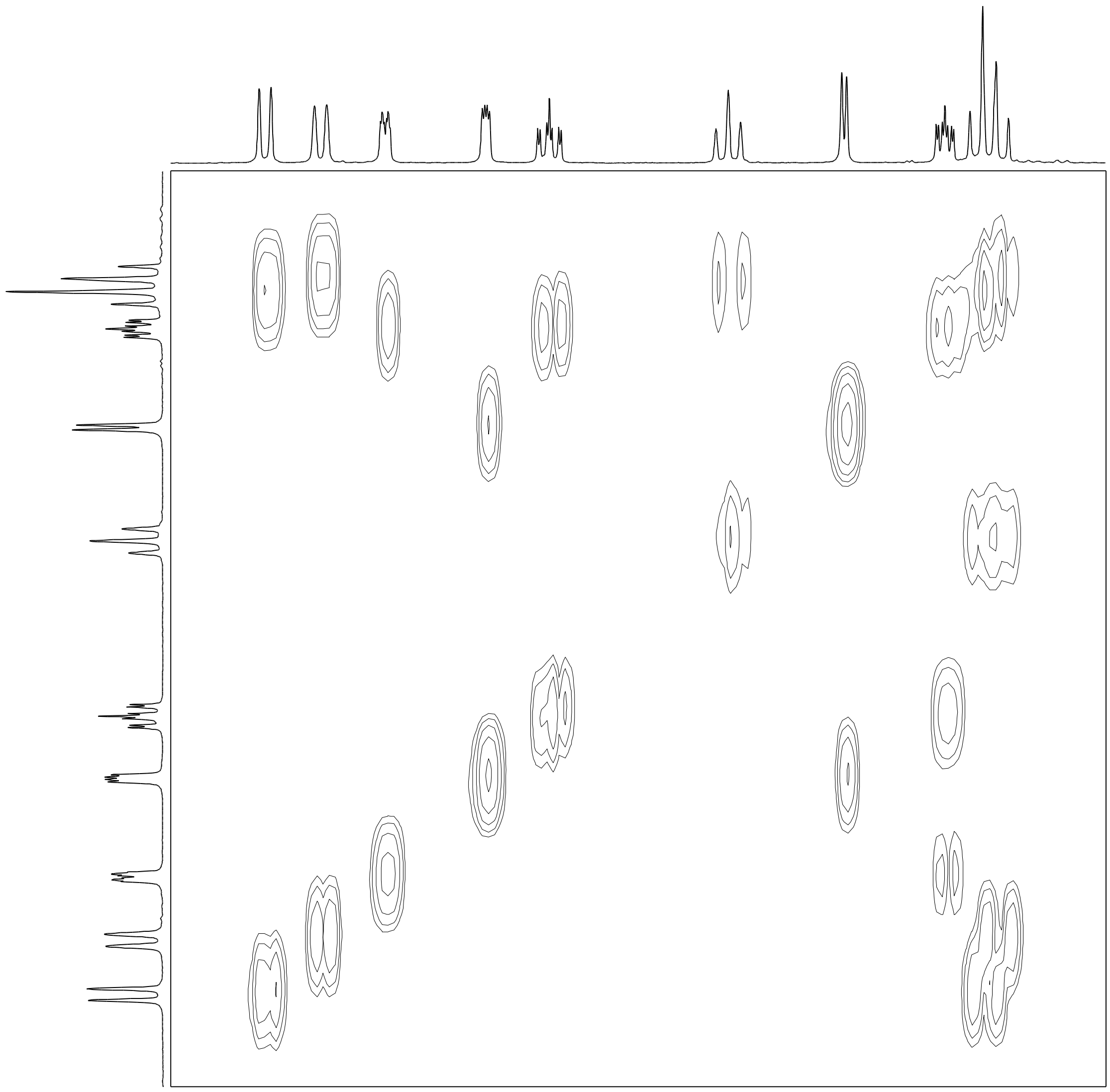

NOESY

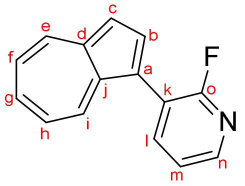

13h (500 MHz, $CDCl_3$)

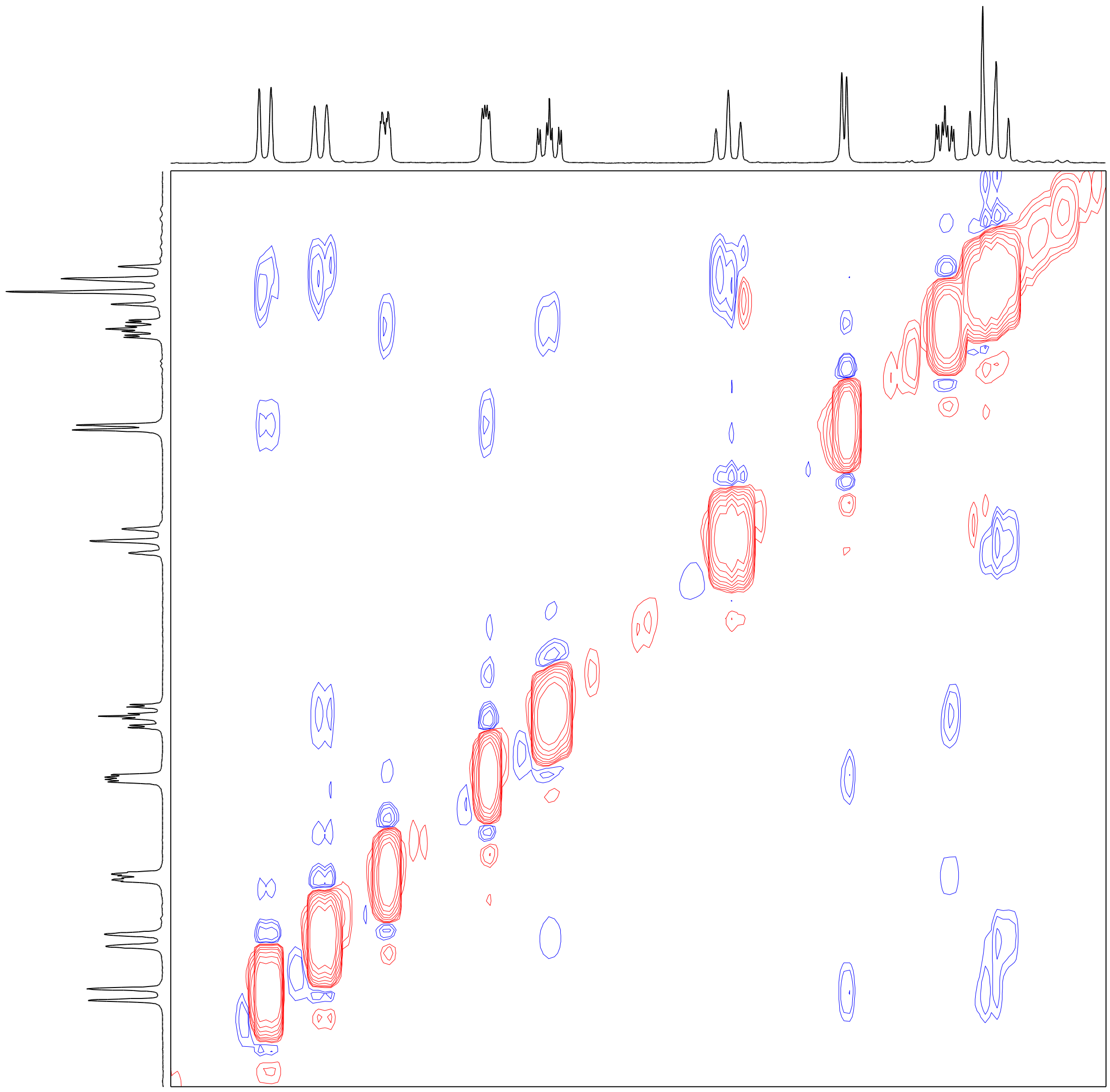

HSQC

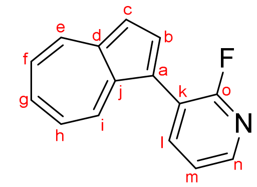

13h (500 MHz, $CDCl_3$)

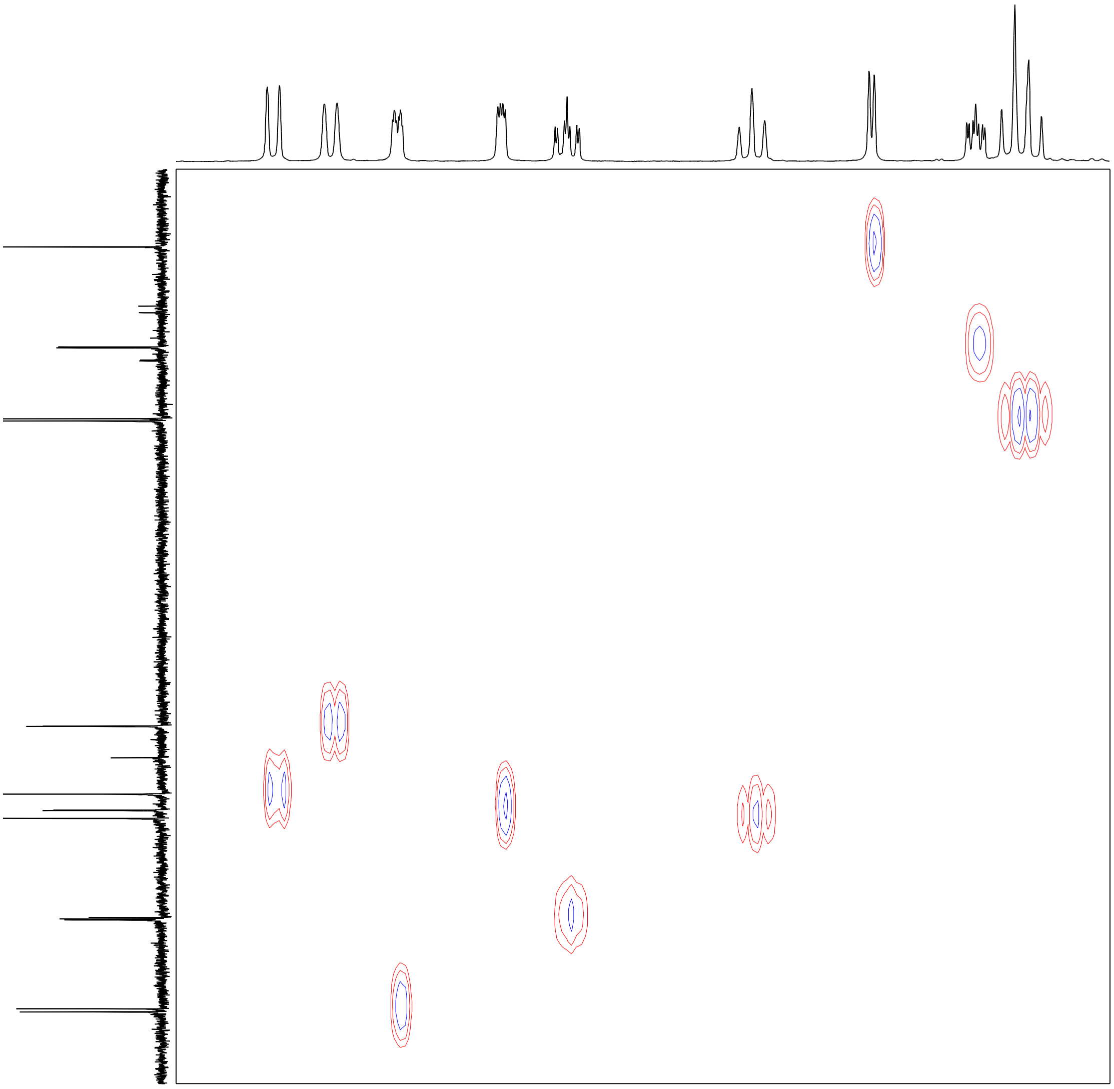

HMBC

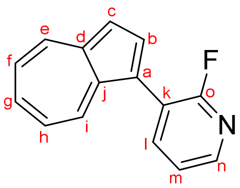

13h (500 MHz, $CDCl_3$)

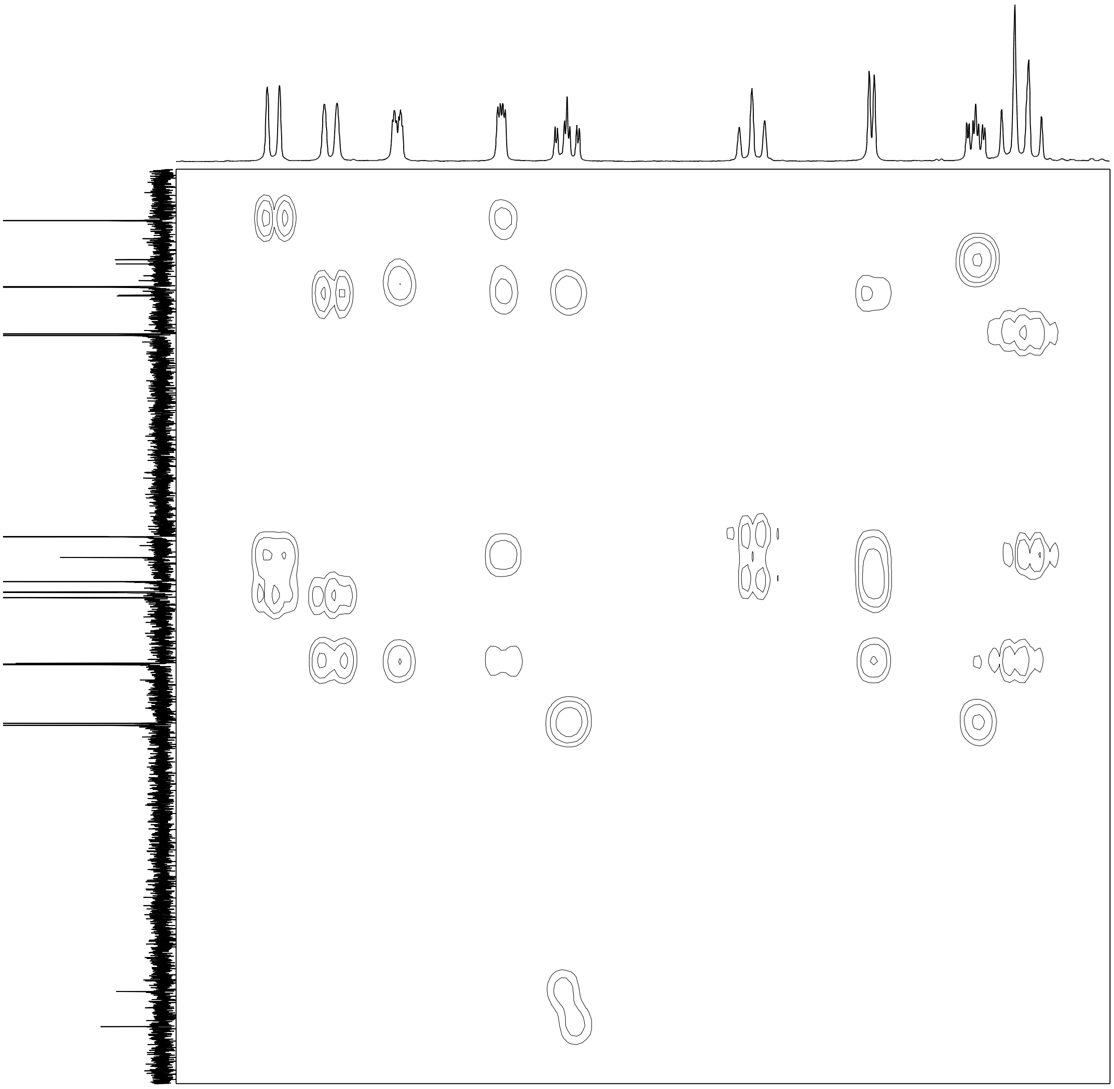

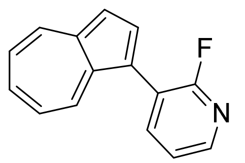

13h (^{19}F NMR, 470.5 MHz, $CDCl_3$)

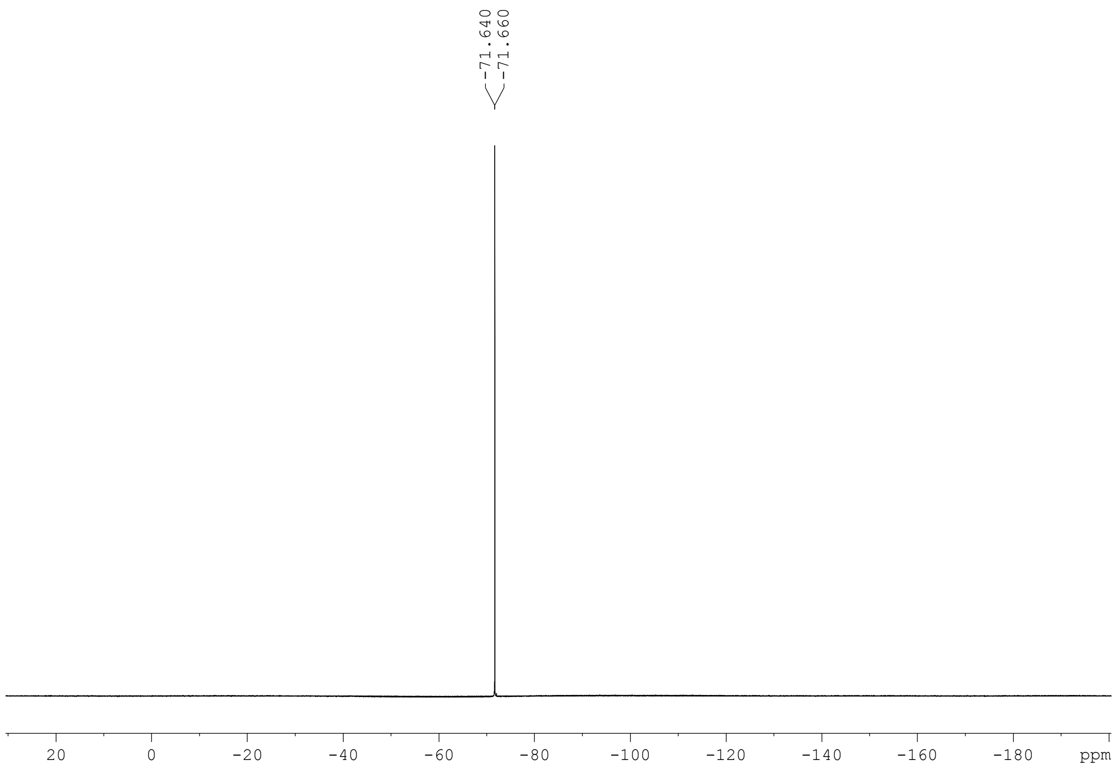

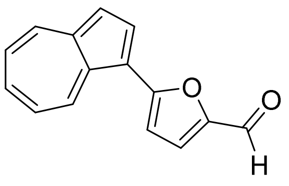

13i (500 MHz, $CDCl_3$)

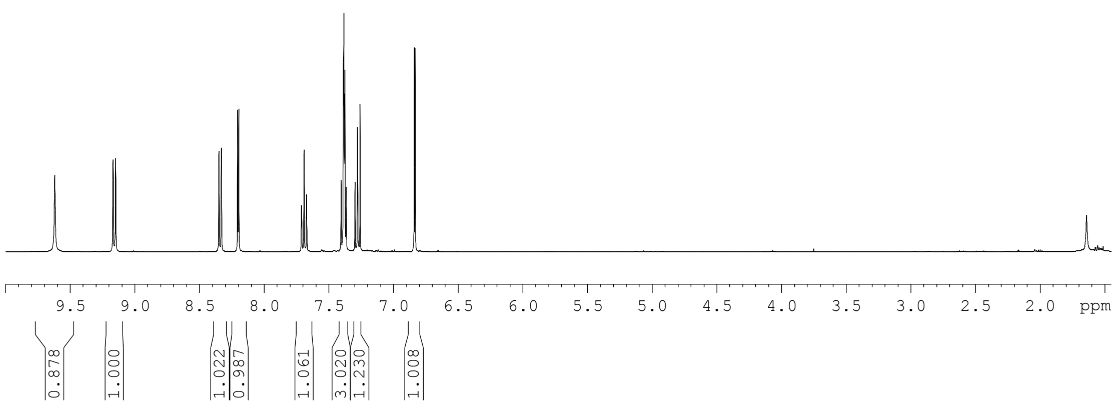

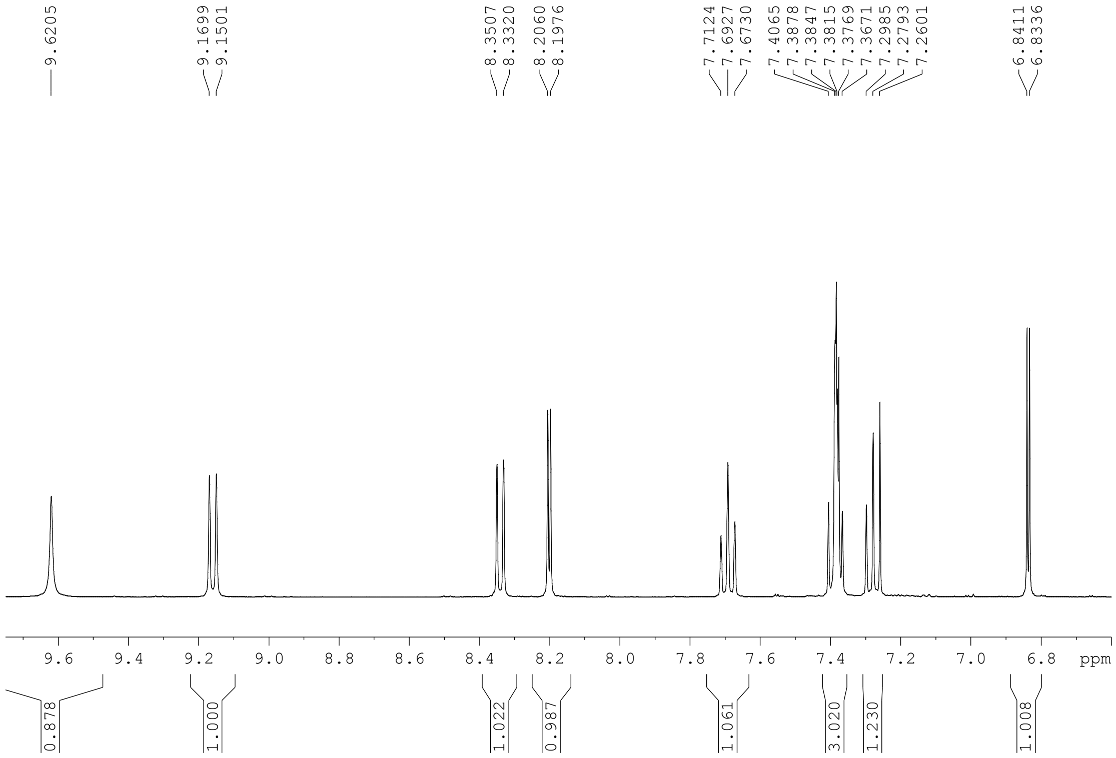

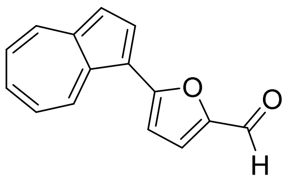

13i (126 MHz, $CDCl_3$)

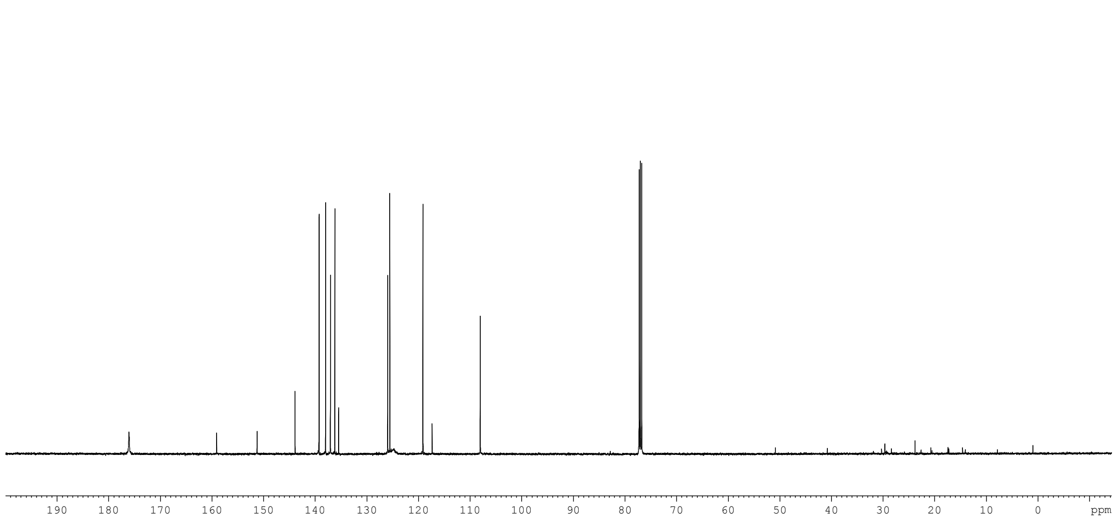

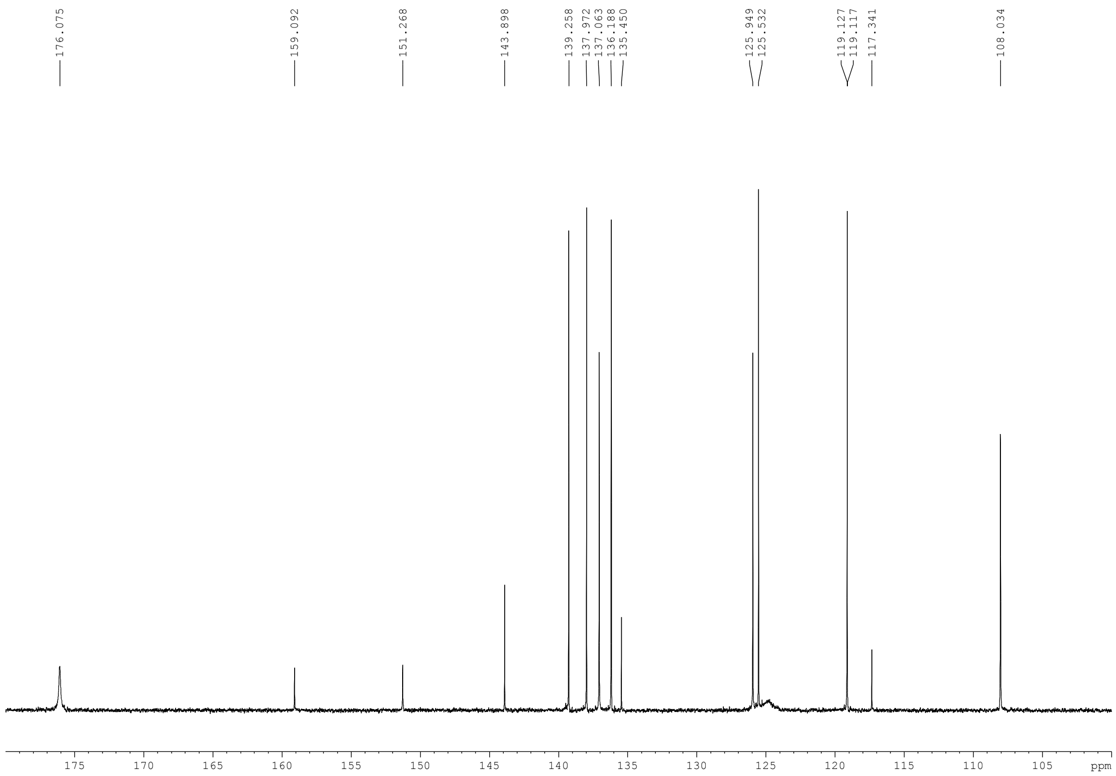

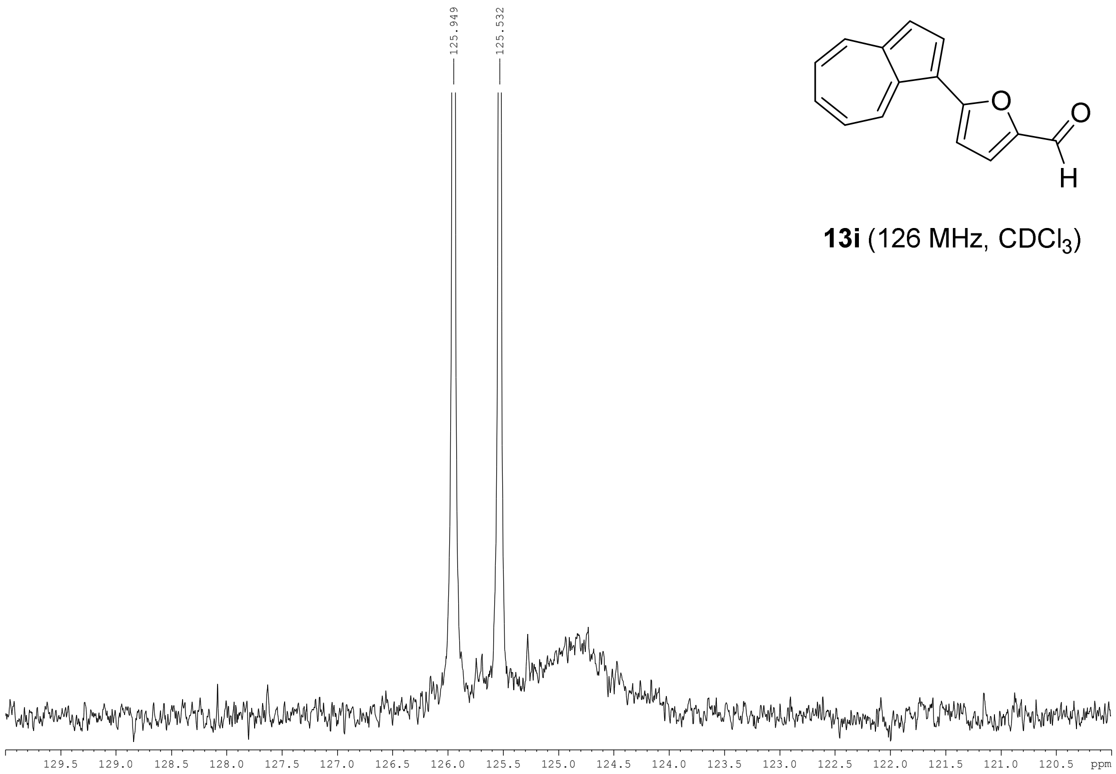

13i (126 MHz, $CDCl_3$)

[Line broadening = 1]

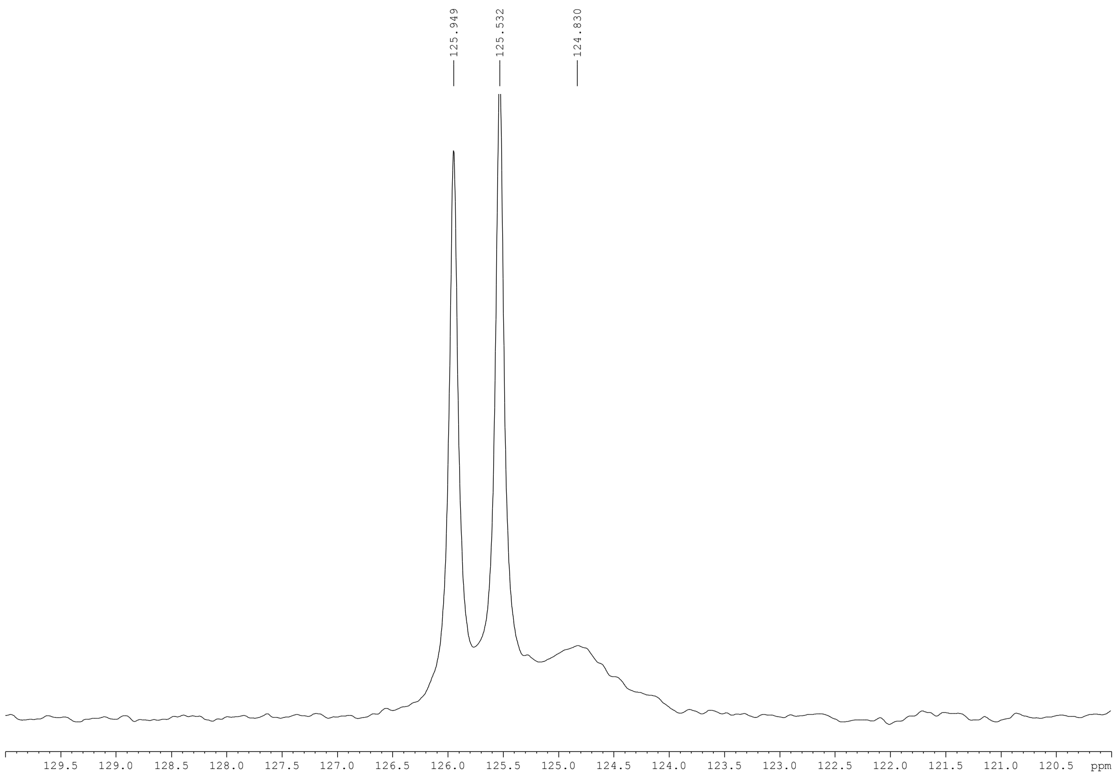

[Line broadening = 10]

COSY

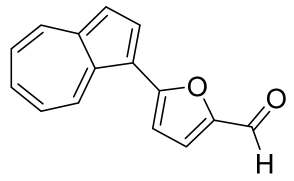

13i (500 MHz, $CDCl_3$)

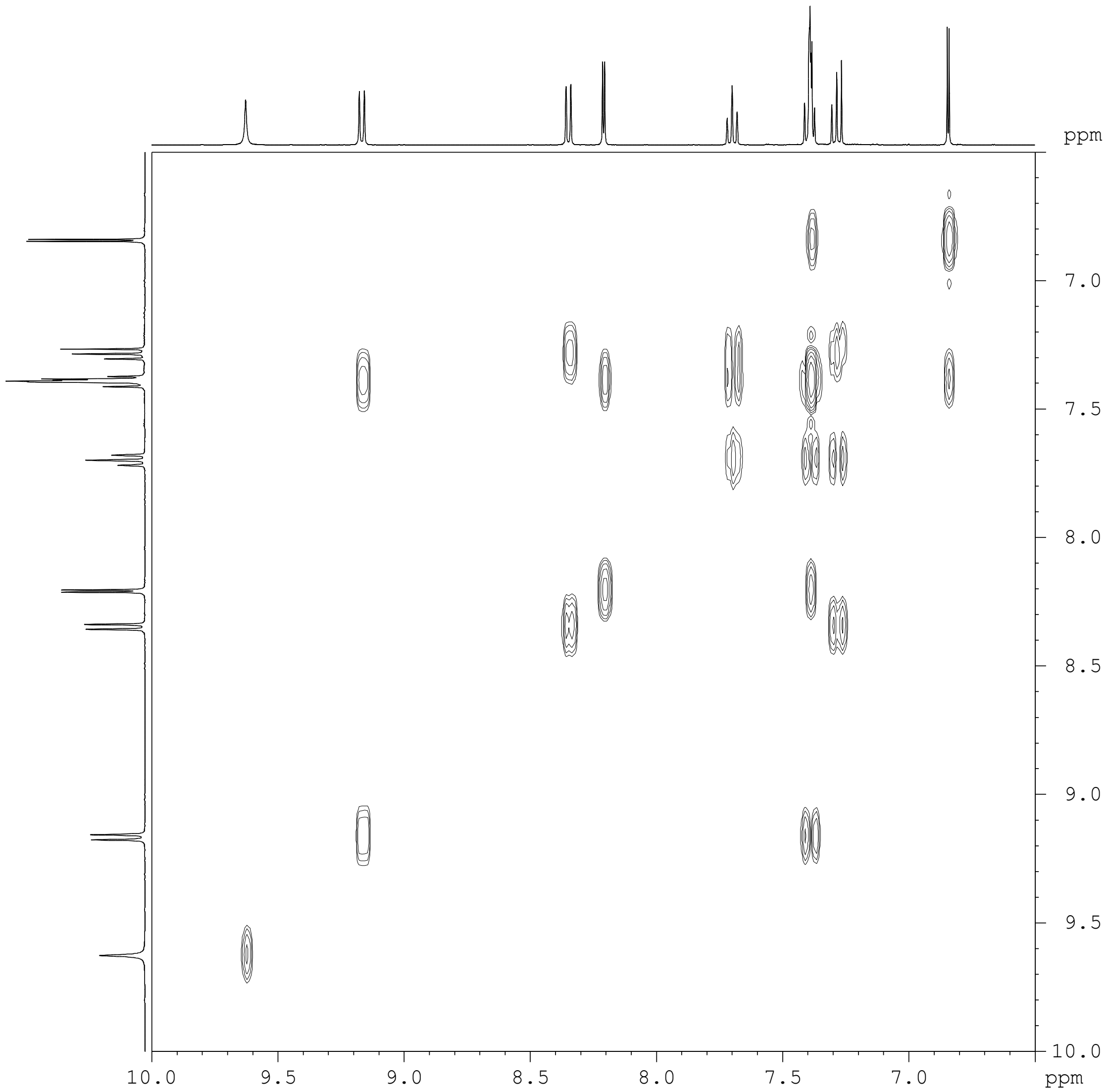

NOESY

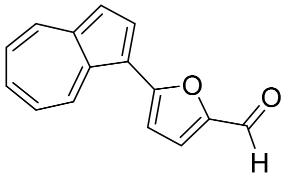

13i (500 MHz, $CDCl_3$)

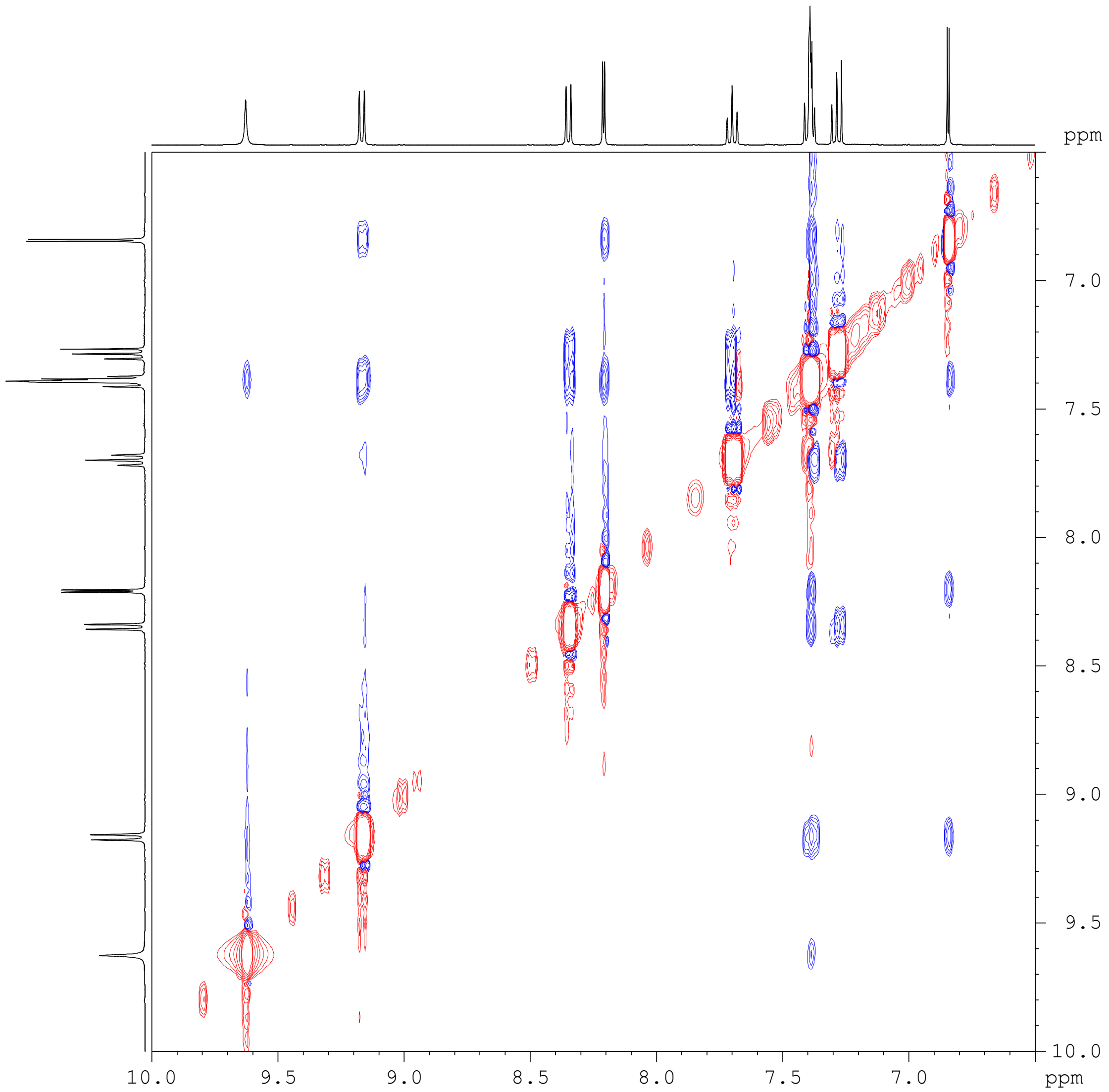

HSQC

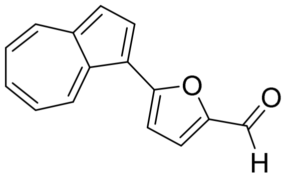

13i (500 MHz, $CDCl_3$)

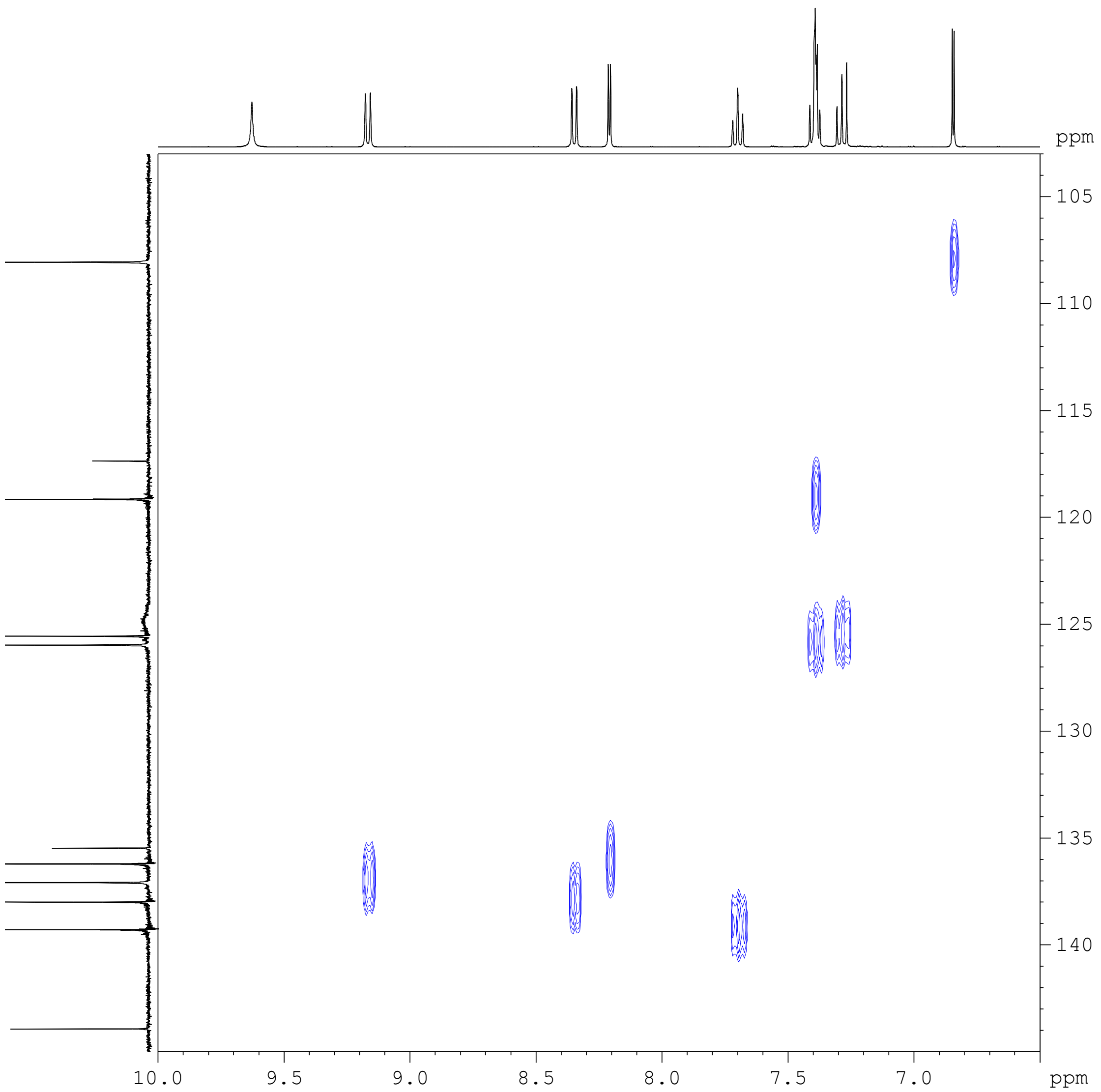

HMBC

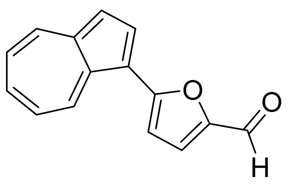

13i (500 MHz, $CDCl_3$)

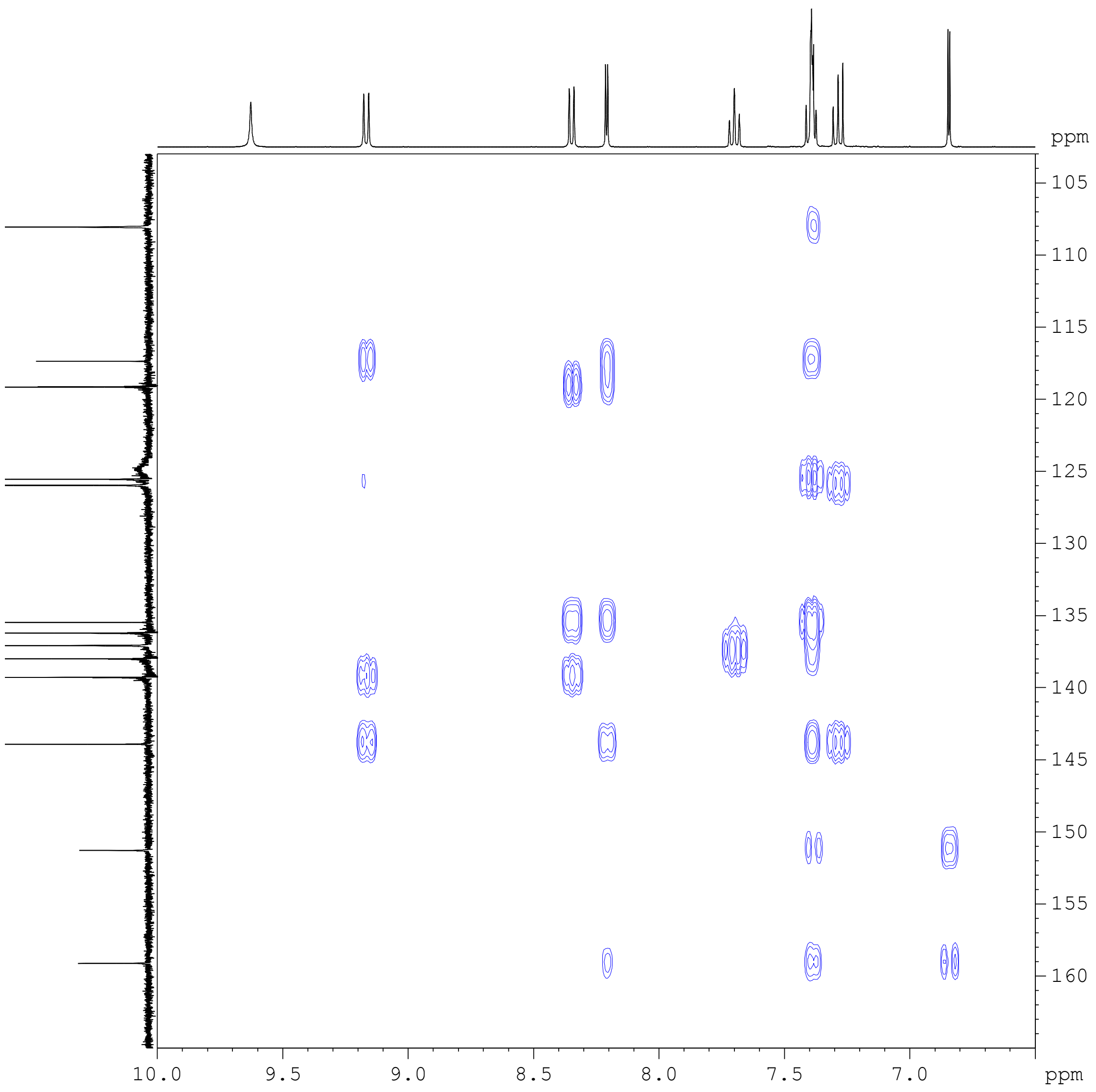

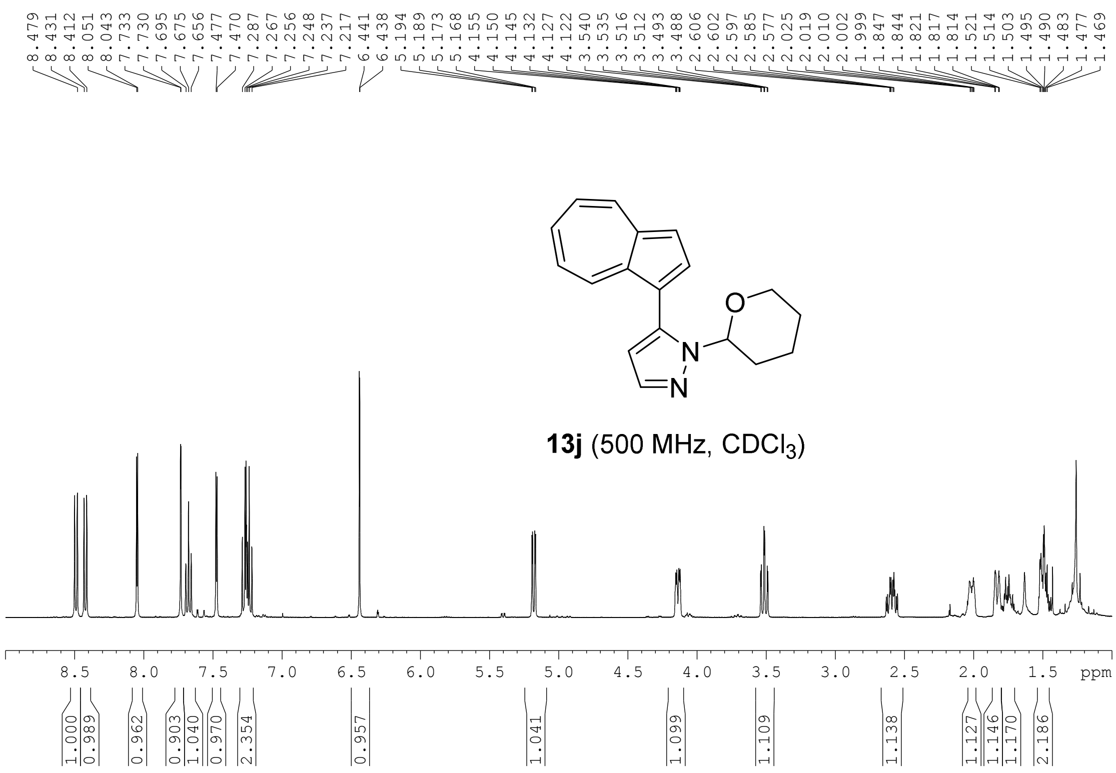

13j (500 MHz, $CDCl_3$)

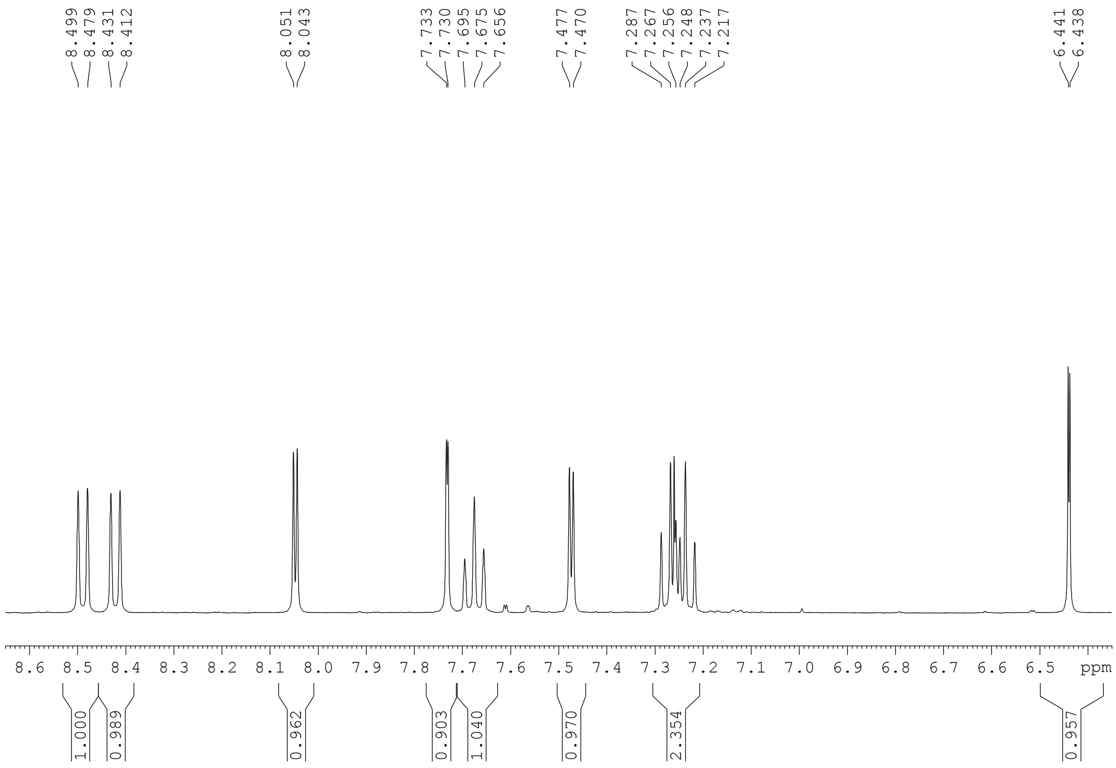

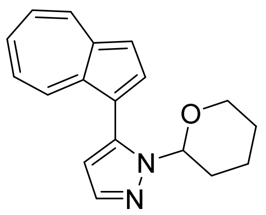

13j (500 MHz, $CDCl_3$)

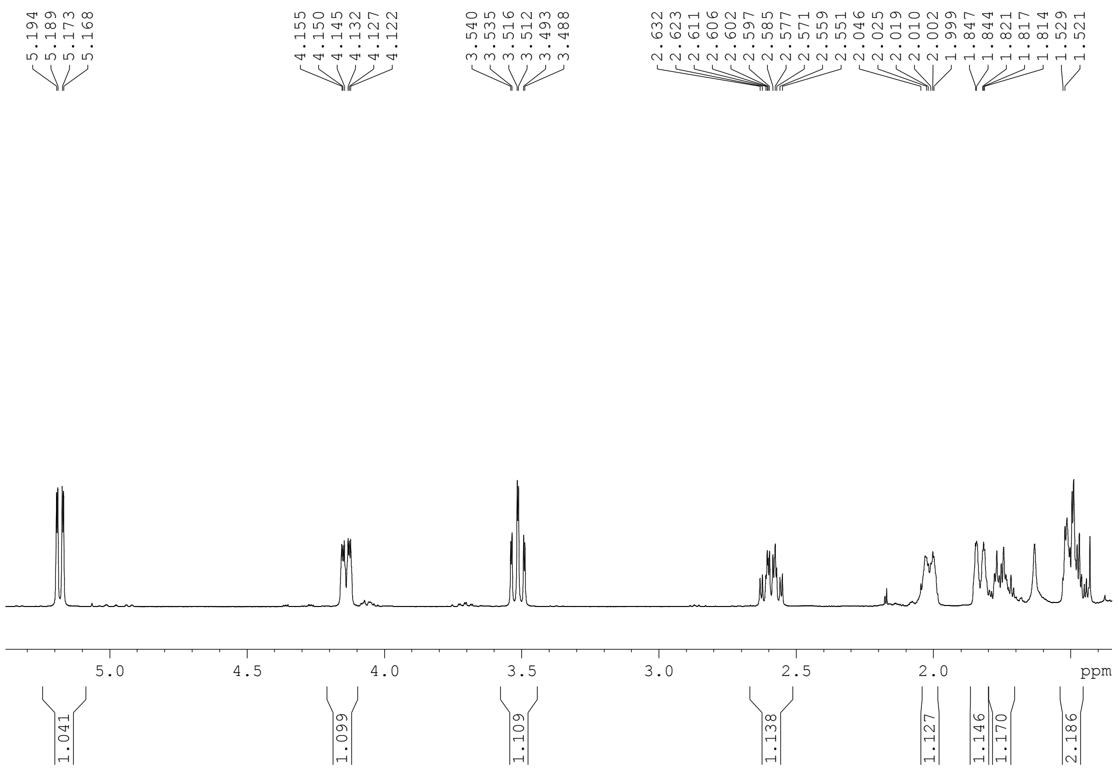

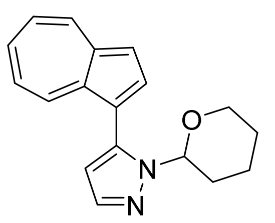

13j (126 MHz, $CDCl_3$)

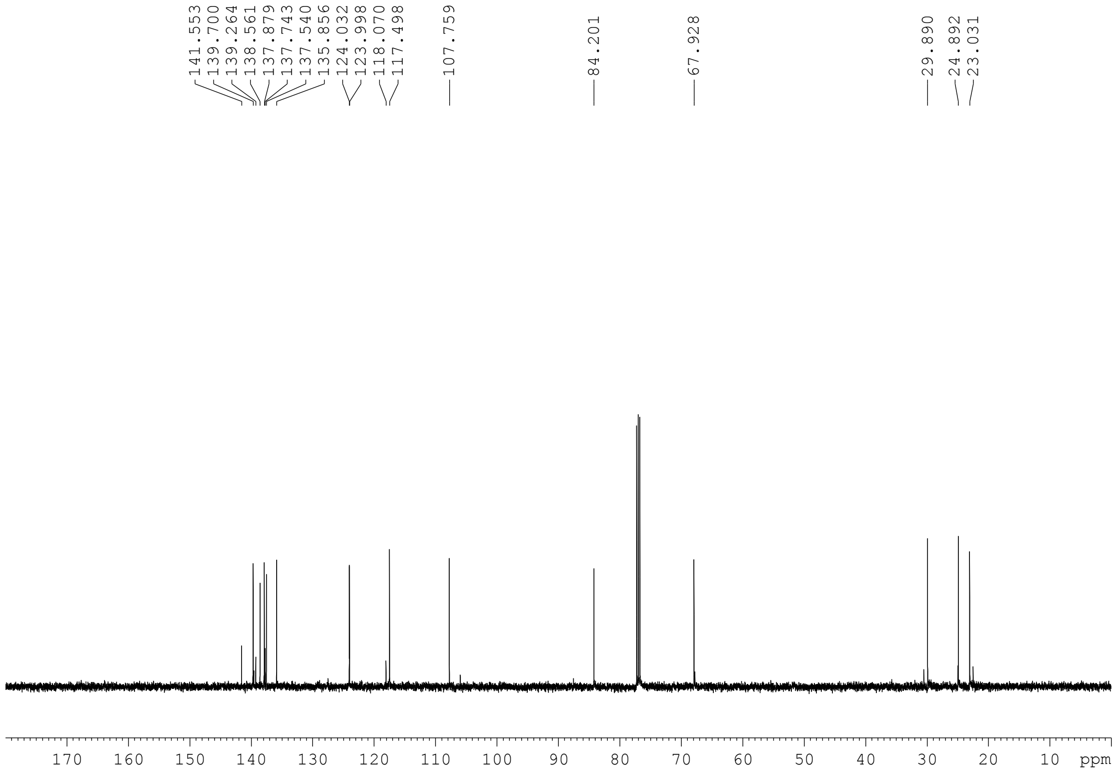

COSY

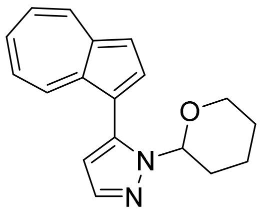

13j (500 MHz, $CDCl_3$)

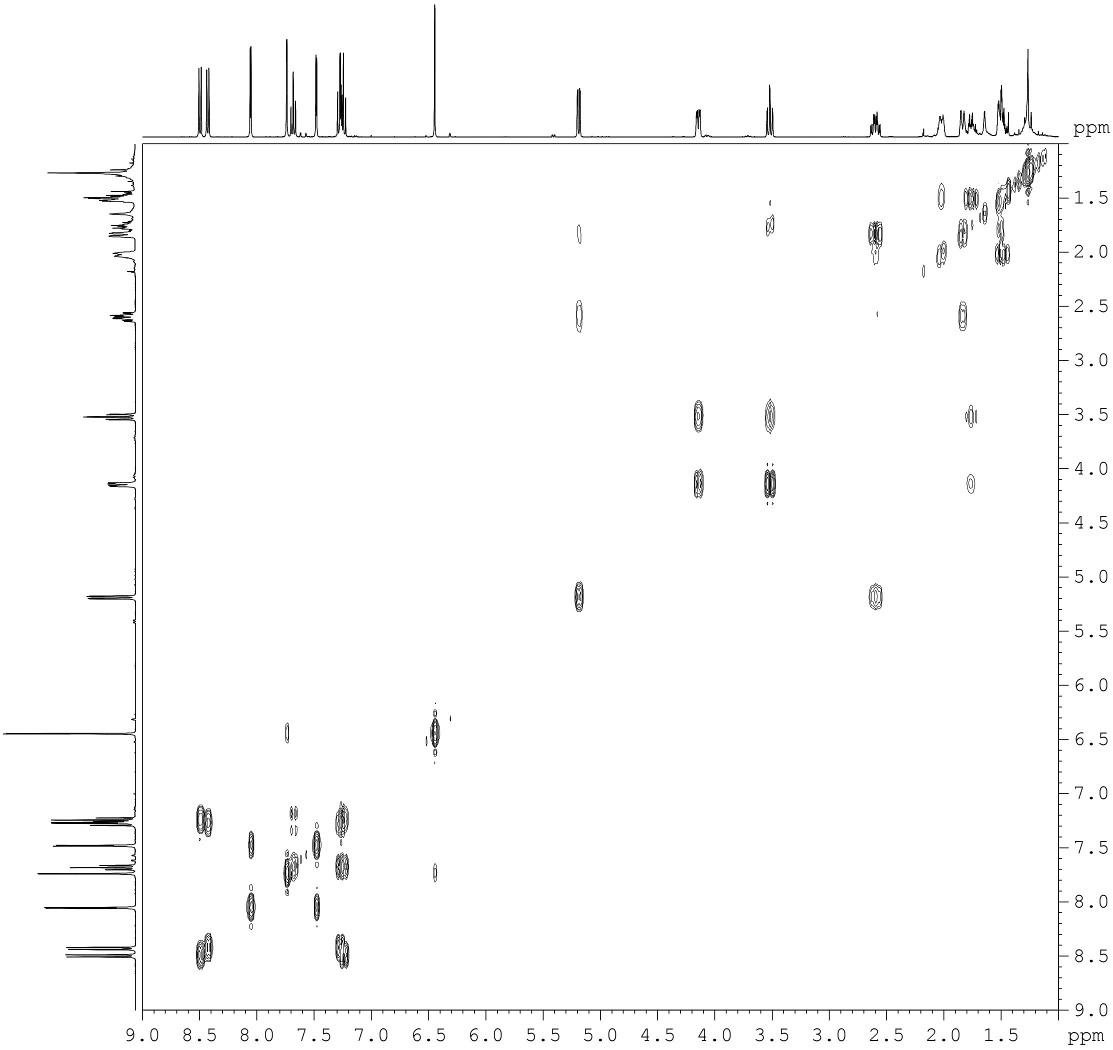

NOESY

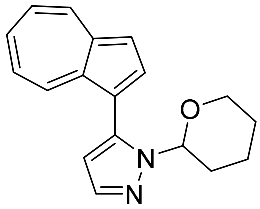

13j (500 MHz, $CDCl_3$)

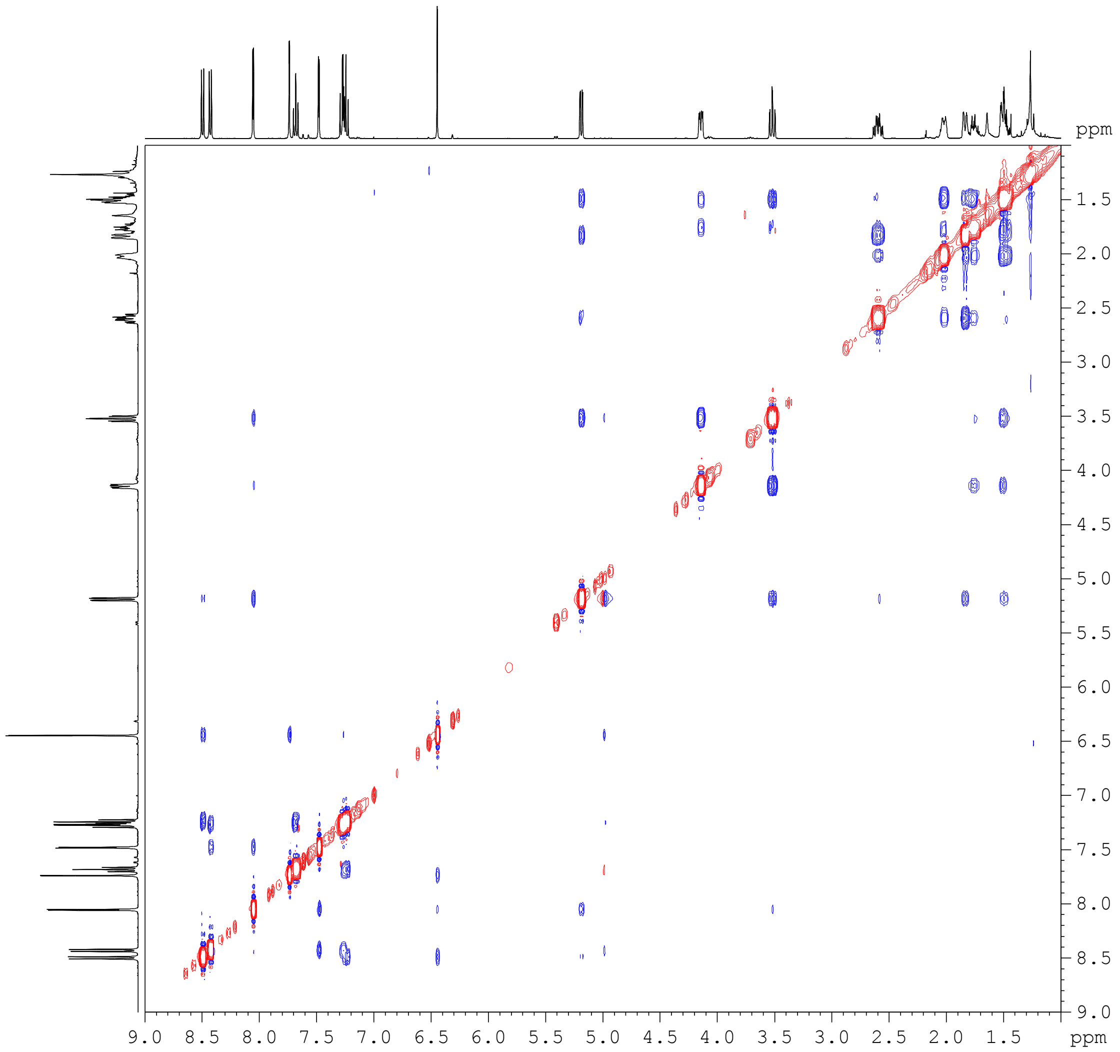

HSQC

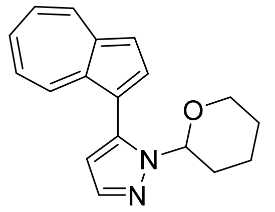

13j (500 MHz, $CDCl_3$)

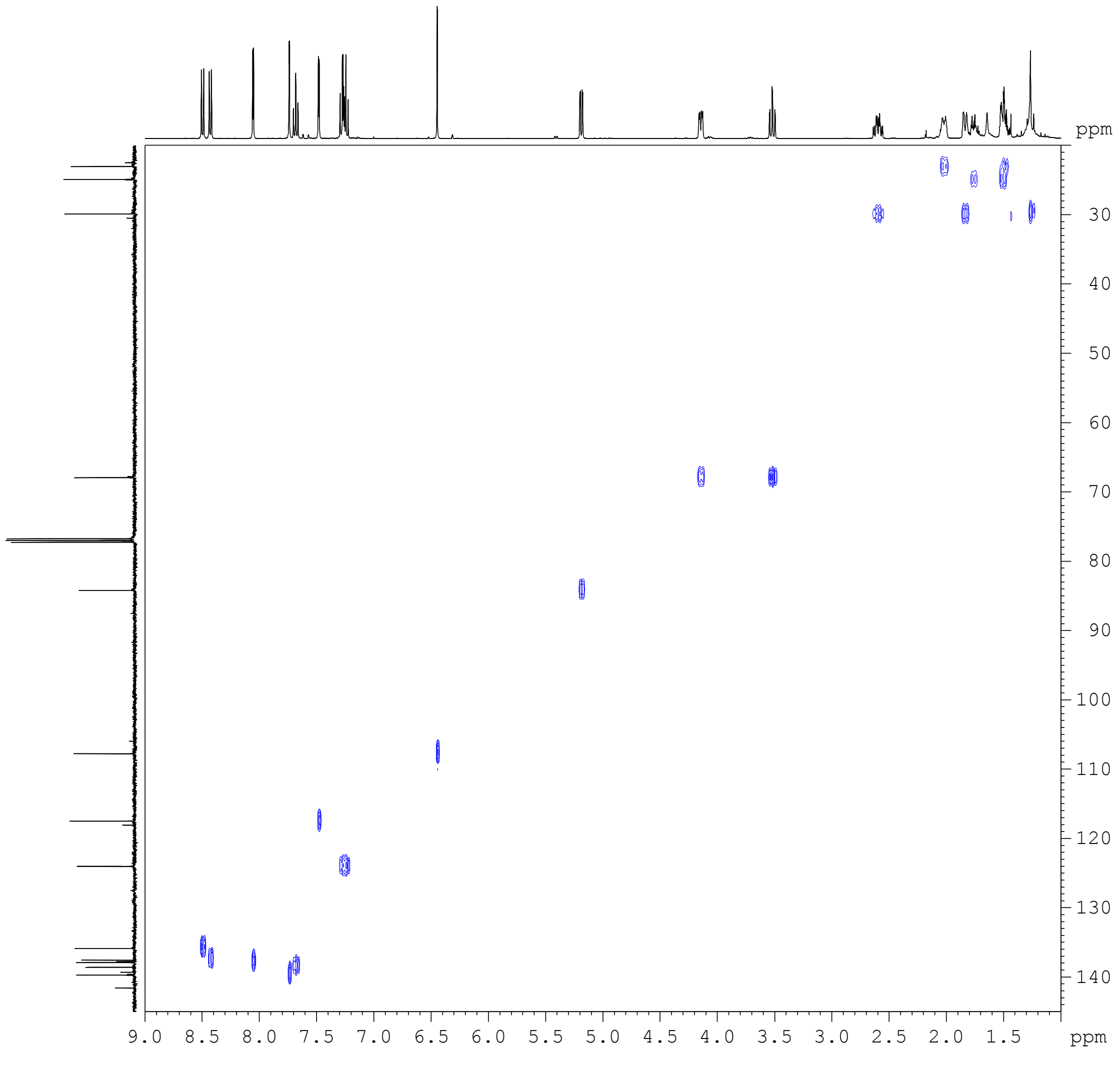

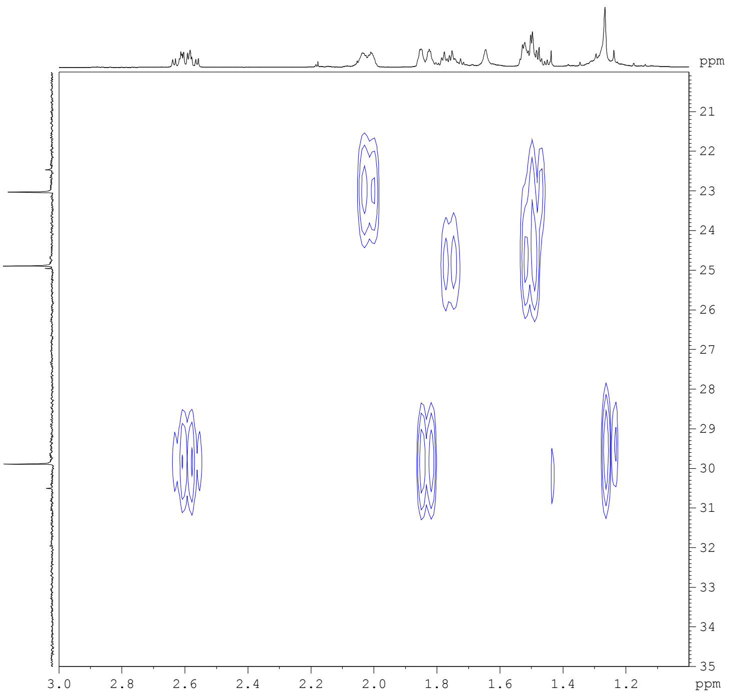

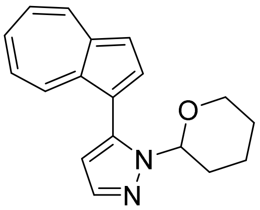

13j (500 MHz, $CDCl_3$)

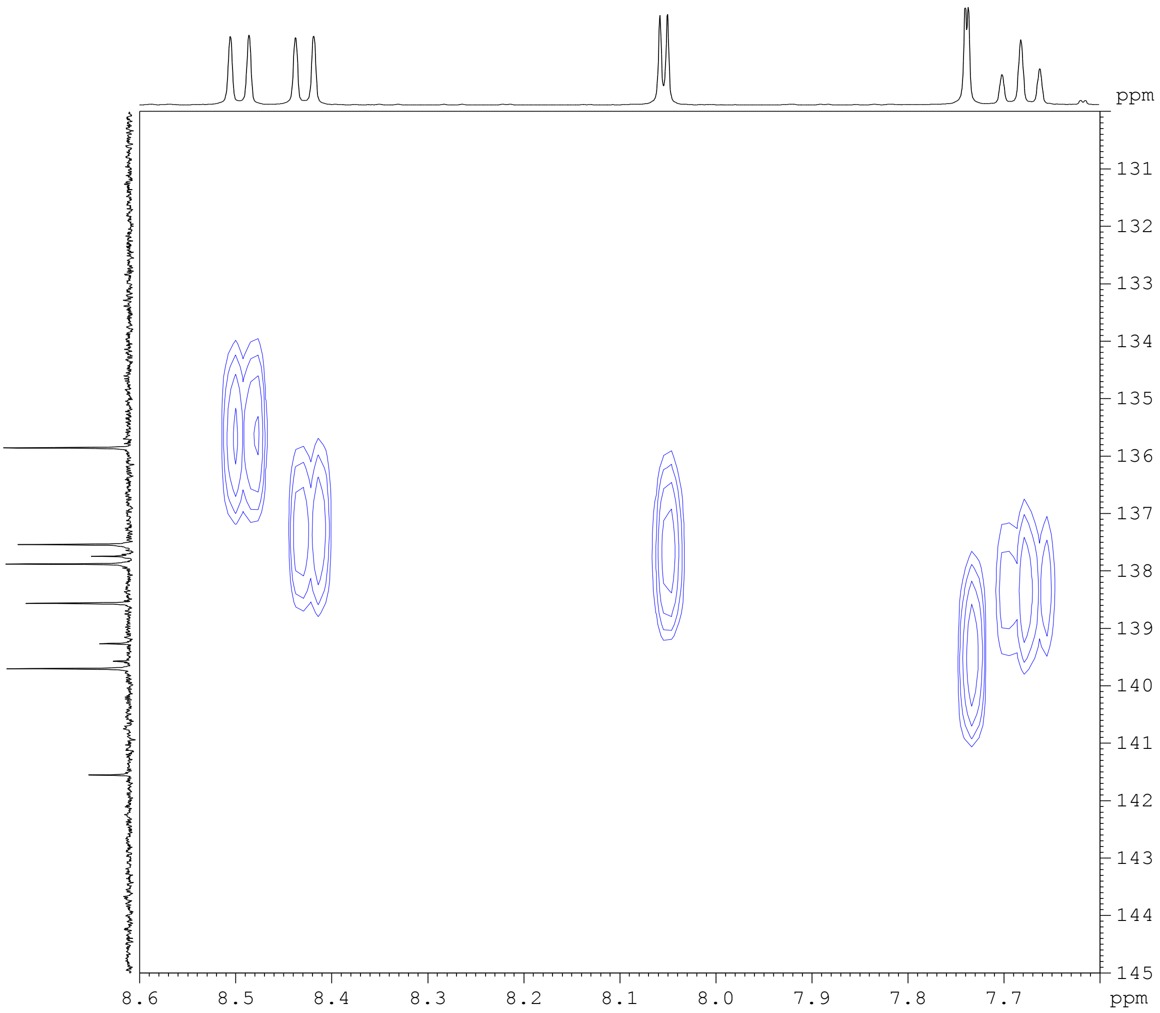

HMBC

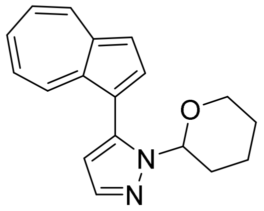

13j (500 MHz, $CDCl_3$)

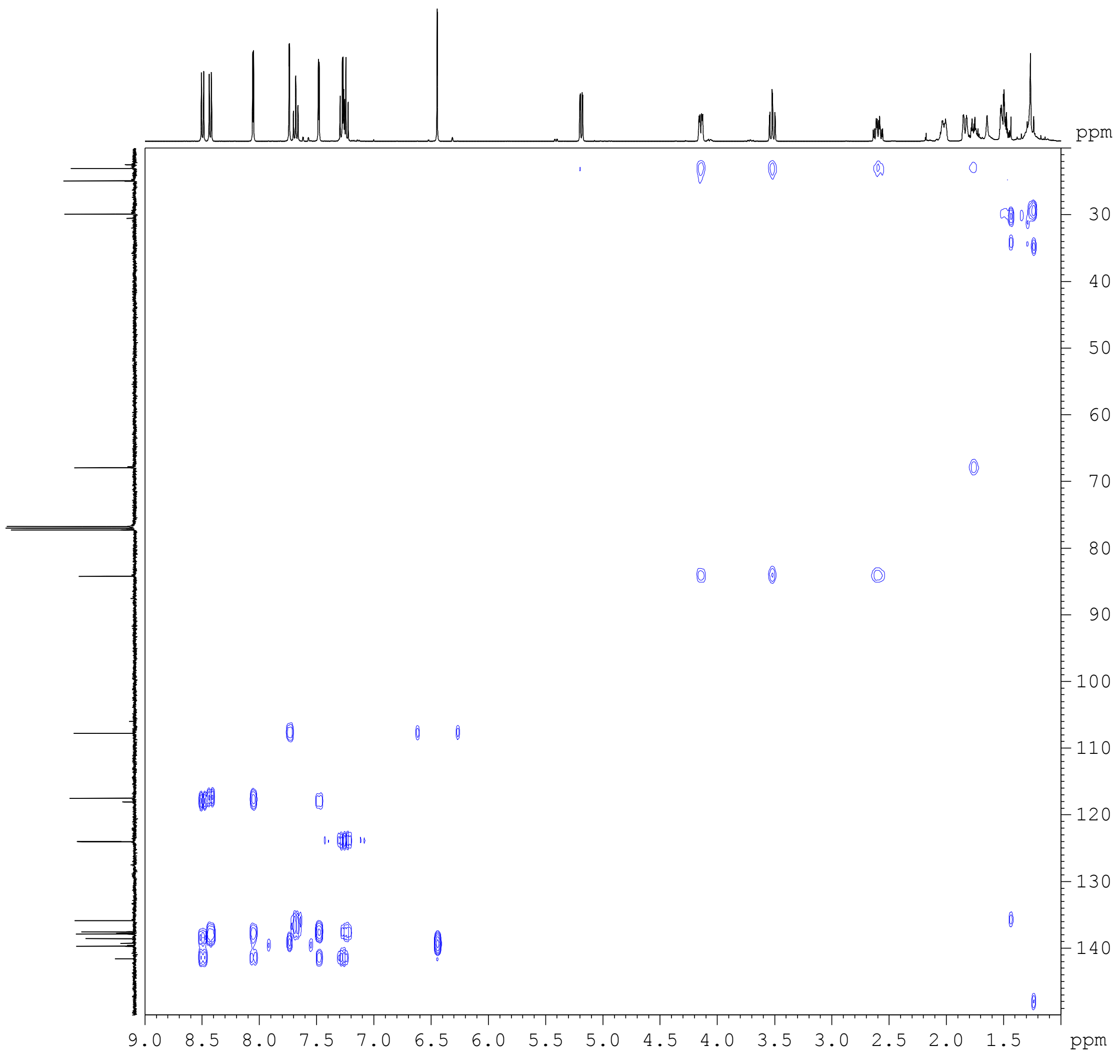

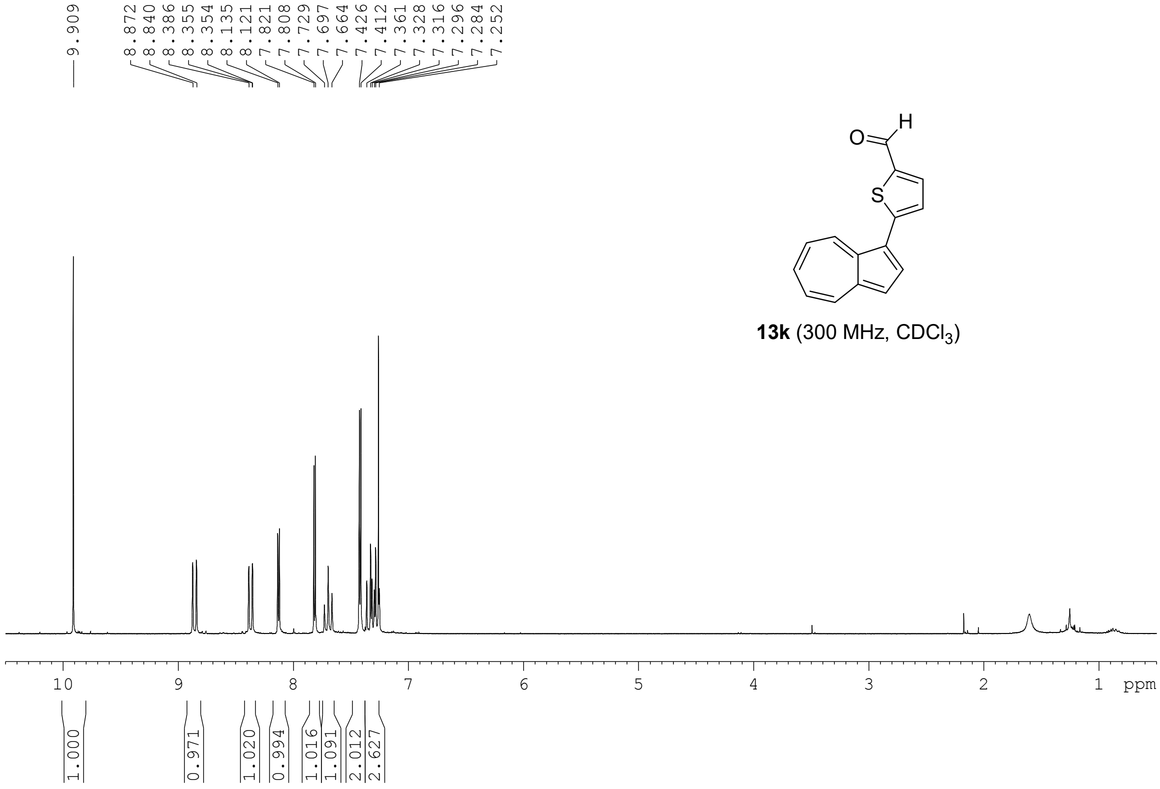

13k (300 MHz, $CDCl_3$)

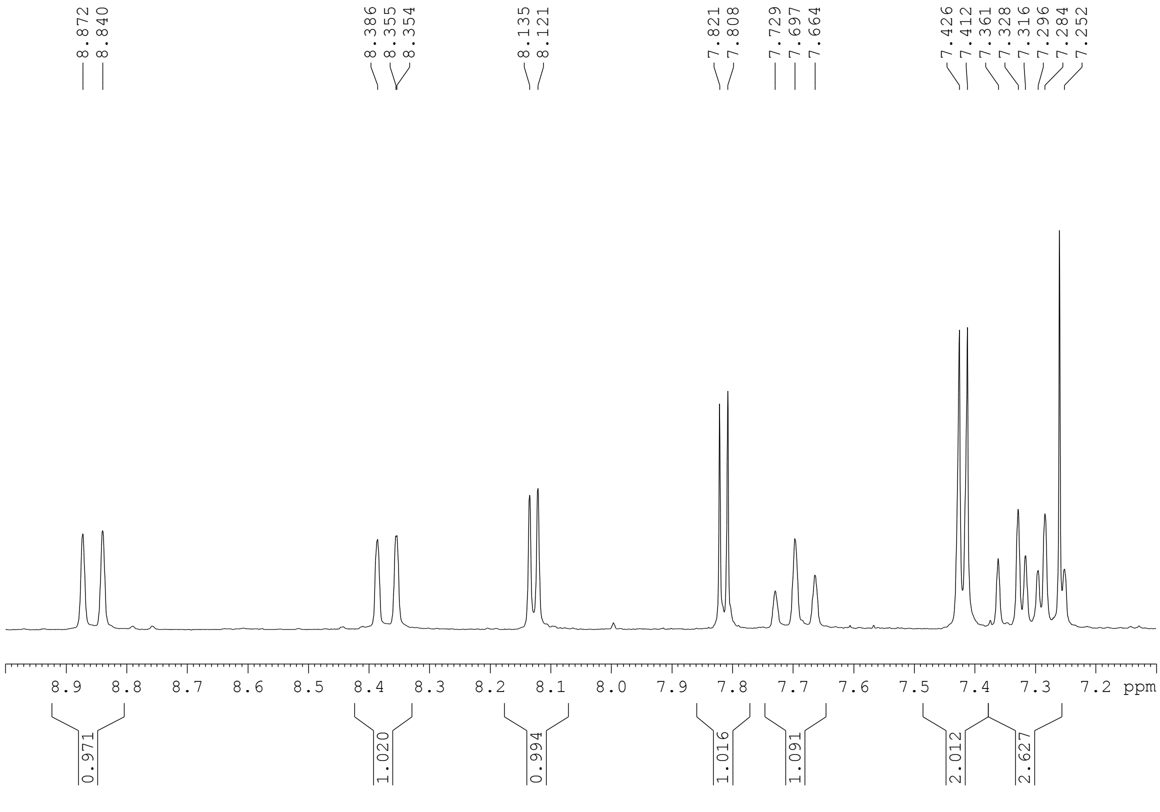

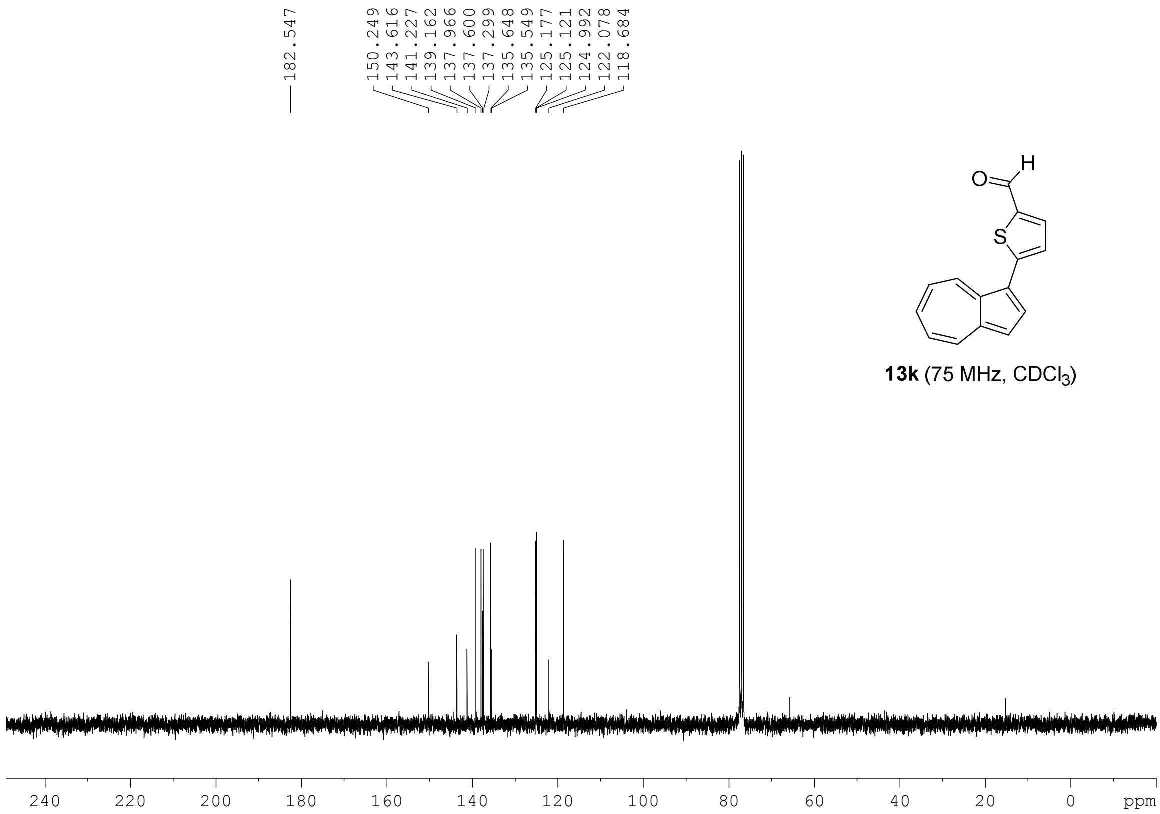

13k (75 MHz, $CDCl_3$)

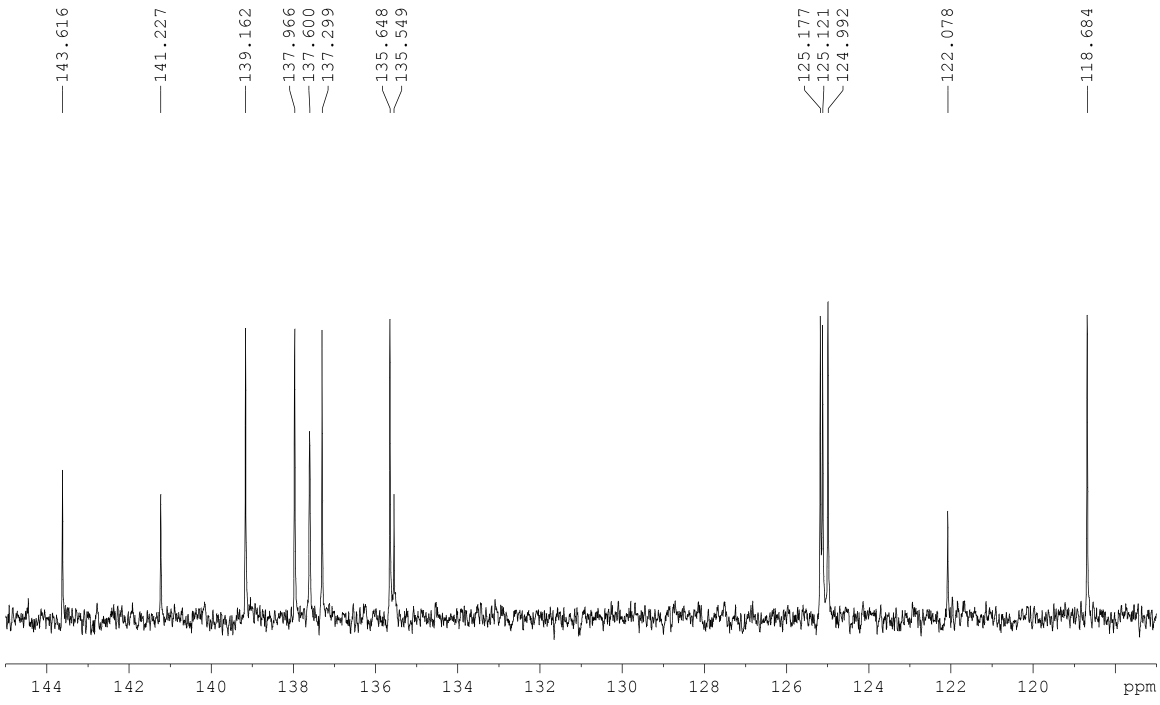

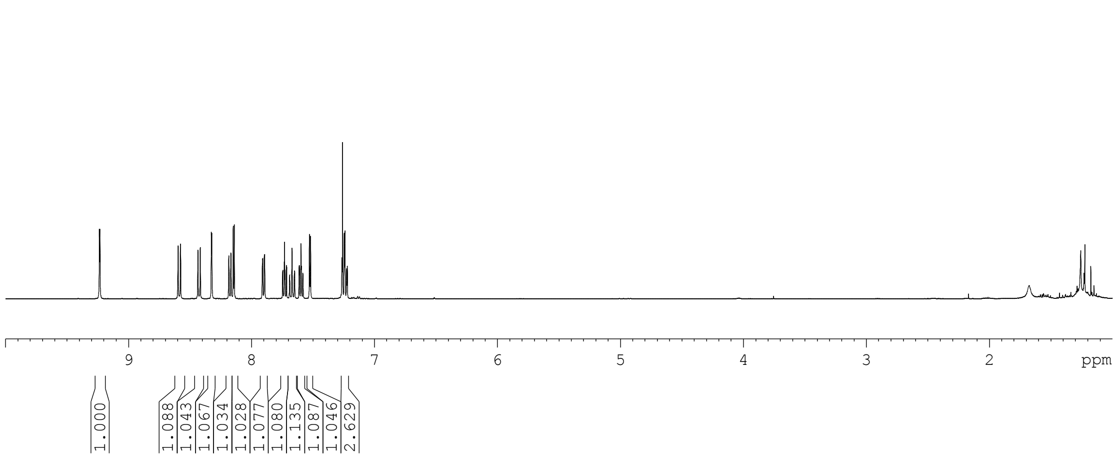

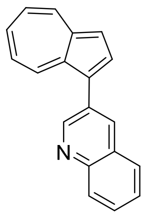

13l (500 MHz, $CDCl_3$)

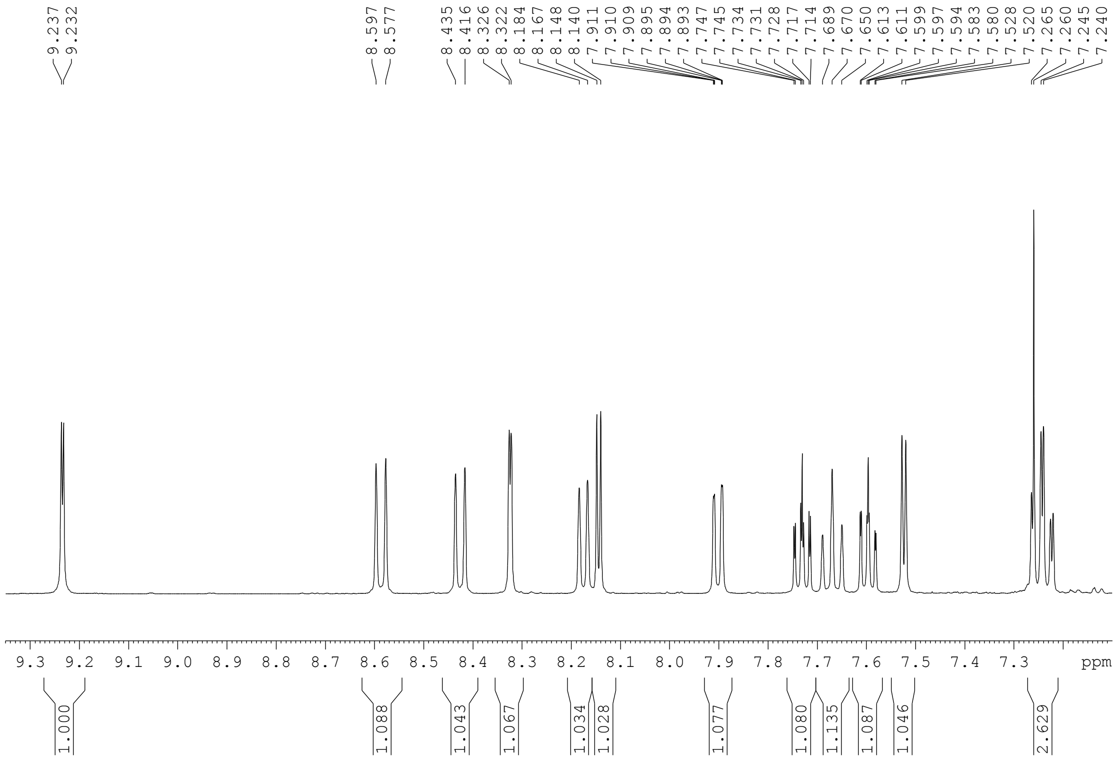

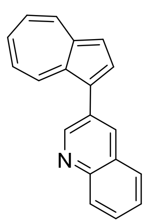

13l (126 MHz, $CDCl_3$)

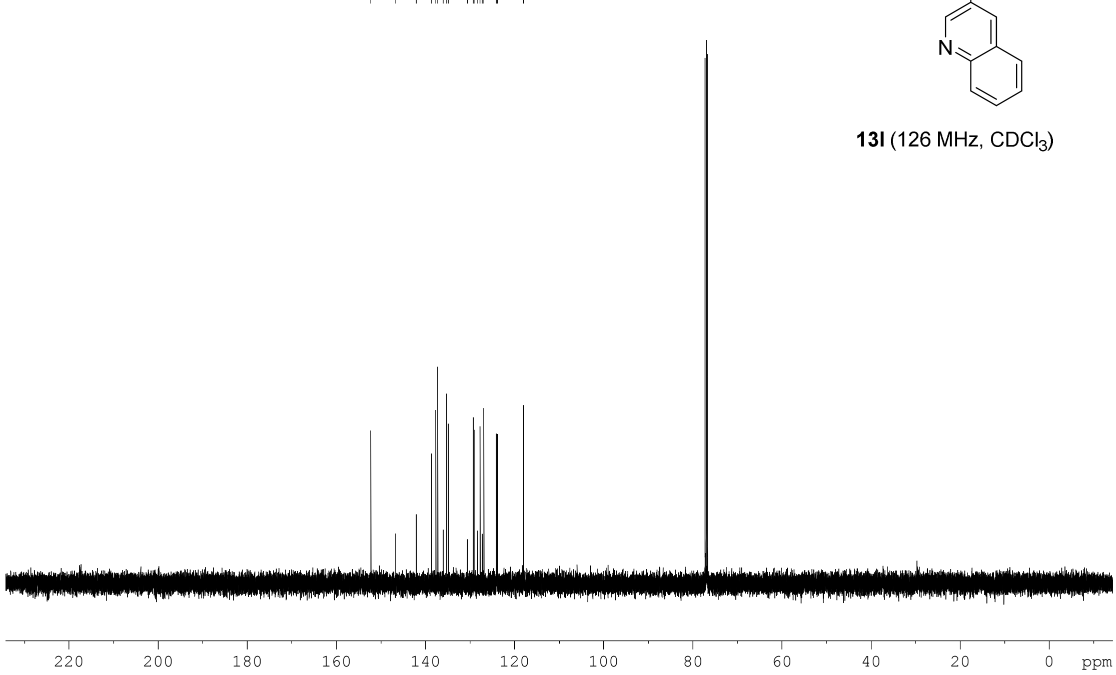

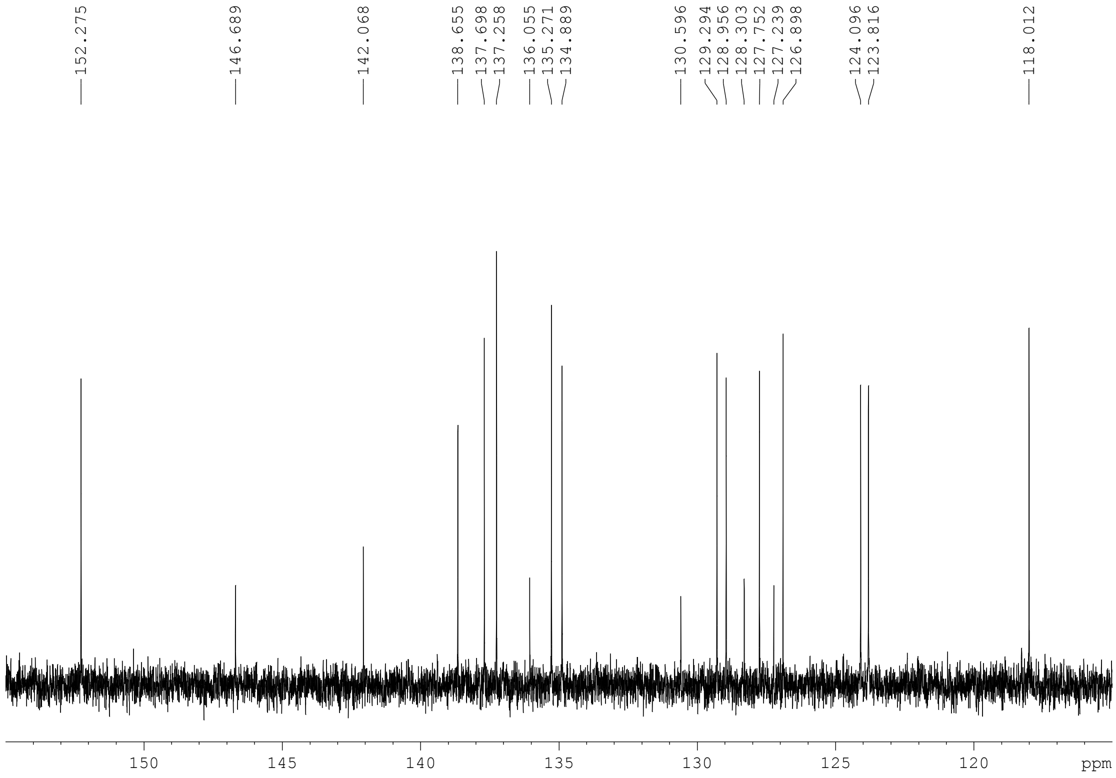

COSY

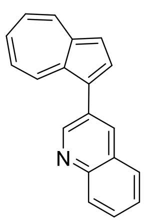

13l (500 MHz, $CDCl_3$)

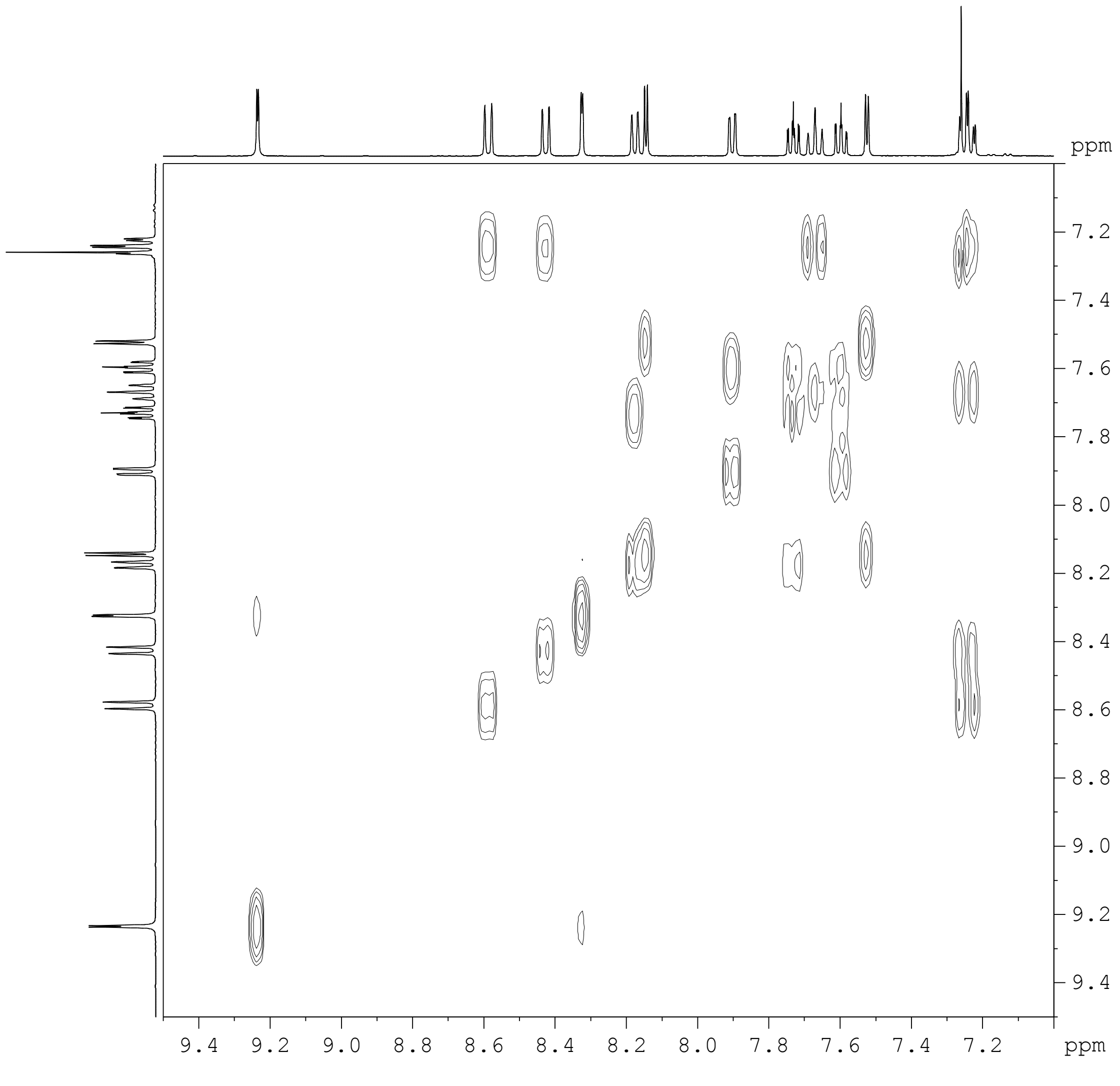

NOESY

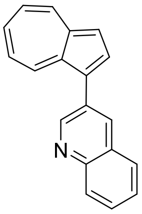

13l (500 MHz, $CDCl_3$)

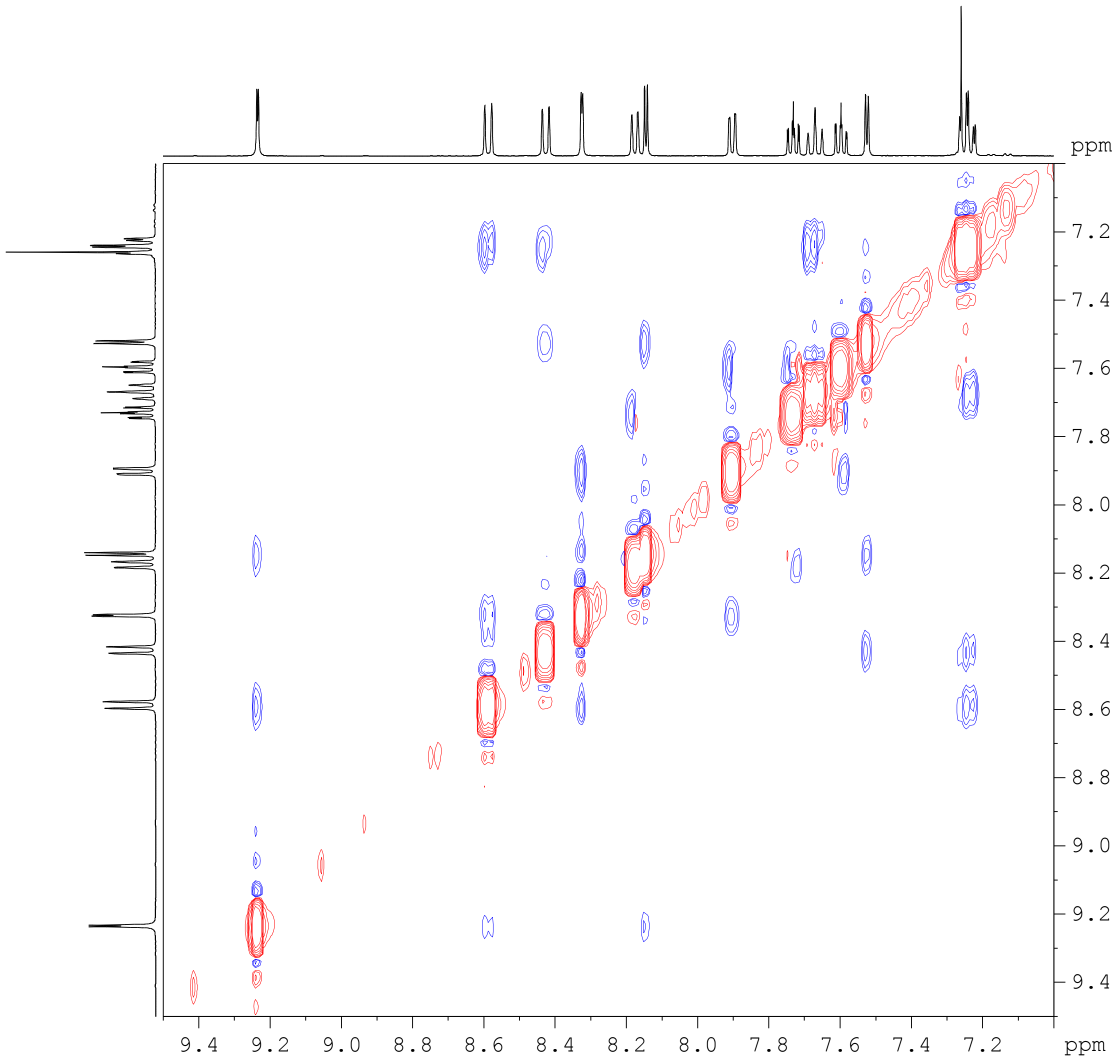

HSQC

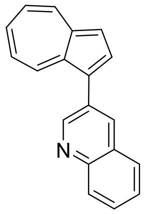

13l (500 MHz, $CDCl_3$)

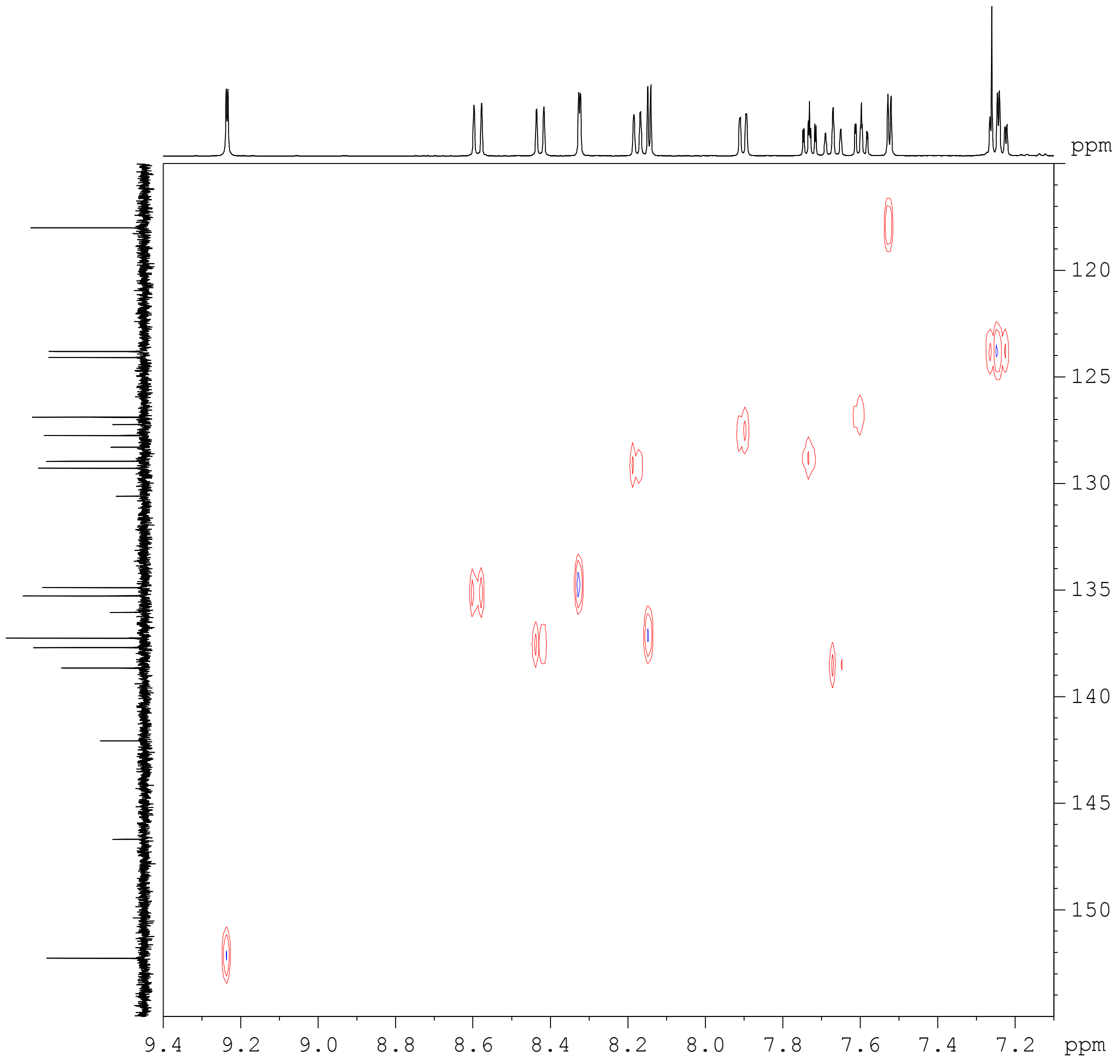

HMBC

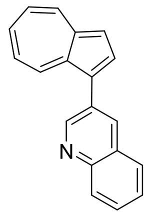

13l (500 MHz, $CDCl_3$)

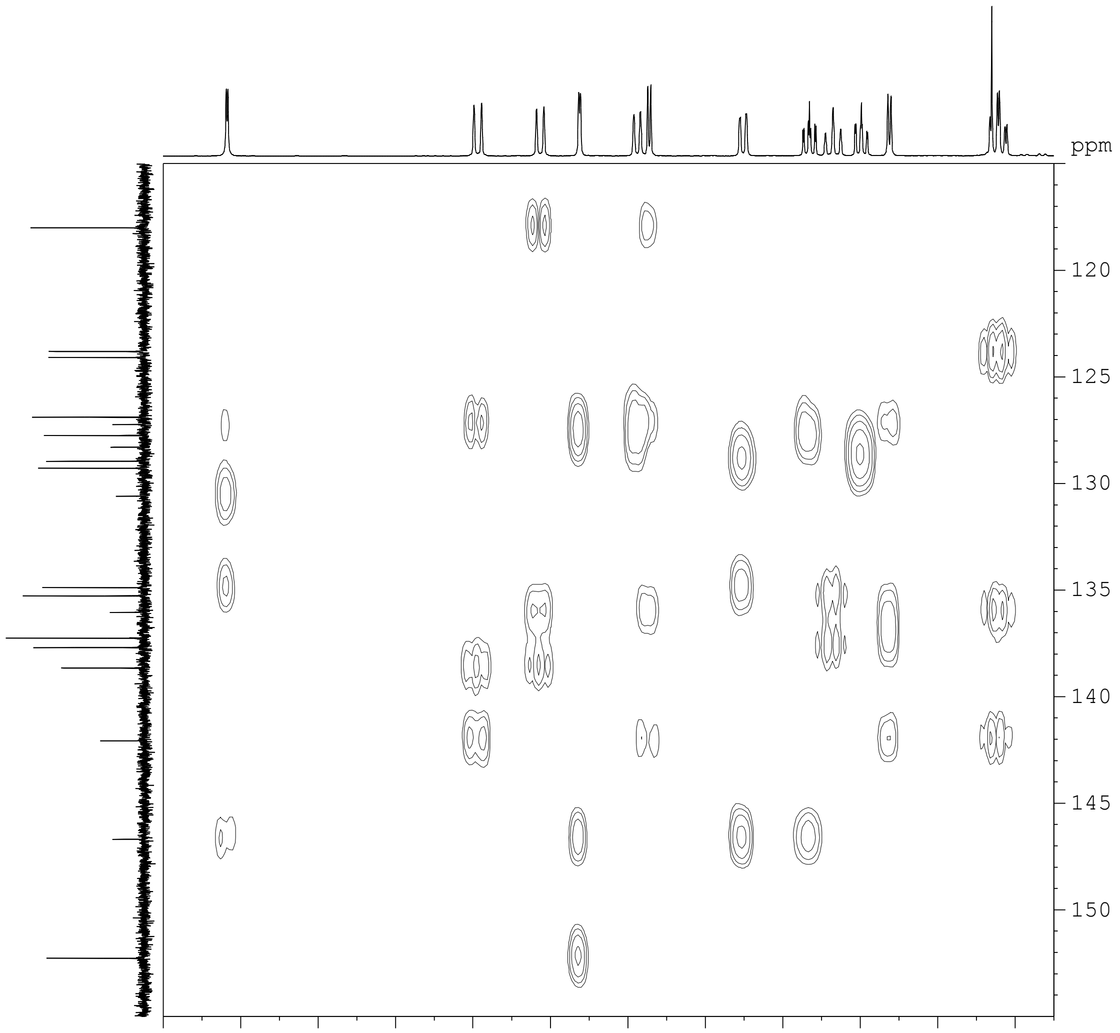

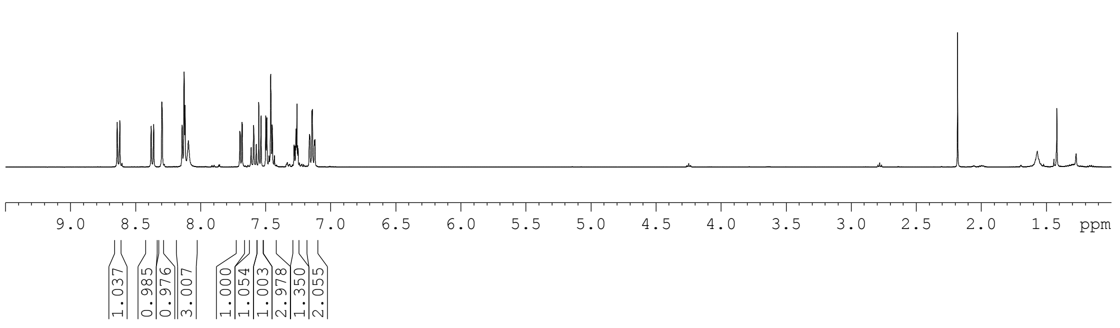

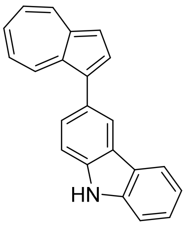

13m (500 MHz, $CDCl_3$)

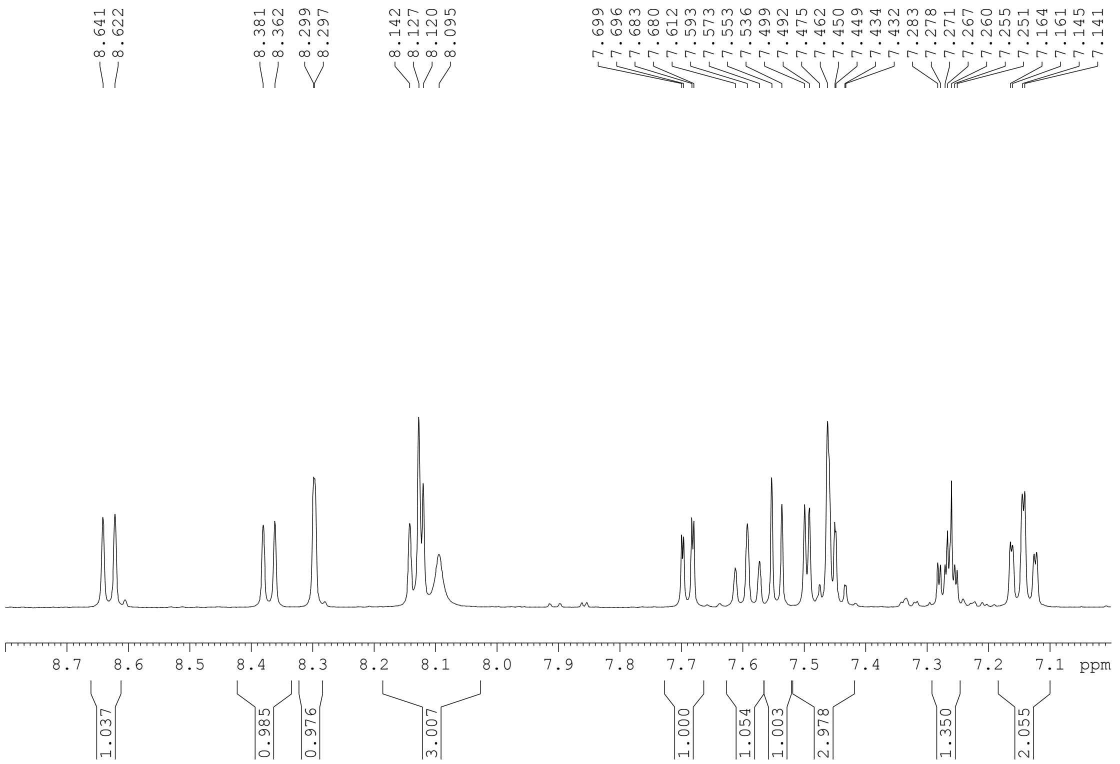

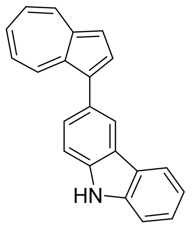

13m (126 MHz, $CDCl_3$)

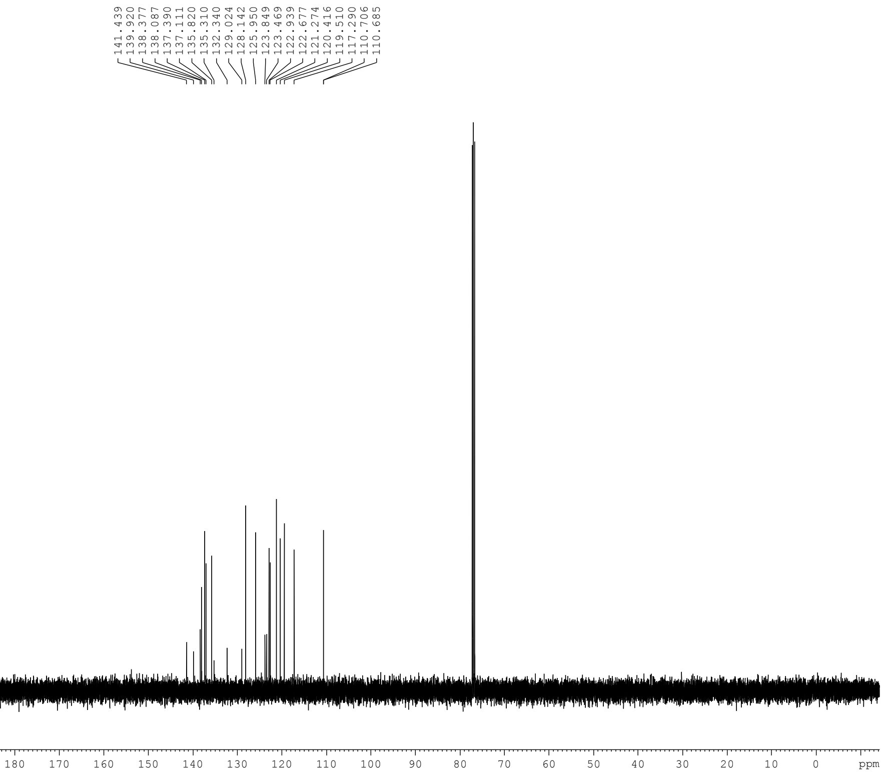

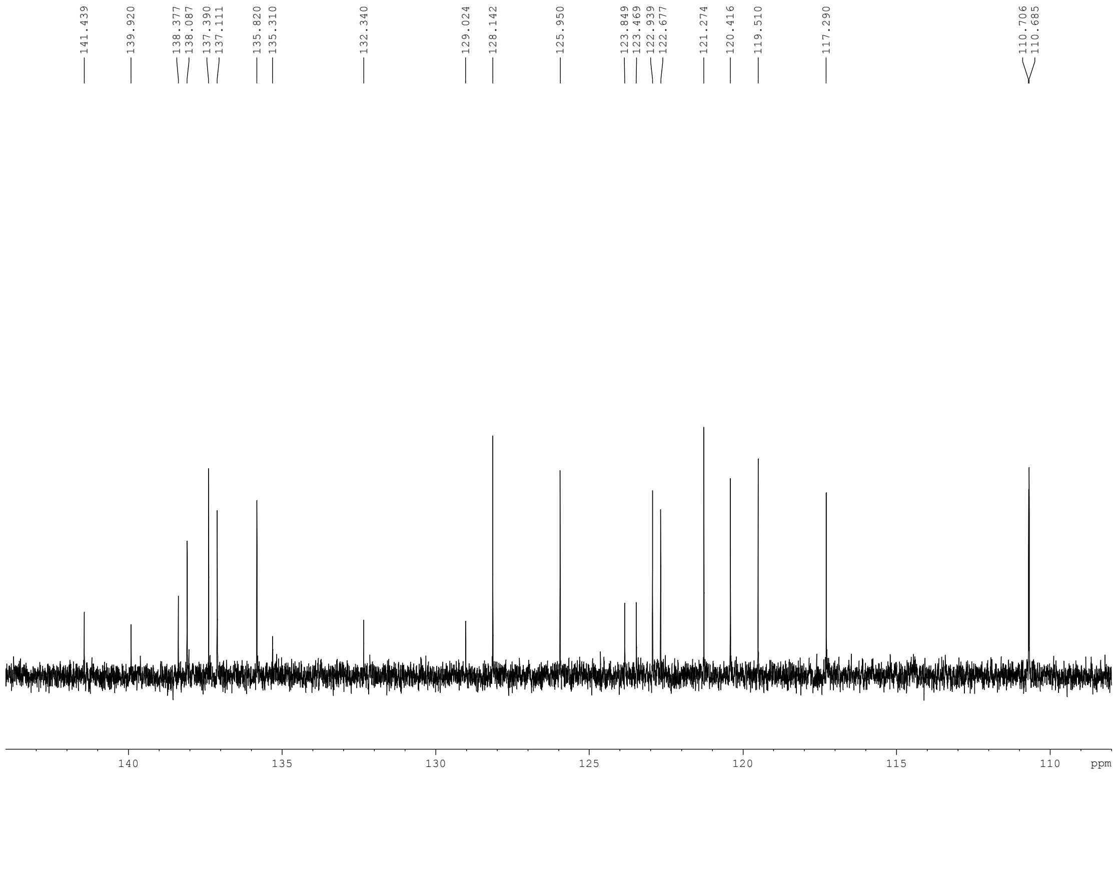

COSY

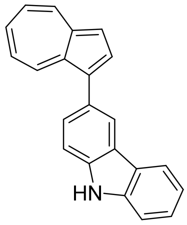

13m (500 MHz, $CDCl_3$)

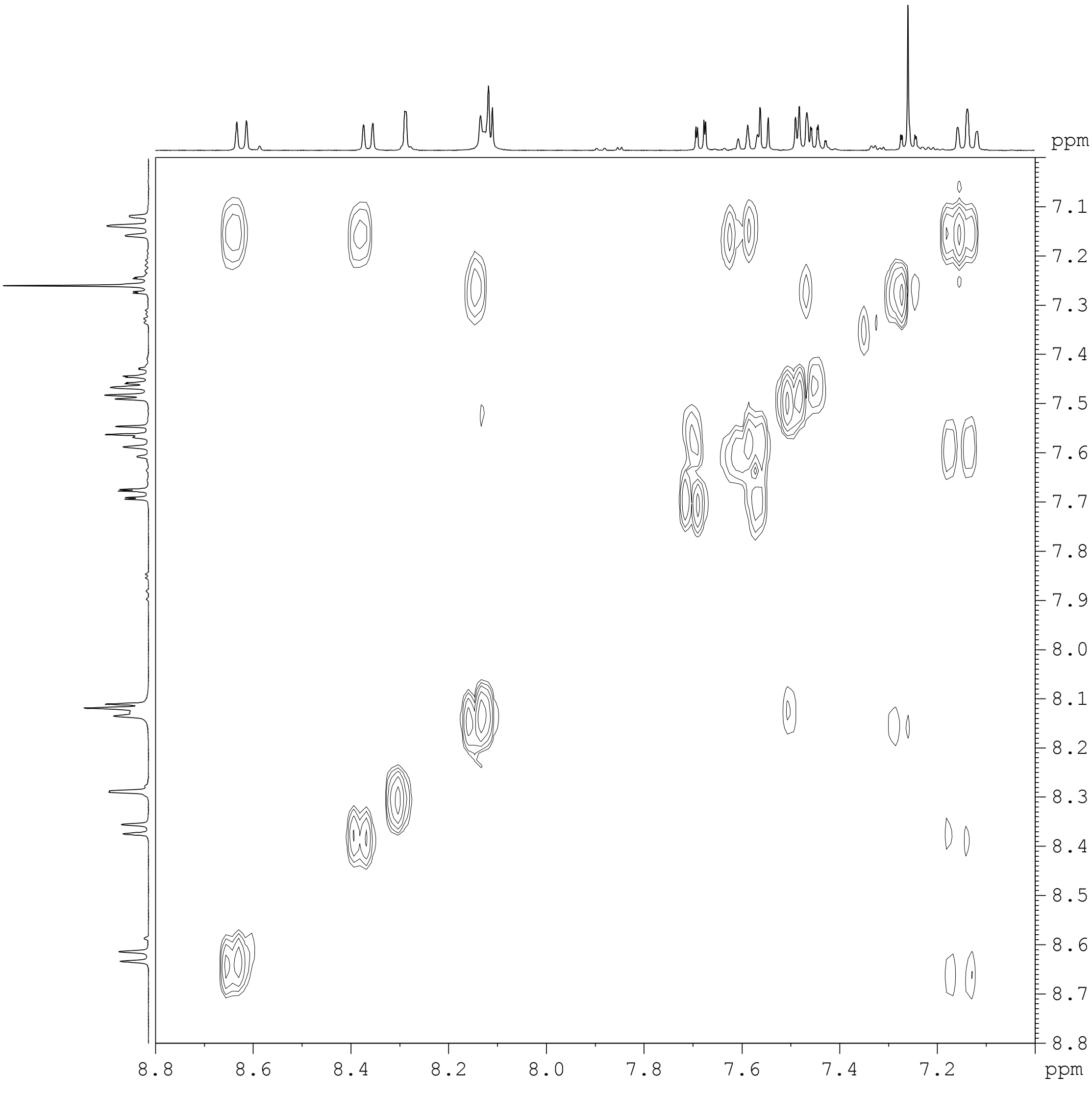

NOESY

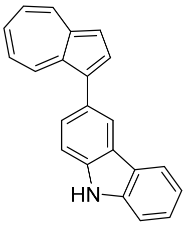

13m (500 MHz, $CDCl_3$)

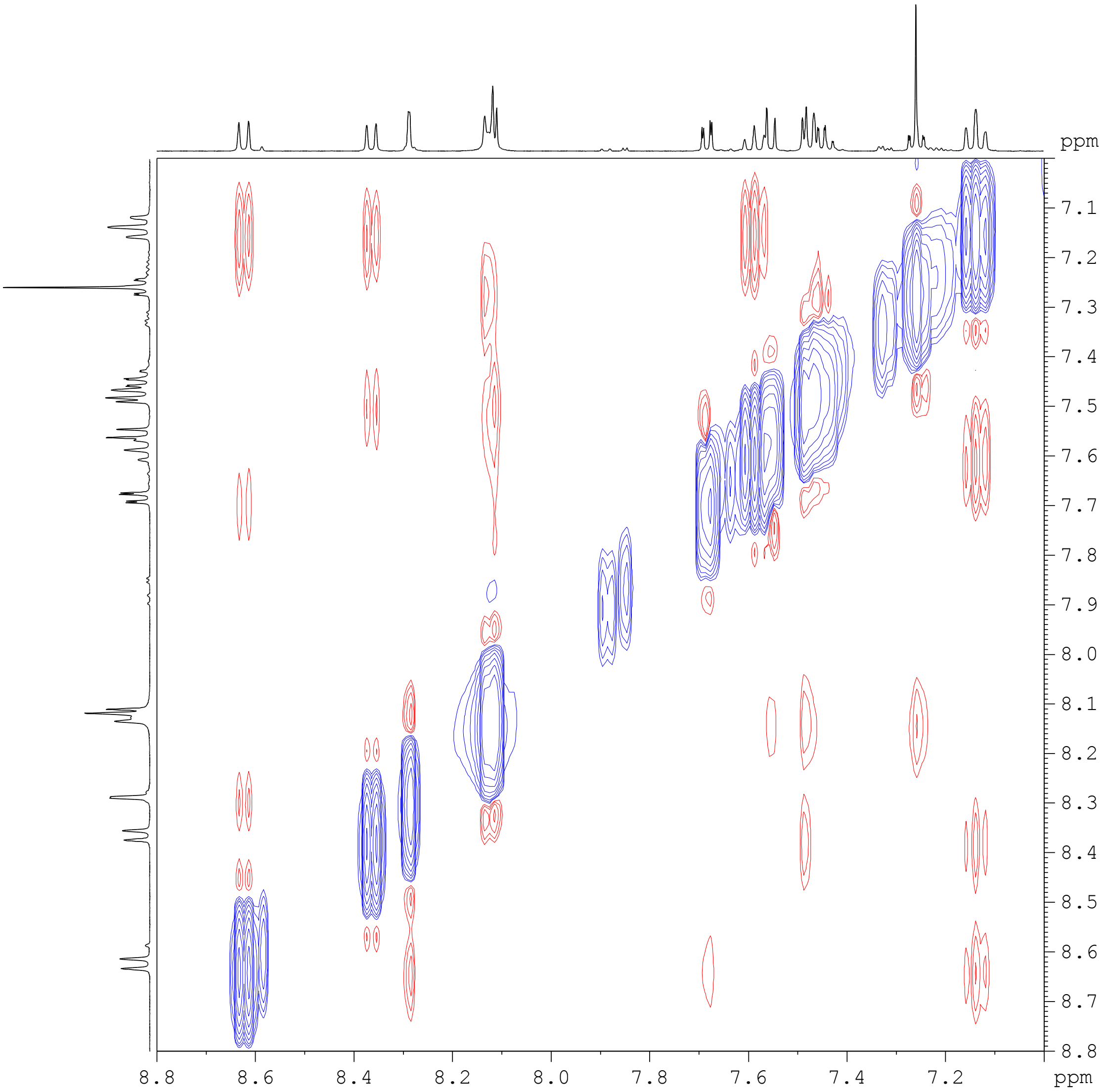

HSQC

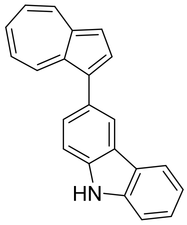

13m (500 MHz, $CDCl_3$)

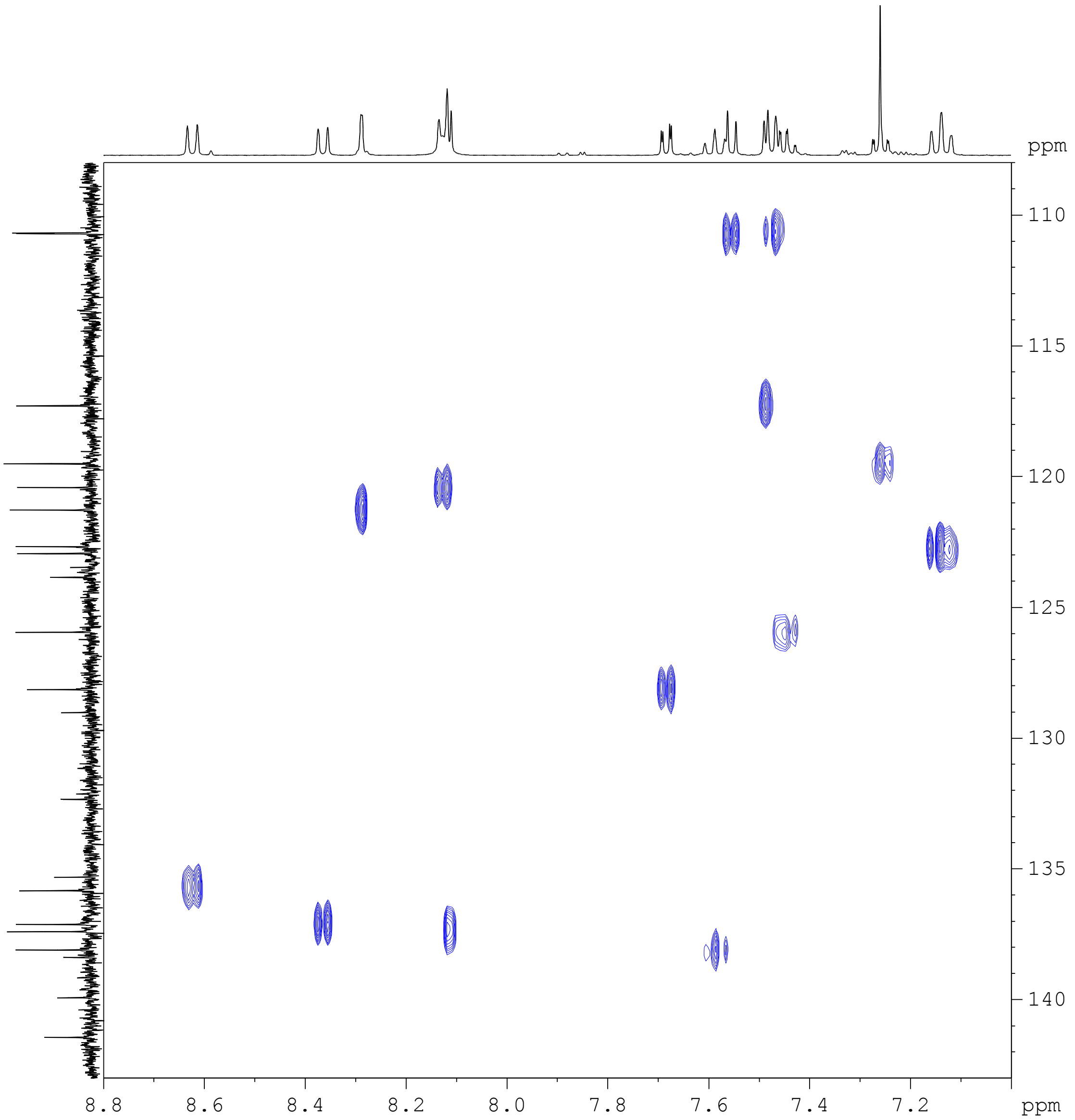

HMBC

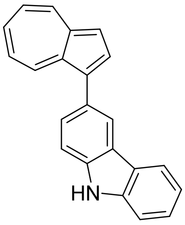

13m (500 MHz, $CDCl_3$)

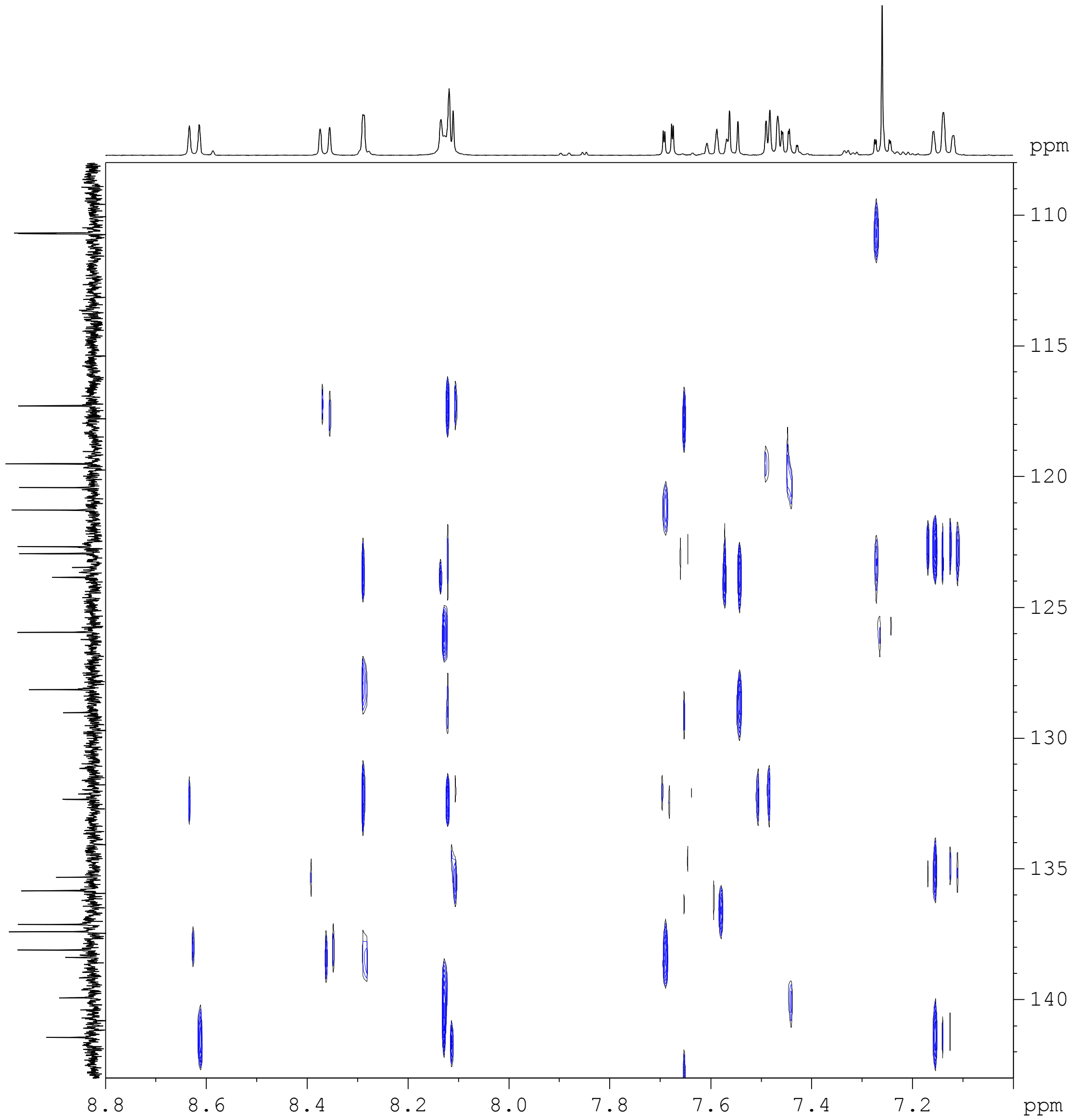

HMBC

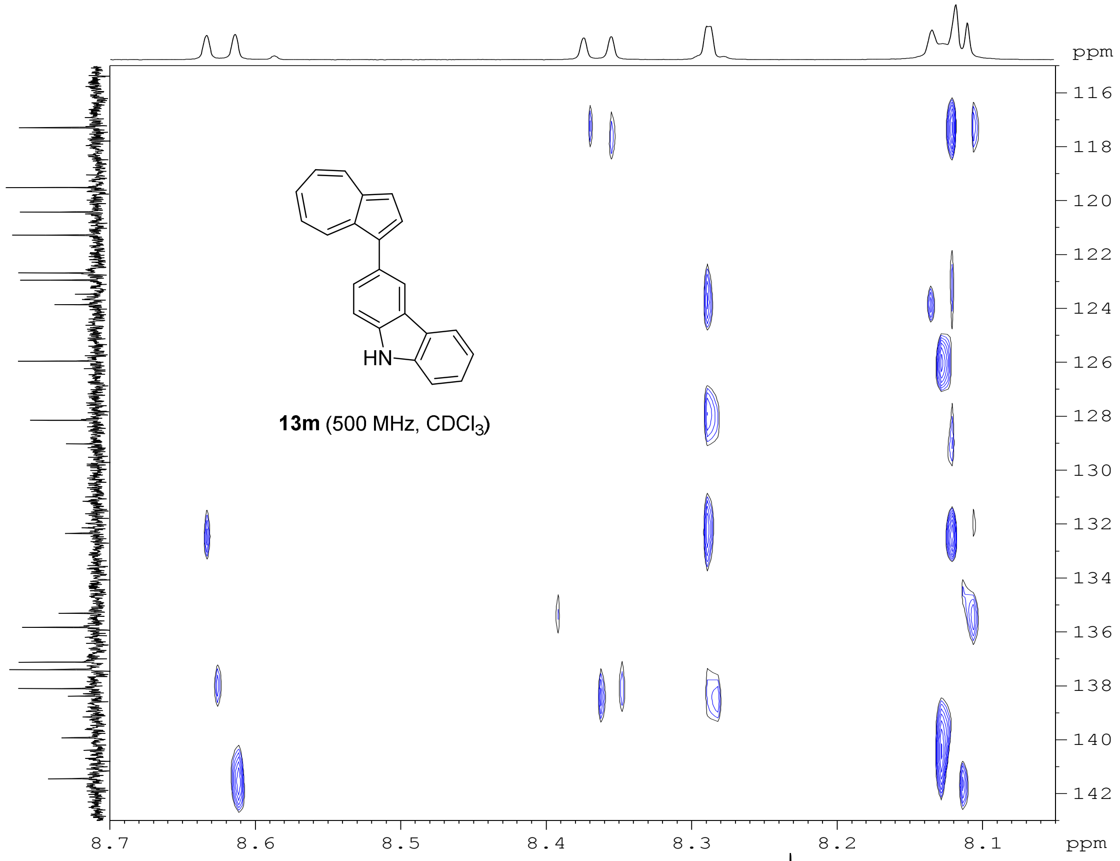
HN
13m (500 MHz, $CDCl_3$)
ppm
116
118
120
122
124
126
128
130
132
134
136
138
140
142
8.7
8.6
8.5
8.4
8.3
8.2
8.1
ppm

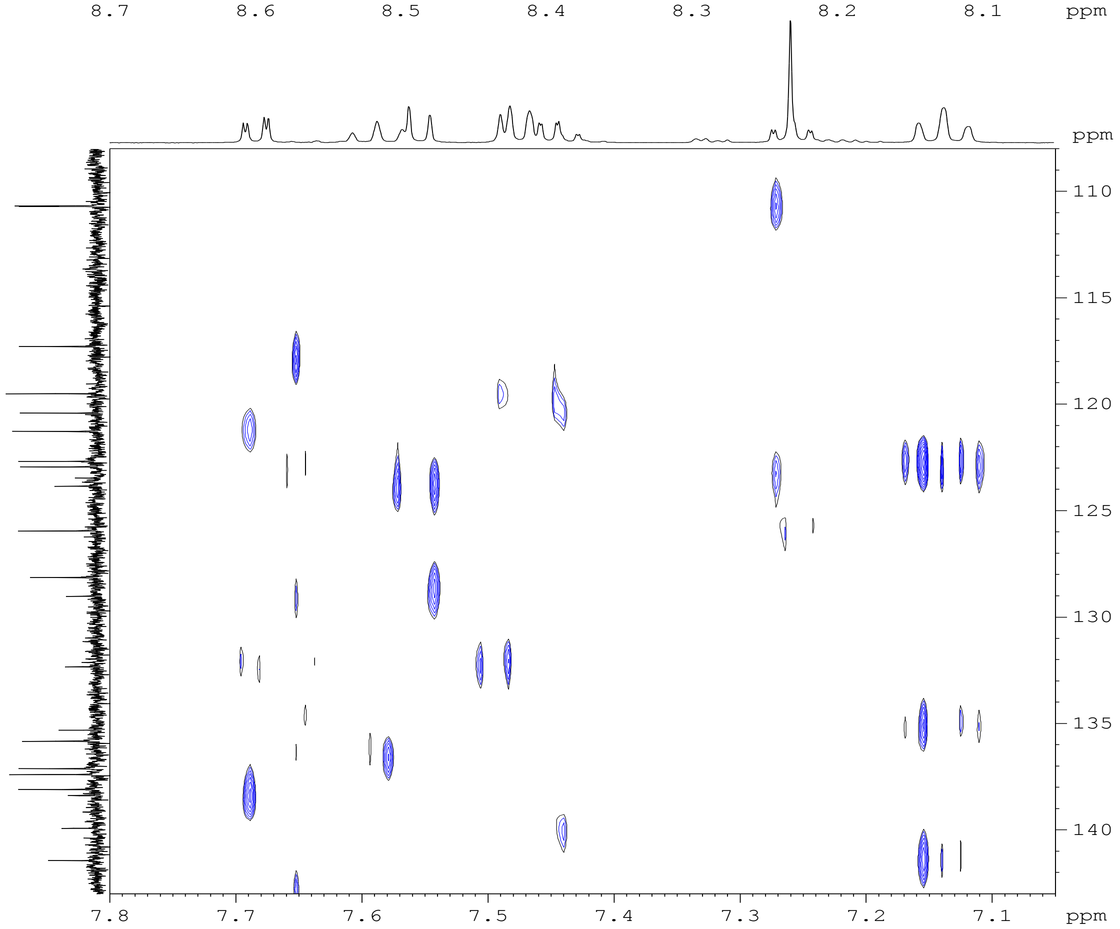
ppm
110
115
120
125
130
135
140
7.8
7.7
7.6
7.5
7.4
7.3
7.2
7.1
ppm

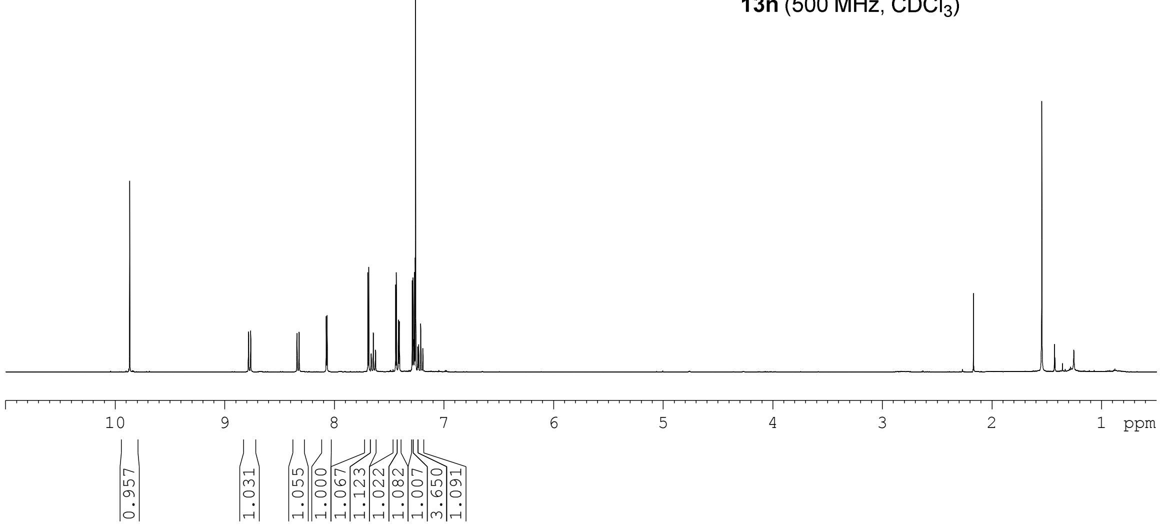

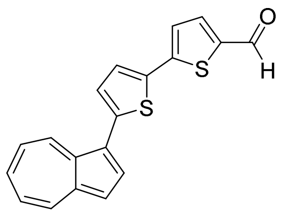

13n (500 MHz, $CDCl_3$)

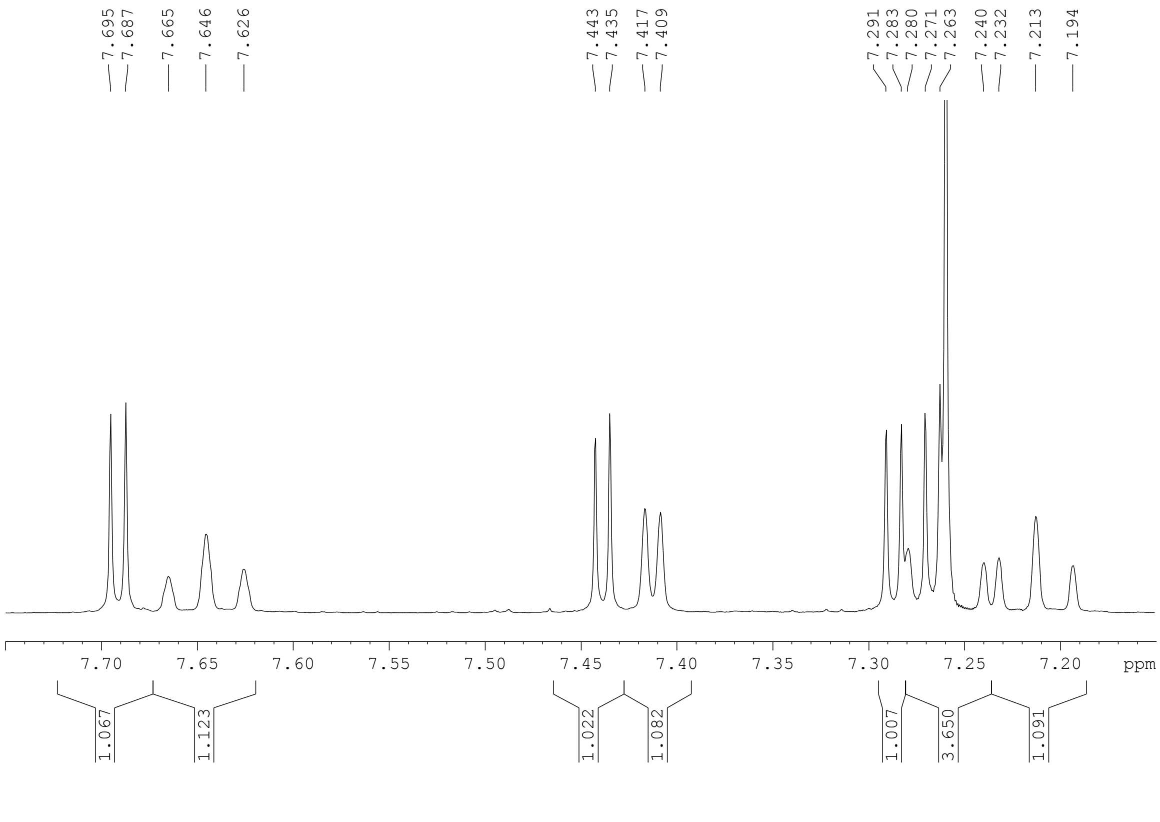

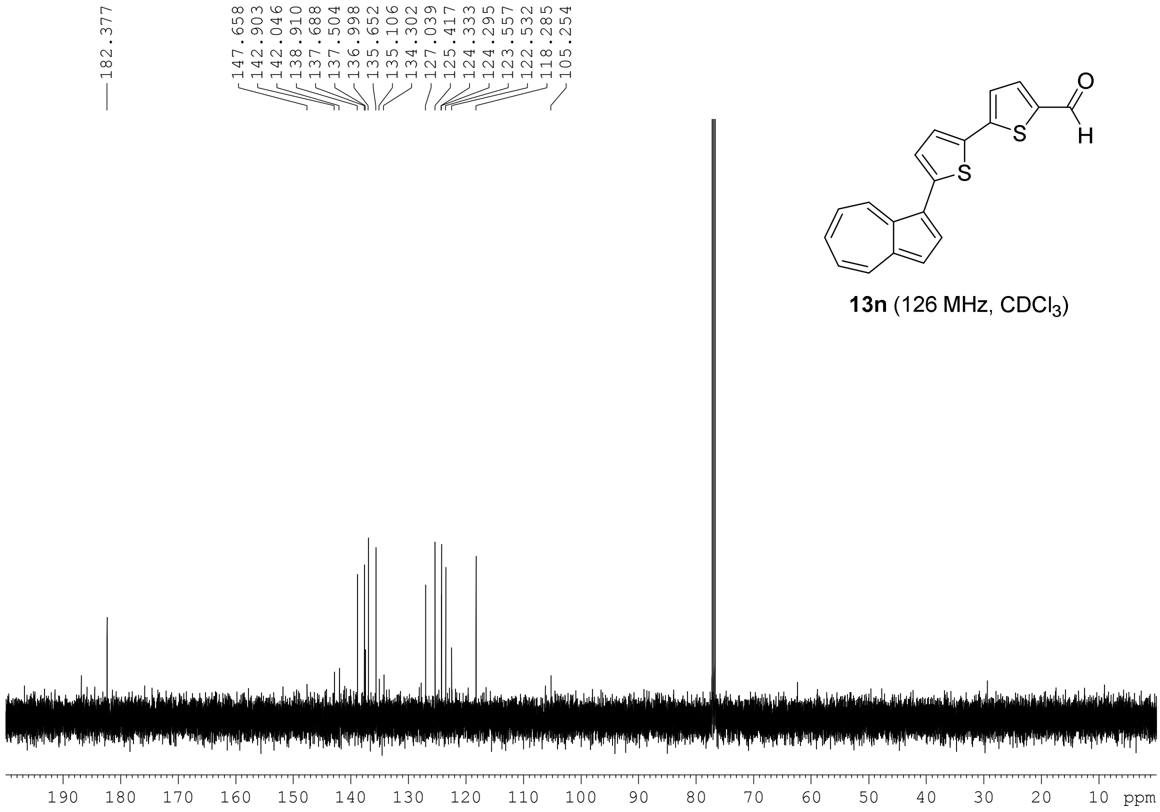

13n (126 MHz, $CDCl_3$)

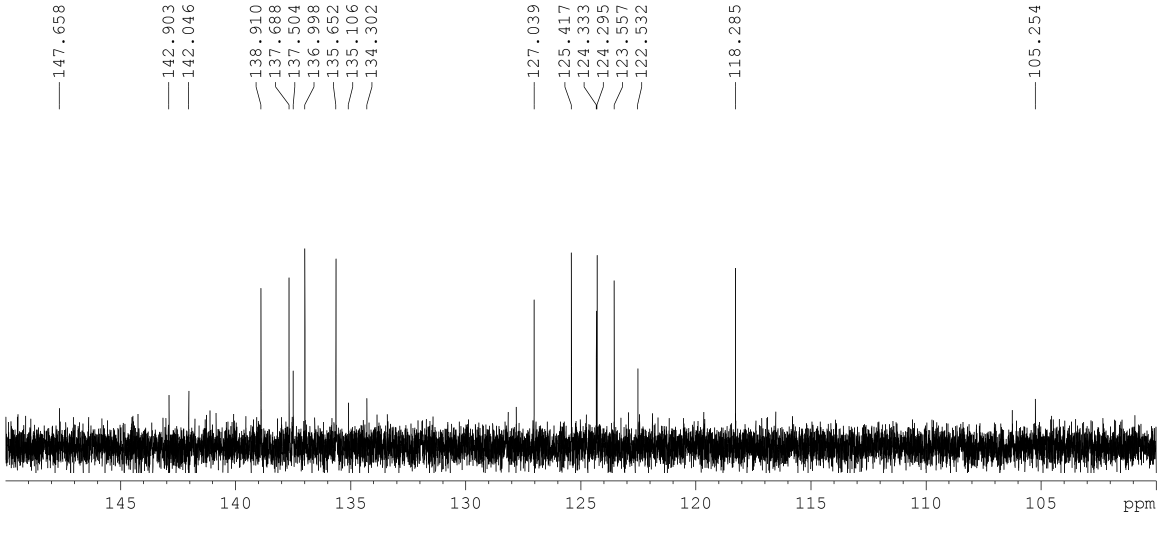

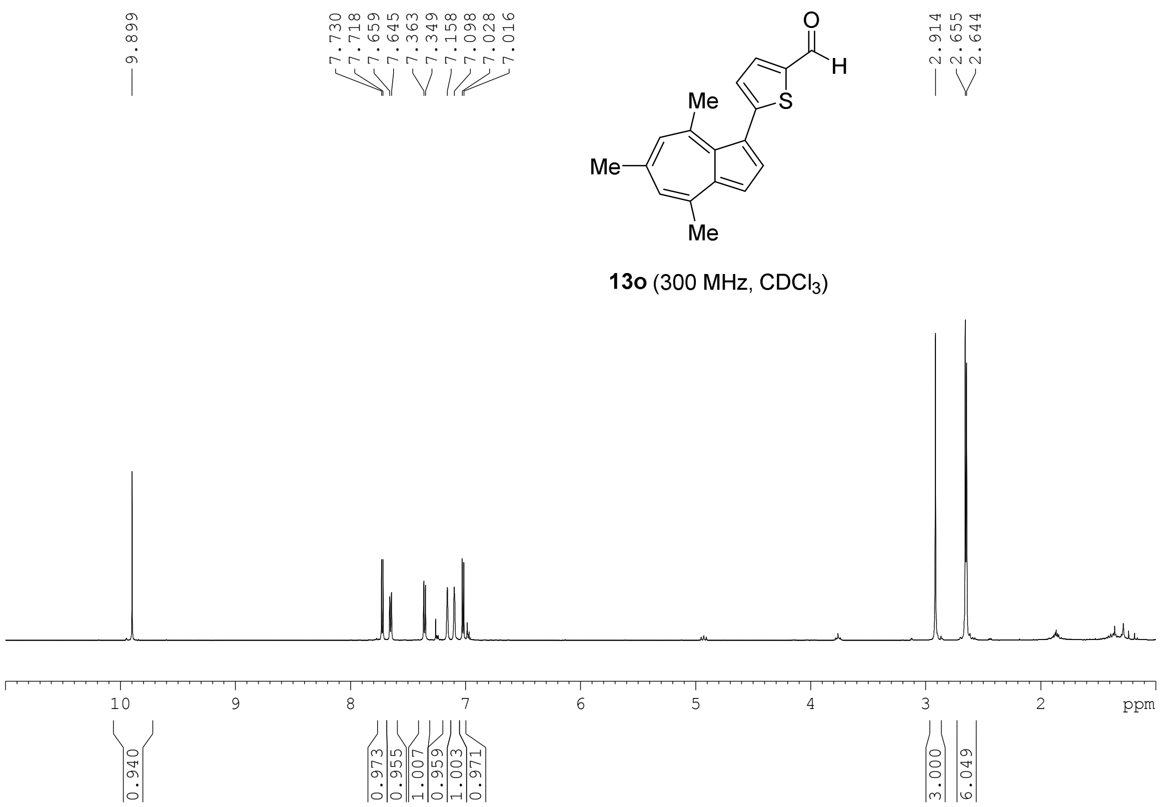

13o (300 MHz, $CDCl_3$)

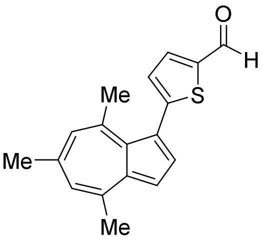

13o (75 MHz, $CDCl_3$)

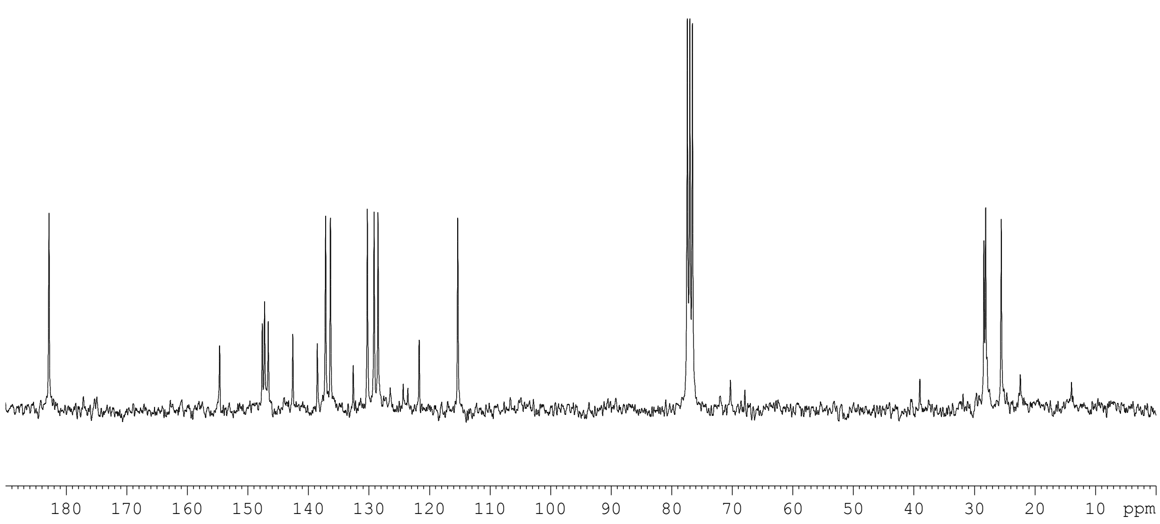

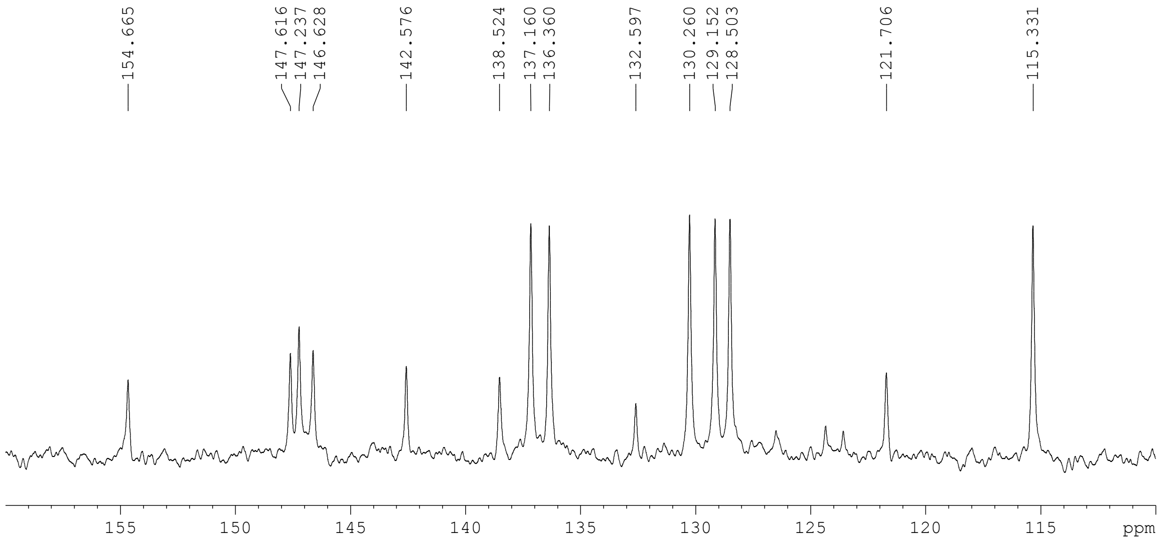

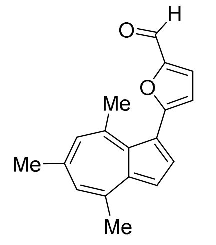

13p (500 MHz, $CDCl_3$)

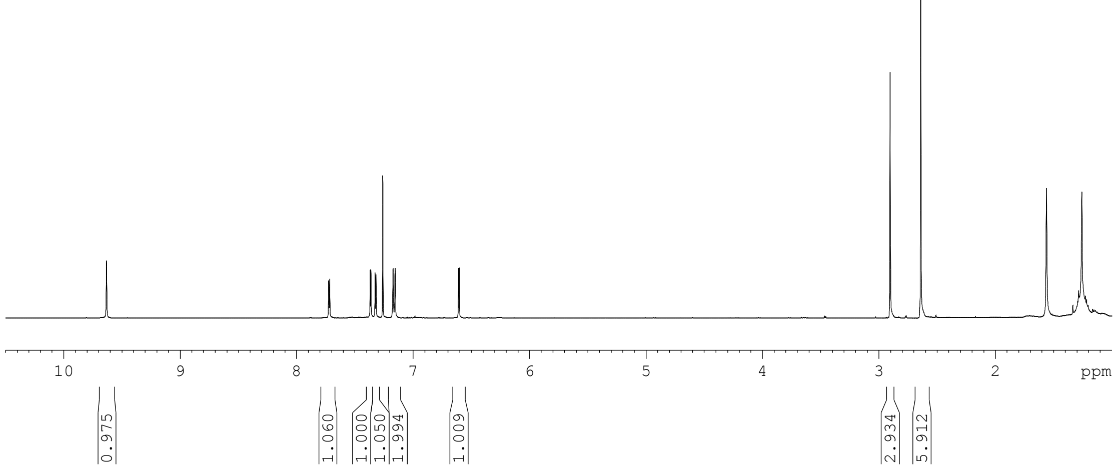

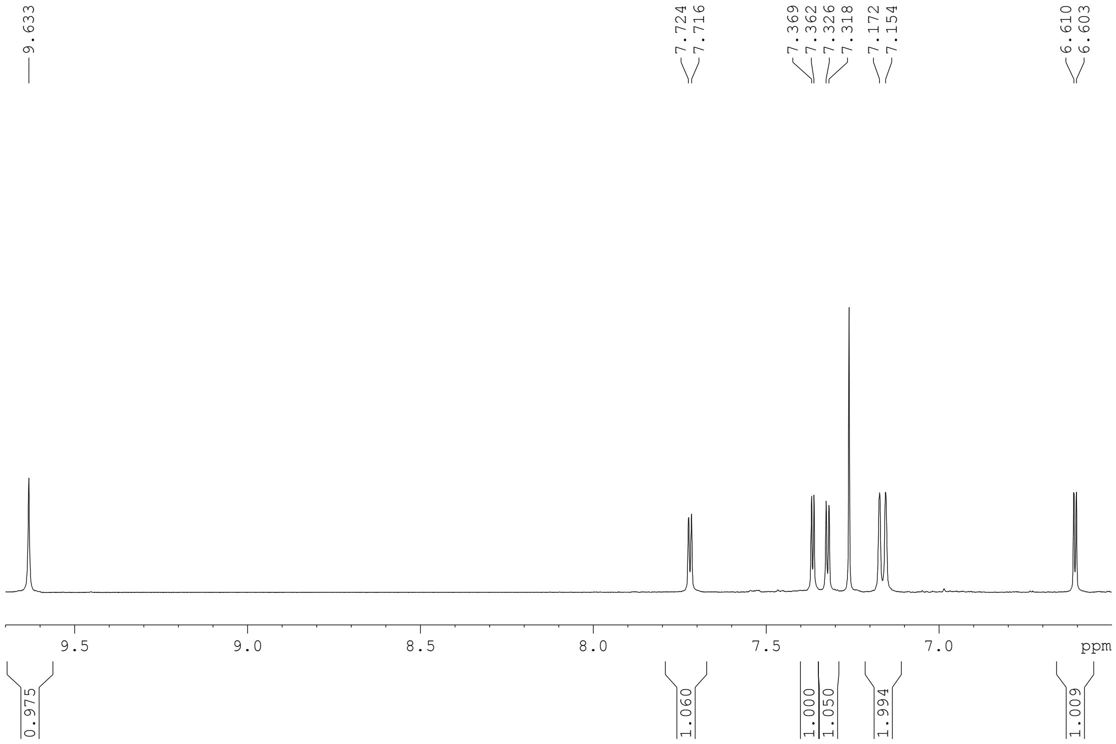

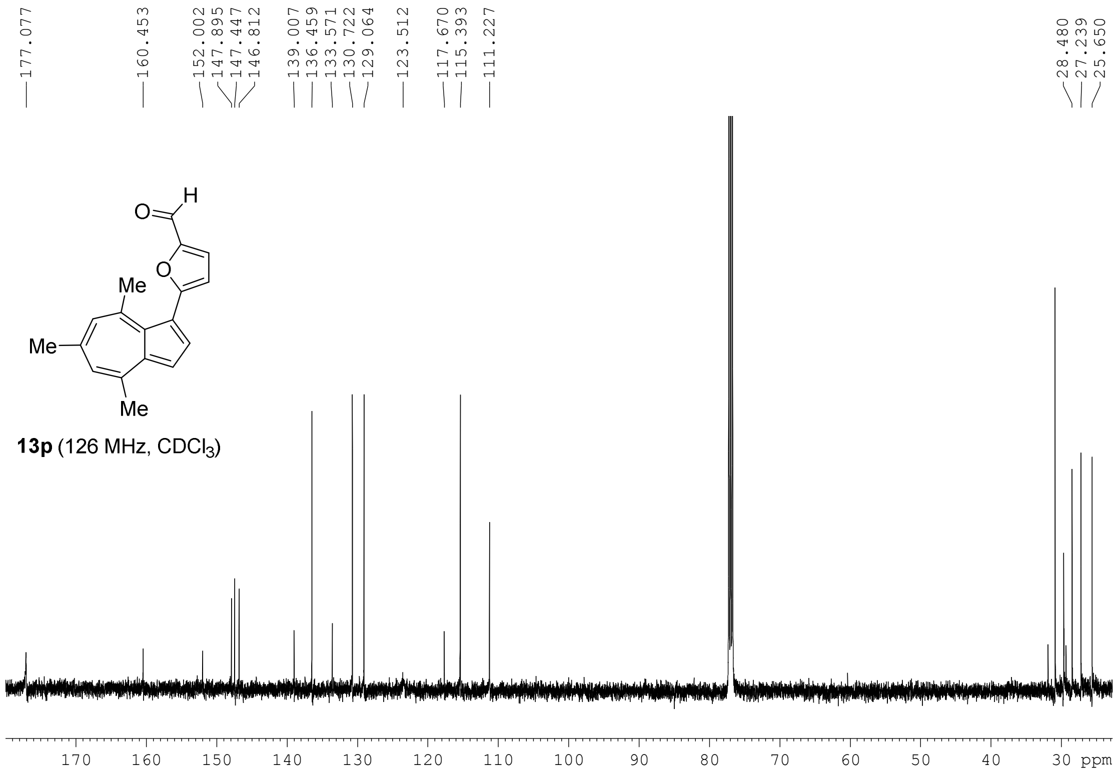

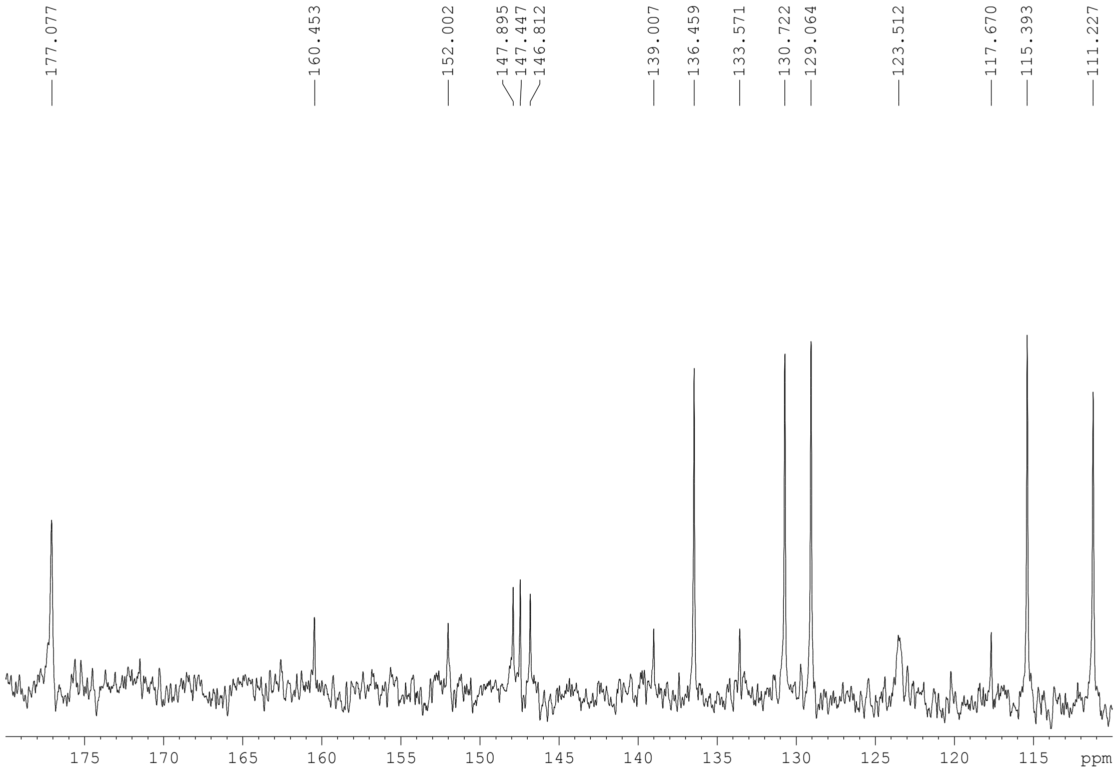

(line broadening = 10)

COSY

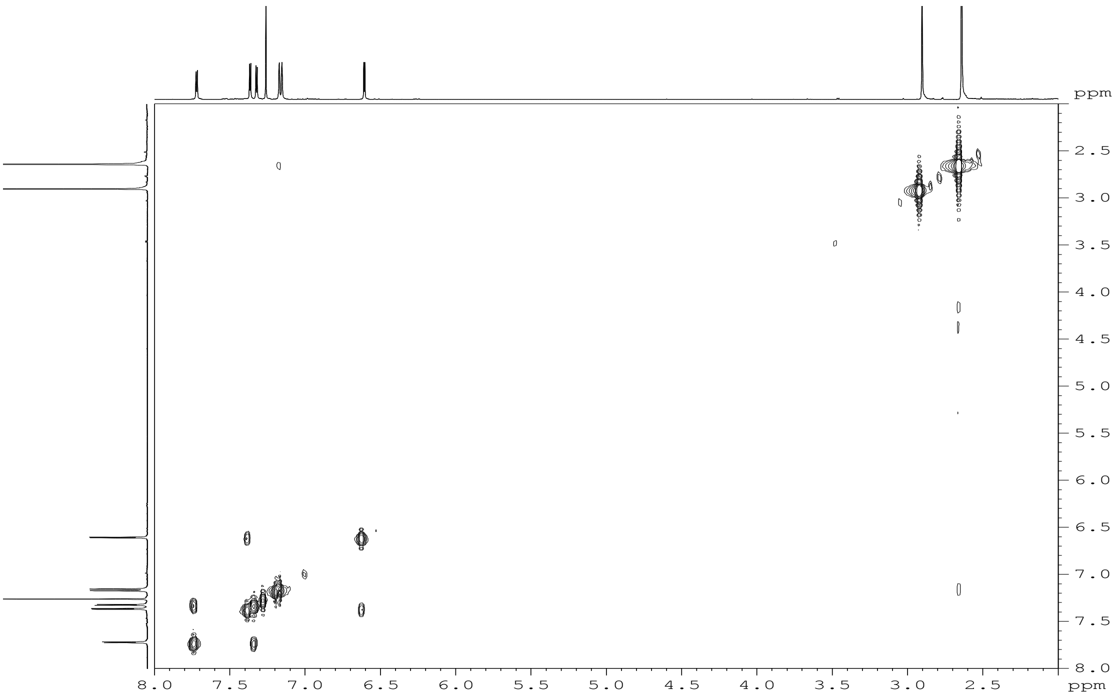

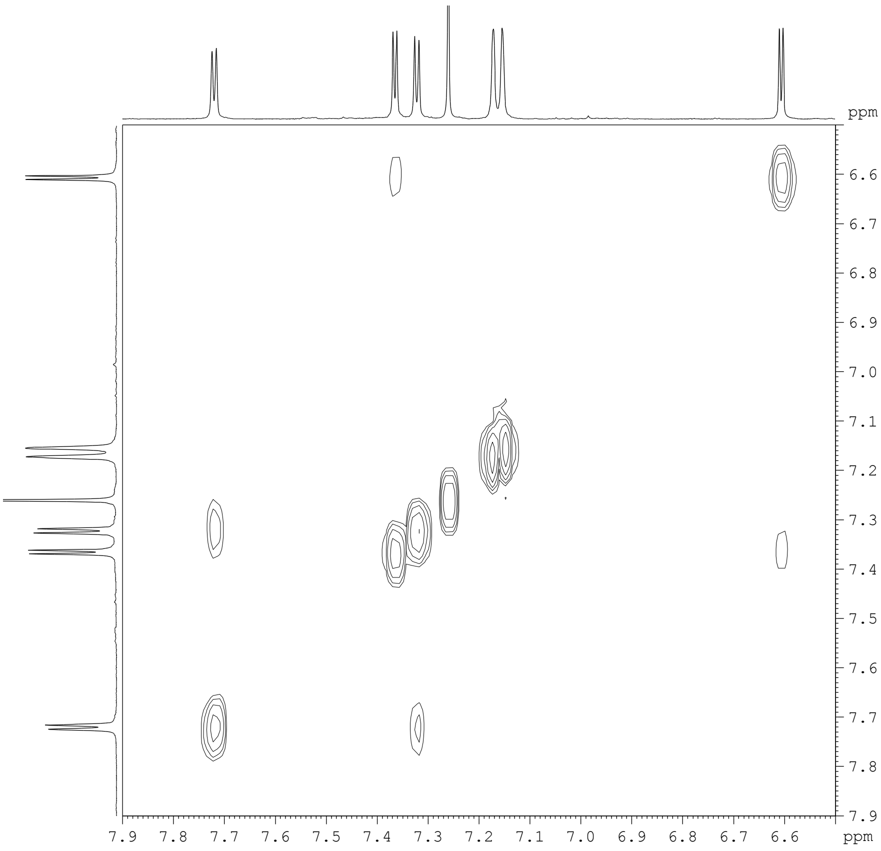

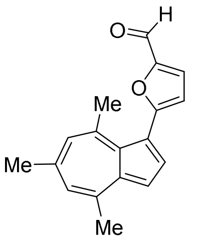

13p (500 MHz, $CDCl_3$)

NOESY

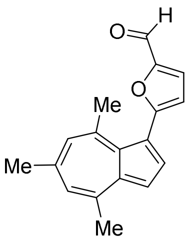

13p (500 MHz, $CDCl_3$)

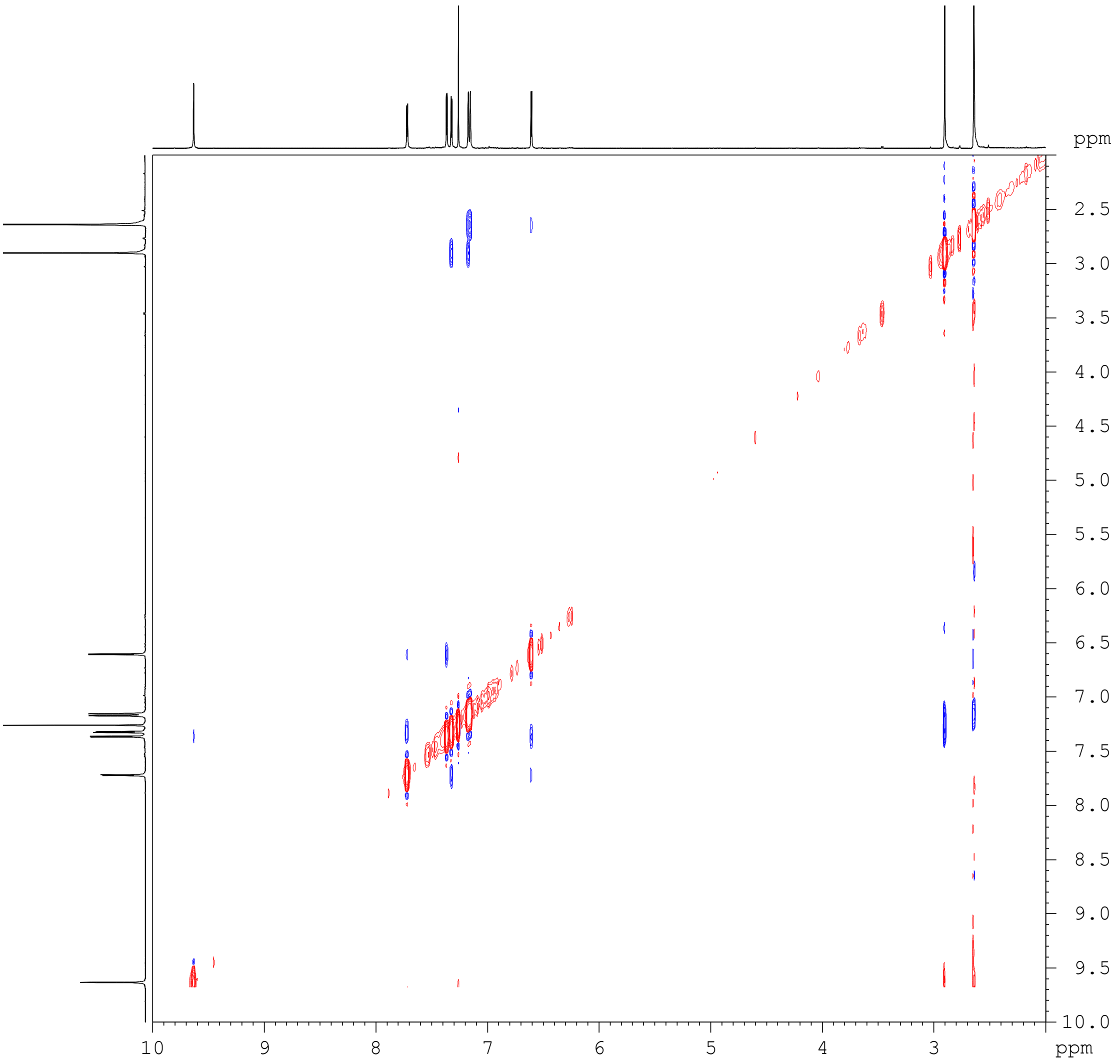

NOESY

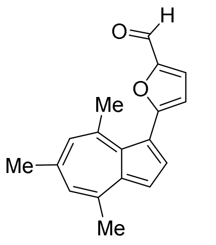

13p (500 MHz, $CDCl_3$)

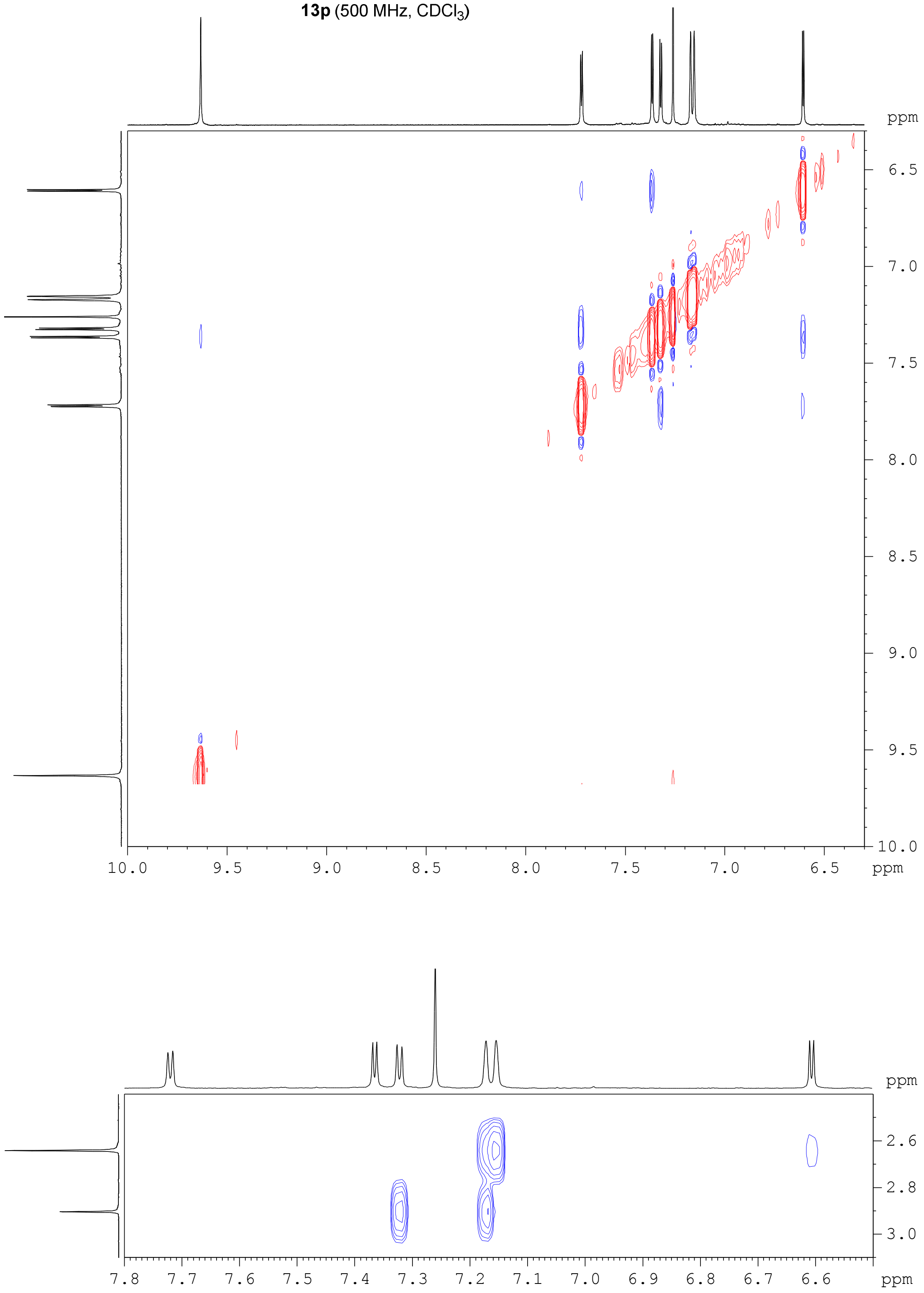

HSQC

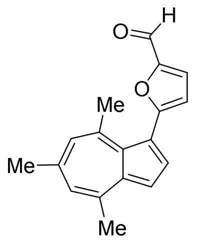

13p (500 MHz, $CDCl_3$)

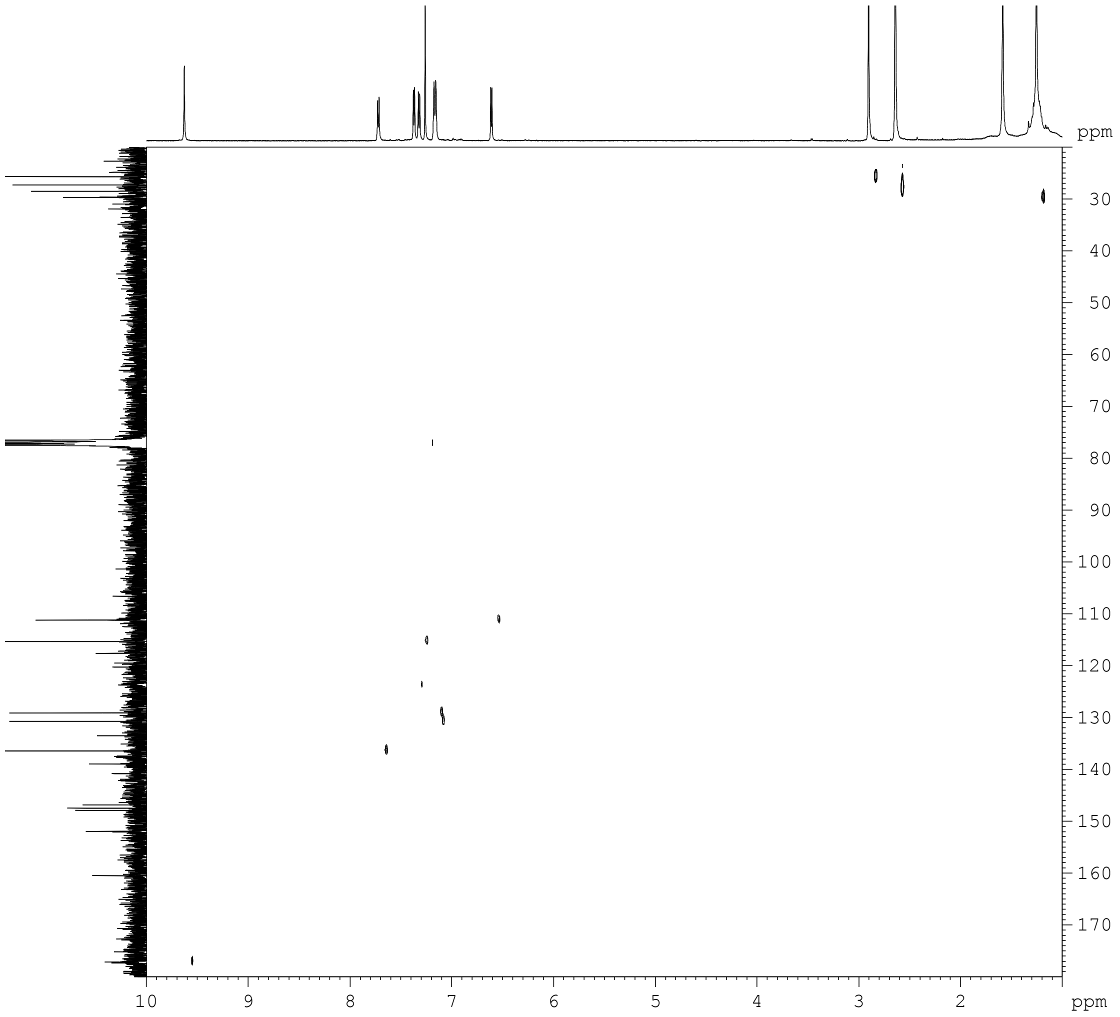

HSQC

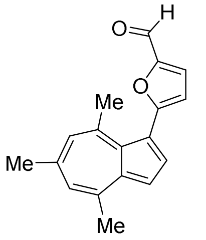

13p (500 MHz, $CDCl_3$)

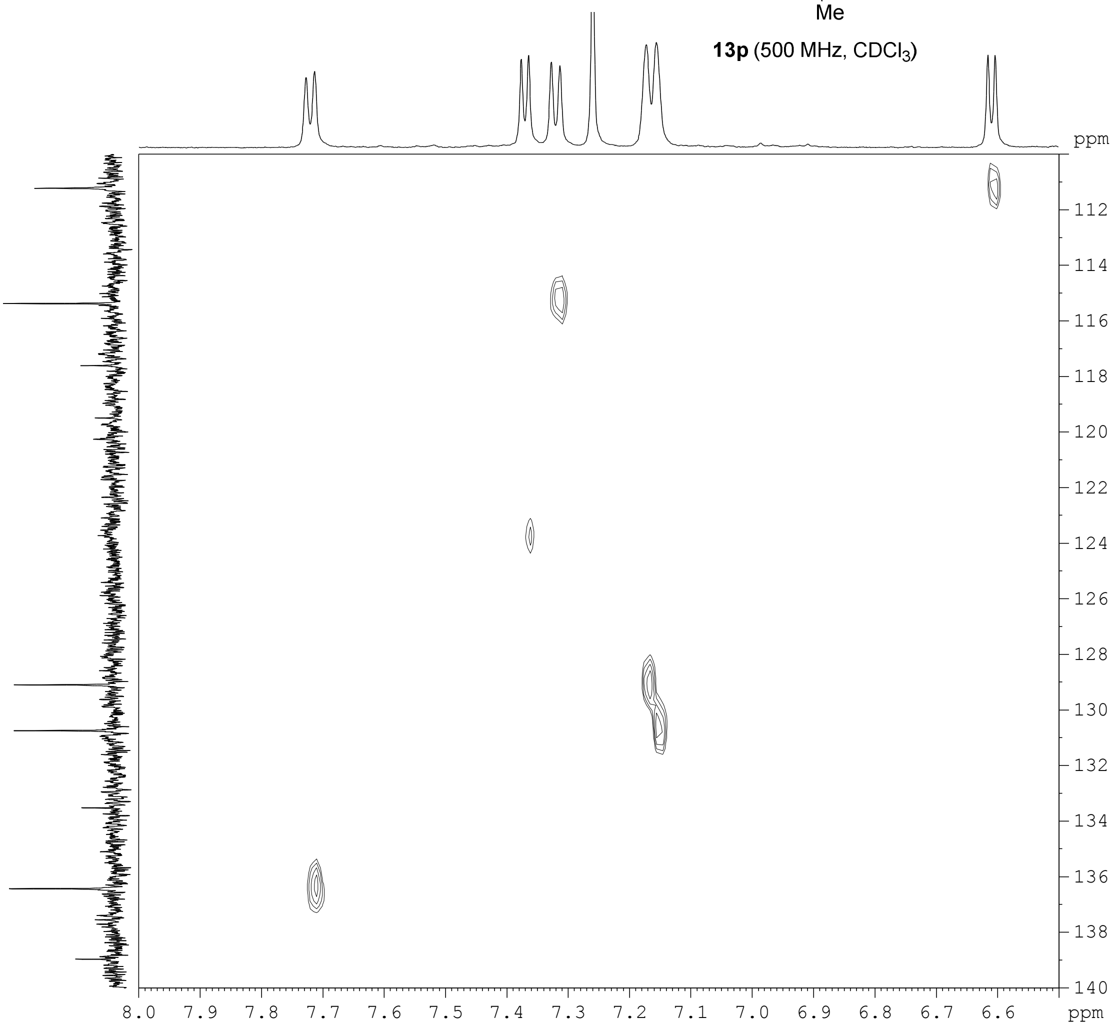

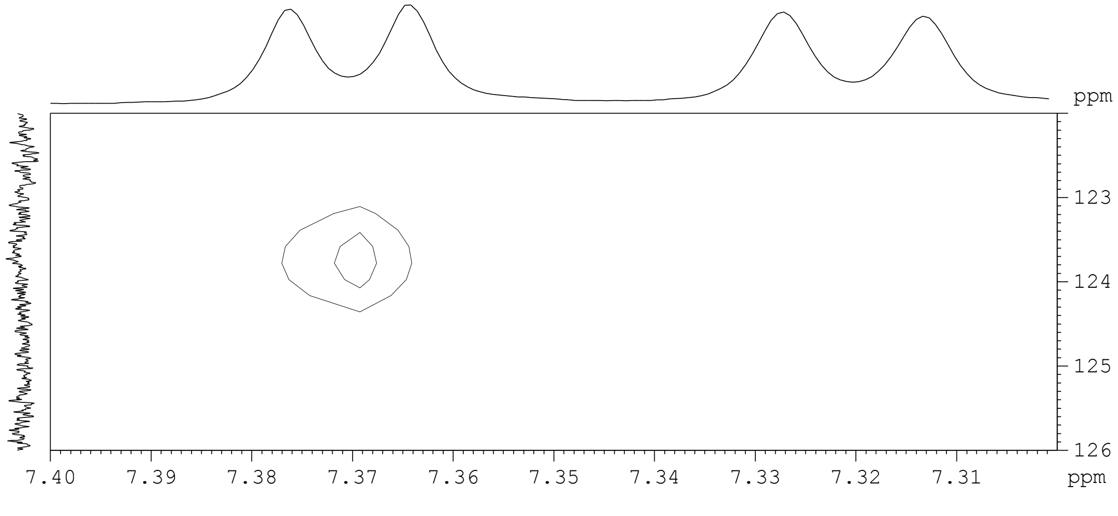

HMBC

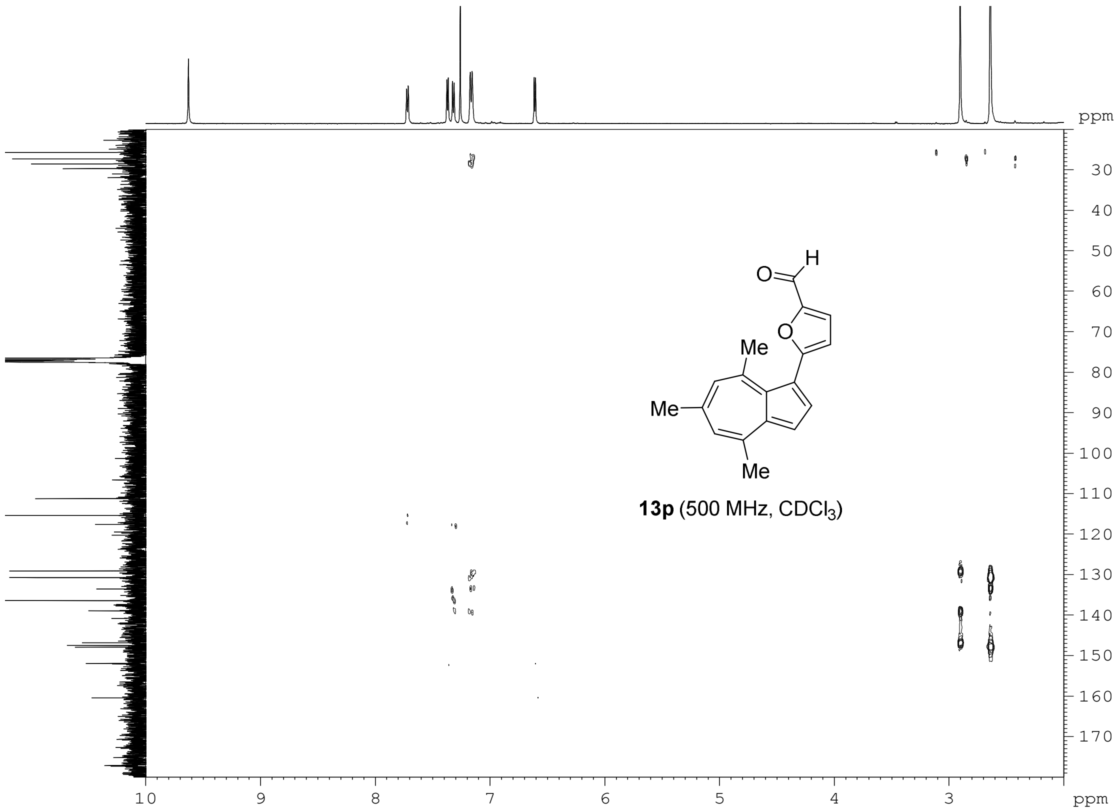

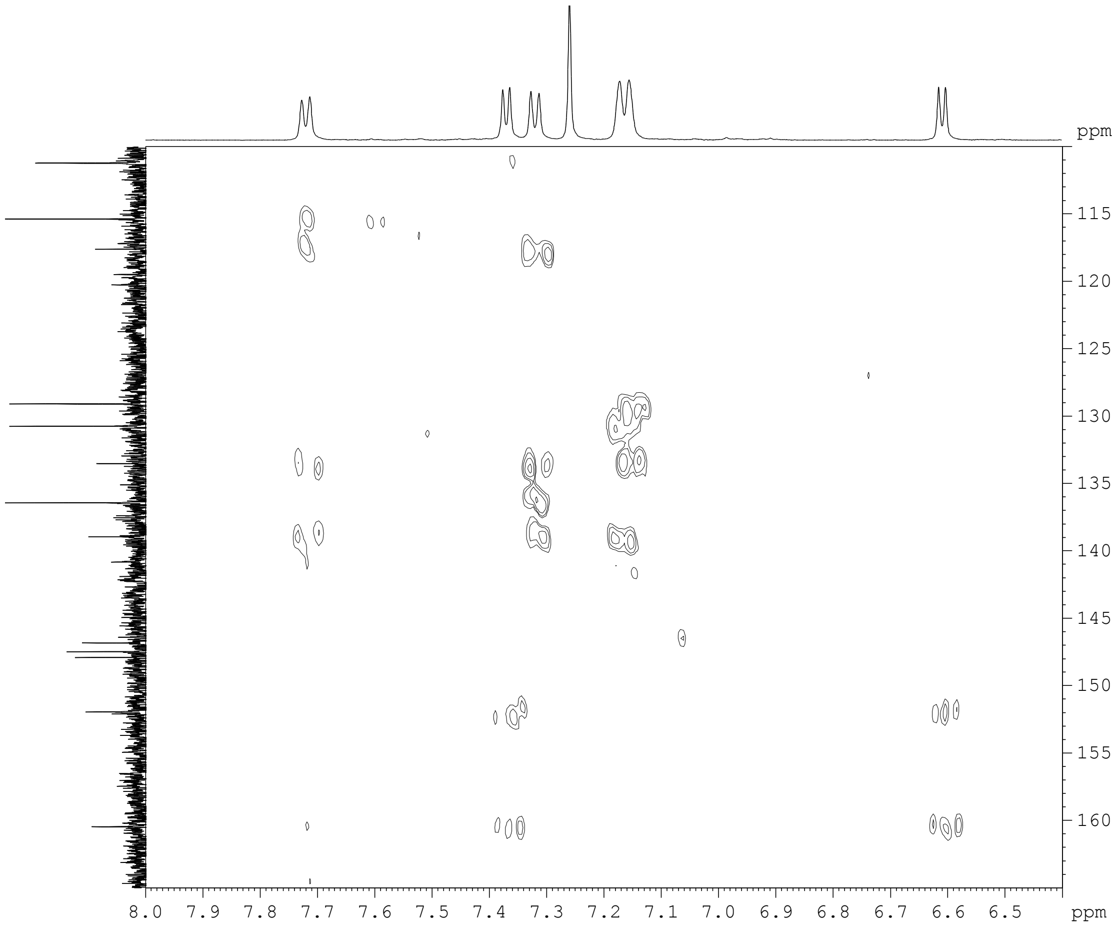

HMBC

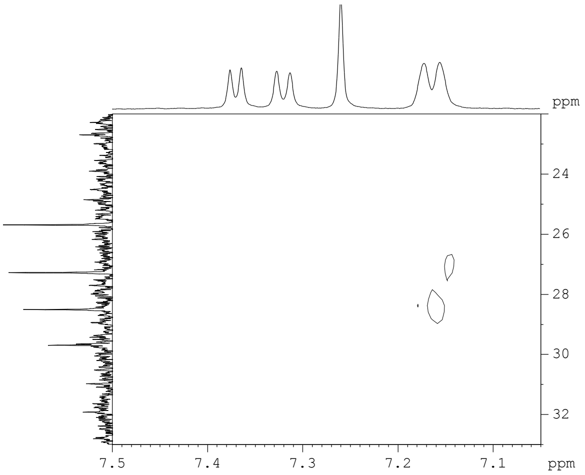

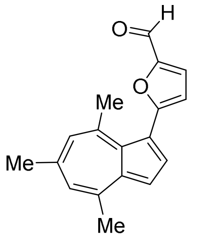

13p (500 MHz, $CDCl_3$)

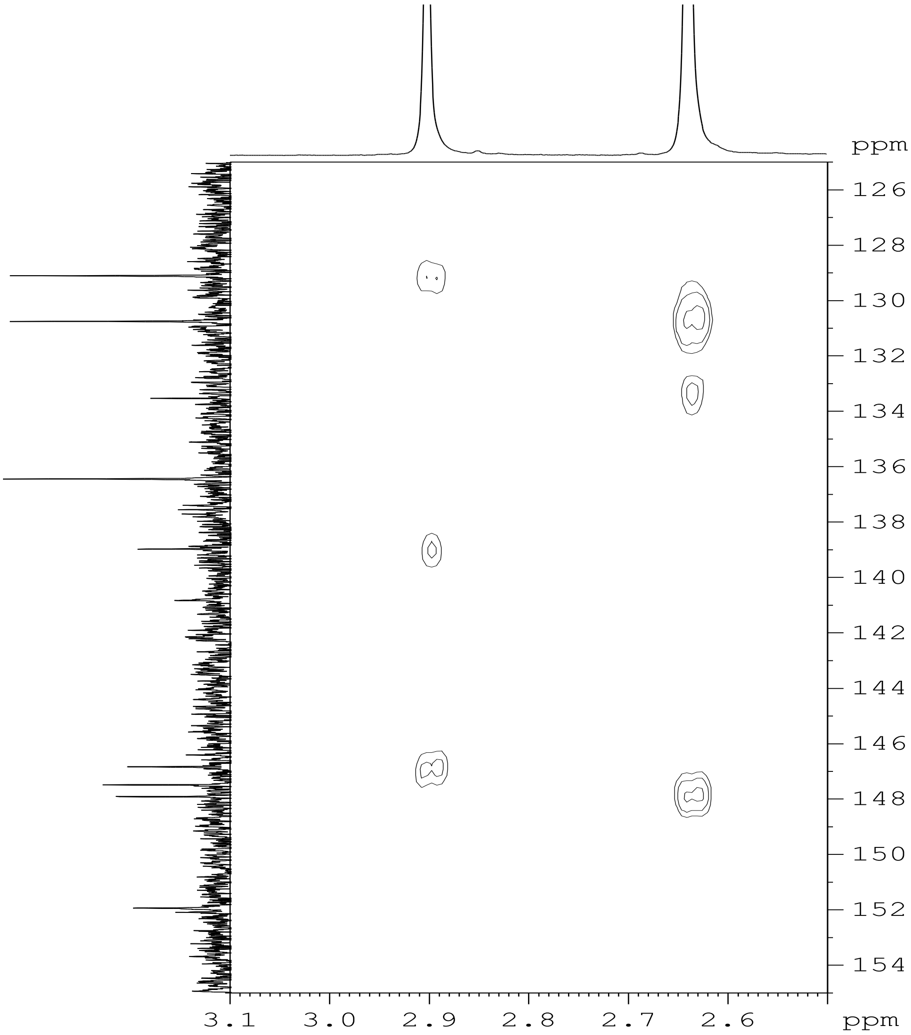

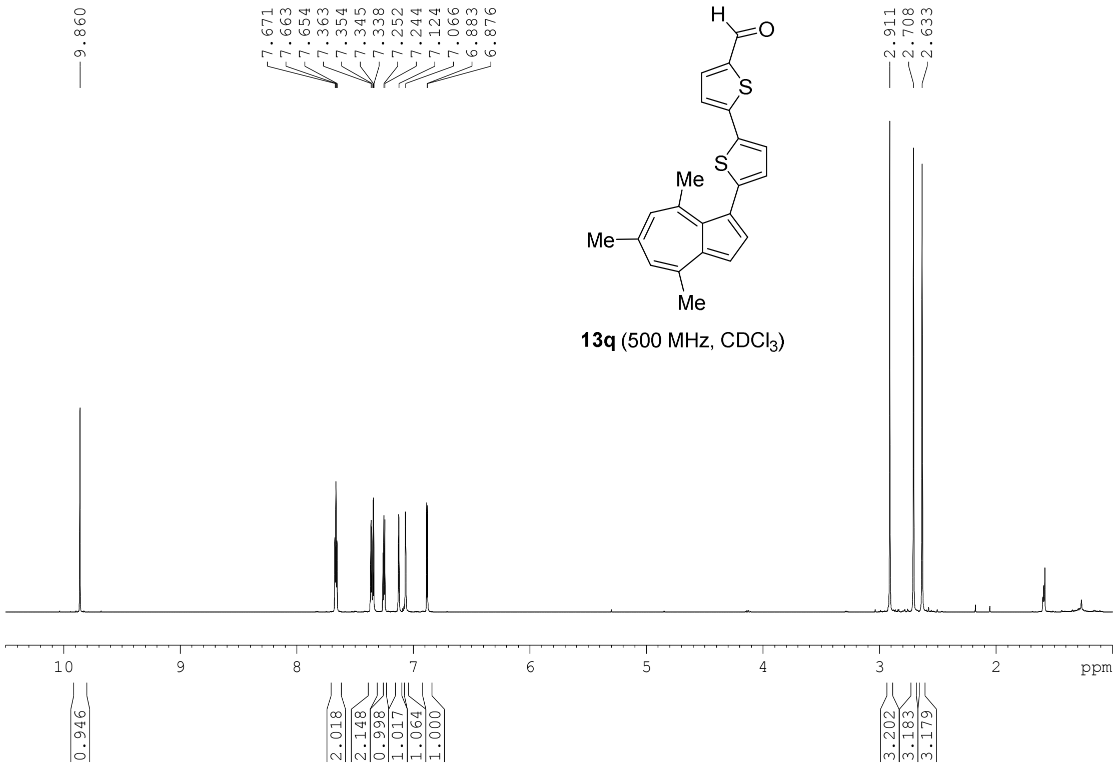
9.860
7.671 7.663 7.654 7.363 7.354 7.345 7.338 7.252 7.244 7.124 7.066 6.883 6.876
2.911 2.708 2.633
H
O
S
S
Me
Me
Me
13q (500 MHz, CDCl3)
10 9 8 7 6 5 4 3 2 ppm
0.946
2.018 2.148 0.998 1.017 1.064 1.000
3.202 3.183 3.179

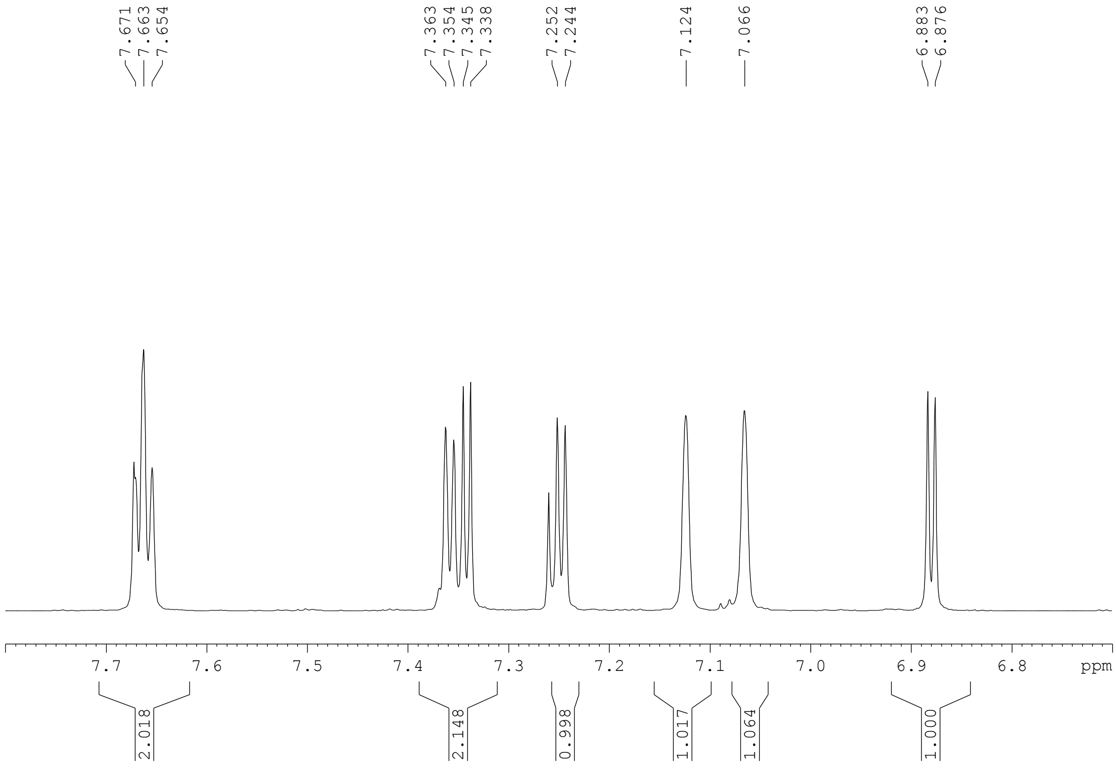
7.671 7.663 7.654
7.363 7.354 7.345 7.338
7.252 7.244
7.124
7.066
6.883 6.876
7.7 7.6 7.5 7.4 7.3 7.2 7.1 7.0 6.9 6.8 ppm
2.018
2.148
0.998
1.017
1.064
1.000

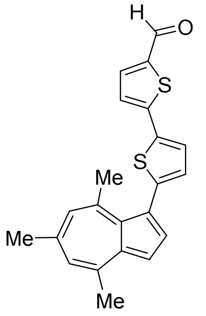

13q (126 MHz, $CDCl_3$)

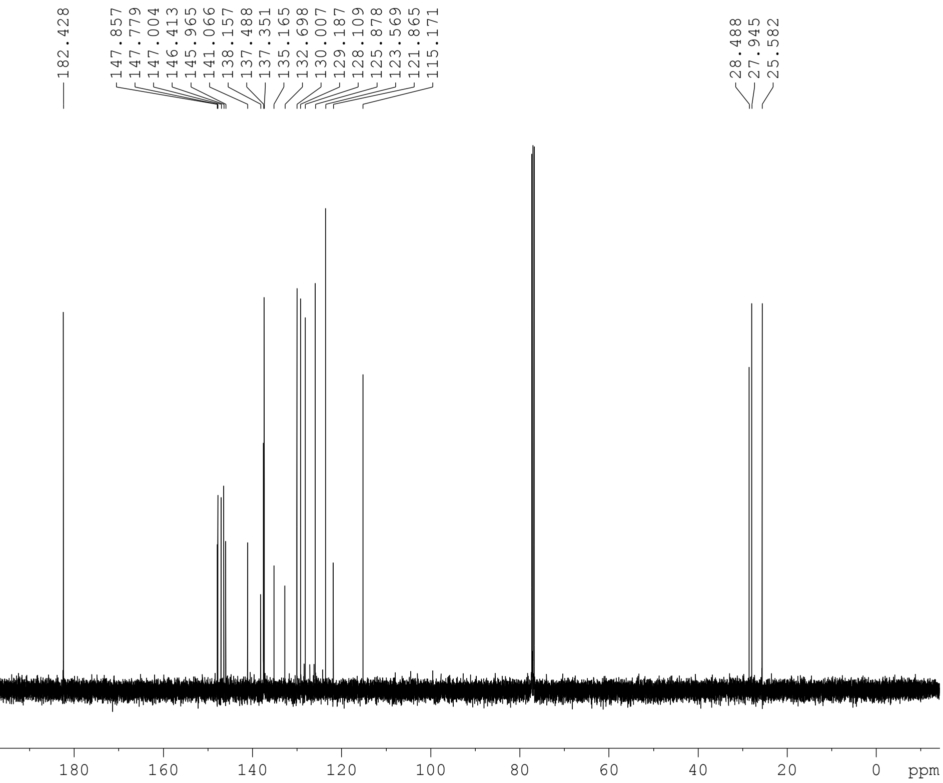

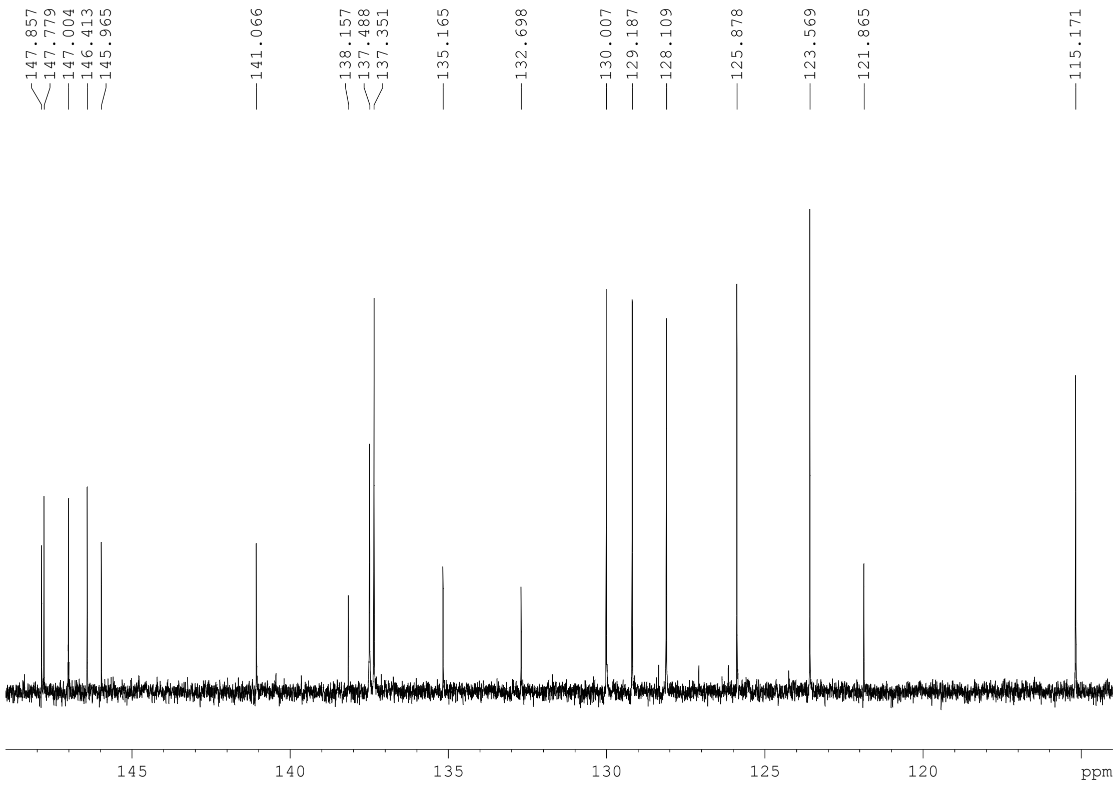

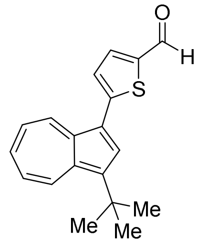

13r (500MHz, $CDCl_3$)

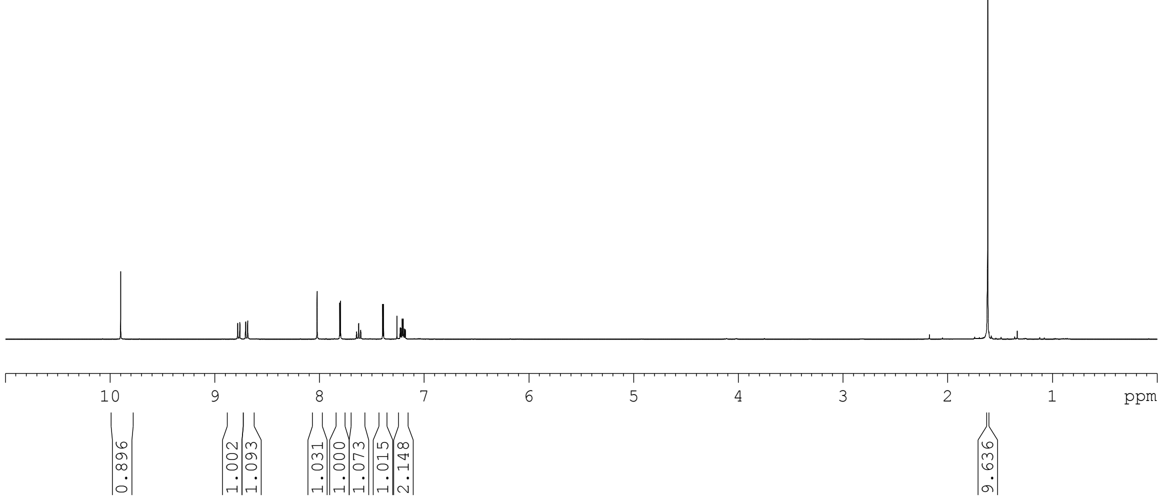

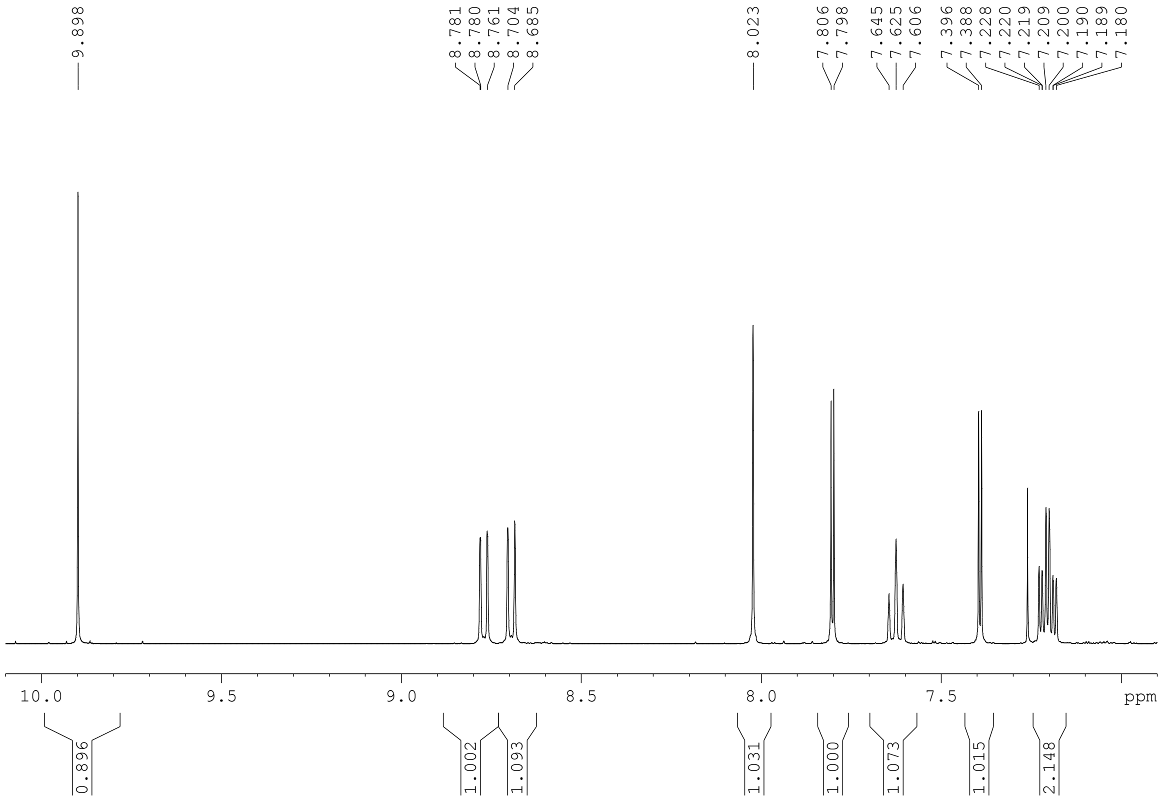

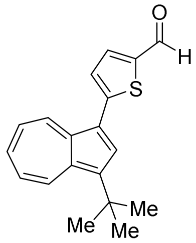

13r (126MHz, $CDCl_3$)

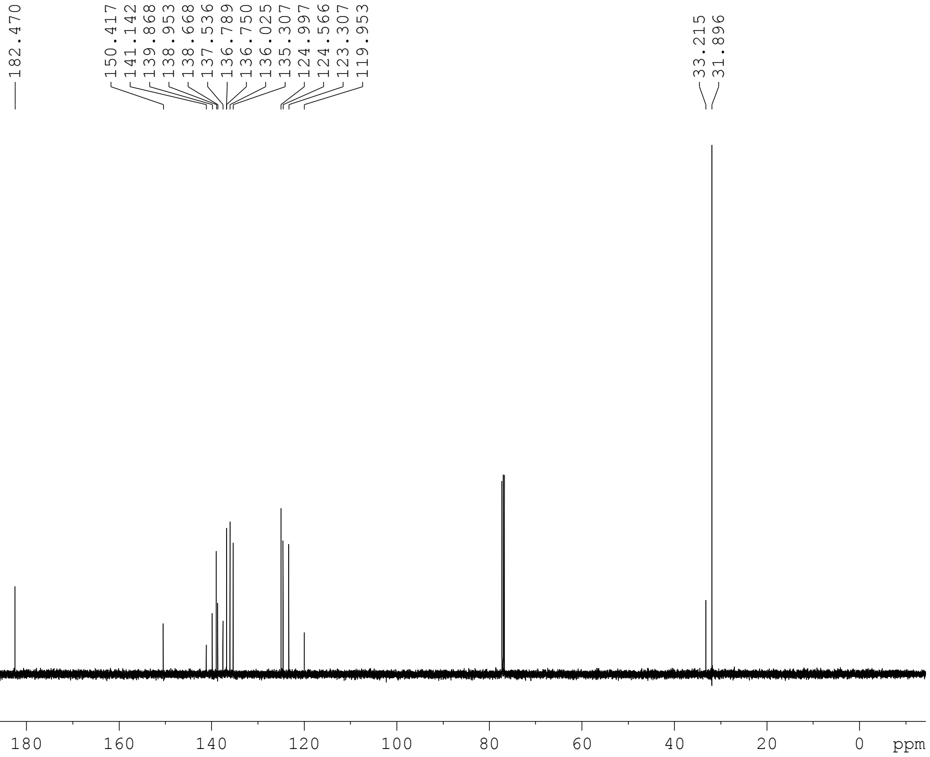

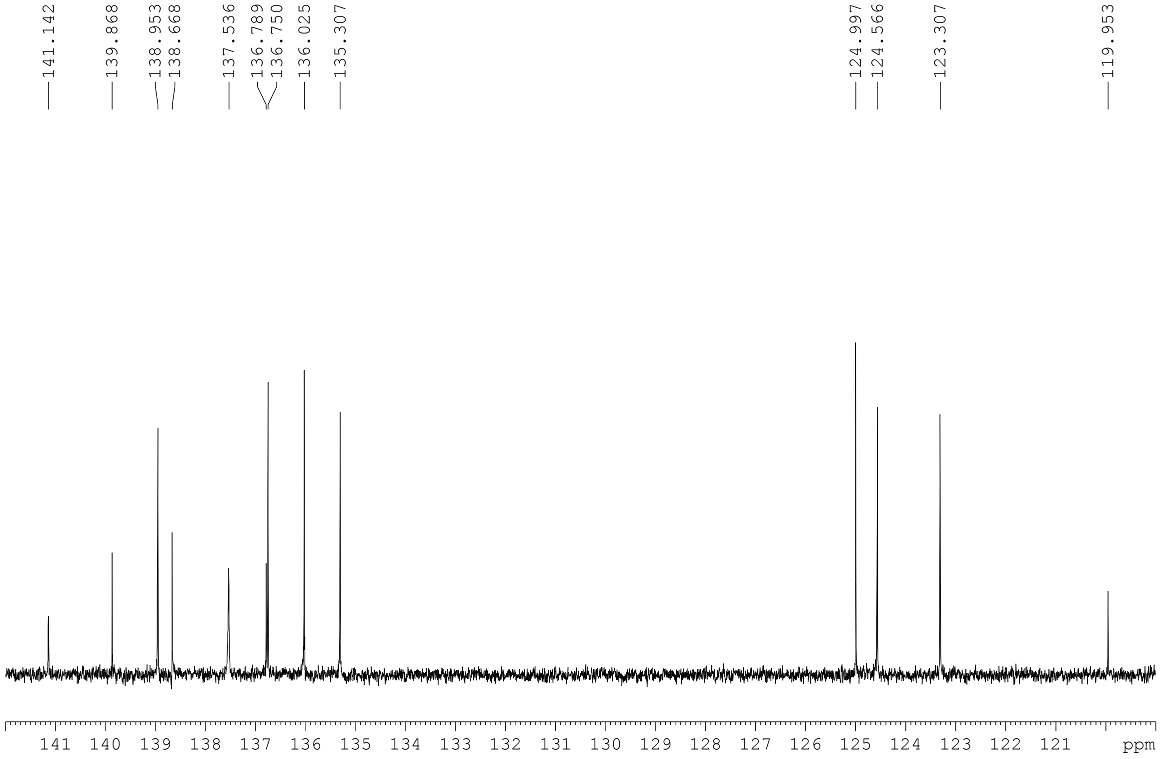

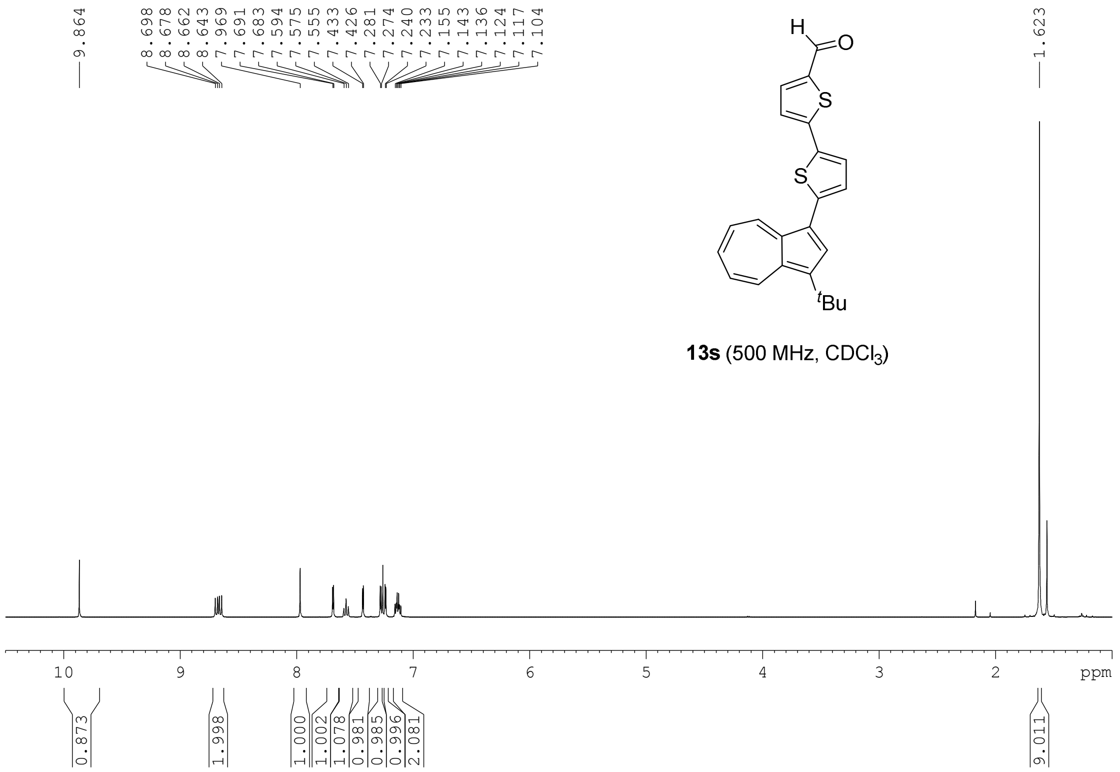

13s (500 MHz, $CDCl_3$)

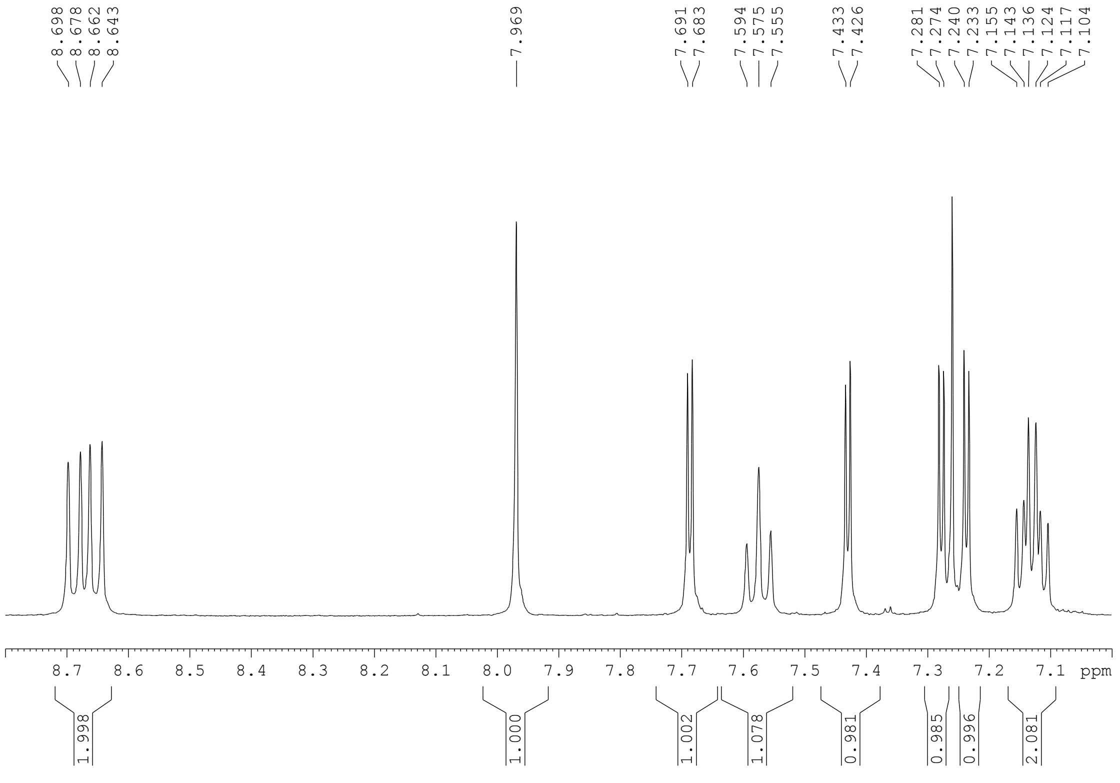

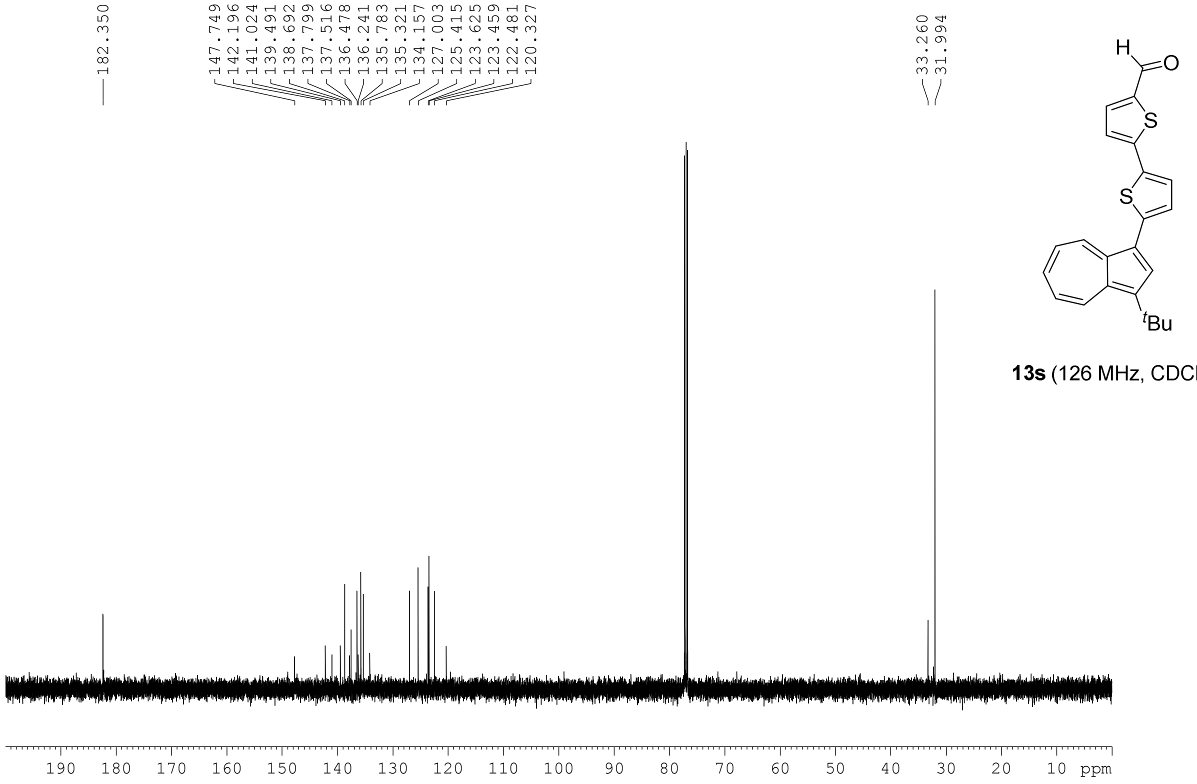

13s (126 MHz, $CDCl_3$)

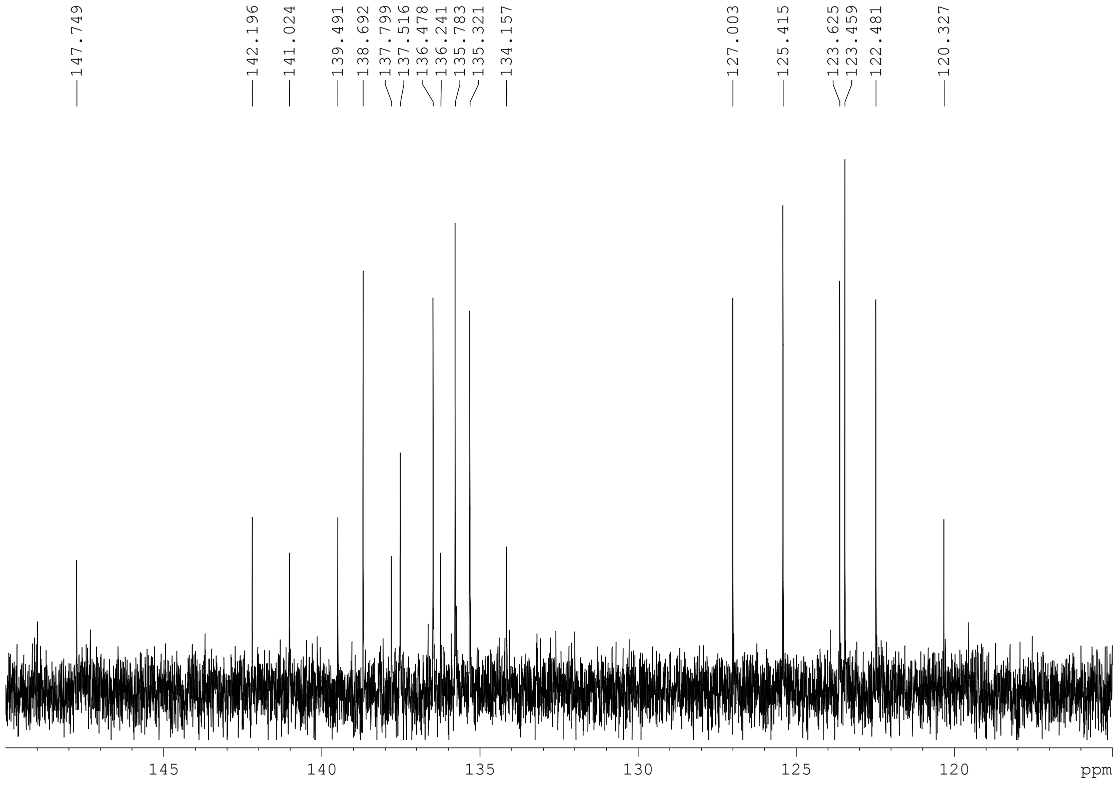

NOESY

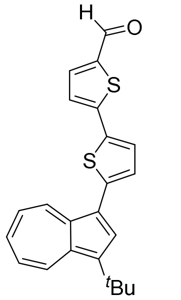

13s (500 MHz, $CDCl_3$)

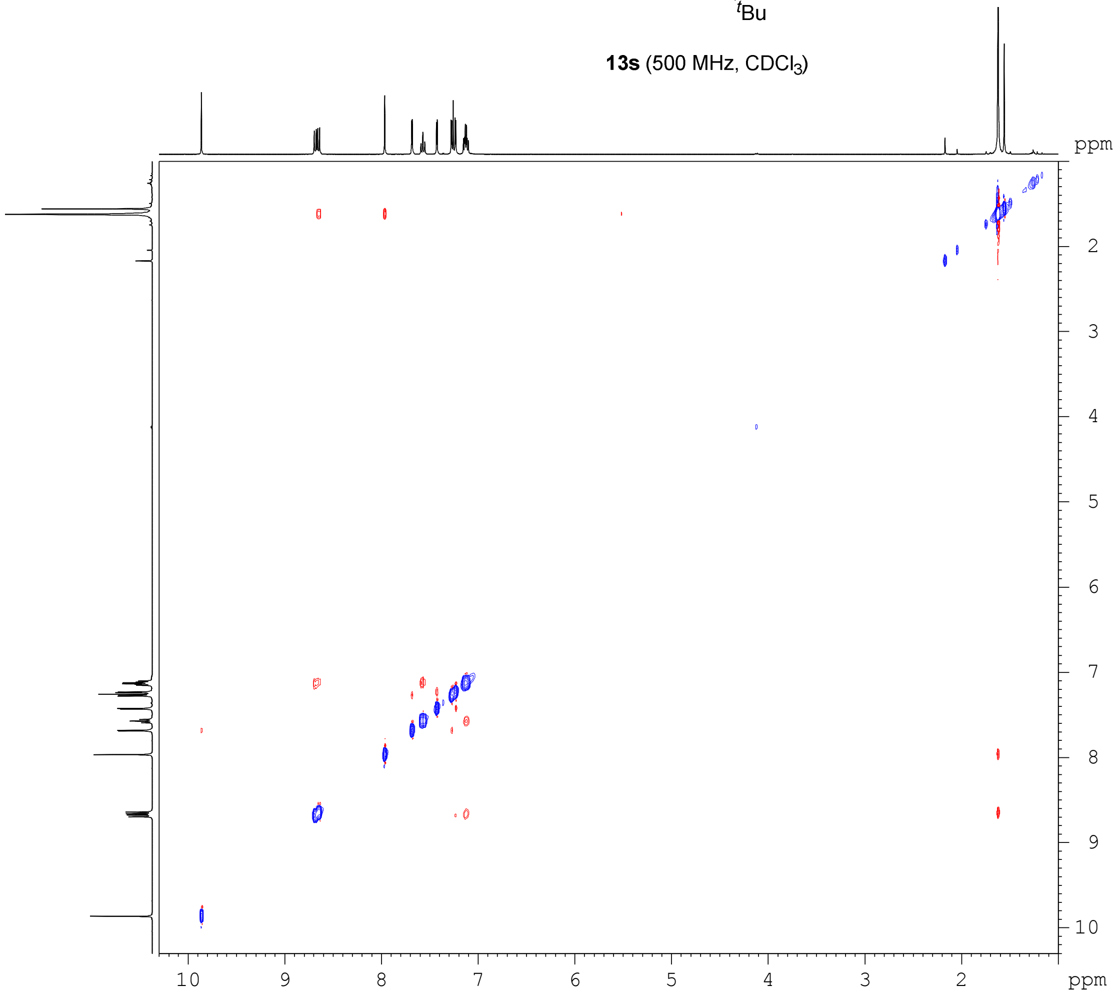

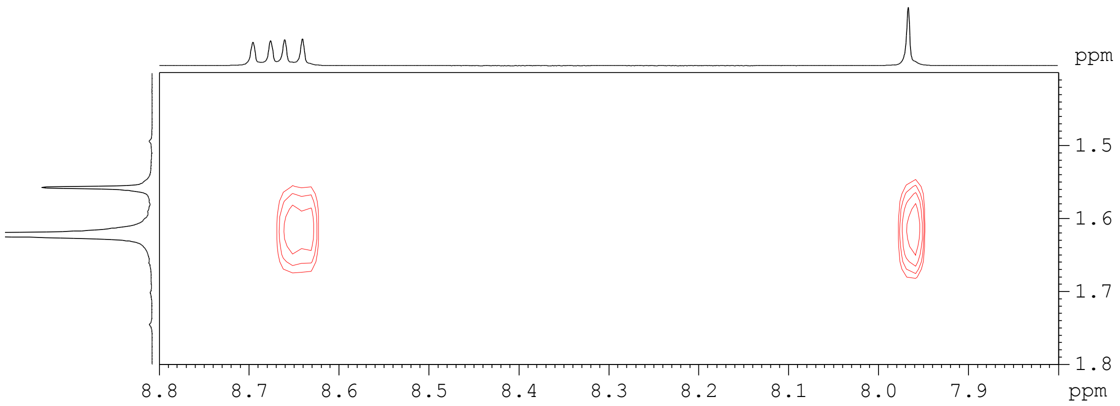

NOESY

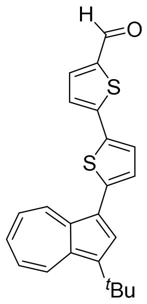

13s (500 MHz, $CDCl_3$)

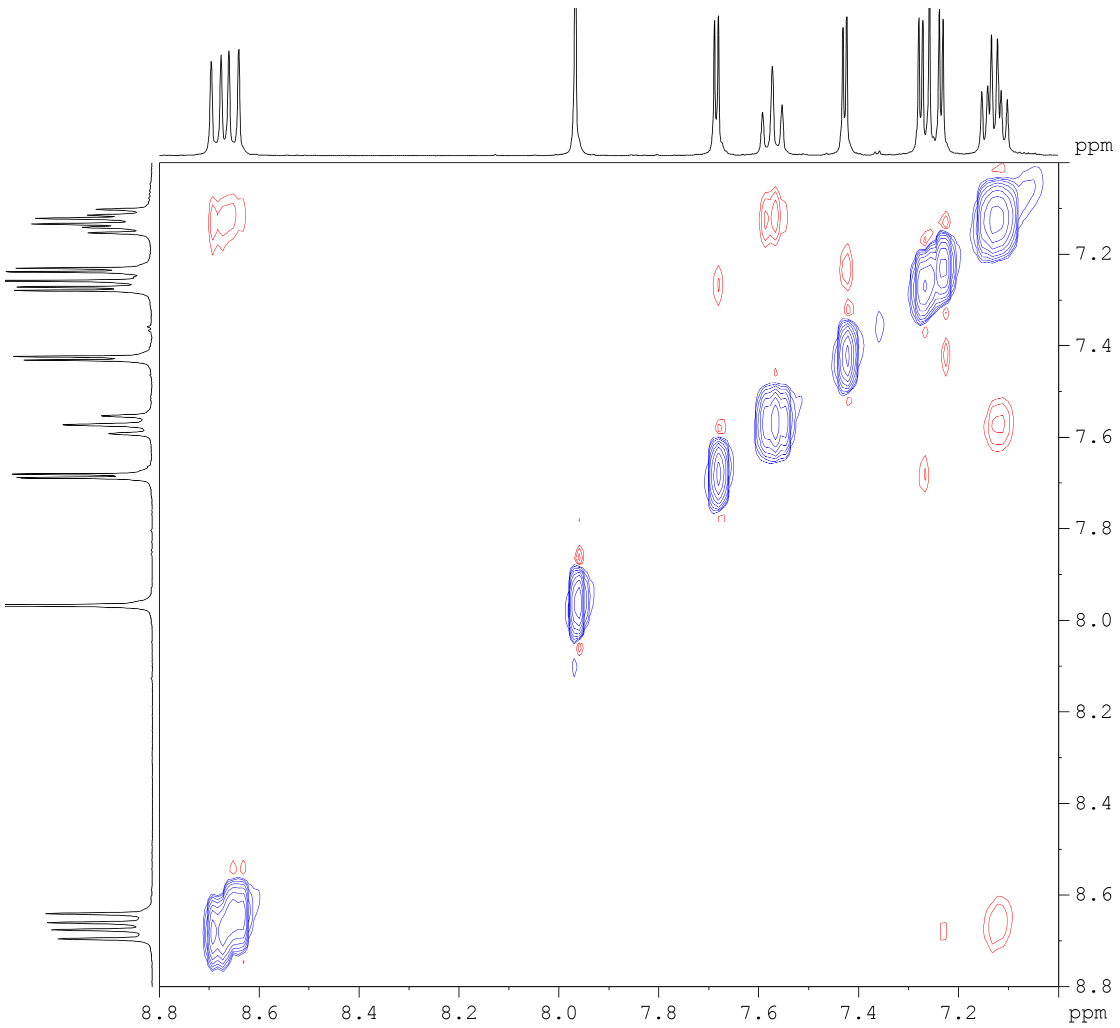

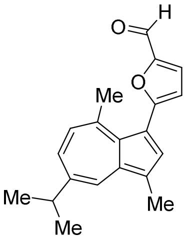

13t (400 MHz, $CDCl_3$)

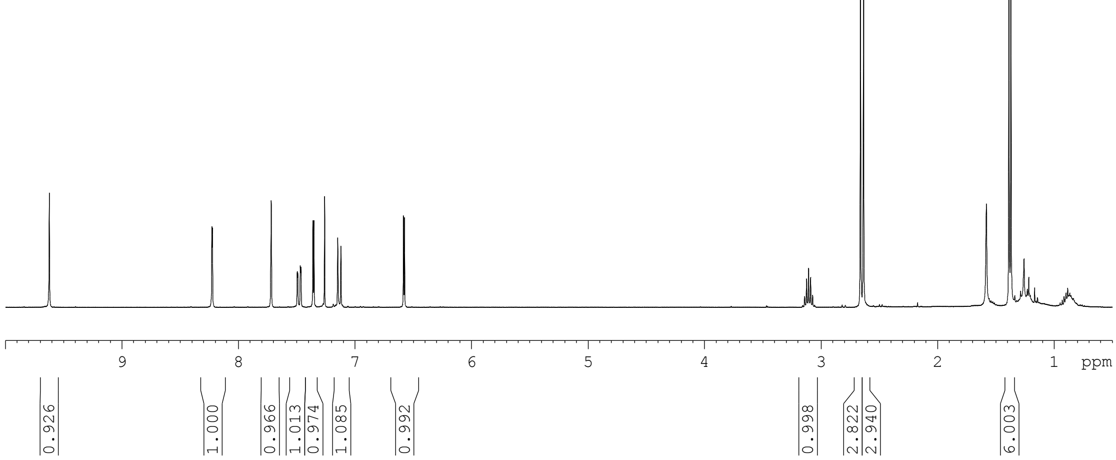

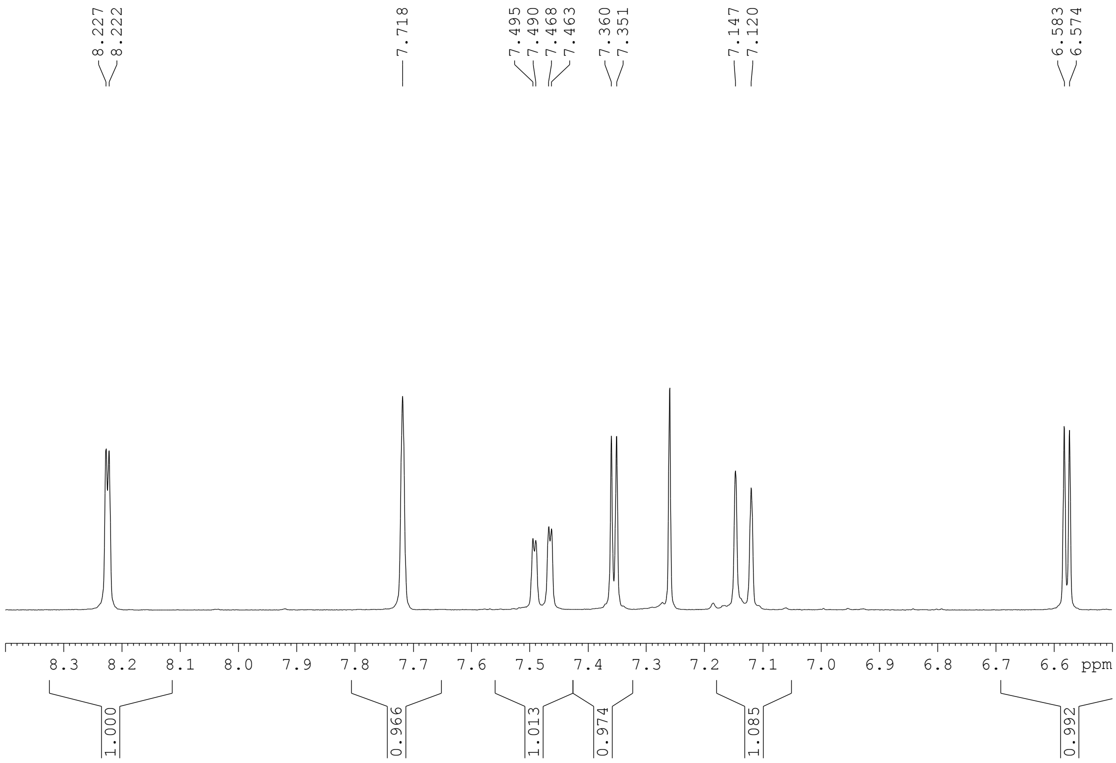

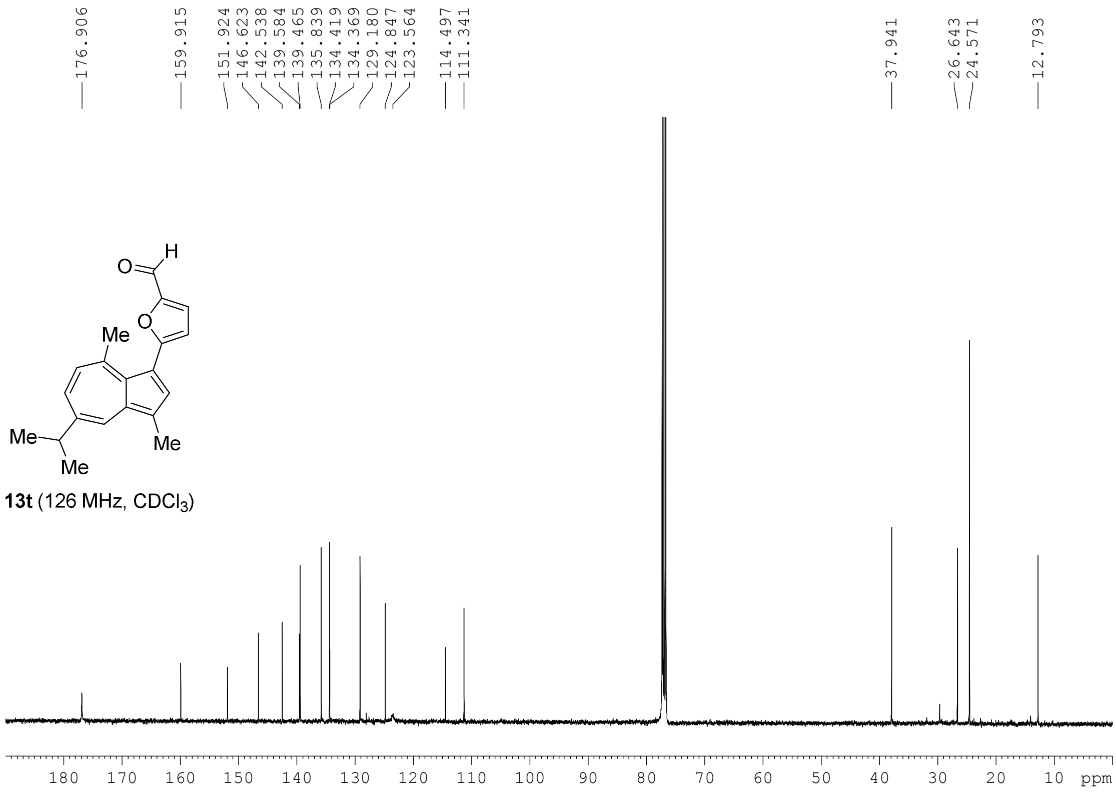

13t (126 MHz, $CDCl_3$)

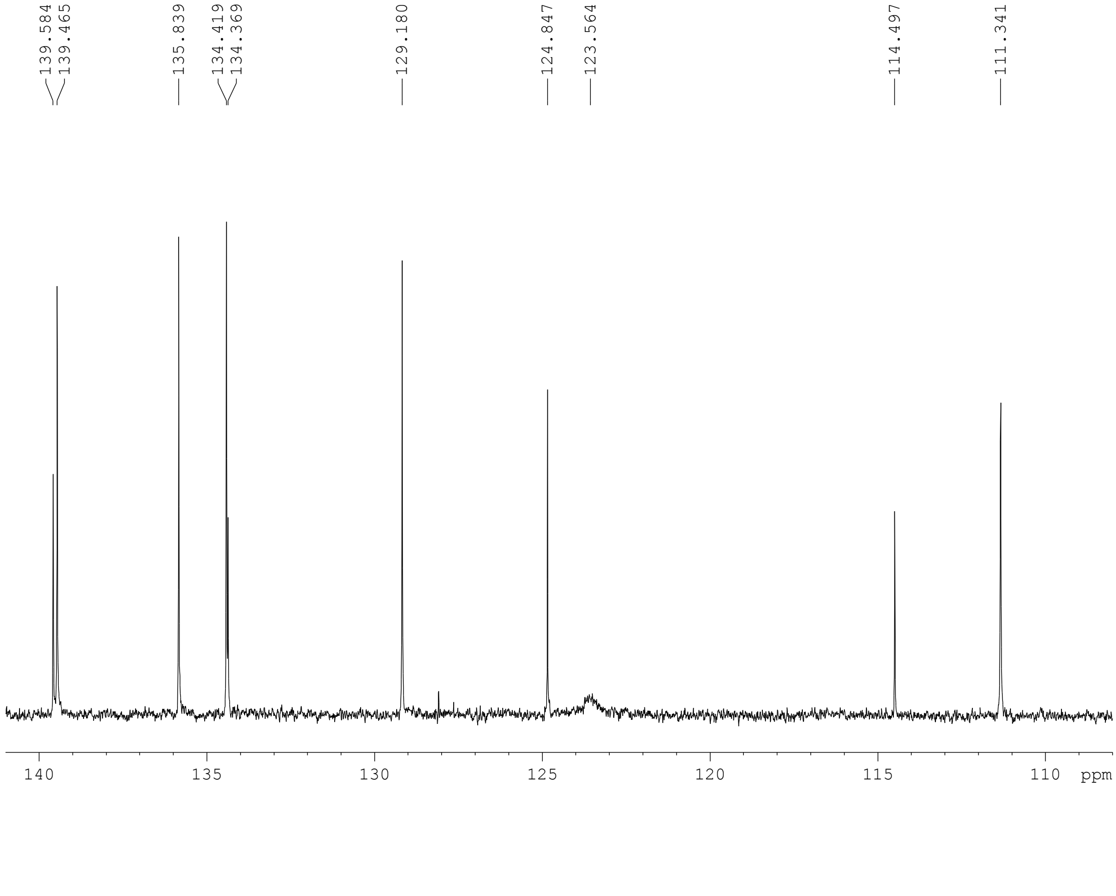

COSY

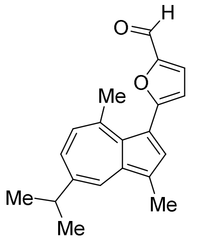

13t (400 MHz, $CDCl_3$)

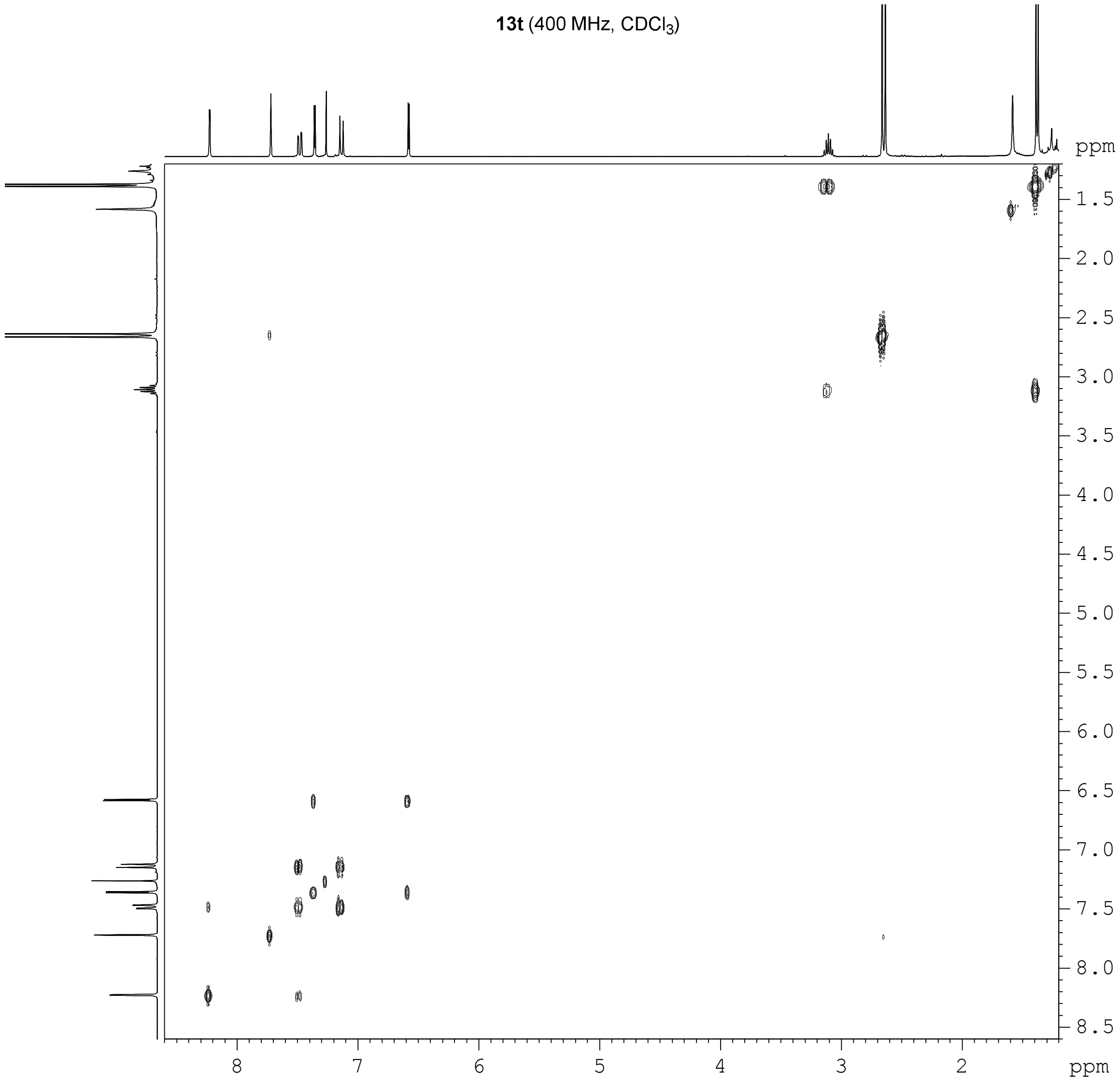

COSY

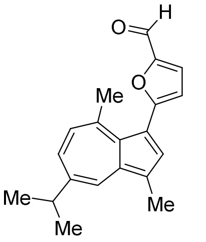

13t (400 MHz, $CDCl_3$)

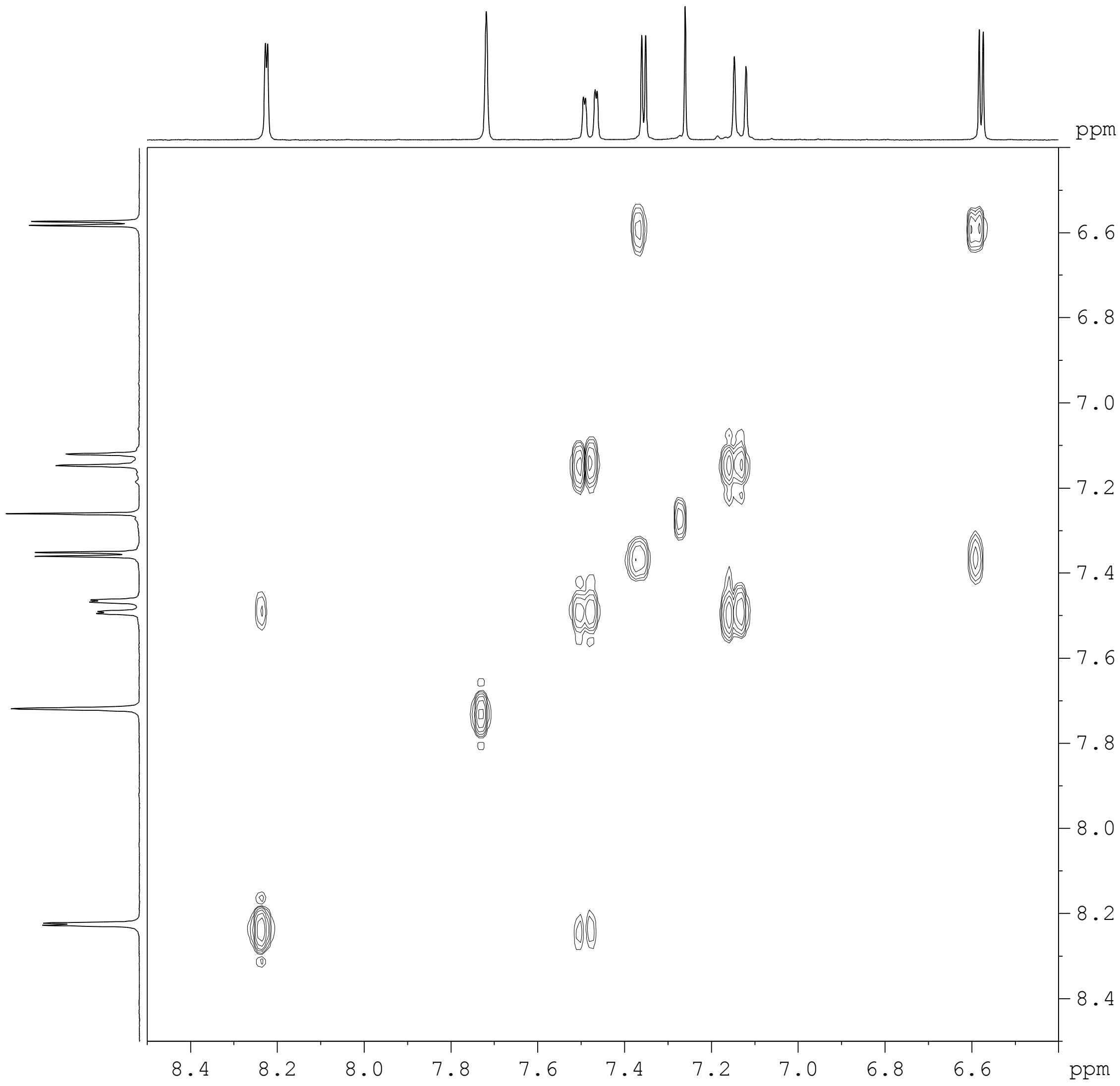

NOESY

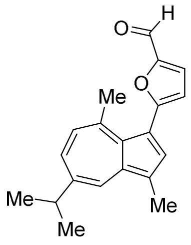

13t (400 MHz, $CDCl_3$)

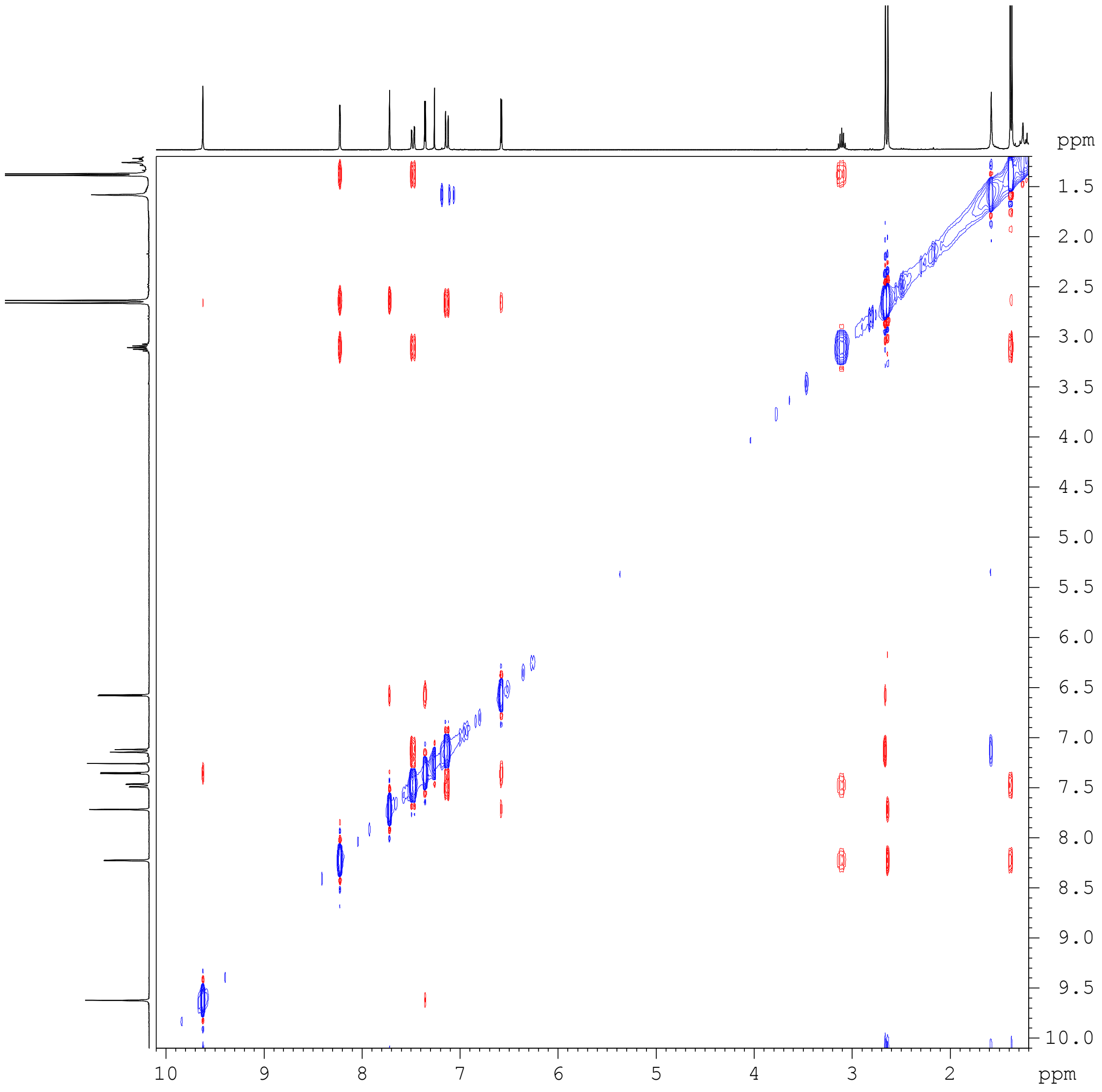

NOESY

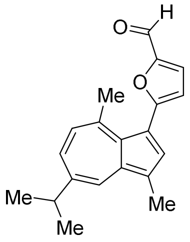

13t (400 MHz, $CDCl_3$)

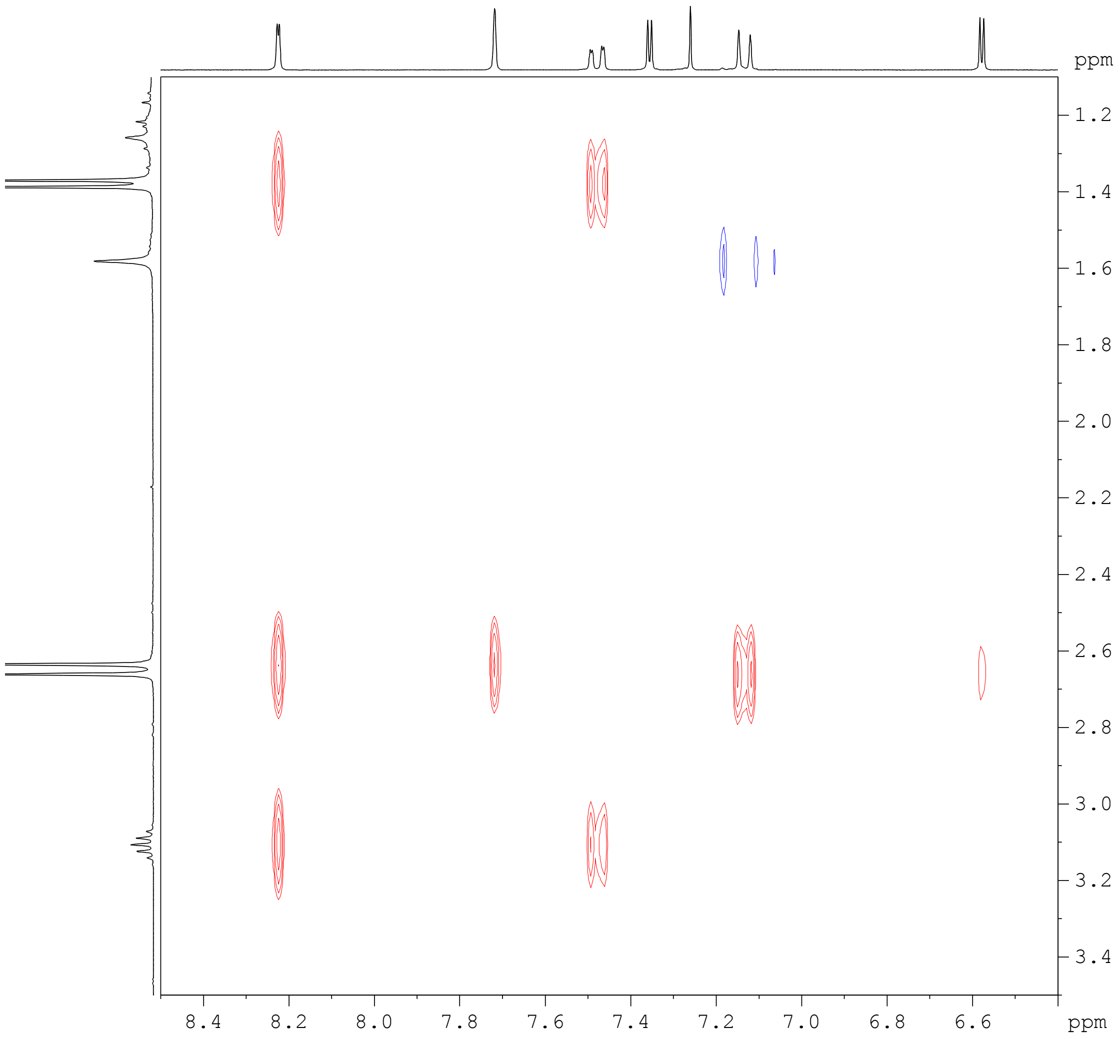

HSQC

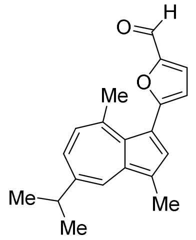

13t (400 MHz, $CDCl_3$)

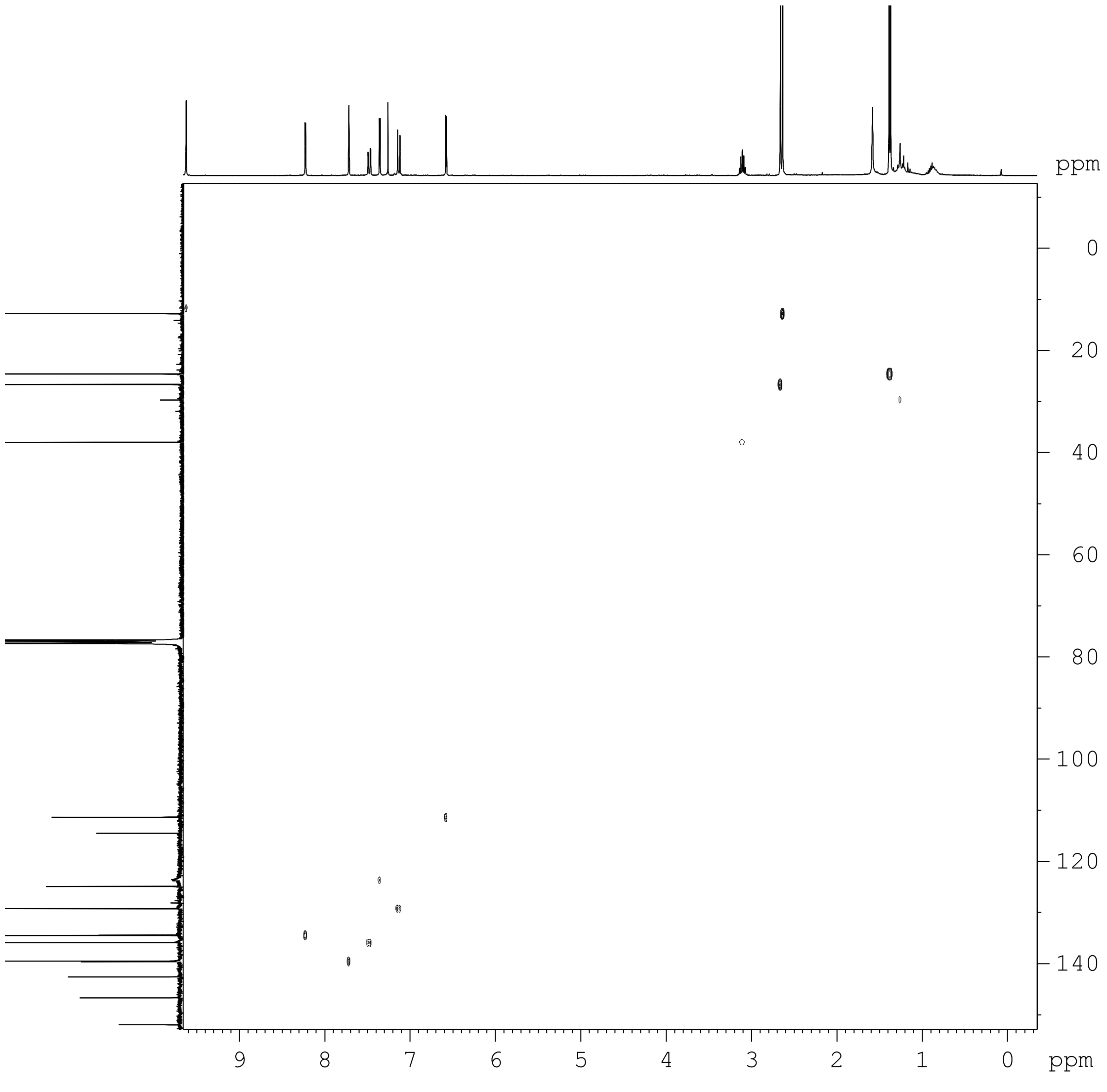

HSQC

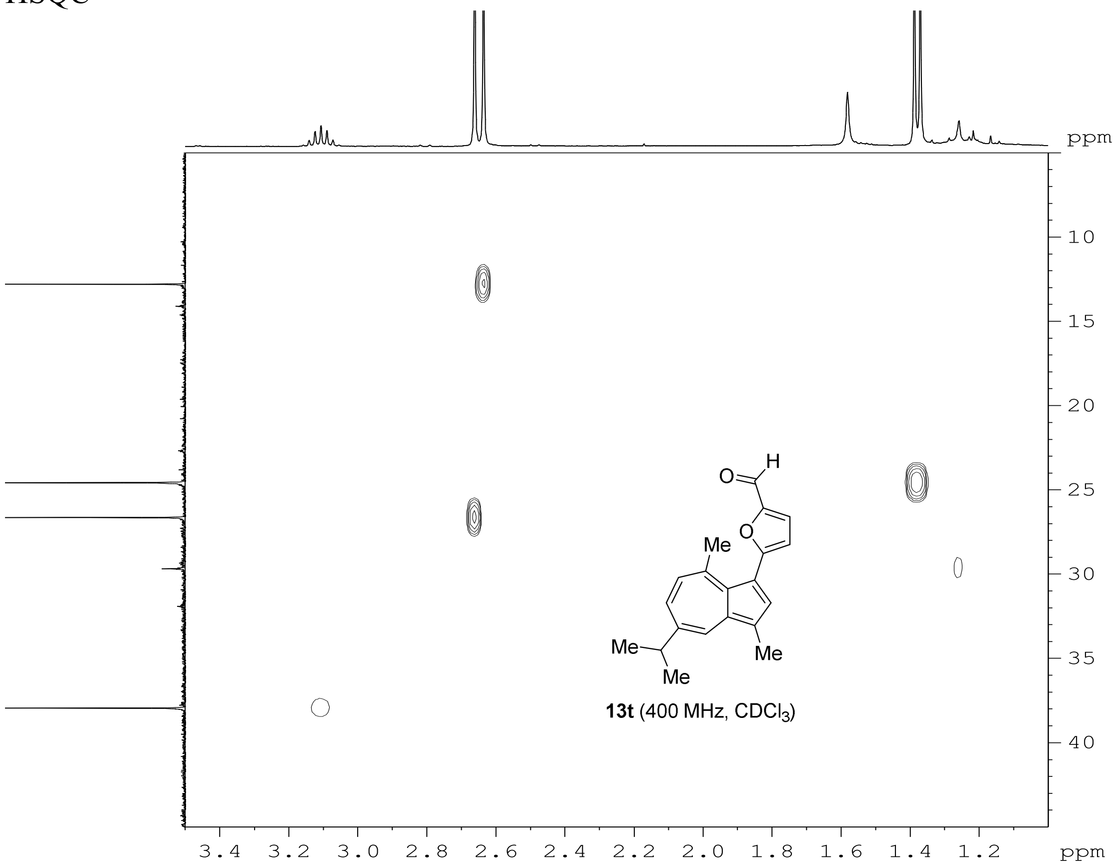
ppm
10
15
20
25
30
35
40
3.4 3.2 3.0 2.8 2.6 2.4 2.2 2.0 1.8 1.6 1.4 1.2
ppm
O
H
O
Me
Me
Me
Me
13t (400 MHz, CDCl3)

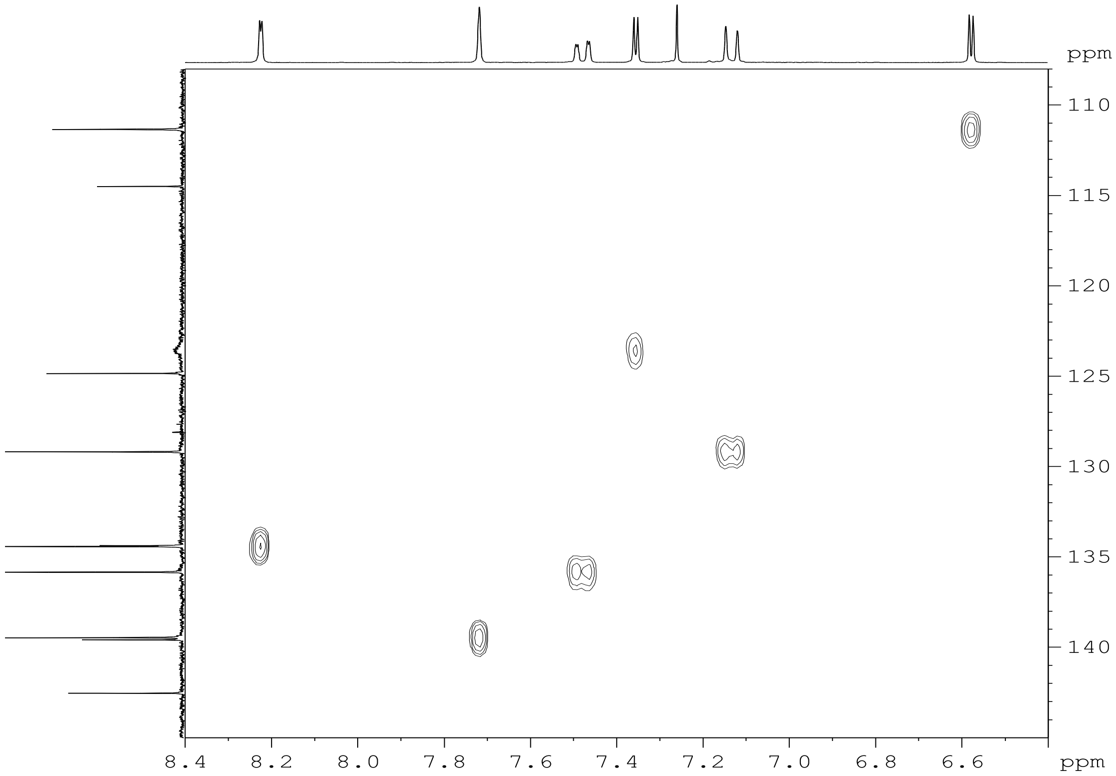
ppm
110
115
120
125
130
135
140
8.4 8.2 8.0 7.8 7.6 7.4 7.2 7.0 6.8 6.6
ppm

HMBC

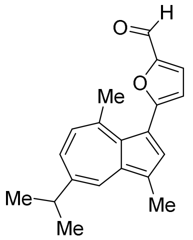

13t (400 MHz, $CDCl_3$)

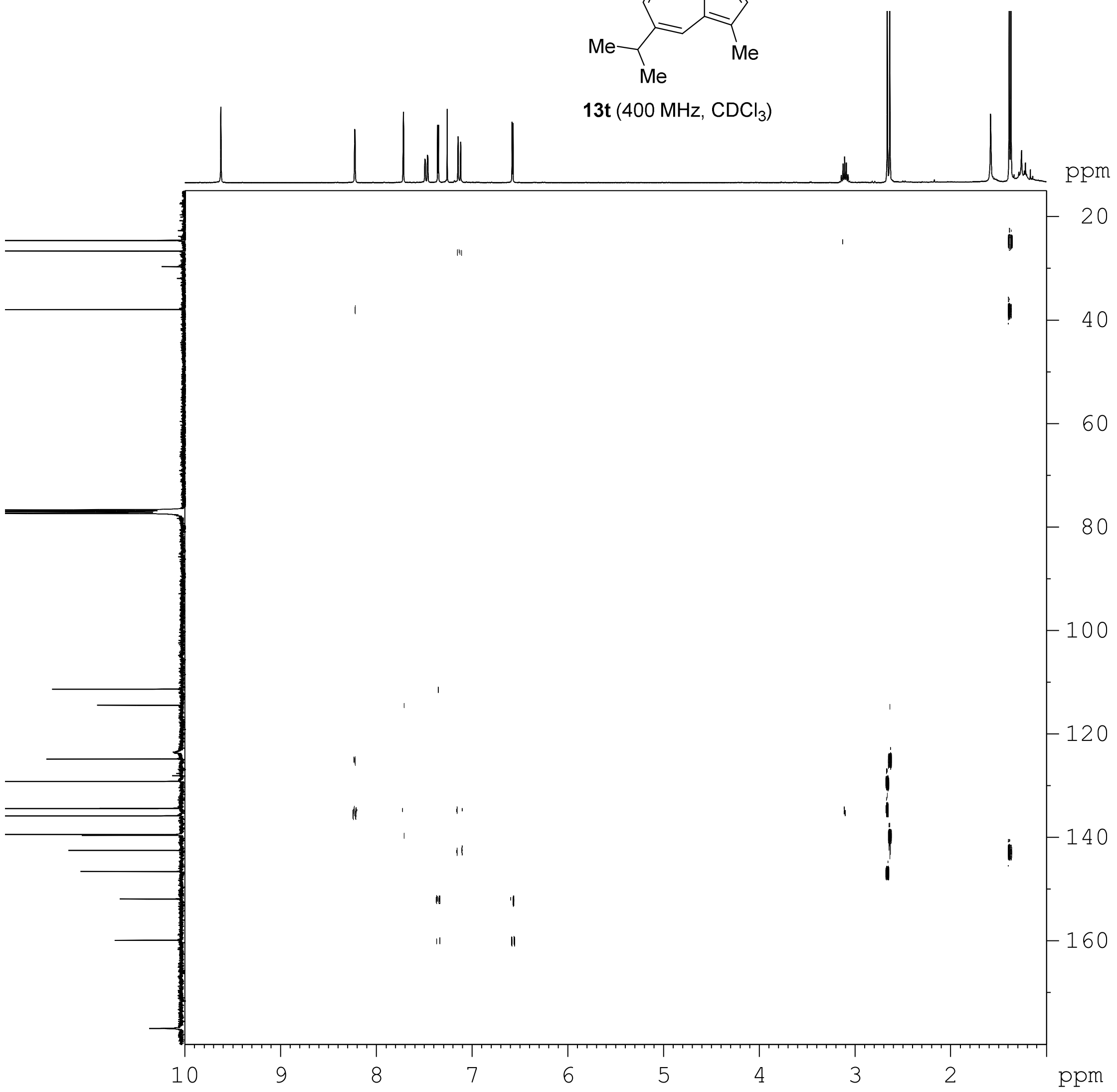

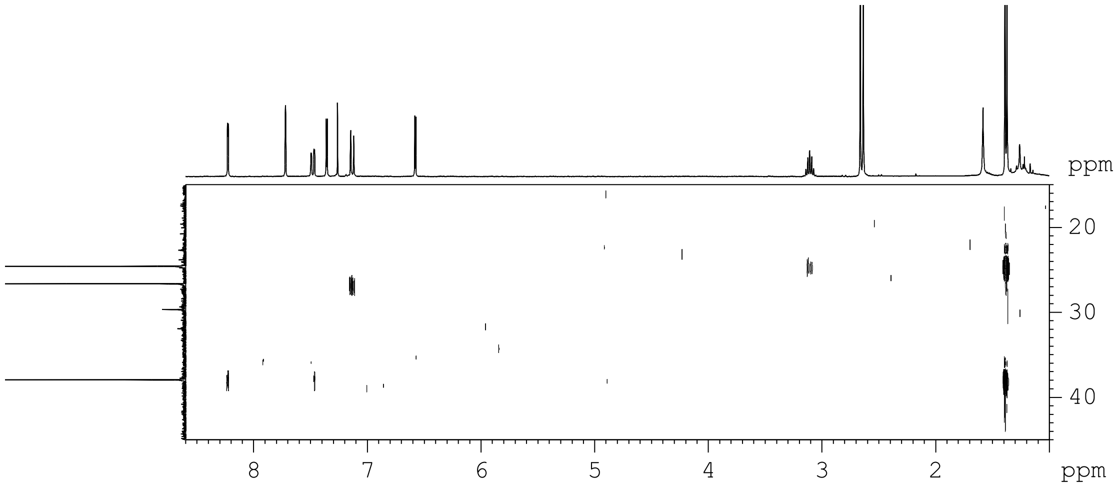

HMBC

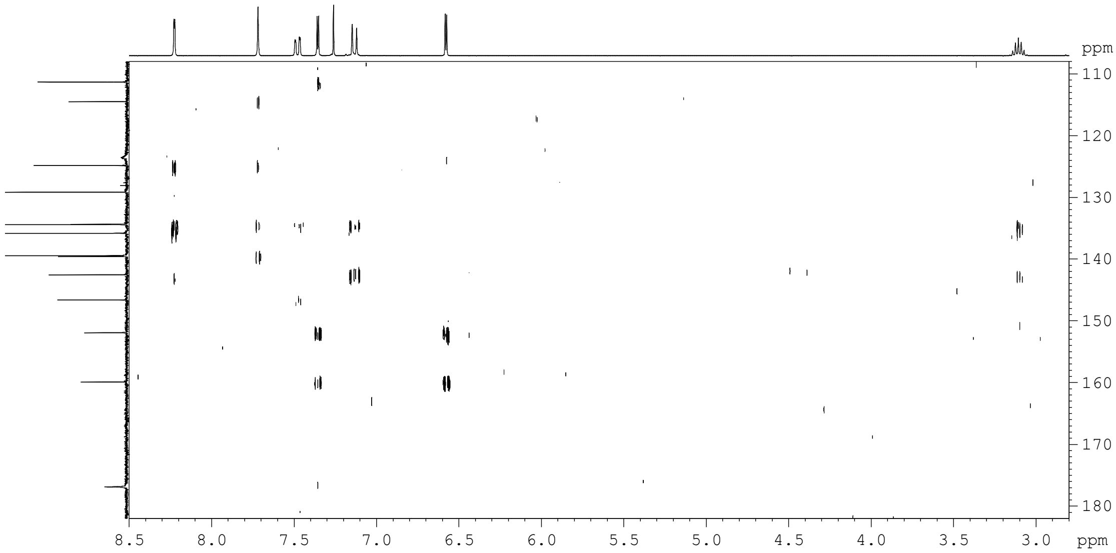

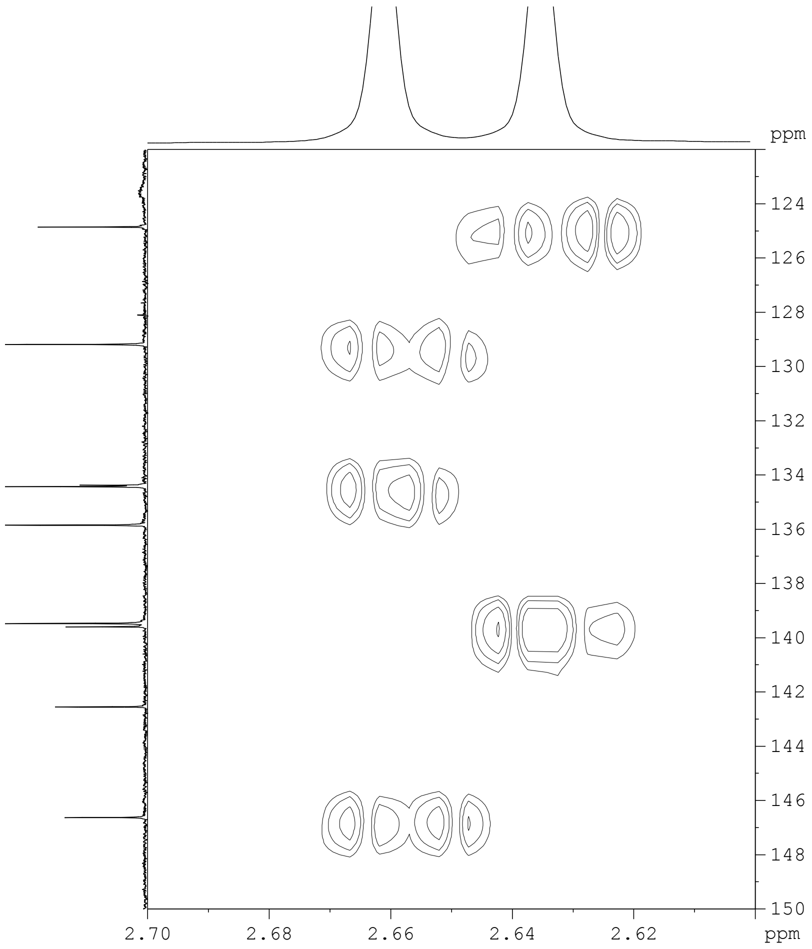

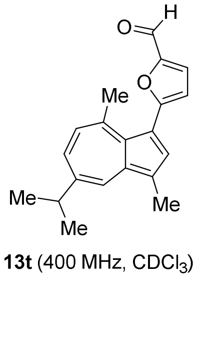

13t (400 MHz, $CDCl_3$)

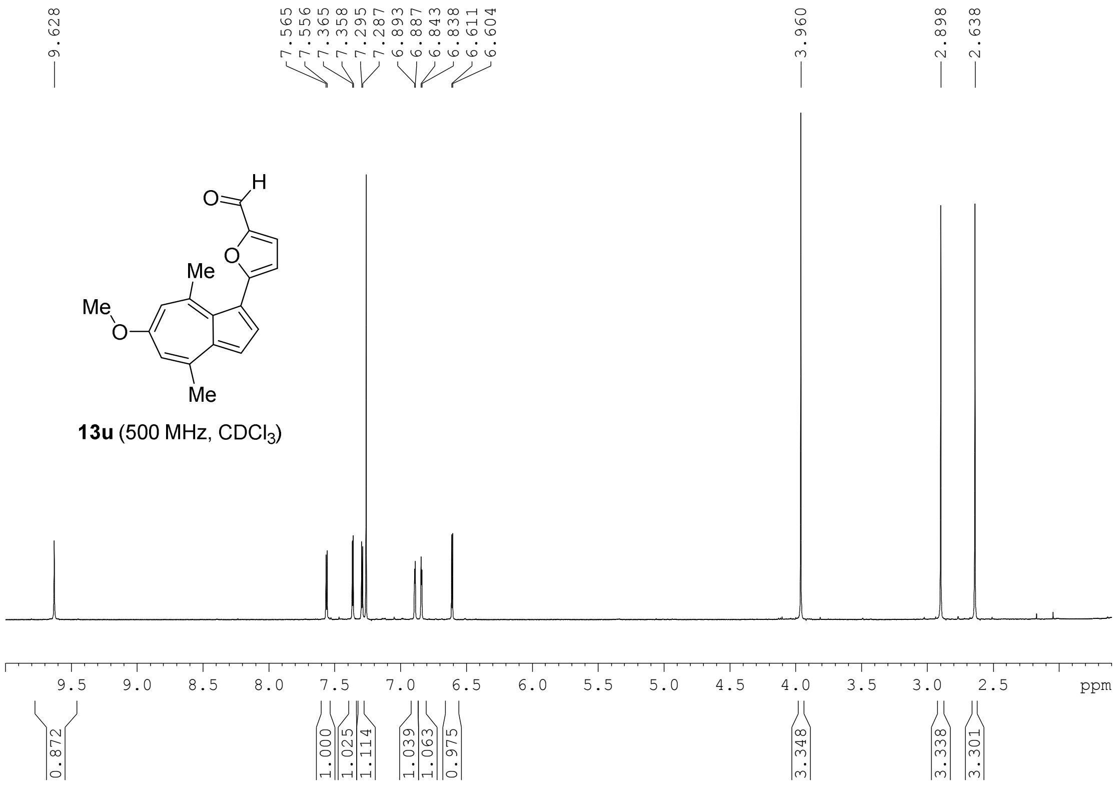
9.628
7.565
7.556
7.365
7.358
7.295
7.287
6.893
6.887
6.843
6.838
6.611
6.604
3.960
2.898
2.638
O
H
O
Me
Me
O
Me
13u (500 MHz, CDCl3)
ppm
0.872
1.000
1.025
1.114
1.039
1.063
0.975
3.348
3.338
3.301

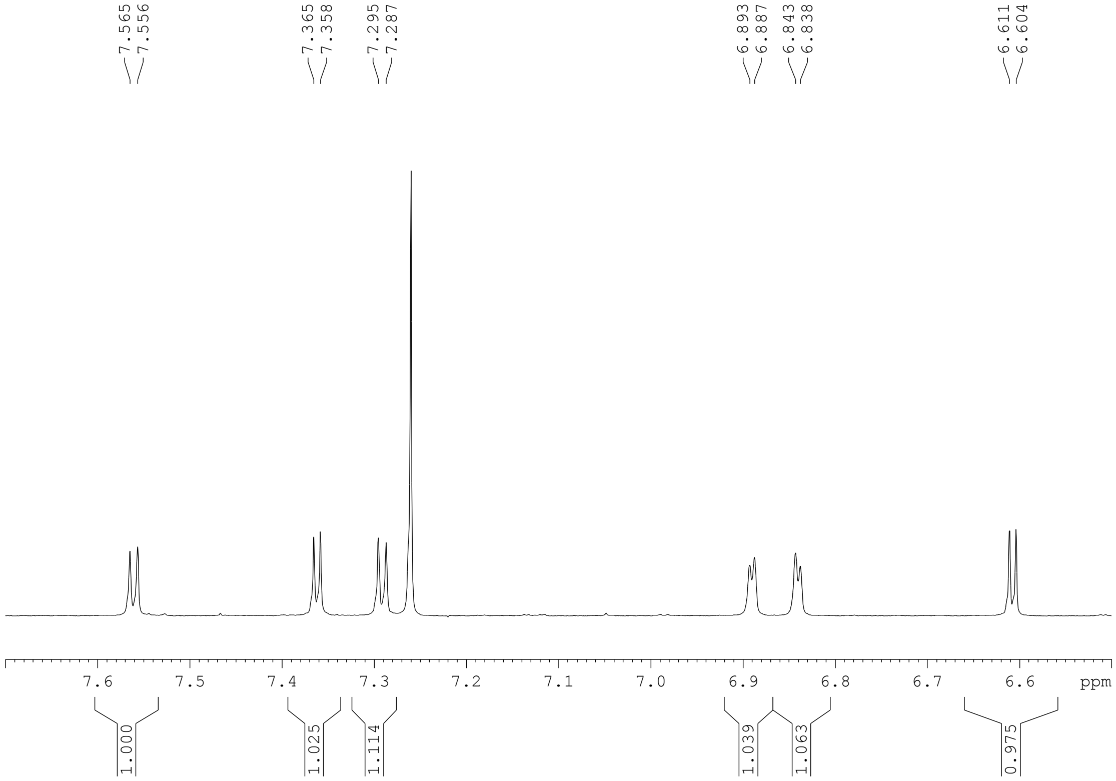
7.565
7.556
7.365
7.358
7.295
7.287
6.893
6.887
6.843
6.838
6.611
6.604
ppm
1.000
1.025
1.114
1.039
1.063
0.975

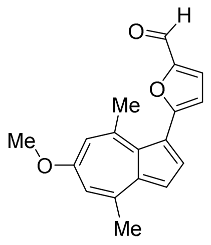

13u (126 MHz, $CDCl_3$)

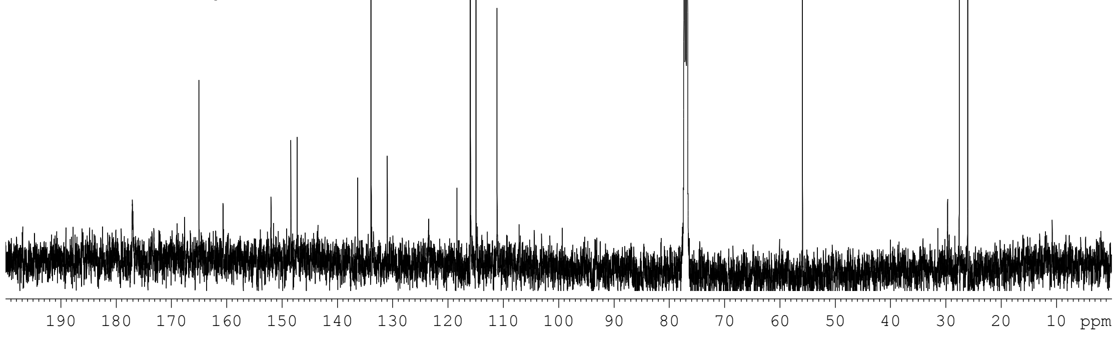

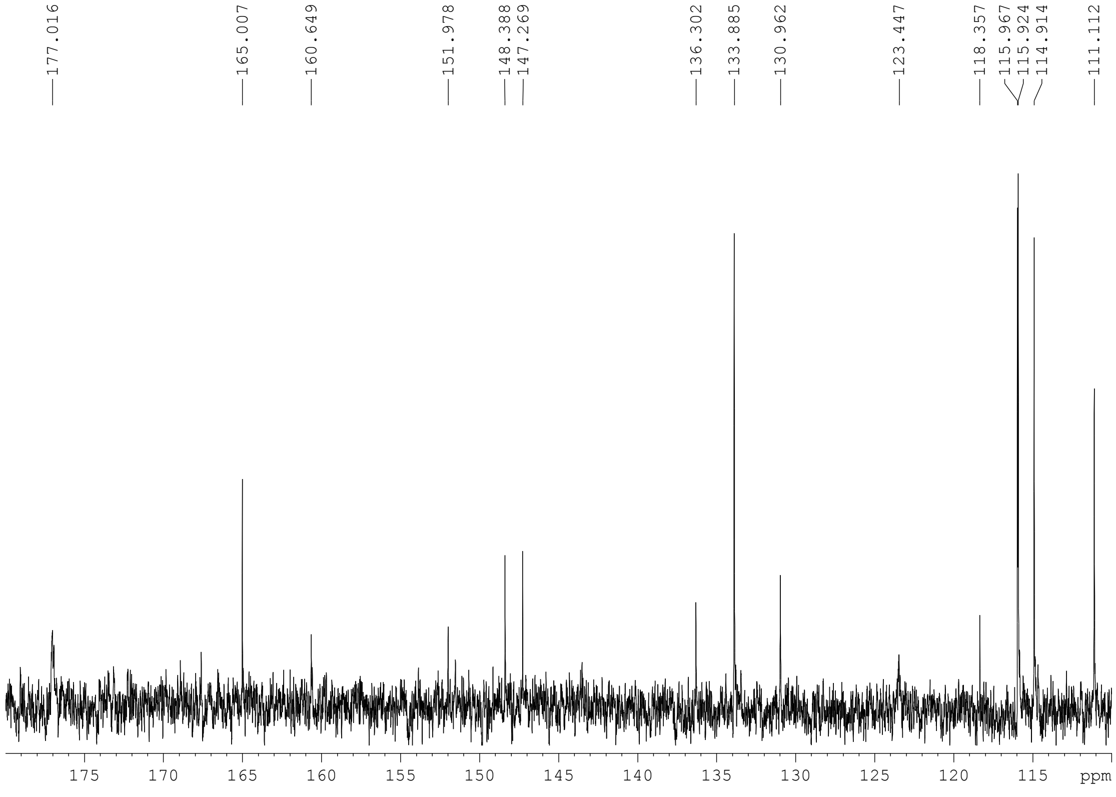

COSY

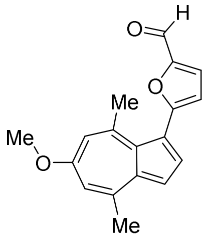

13u (500 MHz, $CDCl_3$)

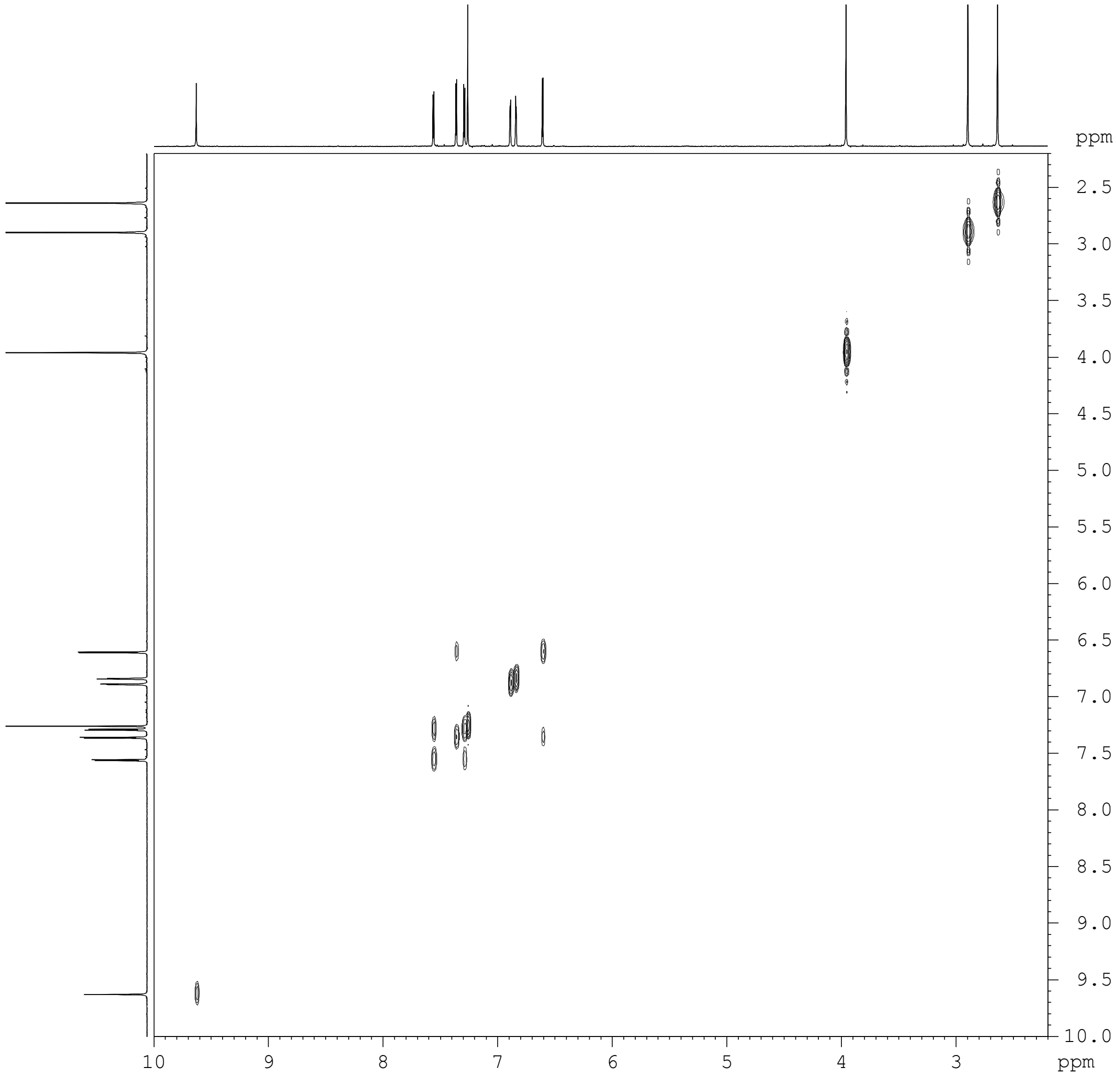

NOESY

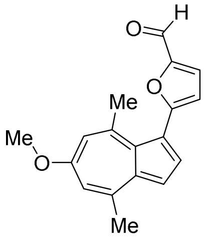

13u (500 MHz, $CDCl_3$)

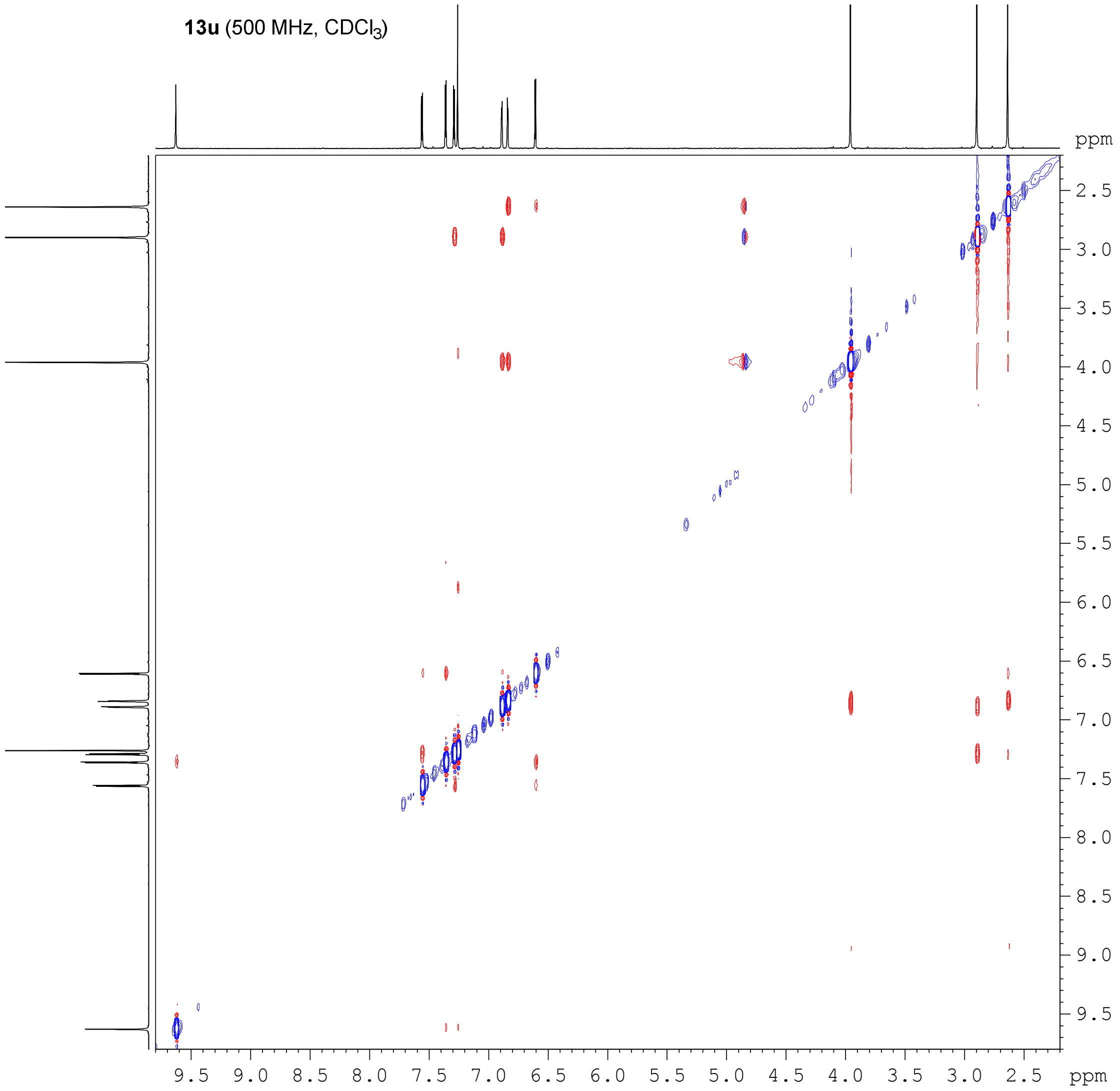

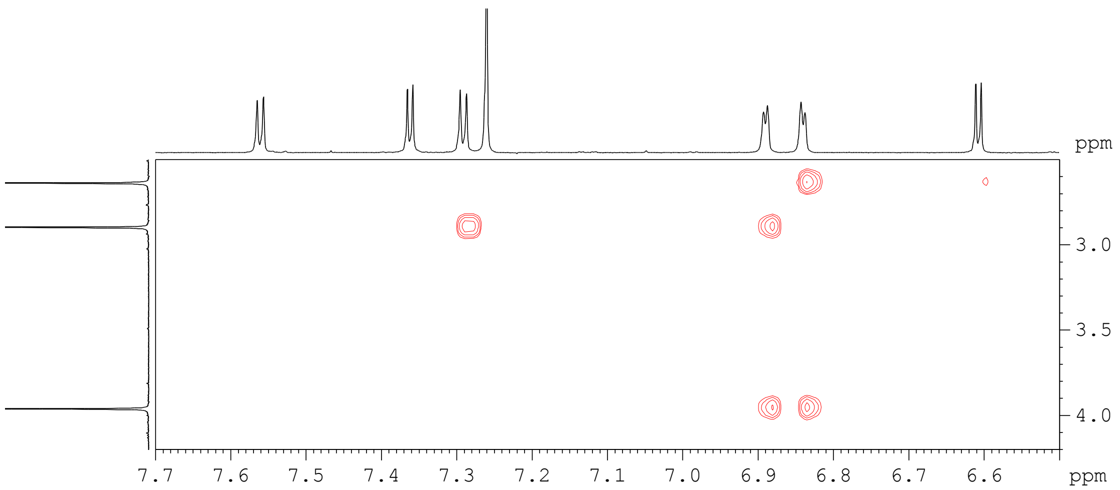

HSQC

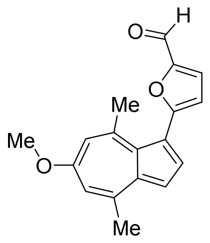

13u (500 MHz, $CDCl_3$)

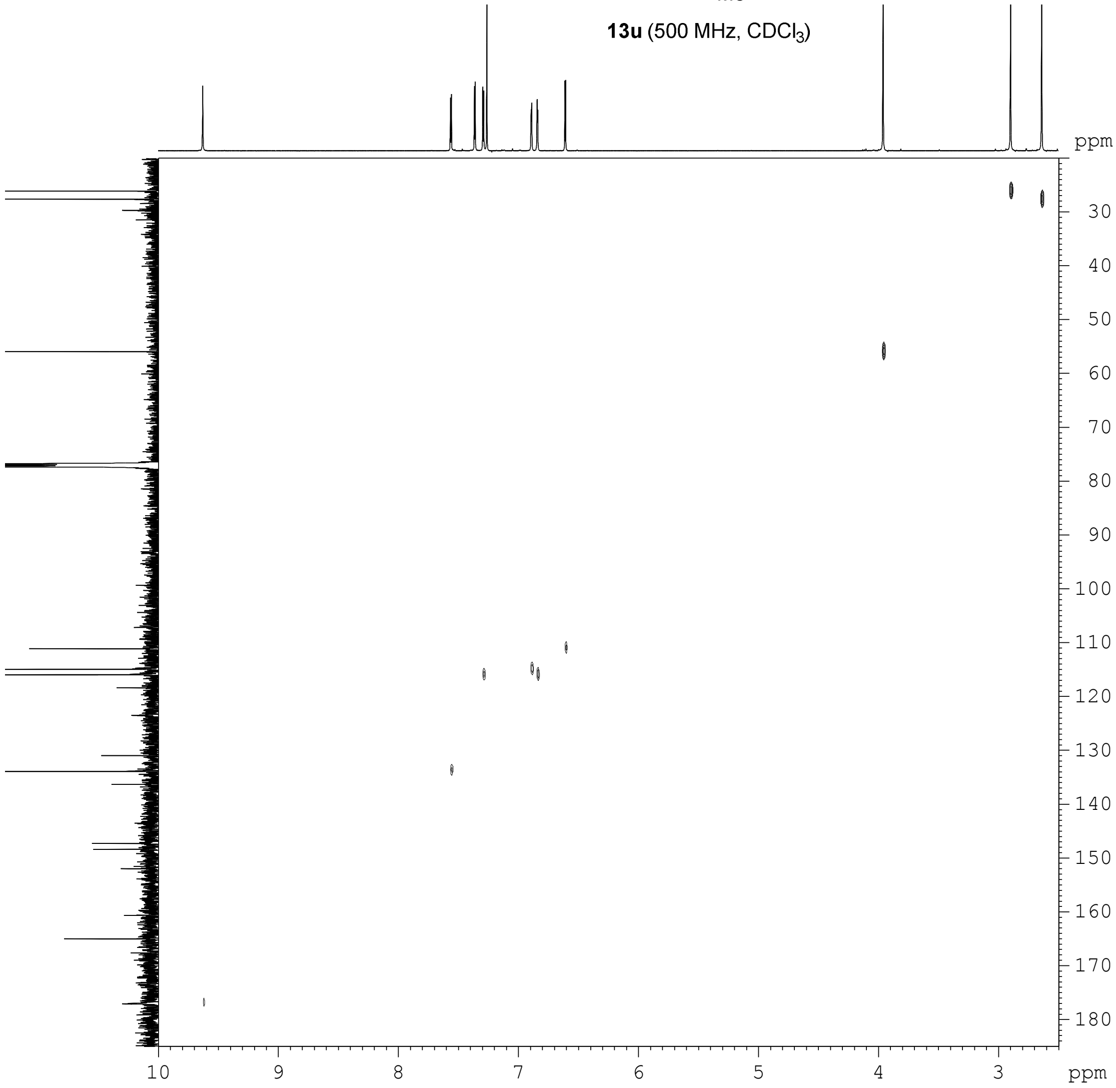

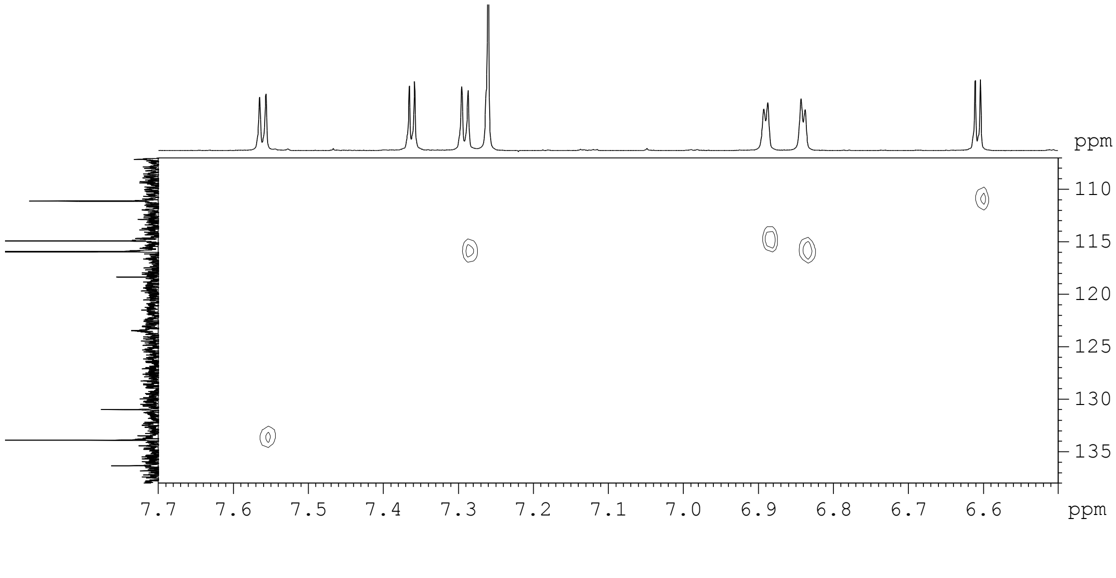

HMBC

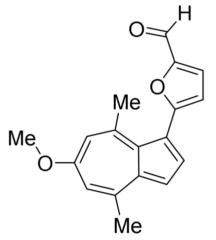

13u (500 MHz, $CDCl_3$)

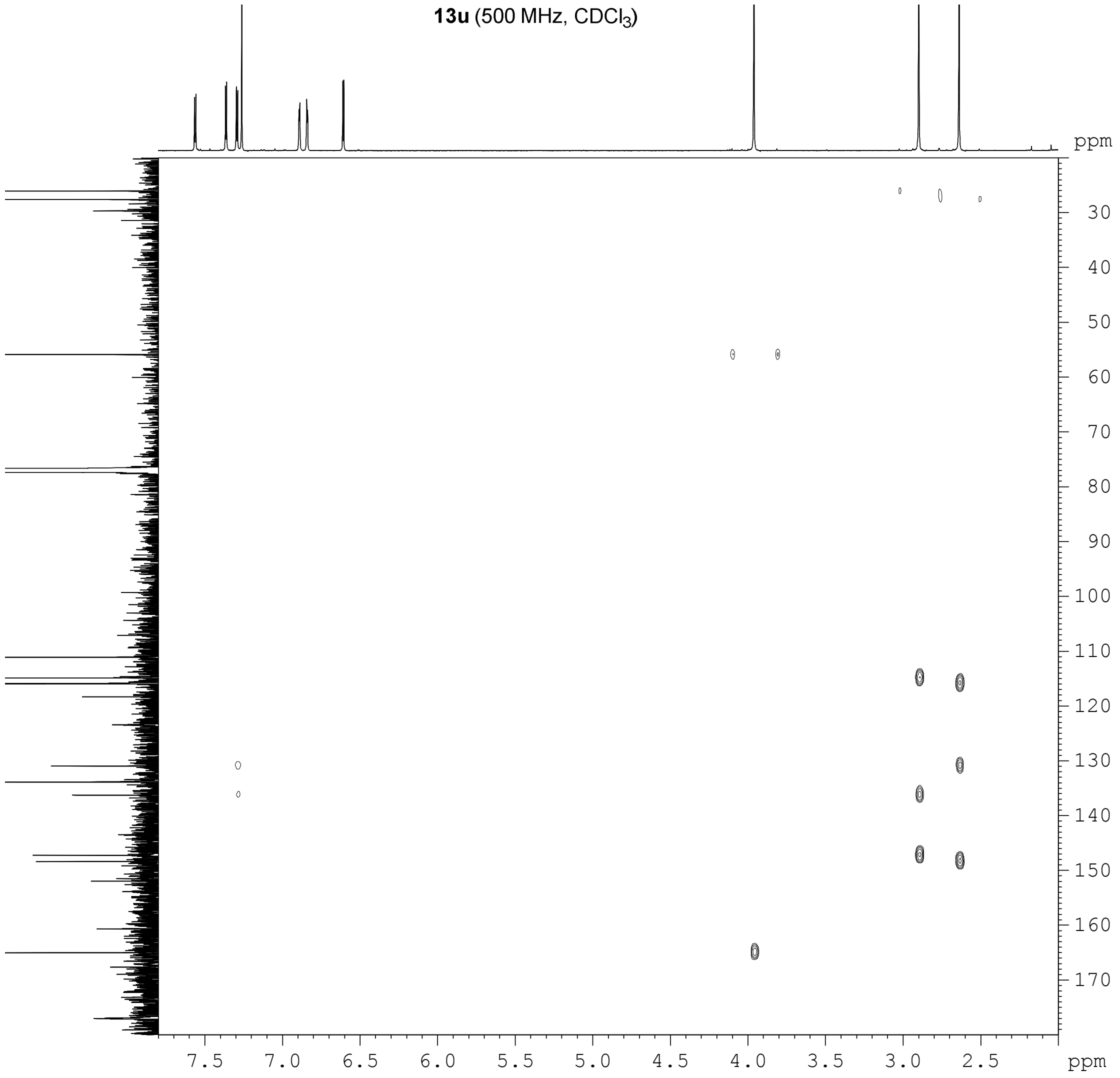

HMBC

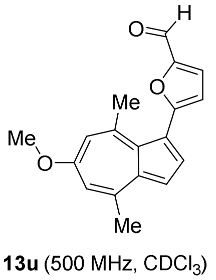
O
H
O
Me
Me
O
Me
13u (500 MHz, CDCl3)

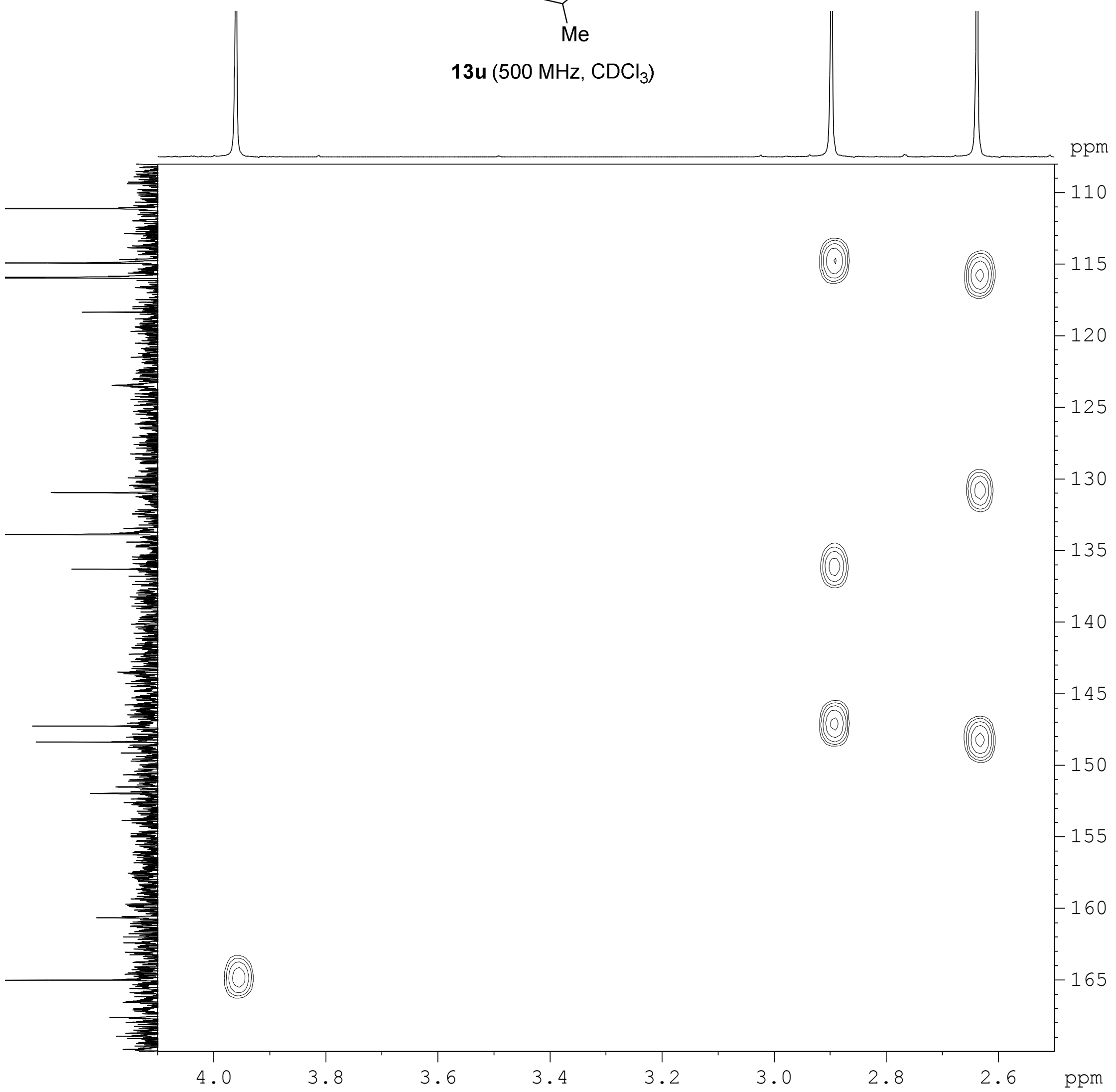
ppm
110
115
120
125
130
135
140
145
150
155
160
165
4.0
3.8
3.6
3.4
3.2
3.0
2.8
2.6
ppm

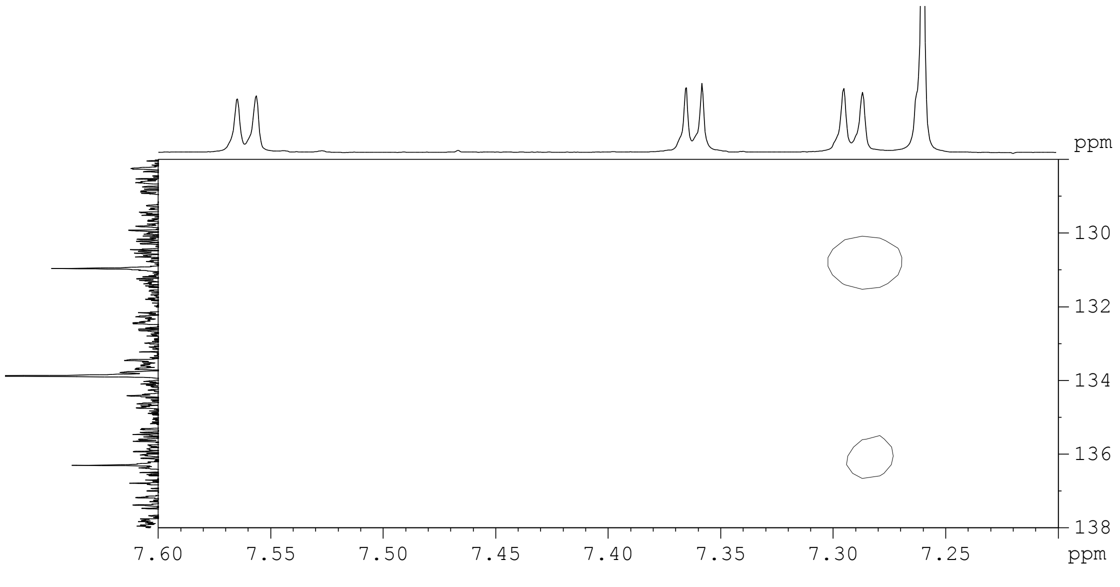
ppm
130
132
134
136
138
7.60
7.55
7.50
7.45
7.40
7.35
7.30
7.25
ppm

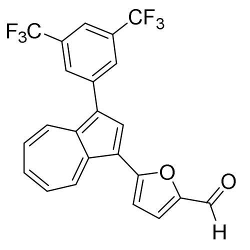

13v (500 MHz, $CDCl_3$)

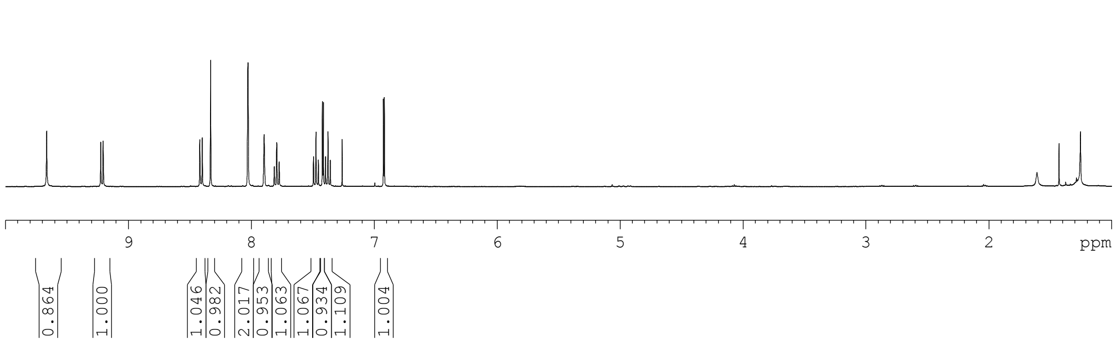

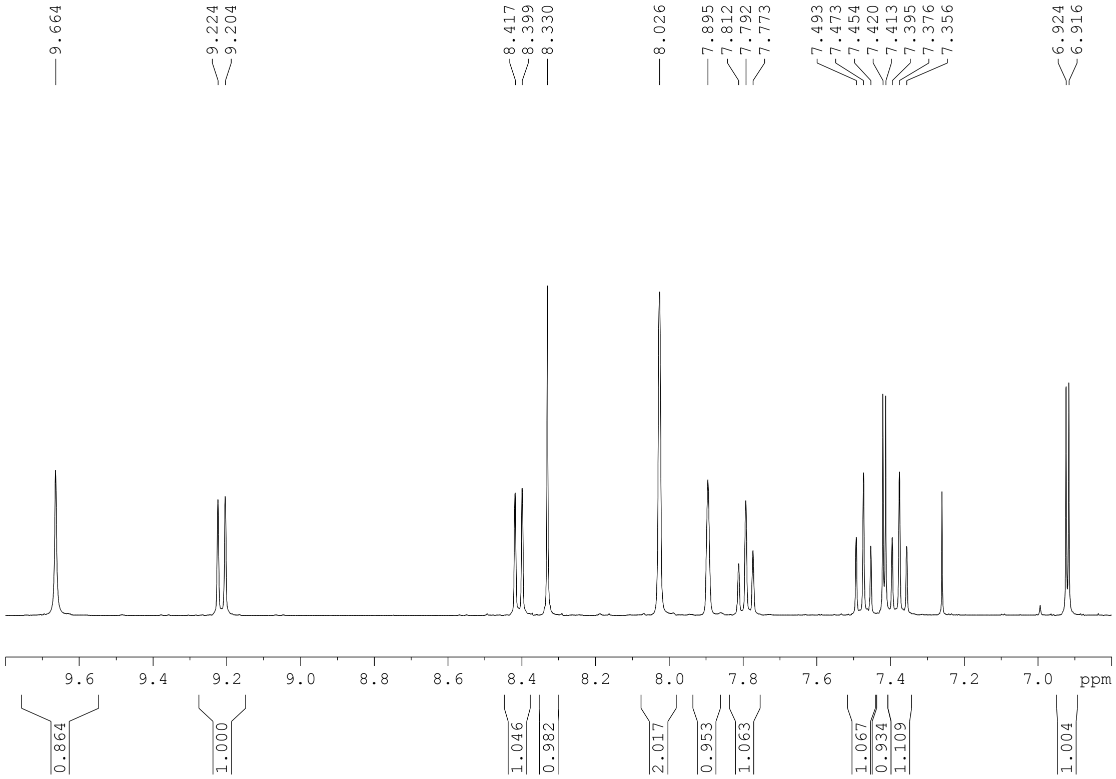

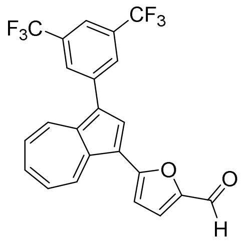

13v (126 MHz, $CDCl_3$)

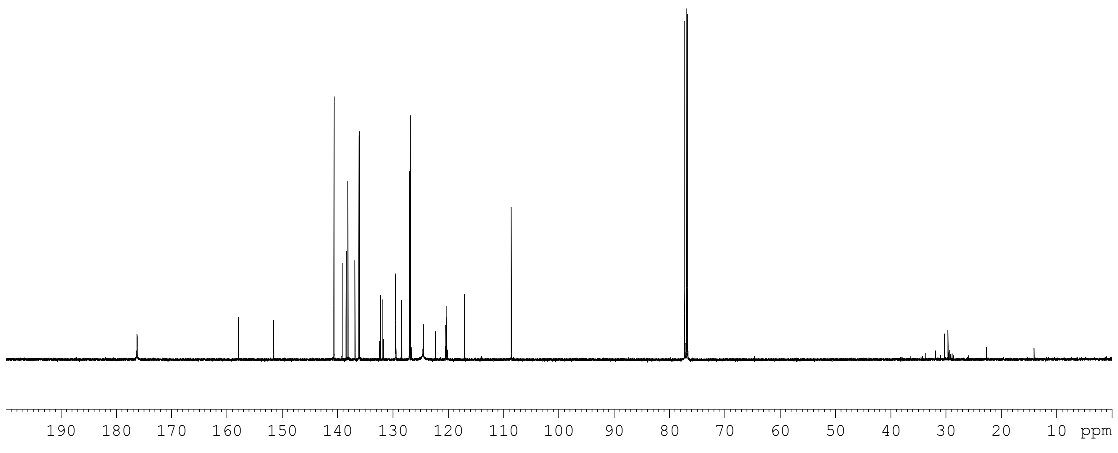

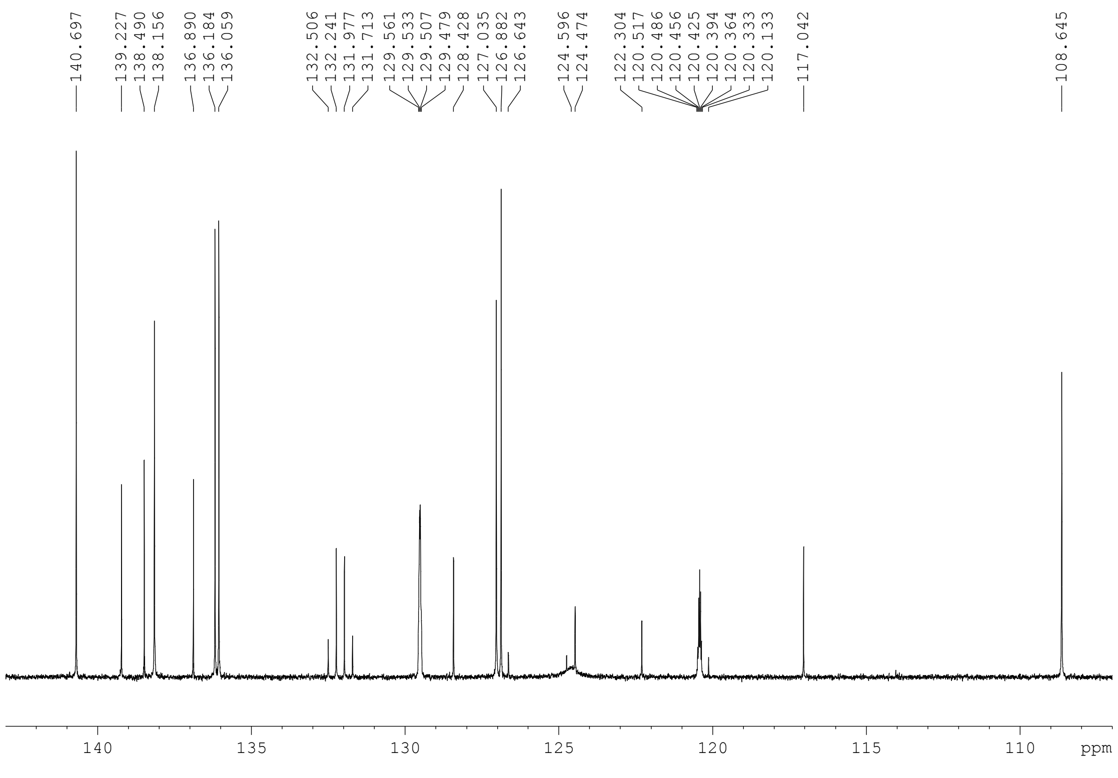

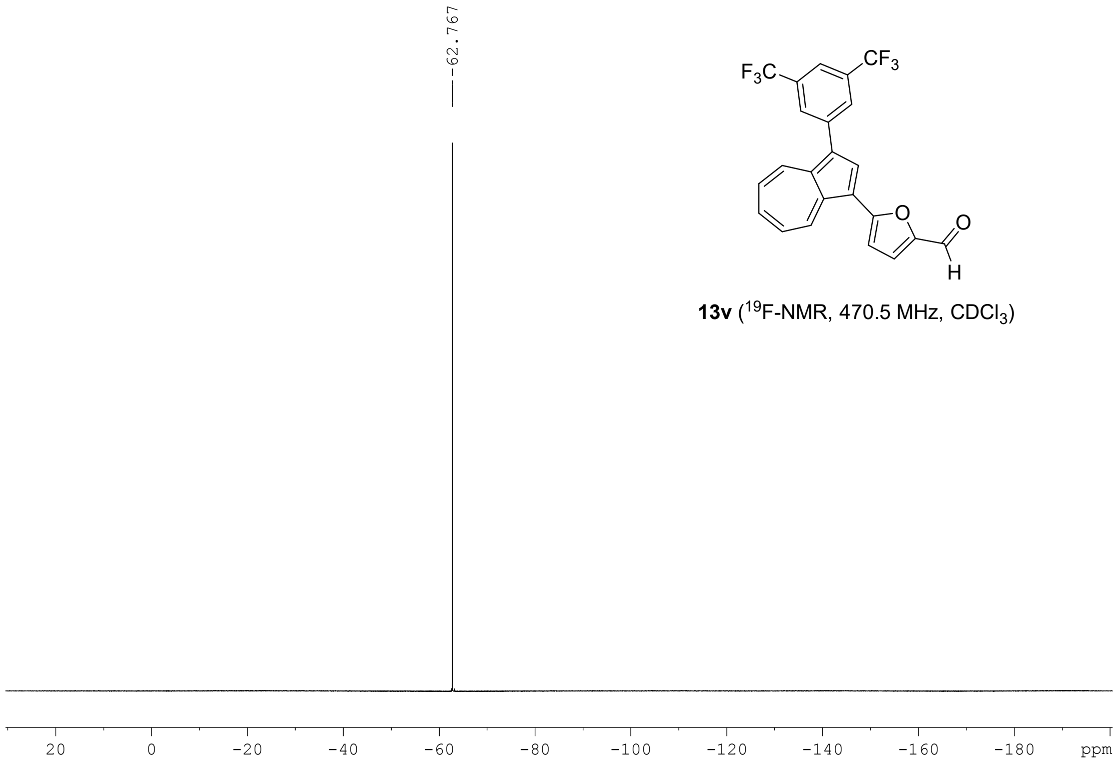

13v (^{19}F-NMR, 470.5 MHz, $CDCl_3$)

COSY

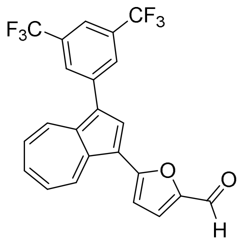

13v (500 MHz, $CDCl_3$)

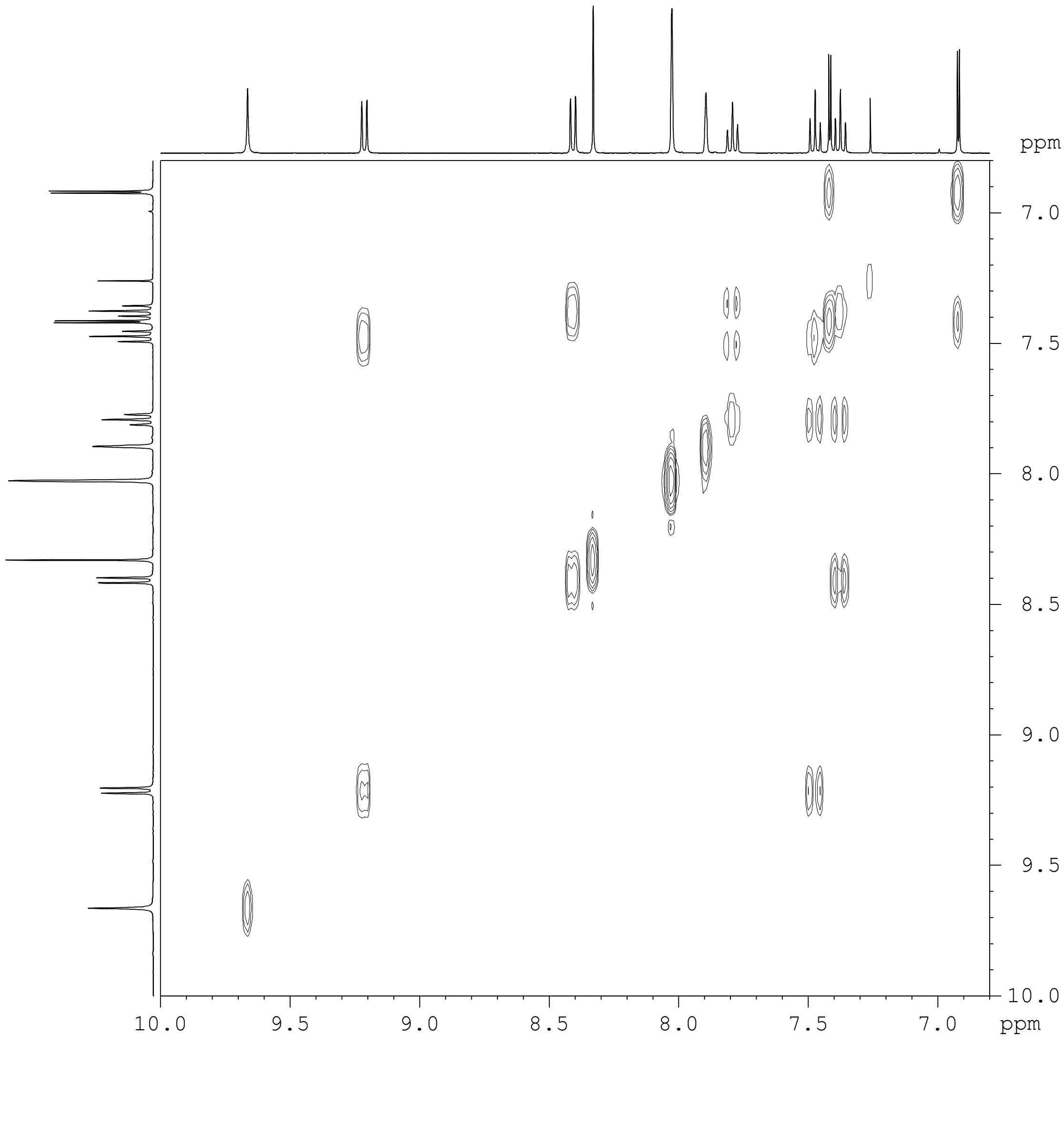

NOESY

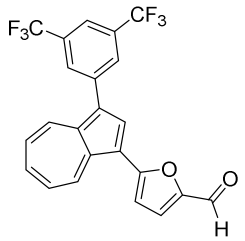

13v (500 MHz, $CDCl_3$)

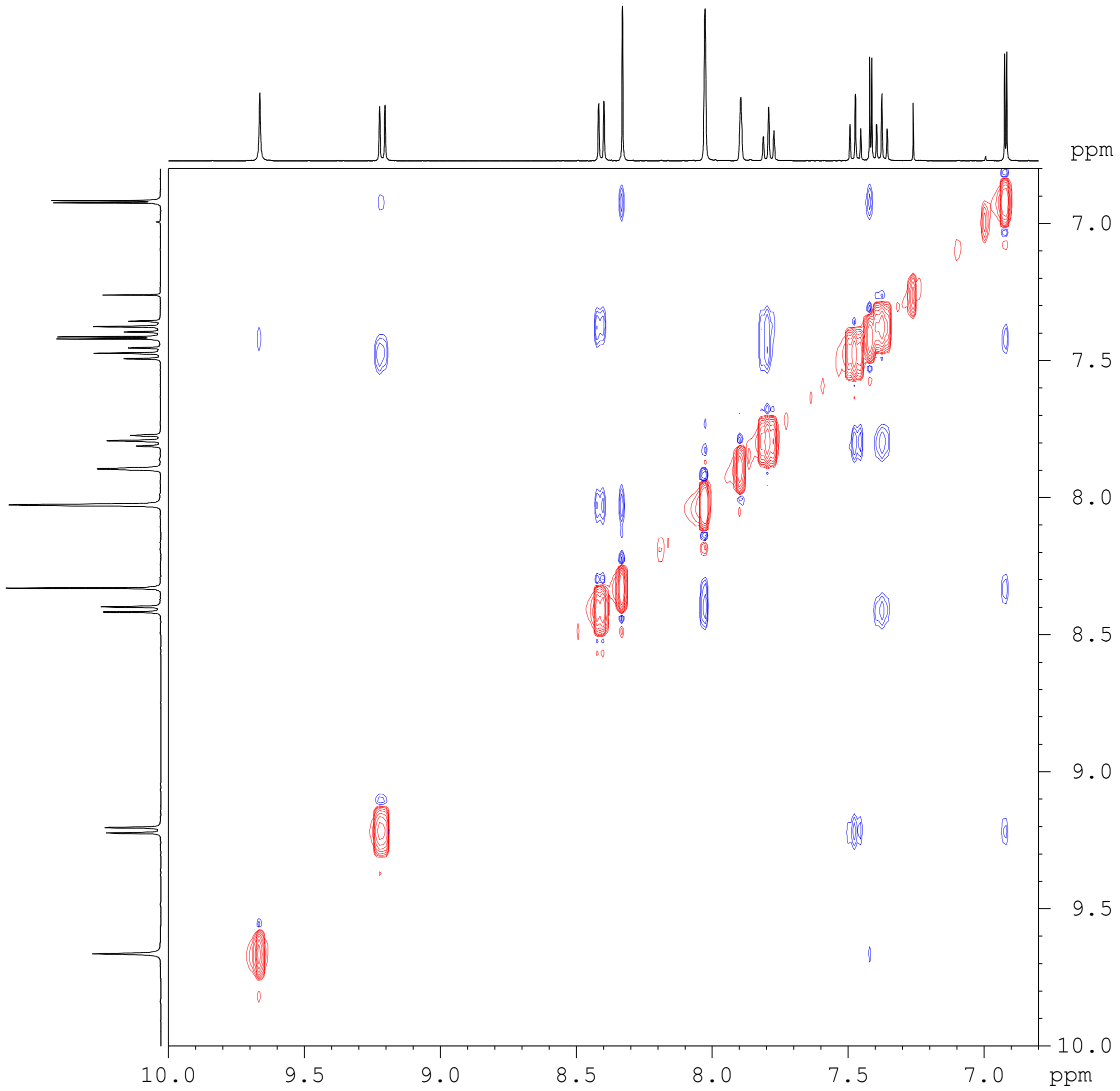

HSQC

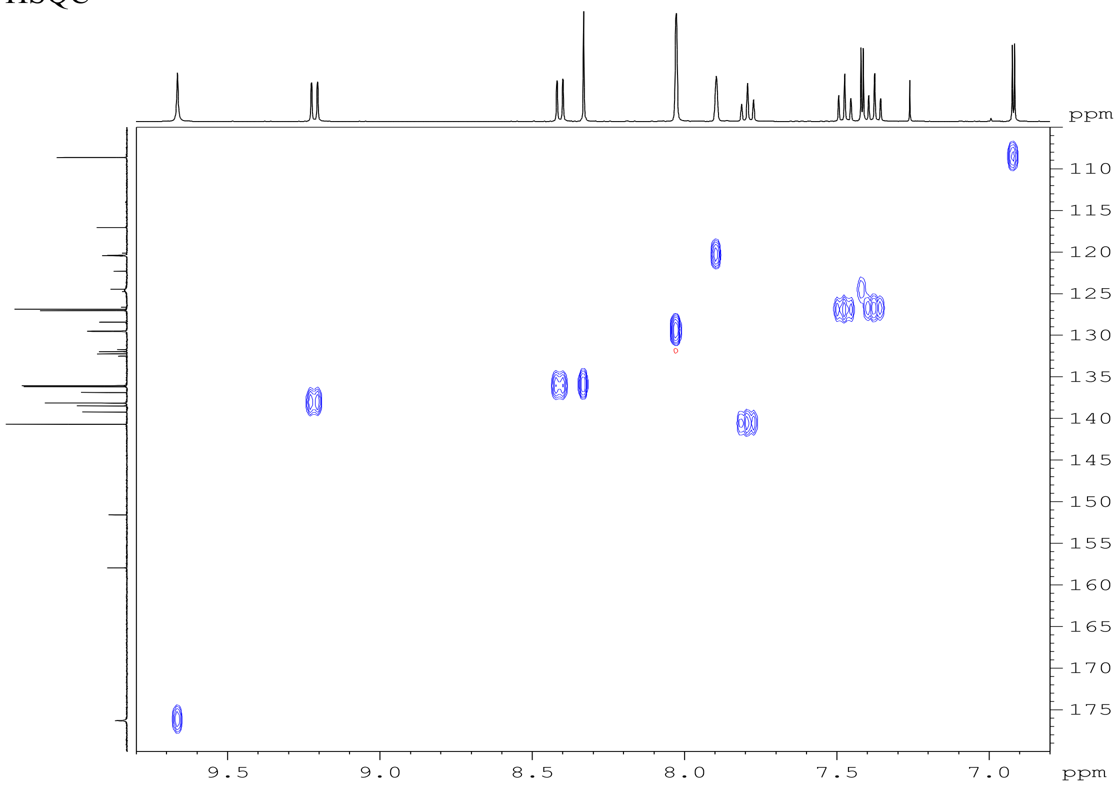

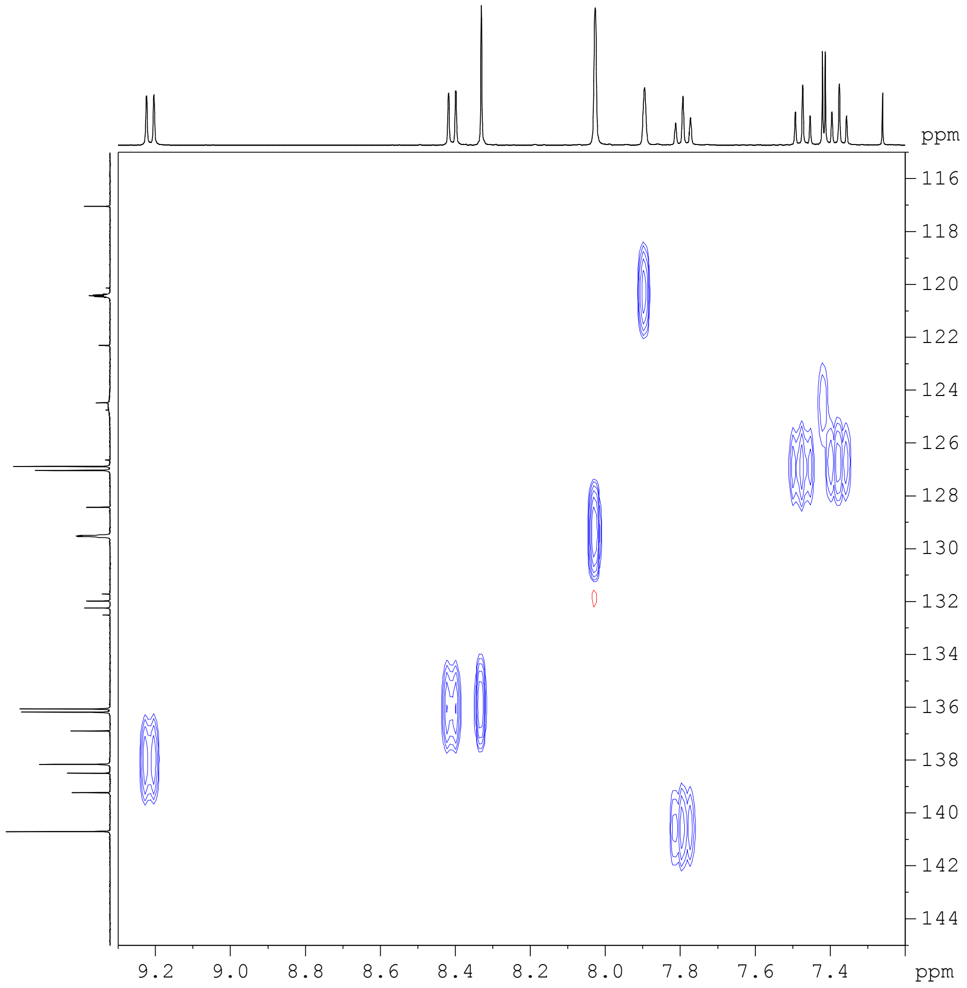

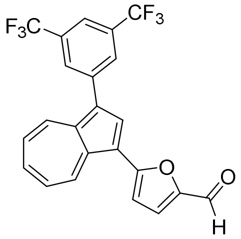

13v (500 MHz, $CDCl_3$)

HMBC

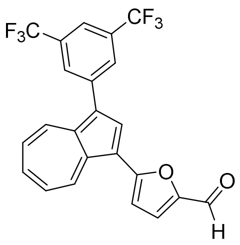

13v (500 MHz, $CDCl_3$)

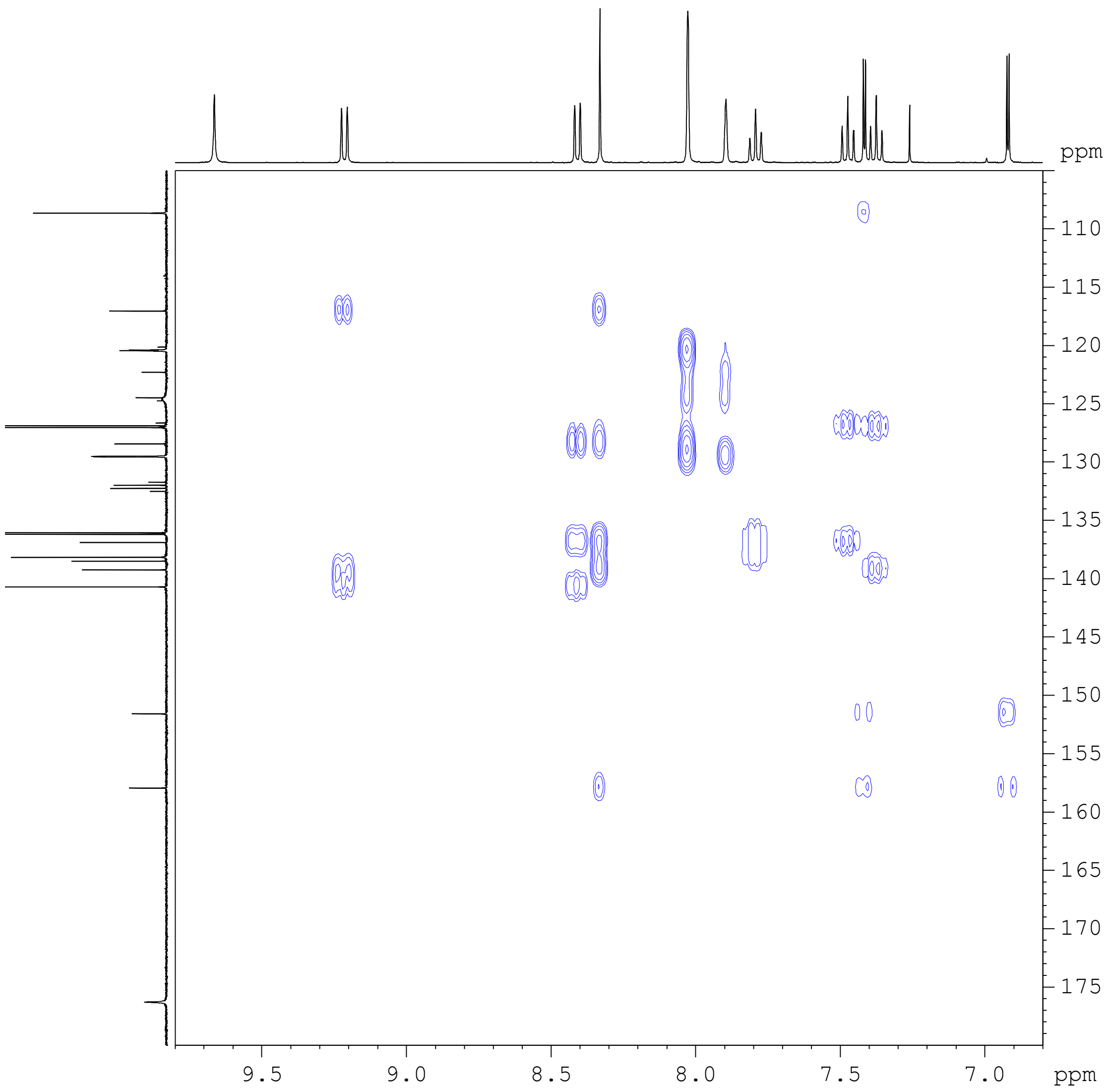

X-ray crystallography

Data for s14sel1(**11a**) and s15sel5(**11b**) were recorded at 150 K on an Agilent SuperNova E-Dual Diffractometer equipped with an Oxford Cryostream using Cu-Kα radiation (λ= 1.54184 Å). Data for e15sel3(**11c**), e15sel3(**11d**), e15sel4(**11e**) and e15sel1(**11f**) were collected on an Agilent XCalibur EosS2 diffractometer using MoKα radiation (λ= 0.71073 Å). For all structures a symmetry-related (multi-scan) absorption correction had been applied. Crystal parameters and details on data collection, solution and refinement for the complexes are provided in Tables S2-43. Structure solution, followed by full-matrix least squares refinement was performed using the WINGX-v2014.1 suite of programs throughout.[5]

Table S2. Crystal data and structure refinement for **11a**

Identification code	s14sel1	
CCDC Number	#1435808	
Empirical formula	$C_{14} H_{15} F_6 P S$	
Formula weight	360.29	
Temperature	150(2) K	
Wavelength	1.54184 Å	
Crystal system	Monoclinic	
Space group	$P2_1/n$	
Unit cell dimensions	a = 6.9015(2) Å	α= 90°.
	b = 8.1374(3) Å	β= 91.477(3)°.
	c = 26.9719(9) Å	γ = 90°.
Volume	1514.25(9) $Å^3$	
Z	4	
Density (calculated)	1.580 Mg/m^3	
Absorption coefficient	3.462 mm^{-1}	
F(000)	736	
Crystal size	0.190 x 0.074 x 0.033 mm^3	
Theta range for data collection	5.679 to 72.148°.	
Index ranges	-5<=h<=8, -10<=k<=10, -33<=l<=33	
Reflections collected	14711	
Independent reflections	2975 [R(int) = 0.0680]	
Completeness to theta = 67.684°	99.9 %	
Absorption correction	Semi-empirical from equivalents	
Max. and min. transmission	1.00000 and 0.70011	
Refinement method	Full-matrix least-squares on F^2	
Data / restraints / parameters	2975 / 0 / 254	
Goodness-of-fit on F^2	1.025	
Final R indices [I>2sigma(I)]	R1 = 0.0626, wR2 = 0.1732	
R indices (all data)	R1 = 0.0755, wR2 = 0.1869	
Extinction coefficient	n/a	
Largest diff. peak and hole	0.453 and -0.343 e.$Å^{-3}$	

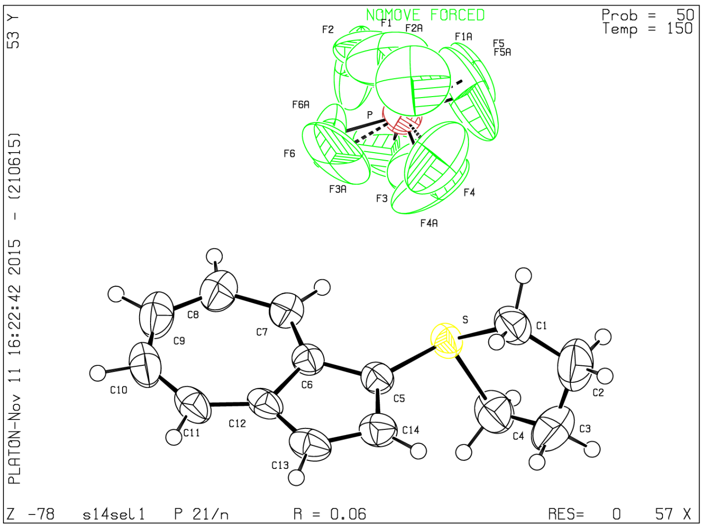

Table S3. Atomic coordinates (x 10^4) and equivalent isotropic displacement parameters (Å^{2}x 10^3) for **11a**. U(eq) is defined as one third of the trace of the orthogonalized U^{ij} tensor.

	x	y	z	U(eq)
S	2523(1)	4804(1)	3411(1)	46(1)
C(1)	4459(5)	3704(5)	3104(1)	60(1)
C(2)	3475(8)	2683(7)	2710(2)	89(1)
C(3)	1615(9)	2114(7)	2891(2)	95(2)
C(4)	605(5)	3535(6)	3132(1)	70(1)
C(5)	2694(4)	4227(4)	4028(1)	42(1)
C(6)	2464(3)	5369(3)	4415(1)	39(1)
C(7)	2119(4)	7038(4)	4361(1)	49(1)
C(8)	1877(5)	8199(5)	4733(2)	63(1)
C(9)	1929(5)	7985(6)	5244(2)	70(1)
C(10)	2202(5)	6585(6)	5519(1)	66(1)
C(11)	2509(4)	4985(5)	5359(1)	56(1)
C(12)	2640(3)	4408(4)	4875(1)	44(1)
C(13)	2989(4)	2777(4)	4741(1)	53(1)
C(14)	3021(5)	2647(4)	4231(1)	52(1)
P	7211(2)	8776(1)	3424(1)	63(1)
F(1)	9430(30)	9230(30)	3558(11)	175(9)
F(2)	6810(30)	10603(17)	3542(5)	159(8)
F(3)	5220(20)	8270(30)	3275(9)	162(8)
F(4)	8050(30)	6918(18)	3420(7)	167(7)
F(5)	7490(14)	9190(20)	2839(4)	119(4)
F(6)	6930(30)	8380(20)	3976(5)	178(8)
F(1A)	9309(19)	8510(30)	3447(9)	160(7)
F(2A)	7370(20)	10651(14)	3424(7)	161(7)
F(3A)	4980(20)	9120(30)	3388(10)	168(7)
F(4A)	6710(30)	6952(13)	3418(7)	180(7)
F(6A)	7090(40)	8823(19)	3986(4)	199(9)
F(5A)	7400(30)	8750(40)	2927(6)	251(12)

Table S4. Bond lengths [Å] for **11a**.

S-C(5)	1.730(3)
S-C(1)	1.824(3)
S-C(4)	1.826(4)
C(1)-C(2)	1.498(6)
C(1)-H(1A)	0.9900
C(1)-H(1B)	0.9900
C(2)-C(3)	1.460(7)
C(2)-H(2A)	0.9900
C(2)-H(2B)	0.9900
C(3)-C(4)	1.506(7)
C(3)-H(3A)	0.9900
C(3)-H(3B)	0.9900
C(4)-H(4A)	0.9900
C(4)-H(4B)	0.9900
C(5)-C(6)	1.409(4)
C(5)-C(14)	1.414(4)
C(6)-C(7)	1.386(4)
C(6)-C(12)	1.469(4)
C(7)-C(8)	1.392(5)
C(7)-H(7)	0.9500
C(8)-C(9)	1.387(6)
C(8)-H(8)	0.9500
C(9)-C(10)	1.370(7)
C(9)-H(9)	0.9500
C(10)-C(11)	1.389(6)
C(10)-H(10)	0.9500
C(11)-C(12)	1.394(4)
C(11)-H(11)	0.9500
C(12)-C(13)	1.397(5)
C(13)-C(14)	1.382(5)
C(13)-H(13)	0.9500
C(14)-H(14)	0.9500
P-F(5A)	1.351(17)
P-F(1A)	1.464(12)
P-F(3)	1.483(14)
P-F(6A)	1.520(10)
P-F(4A)	1.524(9)
P-F(2A)	1.530(11)
P-F(6)	1.540(12)
P-F(2)	1.547(11)
P-F(3A)	1.563(15)
P-F(1)	1.610(18)
P-F(4)	1.618(12)
P-F(5)	1.629(11)

Table S5. Bond angles [°] for **11a**.

C(5)-S-C(1)	105.70(16)	C(11)-C(10)-H(10)	115.4
C(5)-S-C(4)	105.86(16)	C(10)-C(11)-C(12)	128.4(3)
C(1)-S-C(4)	93.85(18)	C(10)-C(11)-H(11)	115.8
C(2)-C(1)-S	105.7(3)	C(12)-C(11)-H(11)	115.8
C(2)-C(1)-H(1A)	110.6	C(11)-C(12)-C(13)	125.3(3)
S-C(1)-H(1A)	110.6	C(11)-C(12)-C(6)	127.3(3)
C(2)-C(1)-H(1B)	110.6	C(13)-C(12)-C(6)	107.4(3)
S-C(1)-H(1B)	110.6	C(14)-C(13)-C(12)	109.6(3)
H(1A)-C(1)-H(1B)	108.7	C(14)-C(13)-H(13)	125.2
C(3)-C(2)-C(1)	109.0(4)	C(12)-C(13)-H(13)	125.2
C(3)-C(2)-H(2A)	109.9	C(13)-C(14)-C(5)	108.1(3)
C(1)-C(2)-H(2A)	109.9	C(13)-C(14)-H(14)	126.0
C(3)-C(2)-H(2B)	109.9	C(5)-C(14)-H(14)	126.0
C(1)-C(2)-H(2B)	109.9	F(5A)-P-F(1A)	85.4(13)
H(2A)-C(2)-H(2B)	108.3	F(5A)-P-F(6A)	177.5(14)
C(2)-C(3)-C(4)	108.7(4)	F(1A)-P-F(6A)	92.2(14)
C(2)-C(3)-H(3A)	109.9	F(5A)-P-F(4A)	90.2(15)
C(4)-C(3)-H(3A)	109.9	F(1A)-P-F(4A)	94.7(9)
C(2)-C(3)-H(3B)	109.9	F(6A)-P-F(4A)	91.0(8)
C(4)-C(3)-H(3B)	109.9	F(5A)-P-F(2A)	90.2(14)
H(3A)-C(3)-H(3B)	108.3	F(1A)-P-F(2A)	94.4(10)
C(3)-C(4)-S	105.9(3)	F(6A)-P-F(2A)	88.9(8)
C(3)-C(4)-H(4A)	110.6	F(4A)-P-F(2A)	170.9(10)
S-C(4)-H(4A)	110.6	F(3)-P-F(6)	93.6(13)
C(3)-C(4)-H(4B)	110.6	F(3)-P-F(2)	98.9(10)
S-C(4)-H(4B)	110.6	F(6)-P-F(2)	88.5(8)
H(4A)-C(4)-H(4B)	108.7	F(5A)-P-F(3A)	93.4(13)
C(6)-C(5)-C(14)	109.4(3)	F(1A)-P-F(3A)	177.8(12)
C(6)-C(5)-S	121.8(2)	F(6A)-P-F(3A)	88.9(14)
C(14)-C(5)-S	128.7(2)	F(4A)-P-F(3A)	87.3(8)
C(7)-C(6)-C(5)	126.2(3)	F(2A)-P-F(3A)	83.7(10)
C(7)-C(6)-C(12)	128.3(3)	F(3)-P-F(1)	175.8(12)
C(5)-C(6)-C(12)	105.4(3)	F(6)-P-F(1)	88.5(13)
C(6)-C(7)-C(8)	127.9(3)	F(2)-P-F(1)	84.7(11)
C(6)-C(7)-H(7)	116.1	F(3)-P-F(4)	93.8(9)
C(8)-C(7)-H(7)	116.1	F(6)-P-F(4)	82.3(10)
C(9)-C(8)-C(7)	129.0(4)	F(2)-P-F(4)	164.7(9)
C(9)-C(8)-H(8)	115.5	F(1)-P-F(4)	82.9(9)
C(7)-C(8)-H(8)	115.5	F(3)-P-F(5)	85.9(10)
C(10)-C(9)-C(8)	130.0(4)	F(6)-P-F(5)	179.5(10)
C(10)-C(9)-H(9)	115.0	F(2)-P-F(5)	91.6(9)
C(8)-C(9)-H(9)	115.0	F(1)-P-F(5)	92.0(11)
C(9)-C(10)-C(11)	129.1(3)	F(4)-P-F(5)	97.8(10)
C(9)-C(10)-H(10)	115.4		

Symmetry transformations used to generate equivalent atoms:

Table S6. Anisotropic displacement parameters ($Å^2 x 10^3$) for **11a**. The anisotropic displacement factor exponent takes the form: $-2\pi^2[h^2 a^{*2}U^{11} + ... + 2 h k a^* b^* U^{12}]$

	U^{11}	U^{22}	U^{33}	U^{23}	U^{13}	U^{12}
S	54(1)	49(1)	35(1)	3(1)	2(1)	1(1)
C(1)	60(2)	67(2)	54(2)	2(2)	16(1)	1(2)
C(2)	103(3)	95(3)	69(3)	-30(2)	13(2)	4(3)
C(3)	120(4)	83(3)	82(3)	-23(3)	-9(3)	-29(3)
C(4)	53(2)	105(3)	50(2)	-1(2)	-5(1)	-15(2)
C(5)	44(1)	44(1)	39(1)	3(1)	2(1)	-1(1)
C(6)	32(1)	48(2)	39(1)	0(1)	1(1)	-3(1)
C(7)	51(1)	49(2)	47(2)	-3(1)	0(1)	-2(1)
C(8)	68(2)	58(2)	64(2)	-11(2)	1(2)	1(2)
C(9)	58(2)	88(3)	64(2)	-28(2)	6(2)	1(2)
C(10)	46(2)	108(3)	42(2)	-18(2)	4(1)	-9(2)
C(11)	39(1)	91(2)	38(1)	7(2)	0(1)	-14(1)
C(12)	32(1)	60(2)	39(1)	10(1)	0(1)	-9(1)
C(13)	50(2)	59(2)	51(2)	21(1)	-4(1)	-6(1)
C(14)	58(2)	45(2)	54(2)	6(1)	4(1)	-3(1)
P	76(1)	58(1)	56(1)	6(1)	2(1)	-3(1)
F(1)	147(10)	164(15)	209(19)	63(13)	-93(11)	-56(10)
F(2)	272(19)	110(9)	93(6)	-34(6)	-34(8)	101(11)
F(3)	93(9)	200(20)	195(13)	-42(14)	0(9)	-69(11)
F(4)	185(14)	97(8)	219(14)	-60(8)	-20(12)	47(10)
F(5)	93(5)	234(12)	31(4)	32(5)	6(3)	2(5)
F(6)	267(17)	150(11)	120(10)	100(10)	64(10)	24(10)
F(1A)	85(6)	194(19)	197(13)	21(13)	-48(7)	34(9)
F(2A)	174(9)	77(6)	233(16)	58(8)	37(9)	-29(7)
F(3A)	85(5)	182(15)	240(20)	21(13)	48(8)	6(9)
F(4A)	223(16)	55(4)	257(15)	-14(6)	-73(14)	-18(8)
F(6A)	400(20)	134(9)	66(6)	-49(7)	24(9)	-78(12)
F(5A)	300(20)	400(30)	57(6)	70(10)	37(7)	44(18)

Table S7. Hydrogen coordinates (x 10^4) and isotropic displacement parameters ($Å^2$x 10^3) for **11a**.

	x	y	z	U(eq)
H(1A)	5376	4486	2955	72
H(1B)	5181	2998	3344	72
H(2A)	3268	3345	2405	107
H(2B)	4298	1729	2629	107
H(3A)	1823	1217	3134	114
H(3B)	806	1685	2611	114
H(4A)	-281	3137	3388	84
H(4B)	-157	4171	2882	84
H(7)	2037	7439	4030	59
H(8)	1644	9291	4622	76
H(9)	1746	8958	5431	84
H(10)	2180	6729	5868	79
H(11)	2646	4181	5613	67
H(13)	3176	1892	4967	64
H(14)	3227	1667	4048	63

Table S8. Torsion angles [°] for **11a**.

C(5)-S-C(1)-C(2)	121.0(3)
C(4)-S-C(1)-C(2)	13.3(3)
S-C(1)-C(2)-C(3)	-35.2(5)
C(1)-C(2)-C(3)-C(4)	45.2(6)
C(2)-C(3)-C(4)-S	-33.0(5)
C(5)-S-C(4)-C(3)	-96.9(3)
C(1)-S-C(4)-C(3)	10.6(3)
C(1)-S-C(5)-C(6)	138.7(2)
C(4)-S-C(5)-C(6)	-122.5(3)
C(1)-S-C(5)-C(14)	-42.7(3)
C(4)-S-C(5)-C(14)	56.1(3)
C(14)-C(5)-C(6)-C(7)	-180.0(3)
S-C(5)-C(6)-C(7)	-1.1(4)
C(14)-C(5)-C(6)-C(12)	-0.7(3)
S-C(5)-C(6)-C(12)	178.10(18)
C(5)-C(6)-C(7)-C(8)	179.7(3)
C(12)-C(6)-C(7)-C(8)	0.7(5)
C(6)-C(7)-C(8)-C(9)	0.3(6)
C(7)-C(8)-C(9)-C(10)	-1.0(7)
C(8)-C(9)-C(10)-C(11)	0.1(6)
C(9)-C(10)-C(11)-C(12)	1.0(6)
C(10)-C(11)-C(12)-C(13)	178.7(3)
C(10)-C(11)-C(12)-C(6)	-0.7(5)
C(7)-C(6)-C(12)-C(11)	-0.5(4)
C(5)-C(6)-C(12)-C(11)	-179.7(2)
C(7)-C(6)-C(12)-C(13)	-179.9(3)
C(5)-C(6)-C(12)-C(13)	0.9(3)
C(11)-C(12)-C(13)-C(14)	179.8(3)
C(6)-C(12)-C(13)-C(14)	-0.7(3)
C(12)-C(13)-C(14)-C(5)	0.2(4)
C(6)-C(5)-C(14)-C(13)	0.3(3)
S-C(5)-C(14)-C(13)	-178.4(2)

Table S9. Crystal data and structure refinement for **11b**.

Identification code	s15sel5	
CCDC Number	# 1437061	
Empirical formula	$C_{17} H_{21} F_6 P S$	
Formula weight	402.37	
Temperature	150(2) K	
Wavelength	1.54184 Å	
Crystal system	Monoclinic	
Space group	$P2_1$	
Unit cell dimensions	a = 7.45260(10) Å	α= 90°.
	b = 13.57000(10) Å	β= 92.2460(10)°.
	c = 17.7705(2) Å	γ = 90°.
Volume	1795.78(3) $Å^3$	
Z	4	
Density (calculated)	1.488 Mg/m^3	
Absorption coefficient	2.980 mm^{-1}	
F(000)	832	
Crystal size	0.25 x 0.18 x 0.05 mm^3	
Theta range for data collection	4.100 to 72.576°.	
Index ranges	-5<=h<=9, -16<=k<=16, -21<=l<=21	
Reflections collected	20432	
Independent reflections	6860 [R(int) = 0.0219]	
Completeness to theta = 67.684°	99.6 %	
Absorption correction	Semi-empirical from equivalents	
Max. and min. transmission	1.00000 and 0.70200	
Refinement method	Full-matrix least-squares on F^2	
Data / restraints / parameters	6860 / 7 / 512	
Goodness-of-fit on F^2	1.073	
Final R indices [I>2sigma(I)]	R1 = 0.0376, wR2 = 0.0998	
R indices (all data)	R1 = 0.0380, wR2 = 0.1001	
Absolute structure parameter	0.011(6)	
Extinction coefficient	n/a	
Largest diff. peak and hole	0.517 and -0.328 e.$Å^{-3}$	

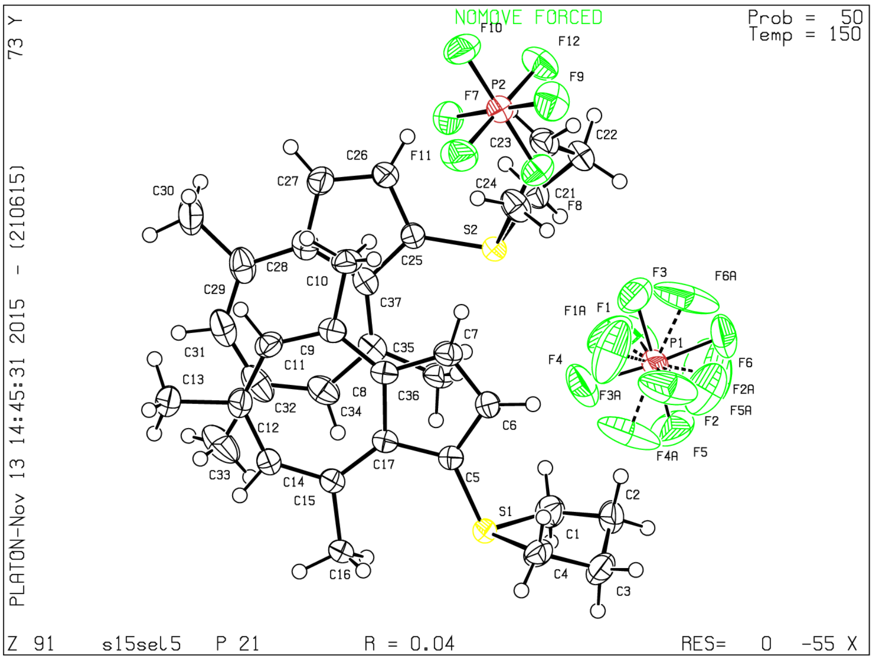

Table S10. Atomic coordinates (x 10^4) and equivalent isotropic displacement parameters (Å^{2}x 10^3) for **11b**. U(eq) is defined as one third of the trace of the orthogonalized U^{ij} tensor.

	x	y	z	U(eq)
S(1)	9998(1)	2710(1)	5566(1)	32(1)
C(1)	8309(6)	3329(4)	4957(2)	43(1)
C(2)	9334(7)	3957(4)	4407(2)	46(1)
C(3)	11045(7)	3414(4)	4264(2)	50(1)
C(4)	11884(6)	3163(4)	5034(2)	41(1)
C(5)	10062(5)	3365(3)	6415(2)	29(1)
C(6)	10063(6)	4397(3)	6440(2)	35(1)
C(7)	9964(5)	4688(3)	7179(2)	33(1)
C(8)	9907(5)	3850(3)	7648(2)	27(1)
C(9)	9742(5)	3905(3)	8435(2)	30(1)
C(10)	9623(6)	4923(3)	8780(2)	38(1)
C(11)	9658(5)	3126(3)	8934(2)	35(1)
C(12)	9761(6)	2116(3)	8803(2)	38(1)
C(13)	9667(9)	1467(4)	9496(3)	55(1)
C(14)	9961(6)	1638(3)	8122(2)	37(1)
C(15)	10073(5)	1980(3)	7387(2)	32(1)
C(16)	10273(9)	1168(3)	6814(3)	59(2)
C(17)	10003(4)	2973(3)	7159(2)	26(1)
S(2)	5164(1)	5826(1)	7137(1)	32(1)
C(21)	3271(6)	6680(3)	7001(2)	37(1)
C(22)	4128(5)	7695(3)	6897(2)	38(1)
C(23)	5708(6)	7752(3)	7457(2)	40(1)
C(24)	6789(5)	6808(3)	7358(3)	42(1)
C(25)	4832(5)	5212(3)	7985(2)	30(1)
C(26)	4590(6)	5720(3)	8666(2)	37(1)
C(27)	4489(6)	5037(3)	9236(2)	40(1)
C(28)	4649(5)	4081(3)	8935(2)	34(1)
C(29)	4567(6)	3231(4)	9384(3)	43(1)
C(30)	4290(8)	3375(5)	10216(3)	61(1)
C(31)	4732(7)	2259(4)	9136(3)	51(1)
C(32)	4982(6)	1879(3)	8427(3)	50(1)
C(33)	5117(9)	770(4)	8356(4)	70(2)
C(34)	5083(6)	2408(3)	7757(3)	40(1)
C(35)	5034(5)	3409(3)	7590(2)	32(1)
C(36)	5147(7)	3657(3)	6767(3)	43(1)
C(37)	4869(5)	4185(3)	8115(2)	28(1)
P(1)	4722(2)	5734(1)	4753(1)	40(1)
F(1)	2677(8)	5648(5)	4962(5)	72(2)
F(2)	6783(9)	5817(5)	4604(5)	99(3)
F(3)	5028(7)	6485(4)	5452(2)	61(1)
F(4)	5121(7)	4839(4)	5319(3)	74(2)
F(5)	4400(14)	5000(6)	4103(4)	109(3)
F(6)	4236(10)	6658(4)	4254(3)	85(2)
F(1A)	3170(30)	5480(19)	5272(11)	76(7)
F(2A)	6260(30)	6070(20)	4198(10)	89(8)
F(3A)	6210(20)	5690(20)	5358(8)	109(9)
F(4A)	4880(20)	4607(13)	4516(19)	98(10)
F(5A)	3270(20)	5880(30)	4073(13)	140(12)
F(6A)	4510(30)	6846(13)	4910(20)	116(9)
P(2)	10450(1)	8422(1)	8463(1)	35(1)
F(7)	9071(4)	7651(2)	8793(2)	56(1)
F(8)	10135(3)	7929(2)	7646(1)	44(1)
F(9)	11821(4)	9184(2)	8122(2)	58(1)
F(10)	10783(4)	8909(3)	9276(2)	60(1)
F(11)	12064(4)	7668(2)	8638(2)	58(1)
F(12)	8832(4)	9168(2)	8286(2)	56(1)

Table S11. Bond lengths [Å] for **11b**.

S(1)-C(5)	1.749(4)	C(23)-C(24)	1.527(6)
S(1)-C(4)	1.830(4)	C(23)-H(23A)	0.9900
S(1)-C(1)	1.832(4)	C(23)-H(23B)	0.9900
C(1)-C(2)	1.523(7)	C(24)-H(24A)	0.9900
C(1)-H(1A)	0.9900	C(24)-H(24B)	0.9900
C(1)-H(1B)	0.9900	C(25)-C(26)	1.411(5)
C(2)-C(3)	1.503(7)	C(25)-C(37)	1.413(5)
C(2)-H(2A)	0.9900	C(26)-C(27)	1.377(6)
C(2)-H(2B)	0.9900	C(26)-H(26)	0.9500
C(3)-C(4)	1.521(6)	C(27)-C(28)	1.410(6)
C(3)-H(3A)	0.9900	C(27)-H(27)	0.9500
C(3)-H(3B)	0.9900	C(28)-C(29)	1.406(6)
C(4)-H(4A)	0.9900	C(28)-C(37)	1.480(6)
C(4)-H(4B)	0.9900	C(29)-C(31)	1.397(7)
C(5)-C(6)	1.401(6)	C(29)-C(30)	1.513(7)
C(5)-C(17)	1.429(5)	C(30)-H(30A)	0.9800
C(6)-C(7)	1.376(6)	C(30)-H(30B)	0.9800
C(6)-H(6)	0.9500	C(30)-H(30C)	0.9800
C(7)-C(8)	1.412(6)	C(31)-C(32)	1.381(8)
C(7)-H(7)	0.9500	C(31)-H(31)	0.9500
C(8)-C(9)	1.411(5)	C(32)-C(34)	1.395(7)
C(8)-C(17)	1.476(5)	C(32)-C(33)	1.514(7)
C(9)-C(11)	1.383(6)	C(33)-H(33A)	0.9800
C(9)-C(10)	1.516(5)	C(33)-H(33B)	0.9800
C(10)-H(10A)	0.9800	C(33)-H(33C)	0.9800
C(10)-H(10B)	0.9800	C(34)-C(35)	1.390(6)
C(10)-H(10C)	0.9800	C(34)-H(34)	0.9500
C(11)-C(12)	1.393(6)	C(35)-C(37)	1.416(6)
C(11)-H(11)	0.9500	C(35)-C(36)	1.506(6)
C(12)-C(14)	1.388(6)	C(36)-H(36A)	0.9800
C(12)-C(13)	1.517(6)	C(36)-H(36B)	0.9800
C(13)-H(13A)	0.9800	C(36)-H(36C)	0.9800
C(13)-H(13B)	0.9800	P(1)-F(3A)	1.517(14)
C(13)-H(13C)	0.9800	P(1)-F(5)	1.536(6)
C(14)-C(15)	1.392(6)	P(1)-F(6A)	1.545(16)
C(14)-H(14)	0.9500	P(1)-F(1A)	1.545(16)
C(15)-C(17)	1.406(5)	P(1)-F(6)	1.569(4)
C(15)-C(16)	1.512(6)	P(1)-F(2)	1.572(6)
C(16)-H(16A)	0.9800	P(1)-F(1)	1.588(7)
C(16)-H(16B)	0.9800	P(1)-F(4A)	1.592(16)
C(16)-H(16C)	0.9800	P(1)-F(4)	1.598(4)
S(2)-C(25)	1.748(4)	P(1)-F(5A)	1.601(16)
S(2)-C(24)	1.833(4)	P(1)-F(2A)	1.605(19)
S(2)-C(21)	1.834(4)	P(1)-F(3)	1.616(4)
C(21)-C(22)	1.533(6)	P(2)-F(9)	1.590(3)
C(21)-H(21A)	0.9900	P(2)-F(7)	1.595(3)
C(21)-H(21B)	0.9900	P(2)-F(12)	1.596(3)
C(22)-C(23)	1.514(6)	P(2)-F(10)	1.598(3)
C(22)-H(22A)	0.9900	P(2)-F(11)	1.601(3)
C(22)-H(22B)	0.9900	P(2)-F(8)	1.609(3)

Table S12. Bond angles [°] for **11b**.

C(5)-S(1)-C(4)	106.2(2)	H(13A)-C(13)-H(13C)	109.5
C(5)-S(1)-C(1)	105.8(2)	H(13B)-C(13)-H(13C)	109.5
C(4)-S(1)-C(1)	93.7(2)	C(12)-C(14)-C(15)	132.5(4)
C(2)-C(1)-S(1)	106.6(3)	C(12)-C(14)-H(14)	113.8
C(2)-C(1)-H(1A)	110.4	C(15)-C(14)-H(14)	113.8
S(1)-C(1)-H(1A)	110.4	C(14)-C(15)-C(17)	125.9(4)
C(2)-C(1)-H(1B)	110.4	C(14)-C(15)-C(16)	113.5(4)
S(1)-C(1)-H(1B)	110.4	C(17)-C(15)-C(16)	120.6(3)
H(1A)-C(1)-H(1B)	108.6	C(15)-C(16)-H(16A)	109.5
C(3)-C(2)-C(1)	106.5(4)	C(15)-C(16)-H(16B)	109.5
C(3)-C(2)-H(2A)	110.4	H(16A)-C(16)-H(16B)	109.5
C(1)-C(2)-H(2A)	110.4	C(15)-C(16)-H(16C)	109.5
C(3)-C(2)-H(2B)	110.4	H(16A)-C(16)-H(16C)	109.5
C(1)-C(2)-H(2B)	110.4	H(16B)-C(16)-H(16C)	109.5
H(2A)-C(2)-H(2B)	108.6	C(15)-C(17)-C(5)	128.4(3)
C(2)-C(3)-C(4)	106.2(4)	C(15)-C(17)-C(8)	127.2(3)
C(2)-C(3)-H(3A)	110.5	C(5)-C(17)-C(8)	104.4(3)
C(4)-C(3)-H(3A)	110.5	C(25)-S(2)-C(24)	106.0(2)
C(2)-C(3)-H(3B)	110.5	C(25)-S(2)-C(21)	106.33(19)
C(4)-C(3)-H(3B)	110.5	C(24)-S(2)-C(21)	93.87(19)
H(3A)-C(3)-H(3B)	108.7	C(22)-C(21)-S(2)	105.2(3)
C(3)-C(4)-S(1)	104.1(3)	C(22)-C(21)-H(21A)	110.7
C(3)-C(4)-H(4A)	110.9	S(2)-C(21)-H(21A)	110.7
S(1)-C(4)-H(4A)	110.9	C(22)-C(21)-H(21B)	110.7
C(3)-C(4)-H(4B)	110.9	S(2)-C(21)-H(21B)	110.7
S(1)-C(4)-H(4B)	110.9	H(21A)-C(21)-H(21B)	108.8
H(4A)-C(4)-H(4B)	109.0	C(23)-C(22)-C(21)	106.4(3)
C(6)-C(5)-C(17)	110.0(3)	C(23)-C(22)-H(22A)	110.4
C(6)-C(5)-S(1)	122.4(3)	C(21)-C(22)-H(22A)	110.4
C(17)-C(5)-S(1)	127.5(3)	C(23)-C(22)-H(22B)	110.4
C(7)-C(6)-C(5)	108.5(4)	C(21)-C(22)-H(22B)	110.4
C(7)-C(6)-H(6)	125.7	H(22A)-C(22)-H(22B)	108.6
C(5)-C(6)-H(6)	125.7	C(22)-C(23)-C(24)	106.4(4)
C(6)-C(7)-C(8)	109.6(4)	C(22)-C(23)-H(23A)	110.4
C(6)-C(7)-H(7)	125.2	C(24)-C(23)-H(23A)	110.4
C(8)-C(7)-H(7)	125.2	C(22)-C(23)-H(23B)	110.4
C(9)-C(8)-C(7)	123.2(3)	C(24)-C(23)-H(23B)	110.4
C(9)-C(8)-C(17)	129.3(3)	H(23A)-C(23)-H(23B)	108.6
C(7)-C(8)-C(17)	107.5(3)	C(23)-C(24)-S(2)	106.7(3)
C(11)-C(9)-C(8)	127.1(4)	C(23)-C(24)-H(24A)	110.4
C(11)-C(9)-C(10)	115.6(4)	S(2)-C(24)-H(24A)	110.4
C(8)-C(9)-C(10)	117.3(4)	C(23)-C(24)-H(24B)	110.4
C(9)-C(10)-H(10A)	109.5	S(2)-C(24)-H(24B)	110.4
C(9)-C(10)-H(10B)	109.5	H(24A)-C(24)-H(24B)	108.6
H(10A)-C(10)-H(10B)	109.5	C(26)-C(25)-C(37)	110.1(3)
C(9)-C(10)-H(10C)	109.5	C(26)-C(25)-S(2)	122.3(3)
H(10A)-C(10)-H(10C)	109.5	C(37)-C(25)-S(2)	127.5(3)
H(10B)-C(10)-H(10C)	109.5	C(27)-C(26)-C(25)	108.4(4)
C(9)-C(11)-C(12)	129.9(4)	C(27)-C(26)-H(26)	125.8
C(9)-C(11)-H(11)	115.1	C(25)-C(26)-H(26)	125.8
C(12)-C(11)-H(11)	115.1	C(26)-C(27)-C(28)	109.4(4)
C(14)-C(12)-C(11)	127.9(4)	C(26)-C(27)-H(27)	125.3
C(14)-C(12)-C(13)	116.6(4)	C(28)-C(27)-H(27)	125.3
C(11)-C(12)-C(13)	115.5(4)	C(29)-C(28)-C(27)	122.2(4)
C(12)-C(13)-H(13A)	109.5	C(29)-C(28)-C(37)	130.3(4)
C(12)-C(13)-H(13B)	109.5	C(27)-C(28)-C(37)	107.5(4)
H(13A)-C(13)-H(13B)	109.5	C(31)-C(29)-C(28)	126.1(4)
C(12)-C(13)-H(13C)	109.5	C(31)-C(29)-C(30)	116.6(4)

C(28)-C(29)-C(30)	117.3(5)	F(5)-P(1)-F(1)	90.3(5)
C(29)-C(30)-H(30A)	109.5	F(6)-P(1)-F(1)	89.3(3)
C(29)-C(30)-H(30B)	109.5	F(2)-P(1)-F(1)	176.1(4)
H(30A)-C(30)-H(30B)	109.5	F(3A)-P(1)-F(4A)	94.9(16)
C(29)-C(30)-H(30C)	109.5	F(6A)-P(1)-F(4A)	175.1(19)
H(30A)-C(30)-H(30C)	109.5	F(1A)-P(1)-F(4A)	90.5(13)
H(30B)-C(30)-H(30C)	109.5	F(5)-P(1)-F(4)	90.0(4)
C(32)-C(31)-C(29)	131.1(4)	F(6)-P(1)-F(4)	174.9(3)
C(32)-C(31)-H(31)	114.5	F(2)-P(1)-F(4)	90.1(4)
C(29)-C(31)-H(31)	114.5	F(1)-P(1)-F(4)	87.3(3)
C(31)-C(32)-C(34)	126.9(4)	F(3A)-P(1)-F(5A)	173.7(15)
C(31)-C(32)-C(33)	117.3(5)	F(6A)-P(1)-F(5A)	87.0(19)
C(34)-C(32)-C(33)	115.8(5)	F(1A)-P(1)-F(5A)	88.9(12)
C(32)-C(33)-H(33A)	109.5	F(4A)-P(1)-F(5A)	88.5(17)
C(32)-C(33)-H(33B)	109.5	F(3A)-P(1)-F(2A)	85.8(10)
H(33A)-C(33)-H(33B)	109.5	F(6A)-P(1)-F(2A)	85.4(13)
C(32)-C(33)-H(33C)	109.5	F(1A)-P(1)-F(2A)	176.0(14)
H(33A)-C(33)-H(33C)	109.5	F(4A)-P(1)-F(2A)	92.6(14)
H(33B)-C(33)-H(33C)	109.5	F(5A)-P(1)-F(2A)	88.7(12)
C(35)-C(34)-C(32)	133.1(5)	F(5)-P(1)-F(3)	178.3(3)
C(35)-C(34)-H(34)	113.5	F(6)-P(1)-F(3)	87.3(3)
C(32)-C(34)-H(34)	113.5	F(2)-P(1)-F(3)	88.6(3)
C(34)-C(35)-C(37)	126.0(4)	F(1)-P(1)-F(3)	88.5(3)
C(34)-C(35)-C(36)	115.0(4)	F(4)-P(1)-F(3)	88.7(3)
C(37)-C(35)-C(36)	118.9(4)	F(9)-P(2)-F(7)	179.10(17)
C(35)-C(36)-H(36A)	109.5	F(9)-P(2)-F(12)	90.25(18)
C(35)-C(36)-H(36B)	109.5	F(7)-P(2)-F(12)	89.75(17)
H(36A)-C(36)-H(36B)	109.5	F(9)-P(2)-F(10)	89.82(18)
C(35)-C(36)-H(36C)	109.5	F(7)-P(2)-F(10)	91.08(18)
H(36A)-C(36)-H(36C)	109.5	F(12)-P(2)-F(10)	90.39(17)
H(36B)-C(36)-H(36C)	109.5	F(9)-P(2)-F(11)	90.06(18)
C(25)-C(37)-C(35)	128.9(4)	F(7)-P(2)-F(11)	89.93(18)
C(25)-C(37)-C(28)	104.6(3)	F(12)-P(2)-F(11)	179.48(18)
C(35)-C(37)-C(28)	126.5(4)	F(10)-P(2)-F(11)	90.03(16)
F(3A)-P(1)-F(6A)	89.4(17)	F(9)-P(2)-F(8)	89.95(15)
F(3A)-P(1)-F(1A)	96.4(11)	F(7)-P(2)-F(8)	89.15(15)
F(6A)-P(1)-F(1A)	91.3(12)	F(12)-P(2)-F(8)	90.14(16)
F(5)-P(1)-F(6)	93.9(4)	F(10)-P(2)-F(8)	179.43(17)
F(5)-P(1)-F(2)	92.6(5)	F(11)-P(2)-F(8)	89.45(15)
F(6)-P(1)-F(2)	93.1(4)		

Table S13. Anisotropic displacement parameters ($Å^2$x 10^3) for **11b**. The anisotropic displacement factor exponent takes the form: $-2\pi^2[h^2 a^{*2}U^{11} + ... + 2 h k a^* b^* U^{12}]$

	U^{11}	U^{22}	U^{33}	U^{23}	U^{13}	U^{12}
S(1)	45(1)	25(1)	27(1)	0(1)	2(1)	0(1)
C(1)	41(2)	49(3)	38(2)	2(2)	-6(2)	3(2)
C(2)	63(3)	42(2)	33(2)	8(2)	-6(2)	3(2)
C(3)	66(3)	54(3)	29(2)	-1(2)	9(2)	-6(2)
C(4)	44(2)	48(2)	32(2)	-1(2)	9(2)	10(2)
C(5)	32(2)	27(2)	29(2)	-1(2)	3(1)	1(2)
C(6)	45(2)	28(2)	33(2)	2(2)	8(2)	-2(2)
C(7)	35(2)	28(2)	37(2)	-4(2)	3(2)	-2(2)
C(8)	22(2)	27(2)	32(2)	-5(2)	-1(1)	1(1)
C(9)	26(2)	30(2)	33(2)	-5(2)	-6(1)	3(2)
C(10)	46(2)	34(2)	35(2)	-10(2)	-1(2)	2(2)
C(11)	38(2)	40(2)	27(2)	-5(2)	-2(2)	5(2)
C(12)	48(2)	37(2)	28(2)	4(2)	-3(2)	0(2)
C(13)	97(4)	39(2)	30(2)	4(2)	2(2)	1(3)
C(14)	52(2)	28(2)	30(2)	2(2)	-1(2)	3(2)
C(15)	38(2)	26(2)	30(2)	-1(2)	0(2)	3(2)
C(16)	120(5)	27(2)	31(2)	1(2)	10(3)	21(3)
C(17)	23(2)	25(2)	29(2)	-3(1)	-1(1)	0(1)
S(2)	43(1)	25(1)	29(1)	1(1)	4(1)	1(1)
C(21)	38(2)	32(2)	40(2)	7(2)	-7(2)	0(2)
C(22)	42(2)	28(2)	45(2)	7(2)	-1(2)	1(2)
C(23)	46(2)	30(2)	44(2)	0(2)	0(2)	-3(2)
C(24)	31(2)	34(2)	59(3)	9(2)	4(2)	-1(2)
C(25)	35(2)	27(2)	29(2)	2(2)	-1(1)	0(2)
C(26)	45(2)	33(2)	34(2)	0(2)	0(2)	10(2)
C(27)	43(2)	45(2)	31(2)	3(2)	4(2)	13(2)
C(28)	25(2)	41(2)	38(2)	6(2)	0(1)	8(2)
C(29)	31(2)	47(3)	50(2)	18(2)	6(2)	2(2)
C(30)	71(3)	63(3)	50(3)	28(3)	17(2)	18(3)
C(31)	52(3)	41(3)	58(3)	21(2)	4(2)	3(2)
C(32)	43(2)	30(2)	76(3)	12(2)	-5(2)	-9(2)
C(33)	85(4)	28(2)	96(5)	8(3)	-5(3)	-6(3)
C(34)	36(2)	30(2)	54(2)	-2(2)	-4(2)	-2(2)
C(35)	23(2)	29(2)	42(2)	1(2)	-2(1)	-3(2)
C(36)	59(3)	28(2)	41(2)	-8(2)	-4(2)	1(2)
C(37)	22(2)	28(2)	36(2)	4(2)	-3(1)	-1(1)
P(1)	49(1)	29(1)	42(1)	5(1)	12(1)	2(1)
F(1)	48(3)	45(2)	122(6)	16(4)	-3(3)	3(2)
F(2)	67(4)	59(3)	177(8)	-28(5)	71(5)	-7(3)
F(3)	78(3)	65(3)	40(2)	-6(2)	11(2)	-14(2)
F(4)	78(3)	52(3)	91(4)	31(3)	6(3)	21(2)
F(5)	205(10)	63(4)	63(4)	-29(3)	33(4)	-42(5)
F(6)	146(6)	49(3)	60(3)	27(2)	-2(3)	-4(3)
F(1A)	73(14)	93(17)	65(11)	-19(10)	46(10)	-43(12)
F(2A)	55(11)	140(20)	71(11)	11(11)	28(8)	-23(11)
F(3A)	79(12)	190(30)	51(8)	29(12)	-27(8)	-27(14)
F(4A)	57(9)	39(9)	200(30)	-38(12)	27(13)	1(7)
F(5A)	61(11)	230(40)	125(17)	50(20)	-47(10)	22(16)
F(6A)	76(11)	46(9)	230(20)	-41(13)	85(14)	-4(8)
P(2)	39(1)	32(1)	33(1)	1(1)	1(1)	3(1)
F(7)	72(2)	45(2)	51(2)	7(1)	23(1)	-3(1)
F(8)	40(1)	54(2)	37(1)	-6(1)	2(1)	-7(1)
F(9)	56(2)	55(2)	62(2)	3(1)	0(1)	-24(1)
F(10)	77(2)	64(2)	40(1)	-15(1)	-2(1)	10(2)
F(11)	58(2)	59(2)	57(2)	-7(2)	-12(1)	23(2)
F(12)	55(2)	41(2)	73(2)	2(1)	-6(1)	13(1)

Table S14. Hydrogen coordinates (x 10^4) and isotropic displacement parameters ($Å^2$x 10^3) for **11b**.

	x	y	z	U(eq)
H(1A)	7553	2838	4681	51
H(1B)	7525	3749	5259	51
H(2A)	8617	4046	3931	55
H(2B)	9601	4613	4626	55
H(3A)	11865	3833	3978	60
H(3B)	10789	2805	3972	60
H(4A)	12429	3753	5277	49
H(4B)	12817	2648	4994	49
H(6)	10121	4824	6018	42
H(7)	9939	5352	7348	40
H(10A)	9706	4869	9331	58
H(10B)	8475	5227	8625	58
H(10C)	10612	5332	8609	58
H(11)	9507	3308	9444	42
H(13A)	9094	840	9357	83
H(13B)	8962	1800	9875	83
H(13C)	10884	1344	9703	83
H(14)	10035	941	8165	44
H(16A)	11382	1268	6546	89
H(16B)	9243	1179	6454	89
H(16C)	10322	529	7072	89
H(21A)	2523	6495	6550	44
H(21B)	2510	6677	7445	44
H(22A)	4537	7770	6376	46
H(22B)	3251	8224	6995	46
H(23A)	5285	7798	7977	48
H(23B)	6454	8337	7356	48
H(24A)	7493	6652	7827	50
H(24B)	7628	6885	6944	50
H(26)	4510	6415	8723	45
H(27)	4336	5187	9752	48
H(30A)	4199	2731	10461	92
H(30B)	3182	3749	10283	92
H(30C)	5310	3740	10443	92
H(31)	4658	1777	9521	61
H(33A)	4922	580	7828	105
H(33B)	4204	459	8660	105
H(33C)	6313	554	8536	105
H(34)	5212	2002	7327	48
H(36A)	4125	4073	6609	65
H(36B)	5121	3048	6470	65
H(36C)	6268	4011	6685	65

Table S15. Torsion angles [°] for **11b**.

C(5)-S(1)-C(1)-C(2)	102.1(3)
C(4)-S(1)-C(1)-C(2)	-5.9(4)
S(1)-C(1)-C(2)-C(3)	32.7(4)
C(1)-C(2)-C(3)-C(4)	-50.7(5)
C(2)-C(3)-C(4)-S(1)	44.5(5)
C(5)-S(1)-C(4)-C(3)	-129.3(3)
C(1)-S(1)-C(4)-C(3)	-21.7(4)
C(4)-S(1)-C(5)-C(6)	54.8(4)
C(1)-S(1)-C(5)-C(6)	-43.9(4)
C(4)-S(1)-C(5)-C(17)	-129.6(3)
C(1)-S(1)-C(5)-C(17)	131.7(3)
C(17)-C(5)-C(6)-C(7)	-1.4(5)
S(1)-C(5)-C(6)-C(7)	174.9(3)
C(5)-C(6)-C(7)-C(8)	0.3(5)
C(6)-C(7)-C(8)-C(9)	-177.8(4)
C(6)-C(7)-C(8)-C(17)	0.9(4)
C(7)-C(8)-C(9)-C(11)	179.3(4)
C(17)-C(8)-C(9)-C(11)	1.0(6)
C(7)-C(8)-C(9)-C(10)	0.0(5)
C(17)-C(8)-C(9)-C(10)	-178.3(4)
C(8)-C(9)-C(11)-C(12)	2.0(7)
C(10)-C(9)-C(11)-C(12)	-178.7(4)
C(9)-C(11)-C(12)-C(14)	-0.7(8)
C(9)-C(11)-C(12)-C(13)	178.4(4)
C(11)-C(12)-C(14)-C(15)	-1.7(9)
C(13)-C(12)-C(14)-C(15)	179.2(5)
C(12)-C(14)-C(15)-C(17)	0.1(8)
C(12)-C(14)-C(15)-C(16)	-179.5(5)
C(14)-C(15)-C(17)-C(5)	-178.1(4)
C(16)-C(15)-C(17)-C(5)	1.4(7)
C(14)-C(15)-C(17)-C(8)	3.9(7)
C(16)-C(15)-C(17)-C(8)	-176.6(4)
C(6)-C(5)-C(17)-C(15)	-176.5(4)
S(1)-C(5)-C(17)-C(15)	7.4(6)
C(6)-C(5)-C(17)-C(8)	1.8(4)
S(1)-C(5)-C(17)-C(8)	-174.2(3)
C(9)-C(8)-C(17)-C(15)	-4.7(6)
C(7)-C(8)-C(17)-C(15)	176.8(4)
C(9)-C(8)-C(17)-C(5)	177.0(4)
C(7)-C(8)-C(17)-C(5)	-1.6(4)
C(25)-S(2)-C(21)-C(22)	125.1(3)
C(24)-S(2)-C(21)-C(22)	17.1(3)
S(2)-C(21)-C(22)-C(23)	-40.5(4)
C(21)-C(22)-C(23)-C(24)	49.6(4)
C(22)-C(23)-C(24)-S(2)	-35.4(4)
C(25)-S(2)-C(24)-C(23)	-98.0(3)
C(21)-S(2)-C(24)-C(23)	10.2(3)
C(24)-S(2)-C(25)-C(26)	44.3(4)
C(21)-S(2)-C(25)-C(26)	-54.7(4)
C(24)-S(2)-C(25)-C(37)	-131.5(3)
C(21)-S(2)-C(25)-C(37)	129.4(3)
C(37)-C(25)-C(26)-C(27)	0.6(5)
S(2)-C(25)-C(26)-C(27)	-175.9(3)
C(25)-C(26)-C(27)-C(28)	-0.5(5)
C(26)-C(27)-C(28)-C(29)	-179.0(4)
C(26)-C(27)-C(28)-C(37)	0.1(5)
C(27)-C(28)-C(29)-C(31)	-179.2(5)
C(37)-C(28)-C(29)-C(31)	1.8(7)

C(27)-C(28)-C(29)-C(30)	0.2(6)
C(37)-C(28)-C(29)-C(30)	-178.7(4)
C(28)-C(29)-C(31)-C(32)	-0.8(9)
C(30)-C(29)-C(31)-C(32)	179.7(5)
C(29)-C(31)-C(32)-C(34)	-2.1(9)
C(29)-C(31)-C(32)-C(33)	179.6(5)
C(31)-C(32)-C(34)-C(35)	2.4(9)
C(33)-C(32)-C(34)-C(35)	-179.3(5)
C(32)-C(34)-C(35)-C(37)	0.6(7)
C(32)-C(34)-C(35)-C(36)	-178.6(5)
C(26)-C(25)-C(37)-C(35)	178.1(4)
S(2)-C(25)-C(37)-C(35)	-5.7(6)
C(26)-C(25)-C(37)-C(28)	-0.5(4)
S(2)-C(25)-C(37)-C(28)	175.7(3)
C(34)-C(35)-C(37)-C(25)	179.2(4)
C(36)-C(35)-C(37)-C(25)	-1.7(6)
C(34)-C(35)-C(37)-C(28)	-2.5(6)
C(36)-C(35)-C(37)-C(28)	176.7(4)
C(29)-C(28)-C(37)-C(25)	179.3(4)
C(27)-C(28)-C(37)-C(25)	0.2(4)
C(29)-C(28)-C(37)-C(35)	0.7(6)
C(27)-C(28)-C(37)-C(35)	-178.4(4)

Table S16. Crystal data and structure refinement for **11c**.

Identification code	e15sel3	
CCDC Number	# 1440661	
Empirical formula	$C_{18} H_{23} F_6 P S$	
Formula weight	416.39	
Temperature	150(2) K	
Wavelength	0.71073 Å	
Crystal system	Monoclinic	
Space group	$P2_1$	
Unit cell dimensions	a = 10.3988(5) Å	α= 90°.
	b = 8.4387(3) Å	β= 99.950(4)°.
	c = 10.9923(4) Å	γ = 90°.
Volume	950.09(7) $Å^3$	
Z	2	
Density (calculated)	1.456 Mg/m^3	
Absorption coefficient	0.310 mm^{-1}	
F(000)	432	
Crystal size	0.350 x 0.200 x 0.150 mm^3	
Theta range for data collection	3.469 to 30.078°.	
Index ranges	-13<=h<=14, -11<=k<=11, -13<=l<=15	
Reflections collected	8526	
Independent reflections	4570 [R(int) = 0.0244]	
Completeness to theta = 25.242°	99.7 %	
Absorption correction	Semi-empirical from equivalents	
Max. and min. transmission	1.00000 and 0.95173	
Refinement method	Full-matrix least-squares on F^2	
Data / restraints / parameters	4570 / 1 / 238	
Goodness-of-fit on F^2	1.031	
Final R indices [I>2sigma(I)]	R1 = 0.0385, wR2 = 0.0740	
R indices (all data)	R1 = 0.0461, wR2 = 0.0771	
Absolute structure parameter	0.45(4)	
Extinction coefficient	n/a	
Largest diff. peak and hole	0.249 and -0.262 e.$Å^{-3}$	

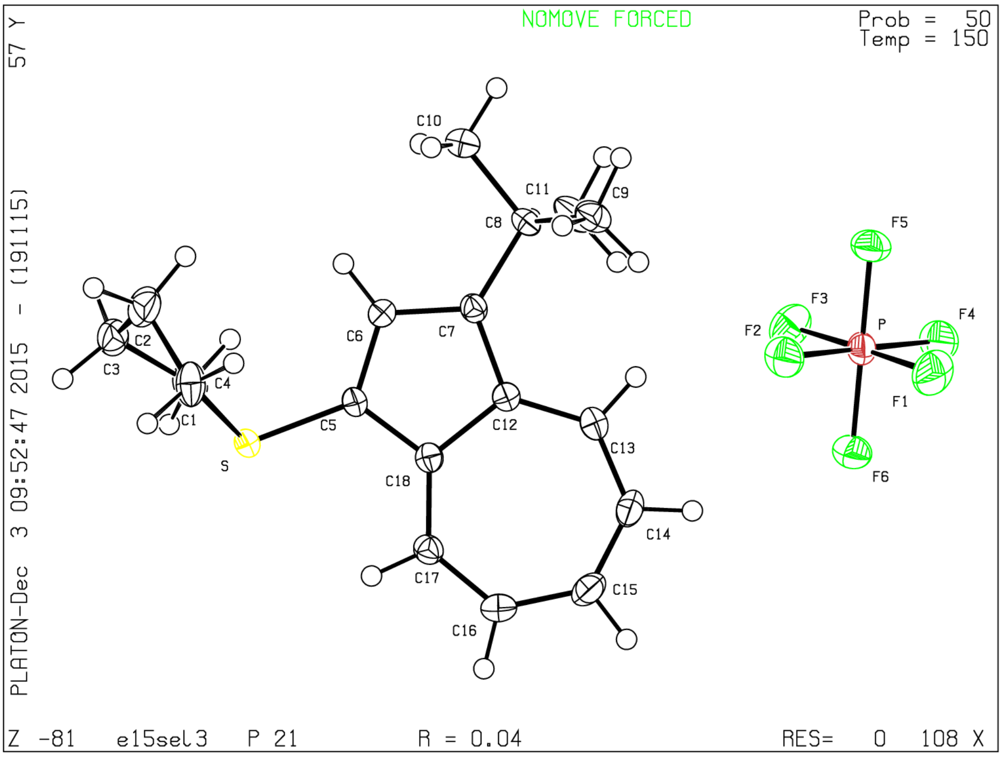

Table S17. Atomic coordinates (x 10^4) and equivalent isotropic displacement parameters (Å^{2}x 10^3) for **11c**. U(eq) is defined as one third of the trace of the orthogonalized U^{ij} tensor.

	x	y	z	U(eq)
S	7093(1)	1643(1)	7889(1)	23(1)
C(1)	8718(3)	1180(4)	8721(3)	30(1)
C(2)	8951(3)	2262(5)	9833(3)	39(1)
C(3)	7660(3)	2453(4)	10270(2)	32(1)
C(4)	6652(3)	2834(4)	9146(3)	33(1)
C(5)	7323(3)	3013(3)	6760(2)	20(1)
C(6)	8074(3)	4404(3)	6903(2)	22(1)
C(7)	7992(3)	5180(3)	5776(2)	19(1)
C(8)	8659(3)	6752(4)	5596(2)	22(1)
C(9)	9534(3)	6620(4)	4604(2)	28(1)
C(10)	9539(4)	7241(4)	6809(3)	37(1)
C(11)	7631(3)	8041(4)	5244(3)	34(1)
C(12)	7157(3)	4257(3)	4881(2)	18(1)
C(13)	6735(3)	4627(3)	3642(2)	22(1)
C(14)	5924(3)	3782(4)	2724(2)	27(1)
C(15)	5334(3)	2329(4)	2786(2)	30(1)
C(16)	5356(3)	1338(4)	3802(2)	28(1)
C(17)	5953(3)	1586(4)	5012(2)	21(1)
C(18)	6735(3)	2839(3)	5512(2)	19(1)
P	7293(1)	7490(1)	635(1)	24(1)
F(1)	7873(2)	7014(2)	-569(2)	47(1)
F(2)	8147(2)	6128(2)	1398(2)	38(1)
F(3)	6695(2)	7939(2)	1832(2)	41(1)
F(4)	6427(2)	8845(2)	-131(2)	44(1)
F(5)	8465(2)	8715(2)	1029(2)	38(1)
F(6)	6124(2)	6249(2)	238(2)	33(1)

Table S18. Bond lengths [Å] for **11c**.

S-C(5)	1.742(3)
S-C(1)	1.819(3)
S-C(4)	1.829(3)
C(1)-C(2)	1.511(4)
C(1)-H(1A)	0.9900
C(1)-H(1B)	0.9900
C(2)-C(3)	1.511(5)
C(2)-H(2A)	0.9900
C(2)-H(2B)	0.9900
C(3)-C(4)	1.511(4)
C(3)-H(3A)	0.9900
C(3)-H(3B)	0.9900
C(4)-H(4A)	0.9900
C(4)-H(4B)	0.9900
C(5)-C(6)	1.404(4)
C(5)-C(18)	1.410(3)
C(6)-C(7)	1.391(4)
C(6)-H(6)	0.9500
C(7)-C(12)	1.426(4)
C(7)-C(8)	1.526(4)
C(8)-C(11)	1.528(4)
C(8)-C(10)	1.538(4)
C(8)-C(9)	1.540(4)
C(9)-H(9A)	0.9800
C(9)-H(9B)	0.9800
C(9)-H(9C)	0.9800
C(10)-H(10A)	0.9800
C(10)-H(10B)	0.9800
C(10)-H(10C)	0.9800
C(11)-H(11A)	0.9800
C(11)-H(11B)	0.9800
C(11)-H(11C)	0.9800
C(12)-C(13)	1.392(3)
C(12)-C(18)	1.488(4)
C(13)-C(14)	1.394(4)
C(13)-H(13)	0.9500
C(14)-C(15)	1.379(4)
C(14)-H(14)	0.9500
C(15)-C(16)	1.392(4)
C(15)-H(15)	0.9500
C(16)-C(17)	1.383(4)
C(16)-H(16)	0.9500
C(17)-C(18)	1.388(4)
C(17)-H(17)	0.9500
P-F(3)	1.5952(18)
P-F(1)	1.5973(18)
P-F(2)	1.599(2)
P-F(5)	1.600(2)
P-F(4)	1.601(2)
P-F(6)	1.6062(19)

Table S19. Bond angles [°] for **11c**.

C(5)-S-C(1)	105.66(14)	C(8)-C(10)-H(10A)	109.5
C(5)-S-C(4)	104.87(14)	C(8)-C(10)-H(10B)	109.5
C(1)-S-C(4)	94.06(15)	H(10A)-C(10)-H(10B)	109.5
C(2)-C(1)-S	106.4(2)	C(8)-C(10)-H(10C)	109.5
C(2)-C(1)-H(1A)	110.5	H(10A)-C(10)-H(10C)	109.5
S-C(1)-H(1A)	110.5	H(10B)-C(10)-H(10C)	109.5
C(2)-C(1)-H(1B)	110.5	C(8)-C(11)-H(11A)	109.5
S-C(1)-H(1B)	110.5	C(8)-C(11)-H(11B)	109.5
H(1A)-C(1)-H(1B)	108.6	H(11A)-C(11)-H(11B)	109.5
C(3)-C(2)-C(1)	107.1(3)	C(8)-C(11)-H(11C)	109.5
C(3)-C(2)-H(2A)	110.3	H(11A)-C(11)-H(11C)	109.5
C(1)-C(2)-H(2A)	110.3	H(11B)-C(11)-H(11C)	109.5
C(3)-C(2)-H(2B)	110.3	C(13)-C(12)-C(7)	127.2(2)
C(1)-C(2)-H(2B)	110.3	C(13)-C(12)-C(18)	124.6(3)
H(2A)-C(2)-H(2B)	108.5	C(7)-C(12)-C(18)	108.1(2)
C(4)-C(3)-C(2)	107.0(2)	C(12)-C(13)-C(14)	130.0(3)
C(4)-C(3)-H(3A)	110.3	C(12)-C(13)-H(13)	115.0
C(2)-C(3)-H(3A)	110.3	C(14)-C(13)-H(13)	115.0
C(4)-C(3)-H(3B)	110.3	C(15)-C(14)-C(13)	129.6(3)
C(2)-C(3)-H(3B)	110.3	C(15)-C(14)-H(14)	115.2
H(3A)-C(3)-H(3B)	108.6	C(13)-C(14)-H(14)	115.2
C(3)-C(4)-S	105.8(2)	C(14)-C(15)-C(16)	128.9(3)
C(3)-C(4)-H(4A)	110.6	C(14)-C(15)-H(15)	115.5
S-C(4)-H(4A)	110.6	C(16)-C(15)-H(15)	115.5
C(3)-C(4)-H(4B)	110.6	C(17)-C(16)-C(15)	128.5(3)
S-C(4)-H(4B)	110.6	C(17)-C(16)-H(16)	115.7
H(4A)-C(4)-H(4B)	108.7	C(15)-C(16)-H(16)	115.7
C(6)-C(5)-C(18)	109.6(2)	C(16)-C(17)-C(18)	129.1(3)
C(6)-C(5)-S	128.1(2)	C(16)-C(17)-H(17)	115.4
C(18)-C(5)-S	122.3(2)	C(18)-C(17)-H(17)	115.4
C(7)-C(6)-C(5)	110.3(2)	C(17)-C(18)-C(5)	126.2(2)
C(7)-C(6)-H(6)	124.9	C(17)-C(18)-C(12)	128.9(2)
C(5)-C(6)-H(6)	124.9	C(5)-C(18)-C(12)	104.9(2)
C(6)-C(7)-C(12)	107.1(2)	F(3)-P-F(1)	178.95(13)
C(6)-C(7)-C(8)	124.4(2)	F(3)-P-F(2)	89.91(10)
C(12)-C(7)-C(8)	128.4(2)	F(1)-P-F(2)	89.92(11)
C(7)-C(8)-C(11)	109.8(2)	F(3)-P-F(5)	90.59(11)
C(7)-C(8)-C(10)	109.7(2)	F(1)-P-F(5)	90.45(11)
C(11)-C(8)-C(10)	108.3(3)	F(2)-P-F(5)	89.67(10)
C(7)-C(8)-C(9)	111.5(2)	F(3)-P-F(4)	90.02(11)
C(11)-C(8)-C(9)	110.2(2)	F(1)-P-F(4)	90.14(11)
C(10)-C(8)-C(9)	107.3(2)	F(2)-P-F(4)	179.54(13)
C(8)-C(9)-H(9A)	109.5	F(5)-P-F(4)	90.79(11)
C(8)-C(9)-H(9B)	109.5	F(3)-P-F(6)	89.69(10)
H(9A)-C(9)-H(9B)	109.5	F(1)-P-F(6)	89.27(10)
C(8)-C(9)-H(9C)	109.5	F(2)-P-F(6)	89.96(11)
H(9A)-C(9)-H(9C)	109.5	F(5)-P-F(6)	179.53(12)
H(9B)-C(9)-H(9C)	109.5	F(4)-P-F(6)	89.59(10)

Table S20. Anisotropic displacement parameters ($Å^2 x 10^3$) for **11c**. The anisotropic displacement factor exponent takes the form: $-2\pi^2[h^2 a^{*2}U^{11} + ... + 2 h k a^* b^* U^{12}]$

	U^{11}	U^{22}	U^{33}	U^{23}	U^{13}	U^{12}
S	27(1)	21(1)	19(1)	5(1)	1(1)	-6(1)
C(1)	28(2)	36(2)	27(1)	11(1)	6(1)	12(1)
C(2)	33(2)	53(2)	27(1)	9(2)	-5(1)	-6(2)
C(3)	49(2)	26(2)	22(1)	-2(1)	9(1)	-1(2)
C(4)	34(2)	36(2)	33(2)	4(1)	18(1)	7(2)
C(5)	23(2)	19(1)	18(1)	4(1)	2(1)	-2(1)
C(6)	27(2)	21(1)	18(1)	1(1)	0(1)	-4(1)
C(7)	22(2)	16(1)	19(1)	1(1)	5(1)	1(1)
C(8)	27(2)	17(1)	23(1)	2(1)	6(1)	-6(1)
C(9)	28(2)	26(1)	33(1)	0(1)	11(1)	-6(1)
C(10)	49(2)	31(2)	28(1)	-1(1)	3(2)	-19(2)
C(11)	38(2)	18(2)	47(2)	4(1)	16(2)	-1(1)
C(12)	19(2)	18(1)	19(1)	0(1)	5(1)	2(1)
C(13)	23(2)	21(1)	23(1)	4(1)	6(1)	2(1)
C(14)	27(2)	36(2)	17(1)	3(1)	2(1)	1(1)
C(15)	30(2)	37(2)	21(1)	-4(1)	-2(1)	-4(2)
C(16)	26(2)	28(2)	29(1)	-3(1)	1(1)	-8(1)
C(17)	20(1)	20(1)	24(1)	3(1)	2(1)	0(1)
C(18)	18(2)	19(1)	20(1)	1(1)	4(1)	2(1)
P	25(1)	25(1)	22(1)	4(1)	3(1)	0(1)
F(1)	46(1)	68(2)	30(1)	-7(1)	18(1)	-10(1)
F(2)	34(1)	31(1)	44(1)	9(1)	-3(1)	6(1)
F(3)	41(1)	53(1)	32(1)	-8(1)	10(1)	4(1)
F(4)	45(1)	34(1)	46(1)	15(1)	-14(1)	1(1)
F(5)	34(1)	32(1)	43(1)	5(1)	-5(1)	-10(1)
F(6)	29(1)	32(1)	38(1)	-1(1)	5(1)	-5(1)

Table S21. Hydrogen coordinates (x 10^4) and isotropic displacement parameters ($Å^2$x 10 ^{3}) for **11c**.

	x	y	z	U(eq)
H(1A)	8768	57	8984	36
H(1B)	9380	1369	8190	36
H(2A)	9607	1793	10495	47
H(2B)	9276	3305	9606	47
H(3A)	7425	1463	10662	38
H(3B)	7712	3322	10882	38
H(4A)	6669	3977	8947	39
H(4B)	5766	2554	9290	39
H(6)	8568	4764	7661	27
H(9A)	10021	7610	4575	43
H(9B)	8990	6426	3797	43
H(9C)	10149	5741	4809	43
H(10A)	9970	8248	6689	55
H(10B)	10201	6421	7055	55
H(10C)	9007	7366	7457	55
H(11A)	8064	9063	5186	50
H(11B)	7074	8104	5876	50
H(11C)	7095	7783	4445	50
H(13)	7048	5606	3381	27
H(14)	5753	4283	1939	32
H(15)	4844	1952	2032	36
H(16)	4901	363	3643	34
H(17)	5807	786	5581	26

Table S22. Torsion angles [°] for **11c**.

C(5)-S-C(1)-C(2)	94.8(2)
C(4)-S-C(1)-C(2)	-11.9(2)
S-C(1)-C(2)-C(3)	35.6(3)
C(1)-C(2)-C(3)-C(4)	-48.1(4)
C(2)-C(3)-C(4)-S	37.5(3)
C(5)-S-C(4)-C(3)	-122.0(2)
C(1)-S-C(4)-C(3)	-14.6(2)
C(1)-S-C(5)-C(6)	-49.3(3)
C(4)-S-C(5)-C(6)	49.4(3)
C(1)-S-C(5)-C(18)	130.8(2)
C(4)-S-C(5)-C(18)	-130.5(2)
C(18)-C(5)-C(6)-C(7)	0.6(3)
S-C(5)-C(6)-C(7)	-179.3(2)
C(5)-C(6)-C(7)-C(12)	0.2(3)
C(5)-C(6)-C(7)-C(8)	178.4(3)
C(6)-C(7)-C(8)-C(11)	-112.9(3)
C(12)-C(7)-C(8)-C(11)	65.0(4)
C(6)-C(7)-C(8)-C(10)	5.9(4)
C(12)-C(7)-C(8)-C(10)	-176.2(3)
C(6)-C(7)-C(8)-C(9)	124.7(3)
C(12)-C(7)-C(8)-C(9)	-57.4(4)
C(6)-C(7)-C(12)-C(13)	175.9(3)
C(8)-C(7)-C(12)-C(13)	-2.2(5)
C(6)-C(7)-C(12)-C(18)	-0.8(3)
C(8)-C(7)-C(12)-C(18)	-179.0(3)
C(7)-C(12)-C(13)-C(14)	-180.0(3)
C(18)-C(12)-C(13)-C(14)	-3.7(5)
C(12)-C(13)-C(14)-C(15)	-1.3(6)
C(13)-C(14)-C(15)-C(16)	2.5(6)
C(14)-C(15)-C(16)-C(17)	1.7(6)
C(15)-C(16)-C(17)-C(18)	-3.7(6)
C(16)-C(17)-C(18)-C(5)	-179.5(3)
C(16)-C(17)-C(18)-C(12)	-0.7(5)
C(6)-C(5)-C(18)-C(17)	178.0(3)
S-C(5)-C(18)-C(17)	-2.0(4)
C(6)-C(5)-C(18)-C(12)	-1.0(3)
S-C(5)-C(18)-C(12)	178.9(2)
C(13)-C(12)-C(18)-C(17)	5.2(5)
C(7)-C(12)-C(18)-C(17)	-177.9(3)
C(13)-C(12)-C(18)-C(5)	-175.7(3)
C(7)-C(12)-C(18)-C(5)	1.1(3)

Table S23. Crystal data and structure refinement for **11d**.

Identification code	e15sel2	
CCDC Number	#	
Empirical formula	$C_{17}H_{23}F_6PS$	
Formula weight	404.38	
Temperature	150(2) K	
Wavelength	0.71073 Å	
Crystal system	Monoclinic	
Space group	$P2_1/n$	
Unit cell dimensions	a = 8.0188(5) Å	α= 90°.
	b = 9.7662(7) Å	β= 93.982(5)°.
	c = 24.2767(14) Å	γ = 90°.
Volume	1896.6(2) $Å^3$	
Z	4	
Density (calculated)	1.416 Mg/m^3	
Absorption coefficient	0.308 mm^{-1}	
F(000)	840	
Crystal size	0.360 x 0.270 x 0.080 mm^3	
Theta range for data collection	3.274 to 26.416°.	
Index ranges	-10<=h<=10, -12<=k<=12, -30<=l<=30	
Reflections collected	23034	
Independent reflections	3875 [R(int) = 0.0768]	
Completeness to theta = 25.242°	99.6 %	
Absorption correction	Analytical	
Max. and min. transmission	0.979 and 0.919	
Refinement method	Full-matrix least-squares on F^2	
Data / restraints / parameters	3875 / 0 / 268	
Goodness-of-fit on F^2	1.048	
Final R indices [I>2sigma(I)]	R1 = 0.0669, wR2 = 0.1673	
R indices (all data)	R1 = 0.0976, wR2 = 0.1900	
Extinction coefficient	n/a	
Largest diff. peak and hole	1.117 and -0.316 e.$Å^{-3}$	

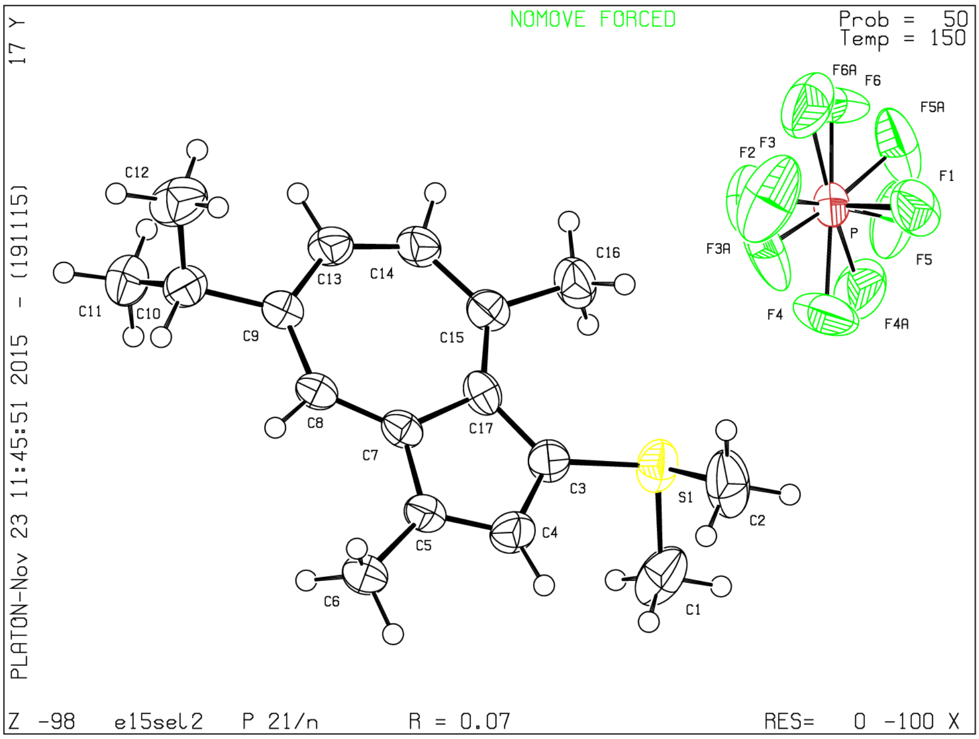

Table S24. Atomic coordinates (x 10^4) and equivalent isotropic displacement parameters ($Å^2$x 10^3) for **11d**. U(eq) is defined as one third of the trace of the orthogonalized U^{ij} tensor.

	x	y	z	U(eq)
S(1)	3274(1)	6609(1)	4352(1)	54(1)
C(1)	1047(6)	6339(5)	4303(2)	71(1)
C(2)	3379(7)	8270(5)	4659(2)	77(1)
C(3)	3720(4)	6899(3)	3670(1)	41(1)
C(4)	2763(4)	7821(4)	3323(2)	46(1)
C(5)	3312(4)	7782(3)	2803(1)	38(1)
C(6)	2583(4)	8638(4)	2324(2)	49(1)
C(7)	4644(4)	6849(3)	2796(1)	35(1)
C(8)	5468(4)	6585(3)	2319(1)	33(1)
C(9)	6804(4)	5741(3)	2234(1)	35(1)
C(10)	7455(4)	5689(4)	1657(1)	47(1)
C(11)	7179(5)	4269(4)	1401(2)	60(1)
C(12)	9259(5)	6136(5)	1656(2)	65(1)
C(13)	7625(4)	4942(3)	2638(1)	43(1)
C(14)	7357(4)	4768(4)	3192(1)	44(1)
C(15)	6174(4)	5310(3)	3530(1)	40(1)
C(16)	6324(5)	4808(4)	4122(1)	56(1)
C(17)	4939(4)	6250(3)	3361(1)	37(1)
P	8328(1)	7779(1)	5563(1)	45(1)
F(1)	8524(3)	8994(3)	5994(1)	79(1)
F(2)	8137(4)	6531(3)	5143(1)	83(1)
F(3)	8938(16)	8741(13)	5118(5)	111(4)
F(4)	6460(9)	8394(10)	5396(4)	97(3)
F(5)	7564(14)	6803(7)	5999(3)	89(2)
F(6)	10030(9)	7186(8)	5688(5)	90(3)
F(3A)	7971(16)	8684(14)	5058(3)	109(5)
F(4A)	6561(9)	7575(15)	5682(4)	124(4)
F(5A)	8964(15)	6838(7)	6087(2)	90(2)
F(6A)	10342(10)	7904(12)	5491(4)	103(4)

Table S25. Bond lengths [Å] for **11d**.

S(1)-C(3)	1.742(3)
S(1)-C(2)	1.785(4)
S(1)-C(1)	1.801(5)
C(1)-H(1A)	0.9800
C(1)-H(1B)	0.9800
C(1)-H(1C)	0.9800
C(2)-H(2A)	0.9800
C(2)-H(2B)	0.9800
C(2)-H(2C)	0.9800
C(3)-C(4)	1.421(5)
C(3)-C(17)	1.422(5)
C(4)-C(5)	1.366(5)
C(4)-H(4)	0.9500
C(5)-C(7)	1.405(4)
C(5)-C(6)	1.517(4)
C(6)-H(6A)	0.9800
C(6)-H(6B)	0.9800
C(6)-H(6C)	0.9800
C(7)-C(8)	1.396(4)
C(7)-C(17)	1.496(4)
C(8)-C(9)	1.378(4)
C(8)-H(8)	0.9500
C(9)-C(13)	1.384(4)
C(9)-C(10)	1.531(4)
C(10)-C(12)	1.511(5)
C(10)-C(11)	1.530(6)
C(10)-H(10)	1.0000
C(11)-H(11A)	0.9800
C(11)-H(11B)	0.9800
C(11)-H(11C)	0.9800
C(12)-H(12A)	0.9800
C(12)-H(12B)	0.9800
C(12)-H(12C)	0.9800
C(13)-C(14)	1.387(5)
C(13)-H(13)	0.9500
C(14)-C(15)	1.400(5)
C(14)-H(14)	0.9500
C(15)-C(17)	1.391(4)
C(15)-C(16)	1.516(5)
C(16)-H(16A)	0.9800
C(16)-H(16B)	0.9800
C(16)-H(16C)	0.9800
P-F(4A)	1.480(6)
P-F(6)	1.495(6)
P-F(3A)	1.522(9)
P-F(3)	1.536(10)
P-F(5)	1.581(6)
P-F(1)	1.584(2)
P-F(2)	1.589(2)
P-F(5A)	1.624(6)
P-F(4)	1.638(6)
P-F(6A)	1.640(8)

Table S26. Bond angles [°] for **11d**.

C(3)-S(1)-C(2)	104.0(2)	C(10)-C(12)-H(12B)	109.5
C(3)-S(1)-C(1)	103.22(19)	H(12A)-C(12)-H(12B)	109.5
C(2)-S(1)-C(1)	100.3(2)	C(10)-C(12)-H(12C)	109.5
S(1)-C(1)-H(1A)	109.5	H(12A)-C(12)-H(12C)	109.5
S(1)-C(1)-H(1B)	109.5	H(12B)-C(12)-H(12C)	109.5
H(1A)-C(1)-H(1B)	109.5	C(9)-C(13)-C(14)	131.1(3)
S(1)-C(1)-H(1C)	109.5	C(9)-C(13)-H(13)	114.4
H(1A)-C(1)-H(1C)	109.5	C(14)-C(13)-H(13)	114.4
H(1B)-C(1)-H(1C)	109.5	C(13)-C(14)-C(15)	132.7(3)
S(1)-C(2)-H(2A)	109.5	C(13)-C(14)-H(14)	113.6
S(1)-C(2)-H(2B)	109.5	C(15)-C(14)-H(14)	113.6
H(2A)-C(2)-H(2B)	109.5	C(17)-C(15)-C(14)	125.1(3)
S(1)-C(2)-H(2C)	109.5	C(17)-C(15)-C(16)	120.0(3)
H(2A)-C(2)-H(2C)	109.5	C(14)-C(15)-C(16)	114.9(3)
H(2B)-C(2)-H(2C)	109.5	C(15)-C(16)-H(16A)	109.5
C(4)-C(3)-C(17)	109.5(3)	C(15)-C(16)-H(16B)	109.5
C(4)-C(3)-S(1)	121.9(3)	H(16A)-C(16)-H(16B)	109.5
C(17)-C(3)-S(1)	128.5(3)	C(15)-C(16)-H(16C)	109.5
C(5)-C(4)-C(3)	109.5(3)	H(16A)-C(16)-H(16C)	109.5
C(5)-C(4)-H(4)	125.2	H(16B)-C(16)-H(16C)	109.5
C(3)-C(4)-H(4)	125.2	C(15)-C(17)-C(3)	129.9(3)
C(4)-C(5)-C(7)	108.9(3)	C(15)-C(17)-C(7)	126.3(3)
C(4)-C(5)-C(6)	124.2(3)	C(3)-C(17)-C(7)	103.8(3)
C(7)-C(5)-C(6)	126.9(3)	F(4A)-P-F(3A)	96.2(7)
C(5)-C(6)-H(6A)	109.5	F(6)-P-F(3)	92.8(6)
C(5)-C(6)-H(6B)	109.5	F(6)-P-F(5)	91.2(5)
H(6A)-C(6)-H(6B)	109.5	F(3)-P-F(5)	175.7(6)
C(5)-C(6)-H(6C)	109.5	F(4A)-P-F(1)	91.3(4)
H(6A)-C(6)-H(6C)	109.5	F(6)-P-F(1)	96.2(3)
H(6B)-C(6)-H(6C)	109.5	F(3A)-P-F(1)	95.9(5)
C(8)-C(7)-C(5)	122.3(3)	F(3)-P-F(1)	89.1(5)
C(8)-C(7)-C(17)	129.4(3)	F(5)-P-F(1)	91.9(3)
C(5)-C(7)-C(17)	108.3(3)	F(4A)-P-F(2)	88.4(4)
C(9)-C(8)-C(7)	130.6(3)	F(6)-P-F(2)	83.1(3)
C(9)-C(8)-H(8)	114.7	F(3A)-P-F(2)	85.7(5)
C(7)-C(8)-H(8)	114.7	F(3)-P-F(2)	92.3(5)
C(8)-C(9)-C(13)	124.6(3)	F(5)-P-F(2)	86.7(3)
C(8)-C(9)-C(10)	118.3(3)	F(1)-P-F(2)	178.41(16)
C(13)-C(9)-C(10)	117.0(3)	F(4A)-P-F(5A)	91.3(6)
C(12)-C(10)-C(11)	111.9(3)	F(3A)-P-F(5A)	172.5(6)
C(12)-C(10)-C(9)	112.2(3)	F(1)-P-F(5A)	83.9(2)
C(11)-C(10)-C(9)	110.7(3)	F(2)-P-F(5A)	94.6(2)
C(12)-C(10)-H(10)	107.2	F(6)-P-F(4)	177.2(5)
C(11)-C(10)-H(10)	107.2	F(3)-P-F(4)	86.1(5)
C(9)-C(10)-H(10)	107.2	F(5)-P-F(4)	89.8(5)
C(10)-C(11)-H(11A)	109.5	F(1)-P-F(4)	86.3(3)
C(10)-C(11)-H(11B)	109.5	F(2)-P-F(4)	94.4(3)
H(11A)-C(11)-H(11B)	109.5	F(4A)-P-F(6A)	173.7(7)
C(10)-C(11)-H(11C)	109.5	F(3A)-P-F(6A)	90.1(6)
H(11A)-C(11)-H(11C)	109.5	F(1)-P-F(6A)	87.8(3)
H(11B)-C(11)-H(11C)	109.5	F(2)-P-F(6A)	92.3(3)
C(10)-C(12)-H(12A)	109.5	F(5A)-P-F(6A)	82.4(5)

Table S27. Anisotropic displacement parameters ($Å^2$x 10^3) for **11d**. The anisotropic displacement factor exponent takes the form: $-2\pi^2$[h^2 $a^{*2}U^{11}$ + ... + 2 h k a* b* U^{12}]

	U^{11}	U^{22}	U^{33}	U^{23}	U^{13}	U^{12}
S(1)	76(1)	48(1)	40(1)	4(1)	12(1)	18(1)
C(1)	80(3)	70(3)	68(3)	6(2)	37(2)	9(2)
C(2)	118(4)	63(3)	48(3)	-16(2)	0(2)	10(3)
C(3)	46(2)	37(2)	39(2)	1(1)	2(1)	6(1)
C(4)	46(2)	42(2)	48(2)	2(2)	5(2)	13(2)
C(5)	35(2)	38(2)	39(2)	1(1)	-3(1)	3(1)
C(6)	46(2)	56(2)	44(2)	6(2)	-3(2)	16(2)
C(7)	35(2)	30(2)	38(2)	-1(1)	-6(1)	-1(1)
C(8)	31(2)	30(2)	39(2)	2(1)	-4(1)	-4(1)
C(9)	35(2)	30(2)	39(2)	0(1)	-3(1)	-1(1)
C(10)	43(2)	54(2)	44(2)	6(2)	7(1)	9(2)
C(11)	65(2)	69(3)	48(2)	-12(2)	6(2)	12(2)
C(12)	52(2)	71(3)	75(3)	16(2)	19(2)	0(2)
C(13)	40(2)	41(2)	48(2)	0(2)	1(1)	9(1)
C(14)	47(2)	41(2)	42(2)	3(2)	-5(1)	13(2)
C(15)	43(2)	34(2)	43(2)	-3(1)	-4(1)	4(1)
C(16)	75(3)	57(2)	37(2)	3(2)	-4(2)	21(2)
C(17)	44(2)	33(2)	32(2)	0(1)	-4(1)	-6(1)
P	49(1)	56(1)	31(1)	-5(1)	2(1)	7(1)
F(1)	94(2)	75(2)	64(2)	-31(1)	-21(1)	30(1)
F(2)	113(2)	80(2)	56(2)	-28(1)	9(1)	-12(2)
F(3)	168(11)	66(4)	108(9)	13(6)	78(8)	-5(8)
F(4)	62(4)	127(7)	98(6)	-19(5)	-35(4)	36(4)
F(5)	138(7)	77(4)	58(4)	3(3)	47(4)	-9(5)
F(6)	45(4)	72(5)	147(9)	-42(5)	-32(5)	27(3)
F(3A)	192(13)	90(6)	38(4)	13(4)	-41(7)	8(8)
F(4A)	53(4)	238(14)	84(6)	-60(7)	21(4)	-26(6)
F(5A)	151(7)	76(4)	39(3)	1(3)	-19(4)	5(5)
F(6A)	69(4)	150(9)	92(6)	-67(6)	25(4)	-31(6)

Table S28. Hydrogen coordinates (x 10^4) and isotropic displacement parameters (Å^{2}x 10 ^{3}) for **11d**.

	x	y	z	U(eq)
H(1A)	778	5531	4075	107
H(1B)	488	7143	4134	107
H(1C)	663	6196	4673	107
H(2A)	4533	8605	4671	115
H(2B)	3011	8219	5035	115
H(2C)	2650	8899	4439	115
H(4)	1877	8379	3435	55
H(6A)	2389	8056	1996	73
H(6B)	3367	9371	2245	73
H(6C)	1521	9040	2419	73
H(8)	5037	7067	2000	40
H(10)	6776	6351	1421	56
H(11A)	7483	4285	1017	91
H(11B)	5999	4013	1411	91
H(11C)	7876	3599	1610	91
H(12A)	9582	6195	1275	98
H(12B)	9973	5467	1860	98
H(12C)	9392	7035	1833	98
H(13)	8529	4423	2515	51
H(14)	8129	4161	3379	53
H(16A)	6469	5593	4372	85
H(16B)	7292	4198	4176	85
H(16C)	5308	4309	4201	85

Table S29. Torsion angles [°] for **11d**.

C(2)-S(1)-C(3)-C(4)	-57.0(4)
C(1)-S(1)-C(3)-C(4)	47.3(3)
C(2)-S(1)-C(3)-C(17)	127.4(4)
C(1)-S(1)-C(3)-C(17)	-128.3(3)
C(17)-C(3)-C(4)-C(5)	0.4(4)
S(1)-C(3)-C(4)-C(5)	-176.0(3)
C(3)-C(4)-C(5)-C(7)	-0.5(4)
C(3)-C(4)-C(5)-C(6)	-179.8(3)
C(4)-C(5)-C(7)-C(8)	180.0(3)
C(6)-C(5)-C(7)-C(8)	-0.8(5)
C(4)-C(5)-C(7)-C(17)	0.4(4)
C(6)-C(5)-C(7)-C(17)	179.6(3)
C(5)-C(7)-C(8)-C(9)	178.4(3)
C(17)-C(7)-C(8)-C(9)	-2.1(5)
C(7)-C(8)-C(9)-C(13)	0.2(5)
C(7)-C(8)-C(9)-C(10)	-178.9(3)
C(8)-C(9)-C(10)-C(12)	119.6(3)
C(13)-C(9)-C(10)-C(12)	-59.6(4)
C(8)-C(9)-C(10)-C(11)	-114.6(3)
C(13)-C(9)-C(10)-C(11)	66.2(4)
C(8)-C(9)-C(13)-C(14)	0.5(6)
C(10)-C(9)-C(13)-C(14)	179.6(3)
C(9)-C(13)-C(14)-C(15)	1.1(7)
C(13)-C(14)-C(15)-C(17)	-1.9(6)
C(13)-C(14)-C(15)-C(16)	178.8(4)
C(14)-C(15)-C(17)-C(3)	-177.6(3)
C(16)-C(15)-C(17)-C(3)	1.7(5)
C(14)-C(15)-C(17)-C(7)	0.1(5)
C(16)-C(15)-C(17)-C(7)	179.4(3)
C(4)-C(3)-C(17)-C(15)	177.9(3)
S(1)-C(3)-C(17)-C(15)	-6.0(6)
C(4)-C(3)-C(17)-C(7)	-0.2(4)
S(1)-C(3)-C(17)-C(7)	175.9(3)
C(8)-C(7)-C(17)-C(15)	2.2(5)
C(5)-C(7)-C(17)-C(15)	-178.3(3)
C(8)-C(7)-C(17)-C(3)	-179.6(3)
C(5)-C(7)-C(17)-C(3)	-0.1(3)

Table S30. Crystal data and structure refinement for **11e**.

Identification code	e15sel4	
CCDC Number	# 1440662	
Empirical formula	$C_{17} H_{21} F_6 O P S$	
Formula weight	418.37	
Temperature	150(2) K	
Wavelength	0.71073 Å	
Crystal system	Monoclinic	
Space group	$P2_1/c$	
Unit cell dimensions	a = 7.1045(2) Å	α= 90°.
	b = 10.0526(2) Å	β= 106.783(3)°.
	c = 25.7337(7) Å	γ = 90°.
Volume	1759.58(8) $Å^3$	
Z	4	
Density (calculated)	1.579 Mg/m^3	
Absorption coefficient	0.339 mm^{-1}	
F(000)	864	
Crystal size	0.400 x 0.200 x 0.100 mm^3	
Theta range for data collection	3.308 to 29.401°.	
Index ranges	-9<=h<=8, -11<=k<=13, -35<=l<=34	
Reflections collected	22345	
Independent reflections	4410 [R(int) = 0.0390]	
Completeness to theta = 25.242°	99.6 %	
Absorption correction	Semi-empirical from equivalents	
Max. and min. transmission	1.00000 and 0.67572	
Refinement method	Full-matrix least-squares on F^2	
Data / restraints / parameters	4410 / 0 / 257	
Goodness-of-fit on F^2	1.066	
Final R indices [I>2sigma(I)]	R1 = 0.0504, wR2 = 0.1251	
R indices (all data)	R1 = 0.0666, wR2 = 0.1347	
Extinction coefficient	n/a	
Largest diff. peak and hole	0.523 and -0.507 e.$Å^{-3}$	

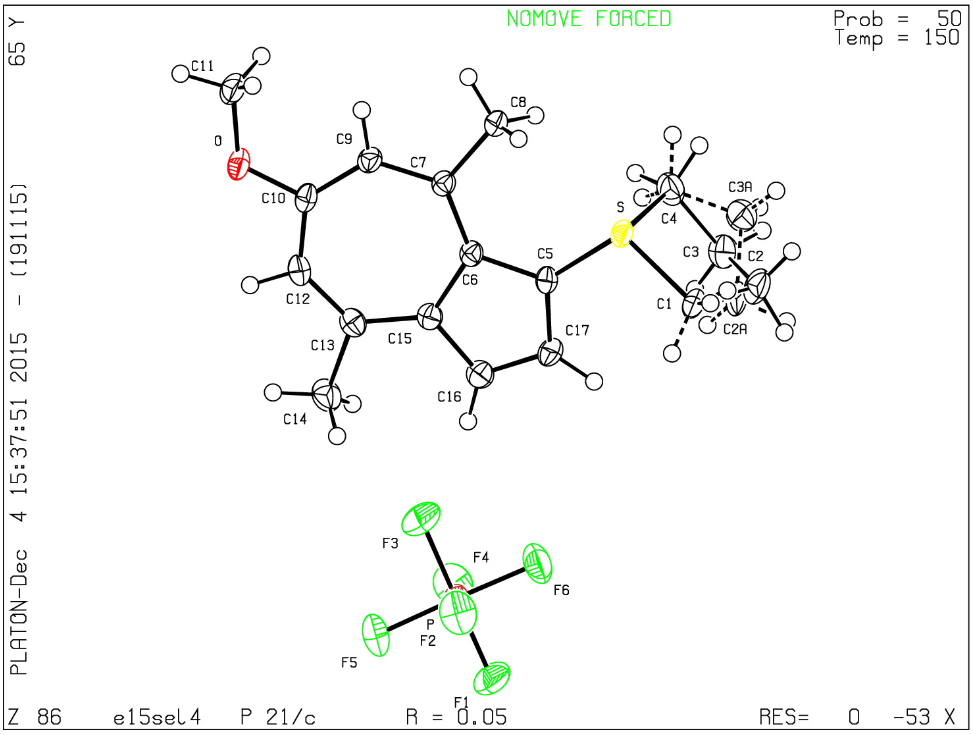

Table S31. Atomic coordinates (x 10^4) and equivalent isotropic displacement parameters ($Å^2$x 10^3) for **11e**. U(eq) is defined as one third of the trace of the orthogonalized U^{ij} tensor.

	x	y	z	U(eq)
S	2516(1)	7278(1)	3646(1)	23(1)
C(1)	2951(4)	7089(3)	2986(1)	29(1)
C(2)	1219(7)	7629(7)	2562(2)	35(1)
C(3)	-573(5)	7356(6)	2762(2)	32(1)
C(2A)	620(20)	7065(14)	2586(4)	29(3)
C(3A)	-422(17)	8156(19)	2812(5)	36(3)
C(4)	5(4)	7889(3)	3358(1)	33(1)
C(5)	2239(3)	5658(2)	3870(1)	21(1)
C(6)	2362(3)	5267(2)	4416(1)	20(1)
C(7)	2482(3)	6082(2)	4867(1)	20(1)
C(8)	2437(4)	7582(2)	4804(1)	26(1)
C(9)	2646(3)	5670(2)	5396(1)	22(1)
C(10)	2730(3)	4400(2)	5616(1)	22(1)
O	2946(2)	4232(2)	6154(1)	28(1)
C(11)	3094(4)	5372(3)	6501(1)	31(1)
C(12)	2581(3)	3165(2)	5354(1)	24(1)
C(13)	2322(3)	2877(2)	4816(1)	24(1)
C(14)	2147(4)	1416(2)	4664(1)	34(1)
C(15)	2226(3)	3797(2)	4392(1)	22(1)
C(16)	2022(3)	3404(2)	3857(1)	26(1)
C(17)	2027(3)	4525(2)	3544(1)	25(1)
P	5787(1)	582(1)	3376(1)	26(1)
F(1)	5812(3)	-258(2)	2852(1)	50(1)
F(2)	8050(2)	939(2)	3475(1)	48(1)
F(3)	5781(3)	1430(2)	3896(1)	53(1)
F(4)	3525(2)	215(2)	3282(1)	49(1)
F(5)	6407(3)	-721(2)	3736(1)	46(1)
F(6)	5180(3)	1875(2)	3005(1)	47(1)

Table S32. Bond lengths [Å] for **11e**.

S-C(5)	1.757(2)	C(7)-C(8)	1.516(3)
S-C(1)	1.821(2)	C(8)-H(8A)	0.9800
S-C(4)	1.829(3)	C(8)-H(8B)	0.9800
C(1)-C(2)	1.492(4)	C(8)-H(8C)	0.9800
C(1)-C(2A)	1.678(13)	C(9)-C(10)	1.390(3)
C(1)-H(1A)	0.9900	C(9)-H(9)	0.9500
C(1)-H(1B)	0.9900	C(10)-O	1.359(3)
C(1)-H(1C)	0.9900	C(10)-C(12)	1.403(3)
C(1)-H(1D)	0.9900	O-C(11)	1.438(3)
C(2)-C(3)	1.530(7)	C(11)-H(11A)	0.9800
C(2)-H(2A)	0.9900	C(11)-H(11B)	0.9800
C(2)-H(2B)	0.9900	C(11)-H(11C)	0.9800
C(3)-C(4)	1.564(5)	C(12)-C(13)	1.372(3)
C(3)-H(3A)	0.9900	C(12)-H(12)	0.9500
C(3)-H(3B)	0.9900	C(13)-C(15)	1.417(3)
C(2A)-C(3A)	1.531(19)	C(13)-C(14)	1.516(3)
C(2A)-H(2C)	0.9900	C(14)-H(14A)	0.9800
C(2A)-H(2D)	0.9900	C(14)-H(14B)	0.9800
C(3A)-C(4)	1.376(11)	C(14)-H(14C)	0.9800
C(3A)-H(3C)	0.9900	C(15)-C(16)	1.400(3)
C(3A)-H(3D)	0.9900	C(16)-C(17)	1.386(3)
C(4)-H(4A)	0.9900	C(16)-H(16)	0.9500
C(4)-H(4B)	0.9900	C(17)-H(17)	0.9500
C(4)-H(4C)	0.9900	P-F(3)	1.5874(17)
C(4)-H(4D)	0.9900	P-F(5)	1.5899(16)
C(5)-C(17)	1.397(3)	P-F(2)	1.5946(17)
C(5)-C(6)	1.437(3)	P-F(1)	1.5964(17)
C(6)-C(7)	1.402(3)	P-F(4)	1.5974(17)
C(6)-C(15)	1.481(3)	P-F(6)	1.5978(16)
C(7)-C(9)	1.396(3)		

Table S33. Bond angles [°] for **11e**.

C(5)-S-C(1)	105.96(11)	C(9)-C(7)-C(8)	113.13(19)
C(5)-S-C(4)	104.79(11)	C(6)-C(7)-C(8)	119.87(19)
C(1)-S-C(4)	93.74(12)	C(7)-C(8)-H(8A)	109.5
C(2)-C(1)-S	108.6(2)	C(7)-C(8)-H(8B)	109.5
C(2A)-C(1)-S	99.9(5)	H(8A)-C(8)-H(8B)	109.5
C(2)-C(1)-H(1A)	110.0	C(7)-C(8)-H(8C)	109.5
S-C(1)-H(1A)	110.0	H(8A)-C(8)-H(8C)	109.5
C(2)-C(1)-H(1B)	110.0	H(8B)-C(8)-H(8C)	109.5
S-C(1)-H(1B)	110.0	C(10)-C(9)-C(7)	130.6(2)
H(1A)-C(1)-H(1B)	108.4	C(10)-C(9)-H(9)	114.7
C(2A)-C(1)-H(1C)	111.8	C(7)-C(9)-H(9)	114.7
S-C(1)-H(1C)	111.8	O-C(10)-C(9)	120.4(2)
C(2A)-C(1)-H(1D)	111.8	O-C(10)-C(12)	110.55(19)
S-C(1)-H(1D)	111.8	C(9)-C(10)-C(12)	129.0(2)
H(1C)-C(1)-H(1D)	109.5	C(10)-O-C(11)	120.05(18)
C(1)-C(2)-C(3)	106.2(4)	O-C(11)-H(11A)	109.5
C(1)-C(2)-H(2A)	110.5	O-C(11)-H(11B)	109.5
C(3)-C(2)-H(2A)	110.5	H(11A)-C(11)-H(11B)	109.5
C(1)-C(2)-H(2B)	110.5	O-C(11)-H(11C)	109.5
C(3)-C(2)-H(2B)	110.5	H(11A)-C(11)-H(11C)	109.5
H(2A)-C(2)-H(2B)	108.7	H(11B)-C(11)-H(11C)	109.5
C(2)-C(3)-C(4)	105.0(4)	C(13)-C(12)-C(10)	129.9(2)
C(2)-C(3)-H(3A)	110.7	C(13)-C(12)-H(12)	115.1
C(4)-C(3)-H(3A)	110.7	C(10)-C(12)-H(12)	115.1
C(2)-C(3)-H(3B)	110.7	C(12)-C(13)-C(15)	126.9(2)
C(4)-C(3)-H(3B)	110.7	C(12)-C(13)-C(14)	116.2(2)
H(3A)-C(3)-H(3B)	108.8	C(15)-C(13)-C(14)	116.8(2)
C(3A)-C(2A)-C(1)	104.8(9)	C(13)-C(14)-H(14A)	109.5
C(3A)-C(2A)-H(2C)	110.8	C(13)-C(14)-H(14B)	109.5
C(1)-C(2A)-H(2C)	110.8	H(14A)-C(14)-H(14B)	109.5
C(3A)-C(2A)-H(2D)	110.8	C(13)-C(14)-H(14C)	109.5
C(1)-C(2A)-H(2D)	110.8	H(14A)-C(14)-H(14C)	109.5
H(2C)-C(2A)-H(2D)	108.9	H(14B)-C(14)-H(14C)	109.5
C(4)-C(3A)-C(2A)	105.1(10)	C(16)-C(15)-C(13)	122.8(2)
C(4)-C(3A)-H(3C)	110.7	C(16)-C(15)-C(6)	108.04(19)
C(2A)-C(3A)-H(3C)	110.7	C(13)-C(15)-C(6)	129.1(2)
C(4)-C(3A)-H(3D)	110.7	C(17)-C(16)-C(15)	109.0(2)
C(2A)-C(3A)-H(3D)	110.7	C(17)-C(16)-H(16)	125.5
H(3C)-C(3A)-H(3D)	108.8	C(15)-C(16)-H(16)	125.5
C(3A)-C(4)-S	112.4(5)	C(16)-C(17)-C(5)	109.3(2)
C(3)-C(4)-S	103.4(2)	C(16)-C(17)-H(17)	125.3
C(3)-C(4)-H(4A)	111.1	C(5)-C(17)-H(17)	125.3
S-C(4)-H(4A)	111.1	F(3)-P-F(5)	91.17(10)
C(3)-C(4)-H(4B)	111.1	F(3)-P-F(2)	89.58(11)
S-C(4)-H(4B)	111.1	F(5)-P-F(2)	89.74(10)
H(4A)-C(4)-H(4B)	109.0	F(3)-P-F(1)	179.28(11)
C(3A)-C(4)-H(4C)	109.1	F(5)-P-F(1)	89.25(10)
S-C(4)-H(4C)	109.1	F(2)-P-F(1)	89.83(10)
C(3A)-C(4)-H(4D)	109.1	F(3)-P-F(4)	90.29(10)
S-C(4)-H(4D)	109.1	F(5)-P-F(4)	89.76(10)
H(4C)-C(4)-H(4D)	107.9	F(2)-P-F(4)	179.49(11)
C(17)-C(5)-C(6)	109.29(19)	F(1)-P-F(4)	90.30(10)
C(17)-C(5)-S	124.08(17)	F(3)-P-F(6)	90.09(10)
C(6)-C(5)-S	126.42(17)	F(5)-P-F(6)	178.67(10)
C(7)-C(6)-C(5)	128.4(2)	F(2)-P-F(6)	89.86(10)
C(7)-C(6)-C(15)	127.2(2)	F(1)-P-F(6)	89.49(10)
C(5)-C(6)-C(15)	104.33(18)	F(4)-P-F(6)	90.64(10)
C(9)-C(7)-C(6)	127.0(2)		

Table S34. Anisotropic displacement parameters ($Å^2 x 10^3$) for **11e**. The anisotropic displacement factor exponent takes the form: $-2\pi^2[h^2 a^{*2}U^{11} + ... + 2 h k a^* b^* U^{12}]$

	U^{11}	U^{22}	U^{33}	U^{23}	U^{13}	U^{12}
S	27(1)	25(1)	14(1)	1(1)	3(1)	-1(1)
C(1)	36(1)	35(1)	16(1)	5(1)	10(1)	6(1)
C(2)	35(2)	49(3)	18(2)	4(2)	2(2)	4(2)
C(3)	26(2)	39(3)	26(2)	1(2)	-4(1)	4(2)
C(2A)	37(7)	30(6)	12(4)	3(4)	-6(4)	-7(5)
C(3A)	35(6)	39(9)	34(6)	5(5)	9(4)	11(5)
C(4)	32(1)	34(1)	35(1)	10(1)	12(1)	10(1)
C(5)	20(1)	24(1)	17(1)	3(1)	2(1)	1(1)
C(6)	17(1)	23(1)	17(1)	1(1)	1(1)	1(1)
C(7)	18(1)	24(1)	17(1)	0(1)	3(1)	1(1)
C(8)	35(1)	24(1)	16(1)	1(1)	6(1)	3(1)
C(9)	22(1)	25(1)	17(1)	-1(1)	3(1)	1(1)
C(10)	18(1)	30(1)	16(1)	4(1)	4(1)	1(1)
O	33(1)	33(1)	16(1)	5(1)	4(1)	0(1)
C(11)	34(1)	39(1)	18(1)	2(1)	6(1)	4(1)
C(12)	22(1)	26(1)	24(1)	8(1)	4(1)	1(1)
C(13)	19(1)	24(1)	26(1)	2(1)	2(1)	0(1)
C(14)	42(1)	24(1)	31(1)	0(1)	2(1)	-5(1)
C(15)	18(1)	24(1)	21(1)	0(1)	2(1)	0(1)
C(16)	26(1)	25(1)	23(1)	-2(1)	0(1)	0(1)
C(17)	28(1)	30(1)	16(1)	-2(1)	2(1)	0(1)
P	32(1)	26(1)	18(1)	1(1)	3(1)	1(1)
F(1)	77(1)	46(1)	29(1)	-12(1)	17(1)	-5(1)
F(2)	34(1)	52(1)	53(1)	7(1)	6(1)	-5(1)
F(3)	74(1)	55(1)	30(1)	-14(1)	14(1)	0(1)
F(4)	33(1)	51(1)	58(1)	3(1)	6(1)	-2(1)
F(5)	52(1)	41(1)	43(1)	20(1)	9(1)	6(1)
F(6)	56(1)	35(1)	40(1)	12(1)	0(1)	6(1)

Table S35. Hydrogen coordinates (x 10^4) and isotropic displacement parameters ($Å^2$x 10 ^{3}) for **11e**.

	x	y	z	U(eq)
H(1A)	4155	7577	2981	35
H(1B)	3136	6137	2915	35
H(1C)	3697	7849	2901	35
H(1D)	3648	6249	2962	35
H(2A)	1075	7183	2210	42
H(2B)	1374	8597	2515	42
H(3A)	-861	6391	2752	39
H(3B)	-1745	7828	2534	39
H(2C)	569	7255	2204	35
H(2D)	8	6187	2602	35
H(3C)	74	9046	2751	43
H(3D)	-1858	8126	2637	43
H(4A)	-882	7531	3558	40
H(4B)	-38	8873	3365	40
H(4C)	-930	7215	3416	40
H(4D)	-173	8710	3551	40
H(8A)	2449	7999	5149	38
H(8B)	1241	7844	4522	38
H(8C)	3593	7873	4699	38
H(9)	2712	6373	5648	26
H(11A)	3276	5076	6875	46
H(11B)	1888	5901	6379	46
H(11C)	4221	5917	6485	46
H(12)	2673	2412	5583	29
H(14A)	893	1259	4386	51
H(14B)	2202	880	4986	51
H(14C)	3233	1164	4520	51
H(16)	1900	2512	3729	31
H(17)	1905	4523	3166	30

Table S36. Torsion angles [°] for **11e**.

C(5)-S-C(1)-C(2)	112.1(3)
C(4)-S-C(1)-C(2)	5.6(3)
C(5)-S-C(1)-C(2A)	87.4(5)
C(4)-S-C(1)-C(2A)	-19.2(5)
S-C(1)-C(2)-C(3)	-32.8(6)
C(1)-C(2)-C(3)-C(4)	50.2(6)
S-C(1)-C(2A)-C(3A)	42.3(12)
C(1)-C(2A)-C(3A)-C(4)	-51.7(16)
C(2A)-C(3A)-C(4)-S	37.4(16)
C(2)-C(3)-C(4)-S	-44.3(5)
C(5)-S-C(4)-C(3A)	-117.0(9)
C(1)-S-C(4)-C(3A)	-9.5(9)
C(5)-S-C(4)-C(3)	-85.3(3)
C(1)-S-C(4)-C(3)	22.3(3)
C(1)-S-C(5)-C(17)	-11.0(2)
C(4)-S-C(5)-C(17)	87.4(2)
C(1)-S-C(5)-C(6)	163.25(19)
C(4)-S-C(5)-C(6)	-98.4(2)
C(17)-C(5)-C(6)-C(7)	-176.5(2)
S-C(5)-C(6)-C(7)	8.6(3)
C(17)-C(5)-C(6)-C(15)	0.6(2)
S-C(5)-C(6)-C(15)	-174.35(16)
C(5)-C(6)-C(7)-C(9)	-178.4(2)
C(15)-C(6)-C(7)-C(9)	5.1(4)
C(5)-C(6)-C(7)-C(8)	1.7(3)
C(15)-C(6)-C(7)-C(8)	-174.8(2)
C(6)-C(7)-C(9)-C(10)	-0.1(4)
C(8)-C(7)-C(9)-C(10)	179.8(2)
C(7)-C(9)-C(10)-O	178.2(2)
C(7)-C(9)-C(10)-C(12)	-3.2(4)
C(9)-C(10)-O-C(11)	-0.1(3)
C(12)-C(10)-O-C(11)	-178.95(19)
O-C(10)-C(12)-C(13)	179.0(2)
C(9)-C(10)-C(12)-C(13)	0.2(4)
C(10)-C(12)-C(13)-C(15)	3.0(4)
C(10)-C(12)-C(13)-C(14)	-177.8(2)
C(12)-C(13)-C(15)-C(16)	177.8(2)
C(14)-C(13)-C(15)-C(16)	-1.3(3)
C(12)-C(13)-C(15)-C(6)	-0.5(4)
C(14)-C(13)-C(15)-C(6)	-179.6(2)
C(7)-C(6)-C(15)-C(16)	176.7(2)
C(5)-C(6)-C(15)-C(16)	-0.5(2)
C(7)-C(6)-C(15)-C(13)	-4.8(4)
C(5)-C(6)-C(15)-C(13)	178.0(2)
C(13)-C(15)-C(16)-C(17)	-178.4(2)
C(6)-C(15)-C(16)-C(17)	0.2(3)
C(15)-C(16)-C(17)-C(5)	0.2(3)
C(6)-C(5)-C(17)-C(16)	-0.5(3)
S-C(5)-C(17)-C(16)	174.59(17)

Table S37. Crystal data and structure refinement for **11f**.

Identification code	e15sel1	
CCDC Number	#1437060	
Empirical formula	$C_{94} H_{80} F_{48} O_2 P_4 S_4$	
Formula weight	2405.70	
Temperature	150(2) K	
Wavelength	0.71073 Å	
Crystal system	Monoclinic	
Space group	$P2_1/c$	
Unit cell dimensions	a = 14.9286(8) Å	α= 90°.
	b = 9.4356(3) Å	β= 103.968(4)°.
	c = 18.2583(7) Å	γ = 90°.
Volume	2495.82(19) $Å^3$	
Z	1	
Density (calculated)	1.601 Mg/m^3	
Absorption coefficient	0.298 mm^{-1}	
F(000)	1216	
Crystal size	0.600 x 0.200 x 0.050 mm^3	
Theta range for data collection	3.512 to 29.385°.	
Index ranges	-20<=h<=18, -12<=k<=12, -24<=l<=24	
Reflections collected	21571	
Independent reflections	6038 [R(int) = 0.0339]	
Completeness to theta = 25.242°	99.7 %	
Absorption correction	Semi-empirical from equivalents	
Max. and min. transmission	1.00000 and 0.77881	
Refinement method	Full-matrix least-squares on F^2	
Data / restraints / parameters	6038 / 126 / 457	
Goodness-of-fit on F^2	1.036	
Final R indices [I>2sigma(I)]	R1 = 0.0569, wR2 = 0.1288	
R indices (all data)	R1 = 0.0853, wR2 = 0.1440	
Extinction coefficient	n/a	
Largest diff. peak and hole	1.334 and -0.449 e.$Å^{-3}$	

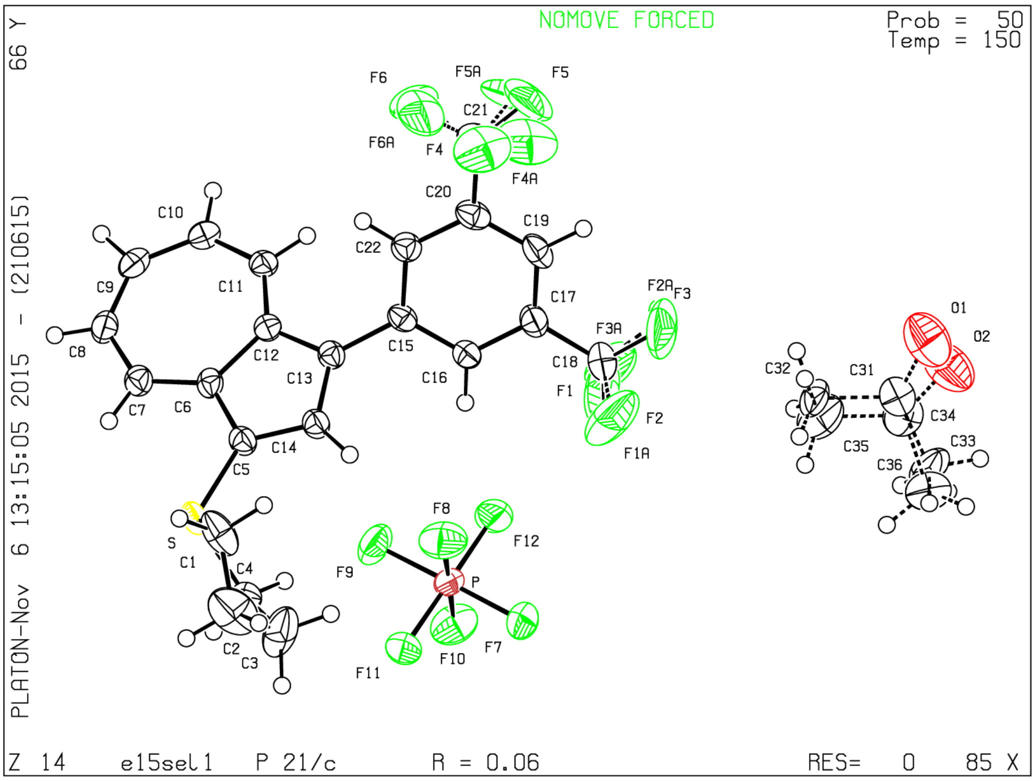

Table S38. Atomic coordinates (x 10^4) and equivalent isotropic displacement parameters ($Å^2$x 10^3) for **11f**. U(eq) is defined as one third of the trace of the orthogonalized U^{ij} tensor.

	x	y	z	U(eq)
S	6545(1)	-2105(1)	2979(1)	32(1)
C(1)	7728(2)	-2388(4)	2902(2)	50(1)
C(2)	7713(3)	-1994(5)	2129(2)	75(1)
C(3)	7185(3)	-585(4)	1970(3)	76(1)
C(4)	6295(2)	-731(3)	2248(2)	39(1)
C(5)	6649(2)	-1249(3)	3838(1)	29(1)
C(6)	6141(2)	-1661(2)	4360(1)	27(1)
C(7)	5483(2)	-2725(3)	4259(1)	32(1)
C(8)	4929(2)	-3127(3)	4736(2)	34(1)
C(9)	4892(2)	-2549(3)	5427(2)	34(1)
C(10)	5390(2)	-1439(3)	5829(1)	32(1)
C(11)	6084(2)	-628(3)	5648(1)	28(1)
C(12)	6450(2)	-716(2)	5018(1)	25(1)
C(13)	7135(2)	195(3)	4856(1)	27(1)
C(14)	7239(2)	-121(3)	4136(1)	29(1)
C(15)	7683(2)	1279(3)	5353(1)	28(1)
C(16)	7787(2)	2629(3)	5080(1)	31(1)
C(17)	8346(2)	3626(3)	5536(2)	35(1)
C(18)	8428(3)	5077(4)	5226(2)	55(1)
C(19)	8822(2)	3283(3)	6267(2)	38(1)
C(20)	8720(2)	1932(3)	6537(1)	36(1)
C(21)	9278(2)	1543(5)	7312(2)	55(1)
C(22)	8158(2)	944(3)	6093(1)	32(1)
F(1)	7613(11)	5677(17)	4905(16)	109(6)
F(2)	8905(18)	5020(20)	4684(13)	105(6)
F(3)	8800(15)	5988(14)	5741(7)	82(4)
F(1A)	8590(13)	5104(14)	4580(7)	78(3)
F(2A)	9132(14)	5813(16)	5669(10)	121(5)
F(3A)	7680(10)	5786(13)	5168(7)	87(4)
F(4)	10156(4)	1033(16)	7262(5)	81(3)
F(5)	9436(12)	2465(8)	7809(5)	97(4)
F(6)	8977(6)	347(15)	7598(7)	70(2)
F(4A)	10125(5)	1990(30)	7402(5)	89(5)
F(5A)	8986(9)	2471(16)	7817(6)	88(3)
F(6A)	9189(15)	353(18)	7510(10)	103(5)
P	6397(1)	3510(1)	2696(1)	33(1)
F(7)	7006(1)	4700(2)	2430(1)	52(1)
F(8)	7293(1)	2700(2)	3141(1)	61(1)
F(9)	5767(1)	2324(2)	2950(1)	56(1)
F(10)	5491(1)	4306(2)	2244(1)	60(1)
F(11)	6411(1)	2611(2)	1957(1)	50(1)
F(12)	6378(2)	4398(2)	3433(1)	60(1)
O(1)	10648(16)	10430(20)	5996(11)	86(6)
C(31)	10206(19)	10170(20)	5361(12)	42(5)
C(32)	9810(30)	8680(30)	5210(20)	50(8)
C(33)	9940(30)	11130(50)	4680(20)	62(11)
O(2)	10584(18)	10960(20)	5751(11)	88(6)
C(34)	10140(30)	10360(30)	5176(13)	56(8)
C(35)	9780(30)	8850(30)	5030(20)	72(12)
C(36)	9920(30)	11150(40)	4436(14)	62(7)

Table S39. Bond lengths [Å] for **11f**.

S-C(5)	1.736(2)	C(18)-F(3)	1.295(11)
S-C(1)	1.826(3)	C(18)-F(1)	1.342(16)
S-C(4)	1.834(3)	C(18)-F(2A)	1.353(12)
C(1)-C(2)	1.454(5)	C(18)-F(2)	1.35(2)
C(1)-H(1A)	0.9900	C(19)-C(20)	1.388(4)
C(1)-H(1B)	0.9900	C(19)-H(19)	0.9500
C(2)-C(3)	1.536(6)	C(20)-C(22)	1.380(4)
C(2)-H(2A)	0.9900	C(20)-C(21)	1.504(4)
C(2)-H(2B)	0.9900	C(21)-F(6A)	1.196(17)
C(3)-C(4)	1.538(4)	C(21)-F(5)	1.238(8)
C(3)-H(3A)	0.9900	C(21)-F(4A)	1.305(10)
C(3)-H(3B)	0.9900	C(21)-F(6)	1.363(12)
C(4)-H(4A)	0.9900	C(21)-F(5A)	1.414(12)
C(4)-H(4B)	0.9900	C(21)-F(4)	1.419(9)
C(5)-C(14)	1.404(3)	C(22)-H(22)	0.9500
C(5)-C(6)	1.410(3)	P-F(8)	1.5841(19)
C(6)-C(7)	1.386(3)	P-F(12)	1.5915(18)
C(6)-C(12)	1.478(3)	P-F(7)	1.5917(18)
C(7)-C(8)	1.390(4)	P-F(10)	1.5921(19)
C(7)-H(7)	0.9500	P-F(11)	1.5983(17)
C(8)-C(9)	1.388(4)	P-F(9)	1.6004(18)
C(8)-H(8)	0.9500	O(1)-C(31)	1.212(16)
C(9)-C(10)	1.387(4)	C(31)-C(33)	1.510(19)
C(9)-H(9)	0.9500	C(31)-C(32)	1.519(18)
C(10)-C(11)	1.391(4)	C(32)-H(32A)	0.9800
C(10)-H(10)	0.9500	C(32)-H(32B)	0.9800
C(11)-C(12)	1.390(3)	C(32)-H(32C)	0.9800
C(11)-H(11)	0.9500	C(33)-H(33A)	0.9800
C(12)-C(13)	1.420(3)	C(33)-H(33B)	0.9800
C(13)-C(14)	1.393(3)	C(33)-H(33C)	0.9800
C(13)-C(15)	1.477(3)	O(2)-C(34)	1.234(17)
C(14)-H(14)	0.9500	C(34)-C(36)	1.506(18)
C(15)-C(16)	1.390(4)	C(34)-C(35)	1.524(19)
C(15)-C(22)	1.402(3)	C(35)-H(35A)	0.9800
C(16)-C(17)	1.392(4)	C(35)-H(35B)	0.9800
C(16)-H(16)	0.9500	C(35)-H(35C)	0.9800
C(17)-C(19)	1.389(4)	C(36)-H(36A)	0.9800
C(17)-C(18)	1.499(4)	C(36)-H(36B)	0.9800
C(18)-F(1A)	1.262(13)	C(36)-H(36C)	0.9800
C(18)-F(3A)	1.283(13)		

Table S40. Bond angles [°] for **11f**.

C(5)-S-C(1)	105.09(13)	C(16)-C(15)-C(13)	120.5(2)
C(5)-S-C(4)	106.61(12)	C(22)-C(15)-C(13)	120.8(2)
C(1)-S-C(4)	94.46(15)	C(15)-C(16)-C(17)	120.5(2)
C(2)-C(1)-S	104.4(2)	C(15)-C(16)-H(16)	119.8
C(2)-C(1)-H(1A)	110.9	C(17)-C(16)-H(16)	119.8
S-C(1)-H(1A)	110.9	C(19)-C(17)-C(16)	120.7(3)
C(2)-C(1)-H(1B)	110.9	C(19)-C(17)-C(18)	120.4(3)
S-C(1)-H(1B)	110.9	C(16)-C(17)-C(18)	118.9(2)
H(1A)-C(1)-H(1B)	108.9	F(1A)-C(18)-F(3A)	105.7(9)
C(1)-C(2)-C(3)	107.0(3)	F(3)-C(18)-F(1)	102.9(10)
C(1)-C(2)-H(2A)	110.3	F(1A)-C(18)-F(2A)	104.6(9)
C(3)-C(2)-H(2A)	110.3	F(3A)-C(18)-F(2A)	108.5(9)
C(1)-C(2)-H(2B)	110.3	F(3)-C(18)-F(2)	110.3(11)
C(3)-C(2)-H(2B)	110.3	F(1)-C(18)-F(2)	106.2(12)
H(2A)-C(2)-H(2B)	108.6	F(1A)-C(18)-C(17)	115.0(6)
C(2)-C(3)-C(4)	107.8(3)	F(3A)-C(18)-C(17)	111.1(6)
C(2)-C(3)-H(3A)	110.2	F(3)-C(18)-C(17)	113.1(7)
C(4)-C(3)-H(3A)	110.2	F(1)-C(18)-C(17)	113.7(7)
C(2)-C(3)-H(3B)	110.2	F(2A)-C(18)-C(17)	111.5(6)
C(4)-C(3)-H(3B)	110.2	F(2)-C(18)-C(17)	110.2(10)
H(3A)-C(3)-H(3B)	108.5	C(20)-C(19)-C(17)	118.7(2)
C(3)-C(4)-S	105.2(2)	C(20)-C(19)-H(19)	120.6
C(3)-C(4)-H(4A)	110.7	C(17)-C(19)-H(19)	120.6
S-C(4)-H(4A)	110.7	C(22)-C(20)-C(19)	121.0(2)
C(3)-C(4)-H(4B)	110.7	C(22)-C(20)-C(21)	120.5(3)
S-C(4)-H(4B)	110.7	C(19)-C(20)-C(21)	118.4(3)
H(4A)-C(4)-H(4B)	108.8	F(6A)-C(21)-F(4A)	116.2(10)
C(14)-C(5)-C(6)	109.6(2)	F(5)-C(21)-F(6)	108.9(9)
C(14)-C(5)-S	127.91(19)	F(6A)-C(21)-F(5A)	108.1(12)
C(6)-C(5)-S	122.46(19)	F(4A)-C(21)-F(5A)	99.1(7)
C(7)-C(6)-C(5)	126.1(2)	F(5)-C(21)-F(4)	105.7(5)
C(7)-C(6)-C(12)	128.2(2)	F(6)-C(21)-F(4)	98.0(7)
C(5)-C(6)-C(12)	105.7(2)	F(6A)-C(21)-C(20)	115.9(8)
C(6)-C(7)-C(8)	128.5(2)	F(5)-C(21)-C(20)	118.8(5)
C(6)-C(7)-H(7)	115.7	F(4A)-C(21)-C(20)	109.8(5)
C(8)-C(7)-H(7)	115.7	F(6)-C(21)-C(20)	113.8(6)
C(9)-C(8)-C(7)	128.5(3)	F(5A)-C(21)-C(20)	105.9(6)
C(9)-C(8)-H(8)	115.7	F(4)-C(21)-C(20)	109.4(4)
C(7)-C(8)-H(8)	115.7	C(20)-C(22)-C(15)	120.5(3)
C(10)-C(9)-C(8)	130.1(2)	C(20)-C(22)-H(22)	119.7
C(10)-C(9)-H(9)	114.9	C(15)-C(22)-H(22)	119.7
C(8)-C(9)-H(9)	114.9	F(8)-P-F(12)	90.34(11)
C(9)-C(10)-C(11)	128.6(2)	F(8)-P-F(7)	91.32(11)
C(9)-C(10)-H(10)	115.7	F(12)-P-F(7)	90.81(10)
C(11)-C(10)-H(10)	115.7	F(8)-P-F(10)	179.29(13)
C(12)-C(11)-C(10)	128.9(2)	F(12)-P-F(10)	90.18(11)
C(12)-C(11)-H(11)	115.6	F(7)-P-F(10)	89.15(11)
C(10)-C(11)-H(11)	115.6	F(8)-P-F(11)	89.70(11)
C(11)-C(12)-C(13)	125.6(2)	F(12)-P-F(11)	179.63(11)
C(11)-C(12)-C(6)	127.0(2)	F(7)-P-F(11)	89.55(9)
C(13)-C(12)-C(6)	107.2(2)	F(10)-P-F(11)	89.77(11)
C(14)-C(13)-C(12)	108.5(2)	F(8)-P-F(9)	89.84(11)
C(14)-C(13)-C(15)	123.8(2)	F(12)-P-F(9)	89.52(10)
C(12)-C(13)-C(15)	127.7(2)	F(7)-P-F(9)	178.79(12)
C(13)-C(14)-C(5)	109.0(2)	F(10)-P-F(9)	89.69(11)
C(13)-C(14)-H(14)	125.5	F(11)-P-F(9)	90.11(10)
C(5)-C(14)-H(14)	125.5	O(1)-C(31)-C(33)	129(3)
C(16)-C(15)-C(22)	118.6(2)	O(1)-C(31)-C(32)	117(2)

C(33)-C(31)-C(32)	113(3)	O(2)-C(34)-C(35)	132(3)
C(31)-C(32)-H(32A)	109.5	C(36)-C(34)-C(35)	108(2)
C(31)-C(32)-H(32B)	109.5	C(34)-C(35)-H(35A)	109.5
H(32A)-C(32)-H(32B)	109.5	C(34)-C(35)-H(35B)	109.5
C(31)-C(32)-H(32C)	109.5	H(35A)-C(35)-H(35B)	109.5
H(32A)-C(32)-H(32C)	109.5	C(34)-C(35)-H(35C)	109.5
H(32B)-C(32)-H(32C)	109.5	H(35A)-C(35)-H(35C)	109.5
C(31)-C(33)-H(33A)	109.5	H(35B)-C(35)-H(35C)	109.5
C(31)-C(33)-H(33B)	109.5	C(34)-C(36)-H(36A)	109.5
H(33A)-C(33)-H(33B)	109.5	C(34)-C(36)-H(36B)	109.5
C(31)-C(33)-H(33C)	109.5	H(36A)-C(36)-H(36B)	109.5
H(33A)-C(33)-H(33C)	109.5	C(34)-C(36)-H(36C)	109.5
H(33B)-C(33)-H(33C)	109.5	H(36A)-C(36)-H(36C)	109.5
O(2)-C(34)-C(36)	120(3)	H(36B)-C(36)-H(36C)	109.5

Table S41. Anisotropic displacement parameters ($Å^2$x 10^3) for **11f**. The anisotropic displacement factor exponent takes the form: $-2\pi^2[h^2 a^{*2}U^{11} + ... + 2 h k a^* b^* U^{12}]$

	U^{11}	U^{22}	U^{33}	U^{23}	U^{13}	U^{12}
S	34(1)	29(1)	36(1)	-12(1)	11(1)	-6(1)
C(1)	31(1)	58(2)	63(2)	-32(2)	13(1)	-1(1)
C(2)	52(2)	108(4)	74(3)	-30(2)	31(2)	-10(2)
C(3)	111(3)	47(2)	100(3)	-14(2)	85(3)	-20(2)
C(4)	50(2)	40(2)	30(1)	-6(1)	16(1)	-10(1)
C(5)	32(1)	26(1)	29(1)	-5(1)	7(1)	-1(1)
C(6)	28(1)	21(1)	31(1)	1(1)	4(1)	2(1)
C(7)	35(1)	25(1)	33(1)	1(1)	4(1)	1(1)
C(8)	34(1)	28(1)	38(1)	5(1)	4(1)	-6(1)
C(9)	33(1)	35(1)	35(1)	12(1)	8(1)	-1(1)
C(10)	32(1)	34(1)	29(1)	6(1)	8(1)	3(1)
C(11)	31(1)	26(1)	24(1)	2(1)	2(1)	3(1)
C(12)	25(1)	22(1)	26(1)	3(1)	1(1)	4(1)
C(13)	27(1)	24(1)	28(1)	-1(1)	5(1)	1(1)
C(14)	31(1)	27(1)	30(1)	-4(1)	8(1)	-2(1)
C(15)	26(1)	32(1)	25(1)	-5(1)	7(1)	1(1)
C(16)	35(1)	32(1)	27(1)	-4(1)	8(1)	-4(1)
C(17)	37(1)	33(1)	36(1)	-8(1)	11(1)	-7(1)
C(18)	71(2)	42(2)	50(2)	-9(2)	12(2)	-23(2)
C(19)	32(1)	45(2)	37(1)	-16(1)	8(1)	-11(1)
C(20)	27(1)	50(2)	29(1)	-4(1)	3(1)	-2(1)
C(21)	38(2)	79(3)	40(2)	-2(2)	-6(1)	-12(2)
C(22)	30(1)	34(1)	30(1)	-1(1)	7(1)	-1(1)
F(1)	89(7)	45(5)	161(12)	36(7)	-32(8)	-15(5)
F(2)	137(12)	83(6)	129(12)	32(6)	96(10)	5(7)
F(3)	155(12)	40(4)	53(5)	-24(3)	30(6)	-55(6)
F(1A)	135(9)	51(4)	62(4)	0(3)	49(5)	-31(5)
F(2A)	126(8)	77(6)	123(8)	25(5)	-40(5)	-71(5)
F(3A)	131(9)	49(4)	96(6)	27(4)	56(6)	35(5)
F(4)	34(2)	124(7)	77(4)	26(4)	-4(2)	8(3)
F(5)	138(9)	76(3)	46(3)	-15(2)	-41(5)	-1(5)
F(6)	46(3)	107(6)	48(3)	33(3)	-7(2)	-21(3)
F(4A)	30(3)	157(13)	69(4)	9(6)	-10(3)	4(5)
F(5A)	72(6)	156(8)	27(3)	-16(3)	-3(4)	25(5)
F(6A)	141(12)	66(7)	62(7)	14(5)	-52(7)	2(8)
P	42(1)	31(1)	28(1)	3(1)	11(1)	-1(1)
F(7)	62(1)	46(1)	51(1)	-1(1)	21(1)	-18(1)
F(8)	61(1)	69(1)	49(1)	12(1)	4(1)	19(1)
F(9)	78(1)	39(1)	65(1)	3(1)	44(1)	-10(1)
F(10)	49(1)	69(1)	63(1)	20(1)	13(1)	14(1)
F(11)	68(1)	49(1)	36(1)	-9(1)	20(1)	-10(1)
F(12)	106(2)	41(1)	40(1)	-7(1)	31(1)	-3(1)
O(1)	65(9)	94(12)	95(12)	-25(9)	10(9)	-9(9)
C(31)	29(7)	46(8)	52(8)	-7(7)	13(7)	4(6)
C(32)	53(14)	35(9)	51(15)	20(10)	-10(10)	-12(10)
C(33)	63(17)	68(17)	60(20)	18(14)	31(16)	-17(12)
O(2)	72(9)	102(13)	76(10)	-20(9)	-11(8)	0(10)
C(34)	47(10)	61(12)	63(11)	1(8)	20(8)	-2(8)
C(35)	77(17)	58(16)	80(20)	21(13)	22(16)	17(12)
C(36)	62(12)	71(13)	54(13)	7(11)	17(11)	5(10)

Table S42. Hydrogen coordinates (x 10^4) and isotropic displacement parameters ($Å^2$x 10^3) for **11f**.

	x	y	z	U(eq)
H(1A)	8165	-1784	3265	60
H(1B)	7911	-3393	2998	60
H(2A)	7399	-2735	1775	90
H(2B)	8350	-1879	2067	90
H(3A)	7032	-377	1423	91
H(3B)	7568	199	2239	91
H(4A)	5771	-1017	1830	47
H(4B)	6140	177	2460	47
H(7)	5399	-3250	3803	38
H(8)	4523	-3898	4567	41
H(9)	4459	-2981	5662	41
H(10)	5235	-1200	6289	38
H(11)	6345	83	6005	33
H(14)	7643	352	3887	35
H(16)	7475	2873	4579	37
H(19)	9209	3959	6575	46
H(22)	8091	29	6291	38
H(32A)	10046	8243	4806	75
H(32B)	9136	8736	5053	75
H(32C)	9995	8112	5669	75
H(33A)	9291	11000	4436	93
H(33B)	10321	10902	4328	93
H(33C)	10053	12120	4846	93
H(35A)	9821	8558	4521	108
H(35B)	9131	8819	5056	108
H(35C)	10146	8215	5404	108
H(36A)	9666	10485	4025	92
H(36B)	10487	11574	4352	92
H(36C)	9469	11894	4449	92

Table S43. Torsion angles [°] for **11f**.

C(5)-S-C(1)-C(2)	-135.7(3)
C(4)-S-C(1)-C(2)	-27.1(3)
S-C(1)-C(2)-C(3)	45.8(3)
C(1)-C(2)-C(3)-C(4)	-47.2(4)
C(2)-C(3)-C(4)-S	24.8(4)
C(5)-S-C(4)-C(3)	107.9(2)
C(1)-S-C(4)-C(3)	0.7(2)
C(1)-S-C(5)-C(14)	44.2(3)
C(4)-S-C(5)-C(14)	-55.2(3)
C(1)-S-C(5)-C(6)	-133.6(2)
C(4)-S-C(5)-C(6)	126.9(2)
C(14)-C(5)-C(6)-C(7)	179.1(2)
S-C(5)-C(6)-C(7)	-2.7(4)
C(14)-C(5)-C(6)-C(12)	-0.2(3)
S-C(5)-C(6)-C(12)	177.99(17)
C(5)-C(6)-C(7)-C(8)	-176.4(3)
C(12)-C(6)-C(7)-C(8)	2.7(4)
C(6)-C(7)-C(8)-C(9)	0.8(5)
C(7)-C(8)-C(9)-C(10)	-0.4(5)
C(8)-C(9)-C(10)-C(11)	-2.0(5)
C(9)-C(10)-C(11)-C(12)	0.7(4)
C(10)-C(11)-C(12)-C(13)	177.1(2)
C(10)-C(11)-C(12)-C(6)	3.7(4)
C(7)-C(6)-C(12)-C(11)	-5.7(4)
C(5)-C(6)-C(12)-C(11)	173.6(2)
C(7)-C(6)-C(12)-C(13)	179.9(2)
C(5)-C(6)-C(12)-C(13)	-0.8(3)
C(11)-C(12)-C(13)-C(14)	-173.0(2)
C(6)-C(12)-C(13)-C(14)	1.5(3)
C(11)-C(12)-C(13)-C(15)	8.7(4)
C(6)-C(12)-C(13)-C(15)	-176.8(2)
C(12)-C(13)-C(14)-C(5)	-1.7(3)
C(15)-C(13)-C(14)-C(5)	176.8(2)
C(6)-C(5)-C(14)-C(13)	1.1(3)
S-C(5)-C(14)-C(13)	-176.90(19)
C(14)-C(13)-C(15)-C(16)	48.7(4)
C(12)-C(13)-C(15)-C(16)	-133.2(3)
C(14)-C(13)-C(15)-C(22)	-127.2(3)
C(12)-C(13)-C(15)-C(22)	50.9(4)
C(22)-C(15)-C(16)-C(17)	-0.5(4)
C(13)-C(15)-C(16)-C(17)	-176.5(2)
C(15)-C(16)-C(17)-C(19)	1.2(4)
C(15)-C(16)-C(17)-C(18)	-179.0(3)
C(19)-C(17)-C(18)-F(1A)	131.7(10)
C(16)-C(17)-C(18)-F(1A)	-48.1(10)
C(19)-C(17)-C(18)-F(3A)	-108.3(8)
C(16)-C(17)-C(18)-F(3A)	71.9(8)
C(19)-C(17)-C(18)-F(3)	-13.7(11)
C(16)-C(17)-C(18)-F(3)	166.4(10)
C(19)-C(17)-C(18)-F(1)	-130.6(14)
C(16)-C(17)-C(18)-F(1)	49.5(14)
C(19)-C(17)-C(18)-F(2A)	12.9(13)
C(16)-C(17)-C(18)-F(2A)	-167.0(13)
C(19)-C(17)-C(18)-F(2)	110.3(13)
C(16)-C(17)-C(18)-F(2)	-69.6(13)
C(16)-C(17)-C(19)-C(20)	-0.9(4)
C(18)-C(17)-C(19)-C(20)	179.3(3)
C(17)-C(19)-C(20)-C(22)	-0.1(4)

C(17)-C(19)-C(20)-C(21)	176.7(3)
C(22)-C(20)-C(21)-F(6A)	-0.9(15)
C(19)-C(20)-C(21)-F(6A)	-177.7(14)
C(22)-C(20)-C(21)-F(5)	-148.3(10)
C(19)-C(20)-C(21)-F(5)	34.9(11)
C(22)-C(20)-C(21)-F(4A)	133.2(12)
C(19)-C(20)-C(21)-F(4A)	-43.6(12)
C(22)-C(20)-C(21)-F(6)	-18.0(7)
C(19)-C(20)-C(21)-F(6)	165.2(6)
C(22)-C(20)-C(21)-F(5A)	-120.7(7)
C(19)-C(20)-C(21)-F(5A)	62.5(7)
C(22)-C(20)-C(21)-F(4)	90.4(7)
C(19)-C(20)-C(21)-F(4)	-86.4(7)
C(19)-C(20)-C(22)-C(15)	0.8(4)
C(21)-C(20)-C(22)-C(15)	-175.9(3)
C(16)-C(15)-C(22)-C(20)	-0.5(4)
C(13)-C(15)-C(22)-C(20)	175.5(2)

[1] Pangborn, A. B.; Giardello, M. A.; Grubbs, R. H.; Rosen, R. K.; Timmers, F. J., *J. Organomet. Chem.* **1996**, *15*, 1518.

[2] Nefedov, V. A.; German, N. A.; Lutsenko, A. I.; Nikishin, G. I. *Zhurnal Organicheskoi Khimii* **1987**, *23*, 172-181; Nefedov, V. A.; German, N. A.; Lutsenko, A. I.; Nikishin, G. I. , *J. Org. Soc. Chem. USSR* (*Engl*), **1987,** 23, 154-162.

[3] Murai, M.; Takami, K.; Takeshima, H.; Takai, K., *Org. Lett.* **2015**, *17*, 1798-1801.

[4] Dubovik, J.; Bredihhin, A., *Synthesis* **2015**, *47*, 538-548.

[5] Farrugia, L. J., *J. Appl. Crystallogr.*, **2012**, *45*, 849-854.
